# Supplementary material for: Lactiplantibacillus plantarum K8 lysates regulate hypoxia-induced gene expression
Source: Sci Rep. 2024 Mar 15;14:6275. doi: 10.1038/s41598-024-56958-7 (PMC10943017; doi:10.1038/s41598-024-56958-7)

Lactiplantibacillus plantarum K8 lysates regulate hypoxia-induced gene expression

Authors: Jaehyeon Jeong[1], Byeong-Hee Kang[2], Sangmin Ju[2], Na Yeon Park[3], Deukyeong Kim[2], Ngoc Thi Bao Dinh[1], Jeongho Lee[2], Chang Yun Rhee[2], Dong-Hyung Cho[3,4,5], Hangeun Kim[6], Dae Kyun Chung[7], Heeyoun Bunch[1,2]*

*Correspondence to: HB at heeyounbunch@gmail.com.

This PDF contains:

Supplementary Figures 1–3

Supplementary Tables 1–4

Uncropped Images for Figs. 1C, 2A, 5B, and 6B

Fig. S1.

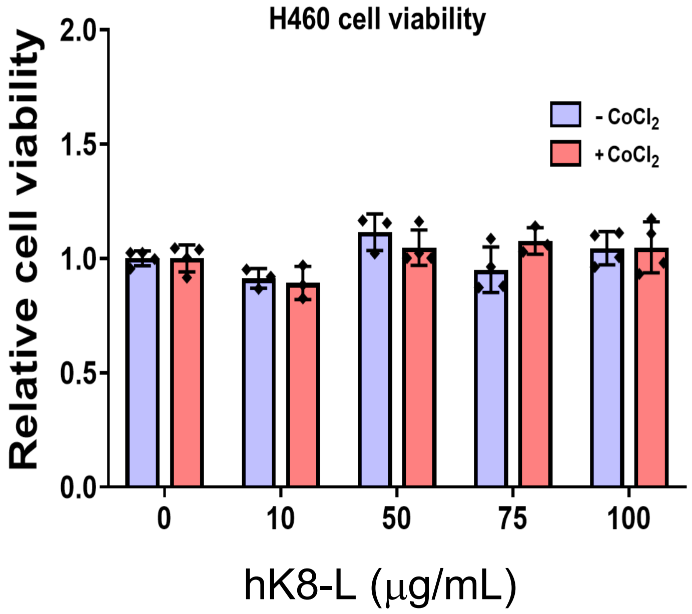

Supplementary Figure 1. Cytotoxicity test results with hK8-L using the WST assay.

$CoCl_2$ was supplemented to a final concentration of 200 μM when indicated as +$CoCl_2$. Cells were treated with the same volume of H_2O when indicated as -$CoCl_2$.

Fig. S2.

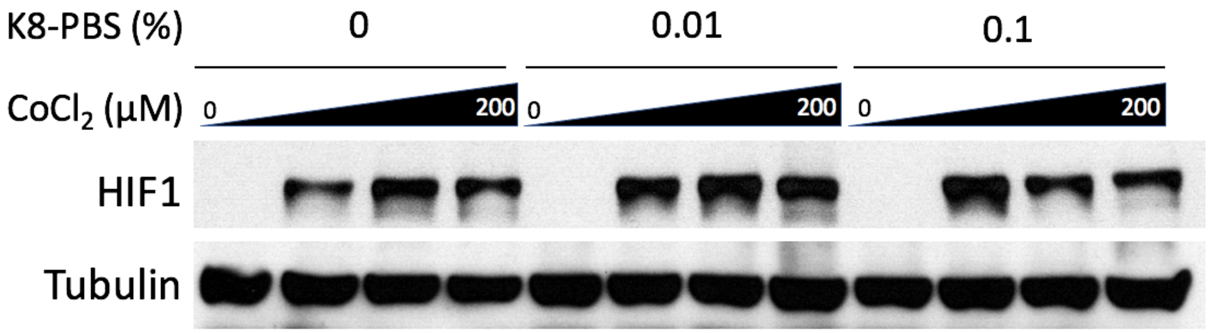

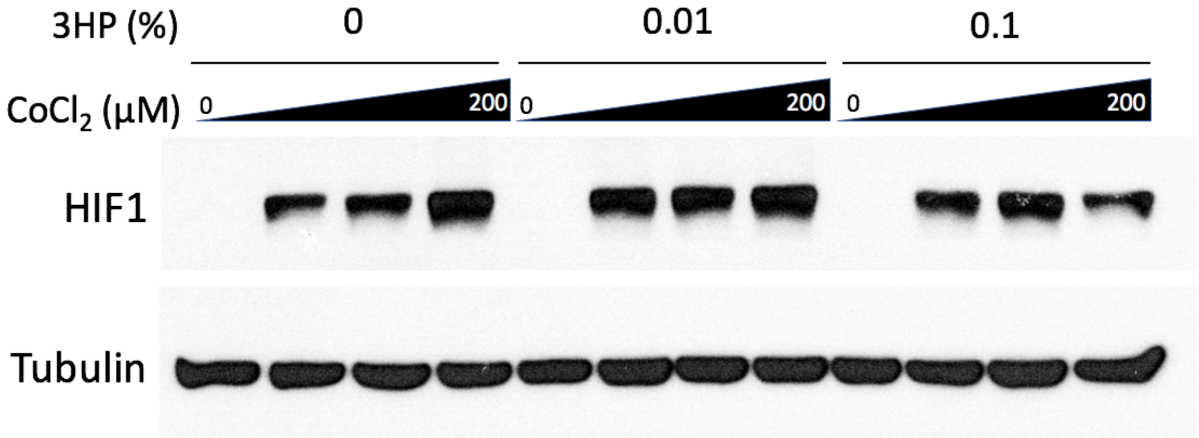

Supplementary Figure 2. PBS-soluble K8 and 3-hydroxypropionic acid don't have the HIF1α destabilizing effect.

PBS-soluble K8 (K8-PBS, upper panel) and 3-hydropropionic acid (3HP, bottom panel) were supplemented to SH-SY5Y cells. HIF1α (HIF1) was quantified through immunoblotting. Tubulin was used as a loading control.

Fig. S3.

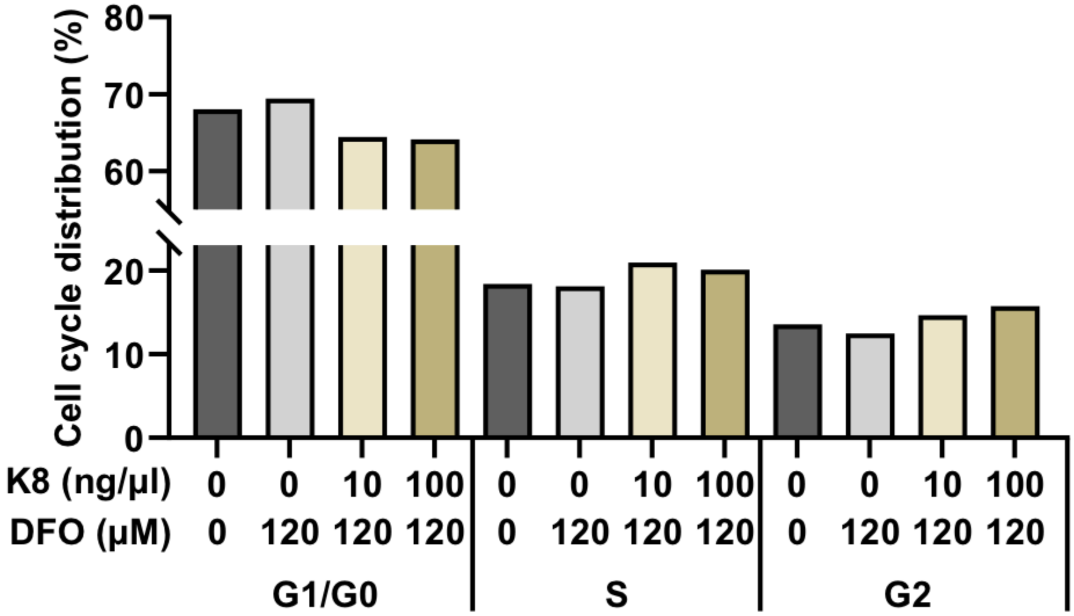

Supplementary Figure 3. hK8-L effect on the cell cycle progression under DFO-induced hypoxic stress.

SH-SY5Y cells were supplemented with hK8-L (K8) 12 h prior to deferoxamine mesylate salt (DFO) treatment to the final concentrations 0, 60, or 120 μM for 24 h. The cell cycle was analyzed using the fluorescence-activated cell sorting (FACS) and the representative result is shown. DFO treatment without K8 slightly increased G0/G1, while DFO treatment after 10 or 100 ng/μL K8 supplementation decreased G0/G1, increasing the cells in S and G2 phases.

SUPPLEMENTARY TABLES

Supplementary Table 1.

The sequences of oligos and primers used in this study

**For ChIP-qPCR Assay**	
**p21 TSS Forward**	GGATGGTAGGAGACAGGAGACC
**p21 TSS Reverse**	GCACACACTCACACAAGCACACATGC
**p21 GB Forward**	GGGAATGTTCATTATGCCAGGC
**p21 GB Reverse**	GGCCAACAGAGTTAAGACACC
**GLUT1 TSS Forward**	GCACGCCAGGGAGCAGGAGACC
**GLUT1 TSS Reverse**	GCGCTGCGCTGGTGGCTCTGGC
**GLUT1 GB Forward**	GGACAAGCCAACTTGTAAATACACC
**GLUT1 GB Reverse**	GCAAGAGGACACTGATGAGAGGTACG
**ALDOC TSS Forward**	GGATCAGAACCCGAGCTGTGCTTGTGG
**ALDOC TSS Reverse**	GCACATCCCGGAGTCCCAGGTAGC
**ALDOC GB Forward**	CCTCCACACTAGGAGTGACCTTCC
**ALDOC GB Reverse**	GCATTGTCCCGTTGCCCTCGC
**IL1β TSS Forward**	CGAGGCACAAGGCACAACAGGCTGC
**IL1β TSS Reverse**	GCATACACACAAAGAGGCAGAGAG
**IL1β GB Forward**	CCTGCTCGTCAGTTTCCTTTCTGG
**IL1β GB Reverse**	CGCTTTTCCATCTTCTTCTTTGGG
**For Real-time PCR Analysis**	
**p21-Forward**	CAT GTG GAC CTG TCA CTG TCT TGT A
**p21-Reverse**	GAA GAT CAG CCG GCG TTT G
**GLUT1-Forward**	GAT TGG CTC CTT CTC TGT GG
**GLUT1-Reverse**	TCA AAG GAC TTG CCC AGT TT
**ALDOC-Forward**	GCG CTG TGT GCT GAA AAT CAG
**ALDOC-Reverse**	CCA CAA TAG GCA CAA TGC CAT T
**IL1β-Forward**	ACA GAT GAA GTG CTC CTT CCA
**IL1β-Reverse**	GTC GGA GAT TCG TAG CTG GAT
**PHD2-Forward**	GCA CGA CAC CGG GAA GTT
**PHD2-Reverse**	CCA GCT TCC CGT TAC AGT
**VHL-Forward**	CTT TGG CTC TTC AGA GAT G
**VHL-Reverse**	CCA GCT TCC CGT TAC AGT
**ACTIN-Forward**	GCC GAC AGG ATG CAG AAG GAG ATC A
**ACTIN-Reverse**	AAG CAT TTG CGG TGG ACG ATG GA

Supplementary Table 2.

Gas chromatography-mass spectrometry data for K8-DMSO

Compound Name	% PEAK	Componen	Match Fact	Componen	Formula	CAS#
Methoxypropionaldehyde	82.91951	2.51E+08	91.6906	45.80081	C4H8O2	990000-34
Methanethiol	10.81693	32734053	75.46146	45.78776	CH4S	74-93-1
2,5-trans-Bis(4-methanethiosulfonylmethylphenyl)-2,	5.04152	15256583	75.89075	45.69745	C22H28NO5S4	990614-54
Dimethyl Sulfoxide	0.451997	1367829	98.68802	59.72653	C2H6OS	67-68-5
(2R)-Amino-3-chloropropionic acid hydrochloride	0.203071	614531.5	87.53784	59.72792	C3H7ClNO2	0-00-0
Dimethylamine-D1	0.151912	459715.5	93.49754	8.678824	C2H6DN	917-72-6
Silanediol, dimethyl-	0.095195	288076.8	93.18628	49.5538	C2H8O2Si	1066-42-8
1,1-Dimethoxy-3-(4-nitrophenyl)propan-2-one	0.063562	192349.9	86.02474	12.24442	C11H13NO5	990152-35
Dimethyl sulfone	0.047064	142425.9	88.25249	62.71782	C2H6O2S	67-71-0
Estra-1,3,5(10)-trien-17-one, 3,4-bis[(trimethylsilyl)ox	0.029203	88373.55	86.69211	30.62446	C24H38O3Si2	51497-39-3
1,2-Di-tert-butylbenzene	0.0279	84431.8	89.02933	36.14315	C14H22	1012-76-6
Pentanoic acid, 5-hydroxy-, 2,4-di-t-butylphenyl ester	0.027365	82810.37	78.30189	82.54106	C19H30O3	166273-38
6-Fluorobenzo[4',5']thieno[2',3':4,5]pyrrolo[1,2-f]phe	0.022431	67881.66	81.17377	30.63244	C22H12FNS	990404-64
Cyclopropane-1,2,3-D3-methanol, (1.alpha.,2.beta.,3	0.018137	54884.87	85.31981	14.03995	C4H5D3O	126036-54
2-(3-Methylanilino)-4-pyridinecarbonitrile	0.017679	53499.79	73.74283	6.567982	C13H11N3	990089-09
Benzeneacetic acid, .alpha.,2-dihydroxy-.alpha.,4-dim	0.011377	34427.95	73.52174	55.97425	C12H16O4	113322-78
N-[2-[3,5-bis(trimethylsilyloxy)phenyl]-2-trimethylsily	0.010872	32901.39	72.36938	51.13627	C23H42F3NO4Si3	325836-92
3,3-Dideutero-(1R,7aR)-hexahydro-1H-pyrrolizin-1-am	0.009969	30166.79	74.18435	80.11869	C7H12D2N2	990004-39
5-Acetyl-Longipinandiolone	0.009026	27314.14	86.41396	80.22815	C17H26O4	990289-11
(S*,S*)-2-Hydroxy(4-methoxy-2-trimethylsilylphenyl)r	0.007131	21578.87	91.62585	41.87247	C18H28O3Si	990354-28
1,3-Dioxolane-2-butanol, .delta.,2-dimethyl-.alpha.-(5	0.005712	17286.89	83.46326	1.103927	C33H60O3	69608-85-1
N(1),N(2)-Dibenzoyl-N(1),N(2)-dimethoxy-hydrazine	0.004699	14221.26	93.30151	47.7331	C16H16N2O4	990304-14
2-Methoxy-6-methyl-9,10-dihydro-9,10-ethanoanthra	0.004205	12724.94	77.55618	102.2049	C20H18O5	990397-11
Methyl 4-(methoxycarbonylmethyl)-2-methyl-5-hydro	0.003528	10675.91	71.38295	10.70994	C13H18N2O5	990257-38

Supplementary Table 3.

Gas chromatography-mass spectrometry data for K8-PBS

Compound Name	Componen	%peak	CAS#	Formula	Match Fact	Componen
2-Oxo-4-phenylbut-3-enyl oct-2-ynoate	5791896	60.86724	990264-51	C18H20O3	85.57901	100.2931
Phenol, 2,4-bis(1,1-dimethylethyl)-	1317730	13.84807	96-76-4	C14H22O	91.8026	82.54074
2(3H)-Furanone, 5-heptyldihydro-	578167.2	6.075979	104-67-6	C11H20O2	89.74623	80.10953
2-(3-Pyridyl)-3-(4-toluenesulfonamido)propylazet	368892.1	3.8767	62247-30-	C15H16N2	92.60122	94.53328
6,7-Dimethoxy-1-[N-(1-phenylethyl)amido]-1,2,3,	217578.8	2.286543	990402-02	C20H24N2	74.52188	82.53788
(anti/syn)-2-Nitro-1-phenylpropan-1,3-diol	130696.1	1.37349	990067-30	C9H11NO4	98.67944	41.69536
Cyclooctasiloxane, hexadecamethyl-	102252.2	1.074572	556-68-3	C16H48O8	72.70593	51.13348
(2R,3S)-2-hydroxy-1-phenyl-3-vinyl-hexan-1-one	92246.56	0.969422	990106-41	C14H18O2	91.33085	47.72387
1,2-Di-tert-butylbenzene	84196.92	0.884828	1012-76-6	C14H22	85.26376	36.14123
Acetic acid ethenyl ester	81025.99	0.851505	108-05-4	C4H6O2	94.69646	9.97751
2-(1',3'-Dioxolan-2'-yl)-3-phenyldecan-4-one	72650.64	0.763488	0-00-0	C18H26O3	82.33333	1.158613
1-Ethyl-2-(4'-fluorophenyl)-4,5-diphenyl-imidazol	60270.97	0.63339	990407-68	C23H19FN	72.23139	30.62656
Methyl 5-[2-(4-Bromophenyl)-2-chloroethyl]-4,6-	60097.73	0.631569	990507-75	C18H18Br	70.24806	82.53236
1-Hexanol, 2-ethyl-	58323.86	0.612928	104-76-7	C8H18O	82.92906	40.16198
trans-3,4-Dimethyl-2,3-epoxypentanal	54514.56	0.572895	990005-04	C7H12O2	82.52884	74.87206
Dimethylamine-D1	53899.81	0.566435	917-72-6	C2H6DN	93.54984	8.673019
1,2,4-Trimethoxy-5-[(1Z)-1-propenyl]benzene	50156.45	0.527096	5273-86-9	C12H16O3	78.65311	88.61464
3-Acetyloxypropyl 2,3,4,6-tetra-O-methyl-.alpha.,	41420.7	0.435292	990391-50	C15H27DO	70.25674	80.21033
2,2-bis(fluoranyl)-1-phenyl-but-3-en-1-ol	38510.76	0.404711	85864-61-	C10H10F2	89.34564	61.79433
2,2-Dimethylpropanoic acid tert-butyl ester	31099.04	0.326821	16474-43-	C9H18O2	84.44331	46.74727
1-(Benzyloxy)-2-fluoro-2-phenyl-3-(p-toluenesulsu	26067.27	0.273942	990534-16	C23H23FO	83.7986	92.01704
4-(p-Methylaminoanilino)pyridine-2-carbonitrile	22935.98	0.241035	990119-48	C13H12N4	86.89953	6.557952
1-Phenyl-3-pentyn-1-ol	22147.14	0.232745	990021-78	C11H12O	87.11352	94.5268
1,1-Dimethoxy-3-(4-nitrophenyl)propan-2-one	20304.48	0.213381	990152-35	C11H13NO	82.5241	12.13586
3-Methyl-1,1-diphenyl-urea	20182.33	0.212097	990124-21	C14H14N2	89.04807	93.79404
Methyl 5-endo-4-hydroxy-2,5,7,7-tetramethylbicy	18681.55	0.196325	990150-69	C14H22O3	86.1174	44.95668
5-Methylene-9-decen-2-one	17948.03	0.188616	990027-19	C11H18O	78.08115	30.2842
6-(2-Aminophenyl)-1-methylpyrido[2,3-d]pyrimid	17778.54	0.186835	990222-52	C14H12N4	72.22201	42.58288
(2S,3S)-3-Methyl-2-(1-phenylethyl)-3-(trifluorome	15004.14	0.157679	990133-36	C11H12F3	91.85487	49.28868
2-Hexoxybenzoic acid methyl ester	14204.5	0.149276	56306-81-	C14H20O3	71.67806	56.36205
Dimethyl ether	13474.4	0.141603	115-10-6	C2H6O	87.91991	54.66559
(1R,4R,5R)-4-fluoranyl-1-methyl-5-[(4-methylpher	10775.96	0.113245	990257-95	C15H19FO	78.84514	10.72008
(S)-[1-(1-Allyloxyethyl)-1-vinylalloxymethyl]benzer	10490.24	0.110242	990198-79	C17H22O2	71.25738	62.29434

Supplementary Table 4.

Gas chromatography-mass spectrometry data for K8-powder

Component F	Compound Name	Match Facto	Best Hit	Formula	Component A	CAS#
6.55551989	2,3-bis[(E)-3-(3,4-dimethoxyphenyl)-1-oxoprop-2-enoxy]butanedioic acid	73.8399058	TRUE	C26H26O12	14959.2957	990620-32-9
8.6721785	Dimethyl ether	94.9951227	TRUE	C2H6O	473357.228	115-10-6
10.7240144	3,5-Bis[(E)-2-phenylethenyl]pyridine	79.1999309	TRUE	C21H17N	61015.1043	990261-79-8
17.1987526	2-Propanol, 1-methoxy-	95.4162693	TRUE	C4H10O2	154279.701	107-98-2
21.6585042	Tridecane, 7-hexyl-	99.5987774	TRUE	C19H40	44178.3971	7225-66-3
24.4837387	2,6,10 - trimethyl - tridecane (without stereochemistry)	85.7213473	TRUE	C16H34	100373.331	3891-99-4
24.4866413	N-Methoxy-N-methyl-2-ethylhexanoylamide	79.1105479	TRUE	C10H21NO2	38017.2542	990051-95-9
26.4624373	Cyclohexane, isocyanato-	88.8130362	TRUE	C7H11NO	188265.487	3173-53-3
27.9117434	Heptadecane, 2,6,10,15-tetramethyl-	98.8905721	TRUE	C21H44	35348.7848	54833-48-6
29.6333346	1,3-Dioxolane	83.0480914	TRUE	C3H6O2	31148.6863	646-06-0
32.8902753	Hexadecane, 1-iodo-	90.5859287	TRUE	C16H33I	13991.9475	544-77-4
33.9234453	Anhydro - sugar	75.1400598	TRUE	C5H8O4	12534.3832	0-00-0
34.4767878	Heptadecane, 2,6,10,15-tetramethyl-	91.0971964	TRUE	C21H44	13057.1115	54833-48-6
36.142223	1,2-Di-tert-butylbenzene	90.1090631	TRUE	C14H22	84506.8858	1012-76-6
36.2431067	2,6-Nonanedione	80.5000142	TRUE	C9H16O2	16310.808	36452-81-0
37.2536849	Decane, 3-ethyl-3-methyl-	87.3468504	TRUE	C13H28	116360.598	17312-66-2
37.8254518	Hexadecane, 1-iodo-	90.9736885	TRUE	C16H33I	32346.6393	544-77-4
38.0085634	Hexadecane, 1-iodo-	91.4338438	TRUE	C16H33I	22538.6873	544-77-4
40.1598161	4,4-Dimethylpentan-1-ol	85.4615539	TRUE	C7H16O	49004.8287	990002-63-9
40.4579876	2,2-Dimethylpropanoic acid 3-acetyloxypropyl ester	93.9552436	TRUE	C10H18O4	33537.0062	990075-06-2
41.0597199	2,2-Dimethylpropanoic acid 3-acetyloxypropyl ester	92.2859016	TRUE	C10H18O4	11810.5711	990075-06-2
41.3152173	4H-1-Benzopyran-4-one, 5,6,7-trimethoxy-2-(4-methoxyphenyl)-	74.2396164	TRUE	C19H18O6	30496.185	1168-42-9
41.6899887	.beta.,D-Xylopyranose Tetrabenzoate	93.9375056	TRUE	C33H26O9	32584.3733	990630-11-9
47.92881	2,2-Dimethylpropanoic acid 3-acetyloxypropyl ester	89.6256119	TRUE	C10H18O4	10104.8958	990075-06-2
48.9912017	Hexadecane, 1-iodo-	91.2583706	TRUE	C16H33I	19014.2452	544-77-4
50.2964237	1,1-Cyclopropanedicarbonitrile, 2-methyl-2-pentyl-	79.2801454	TRUE	C11H16N2	10479.0304	16738-90-2
50.5562431	3,5-bis[14C]-Trachelantamidine	84.1754905	TRUE	C8H15NO	49659.5552	990009-77-6
51.1328211	3-Methoxy-2'-phenyl-16.beta.,17.beta.-dihydro-4'H-[1,3]oxazino[5',6' : 16,17]estra-1,3	71.0410185	TRUE	C27H31NO2	31498.157	990516-57-1
51.1390334	2,3-Diphenyl-5,8,9,10-tetrahydropyrimido[1,2-c]pteridin-6-one	82.2482252	TRUE	C21H17N5O	34089.6143	990434-90-9
56.3454673	5-Diazo-1,3-cyclopentadiene	72.8312091	TRUE	C5H4N2	35619.1322	1192-27-4
56.355289	Methyl salicylate	92.5419974	TRUE	C8H8O3	118664.987	119-36-8
56.4253057	2-Acetamidopentyl acetate	88.7578257	TRUE	C9H17NO3	14216.764	990051-78-0
59.0746719	4-Methylphthalide	84.9063943	TRUE	C9H8O2	39555.194	990013-04-1
61.7813267	(Z,Z)-(+-)-cis-2-(2,5-Octdienyl)-3-undecyloxirane	91.2671986	TRUE	C21H38O	195490.857	990320-72-3
61.7888043	(R)-S-Ethylthio mandelate	86.1929925	TRUE	C10H12O2S	134906.852	990065-39-2
66.7159322	Bis-(3,5,5-trimethylhexyl) ether	85.9591101	TRUE	C18H38O	44401.6611	990229-48-0
71.6198022	(phenylmethyl) undec-10-ynoate	90.3906133	TRUE	C18H24O2	63155.9863	990234-29-8
71.6832479	2-Methoxyindan-1-one	78.1586553	TRUE	C10H10O2	12046.6642	990023-11-2
74.8737365	2(3H)-Furanone, 5-hexyldihydro-	93.1540036	TRUE	C10H18O2	529409.773	706-14-9
74.8826955	Methyl (2RS,3RS)-3-Acetoxy-2-[(SR)-1-hydroxyethyl]butanoate	74.8754002	TRUE	C9H16O5	35865.1011	990078-31-9
74.9158627	2H-Cyclopropa[b]naphthalen-2-one, 1,1a,2a,3,6,6a,7,7a-octahydro-1,1,2a,4,5-pentame	88.7276822	TRUE	C16H24O	33018.8979	127279-91-8
76.8483439	2-Propenoic acid 3-[(1E,3Z)-penta-1,3-dienoxy]propyl ester	86.054029	TRUE	C11H16O3	14829.6759	990066-09-6
78.9879232	Benzyl lactate	88.3763808	TRUE	C10H12O3	189652.886	990042-49-0
78.9924983	Heptane, 1,1,1,2,3,3-hexafluoro-	86.8541151	TRUE	C7H10F6	18881.5402	57915-71-6
80.1048133	2-Methyl-1,3-oxazole-4-carboxylic acid ethyl ester	74.8847019	TRUE	C7H9NO3	39466.2928	990017-80-8
80.123786	2(3H)-Furanone, 5-heptyldihydro-	94.4535478	TRUE	C11H20O2	3243608.51	104-67-6
80.1241278	(E)-1-(2-Tetrahydropyranyloxy)-7-decene	76.0774499	TRUE	C15H28O2	1713054.04	990155-85-8
80.1344537	1-(Bromoethynyl)cyclohexan-1-on	86.6239124	TRUE	C8H11BrO	64736.9598	0-00-0
82.5334733	Pyrimidinium, 5-carboxy-4-(1,1-dimethylethyl)-1,6-dimethyl-2-(1-methylethyl)-, hydro	82.9198777	TRUE	C14H22N2O2	110617.412	108169-08-0
83.7396623	Benzene, 1,2,3-trimethoxy-5-(1-propenyl)-, (E)-	91.5650593	TRUE	C12H16O3	2447566.89	5273-85-8
88.6036072	tert-Butyl (2S*,7aR*)-2-(2-methoxy-2-oxoethyl)-2-[(methylsulfonyl)oxy]-3-oxotetrahyd	84.1056309	TRUE	C16H25NO8	18493.9147	0-00-0
88.6186997	.beta.-Asarone	83.953534	TRUE	C12H16O3	294602.211	5273-86-9
92.6781031	(E)-phenyl 3-cyclohexyl-2-methylacrylate	77.7460694	TRUE	C16H20O2	80979.3364	990165-06-3
93.8068026	3-(3-Methylphenyl)-pyridine	91.5264398	TRUE	C12H11N	151127.955	990030-50-7
94.530909	3-(Benzyloxy)-1-propanal	75.3801048	TRUE	C10H12O2	210048.443	990024-51-7
94.5380978	Benzyl Benzoate	95.5243706	TRUE	C14H12O2	540862.478	120-51-4
96.4922603	Spiro[cyclopentane-1,1'(2'H)-naphthalene]-5'-ethanamine, N-ethyl-7',8'-dimethoxy-N-	79.4157092	TRUE	C21H31NO2	818688.291	63080-55-7
100.571827	(2R)-2-(6-methoxy-2-naphthalenyl)propanenitrile	76.8498708	TRUE	C14H13NO	23117.789	108865-01-6

Raw Data for Representative Immunoblots

Fig. 1C: Uncropped Image

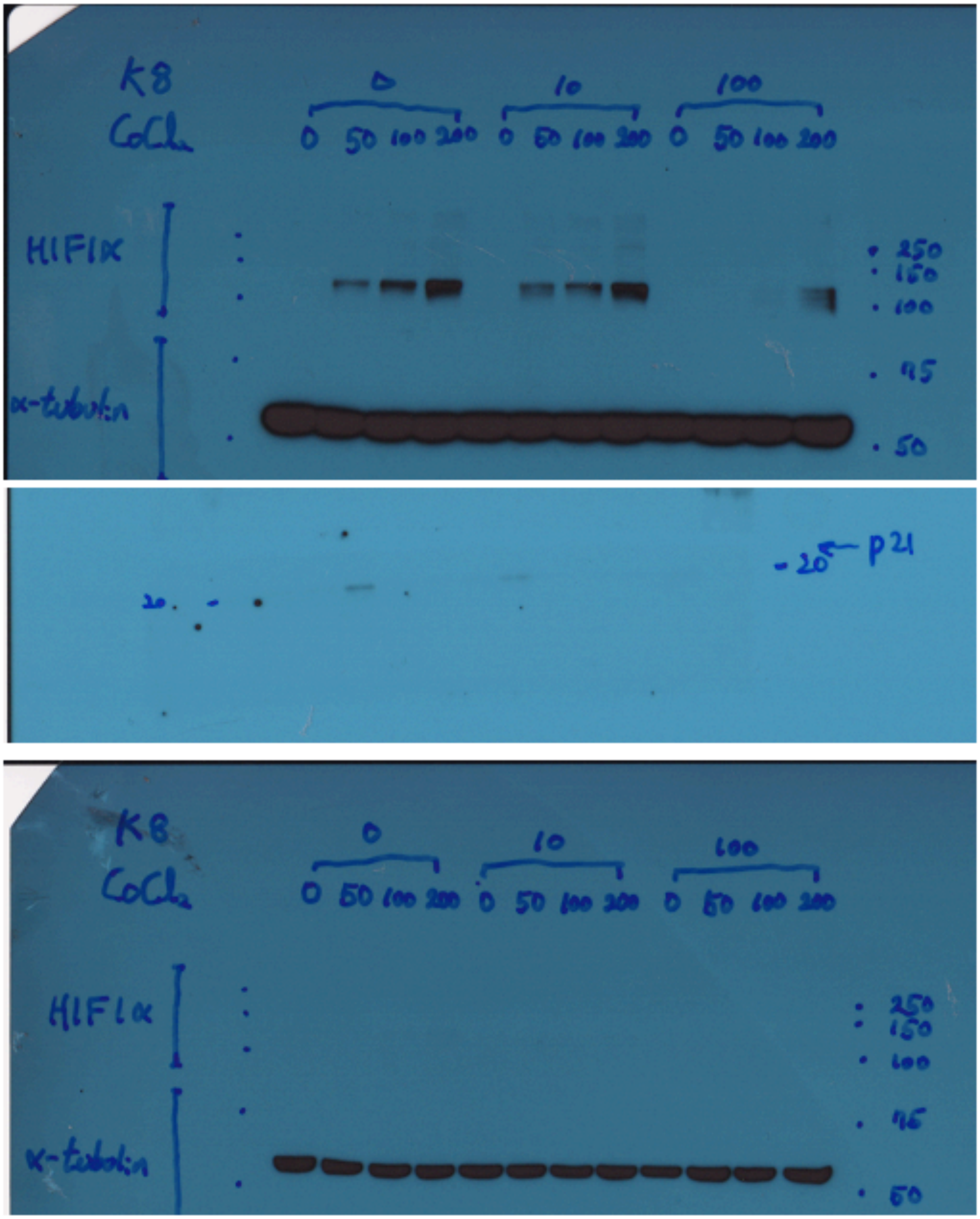

Fig. 2A: Uncropped Image

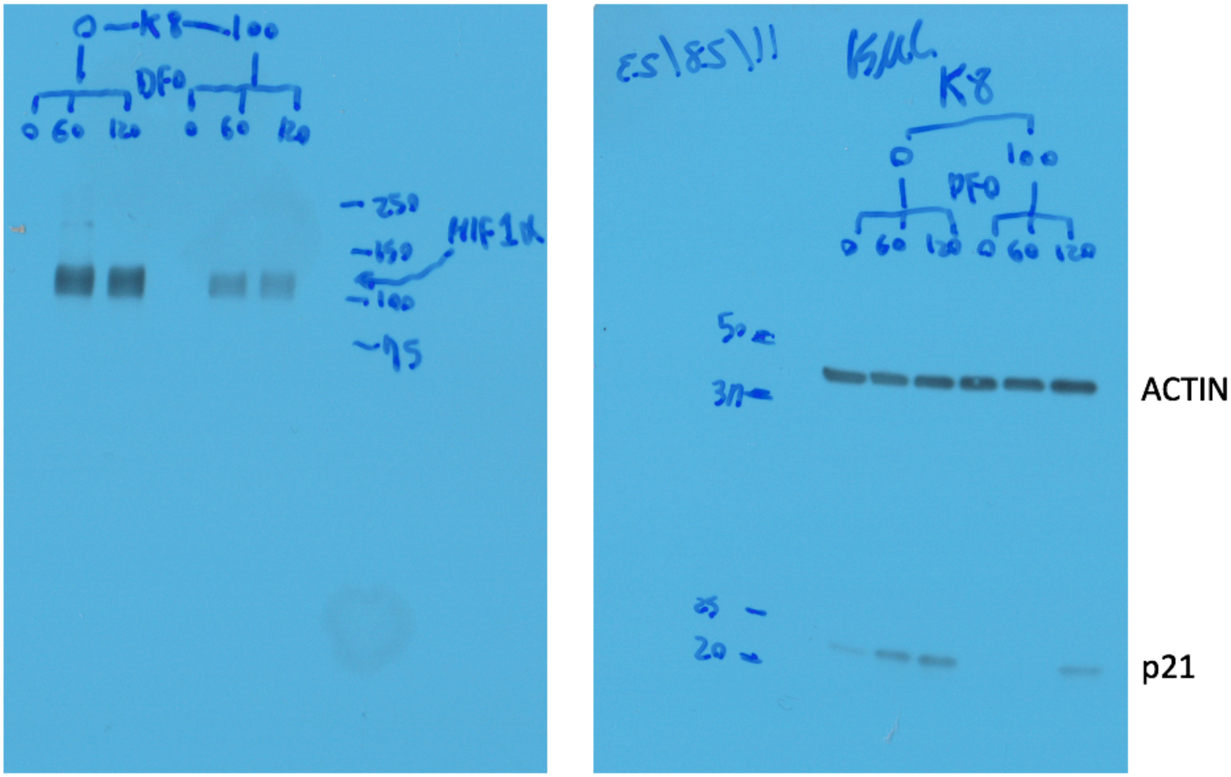

Fig. 5B: Uncropped Image

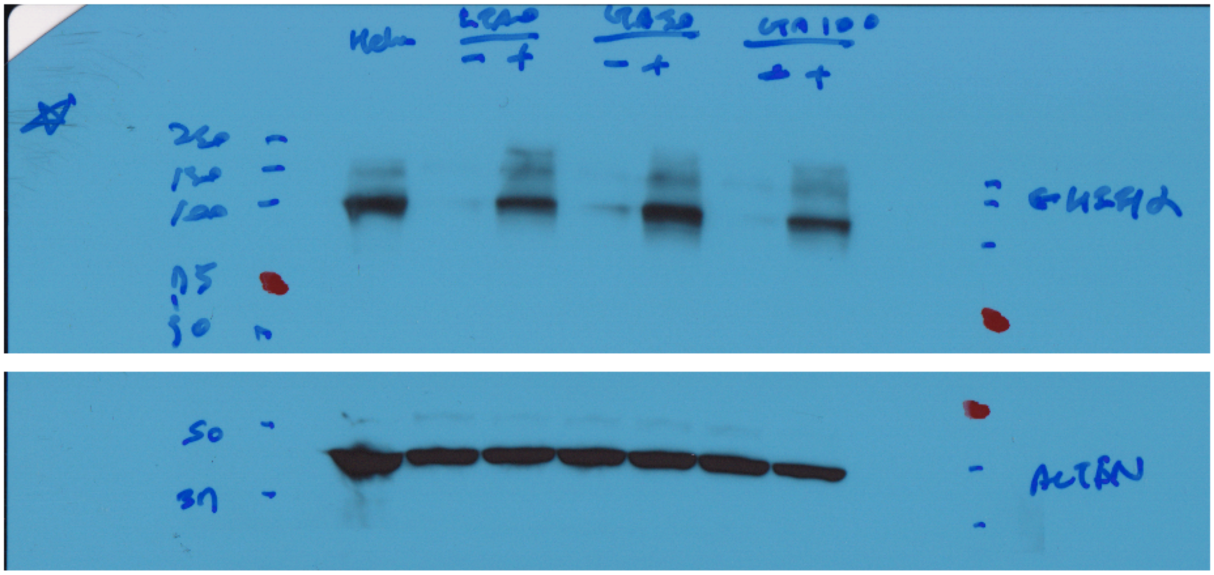

Fig. 6B: Uncropped Image

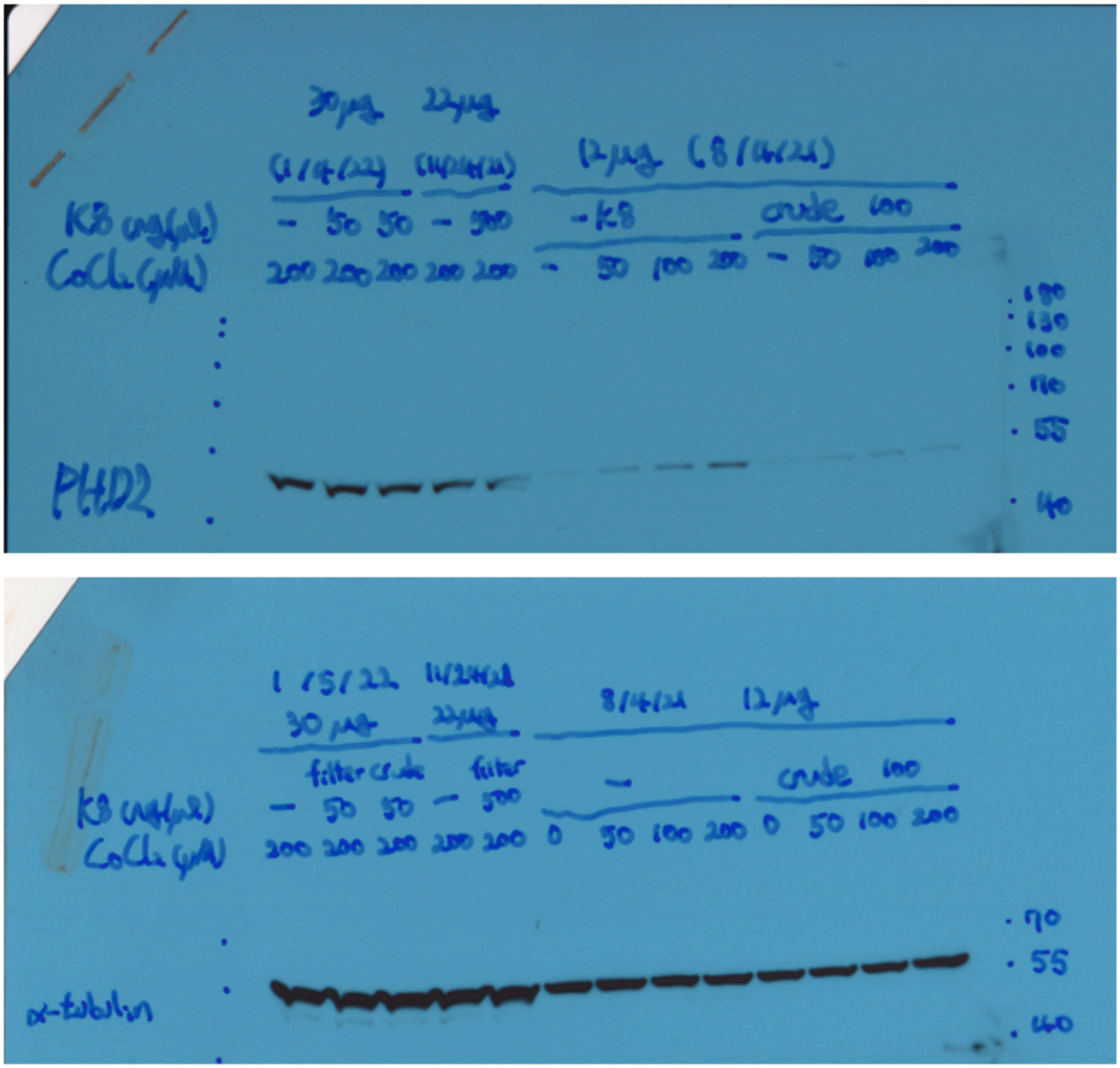

Supplement: Supplementary file 1 — Supplementary Information 1. [file 41598_2024_56958_MOESM1_ESM.pdf]
