# Supplementary material for: Lactiplantibacillus plantarum K8 lysates regulate hypoxia-induced gene expression
Source: Sci Rep. 2024 Mar 15;14:6275. doi: 10.1038/s41598-024-56958-7 (PMC10943017; doi:10.1038/s41598-024-56958-7)
Supplement: Supplementary file 2 — Supplementary Information 2. [file 41598_2024_56958_MOESM2_ESM.pdf]

# Unknown Analysis Report - Best Hits

| | | | |
|---|---|---|---|
| **Batch Path** | D:\MassHunter\GCMS\1\data\2023\2023-10 | | |
| **Analysis File Name** | 11795.uaf | | |
| **Analyst Name** | admin | | |
| **Analysis Time** | 11/14/2023 4:36:51 PM | | |
| **Data File Name** | 11795-4.D | **Data Path Name** | D:\MassHunter\GCMS\1\data\2023\2023-10 |
| **Sample Name** | K8 DMSO | **Sample Type** | Sample |
| **Acq Method File** | DB-WAX | **Acq Method Path** | D:\MassHunter\GCMS\1\methods\ |
| **Acq Time** | 11/13/2023 11:43:31 PM | **Operator** | |
| **Instrument Name** | GCMS | **Dilution** | 1 |

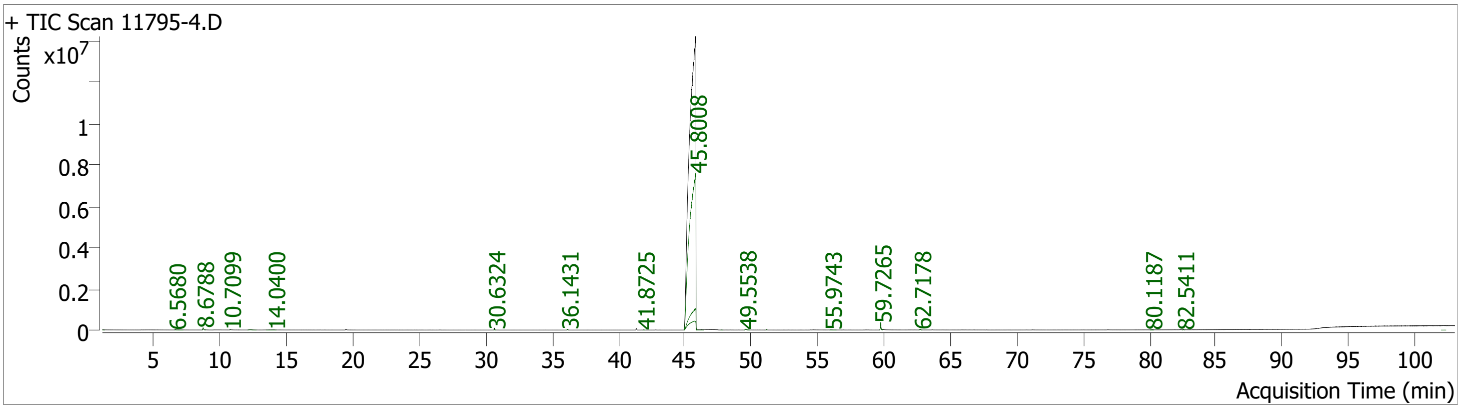


| RT | Compound Name | CAS# | Formula | Area | MI | Match Score | Sample | Sample |
|---|---|---|---|---|---|---|---|---|
| 1.1039 | 1,3-Dioxolane-2-butanol, .delta.,2-dimethyl-.alpha.-(5-methyl-4-tricosen-6-ynyl)- | 69608-85-1 | C33H60O3 | 17287 | | 83.5 | 0.01 | 0.01 |
| 6.5680 | 2-(3-Methylanilino)-4-pyridinecarbonitrile | 990089-09-2 | C13H11N3 | 53500 | | 73.7 | 0.02 | 0.02 |
| 8.6788 | Dimethylamine-D1 | 917-72-6 | C2H6DN | 459716 | | 93.5 | 0.15 | 0.18 |
| 10.7099 | Methyl 4-(methoxycarbonylmethyl)-2-methyl-5-hydroxyiminomethylpyrrole-3-propanoate | 990257-38-9 | C13H18N2O5 | 10676 | | 71.4 | 0.00 | 0.00 |
| 12.2444 | 1,1-Dimethoxy-3-(4-nitrophenyl)propan-2-one | 990152-35-6 | C11H13NO5 | 192350 | | 86.0 | 0.06 | 0.08 |
| 14.0400 | Cyclopropane-1,2,3-D3-methanol, (1.alpha.,2.beta.,3.beta.)- | 126036-54-2 | C4H5D3O | 54885 | | 85.3 | 0.02 | 0.02 |
| 30.6245 | Estra-1,3,5(10)-trien-17-one, 3,4-bis[(trimethylsilyl)oxy]- | 51497-39-3 | C24H38O3Si2 | 88374 | | 86.7 | 0.03 | 0.04 |
| 30.6324 | 6-Fluorobenzo[4',5']thieno[2',3':4,5]pyrrolo[1,2-f]phenanthridine | 990404-64-3 | C22H12FNS | 67882 | | 81.2 | 0.02 | 0.03 |
| 36.1431 | 1,2-Di-tert-butylbenzene | 1012-76-6 | C14H22 | 84432 | | 89.0 | 0.03 | 0.03 |
| 41.8725 | (S*,S*)-2-Hydroxy(4-methoxy-2-trimethylsilylphenyl)methyl-1-cycloheptanone | 990354-28-4 | C18H28O3Si | 21579 | | 91.6 | 0.01 | 0.01 |
| 45.6975 | 2,5-trans-Bis(4-methanethiosulfonylmethylphenyl)-2,5-dimethylpyrrolidin-1-yloxyl radical | 990614-54-3 | C22H28NO5S4 | 15256583 | | 75.9 | 5.04 | 6.08 |
| 45.7878 | Methanethiol | 74-93-1 | CH4S | 32734053 | | 75.5 | 10.82 | 13.05 |
| 45.8008 | Methoxypropionaldehyde | 990000-34-9 | C4H8O2 | 250929967 | | 91.7 | 82.92 | 100.00 |
| 47.7331 | N(1),N(2)-Dibenzoyl-N(1),N(2)-dimethoxy-hydrazine | 990304-14-7 | C16H16N2O4 | 14221 | | 93.3 | 0.00 | 0.01 |
| 49.5538 | Silanediol, dimethyl- | 1066-42-8 | C2H8O2Si | 288077 | | 93.2 | 0.10 | 0.11 |
| 51.1363 | N-[2-[3,5-bis(trimethylsilyloxy)phenyl]-2-trimethylsilyloxy-ethyl]-N-tert-butyl-2,2,2-tris(fluoranyl)ethanamide | 325836-92-8 | C23H42F3NO4 Si3 | 32901 | | 72.4 | 0.01 | 0.01 |
| 55.9743 | Benzeneacetic acid, .alpha.,2-dihydroxy-.alpha.,4-dimethyl-, ethyl ester, (R)- | 113322-78-4 | C12H16O4 | 34428 | | 73.5 | 0.01 | 0.01 |
| 59.7265 | Dimethyl Sulfoxide | 67-68-5 | C2H6OS | 1367829 | | 98.7 | 0.45 | 0.55 |
| 59.7279 | (2R)-Amino-3-chloropropionic acid hydrochloride | 0-00-0 | C3H7ClNO2 | 614532 | | 87.5 | 0.20 | 0.24 |
| 62.7178 | Dimethyl sulfone | 67-71-0 | C2H6O2S | 142426 | | 88.3 | 0.05 | 0.06 |
| 80.1187 | 3,3-Dideutero-(1R,7aR)-hexahydro-1H-pyrrolizin-1-amine | 990004-39-6 | C7H12D2N2 | 30167 | | 74.2 | 0.01 | 0.01 |
| 80.2282 | 5-Acetyl-Longipinandiolone | 990289-11-0 | C17H26O4 | 27314 | | 86.4 | 0.01 | 0.01 |
| 82.5411 | Pentanoic acid, 5-hydroxy-, 2,4-di-t-butylphenyl esters | 166273-38-7 | C19H30O3 | 82810 | | 78.3 | 0.03 | 0.03 |
| 102.2049 | 2-Methoxy-6-methyl-9,10-dihydro-9,10-ethanoanthracene-11,12-dicarboxylic acid | 990397-11-4 | C20H18O5 | 12725 | | 77.6 | 0.00 | 0.01 |

# Unknown Analysis Report - Best Hits

| RT | Compound Name | CAS# | Formula | Area | MI | Match Score | Sample | Sample |
|---|---|---|---|---|---|---|---|---|
| 1.1039 | 1,3-Dioxolane-2-butanol, .delta.,2-dimethyl-.alpha.-(5-methyl-4-tricosen-6-ynyl)- | 69608-85-1 | C33H60O3 | 17287 | | 83.5 | 0.01 | 0.01 |

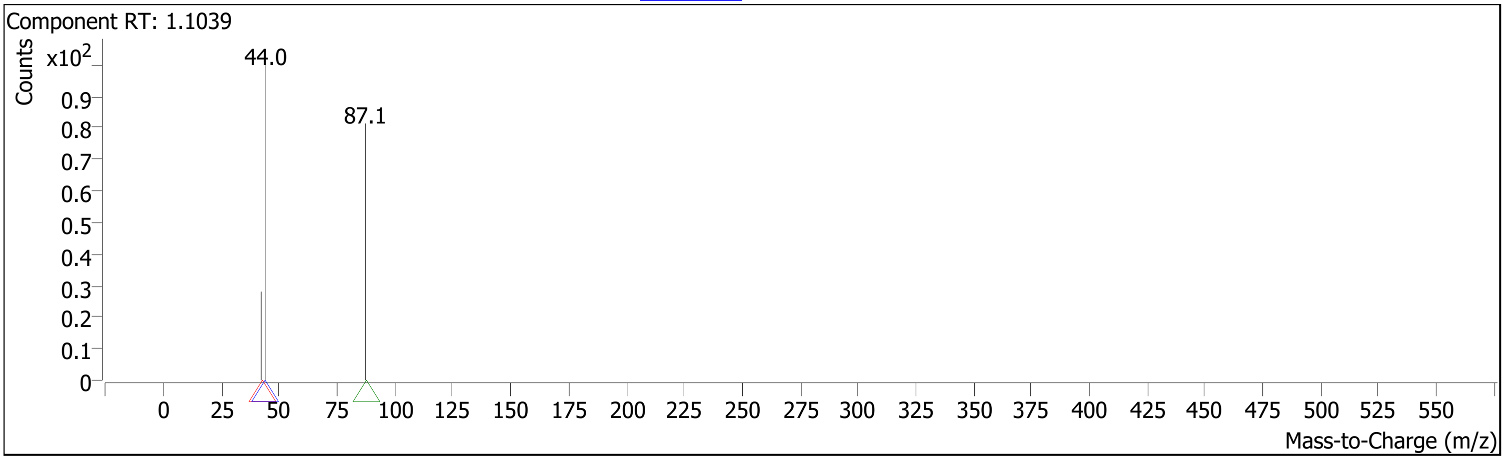


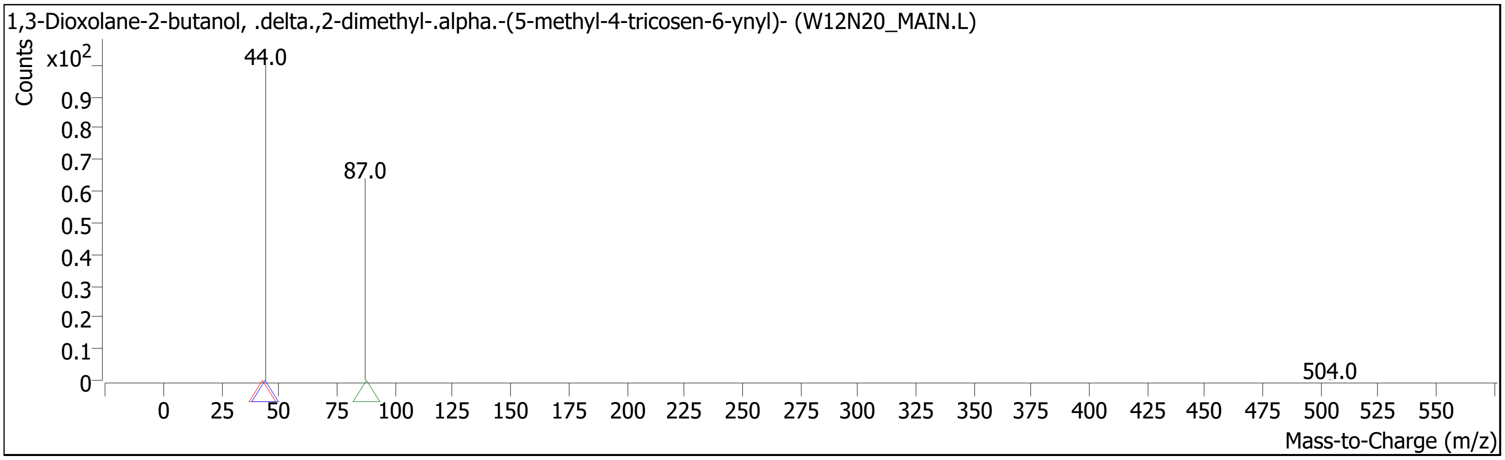


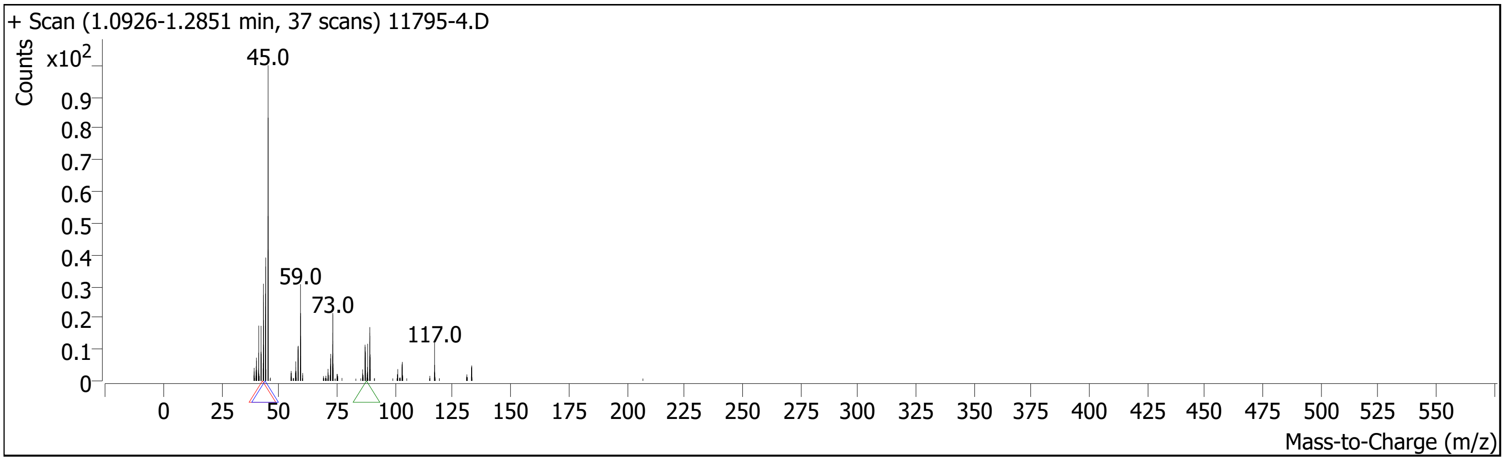


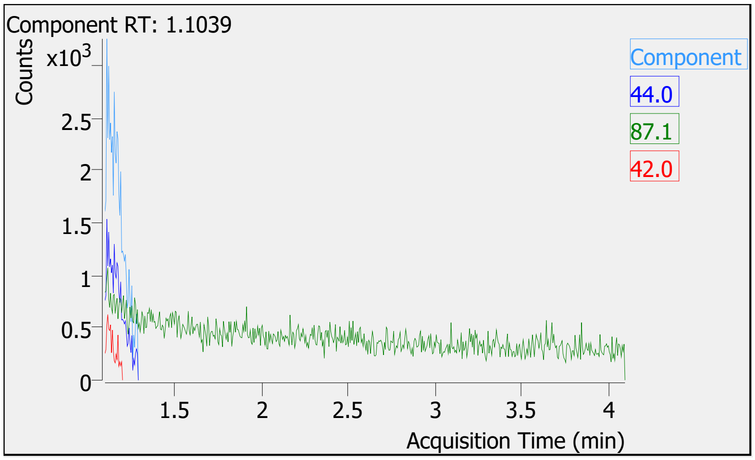


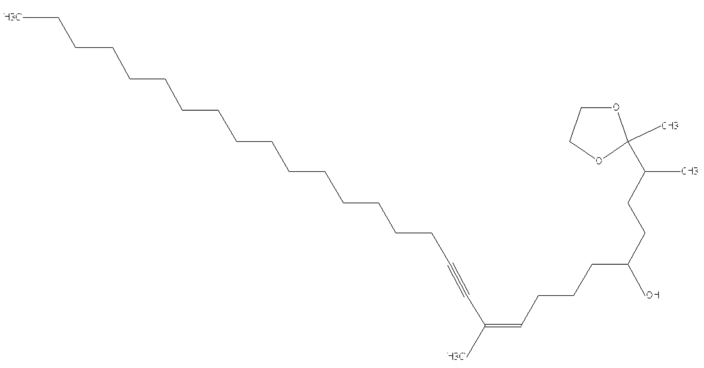

 Generated at 4:37 PM on 11/14/2023

| RT | Compound Name | CAS# | Formula | Area | MI | Match Score | Sample | Sample |
|---|---|---|---|---|---|---|---|---|
| 6.5680 | 2-(3-Methylanilino)-4-pyridinecarbonitrile | 990089-09-2 | C13H11N3 | 53500 | | 73.7 | 0.02 | 0.02 |

Component RT: 6.5680

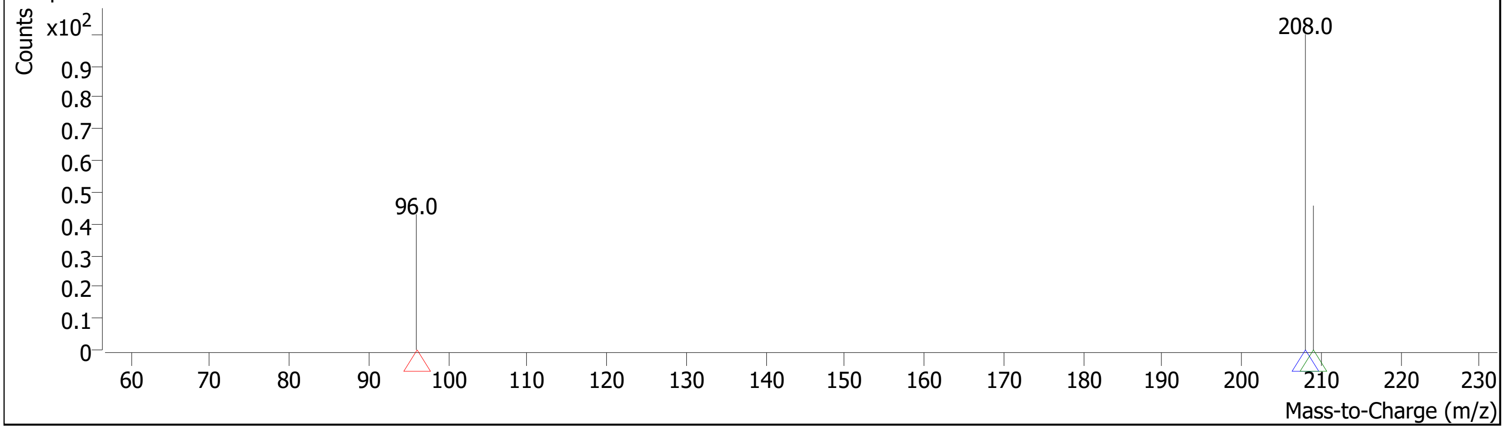


2-(3-Methylanilino)-4-pyridinecarbonitrile (W12N20_MAIN.L)

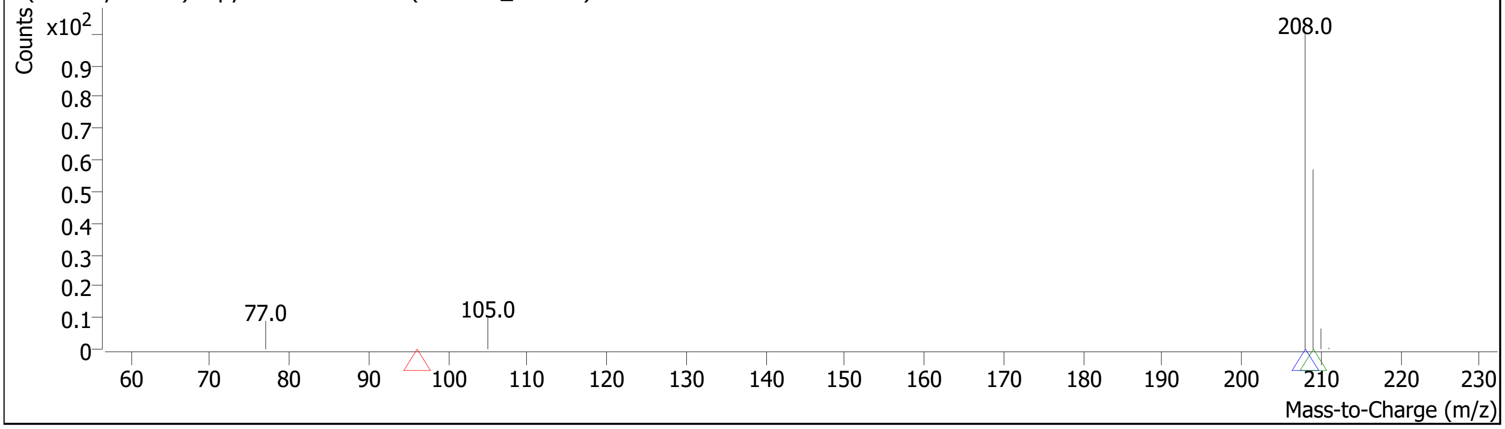


+ Scan (6.5215-7.2008 min, 128 scans) 11795-4.D

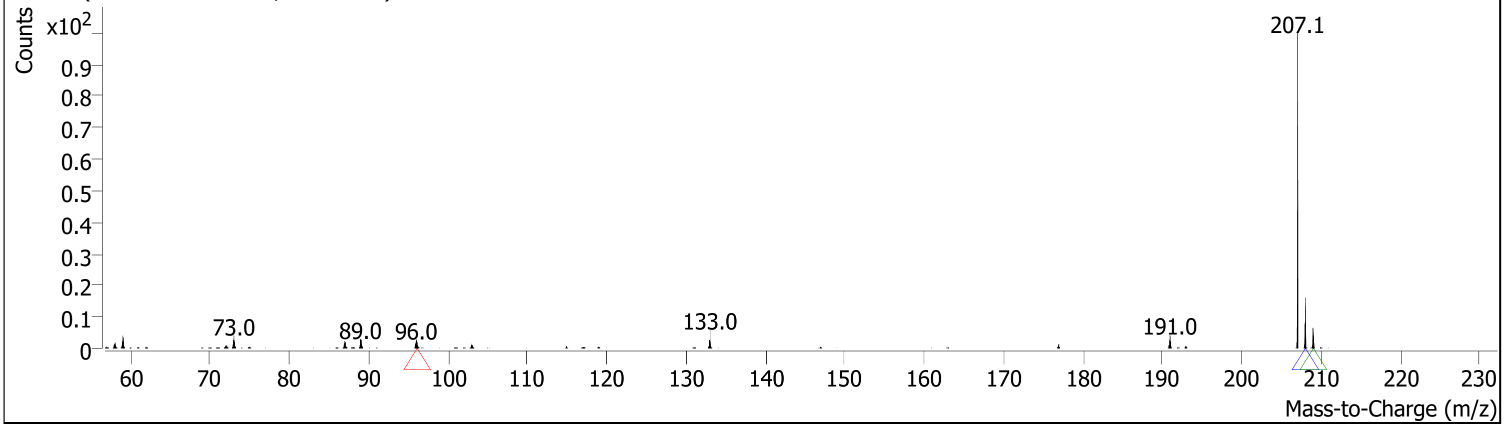


Component RT: 6.5680

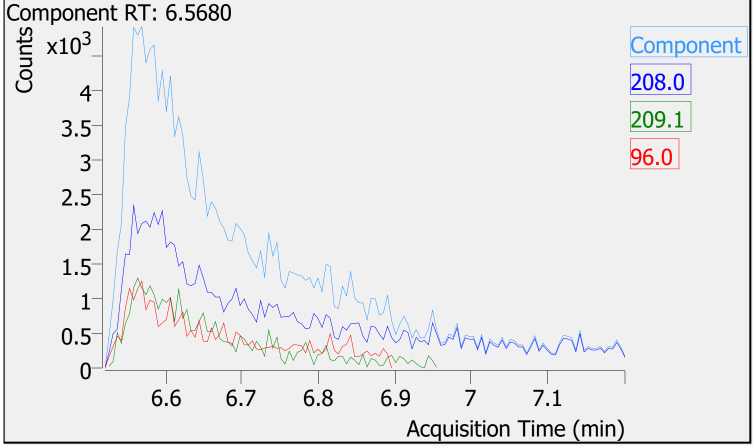


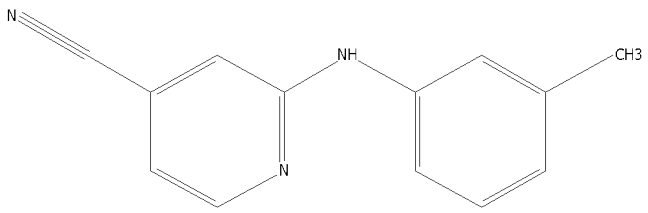

# Unknown Analysis Report - Best Hits

| RT | Compound Name | CAS# | Formula | Area | MI | Match Score | Sample | Sample |
|---|---|---|---|---|---|---|---|---|
| 8.6788 | Dimethylamine-D1 | 917-72-6 | C2H6DN | 459716 | | 93.5 | 0.15 | 0.18 |

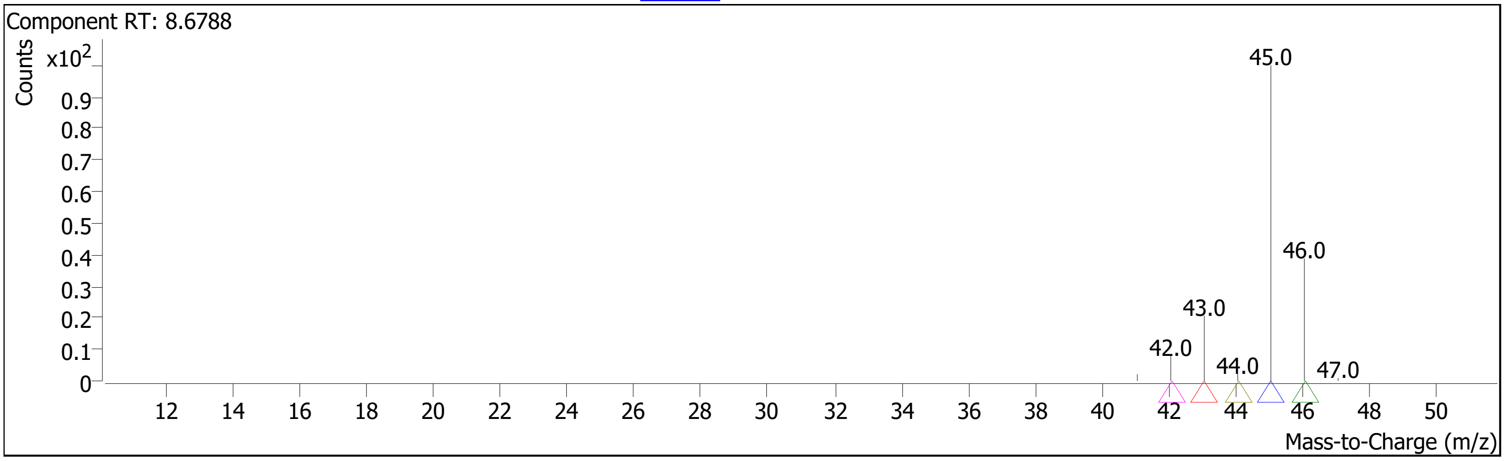


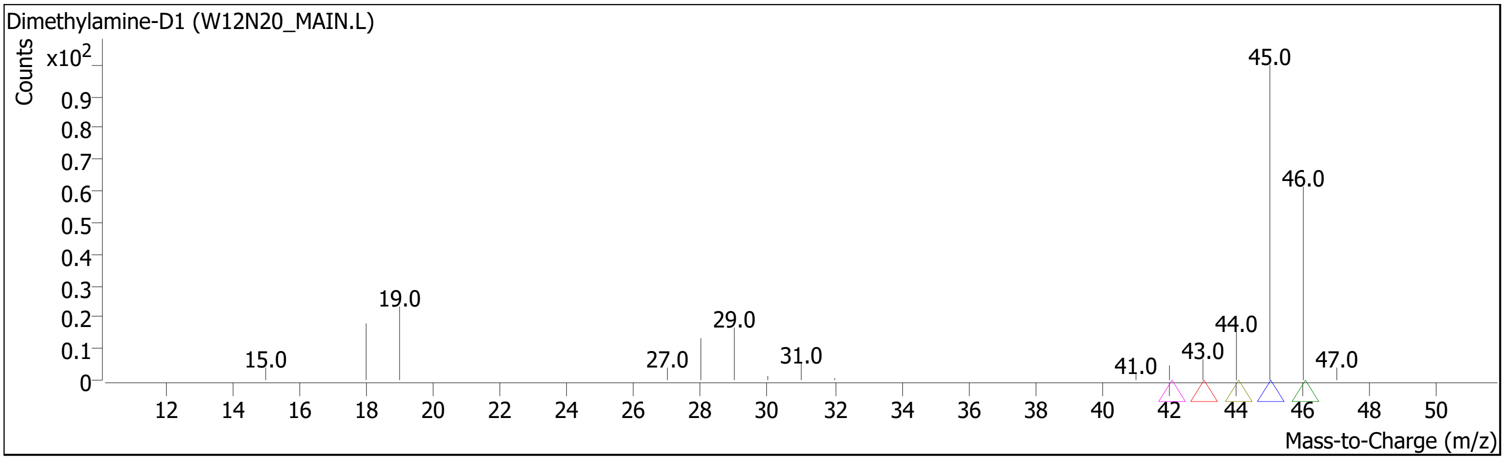


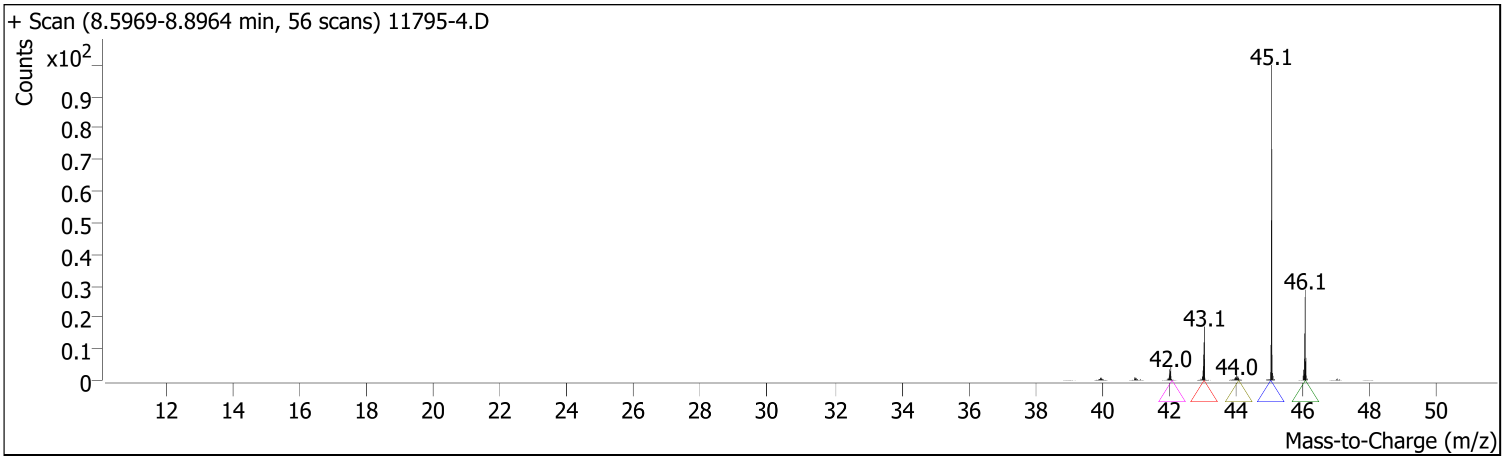


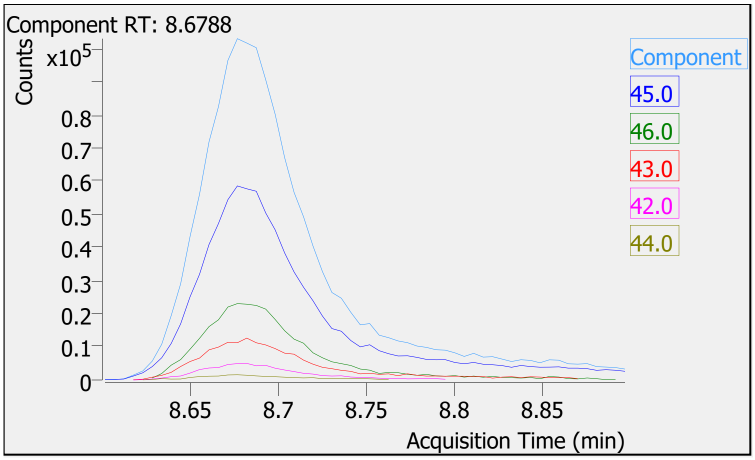


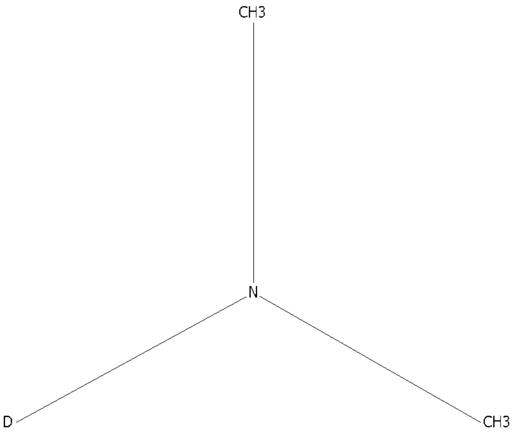


 Generated at 4:37 PM on 11/14/2023

# Unknown Analysis Report - Best Hits

| RT | Compound Name | CAS# | Formula | Area | MI | Match Score | Sample | Sample |
|---|---|---|---|---|---|---|---|---|
| 10.7099 | Methyl 4-(methoxycarbonylmethyl)-2-methyl-5-hydroxyiminomethylpyrrole-3-propanoate | 990257-38-9 | C13H18N2O5 | 10676 | | 71.4 | 0.00 | 0.00 |

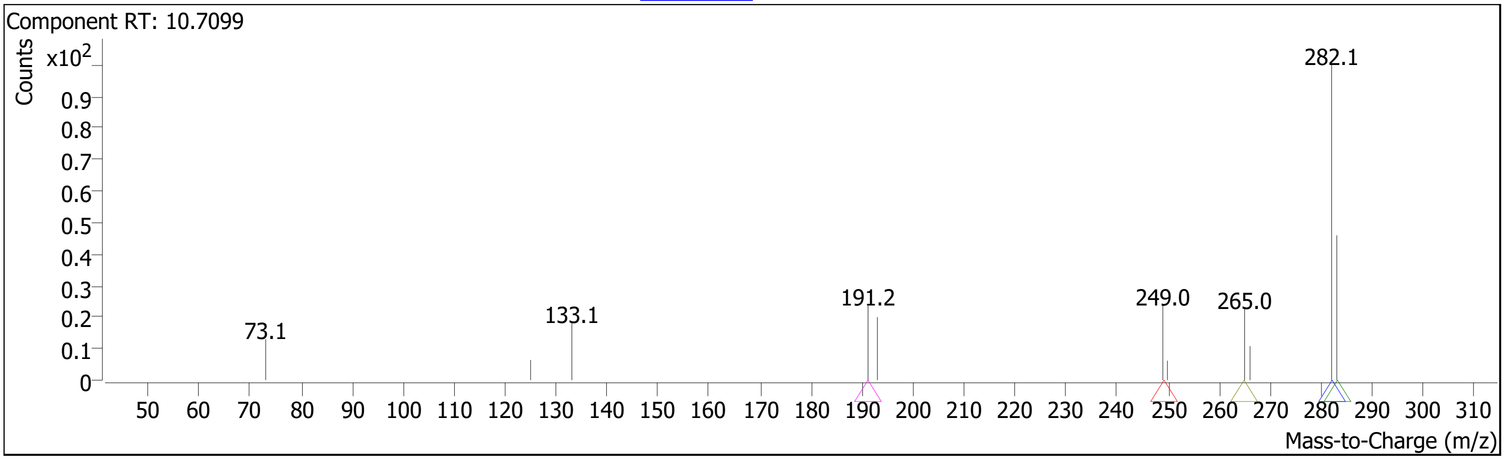


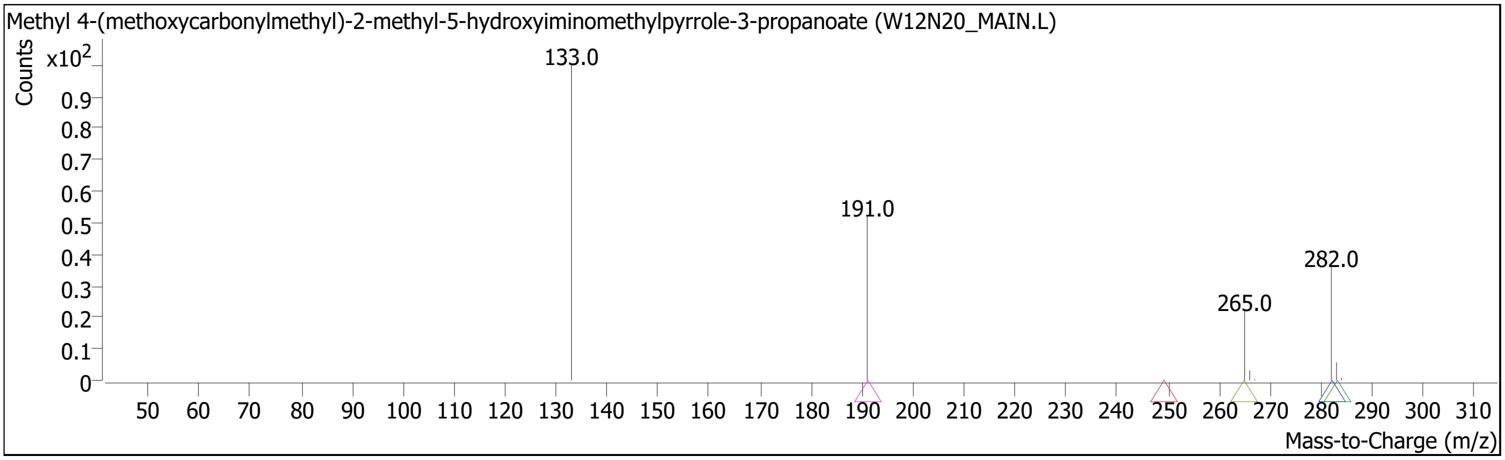


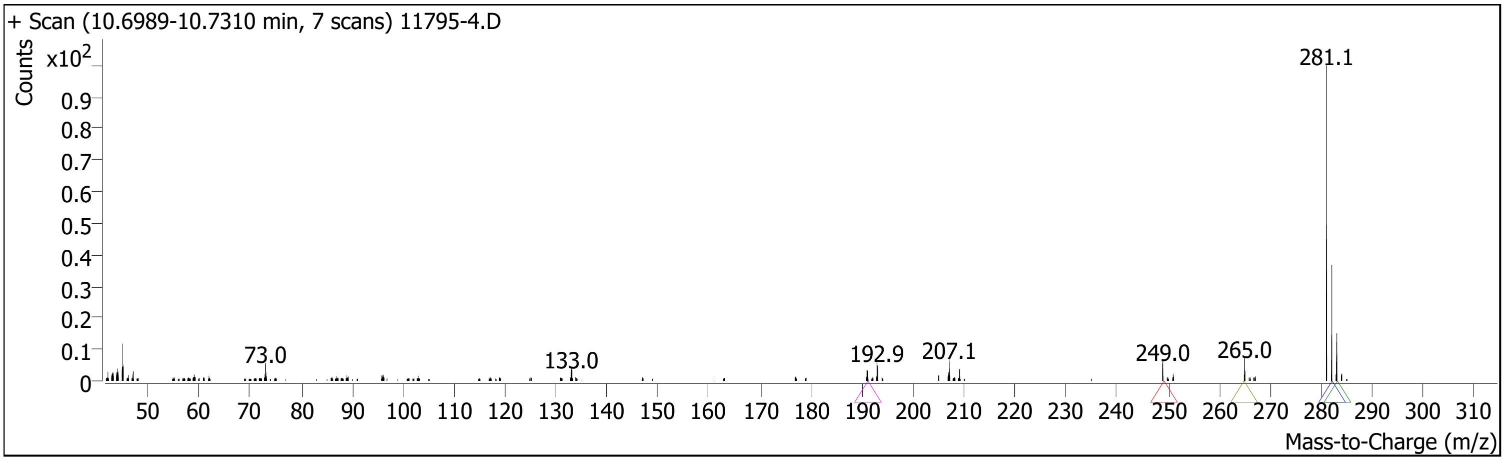


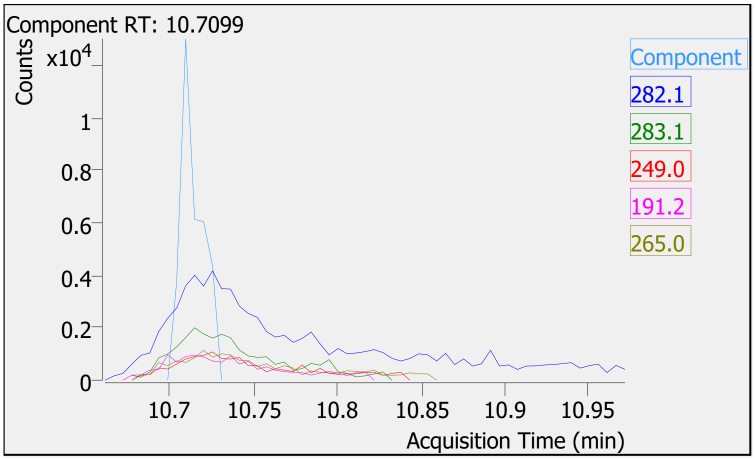


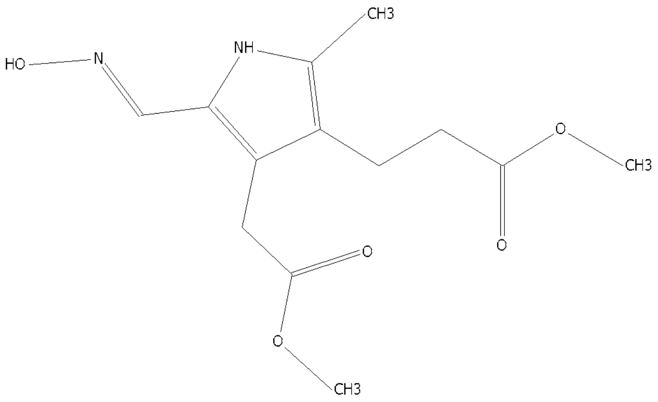

# Unknown Analysis Report - Best Hits

| RT | Compound Name | CAS# | Formula | Area | MI | Match Score | Sample | Sample |
|---|---|---|---|---|---|---|---|---|
| 12.2444 | 1,1-Dimethoxy-3-(4-nitrophenyl)propan-2-one | 990152-35-6 | C11H13NO5 | 192350 | | 86.0 | 0.06 | 0.08 |

Component RT: 12.2444

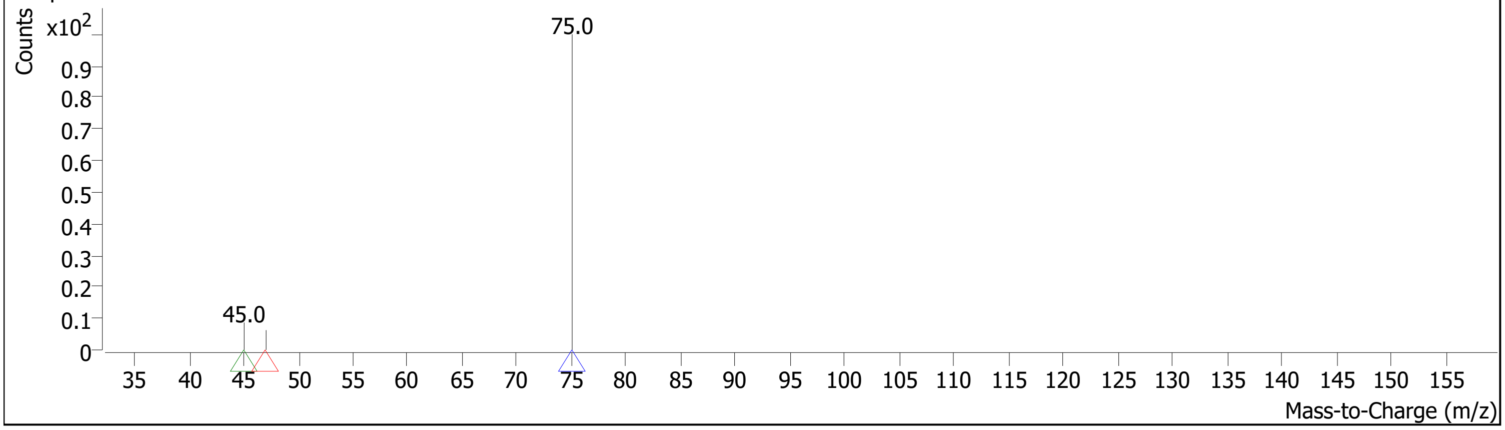


1,1-Dimethoxy-3-(4-nitrophenyl)propan-2-one (W12N20_MAIN.L)

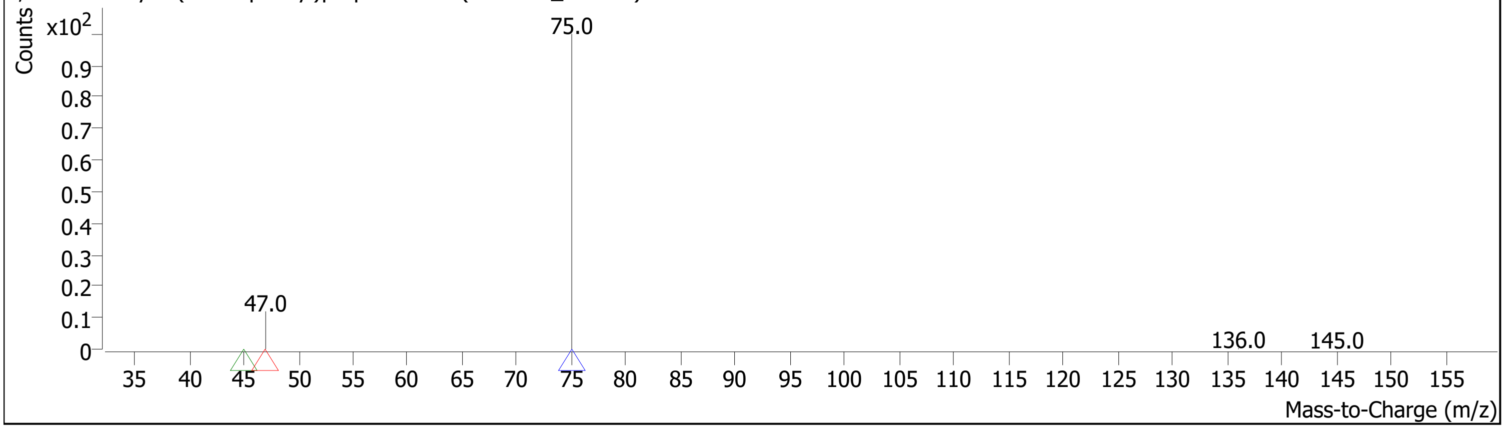


+ Scan (12.0227-12.7100 min, 129 scans) 11795-4.D

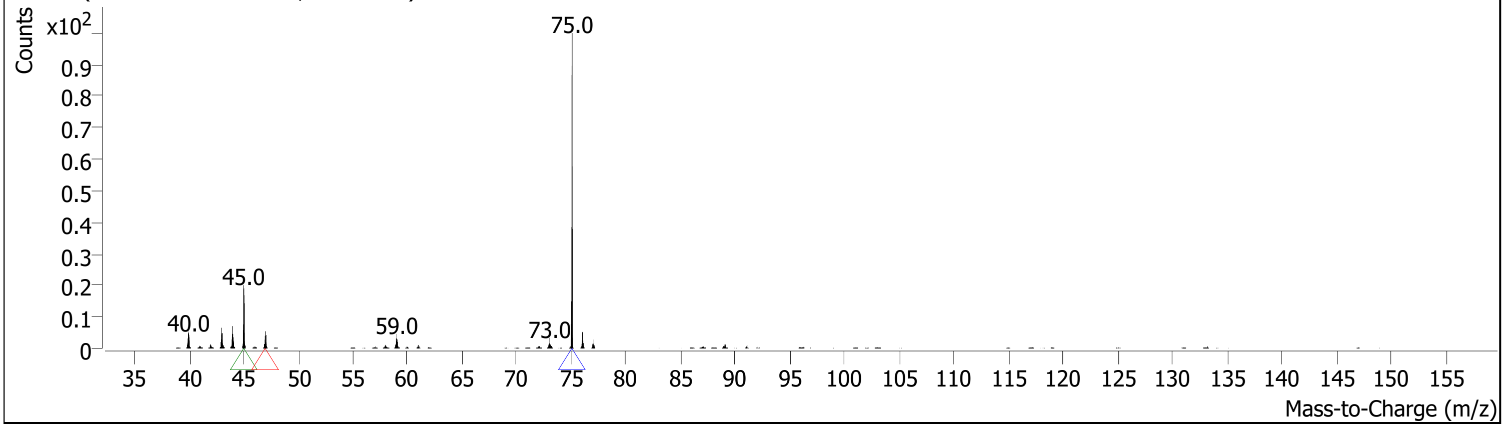


Component RT: 12.2444

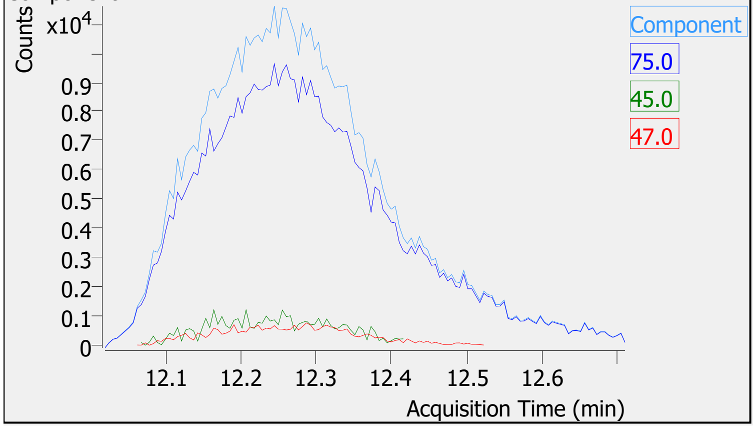


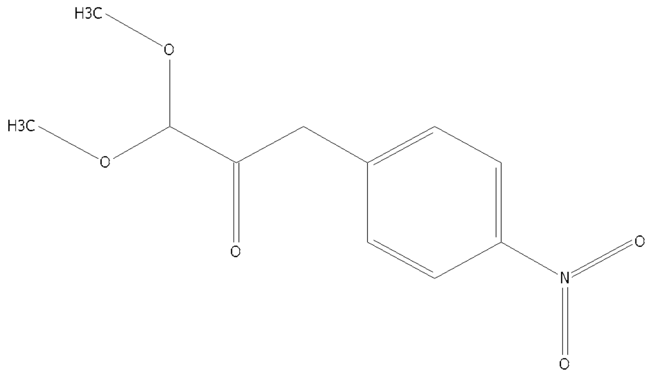

| RT | Compound Name | CAS# | Formula | Area | MI | Match Score | Sample | Sample |
|---|---|---|---|---|---|---|---|---|
| 14.0400 | Cyclopropane-1,2,3-D3-methanol, (1.alpha.,2.beta.,3.beta.)- | 126036-54-2 | C4H5D3O | 54885 | | 85.3 | 0.02 | 0.02 |

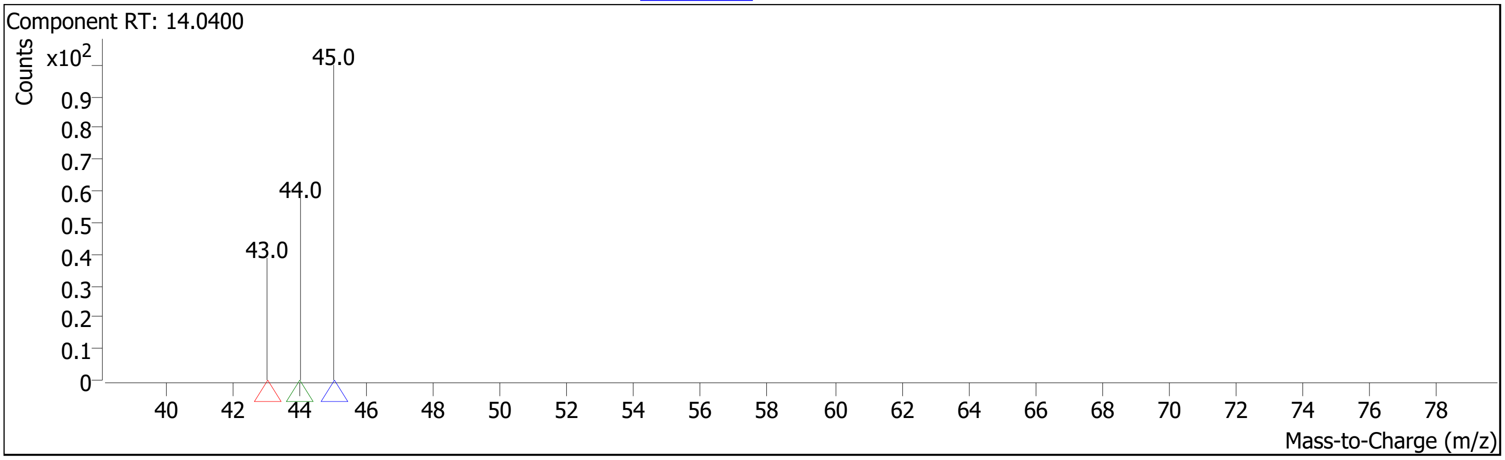


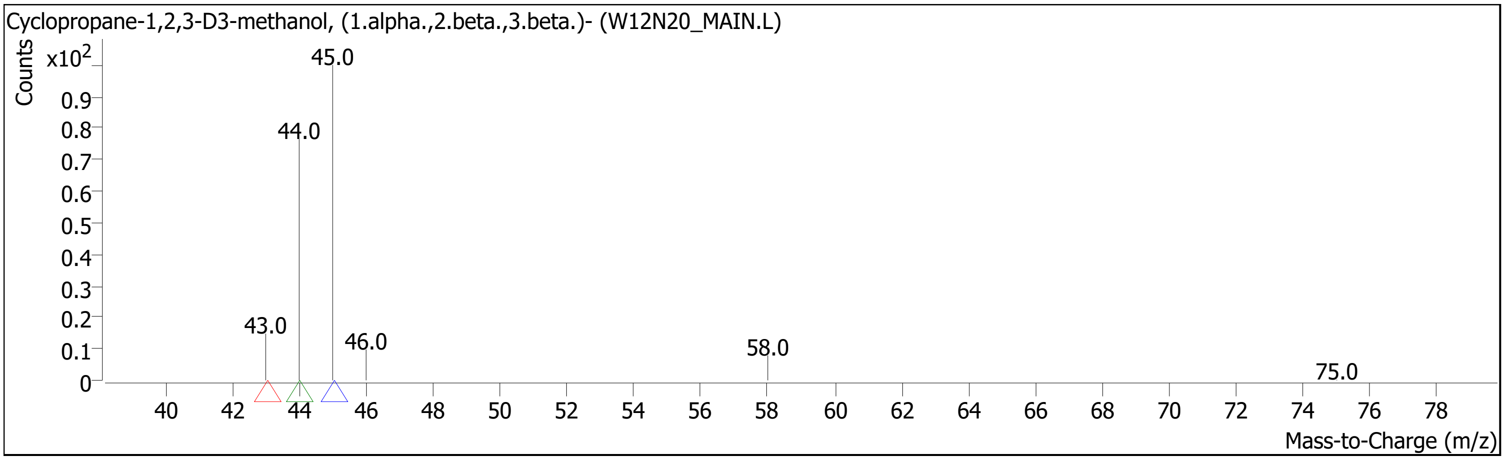


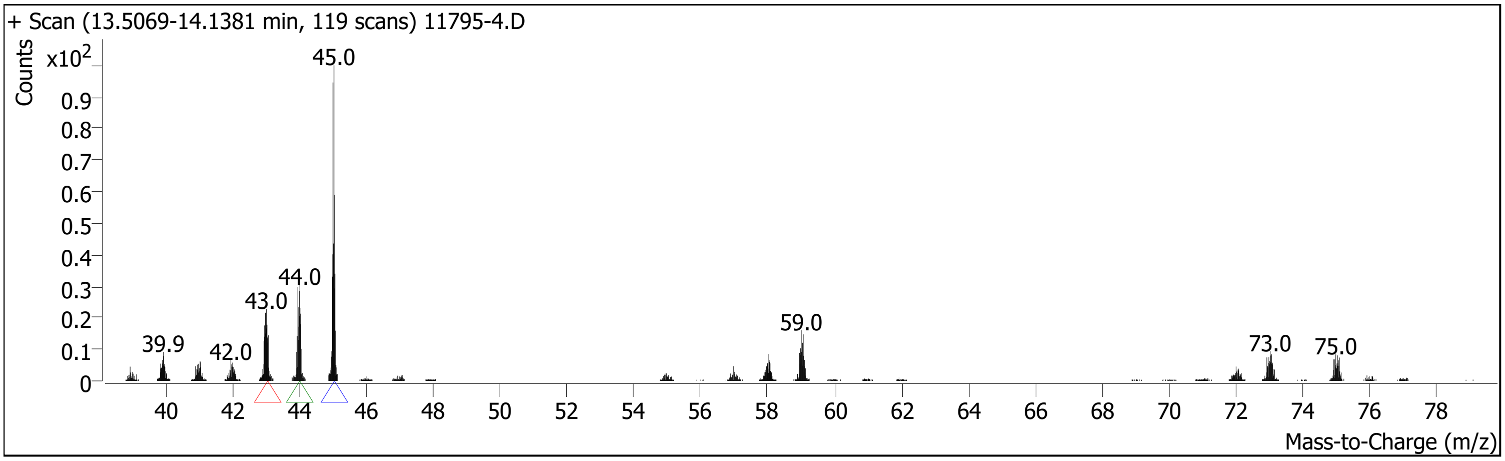


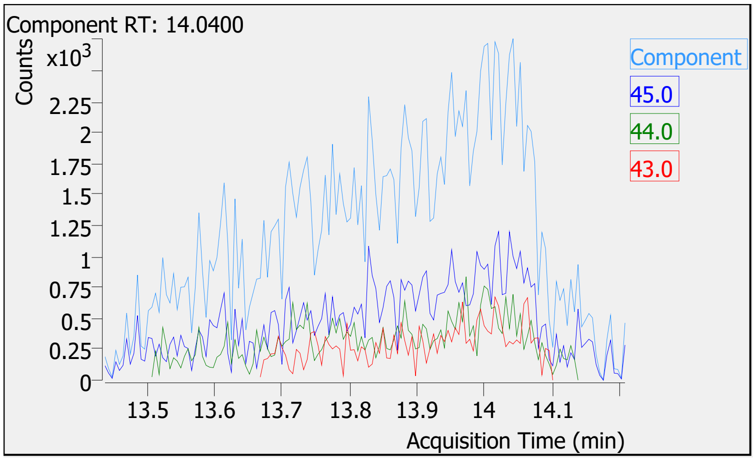


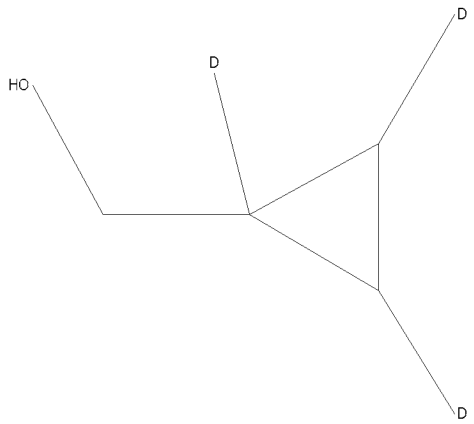

# Unknown Analysis Report - Best Hits

| RT | Compound Name | CAS# | Formula | Area | MI | Match Score | Sample | Sample |
|---|---|---|---|---|---|---|---|---|
| 30.6245 | Estra-1,3,5(10)-trien-17-one, 3,4-bis[(trimethylsilyl)oxy]- | 51497-39-3 | C24H38O3Si2 | 88374 | | 86.7 | 0.03 | 0.04 |

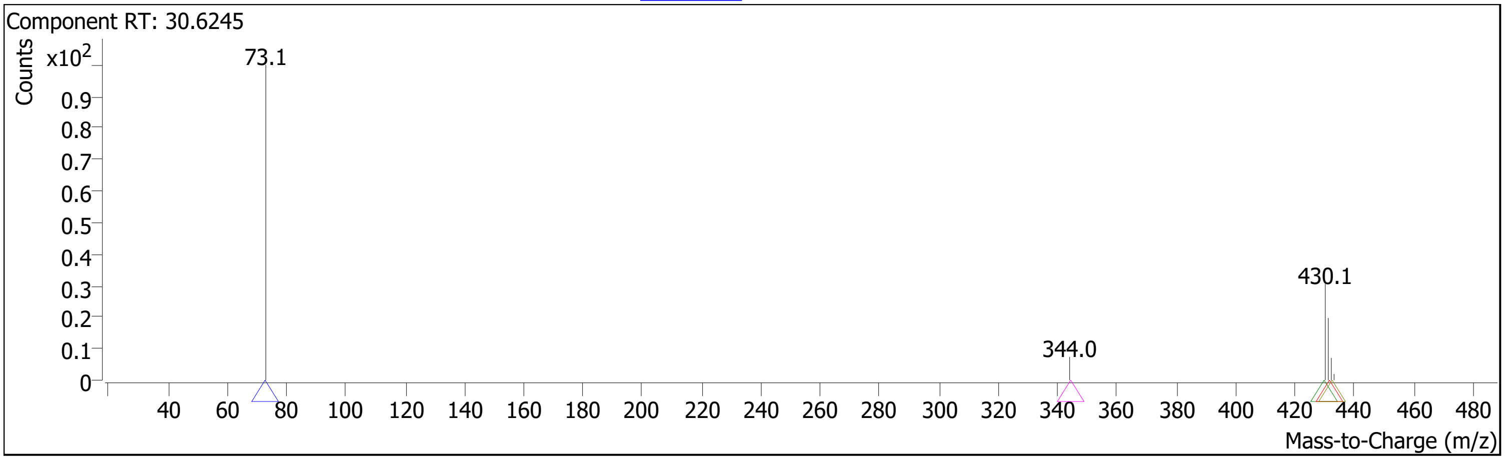


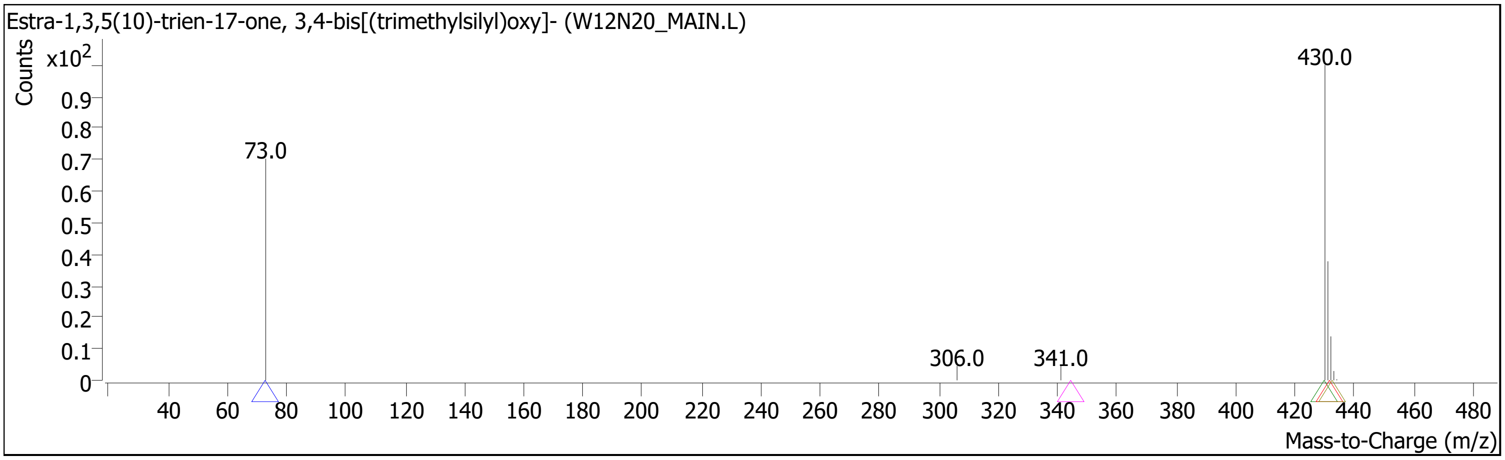


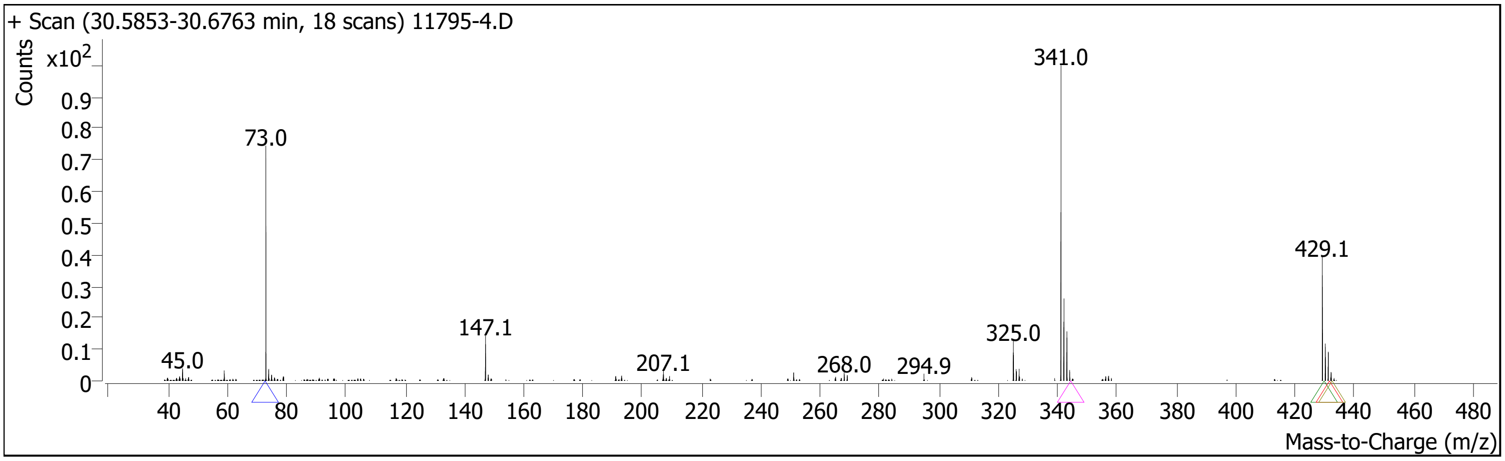


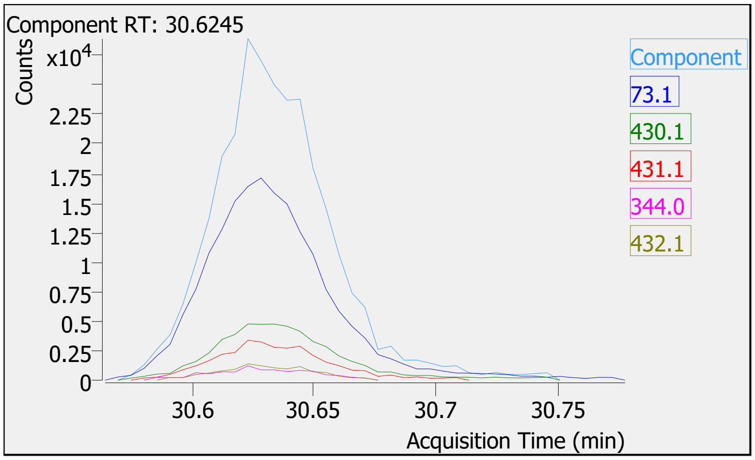


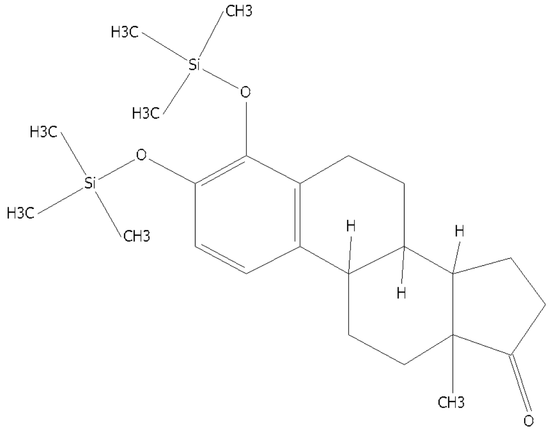

 Generated at 4:37 PM on 11/14/2023

# Unknown Analysis Report - Best Hits

| RT | Compound Name | CAS# | Formula | Area | MI | Match Score | Sample | Sample |
|---|---|---|---|---|---|---|---|---|
| 30.6324 | 6-Fluorobenzo[4',5']thieno[2',3':4,5]pyrrolo[1,2-f]phenanthridine | 990404-64-3 | C22H12FNS | 67882 | | 81.2 | 0.02 | 0.03 |

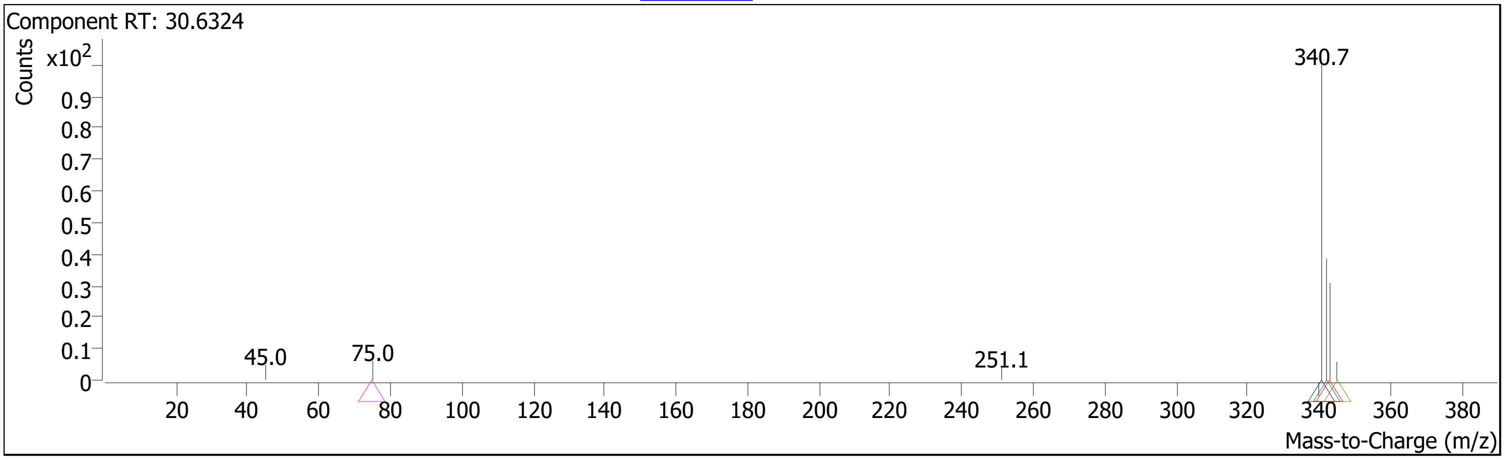


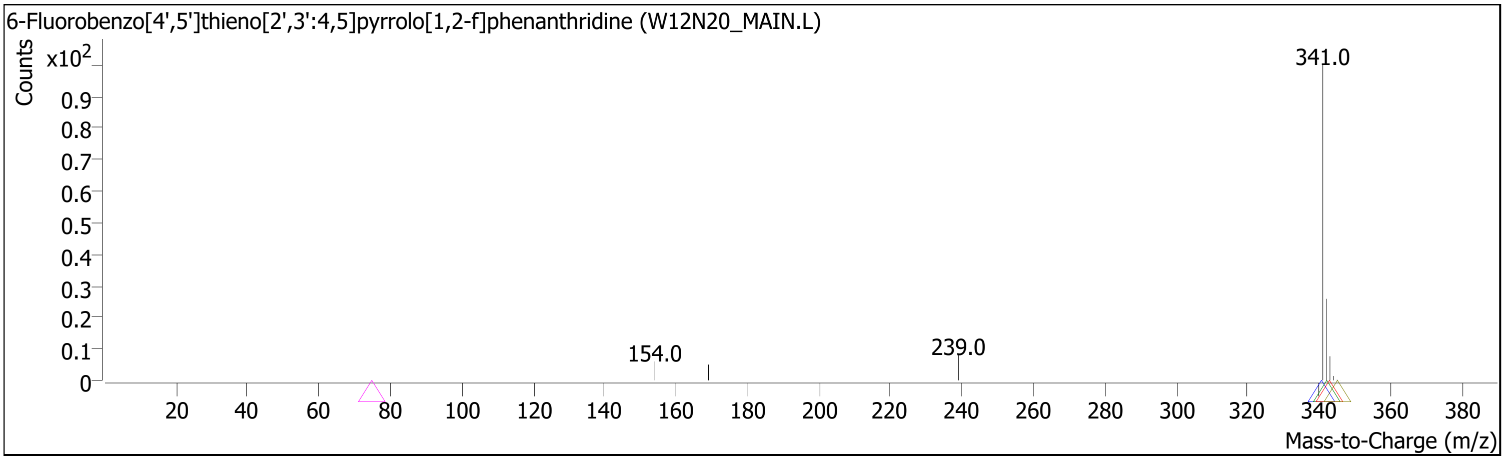


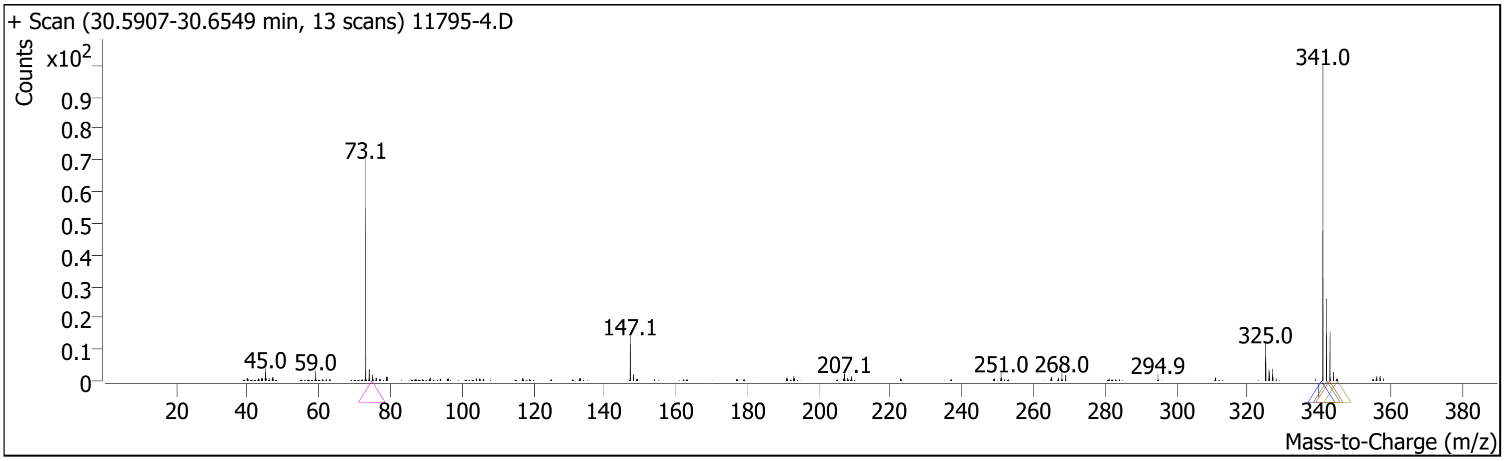


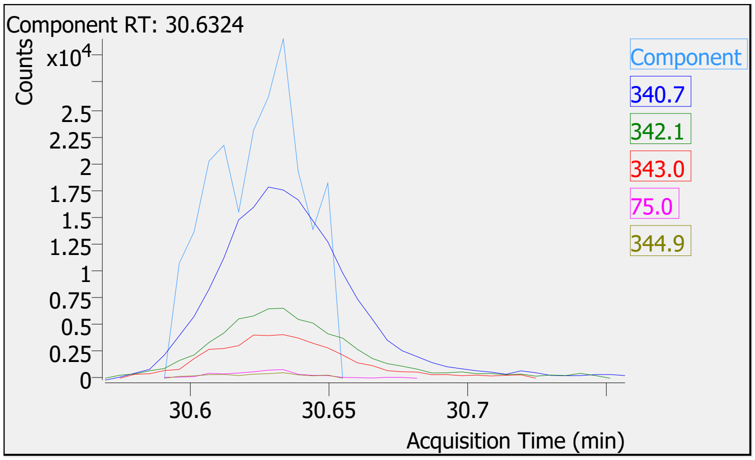


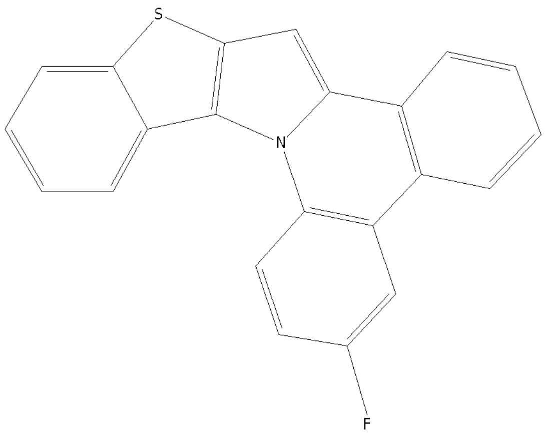

# Unknown Analysis Report - Best Hits

| RT | Compound Name | CAS# | Formula | Area | MI | Match Score | Sample | Sample |
|---|---|---|---|---|---|---|---|---|
| 36.1431 | 1,2-Di-tert-butylbenzene | 1012-76-6 | C14H22 | 84432 | | 89.0 | 0.03 | 0.03 |

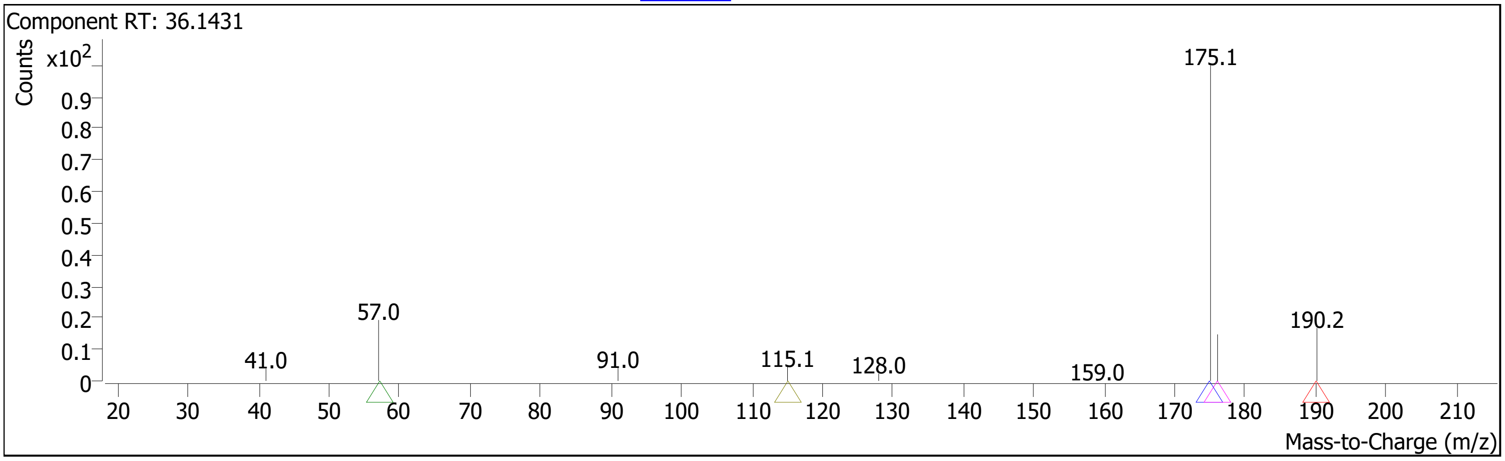


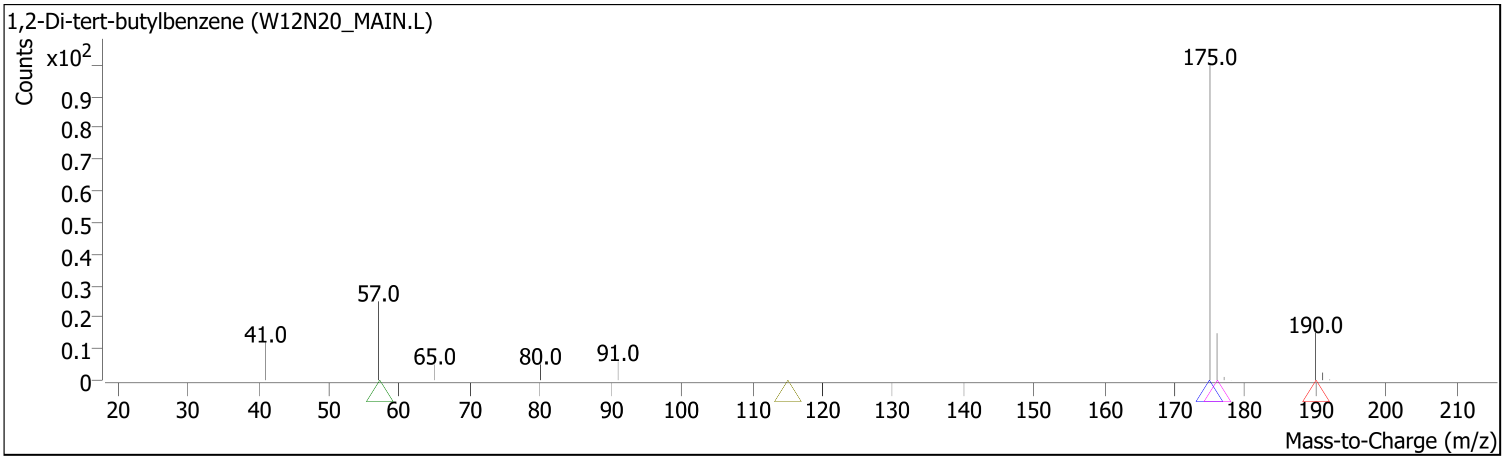


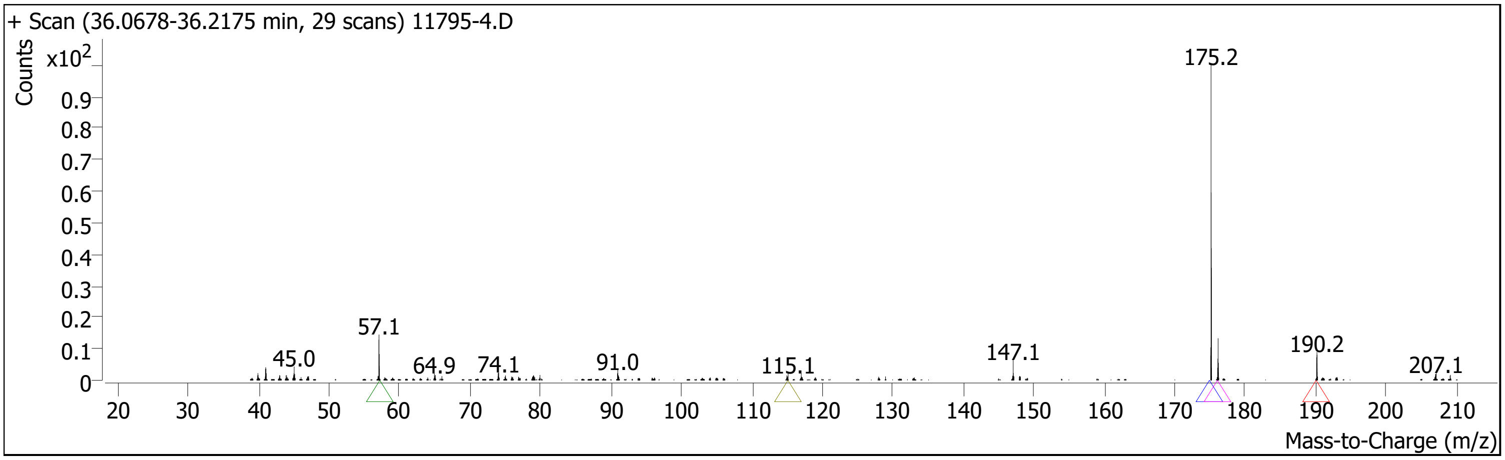


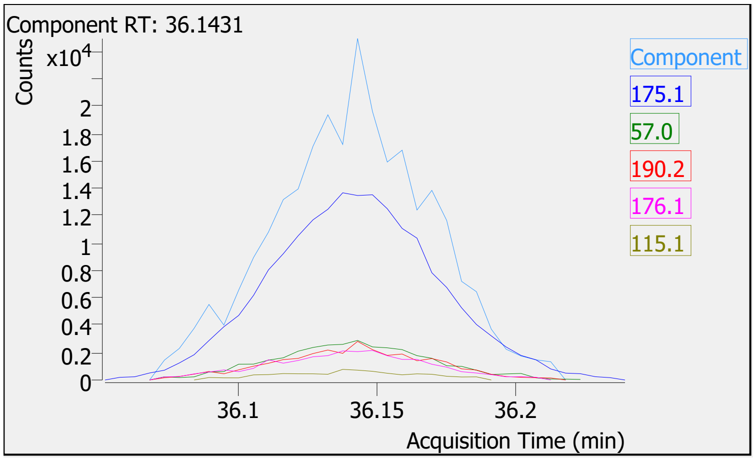


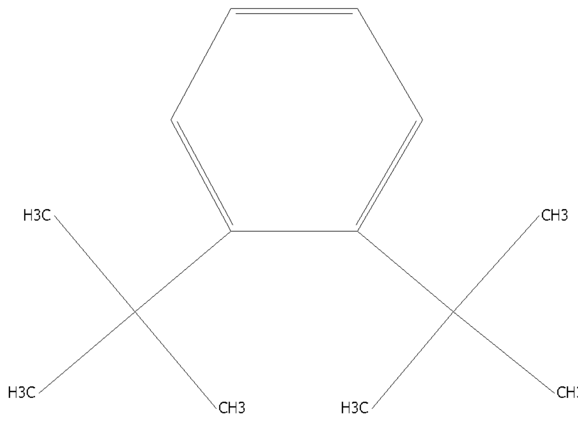

# Unknown Analysis Report - Best Hits

| RT | Compound Name | CAS# | Formula | Area | MI | Match Score | Sample | Sample |
|---|---|---|---|---|---|---|---|---|
| 41.8725 | (S*,S*)-2-Hydroxy(4-methoxy-2-trimethylsilylphenyl)methyl-1-cycloheptanone | 990354-28-4 | C18H28O3Si | 21579 | | 91.6 | 0.01 | 0.01 |

Component RT: 41.8725

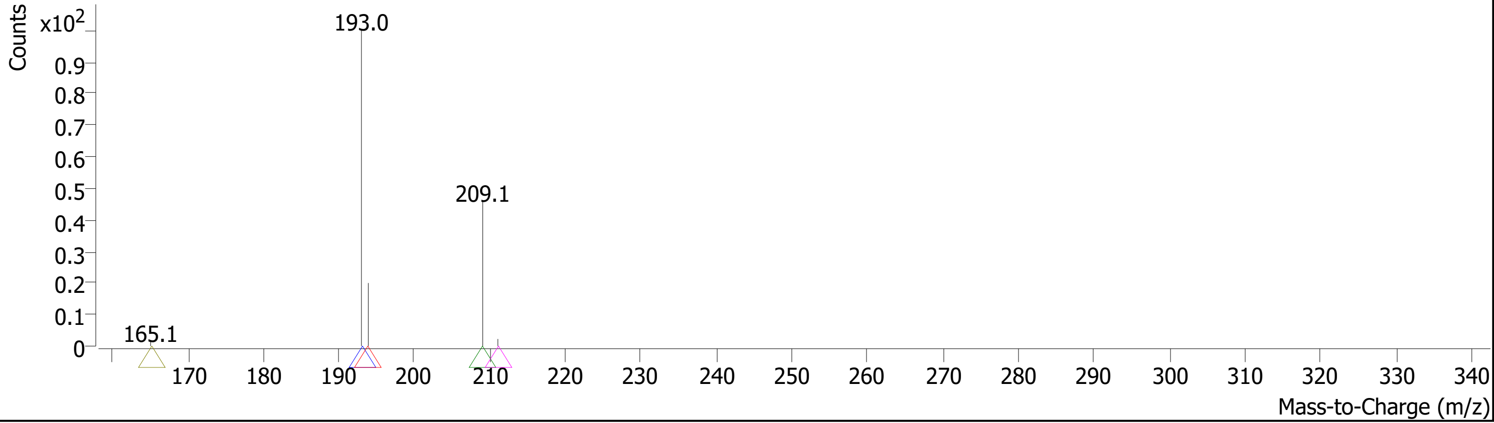


(S*,S*)-2-Hydroxy(4-methoxy-2-trimethylsilylphenyl)methyl-1-cycloheptanone (W12N20_MAIN.L)

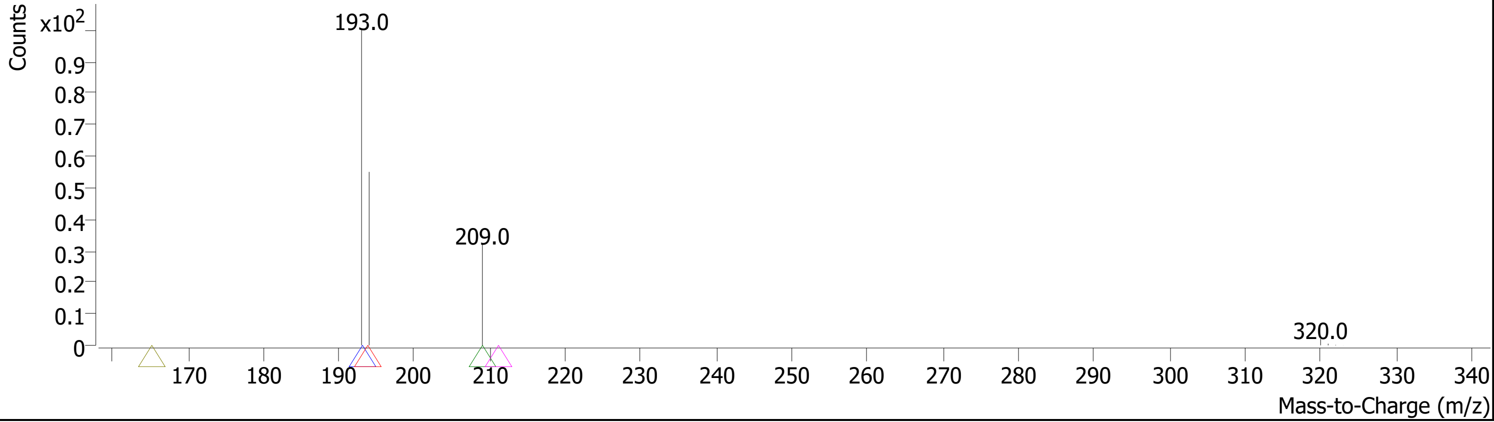


+ Scan (41.8658-41.8872 min, 4 scans) 11795-4.D

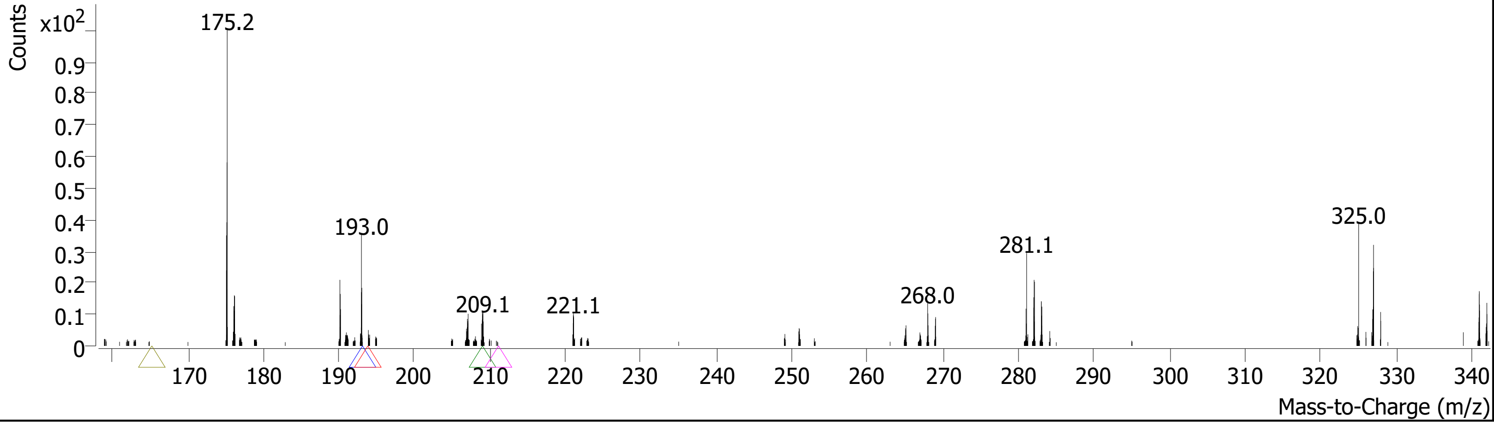


Component RT: 41.8725

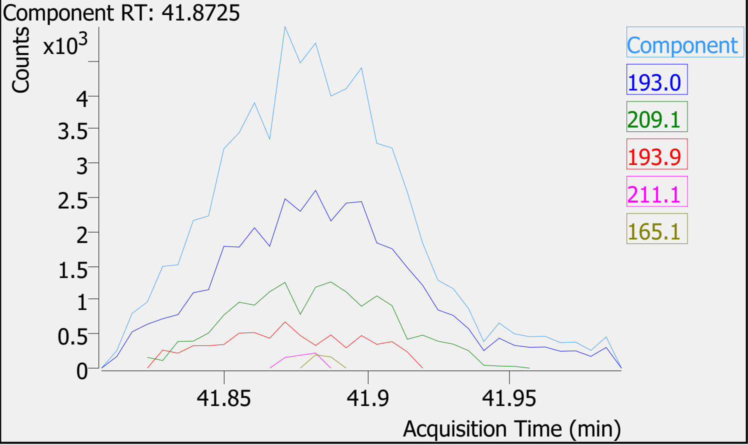


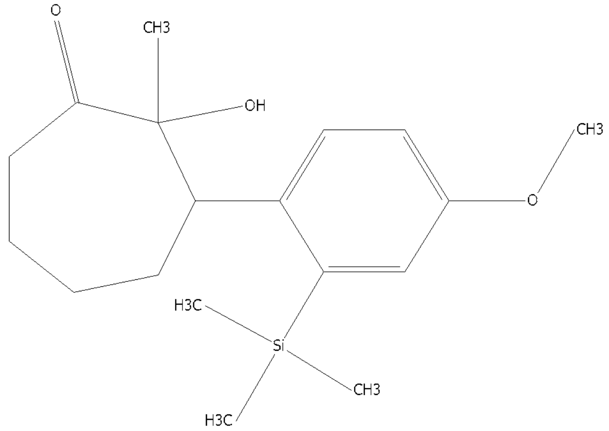


 Generated at 4:37 PM on 11/14/2023

| RT | Compound Name | CAS# | Formula | Area | MI | Match Score | Sample | Sample |
|---|---|---|---|---|---|---|---|---|
| 45.6975 | 2,5-trans-Bis(4-methanethiosulfonylmethylphenyl)-2,5-dimethylpyrrolidin-1-yloxyl radical | 990614-54-3 | C22H28NO5S4 | 15256583 | | 75.9 | 5.04 | 6.08 |

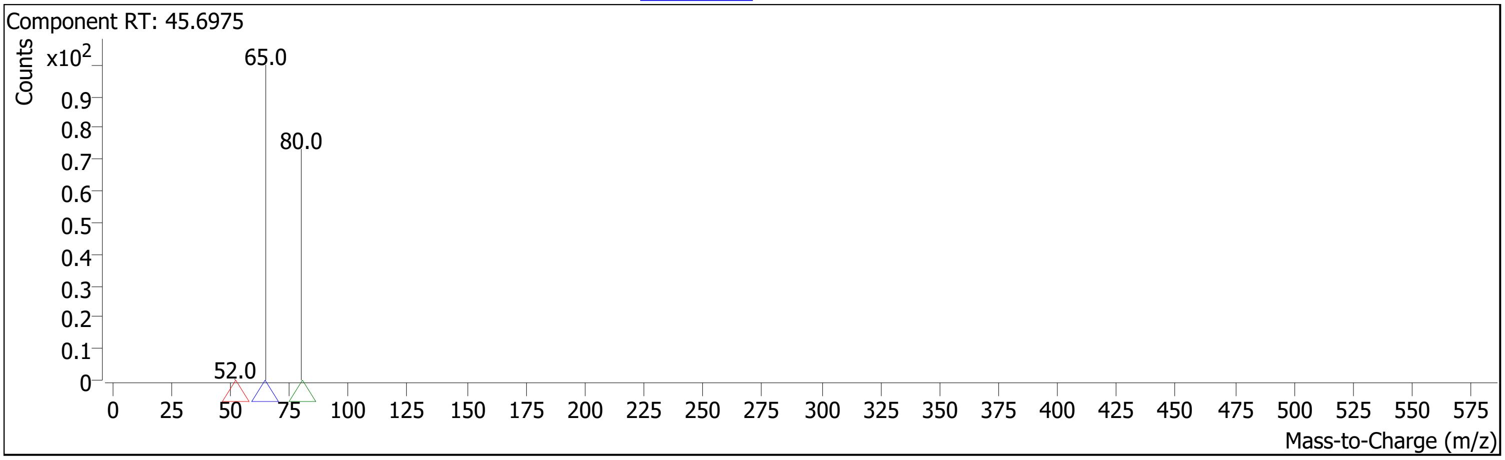


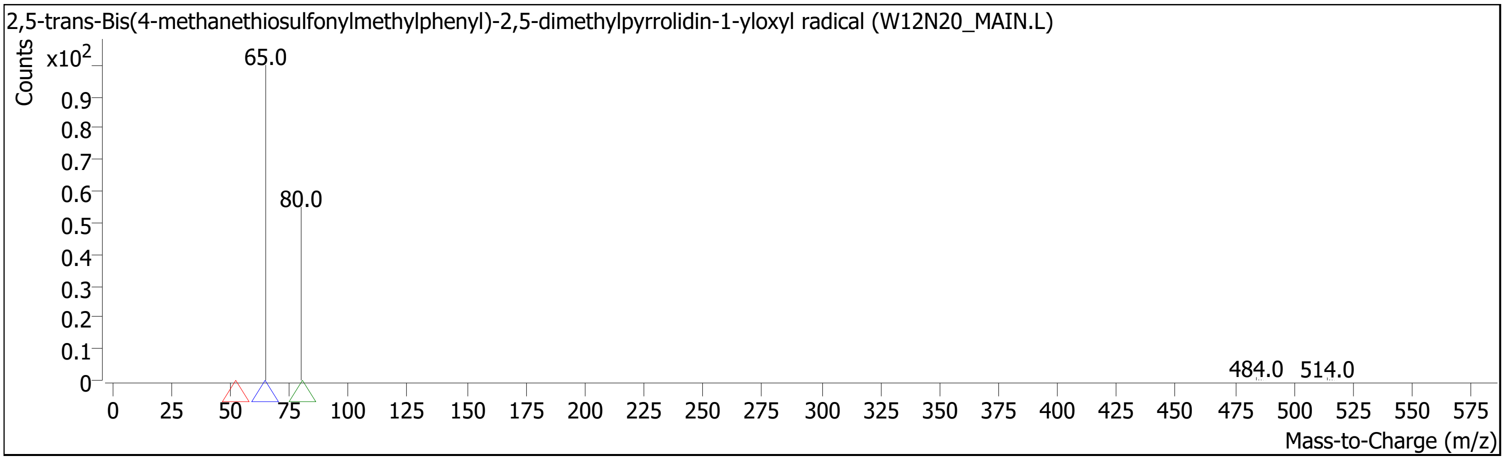


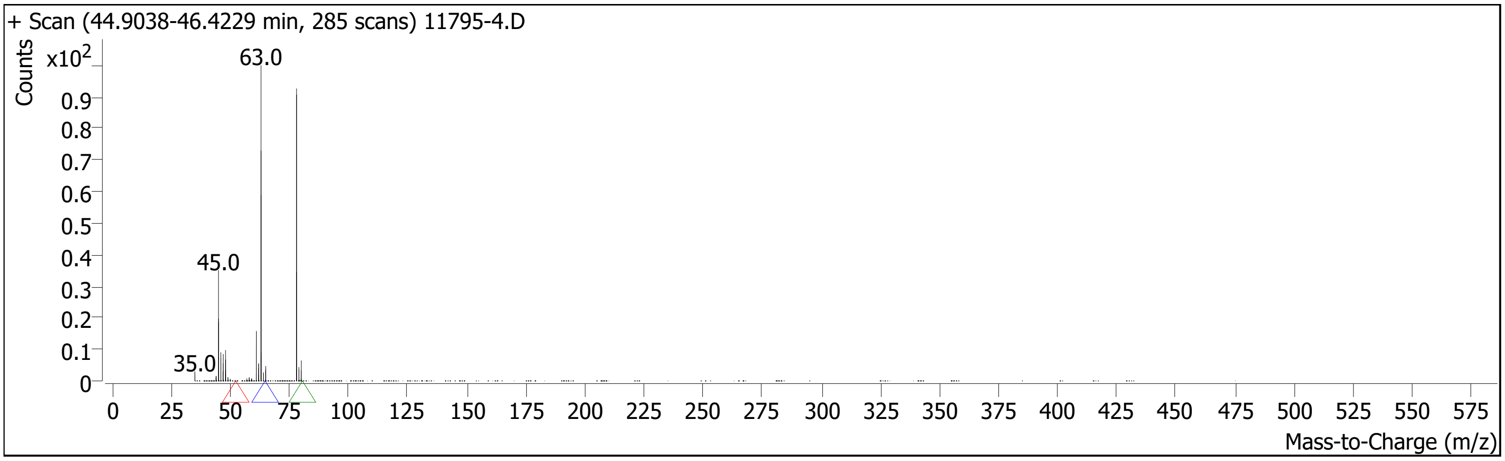


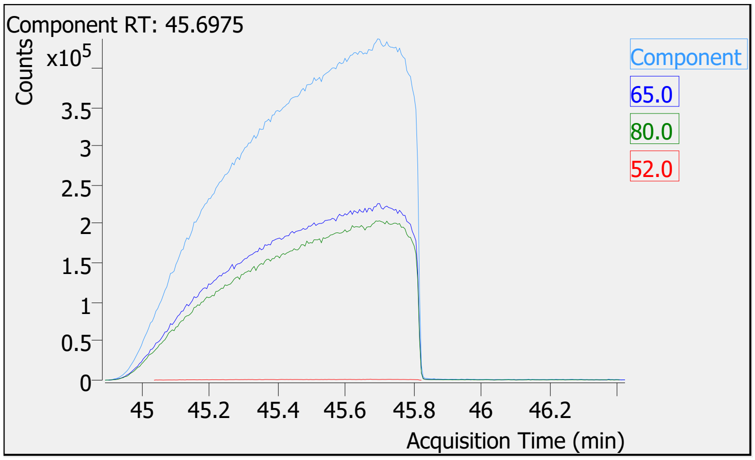


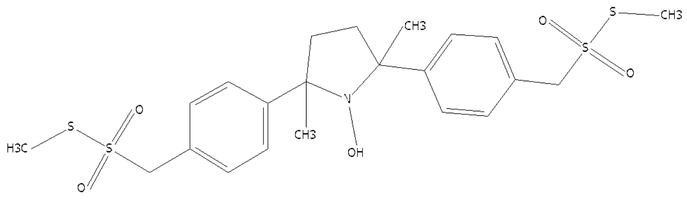


 Generated at 4:37 PM on 11/14/2023

# Unknown Analysis Report - Best Hits

| RT | Compound Name | CAS# | Formula | Area | MI | Match Score | Sample | Sample |
|---|---|---|---|---|---|---|---|---|
| 45.7878 | Methanethiol | 74-93-1 | CH4S | 32734053 | | 75.5 | 10.82 | 13.05 |

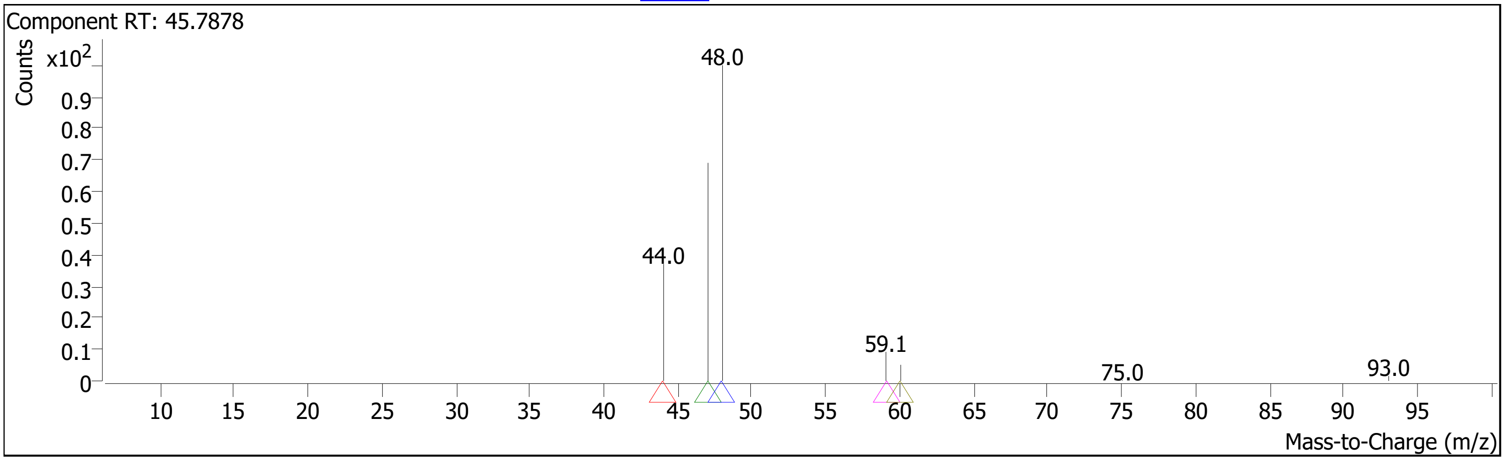


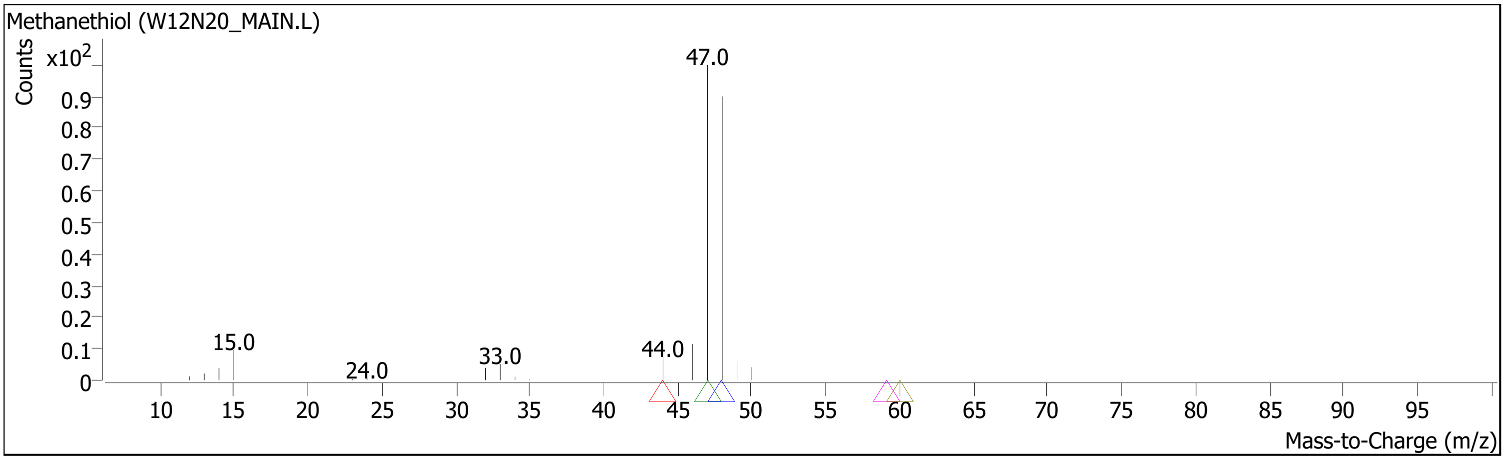


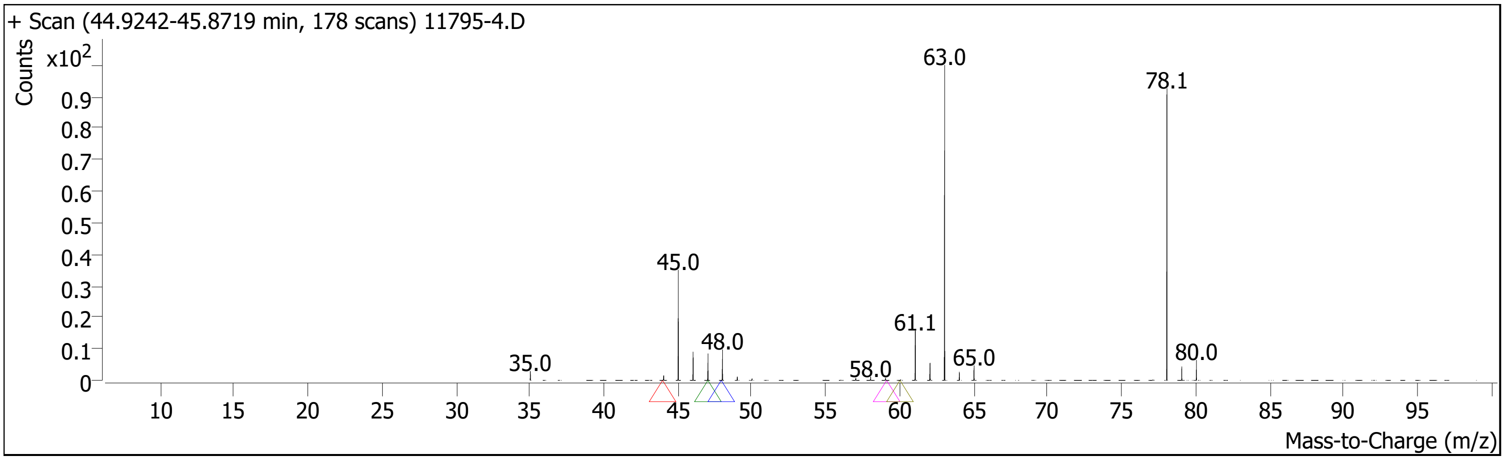


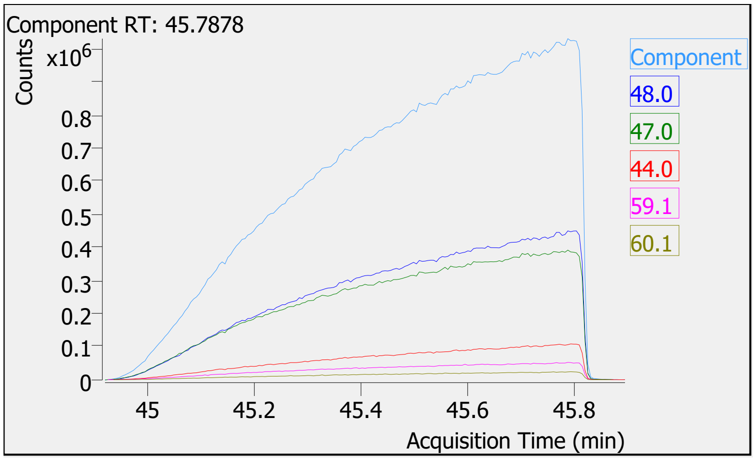


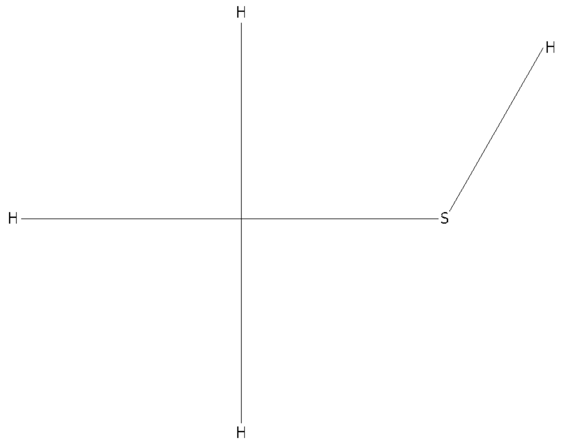

# Unknown Analysis Report - Best Hits

| RT | Compound Name | CAS# | Formula | Area | MI | Match Score | Sample | Sample |
|---|---|---|---|---|---|---|---|---|
| 45.8008 | Methoxypropionaldehyde | 990000-34-9 | C4H8O2 | 250929967 | | 91.7 | 82.92 | 100.00 |

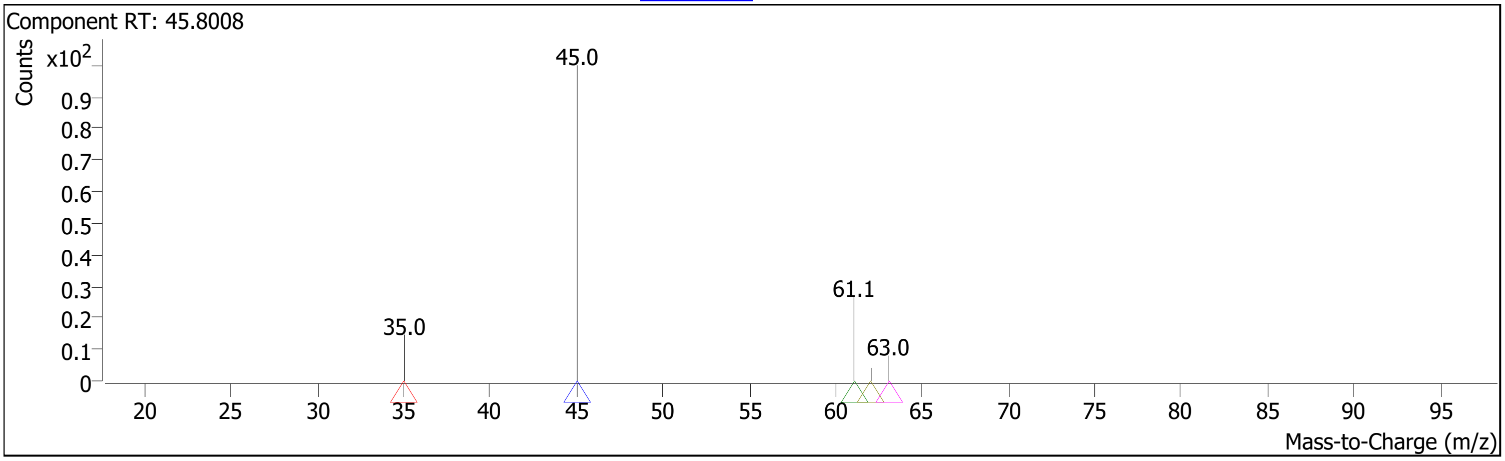


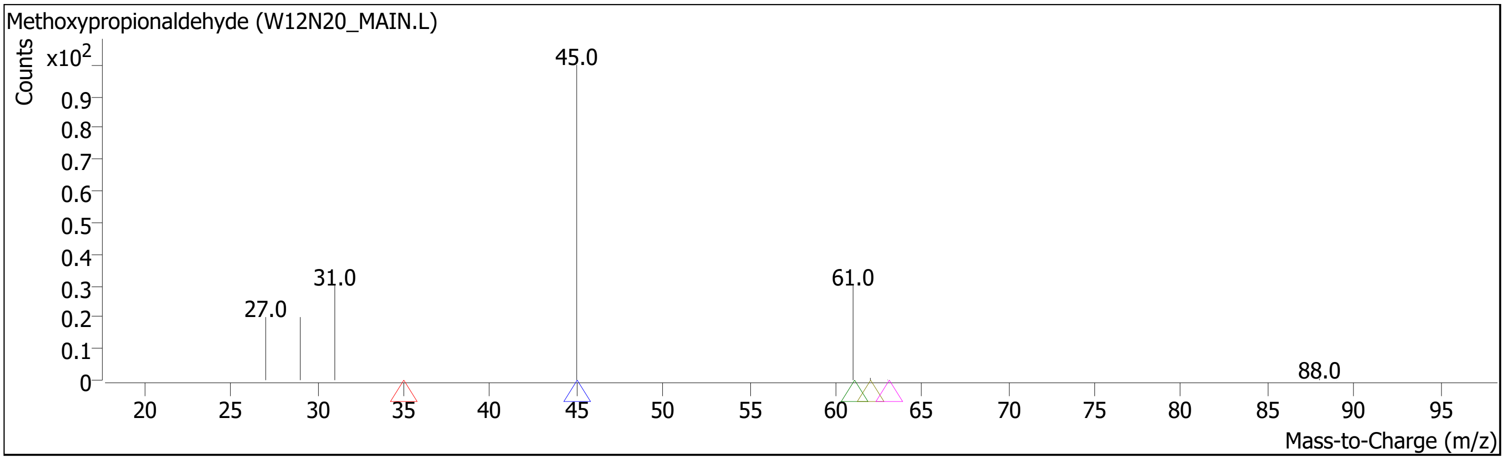


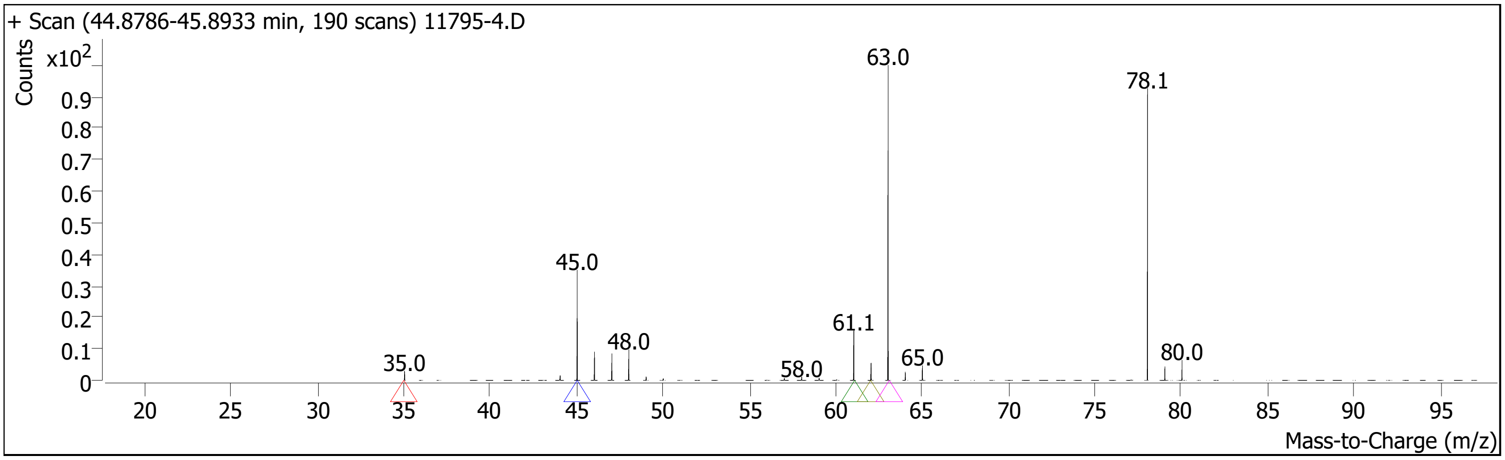


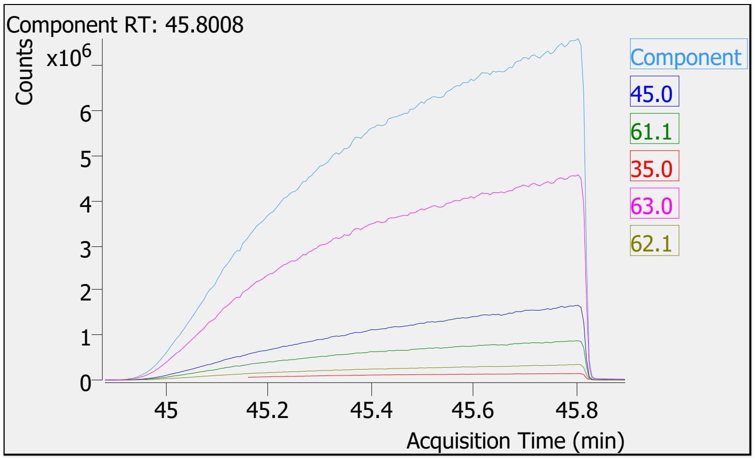


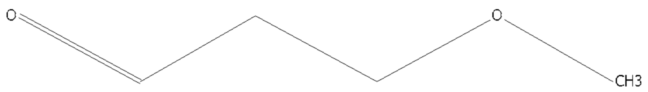

# Unknown Analysis Report - Best Hits

| RT | Compound Name | CAS# | Formula | Area | MI | Match Score | Sample | Sample |
|---|---|---|---|---|---|---|---|---|
| 47.7331 | N(1),N(2)-Dibenzoyl-N(1),N(2)-dimethoxy-hydrazine | 990304-14-7 | C16H16N2O4 | 14221 | | 93.3 | 0.00 | 0.01 |

Component RT: 47.7331

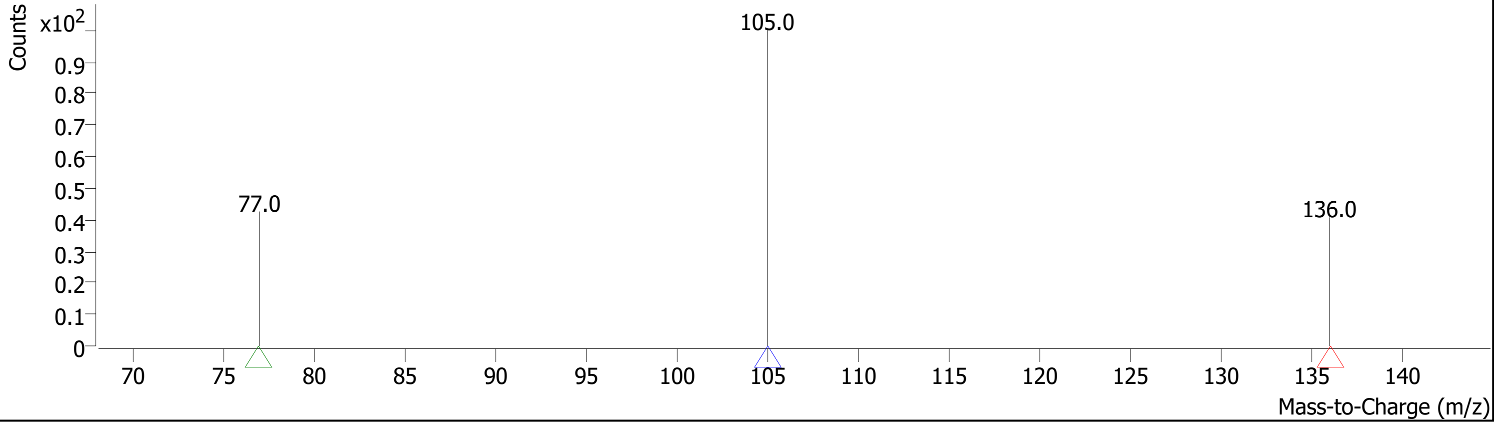


N(1),N(2)-Dibenzoyl-N(1),N(2)-dimethoxy-hydrazine (W12N20_MAIN.L)

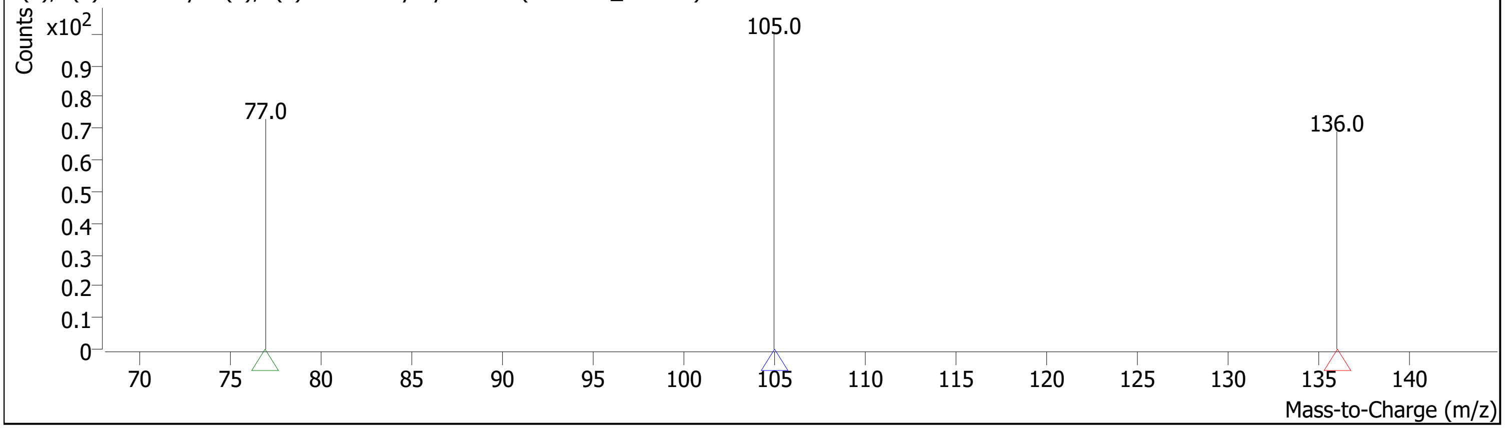


+ Scan (47.6691-47.8403 min, 33 scans) 11795-4.D

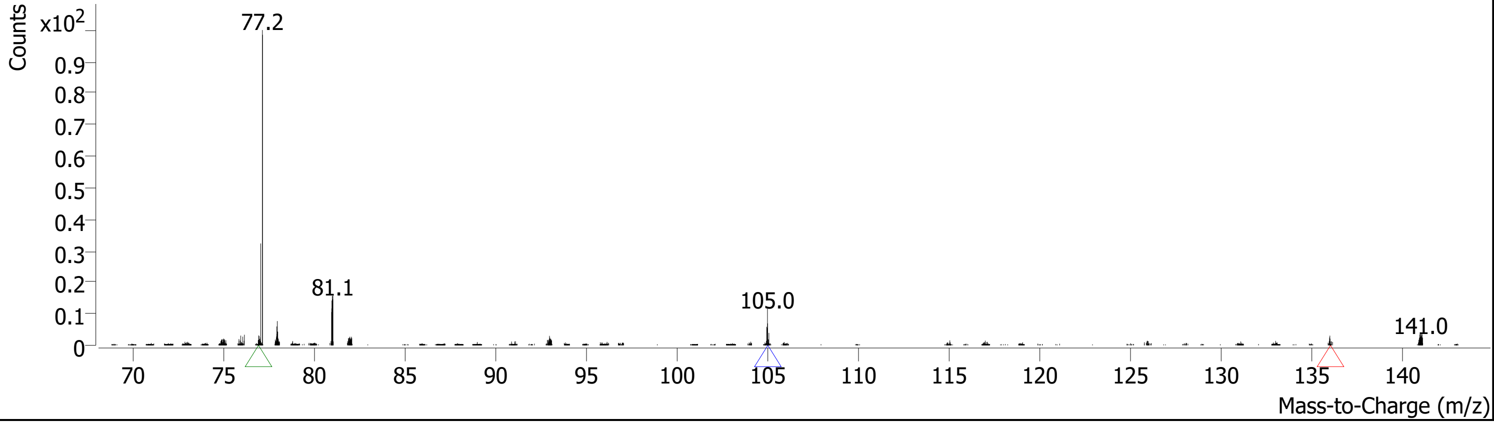


Component RT: 47.7331

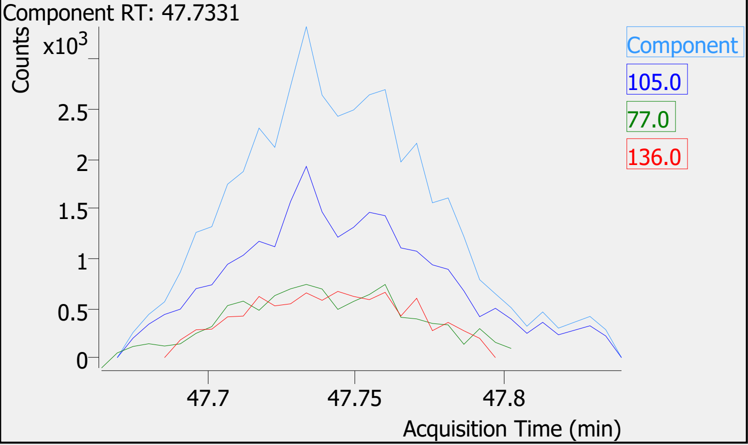


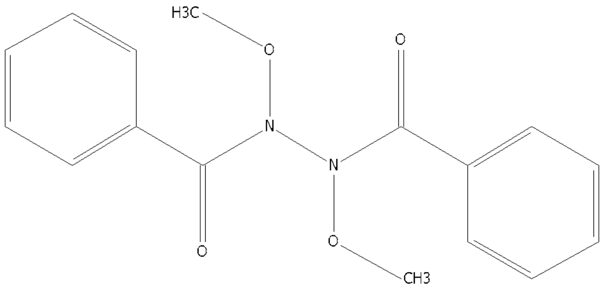

| RT | Compound Name | CAS# | Formula | Area | MI | Match Score | Sample | Sample |
|---|---|---|---|---|---|---|---|---|
| 49.5538 | Silanediol, dimethyl- | 1066-42-8 | C2H8O2Si | 288077 | | 93.2 | 0.10 | 0.11 |

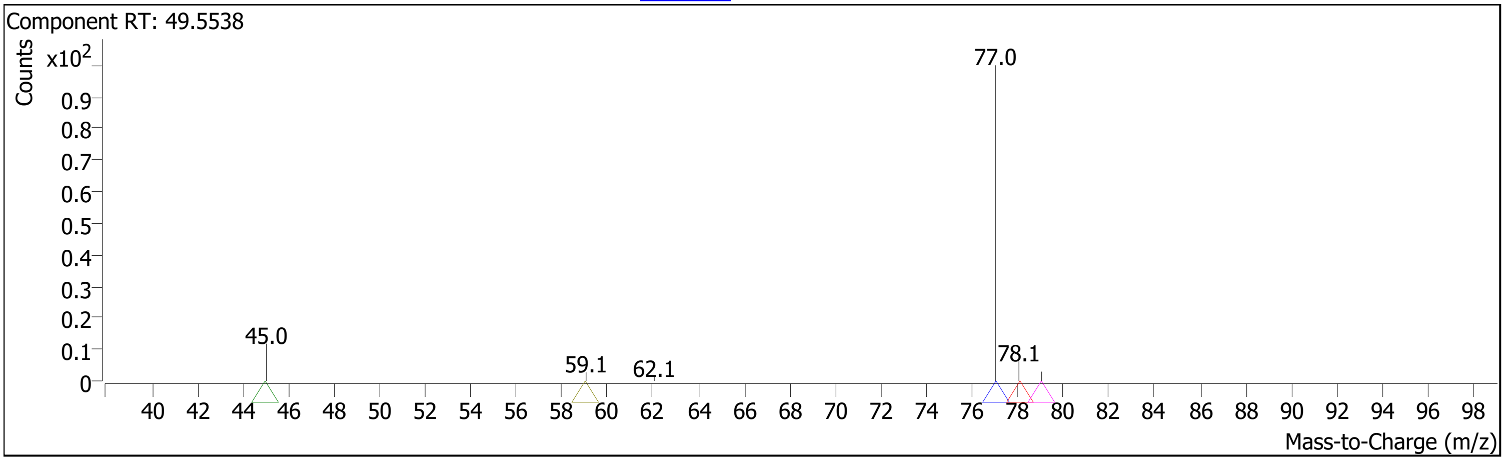


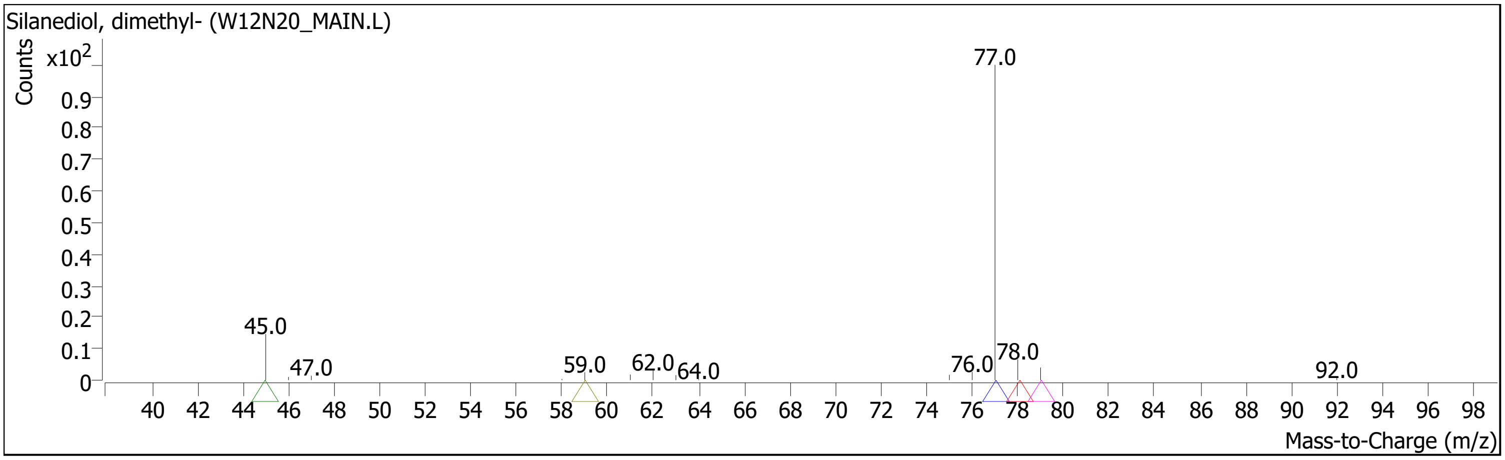


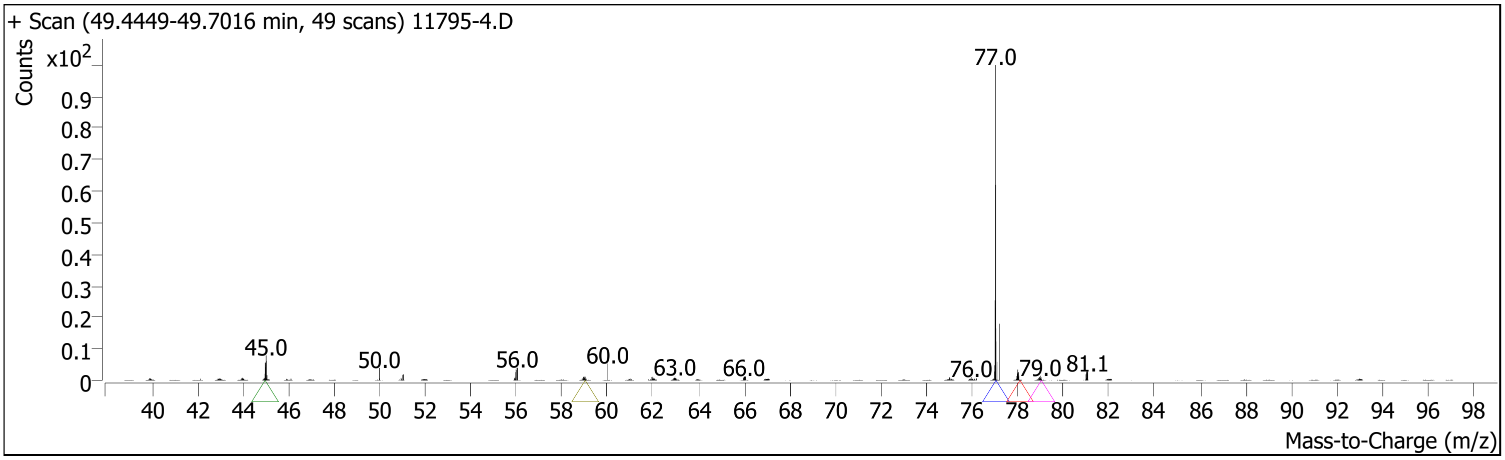


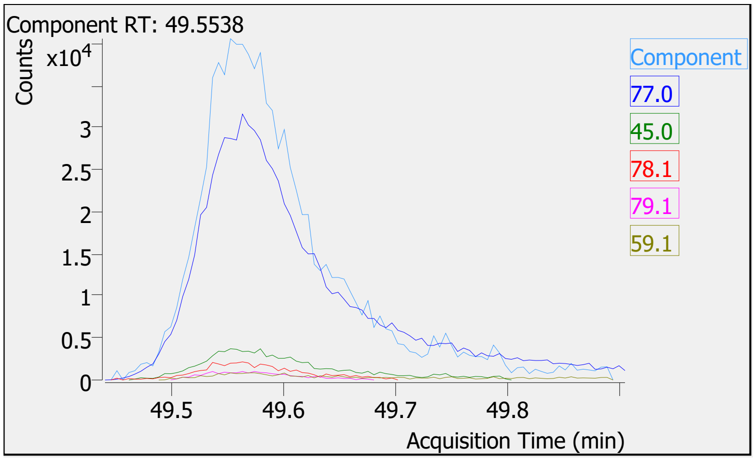


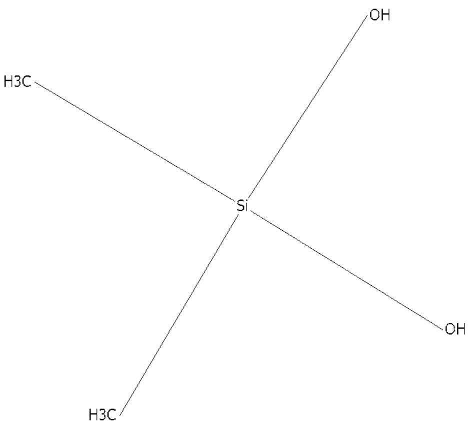


 Generated at 4:37 PM on 11/14/2023

# Unknown Analysis Report - Best Hits

| RT | Compound Name | CAS# | Formula | Area | MI | Match Score | Sample | Sample |
|---|---|---|---|---|---|---|---|---|
| 51.1363 | N-[2-[3,5-bis(trimethylsilyloxy)phenyl]-2-trimethylsilyloxy-ethyl]-N-tert-butyl-2,2,2-tris(fluoranyl)ethanamide | 325836-92-8 | C23H42F3NO4 Si3 | 32901 | | 72.4 | 0.01 | 0.01 |

Component RT: 51.1363

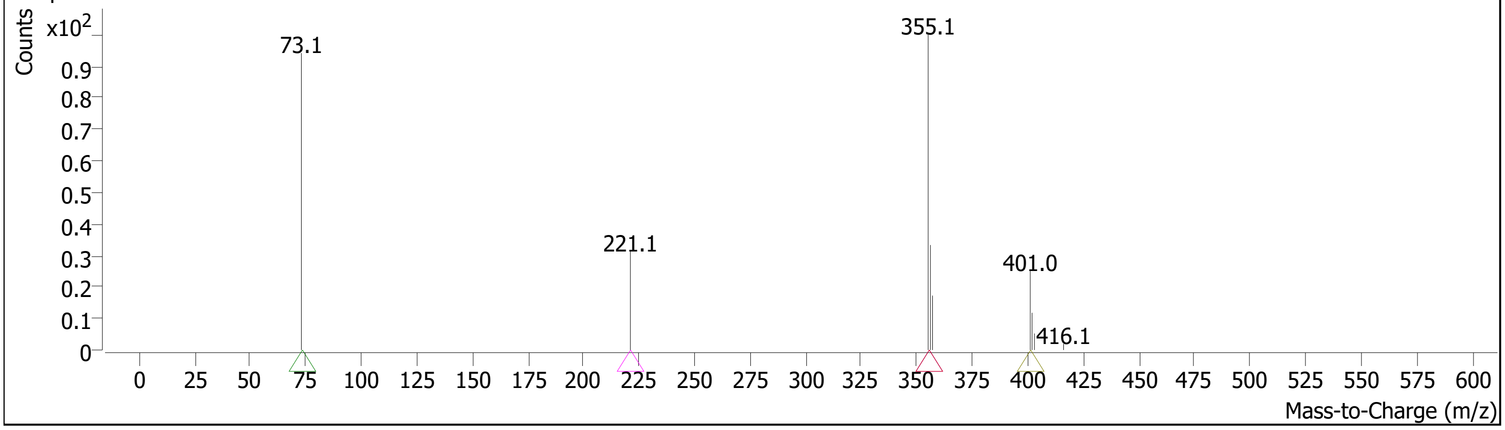


N-[2-[3,5-bis(trimethylsilyloxy)phenyl]-2-trimethylsilyloxy-ethyl]-N-tert-butyl-2,2,2-tris(fluoranyl)ethanamide (W12N20_MAIN.L)

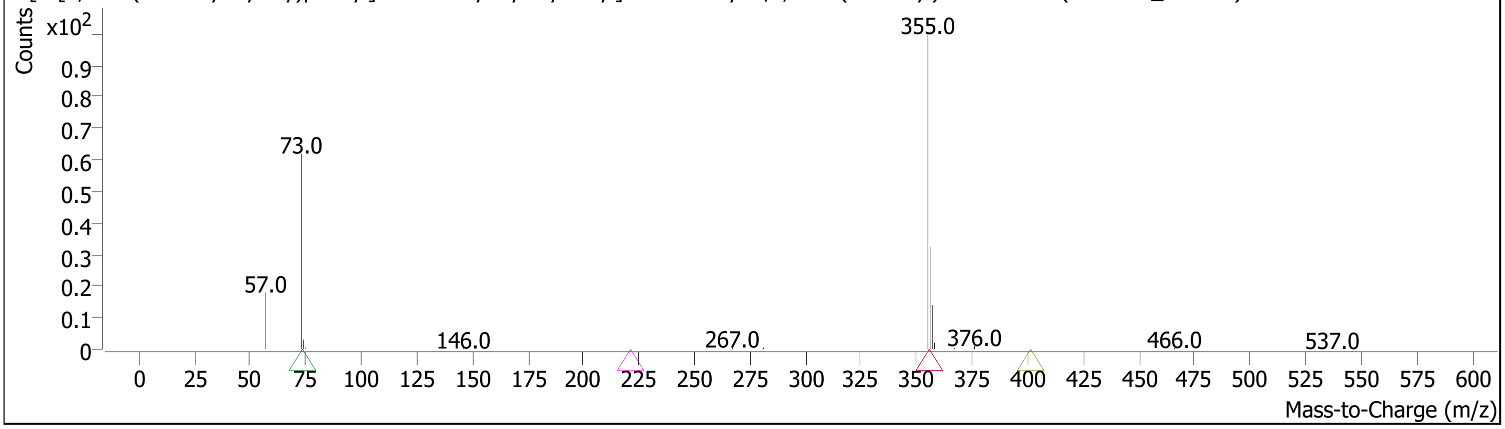


+ Scan (51.1083-51.1404 min, 7 scans) 11795-4.D

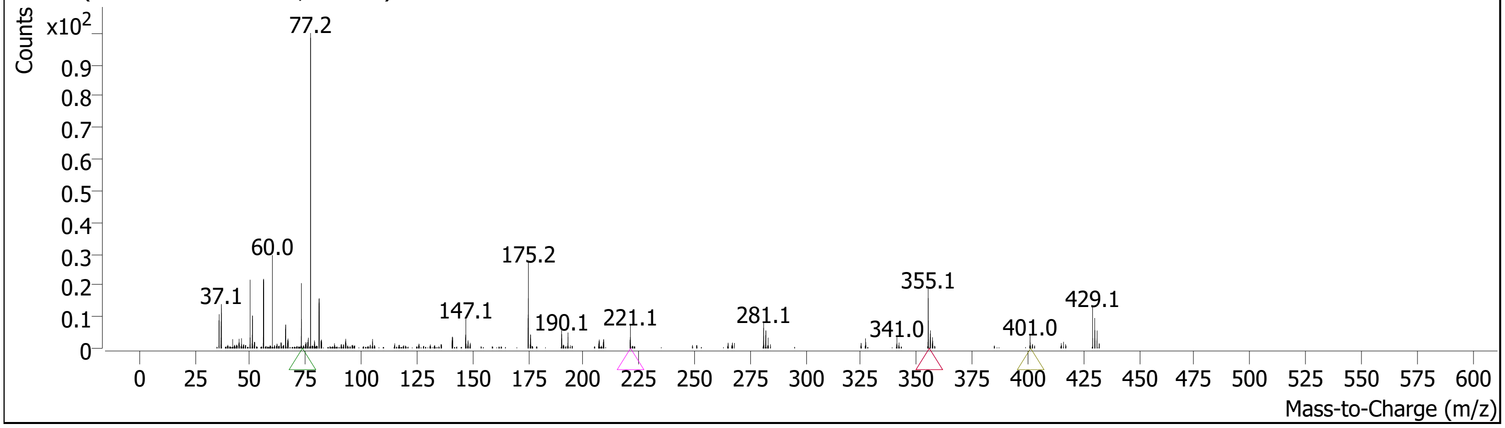


Component RT: 51.1363

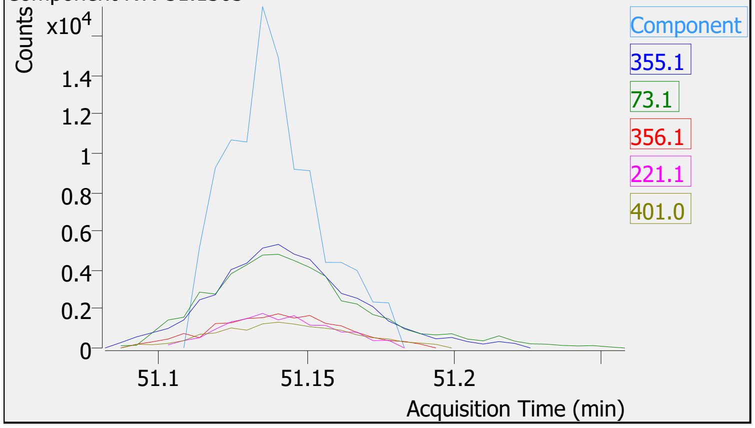


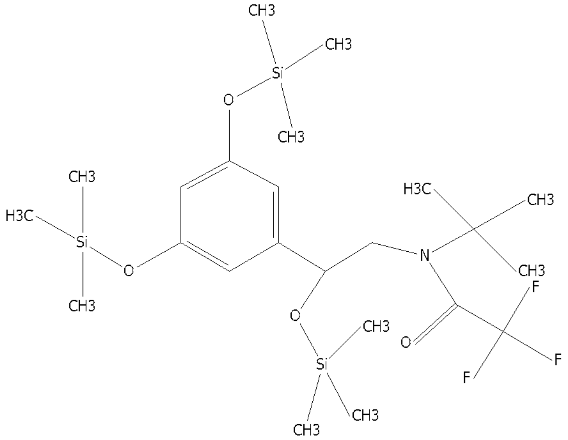


 Generated at 4:37 PM on 11/14/2023

# Unknown Analysis Report - Best Hits

| RT | Compound Name | CAS# | Formula | Area | MI | Match Score | Sample | Sample |
|---|---|---|---|---|---|---|---|---|
| 55.9743 | Benzeneacetic acid, .alpha.,2-dihydroxy-.alpha.,4-dimethyl-, ethyl ester, (R)- | 113322-78-4 | C12H16O4 | 34428 | | 73.5 | 0.01 | 0.01 |

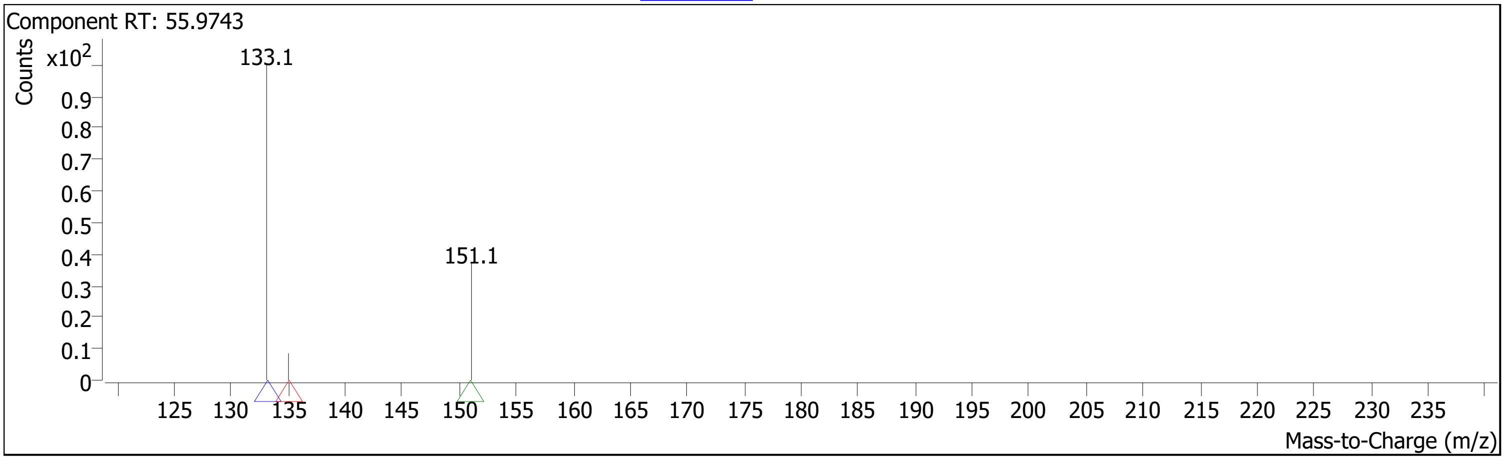


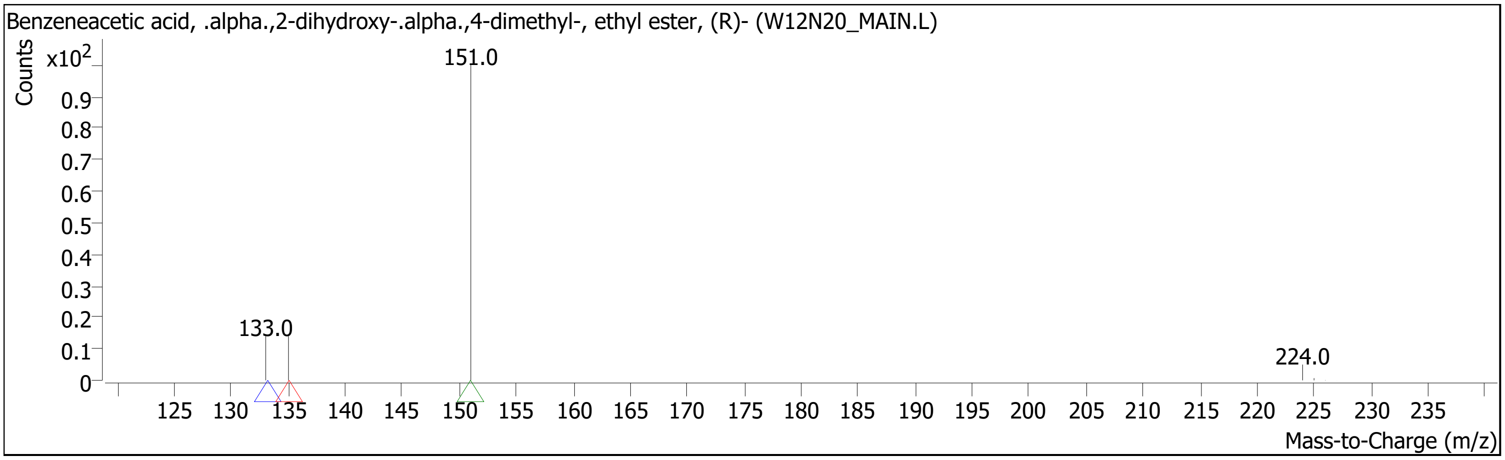


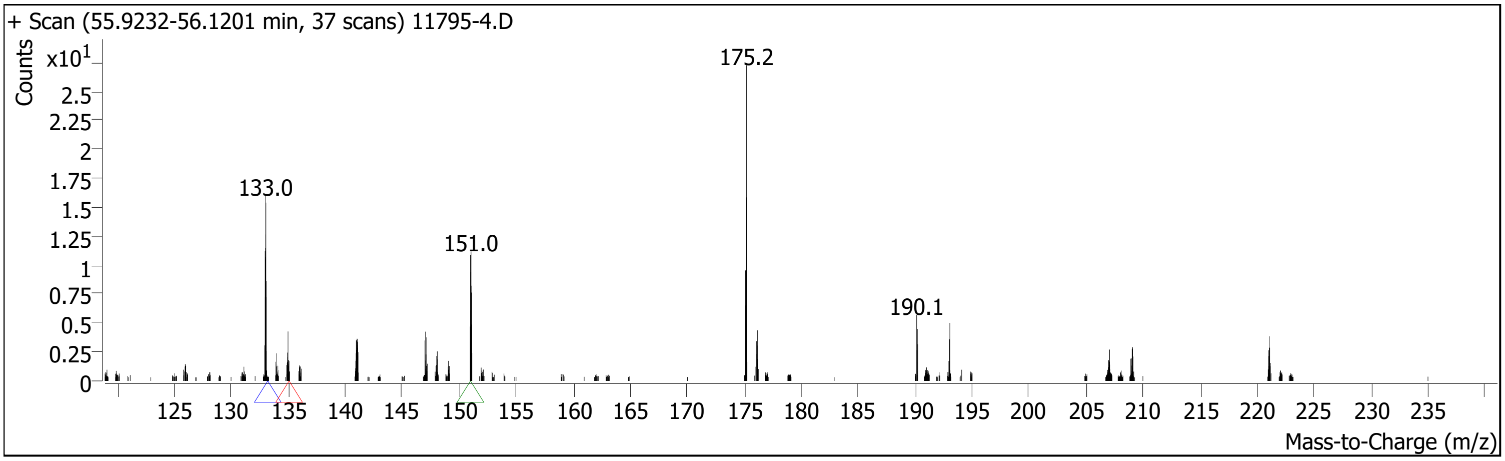


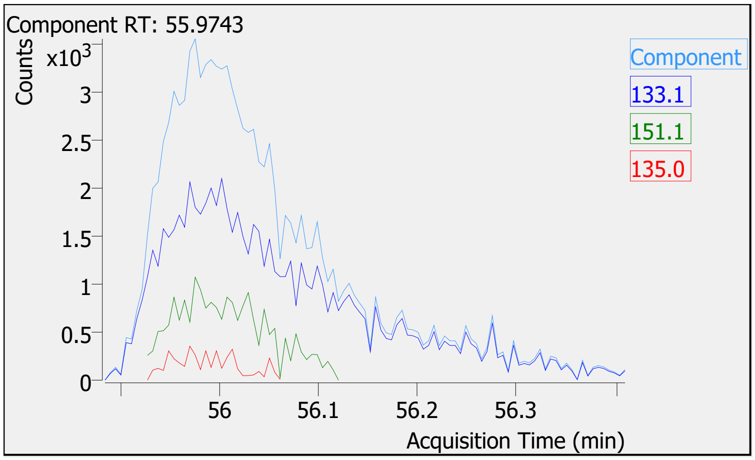


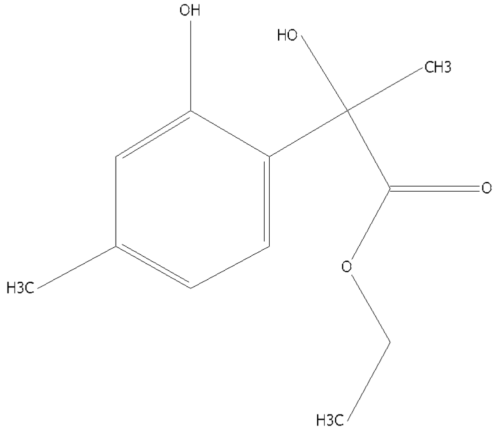

# Unknown Analysis Report - Best Hits

Agilent Trusted Answers

| RT | Compound Name | CAS# | Formula | Area | MI | Match Score | Sample | Sample |
|---|---|---|---|---|---|---|---|---|
| 59.7265 | Dimethyl Sulfoxide | 67-68-5 | C2H6OS | 1367829 | | 98.7 | 0.45 | 0.55 |

Component RT: 59.7265

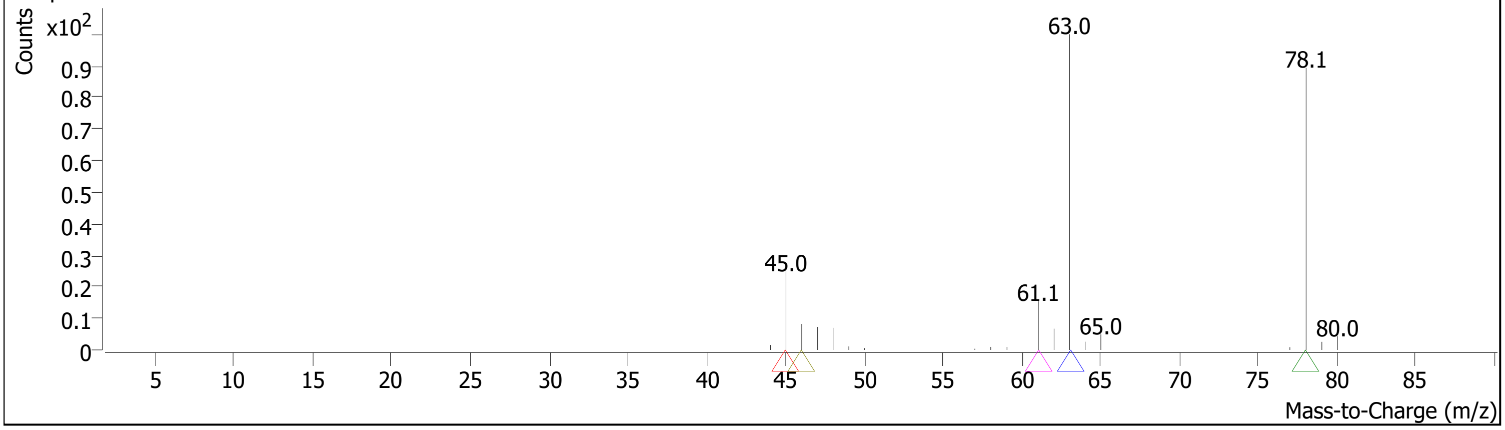


Dimethyl Sulfoxide (W12N20_MAIN.L)

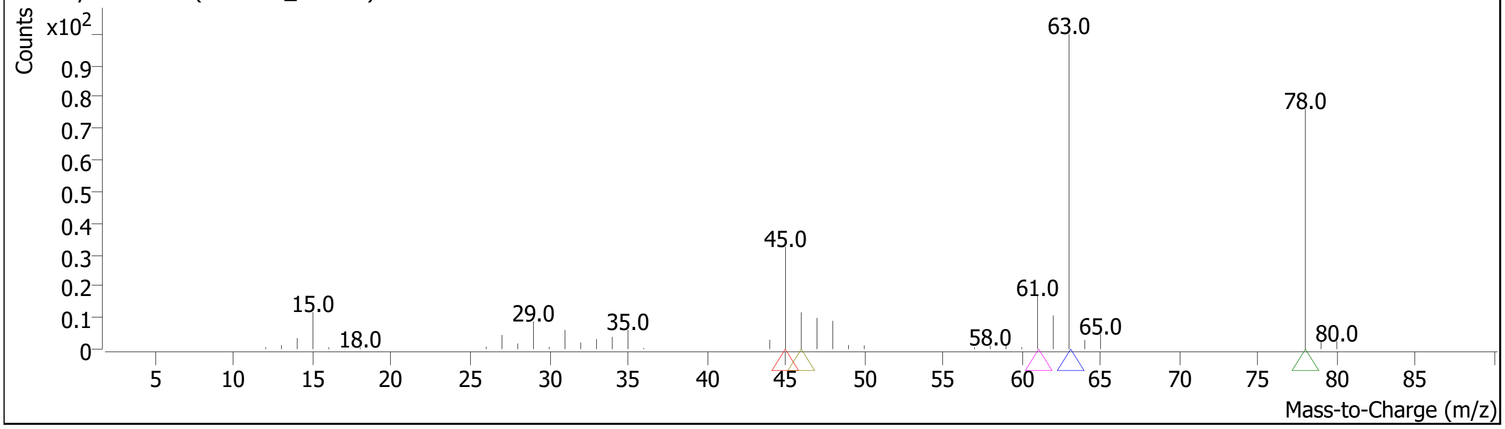


+ Scan (59.6463-59.8802 min, 44 scans) 11795-4.D

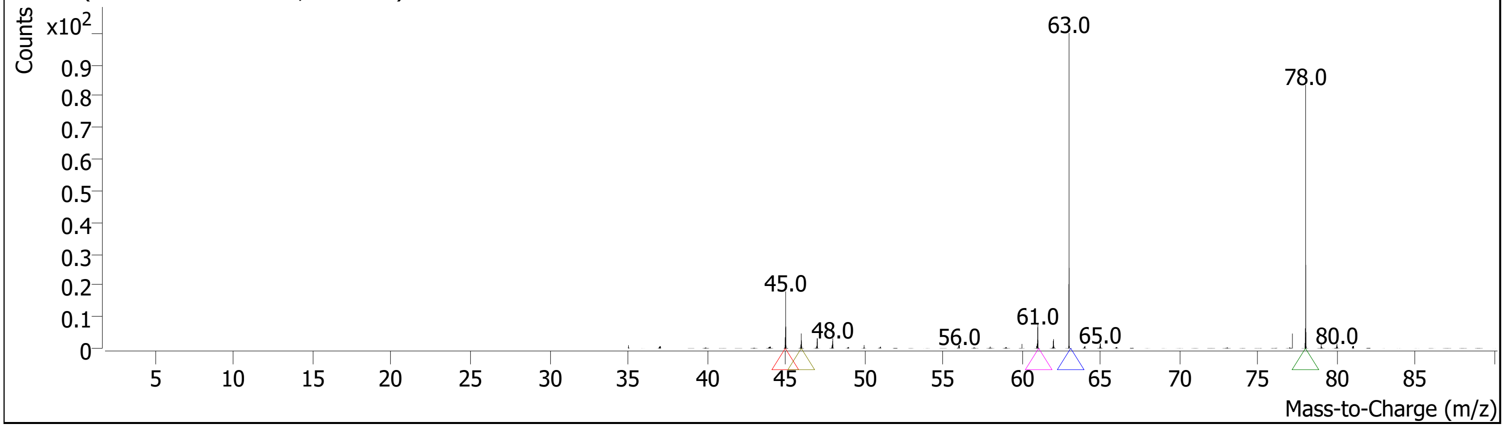


Component RT: 59.7265

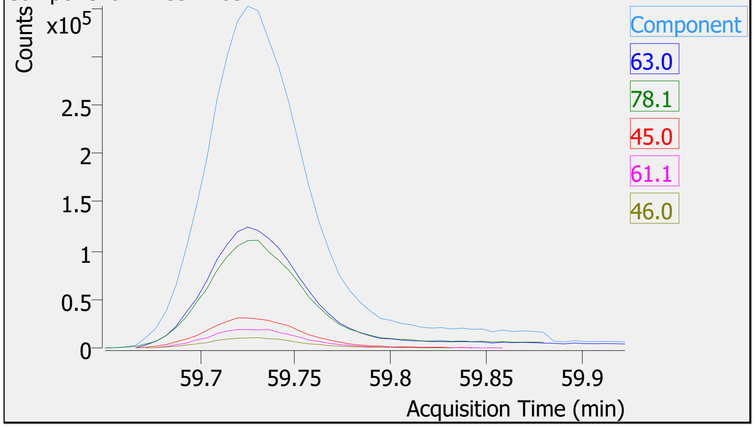


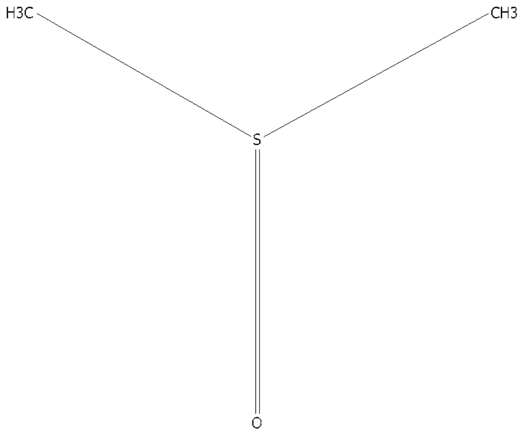

# Unknown Analysis Report - Best Hits

| RT | Compound Name | CAS# | Formula | Area | MI | Match Score | Sample | Sample |
|---|---|---|---|---|---|---|---|---|
| 59.7279 | (2R)-Amino-3-chloropropionic acid hydrochloride | 0-00-0 | C3H7ClNO2 | 614532 | | 87.5 | 0.20 | 0.24 |

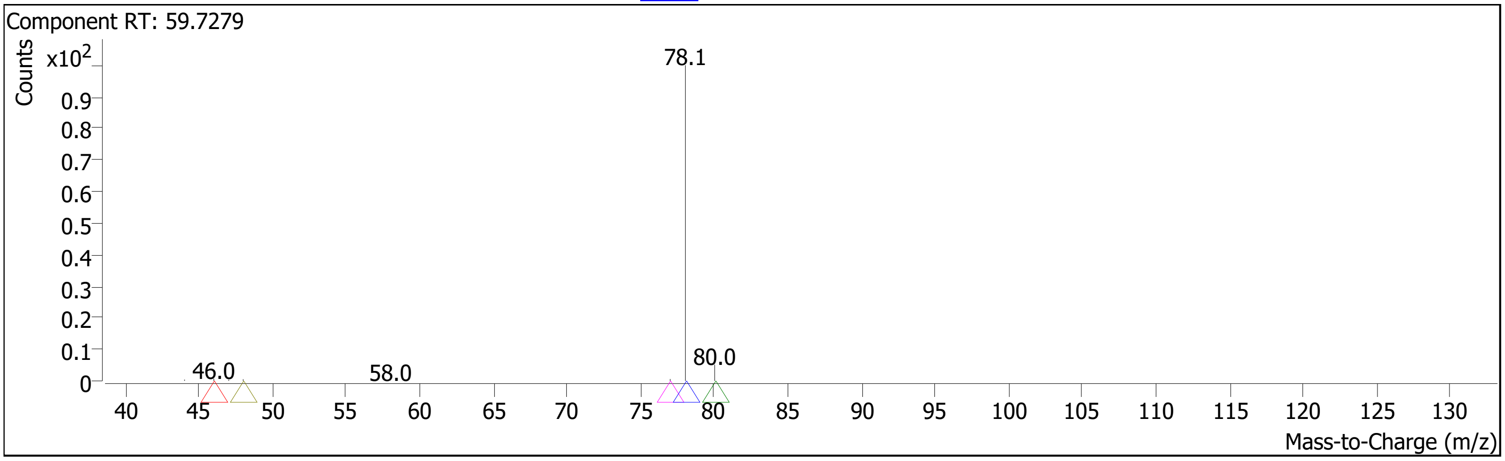


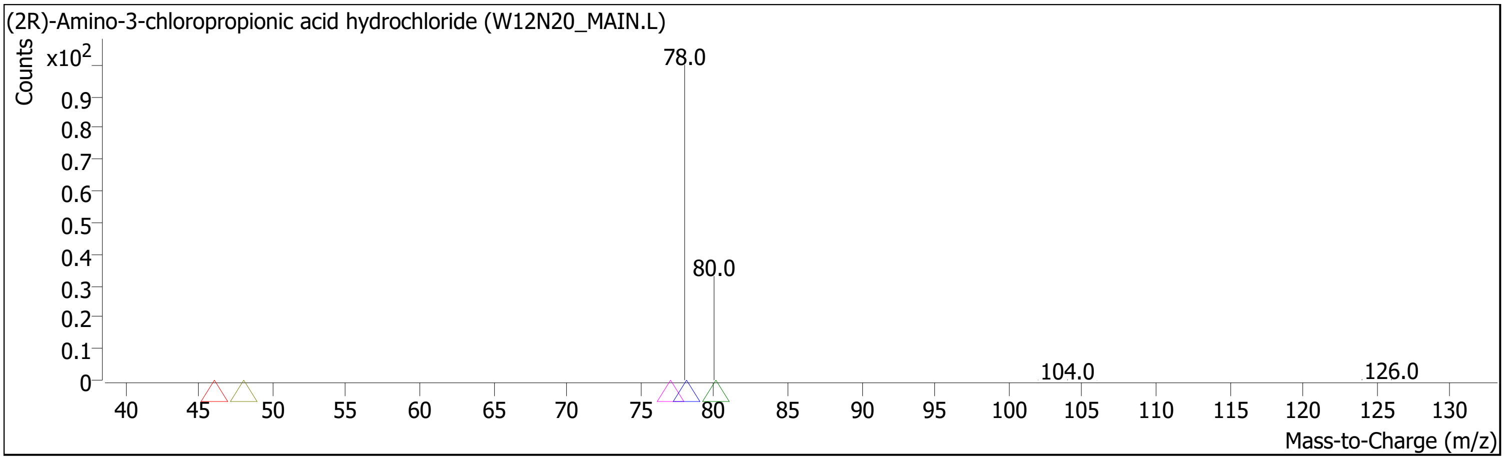


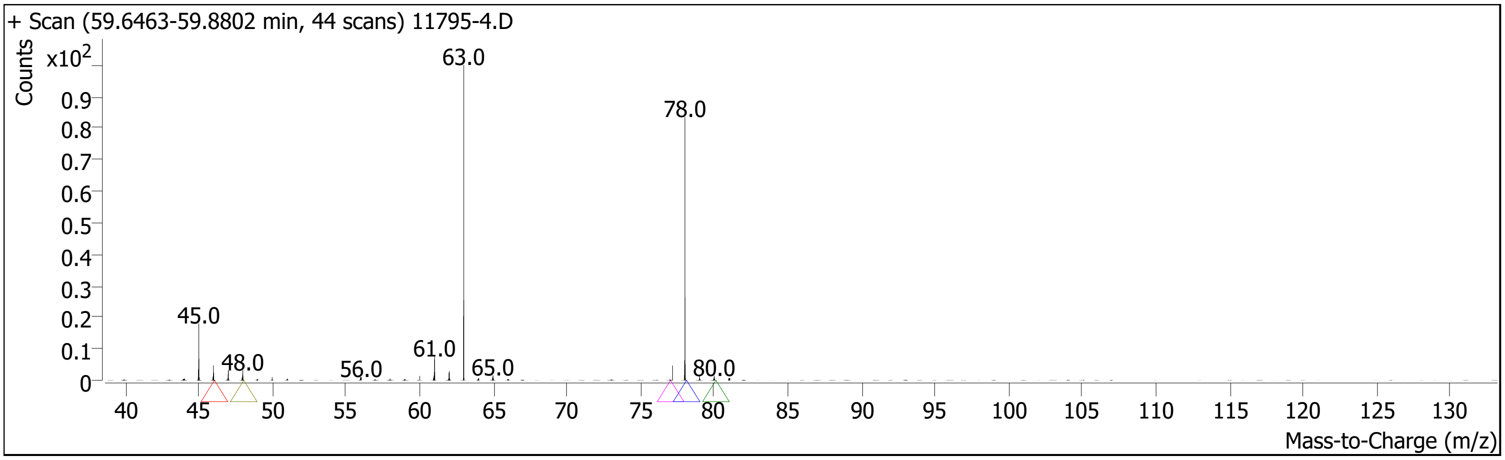


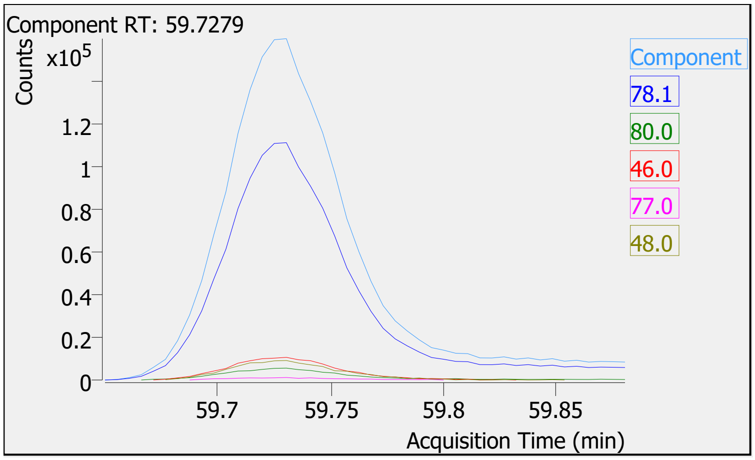


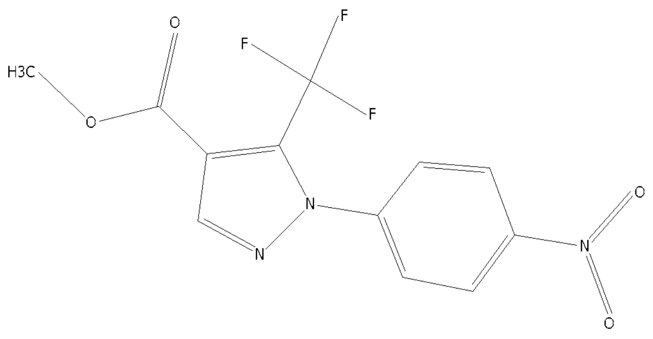

# Unknown Analysis Report - Best Hits

Agilent Trusted Answers

| RT | Compound Name | CAS# | Formula | Area | MI | Match Score | Sample | Sample |
|---|---|---|---|---|---|---|---|---|
| 62.7178 | Dimethyl sulfone | 67-71-0 | C2H6O2S | 142426 | | 88.3 | 0.05 | 0.06 |

Component RT: 62.7178

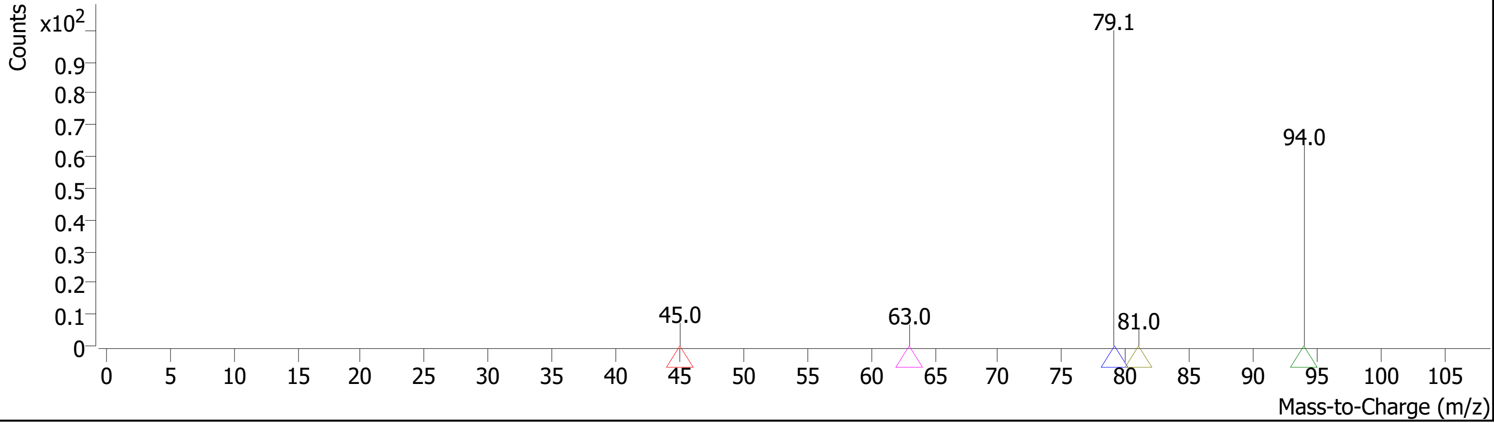


Dimethyl sulfone (W12N20_MAIN.L)

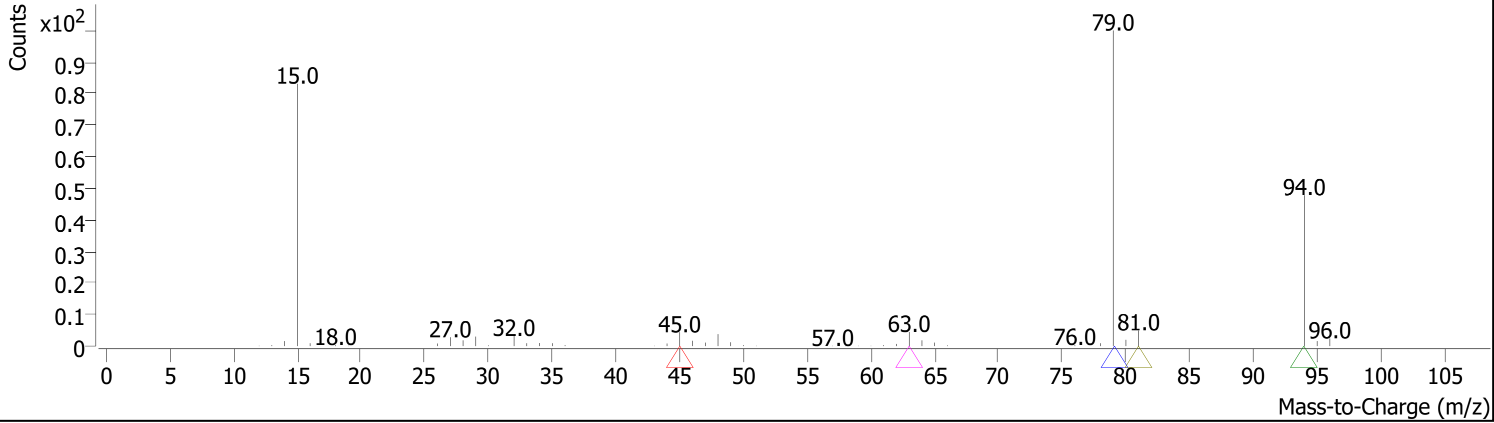


+ Scan (62.6027-62.8541 min, 47 scans) 11795-4.D

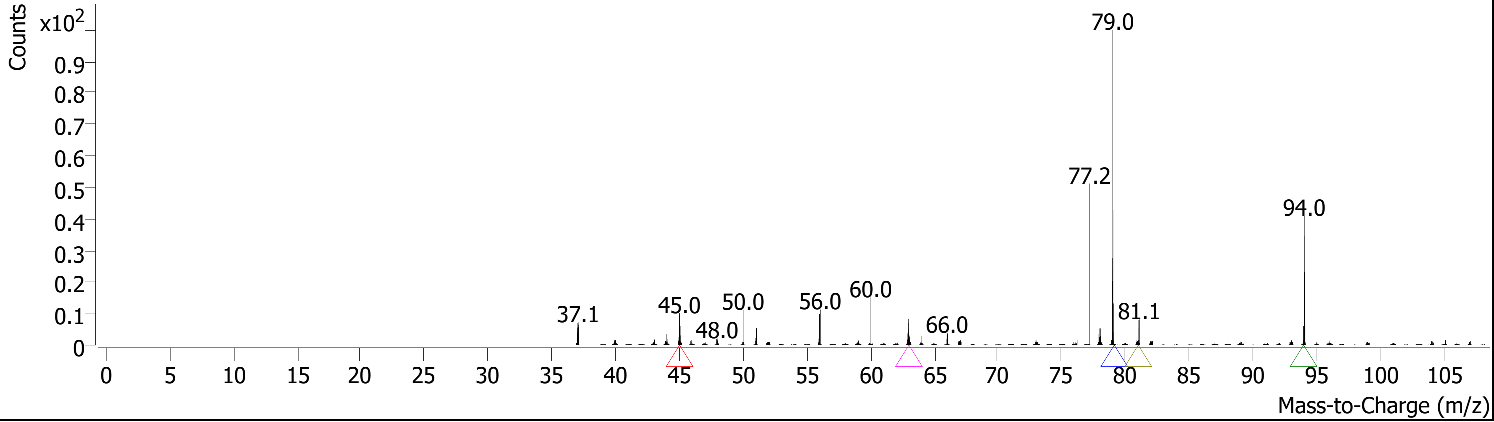


Component RT: 62.7178

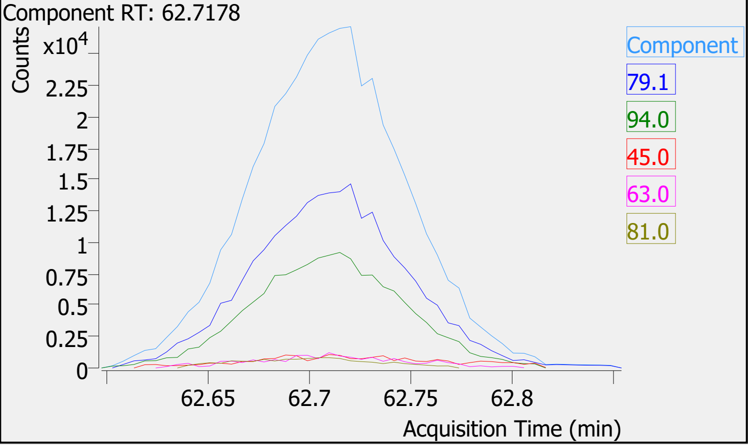


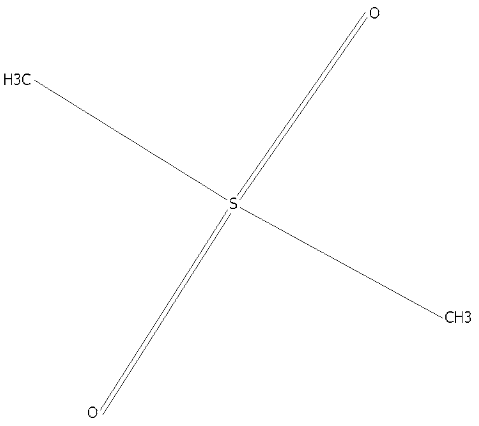

# Unknown Analysis Report - Best Hits

| RT | Compound Name | CAS# | Formula | Area | MI | Match Score | Sample | Sample |
|---|---|---|---|---|---|---|---|---|
| 80.1187 | 3,3-Dideutero-(1R,7aR)-hexahydro-1H-pyrrolizin-1-amine | 990004-39-6 | C7H12D2N2 | 30167 | | 74.2 | 0.01 | 0.01 |

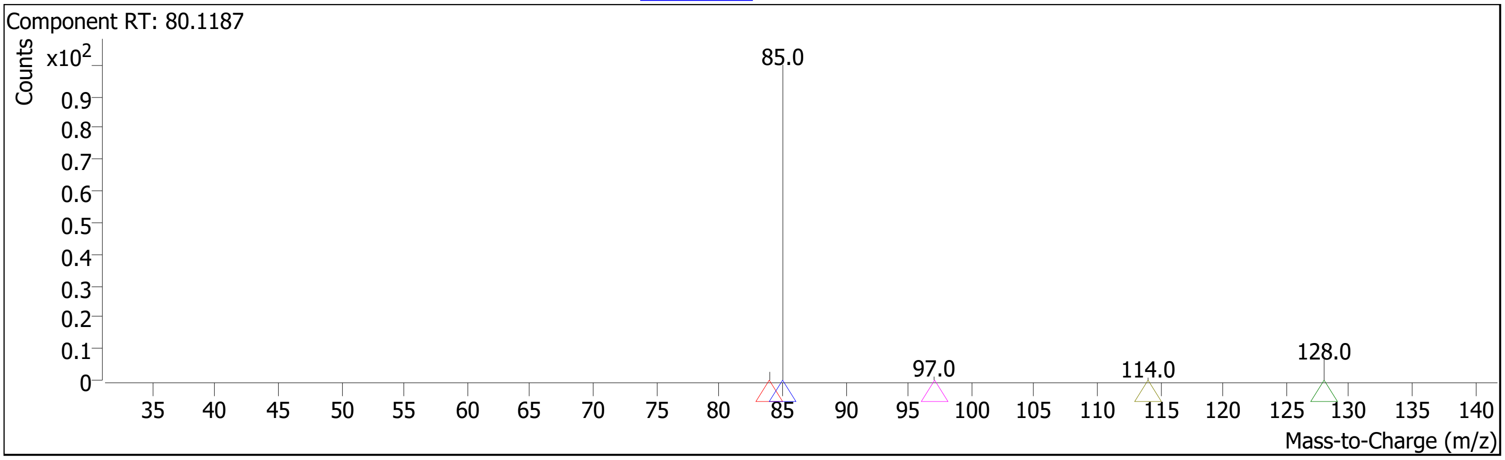


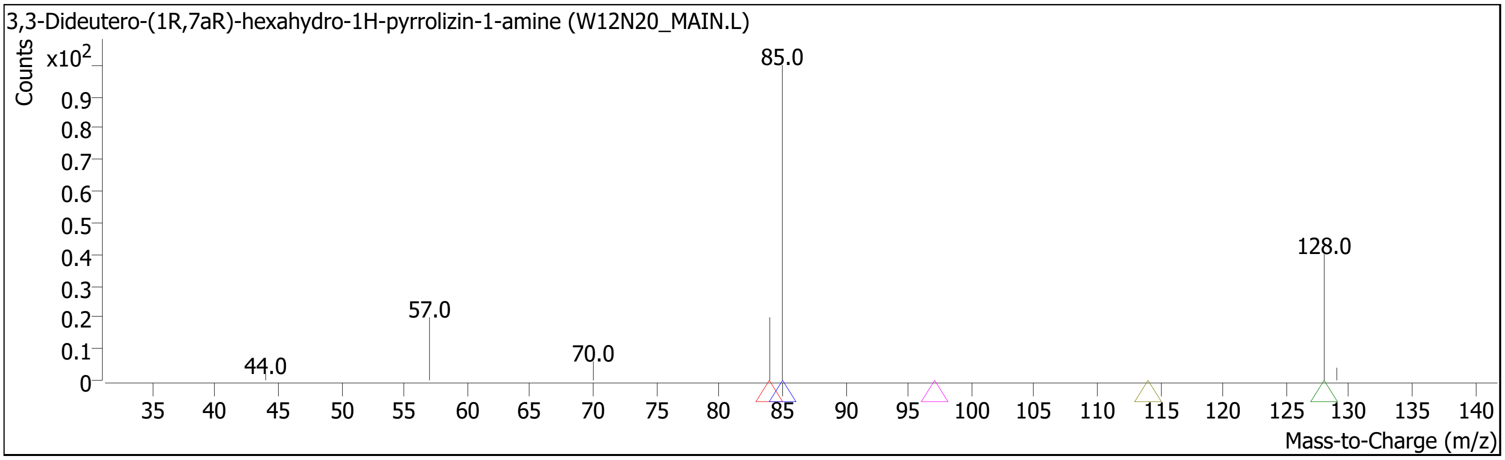


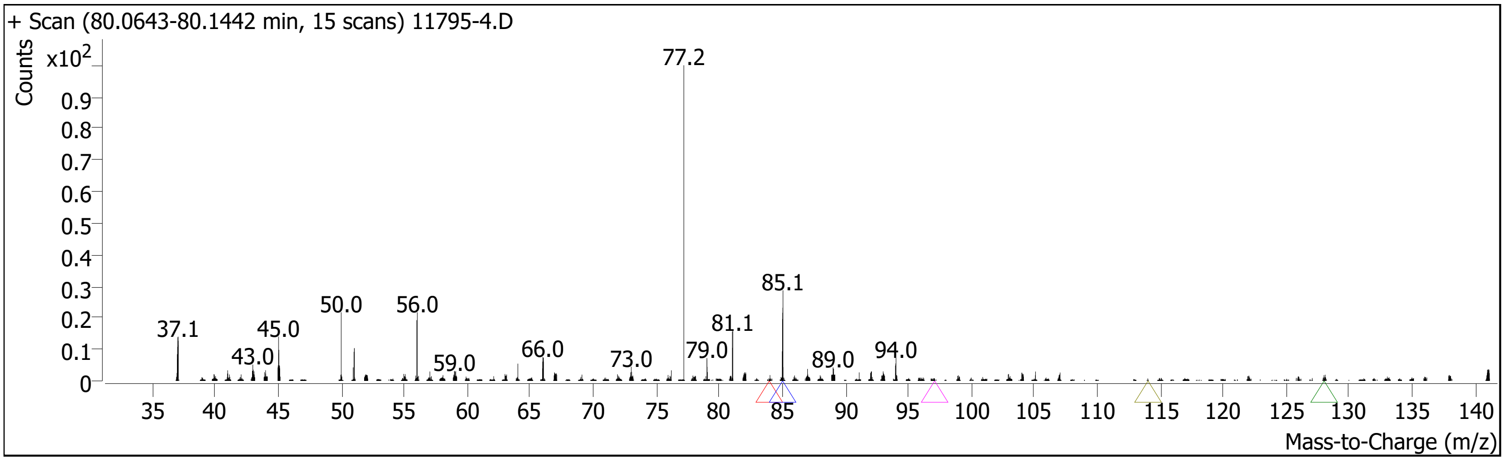


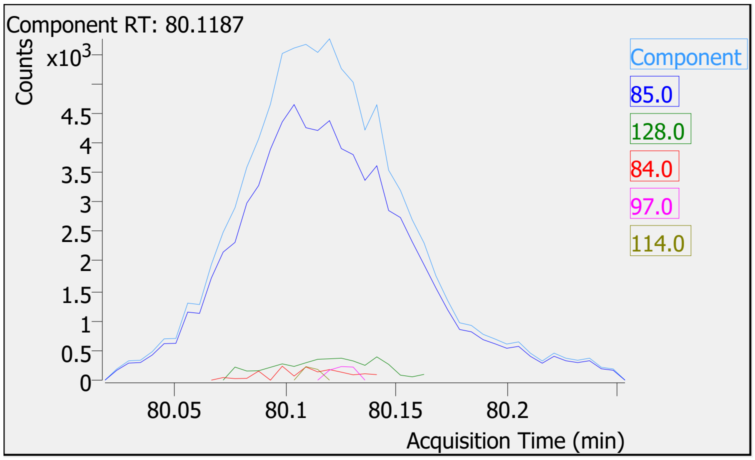


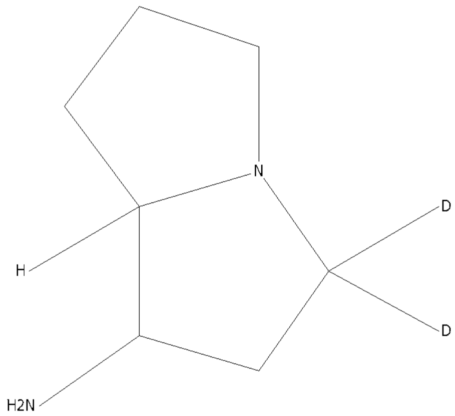

| RT | Compound Name | CAS# | Formula | Area | MI | Match Score | Sample | Sample |
|---|---|---|---|---|---|---|---|---|
| 80.2282 | 5-Acetyl-Longipinandiolone | 990289-11-0 | C17H26O4 | 27314 | | 86.4 | 0.01 | 0.01 |

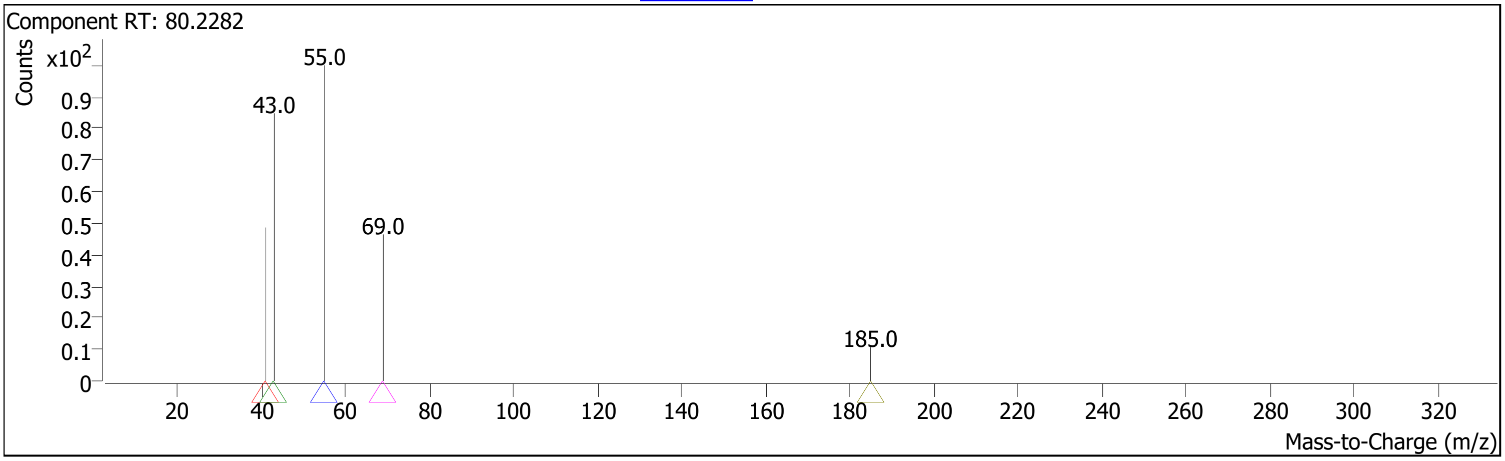


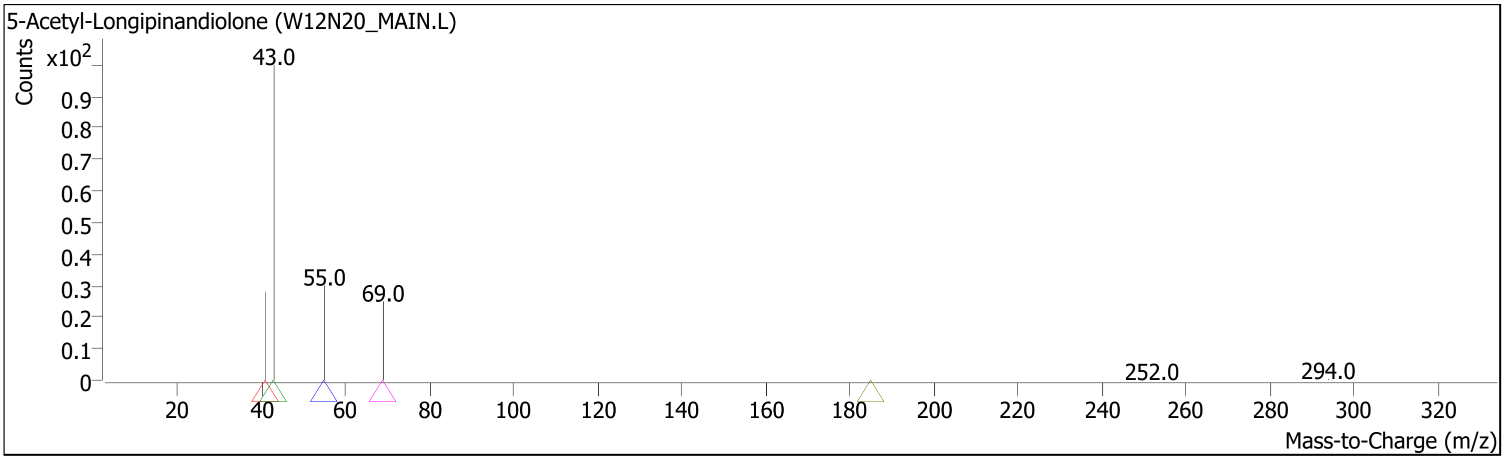


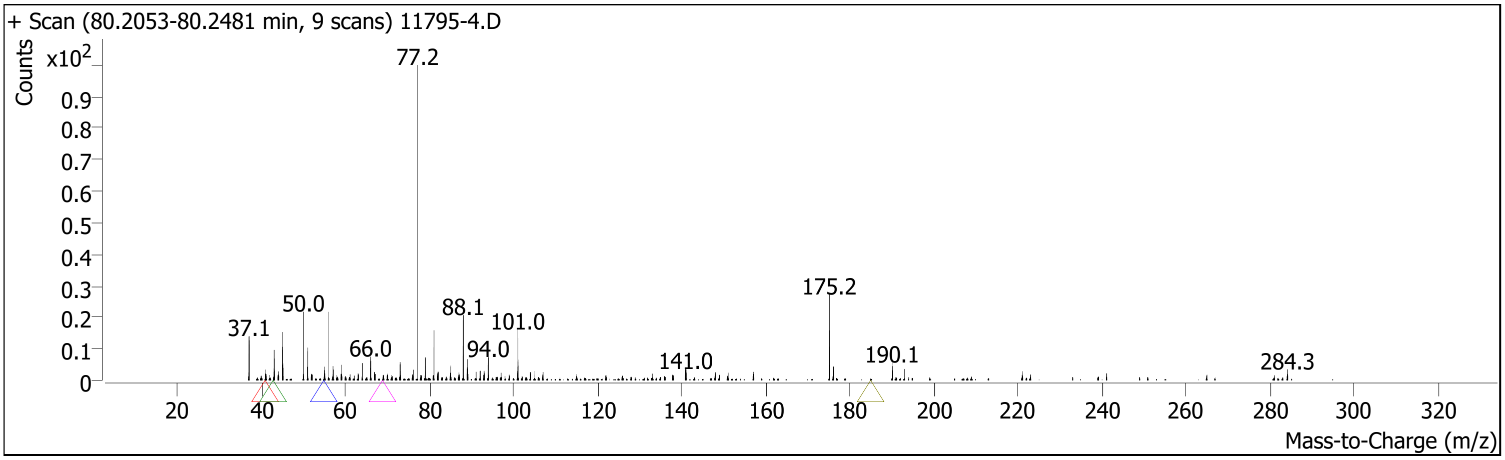


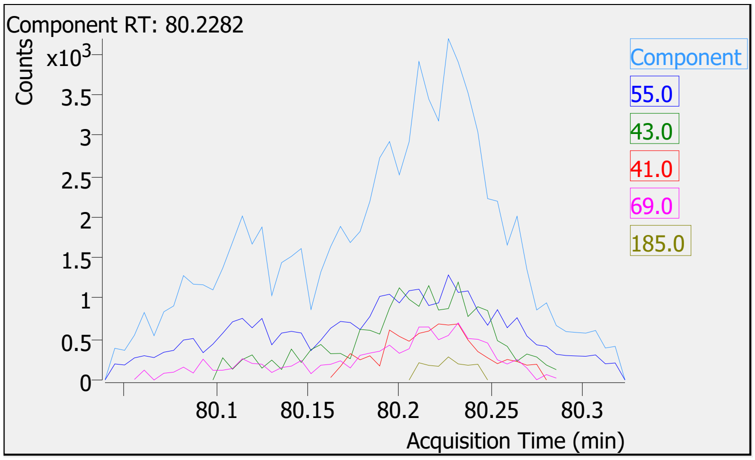


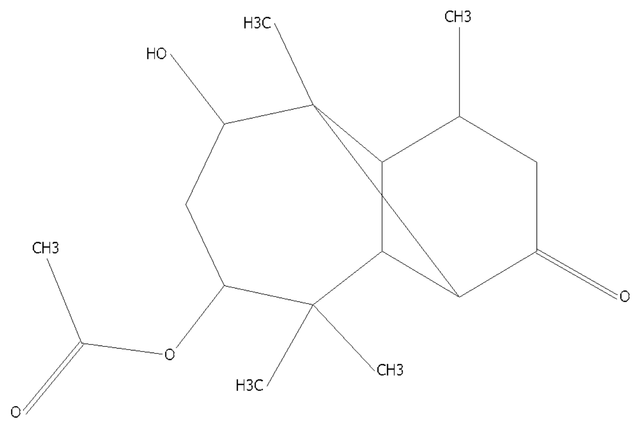




Generated at 4:37 PM on 11/14/2023

| RT | Compound Name | CAS# | Formula | Area | MI | Match Score | Sample | Sample |
|---|---|---|---|---|---|---|---|---|
| 82.5411 | Pentanoic acid, 5-hydroxy-, 2,4-di-t-butylphenyl esters | 166273-38-7 | C19H30O3 | 82810 | | 78.3 | 0.03 | 0.03 |

Component RT: 82.5411

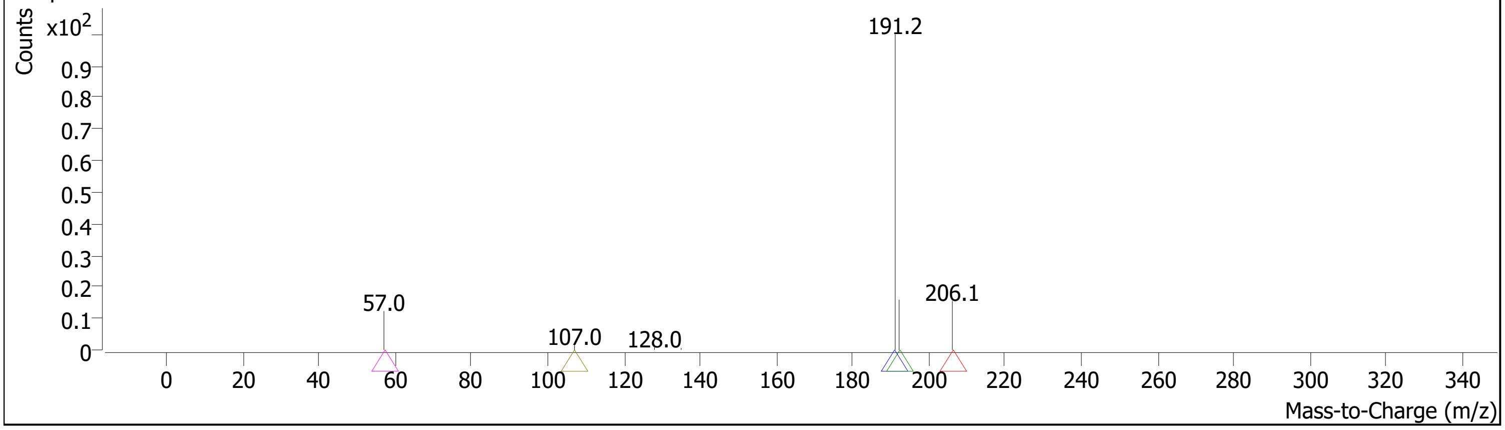


Pentanoic acid, 5-hydroxy-, 2,4-di-t-butylphenyl esters (W12N20_MAIN.L)

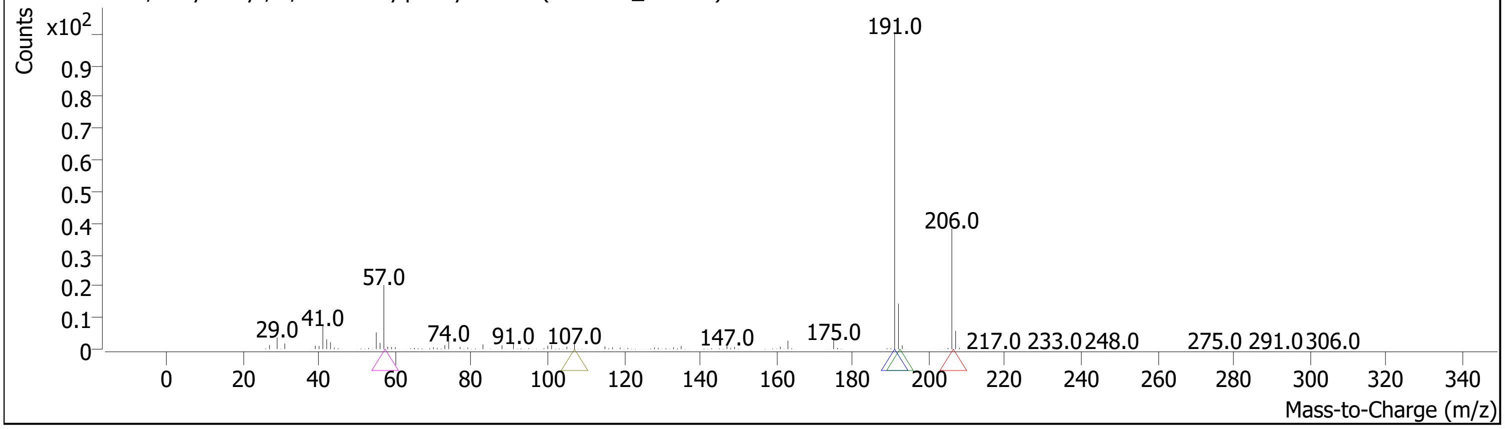


+ Scan (82.4624-82.6068 min, 28 scans) 11795-4.D

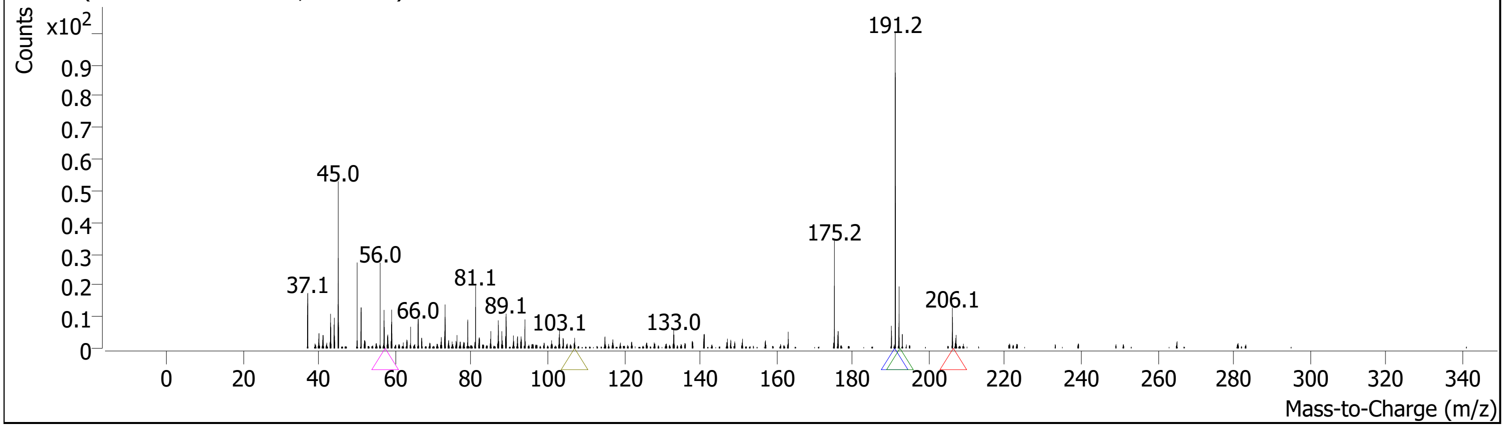


Component RT: 82.5411

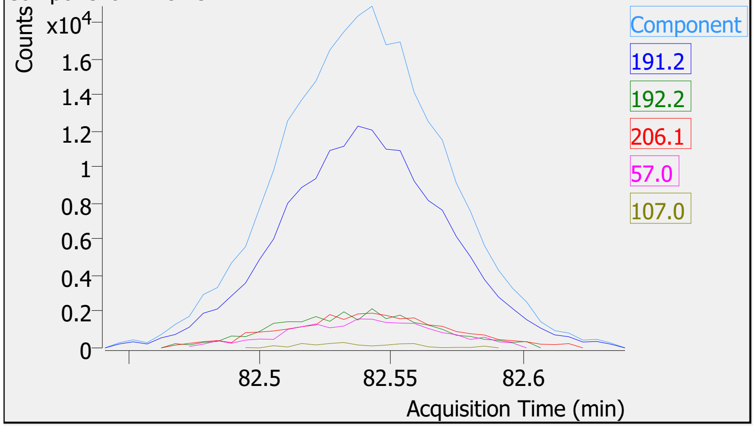


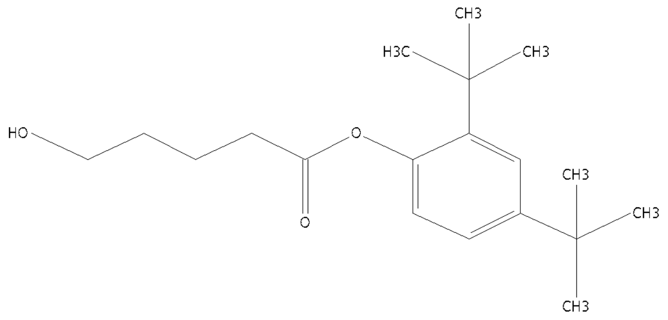


 Generated at 4:37 PM on 11/14/2023

# Unknown Analysis Report - Best Hits

| RT | Compound Name | CAS# | Formula | Area | MI | Match Score | Sample | Sample |
|---|---|---|---|---|---|---|---|---|
| 102.2049 | 2-Methoxy-6-methyl-9,10-dihydro-9,10-ethanoanthracene-11,12-dicarboxylic acid | 990397-11-4 | C20H18O5 | 12725 | | 77.6 | 0.00 | 0.01 |

Component RT: 102.2049

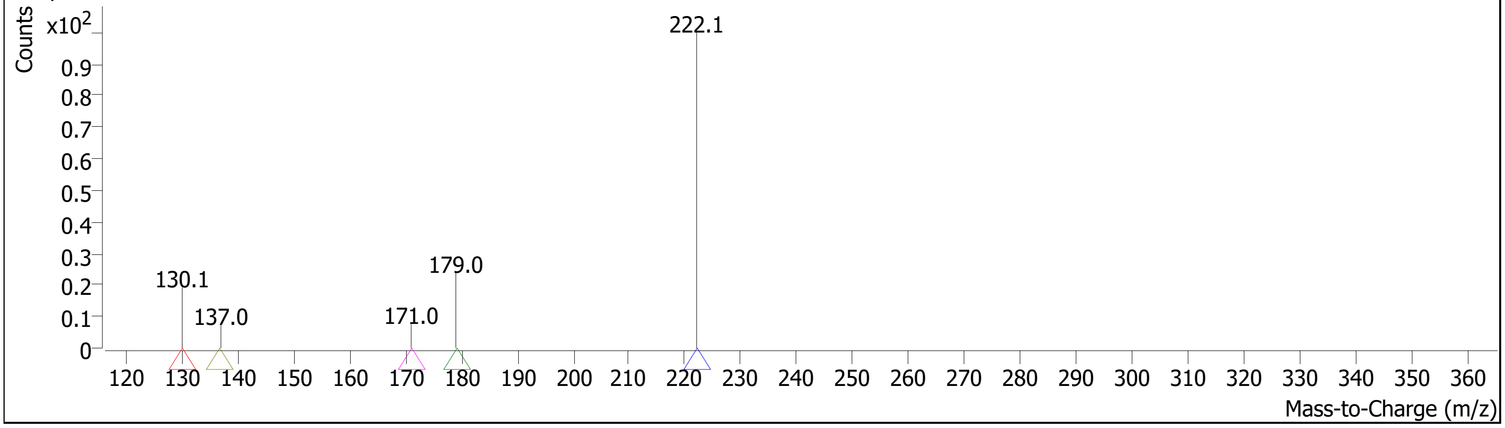


2-Methoxy-6-methyl-9,10-dihydro-9,10-ethanoanthracene-11,12-dicarboxylic acid (W12N20_MAIN.L)

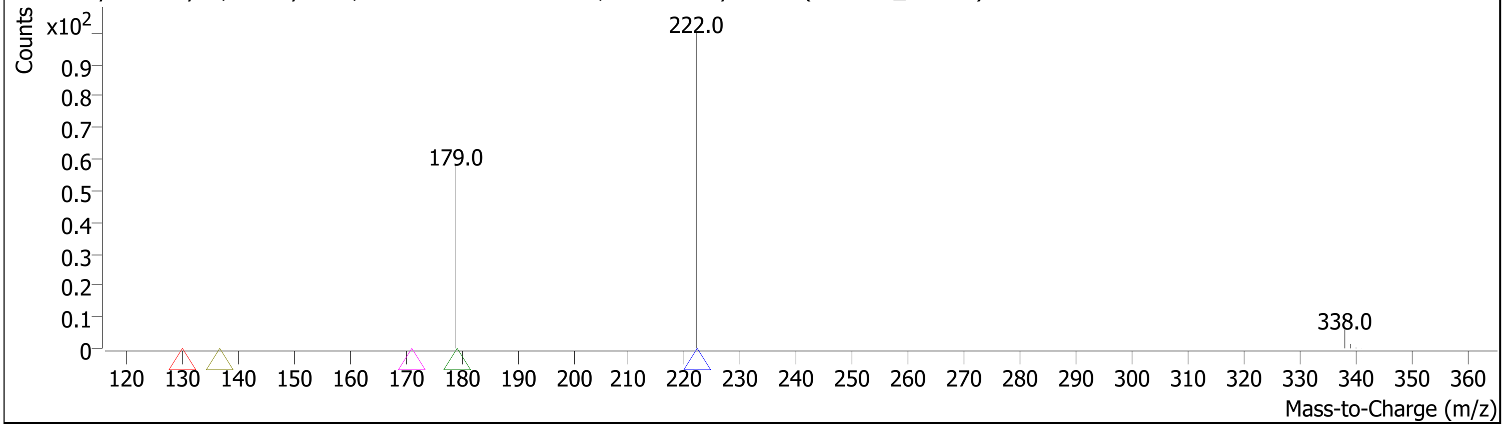


+ Scan (101.9531-102.3382 min, 72 scans) 11795-4.D

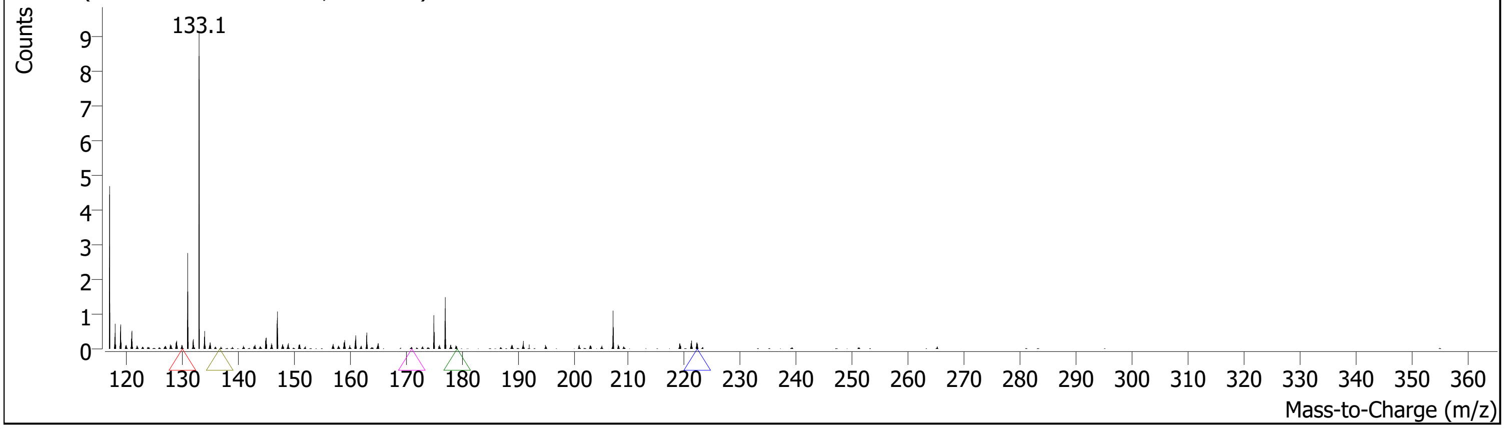


Component RT: 102.2049

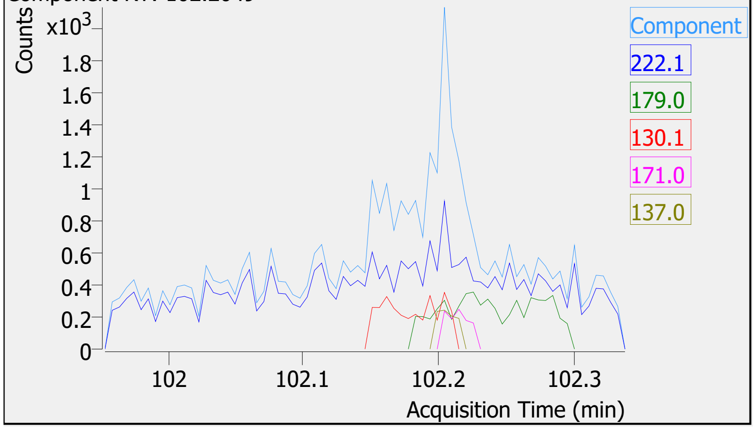


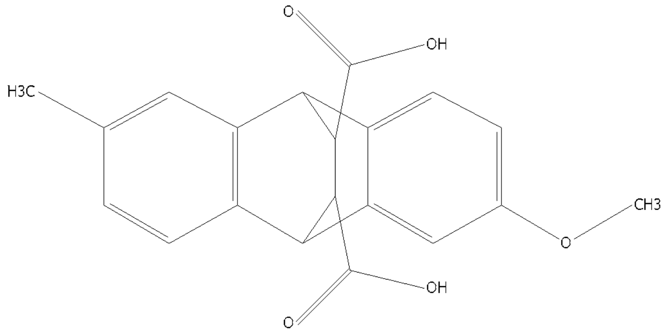


 Generated at 4:37 PM on 11/14/2023
