# Supplementary material for: Lactiplantibacillus plantarum K8 lysates regulate hypoxia-induced gene expression
Source: Sci Rep. 2024 Mar 15;14:6275. doi: 10.1038/s41598-024-56958-7 (PMC10943017; doi:10.1038/s41598-024-56958-7)

Unknown Analysis Report - Best Hits

**Batch Path**	D:\MassHunter\GCMS\1\data\2023\2023-10		
**Analysis File Name**	11795.uaf		
**Analyst Name**	admin		
**Analysis Time**	11/14/2023 4:19:03 PM		
**Data File Name**	11795-3.D	**Data Path Name**	D:\MassHunter\GCMS\1\data\2023\2023-10
**Sample Name**	K8 PBS	**Sample Type**	Sample
**Acq Method File**	DB-WAX	**Acq Method Path**	D:\MassHunter\GCMS\1\methods\
**Acq Time**	11/13/2023 9:52:09 PM	**Operator**	
**Instrument Name**	GCMS	**Dilution**	1

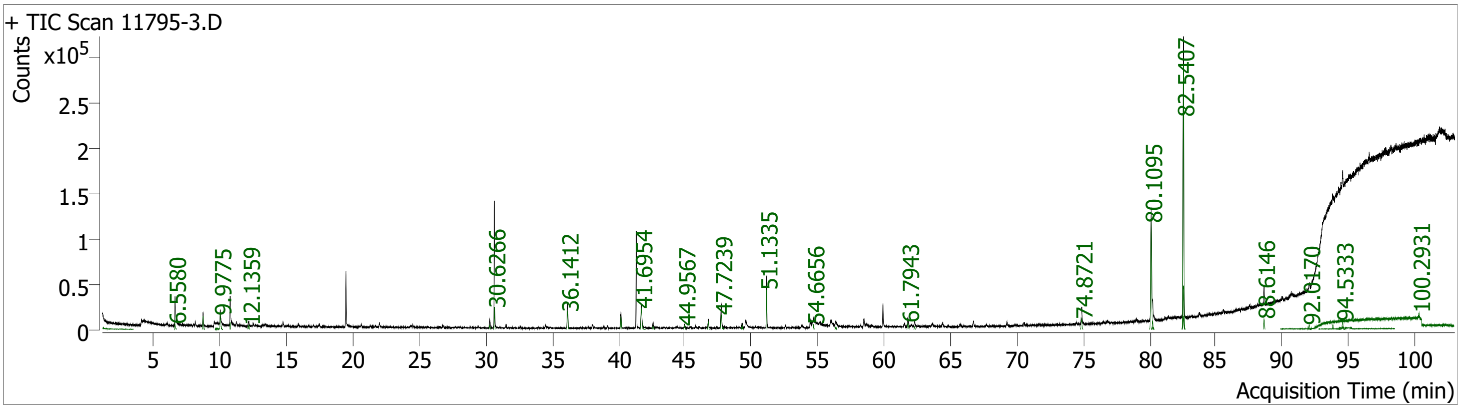

RT	Compound Name	CAS#	Formula	Area	MI	Match Score	Sample	Sample
1.1586	2-(1',3'-Dioxolan-2'-yl)-3-phenyldecan-4-one	0-00-0	C18H26O3	72651		82.3	0.76	1.25
6.5580	4-(p-Methylaminoanilino)pyridine-2-carbonitrile	990119-48-3	C13H12N4	22936		86.9	0.24	0.40
8.6730	Dimethylamine-D1	917-72-6	C2H6DN	53900		93.5	0.57	0.93
9.9775	Acetic acid ethenyl ester	108-05-4	C4H6O2	81026		94.7	0.85	1.40
10.7201	(1R,4R,5R)-4-fluoranyl-1-methyl-5-[(4-methylphenyl)sulfanylmethyl]-7,8-dioxabicyclo[3.2.1]octane	990257-95-4	C15H19FO2S	10776		78.8	0.11	0.19
12.1359	1,1-Dimethoxy-3-(4-nitrophenyl)propan-2-one	990152-35-6	C11H13NO5	20304		82.5	0.21	0.35
30.2842	5-Methylene-9-decen-2-one	990027-19-1	C11H18O	17948		78.1	0.19	0.31
30.6266	1-Ethyl-2-(4'-fluorophenyl)-4,5-diphenyl-imidazole	990407-68-5	C23H19FN2	60271		72.2	0.63	1.04
36.1412	1,2-Di-tert-butylbenzene	1012-76-6	C14H22	84197		85.3	0.88	1.45
40.1620	1-Hexanol, 2-ethyl-	104-76-7	C8H18O	58324		82.9	0.61	1.01
41.6954	(anti/syn)-2-Nitro-1-phenylpropan-1,3-diol	990067-30-9	C9H11NO4	130696		98.7	1.37	2.26
42.5829	6-(2-Aminophenyl)-1-methylpyrido[2,3-d]pyrimidine-2,4(1H,3H)-dione	990222-52-0	C14H12N4O2	17779		72.2	0.19	0.31
44.9567	Methyl 5-endo-4-hydroxy-2,5,7,7-tetramethylbicyclo[2.2.2]oct-2-en-5-carboxylate	990150-69-0	C14H22O3	18682		86.1	0.20	0.32
46.7473	2,2-Dimethylpropanoic acid tert-butyl ester	16474-43-4	C9H18O2	31099		84.4	0.33	0.54
47.7239	(2R,3S)-2-hydroxy-1-phenyl-3-vinyl-hexan-1-one	990106-41-5	C14H18O2	92247		91.3	0.97	1.59
49.2887	(2S,3S)-3-Methyl-2-(1-phenylethyl)-3-(trifluoromethyl)-1,2-oxaziridine	990133-36-1	C11H12F3NO	15004		91.9	0.16	0.26
51.1335	Cyclooctasiloxane, hexadecamethyl-	556-68-3	C16H48O8Si8	102252		72.7	1.07	1.77
54.6656	Dimethyl ether	115-10-6	C2H6O	13474		87.9	0.14	0.23
56.3621	2-Hexoxybenzoic acid methyl ester	56306-81-1	C14H20O3	14205		71.7	0.15	0.25
61.7943	2,2-bis(fluoranyl)-1-phenyl-but-3-en-1-ol	85864-61-5	C10H10F2O	38511		89.3	0.40	0.66
62.2943	(S)-[1-(1-Allyloxyethyl)-1-vinylalloxymethyl]benzene	990198-79-9	C17H22O2	10490		71.3	0.11	0.18
74.8721	trans-3,4-Dimethyl-2,3-epoxypentanal	990005-04-8	C7H12O2	54515		82.5	0.57	0.94
80.1095	2(3H)-Furanone, 5-heptyldihydro-	104-67-6	C11H20O2	578167		89.7	6.08	9.98
80.2103	3-Acetyloxypropyl 2,3,4,6-tetra-O-methyl-.alpha.,L-(5-D)gulopyranoside	990391-50-2	C15H27DO8	41421		70.3	0.44	0.72
82.5324	Methyl 5-[2-(4-Bromophenyl)-2-chloroethyl]-4,6-dimethylsalicylate	990507-75-8	C18H18BrClO3	60098		70.2	0.63	1.04
82.5379	6,7-Dimethoxy-1-[N-(1-phenylethyl)amido]-1,2,3,4-tetrahydroisoquinoline isomer	990402-02-5	C20H24N2O3	217579		74.5	2.29	3.76

RT	Compound Name	CAS#	Formula	Area	MI	Match Score	Sample	Sample
82.5407	Phenol, 2,4-bis(1,1-dimethylethyl)-	96-76-4	C14H22O	1317730		91.8	13.85	22.75
88.6146	1,2,4-Trimethoxy-5-[(1Z)-1-propenyl]benzene	5273-86-9	C12H16O3	50156		78.7	0.53	0.87
92.0170	1-(Benzyloxy)-2-fluoro-2-phenyl-3-(p-toluenesulsulfonyloxy)propane	990534-16-2	C23H23FO4S	26067		83.8	0.27	0.45
93.7940	3-Methyl-1,1-diphenyl-urea	990124-21-3	C14H14N2O	20182		89.0	0.21	0.35
94.5268	1-Phenyl-3-pentyn-1-ol	990021-78-0	C11H12O	22147		87.1	0.23	0.38
94.5333	2-(3-Pyridyl)-3-(4-toluenesulfonamido)propylazetidine	62247-30-7	C15H16N2O2S	368892		92.6	3.88	6.37
100.2931	2-Oxo-4-phenylbut-3-enyl oct-2-ynoate	990264-51-1	C18H20O3	5791896		85.6	60.87	100.00

Unknown Analysis Report - Best Hits

RT	Compound Name	CAS#	Formula	Area	MI	Match Score	Sample	Sample
1.1586	2-(1',3'-Dioxolan-2'-yl)-3-phenyldecan-4-one	0-00-0	C18H26O3	72651		82.3	0.76	1.25

Component RT: 1.1586

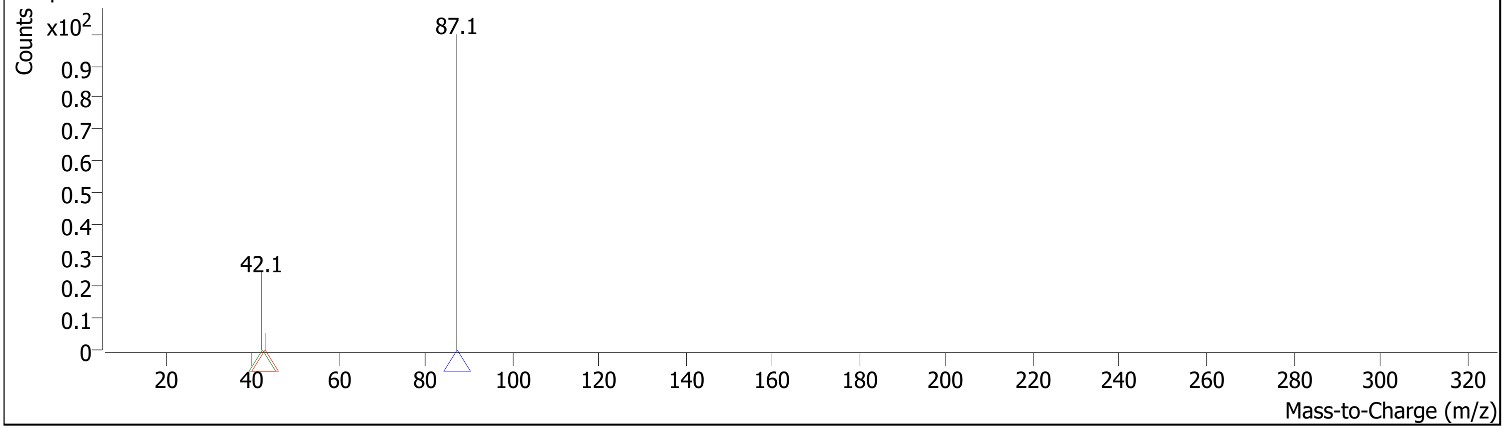

2-(1',3'-Dioxolan-2'-yl)-3-phenyldecan-4-one (W12N20_MAIN.L)

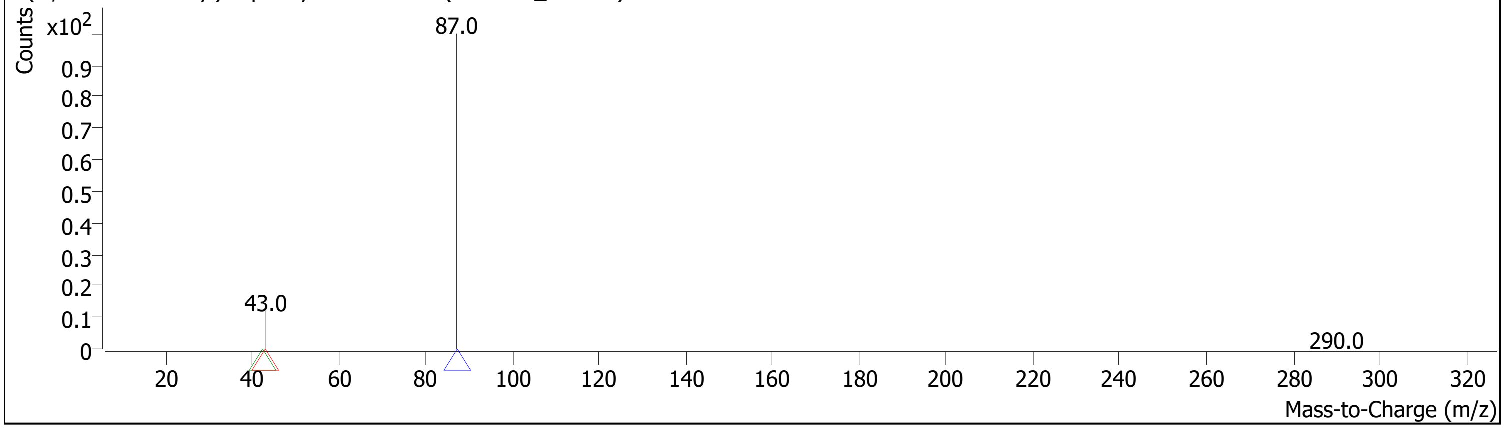

+ Scan (1.1245-1.4454 min, 61 scans) 11795-3.D

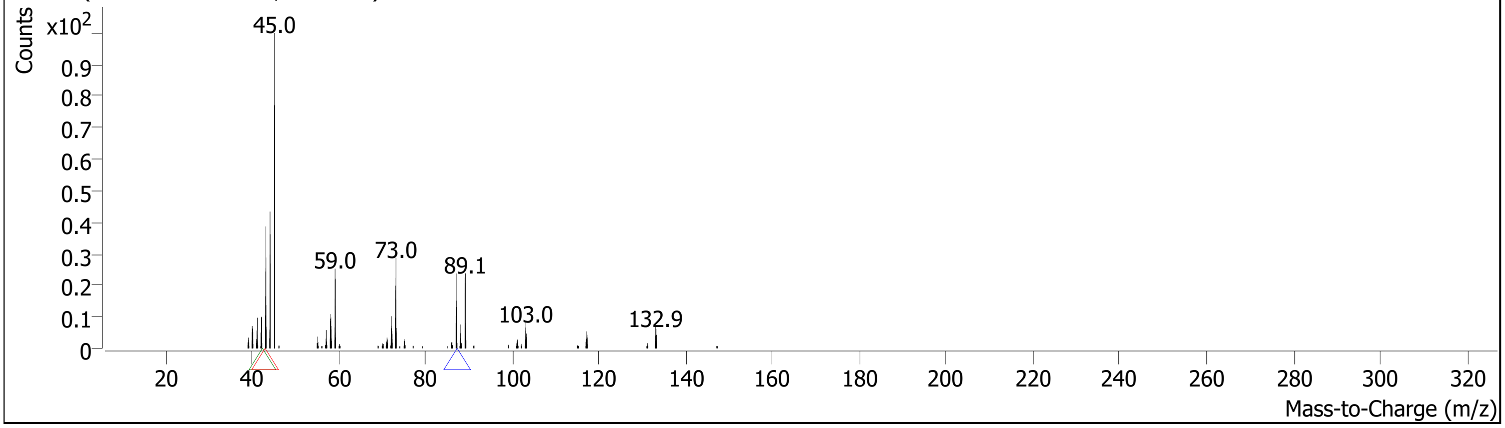

Component RT: 1.1586

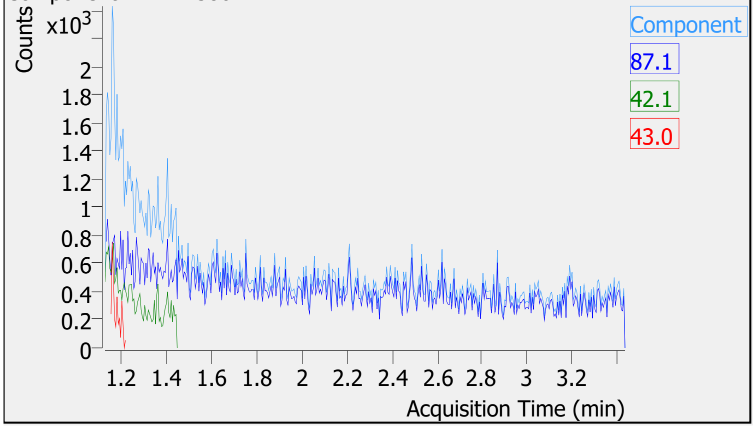

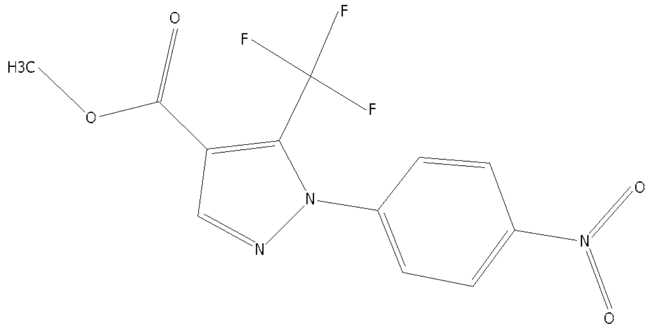

Unknown Analysis Report - Best Hits

RT	Compound Name	CAS#	Formula	Area	MI	Match Score	Sample	Sample
6.5580	4-(p-Methylaminoanilino)pyridine-2-carbonitrile	990119-48-3	C13H12N4	22936		86.9	0.24	0.40

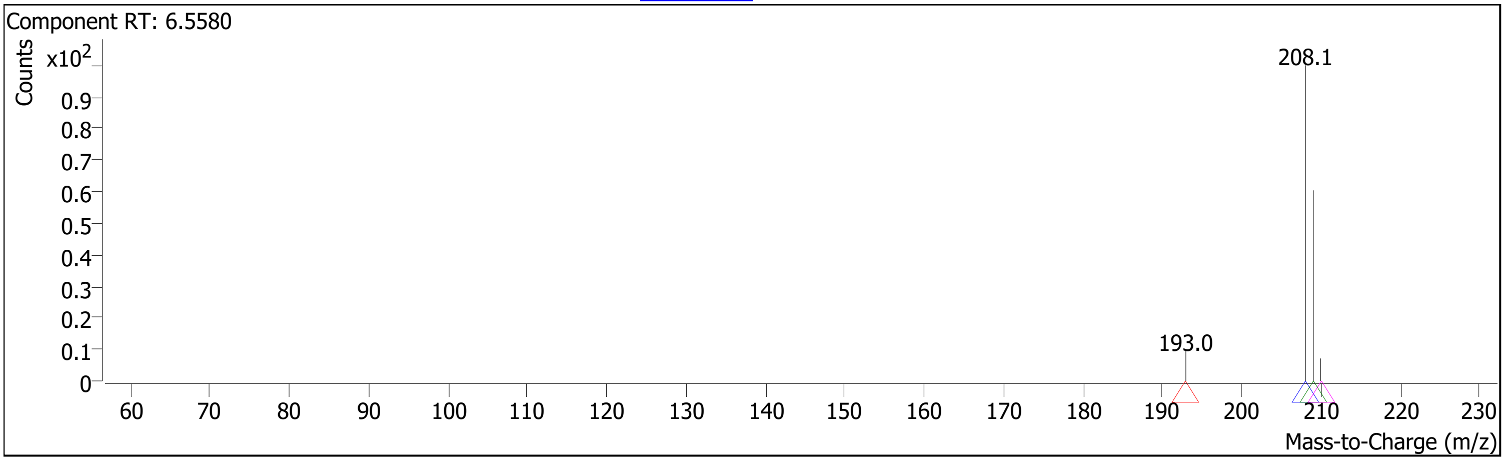

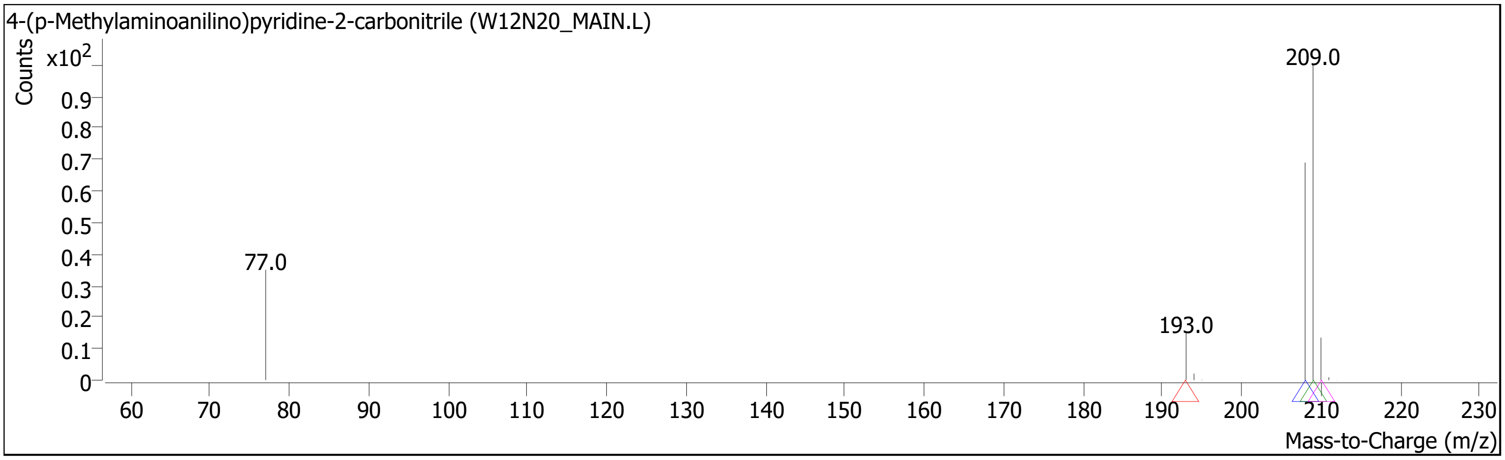

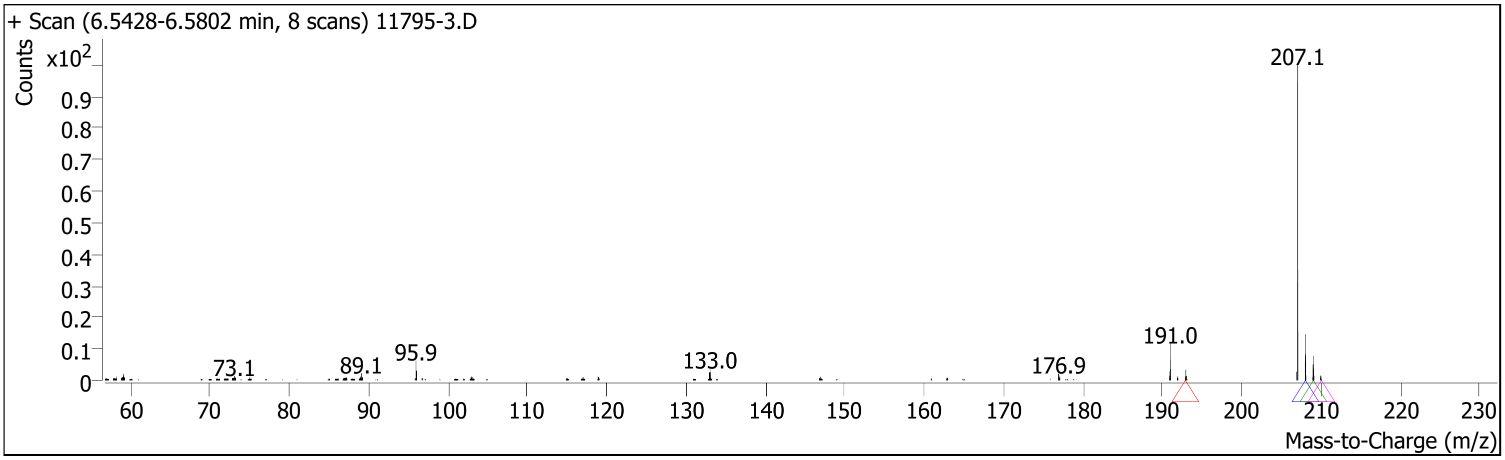

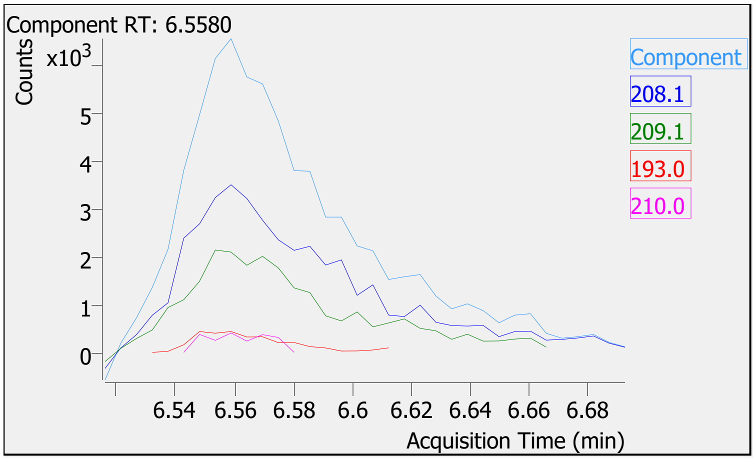

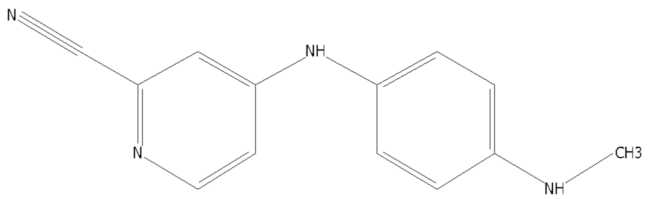

Unknown Analysis Report - Best Hits

RT	Compound Name	CAS#	Formula	Area	MI	Match Score	Sample	Sample
8.6730	Dimethylamine-D1	917-72-6	C2H6DN	53900		93.5	0.57	0.93

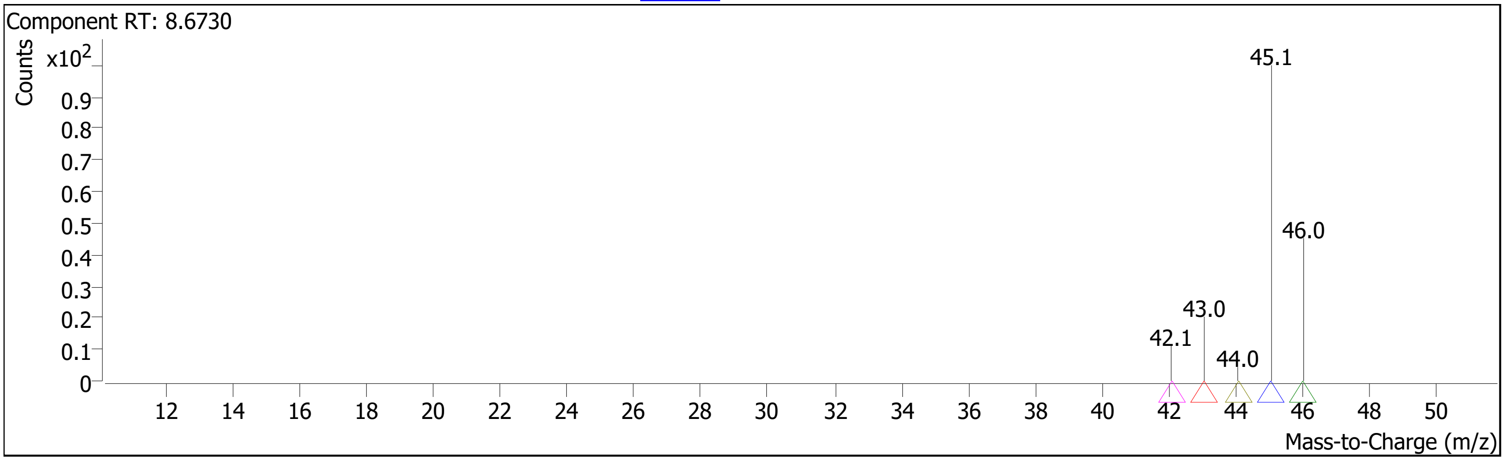

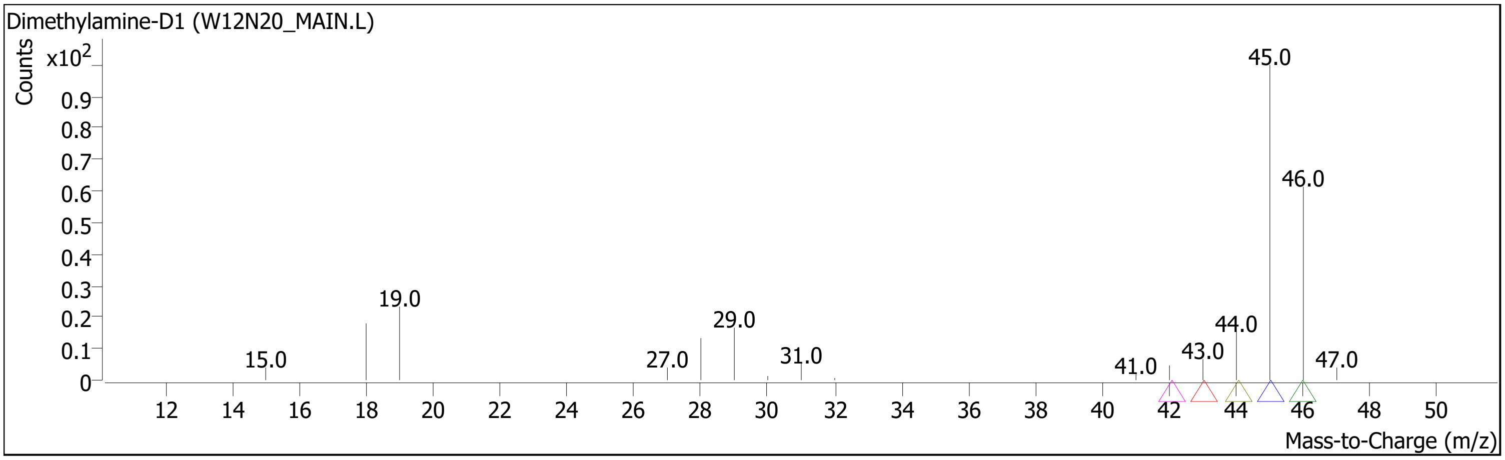

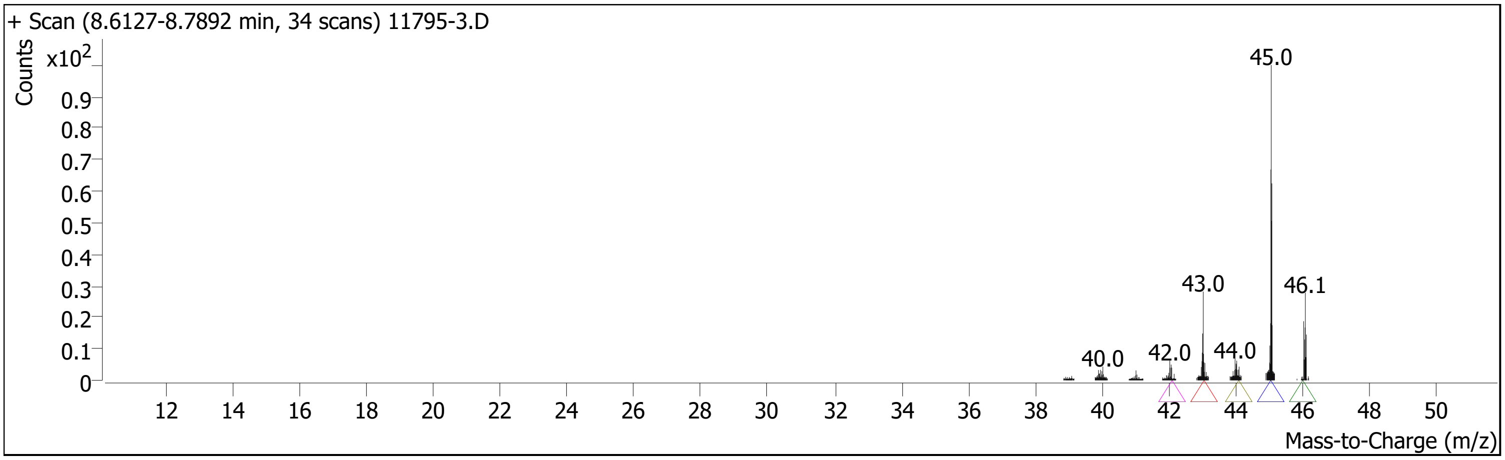

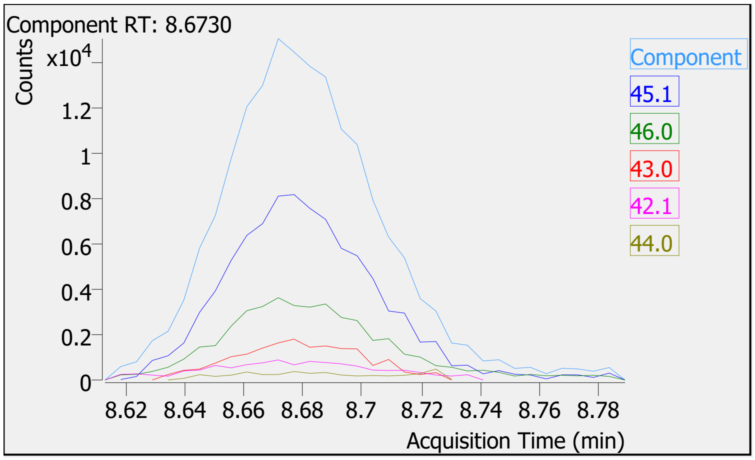

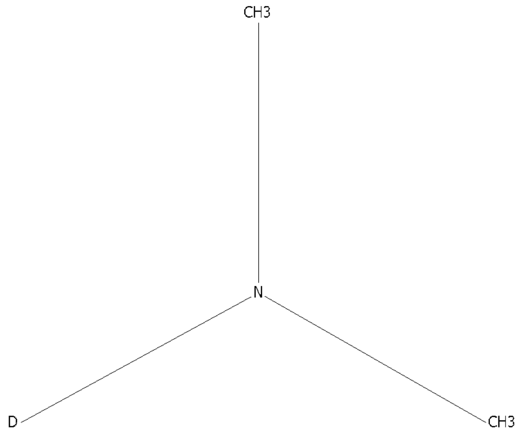

Unknown Analysis Report - Best Hits

RT	Compound Name	CAS#	Formula	Area	MI	Match Score	Sample	Sample
9.9775	Acetic acid ethenyl ester	108-05-4	C4H6O2	81026		94.7	0.85	1.40

Component RT: 9.9775

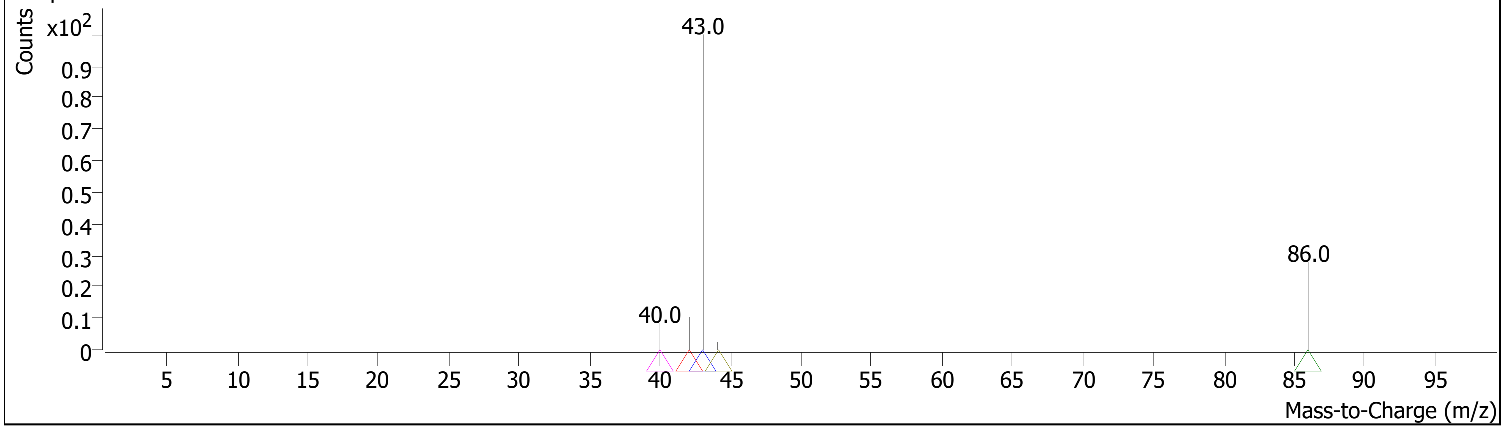

Acetic acid ethenyl ester (W12N20_MAIN.L)

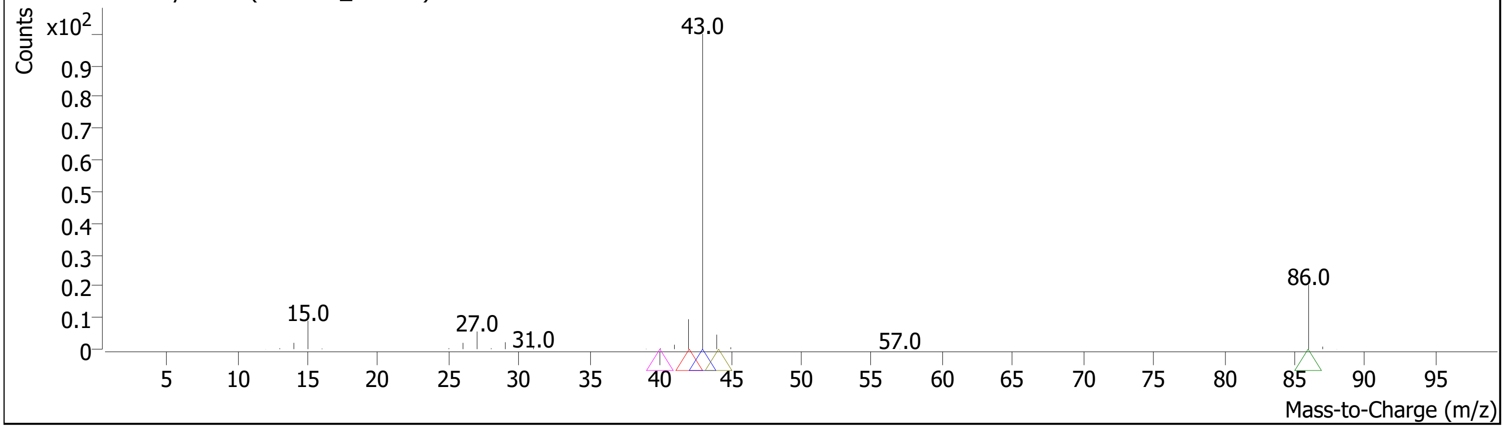

+ Scan (9.9159-10.0943 min, 34 scans) 11795-3.D

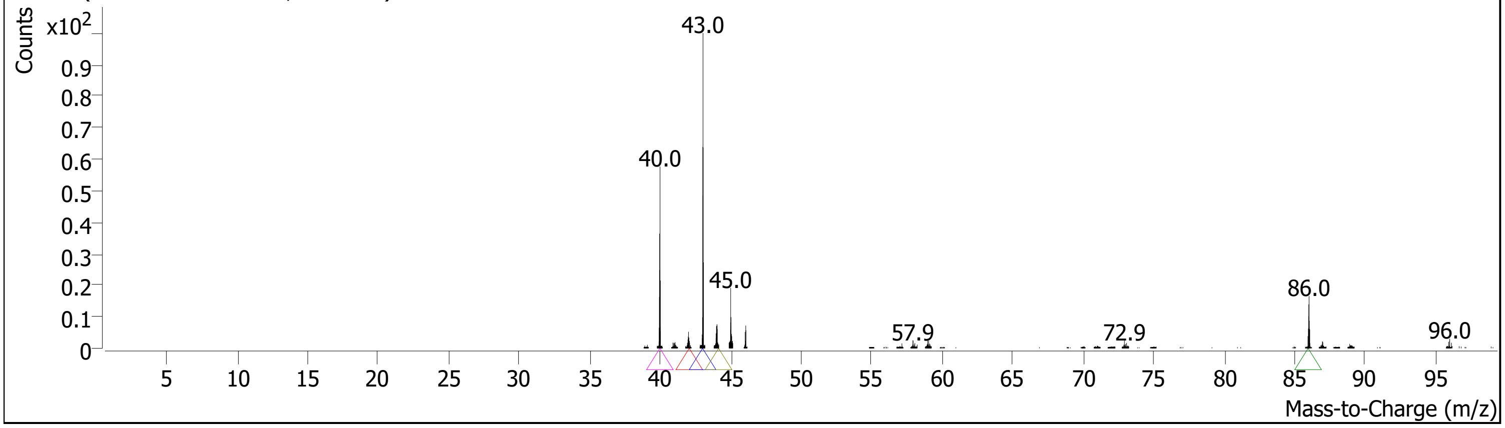

Component RT: 9.9775

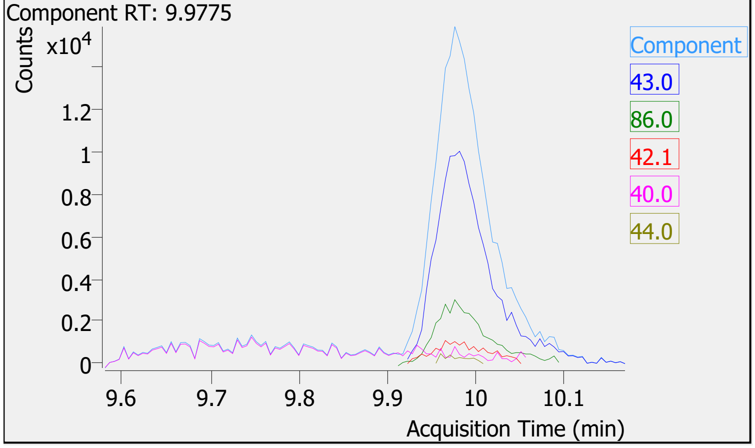

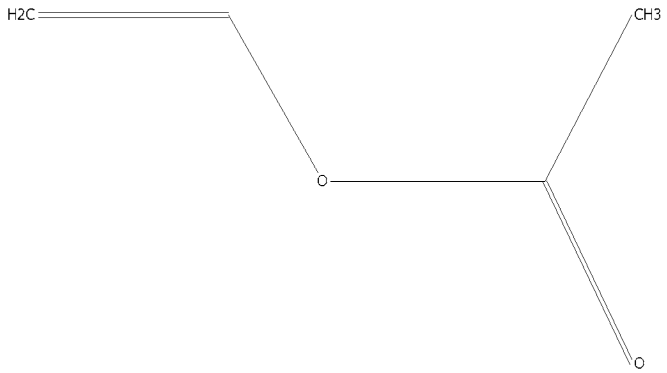

RT	Compound Name	CAS#	Formula	Area	MI	Match Score	Sample	Sample
10.7201	(1R,4R,5R)-4-fluoranyl-1-methyl-5-[(4-methylphenyl)sulfanylmethyl]-7,8-dioxabicyclo[3.2.1]octane	990257-95-4	C15H19FO2S	10776		78.8	0.11	0.19

Component RT: 10.7201

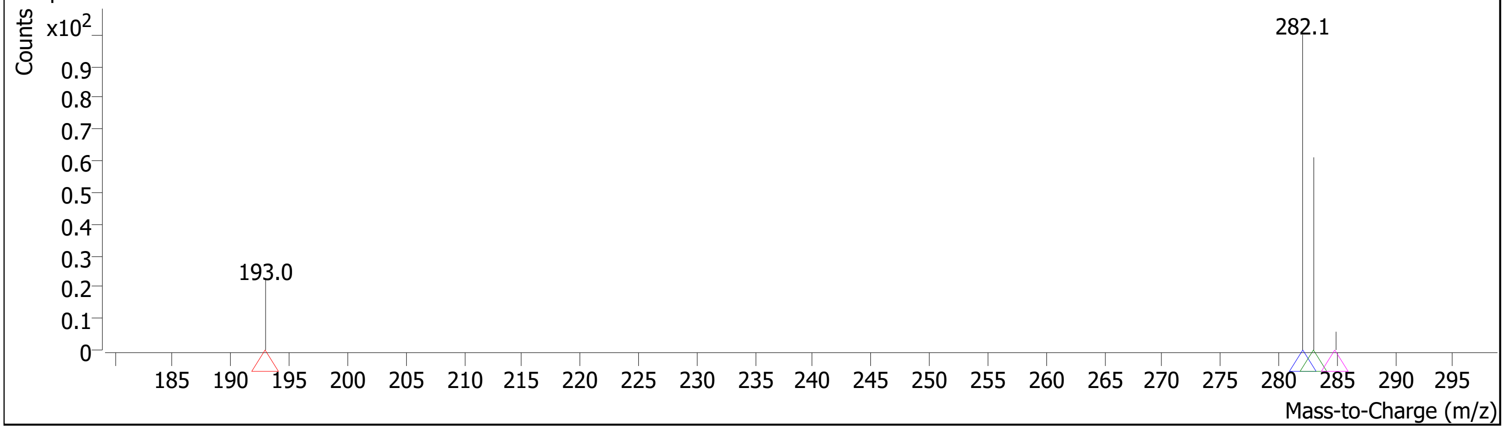

(1R,4R,5R)-4-fluoranyl-1-methyl-5-[(4-methylphenyl)sulfanylmethyl]-7,8-dioxabicyclo[3.2.1]octane (W12N20_MAIN.L)

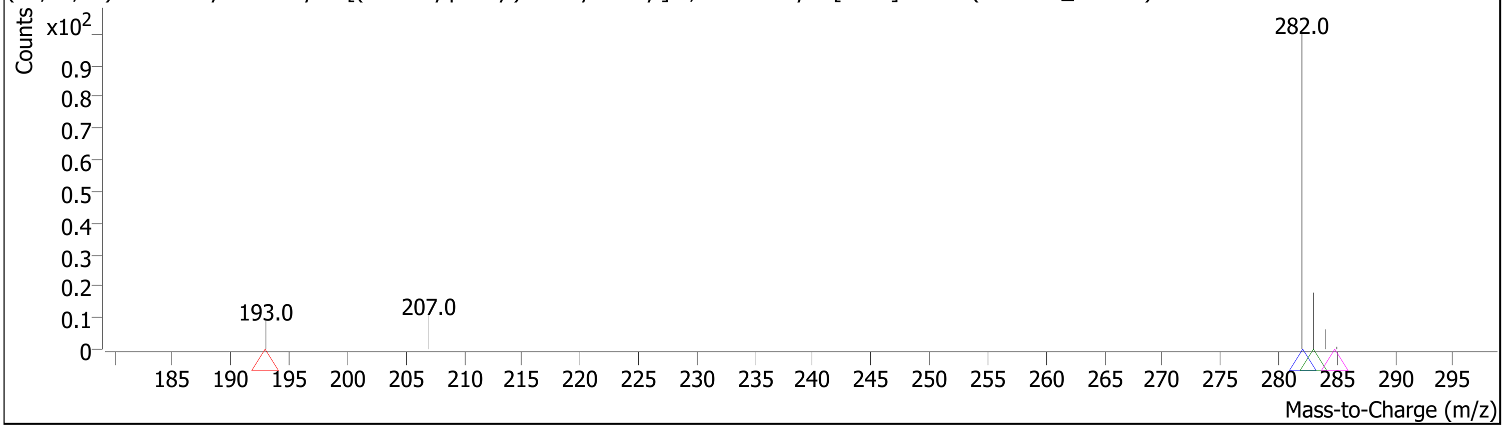

+ Scan (10.6987-10.7361 min, 8 scans) 11795-3.D

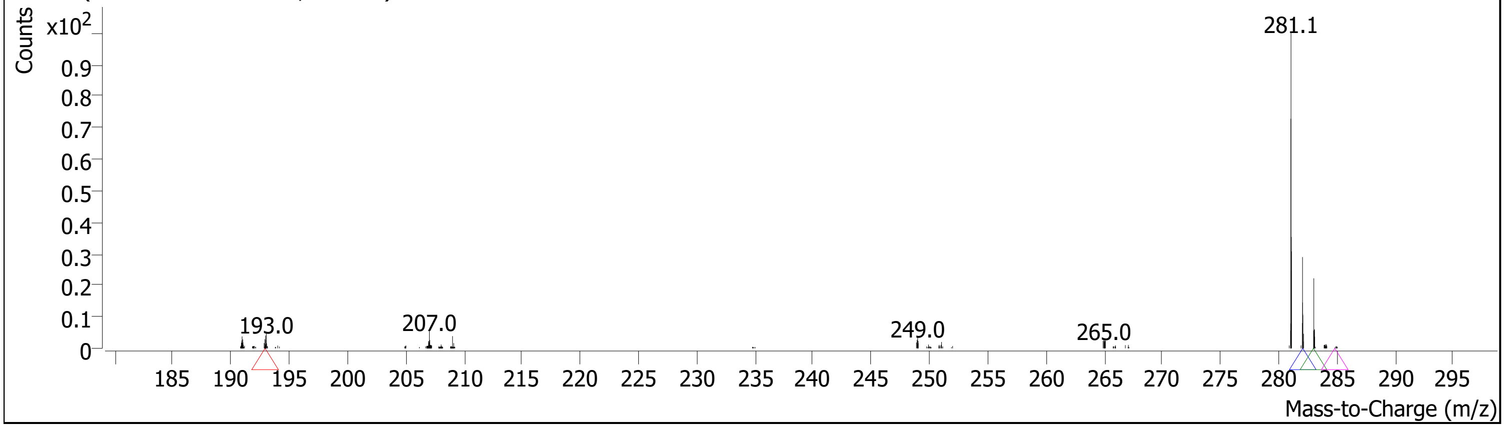

Component RT: 10.7201

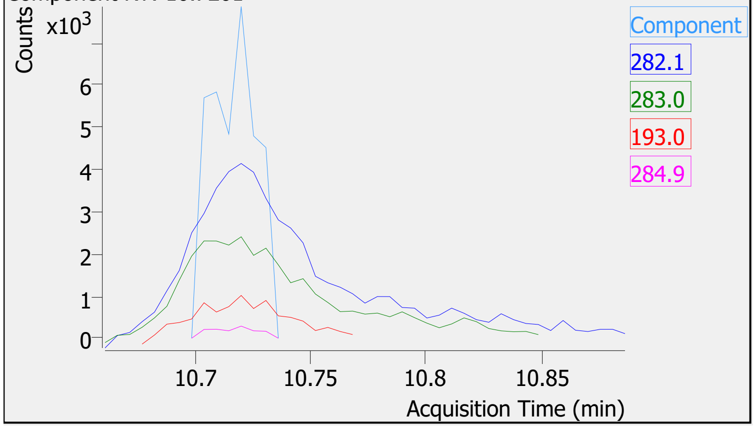

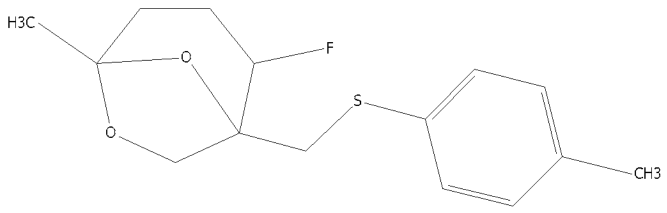

RT	Compound Name	CAS#	Formula	Area	MI	Match Score	Sample	Sample
12.1359	1,1-Dimethoxy-3-(4-nitrophenyl)propan-2-one	990152-35-6	C11H13NO5	20304		82.5	0.21	0.35

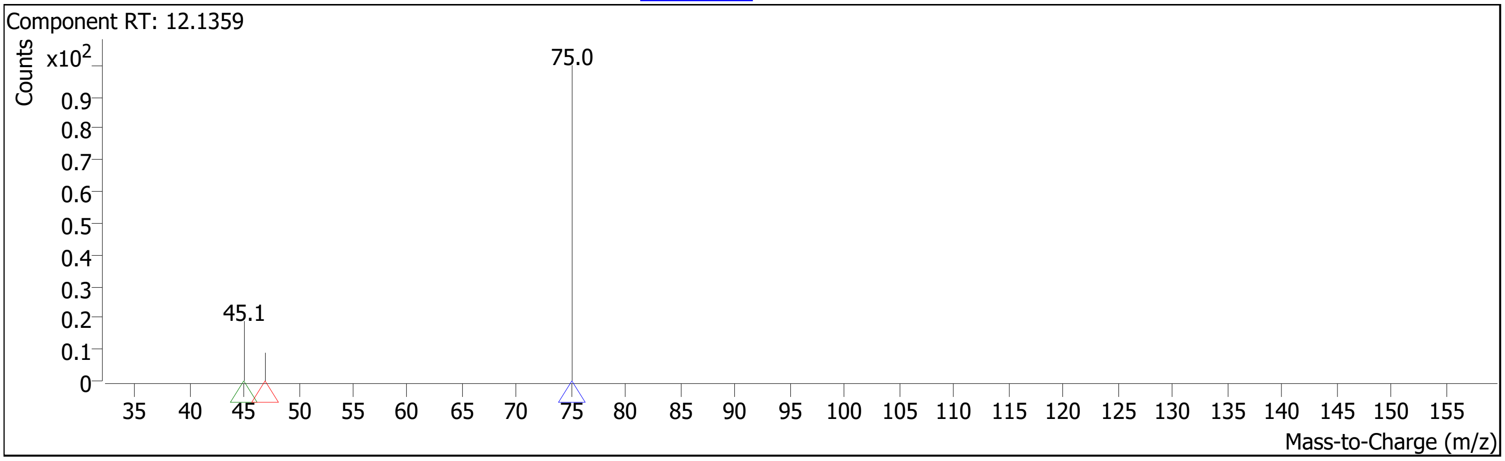

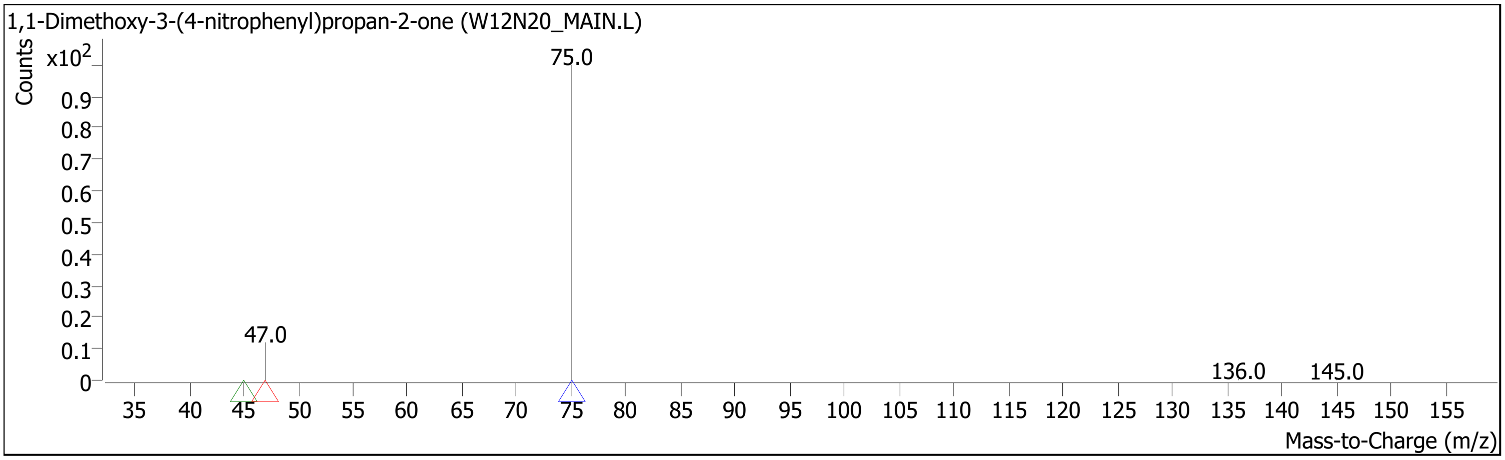

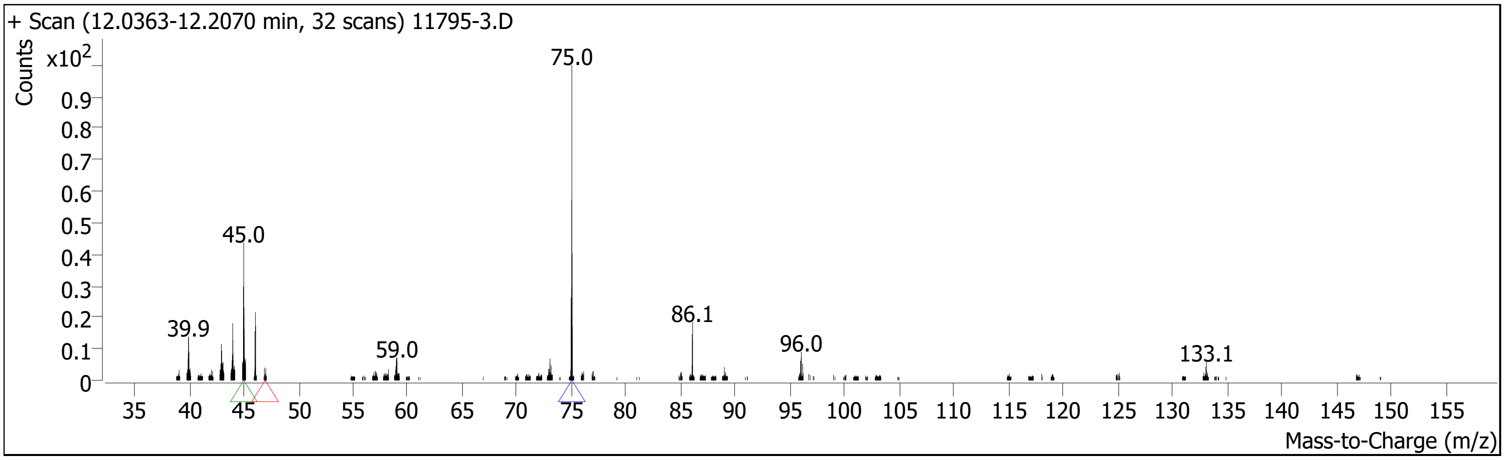

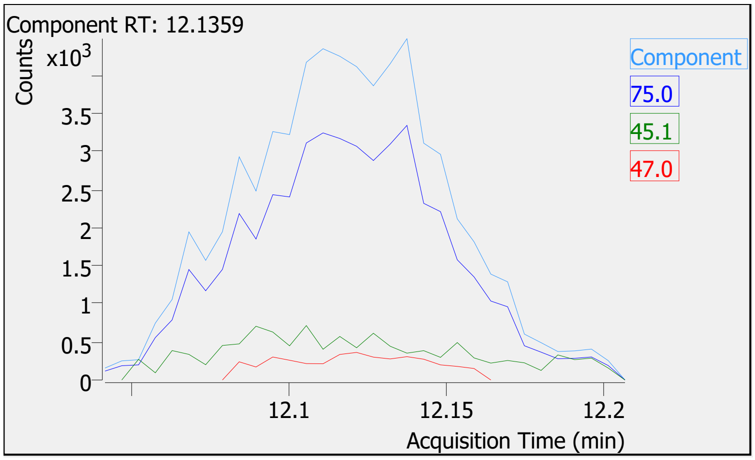

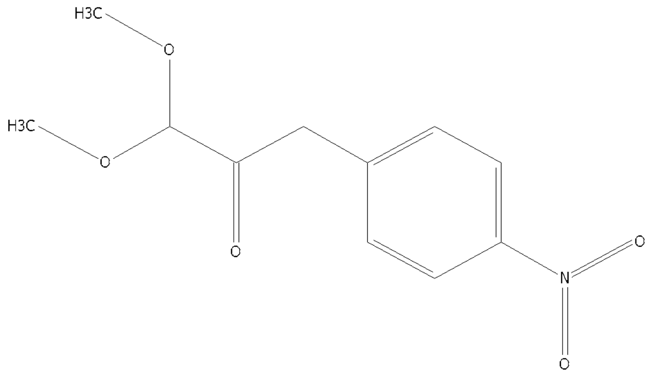

Unknown Analysis Report - Best Hits

RT	Compound Name	CAS#	Formula	Area	MI	Match Score	Sample	Sample
30.2842	5-Methylene-9-decen-2-one	990027-19-1	C11H18O	17948		78.1	0.19	0.31

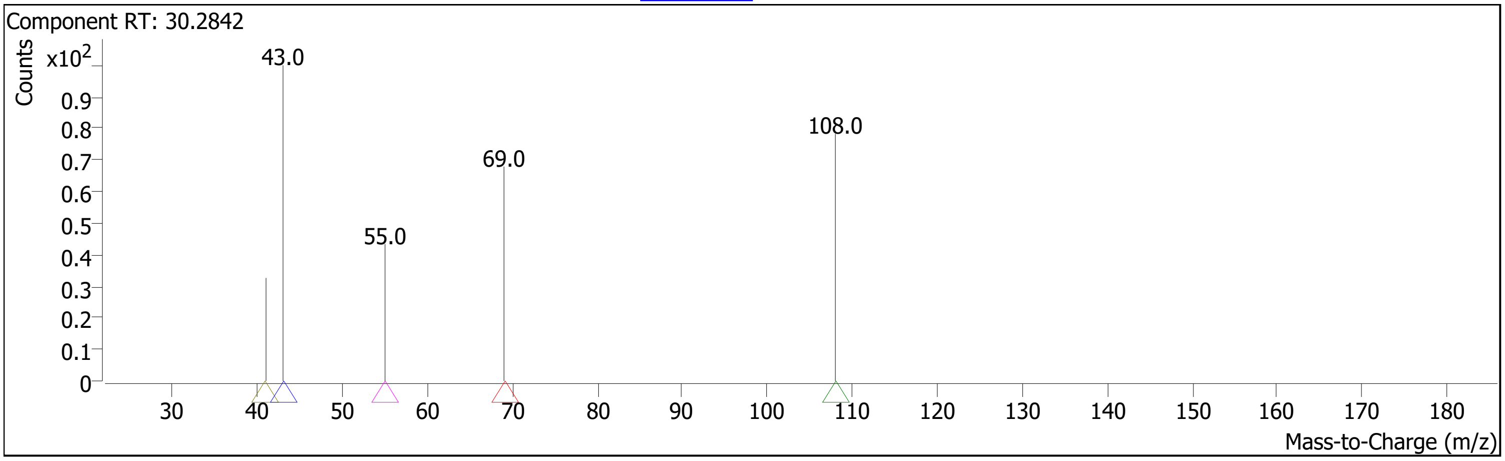

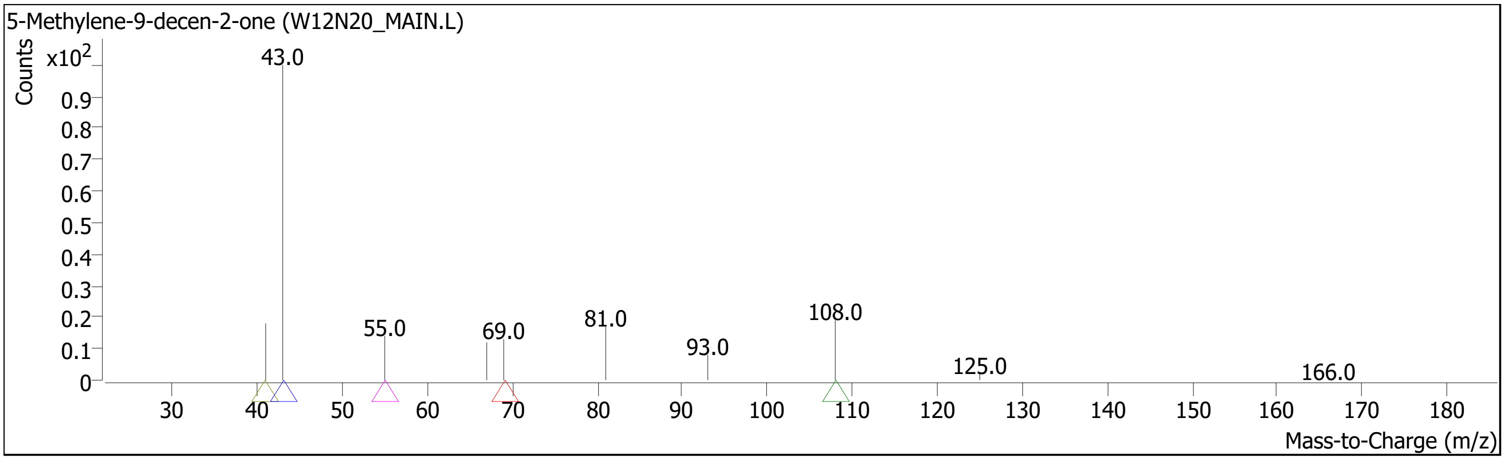

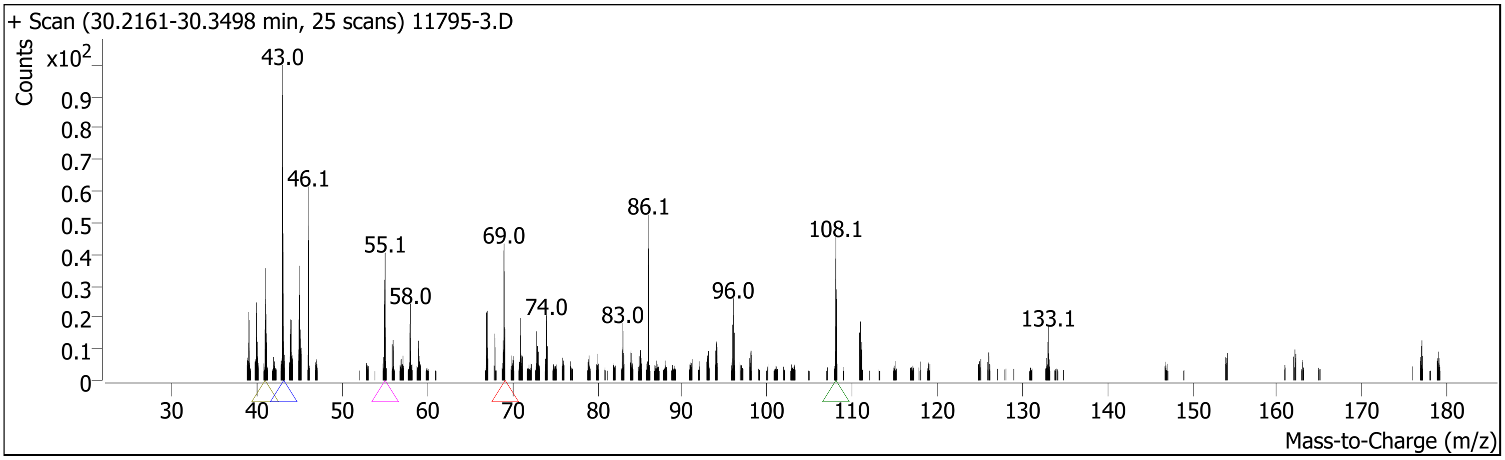

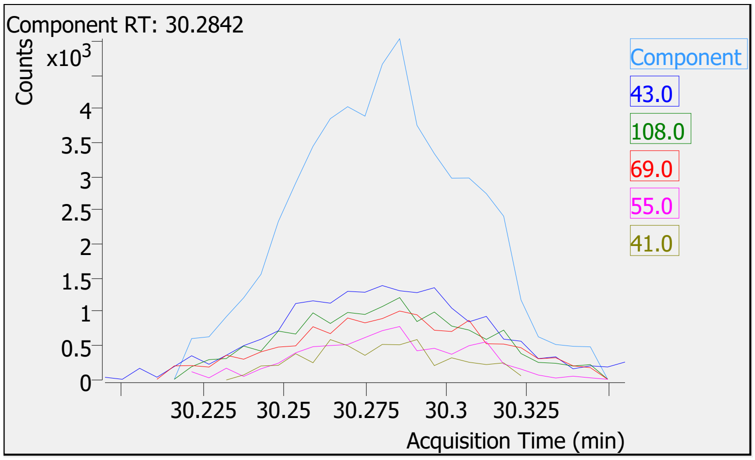

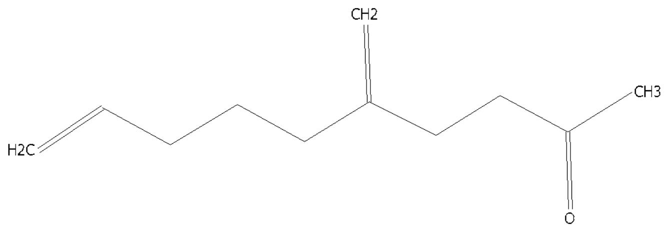

Unknown Analysis Report - Best Hits

RT	Compound Name	CAS#	Formula	Area	MI	Match Score	Sample	Sample
30.6266	1-Ethyl-2-(4'-fluorophenyl)-4,5-diphenyl-imidazole	990407-68-5	C23H19FN2	60271		72.2	0.63	1.04

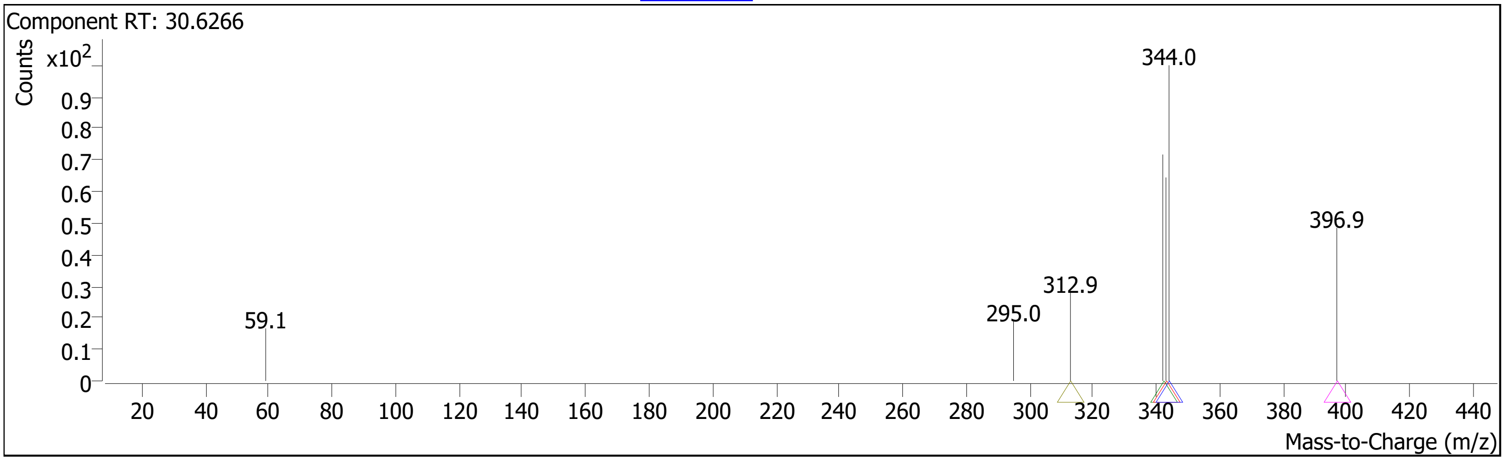

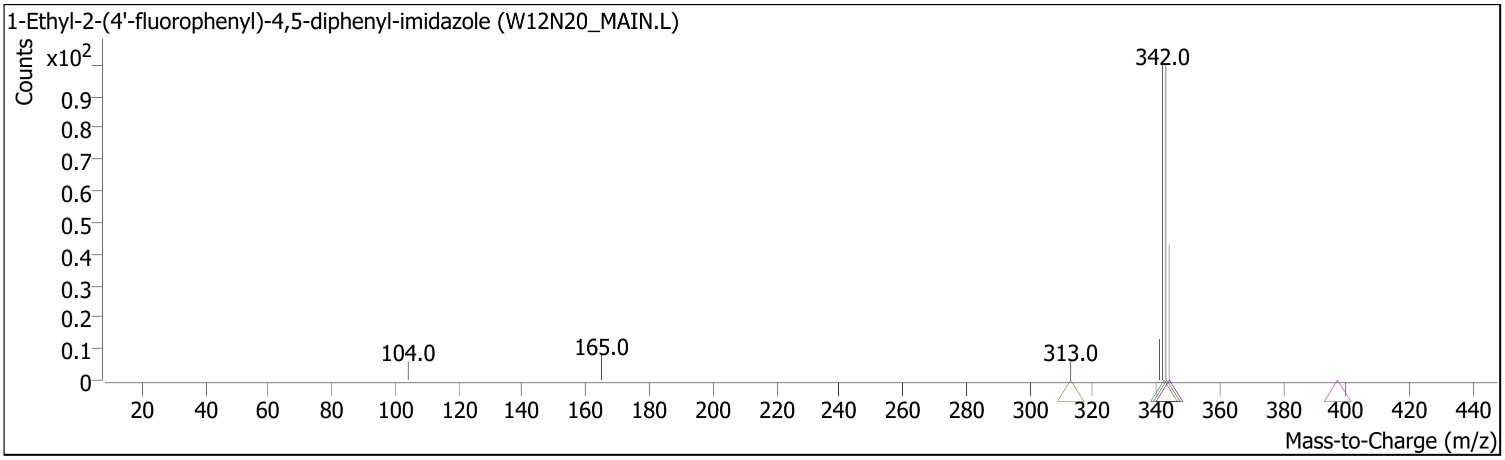

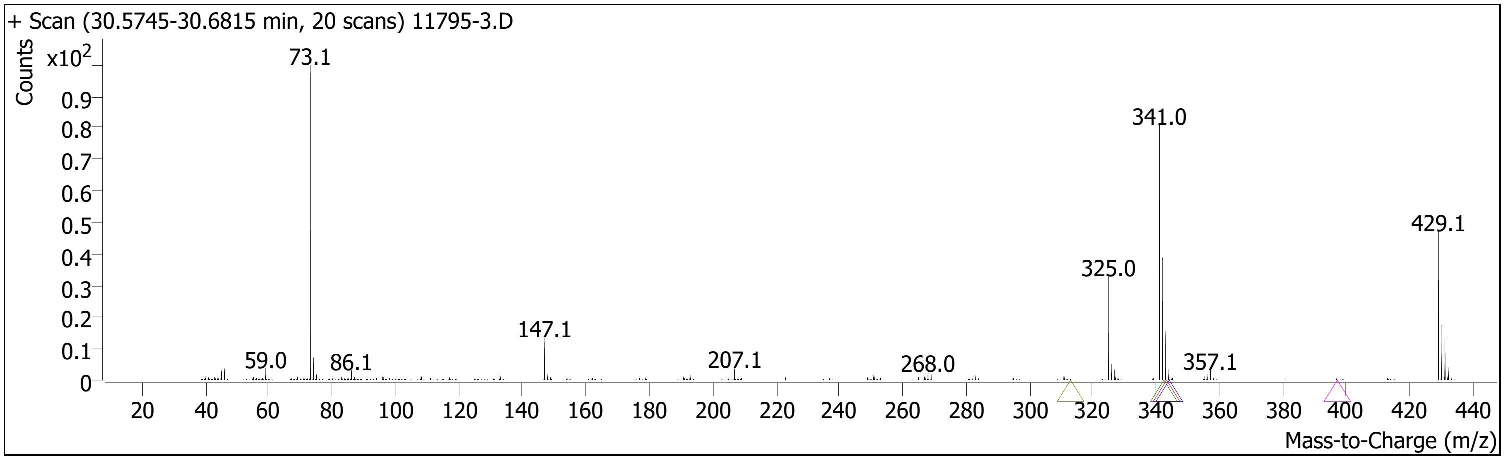

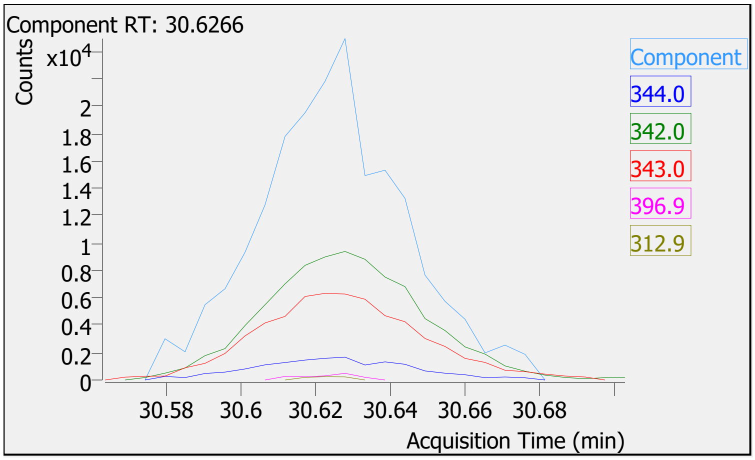

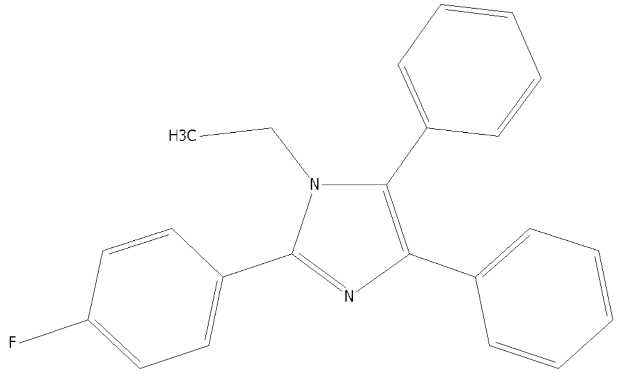

RT	Compound Name	CAS#	Formula	Area	MI	Match Score	Sample	Sample
36.1412	1,2-Di-tert-butylbenzene	1012-76-6	C14H22	84197		85.3	0.88	1.45

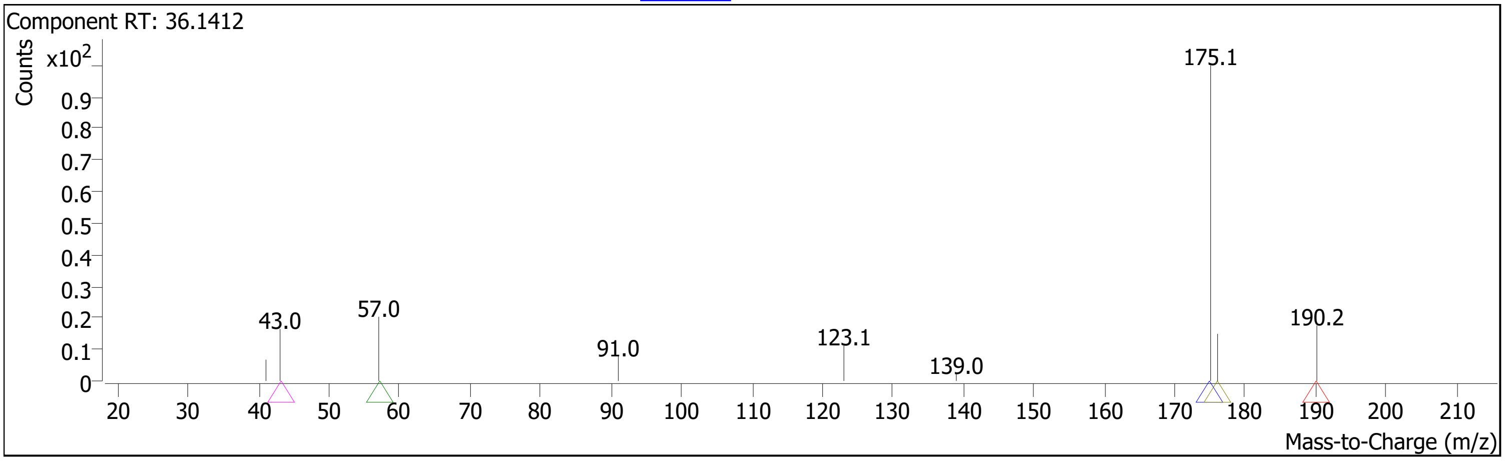

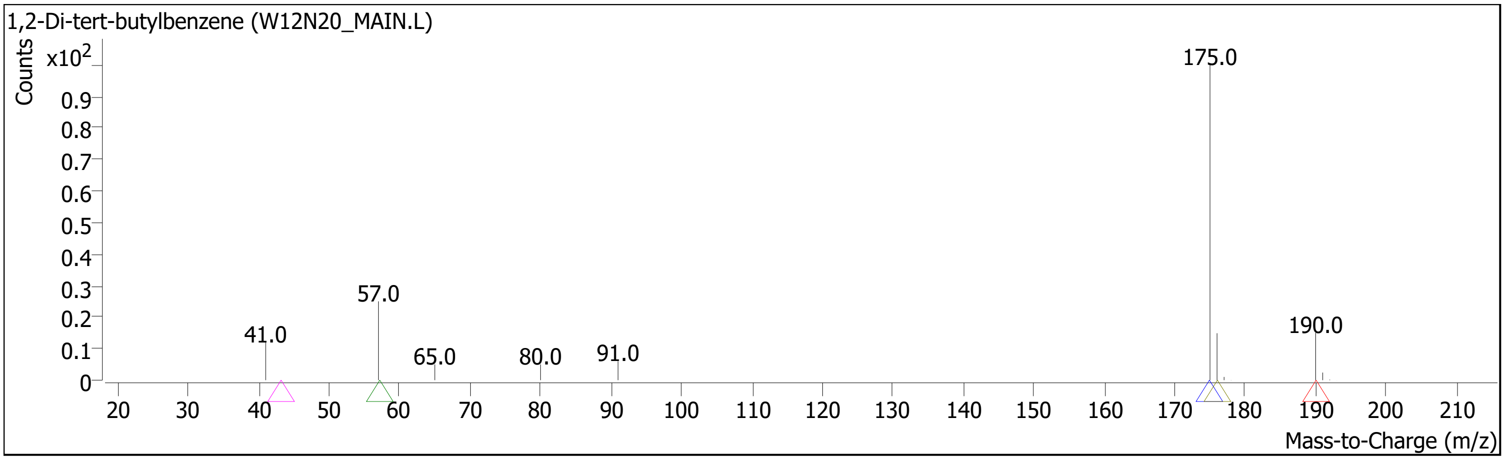

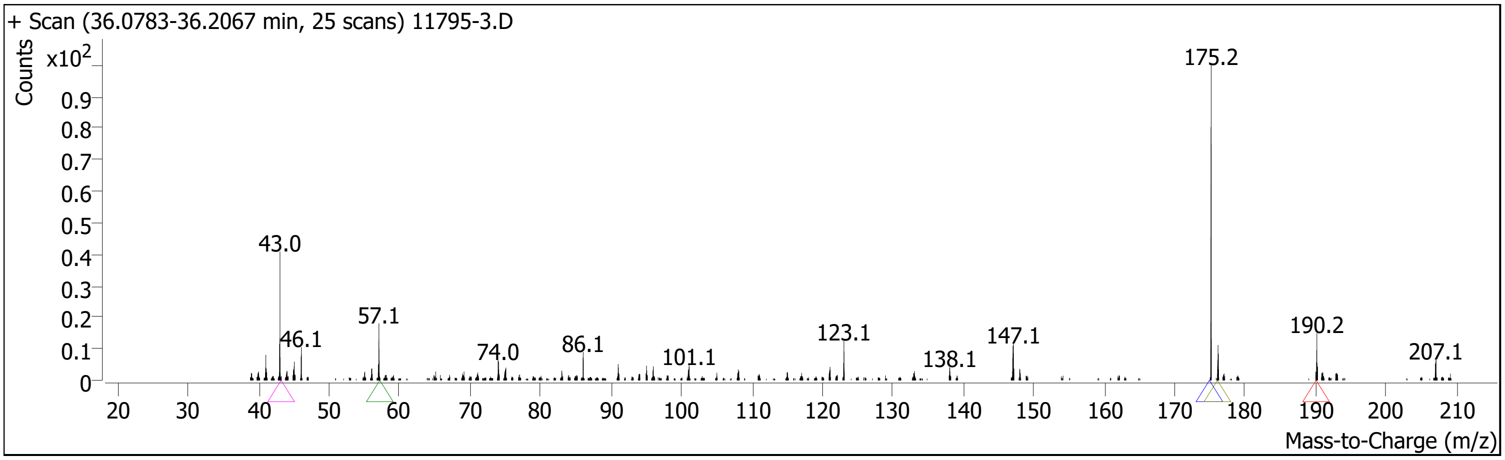

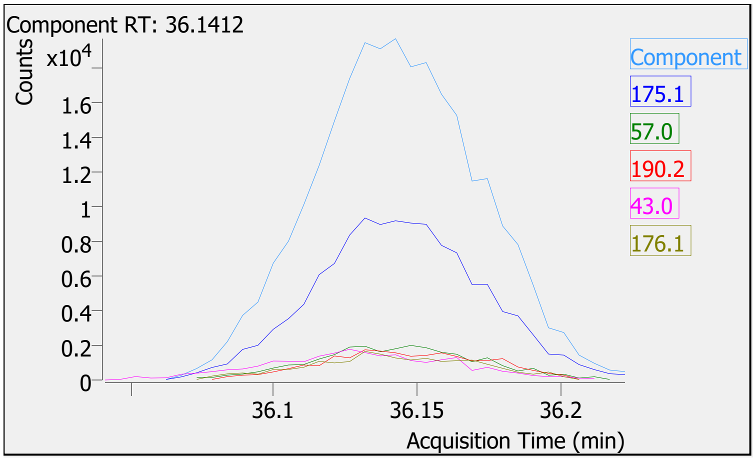

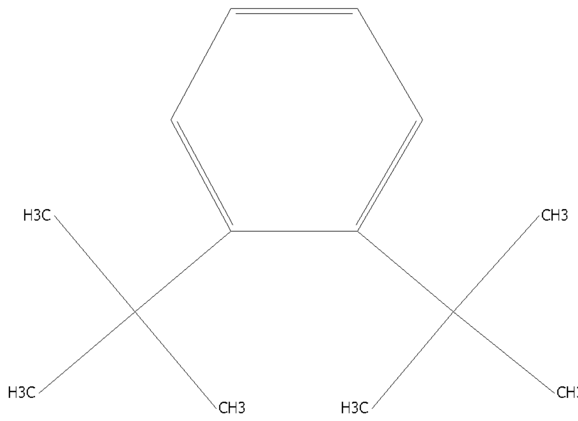

 Generated at 4:34 PM on 11/14/2023

Unknown Analysis Report - Best Hits

RT	Compound Name	CAS#	Formula	Area	MI	Match Score	Sample	Sample
40.1620	1-Hexanol, 2-ethyl-	104-76-7	C8H18O	58324		82.9	0.61	1.01

Component RT: 40.1620

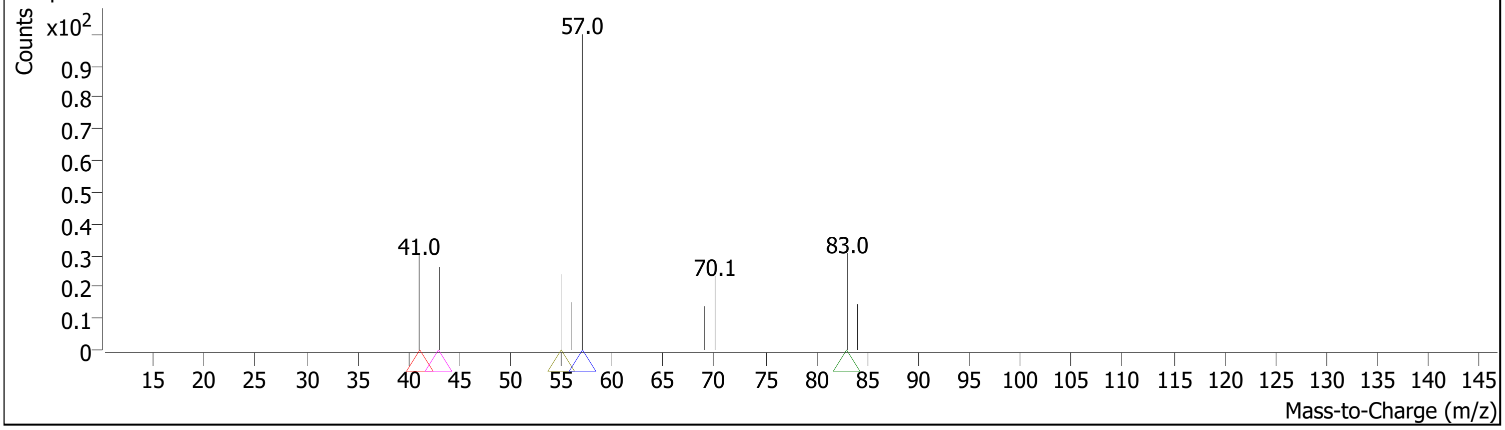

1-Hexanol, 2-ethyl- (W12N20_MAIN.L)

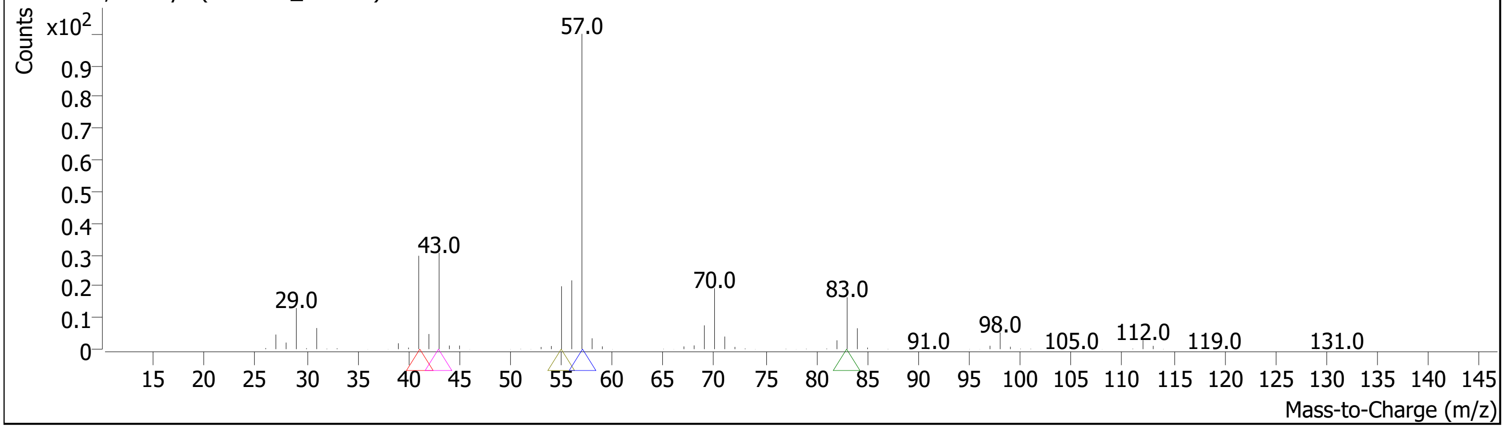

+ Scan (40.0794-40.2396 min, 30 scans) 11795-3.D

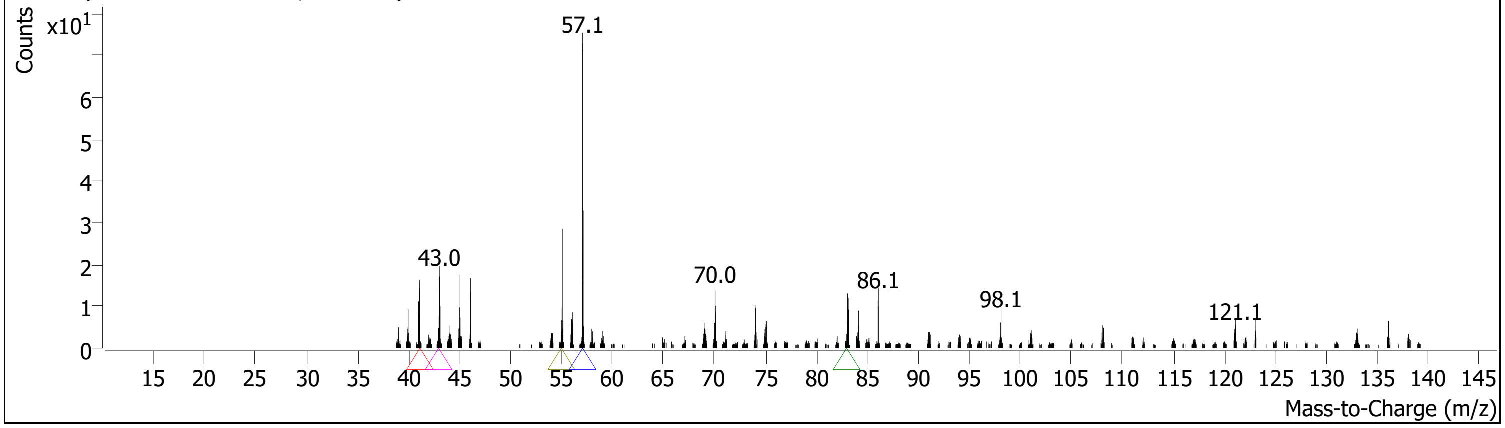

Component RT: 40.1620

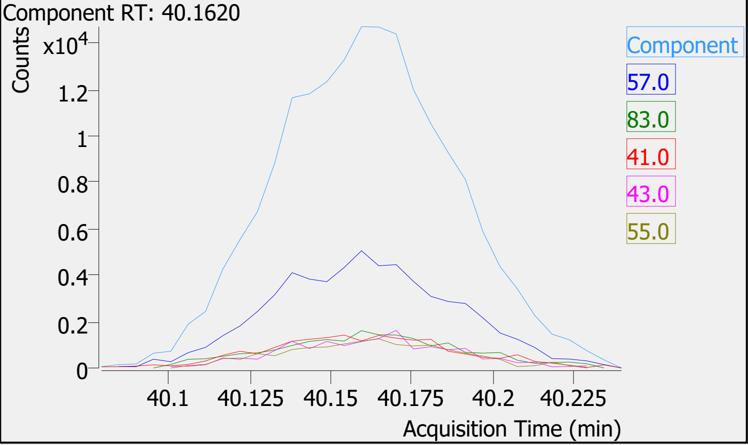

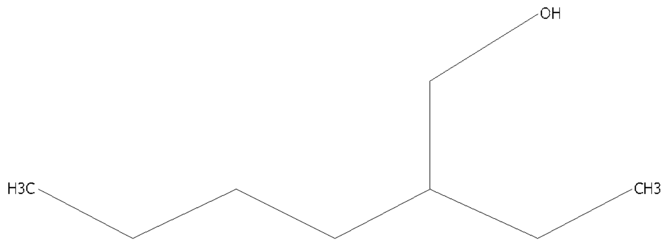

RT	Compound Name	CAS#	Formula	Area	MI	Match Score	Sample	Sample
41.6954	(anti/syn)-2-Nitro-1-phenylpropan-1,3-diol	990067-30-9	C9H11NO4	130696		98.7	1.37	2.26

Component RT: 41.6954

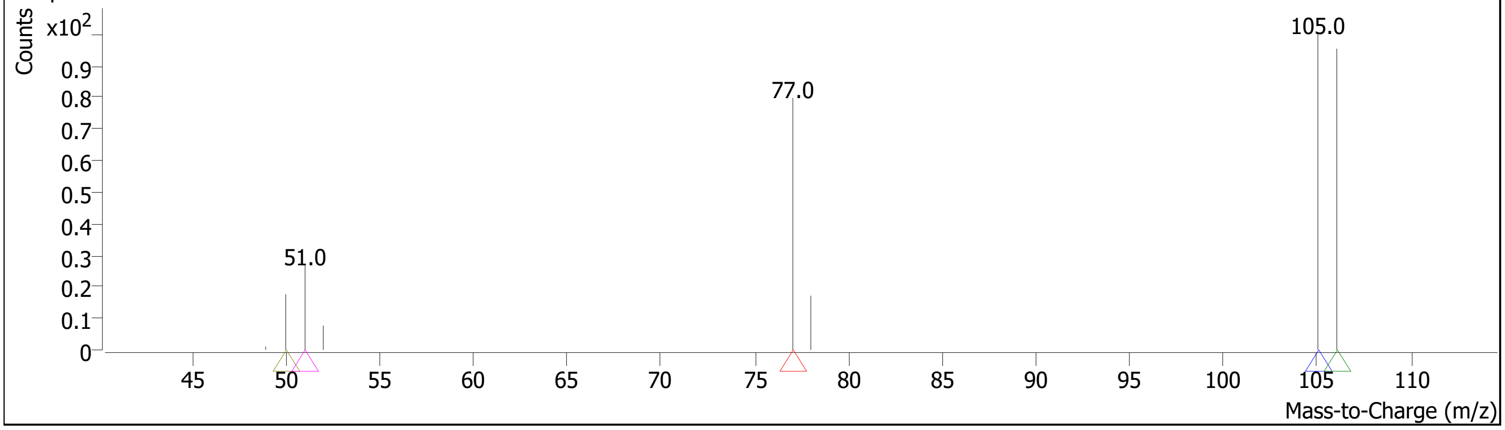

(anti/syn)-2-Nitro-1-phenylpropan-1,3-diol (W12N20_MAIN.L)

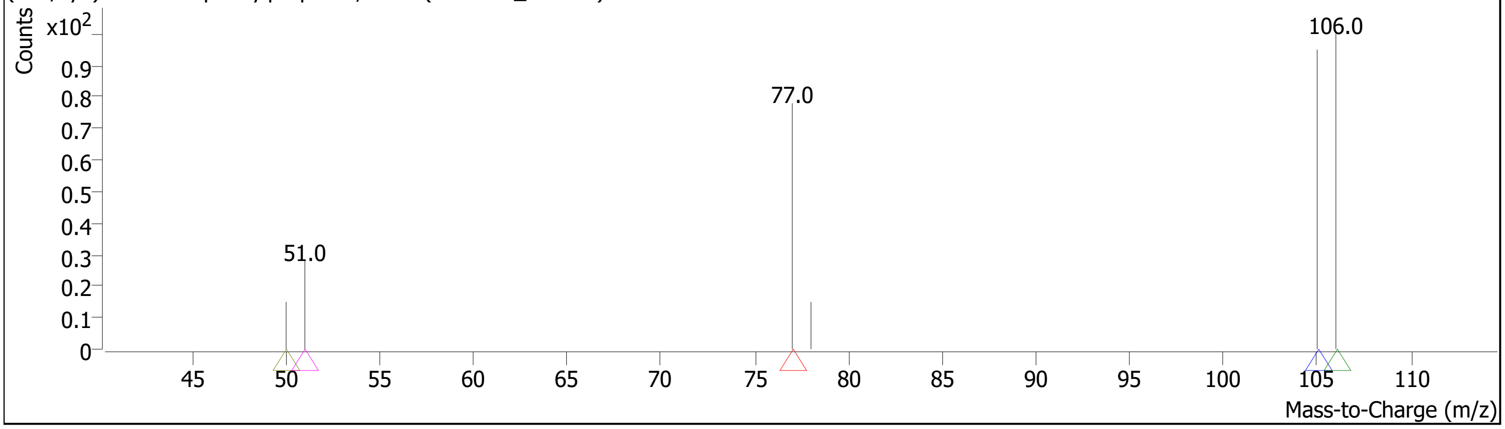

+ Scan (41.6303-41.8014 min, 33 scans) 11795-3.D

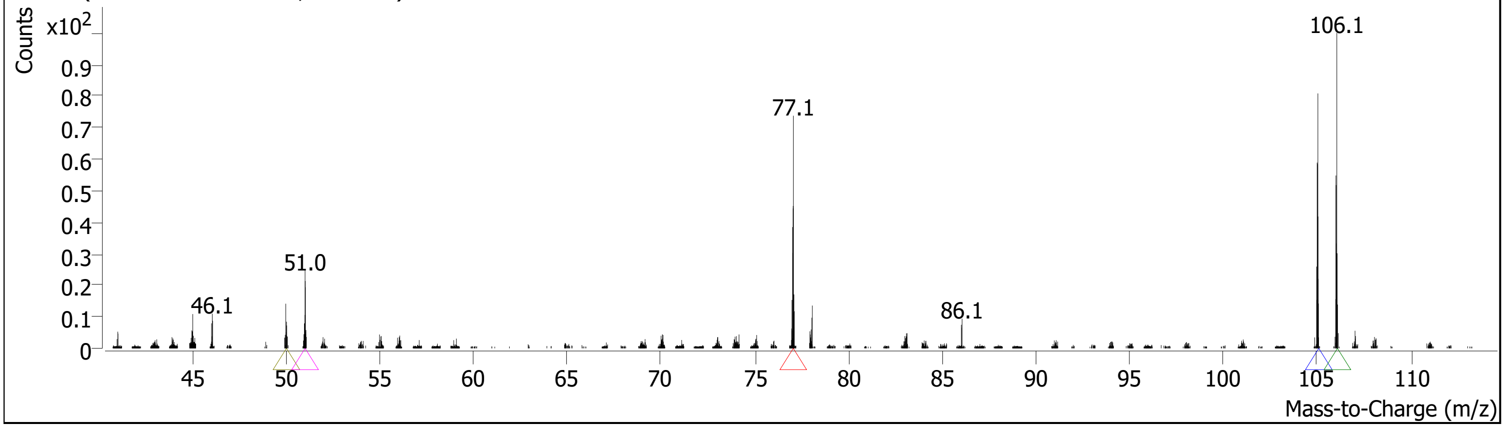

Component RT: 41.6954

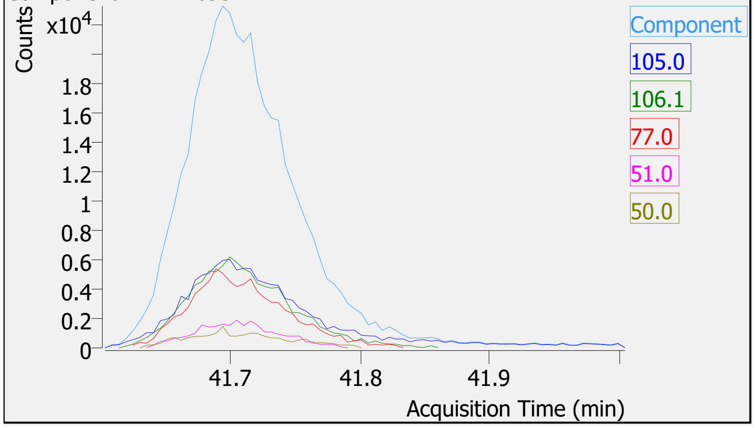

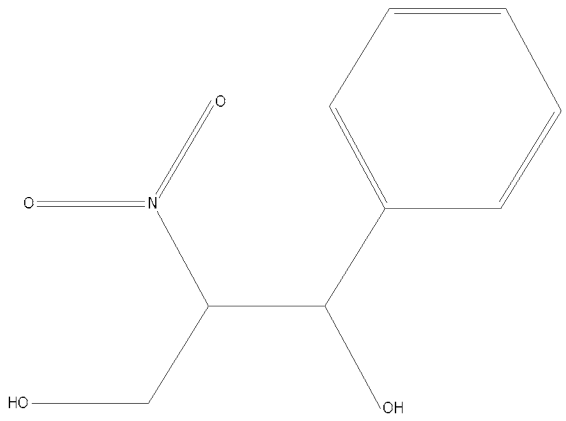

Unknown Analysis Report - Best Hits

RT	Compound Name	CAS#	Formula	Area	MI	Match Score	Sample	Sample
42.5829	6-(2-Aminophenyl)-1-methylpyrido[2,3-d]pyrimidine-2,4(1H,3H)-dione	990222-52-0	C14H12N4O2	17779		72.2	0.19	0.31

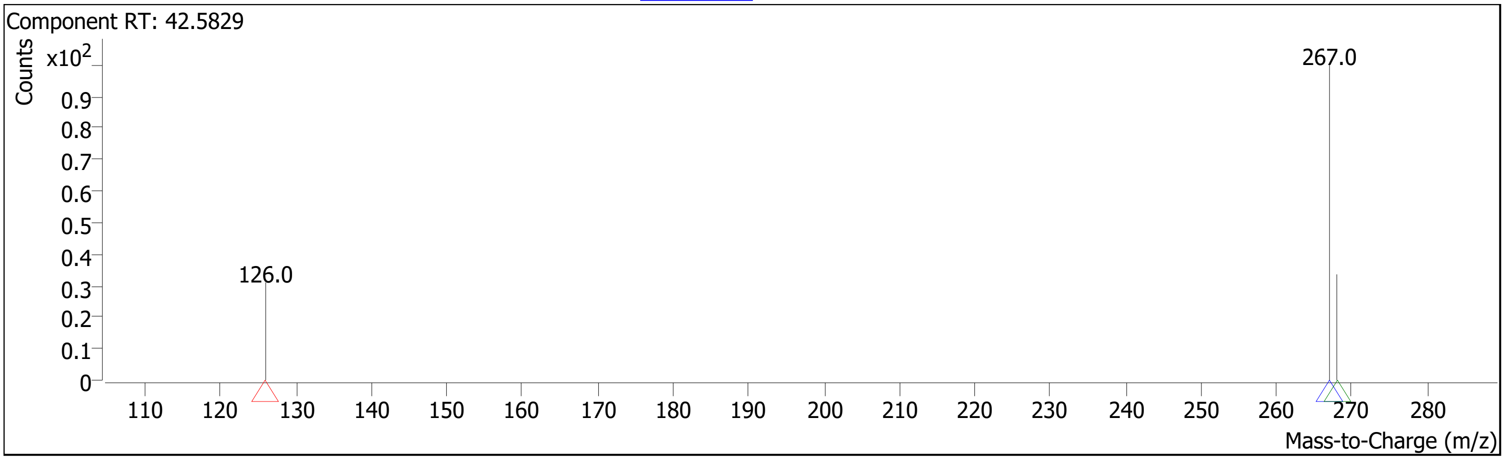

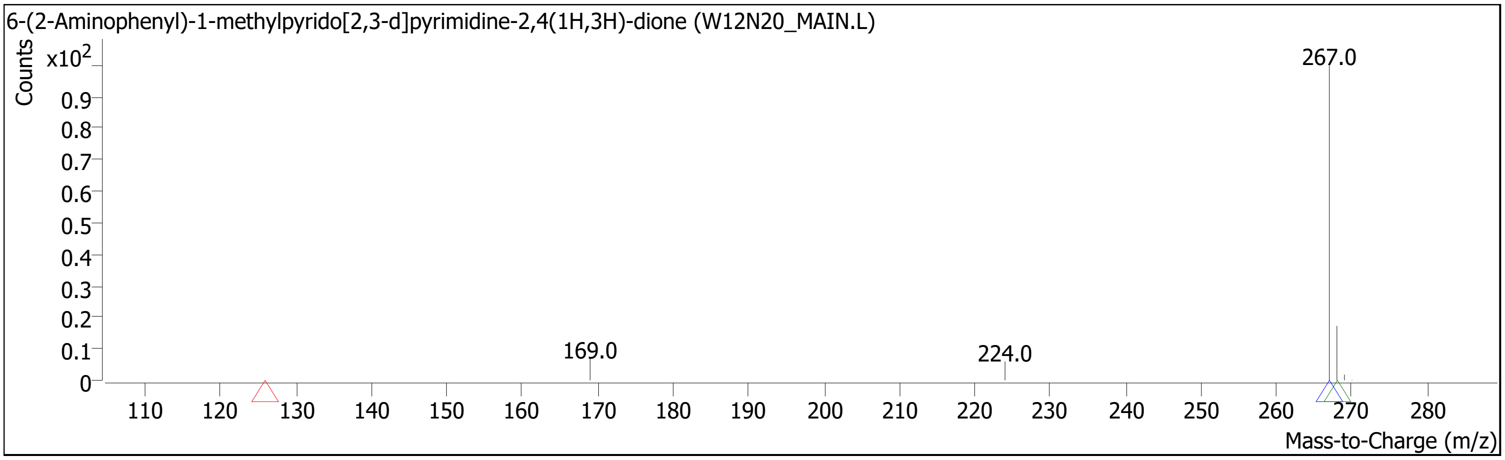

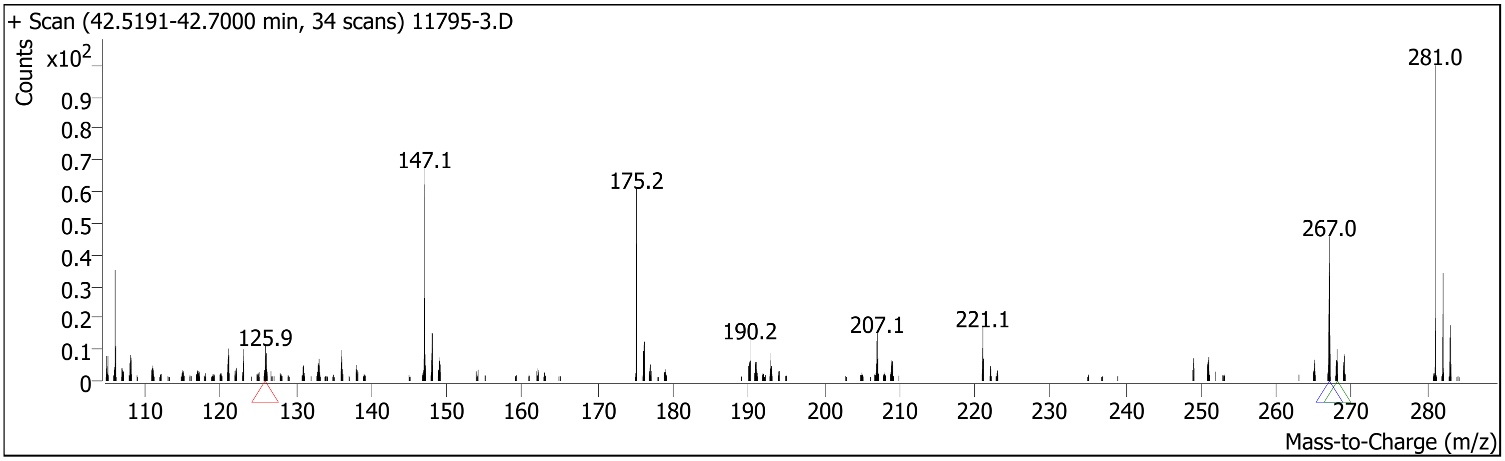

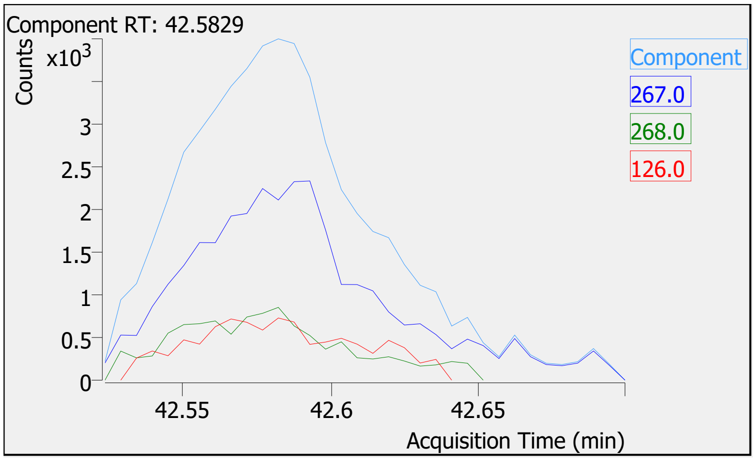

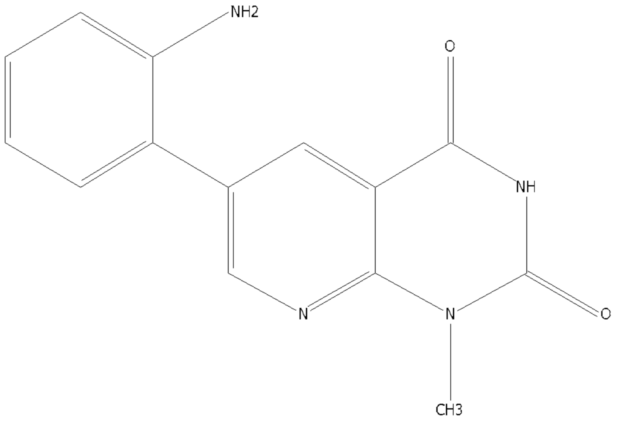

Unknown Analysis Report - Best Hits

RT	Compound Name	CAS#	Formula	Area	MI	Match Score	Sample	Sample
44.9567	Methyl 5-endo-4-hydroxy-2,5,7,7-tetramethylbicyclo[2.2.2]oct-2-en-5-carboxylate	990150-69-0	$C_{14}H_{22}O_3$	18682		86.1	0.20	0.32

Component RT: 44.9567

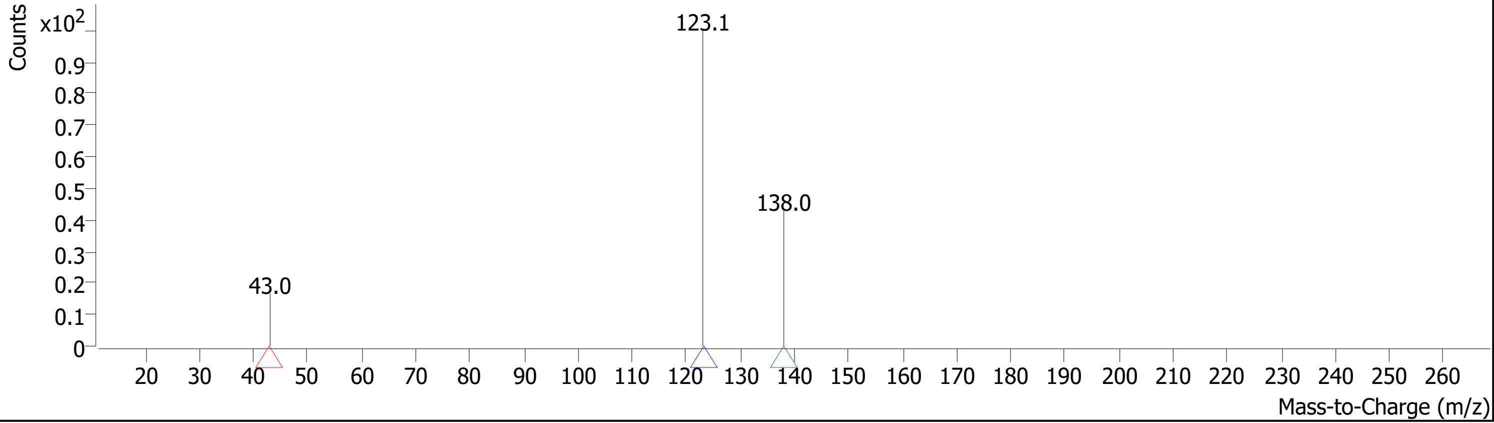

Methyl 5-endo-4-hydroxy-2,5,7,7-tetramethylbicyclo[2.2.2]oct-2-en-5-carboxylate (W12N20_MAIN.L)

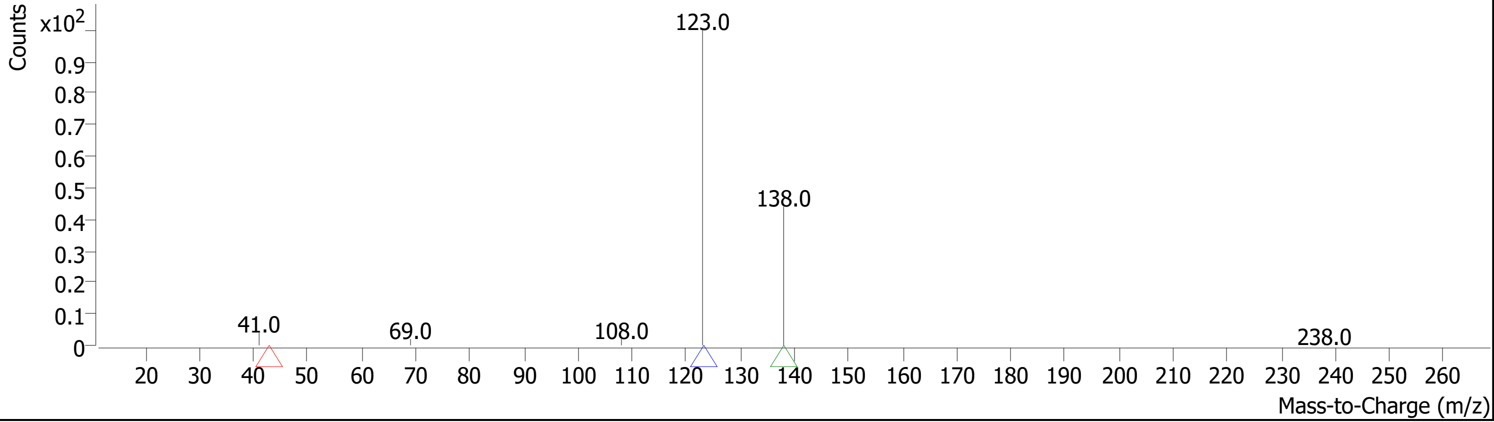

+ Scan (44.8930-45.0213 min, 25 scans) 11795-3.D

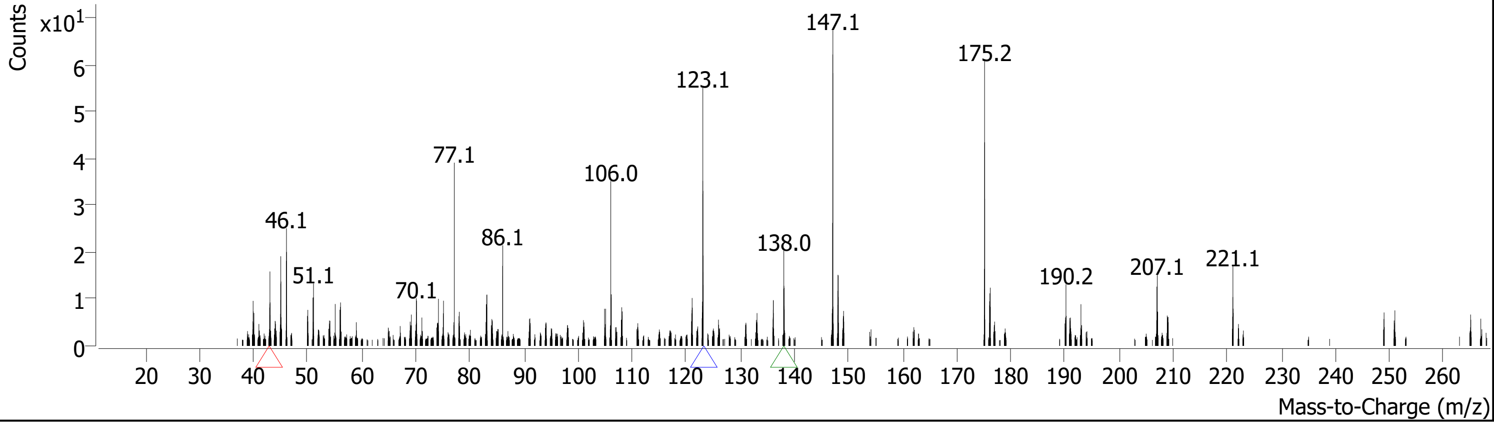

Component RT: 44.9567

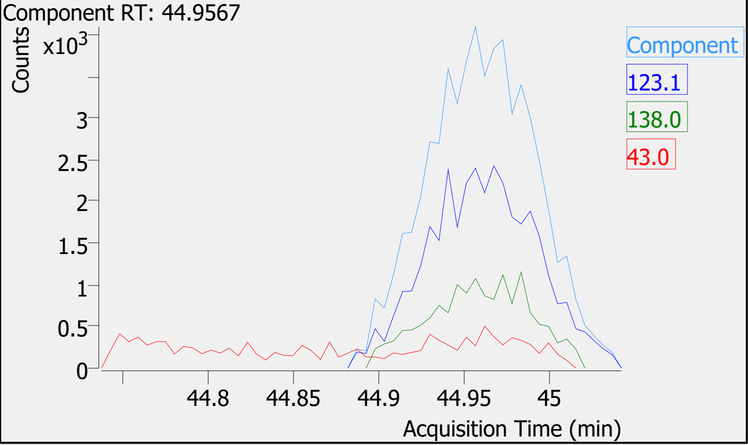

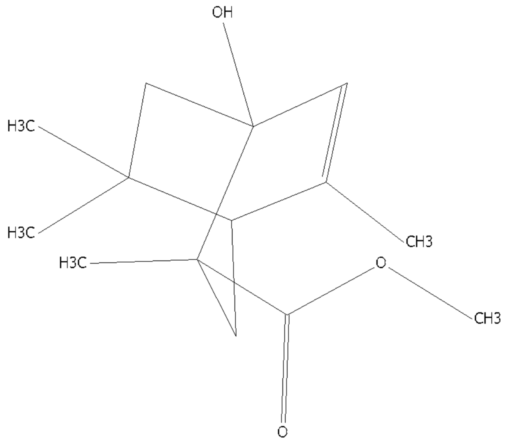

 Generated at 4:34 PM on 11/14/2023

RT	Compound Name	CAS#	Formula	Area	MI	Match Score	Sample	Sample
46.7473	2,2-Dimethylpropanoic acid tert-butyl ester	16474-43-4	C9H18O2	31099		84.4	0.33	0.54

Component RT: 46.7473

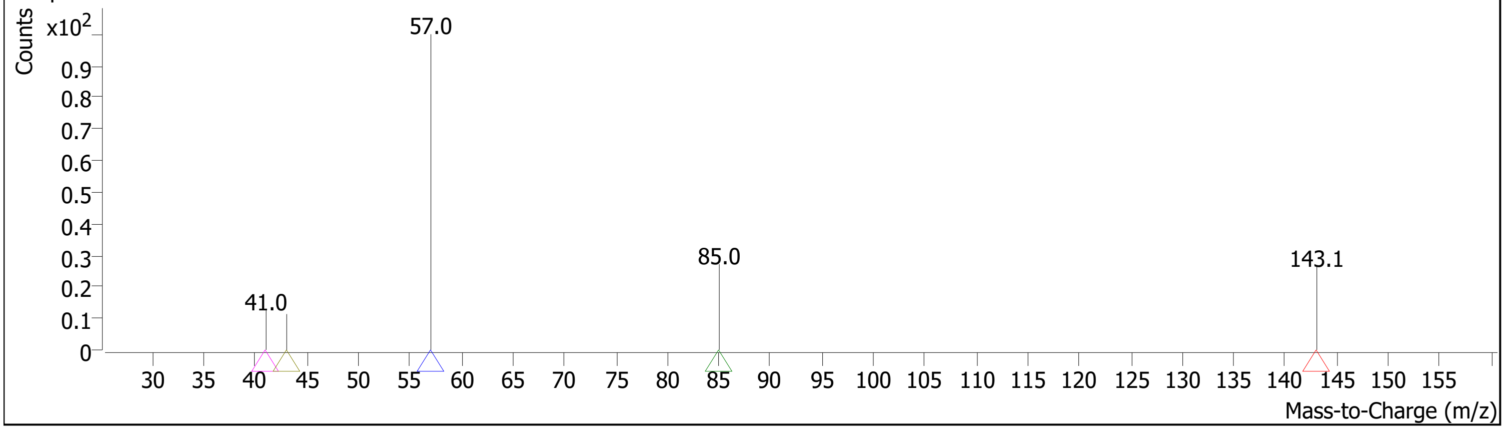

2,2-Dimethylpropanoic acid tert-butyl ester (W12N20_MAIN.L)

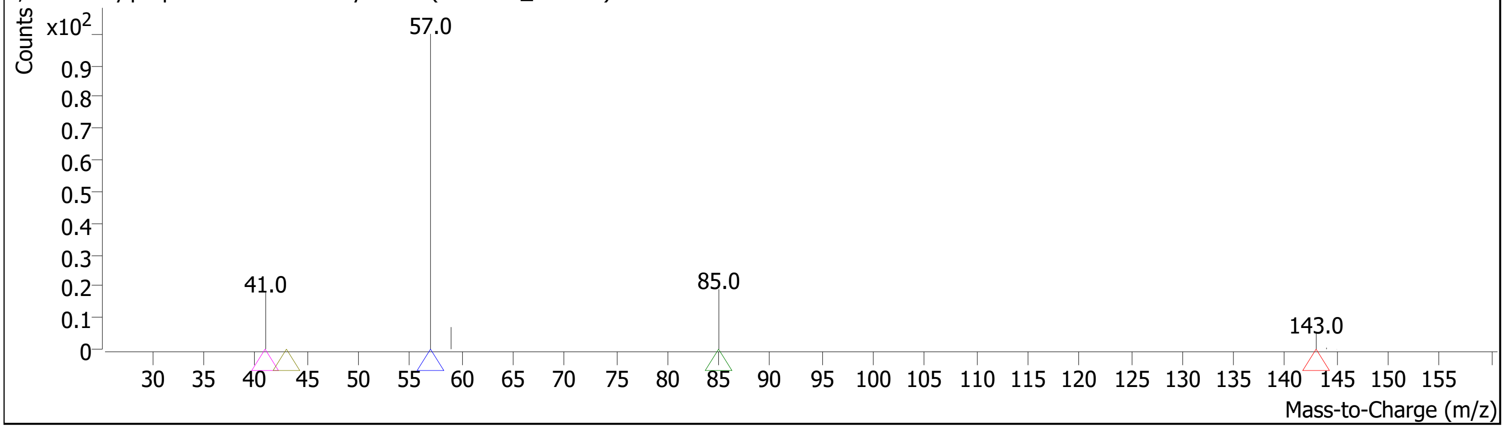

+ Scan (46.6901-46.8025 min, 22 scans) 11795-3.D

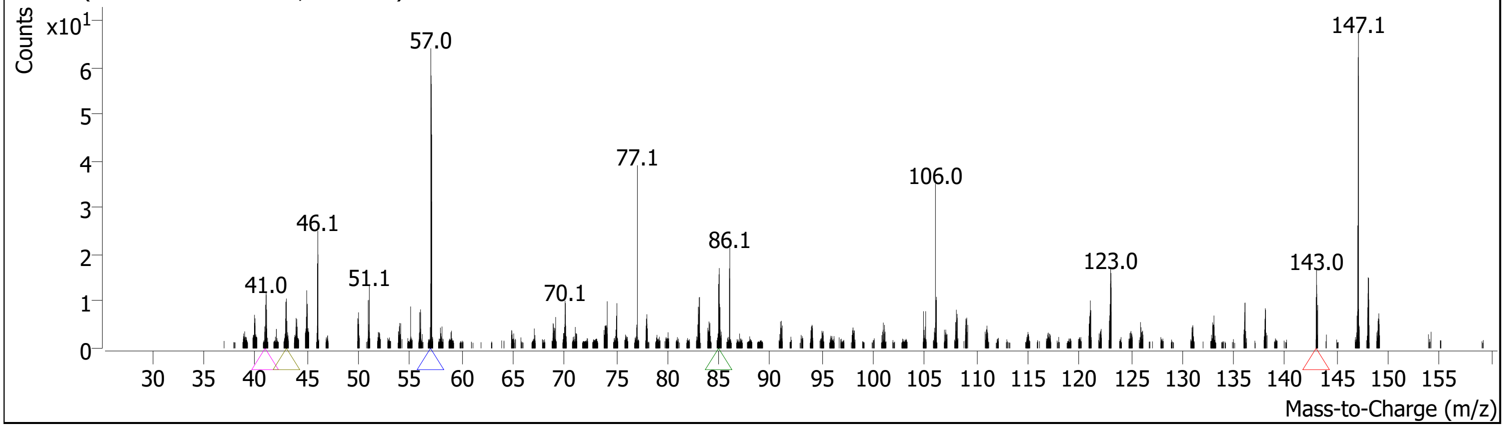

Component RT: 46.7473

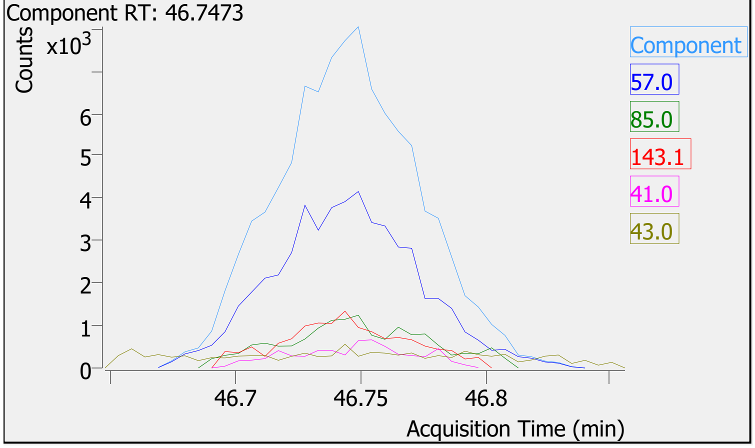

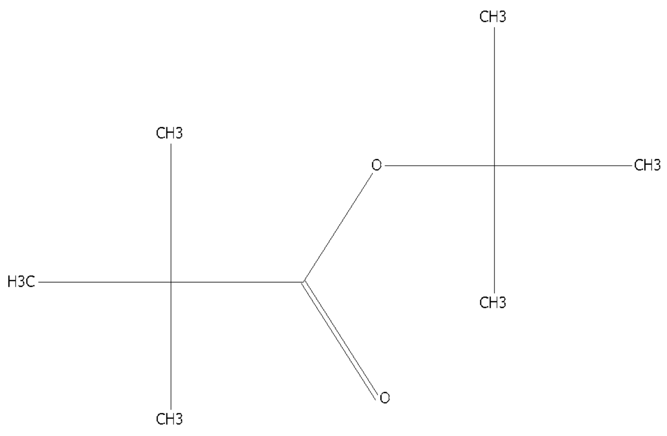

RT	Compound Name	CAS#	Formula	Area	MI	Match Score	Sample	Sample
47.7239	(2R,3S)-2-hydroxy-1-phenyl-3-vinyl-hexan-1-one	990106-41-5	C14H18O2	92247		91.3	0.97	1.59

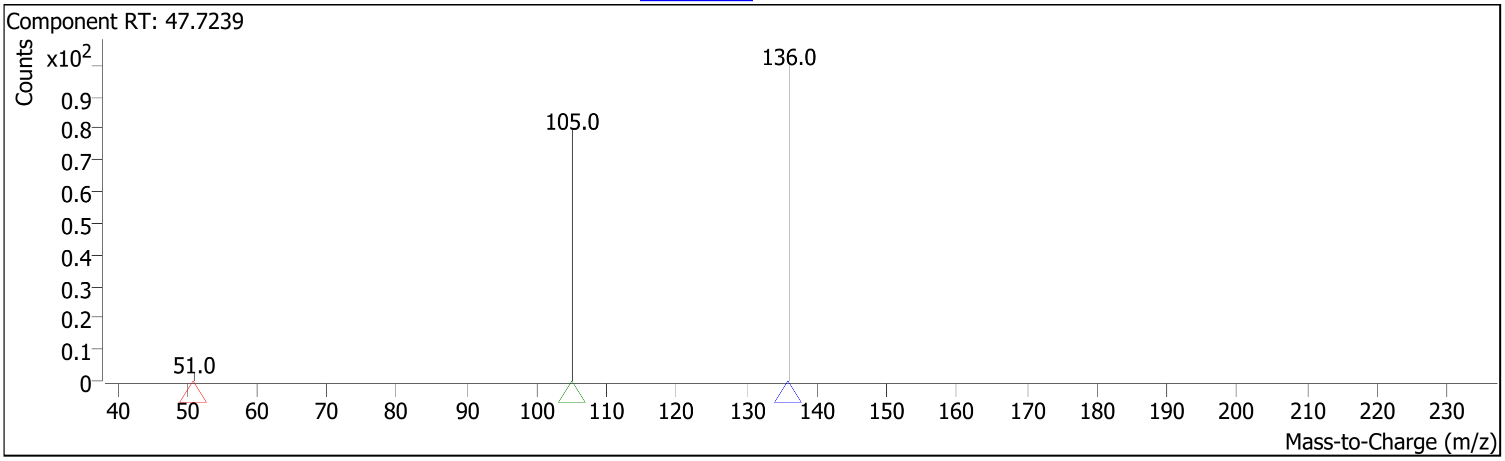

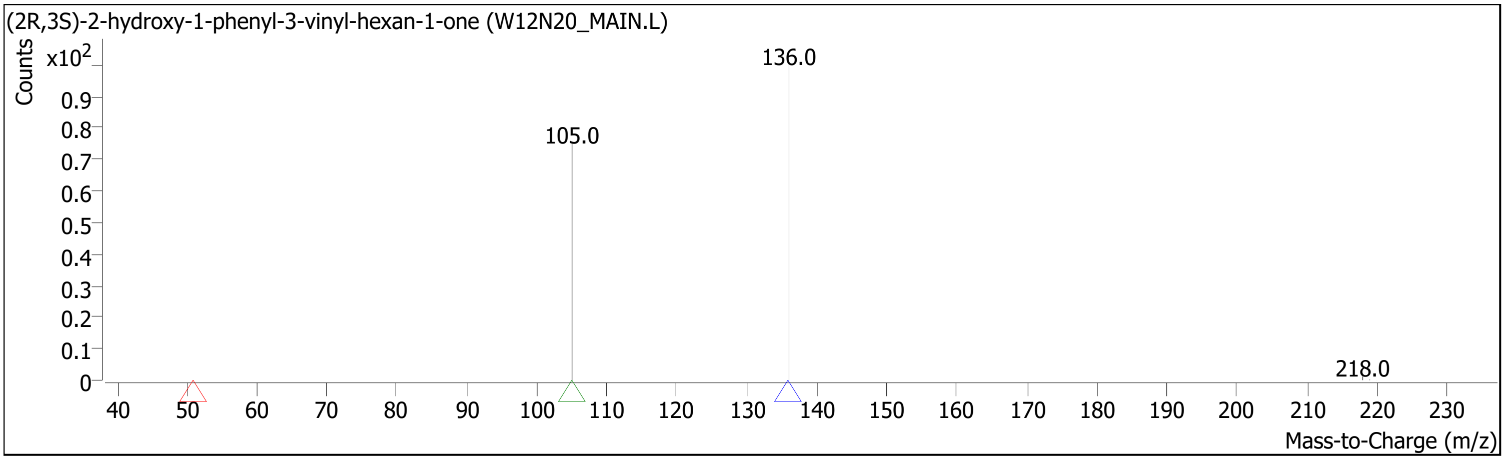

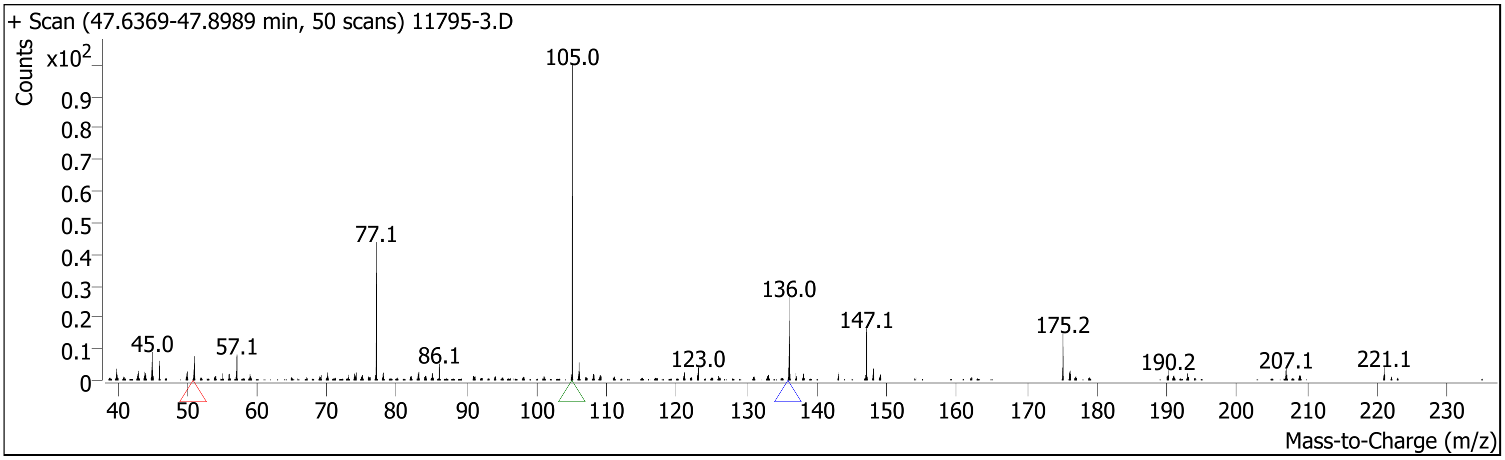

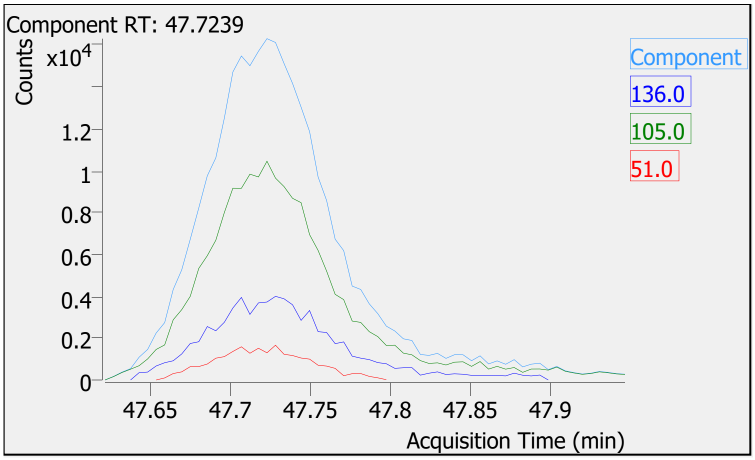

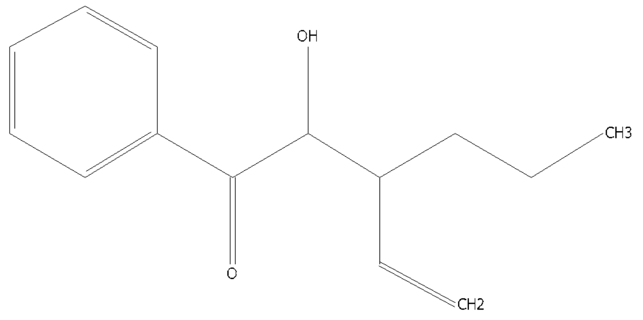

Unknown Analysis Report - Best Hits

RT	Compound Name	CAS#	Formula	Area	MI	Match Score	Sample	Sample
49.2887	(2S,3S)-3-Methyl-2-(1-phenylethyl)-3-(trifluoromethyl)-1,2-oxaziridine	990133-36-1	C11H12F3NO	15004		91.9	0.16	0.26

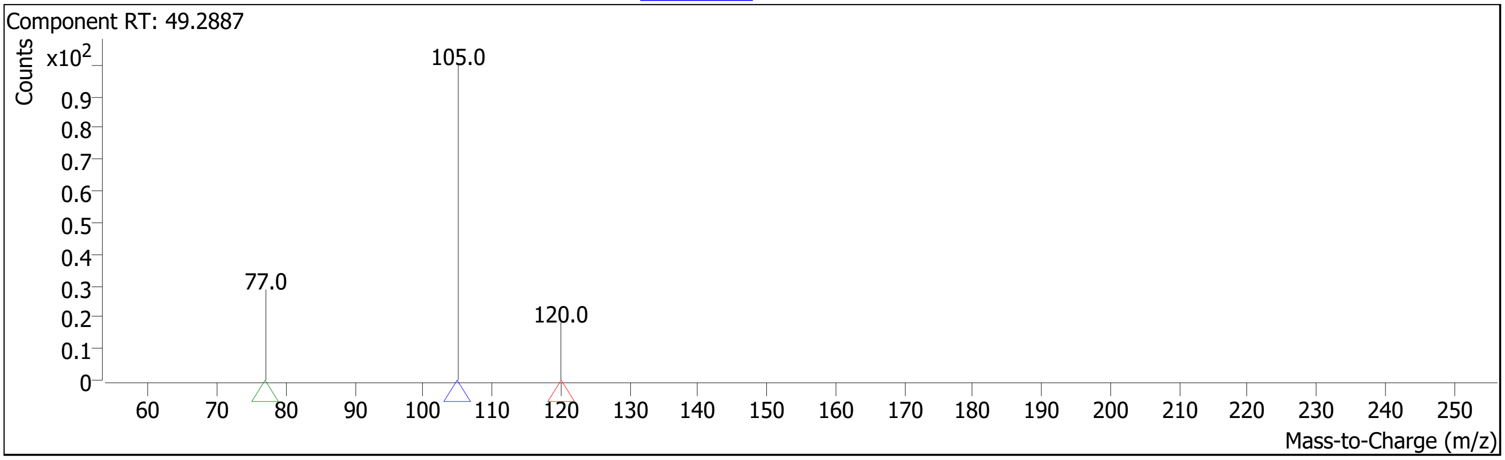

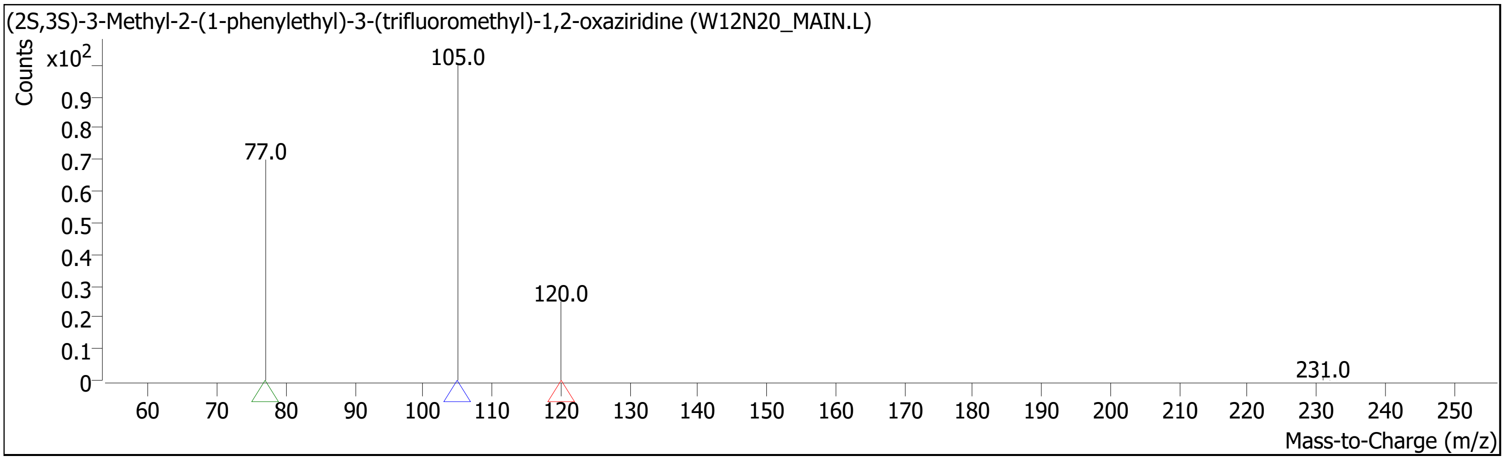

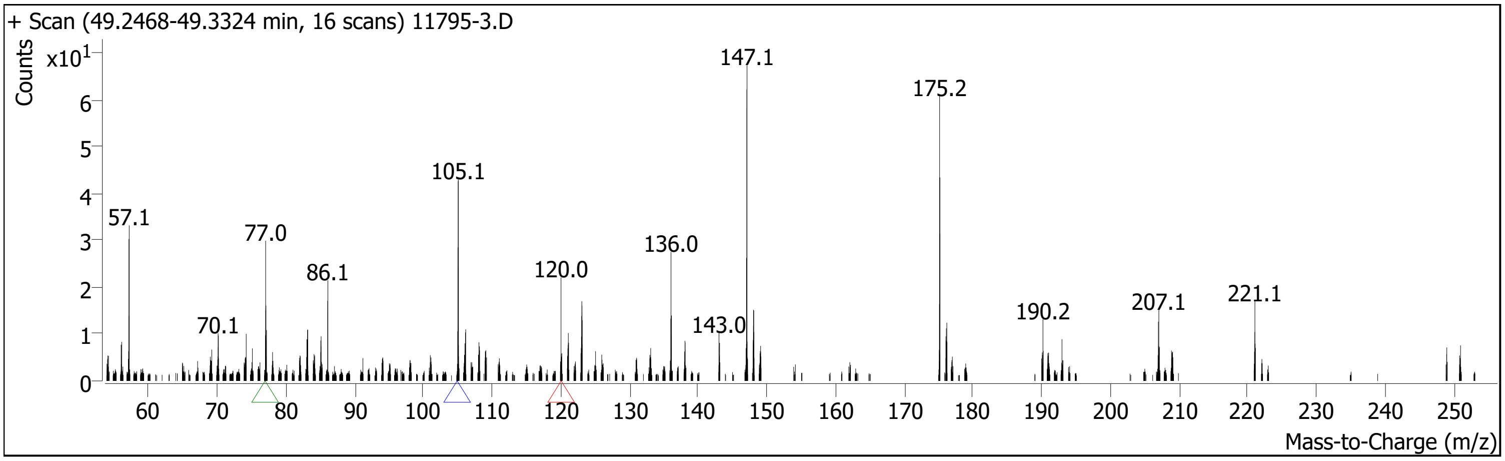

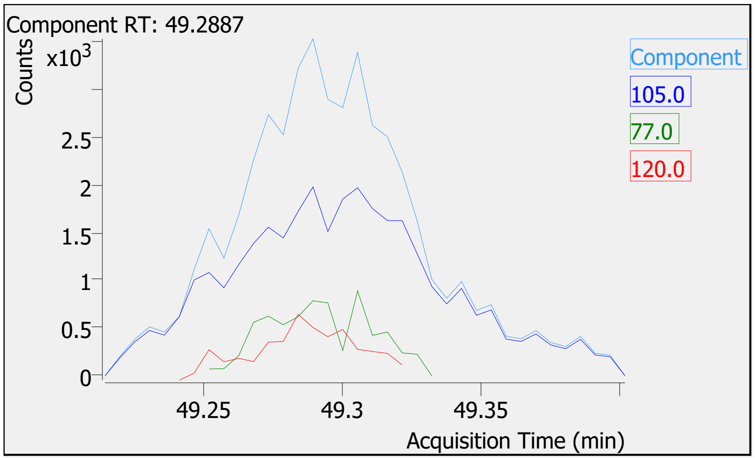

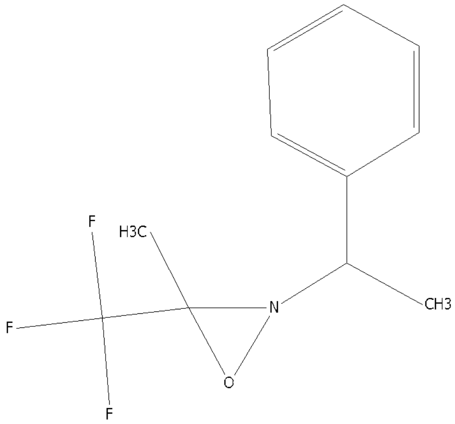

Unknown Analysis Report - Best Hits

RT	Compound Name	CAS#	Formula	Area	MI	Match Score	Sample	Sample
51.1335	Cyclooctasiloxane, hexadecamethyl-	556-68-3	C16H48O8Si8	102252		72.7	1.07	1.77

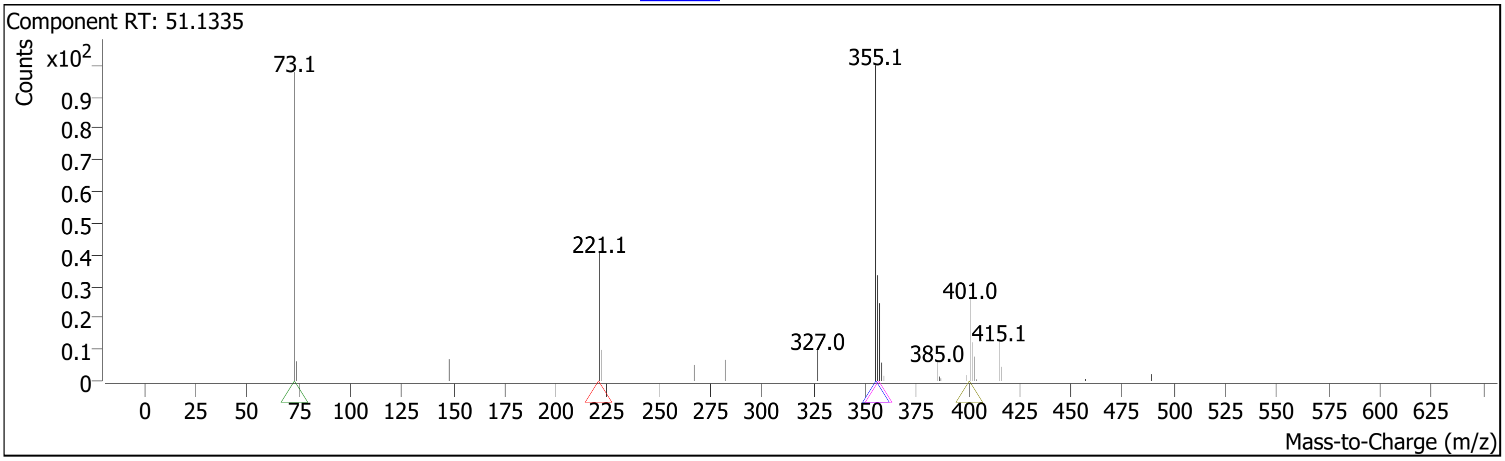

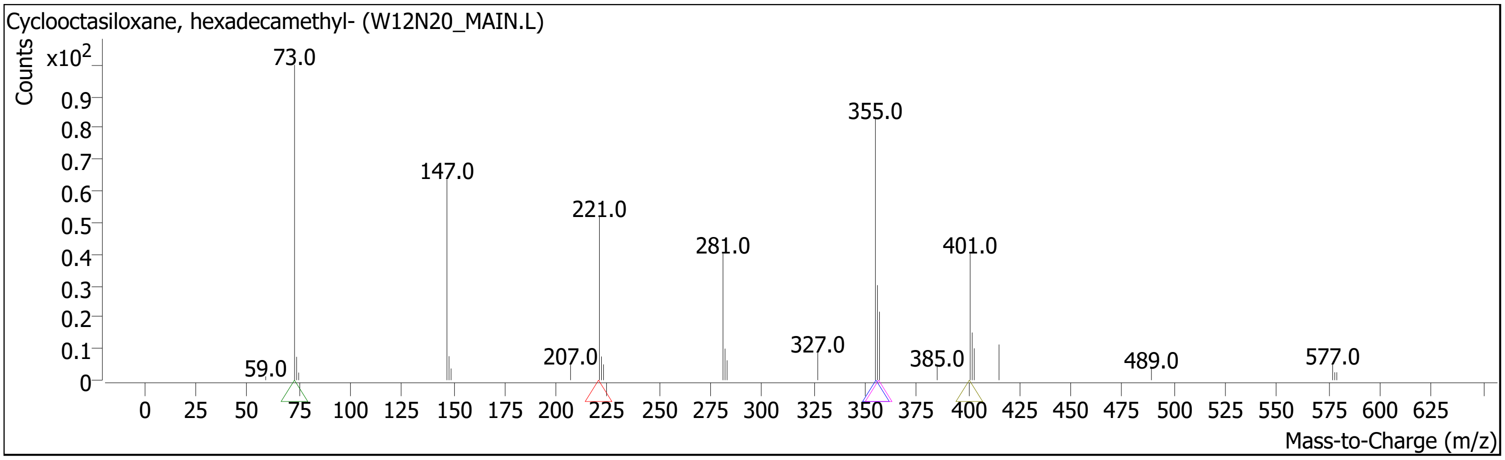

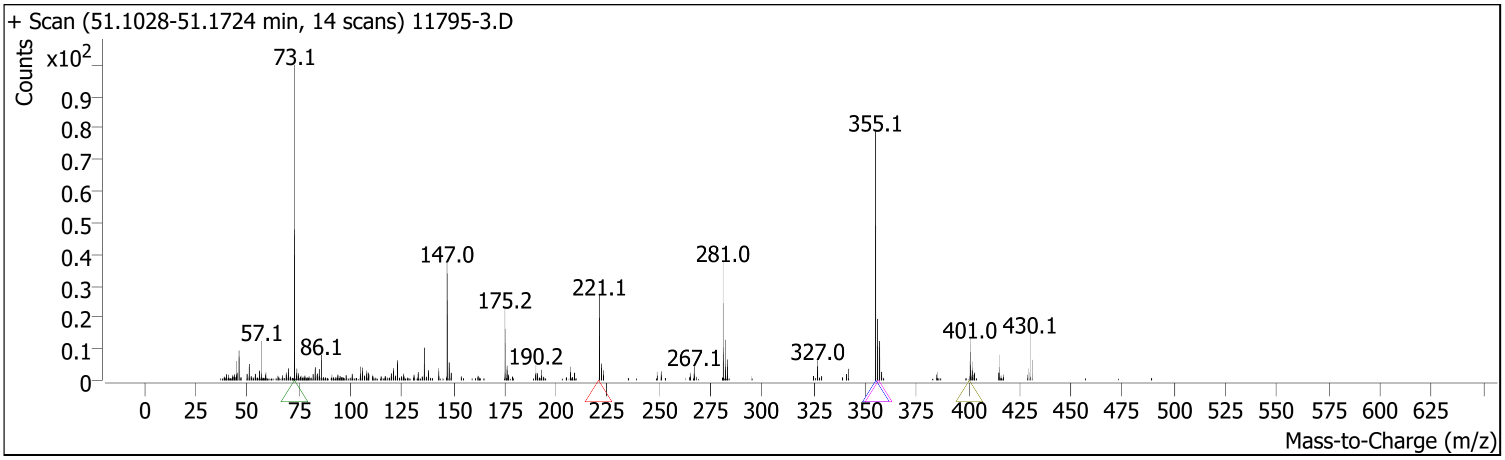

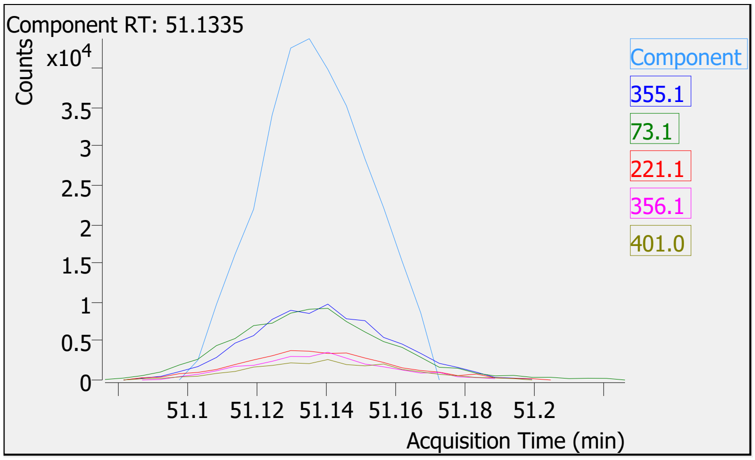

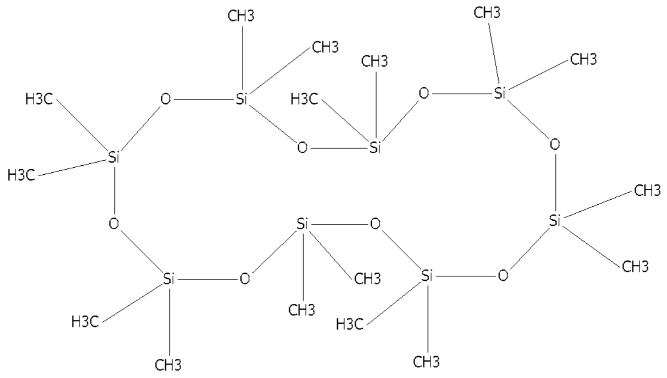

Unknown Analysis Report - Best Hits

RT	Compound Name	CAS#	Formula	Area	MI	Match Score	Sample	Sample
54.6656	Dimethyl ether	115-10-6	C2H6O	13474		87.9	0.14	0.23

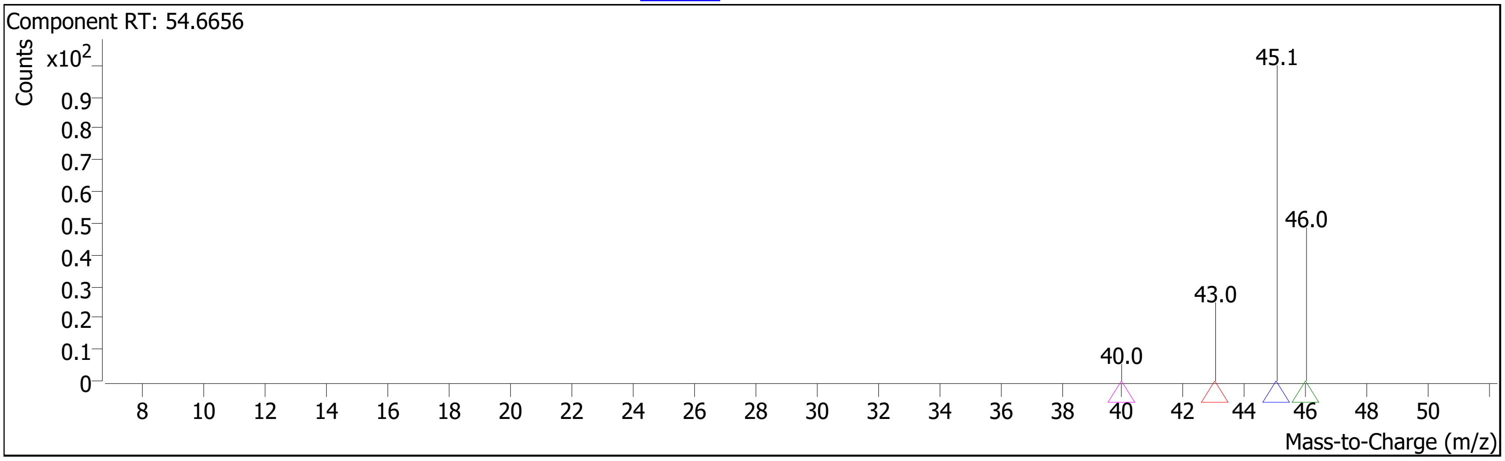

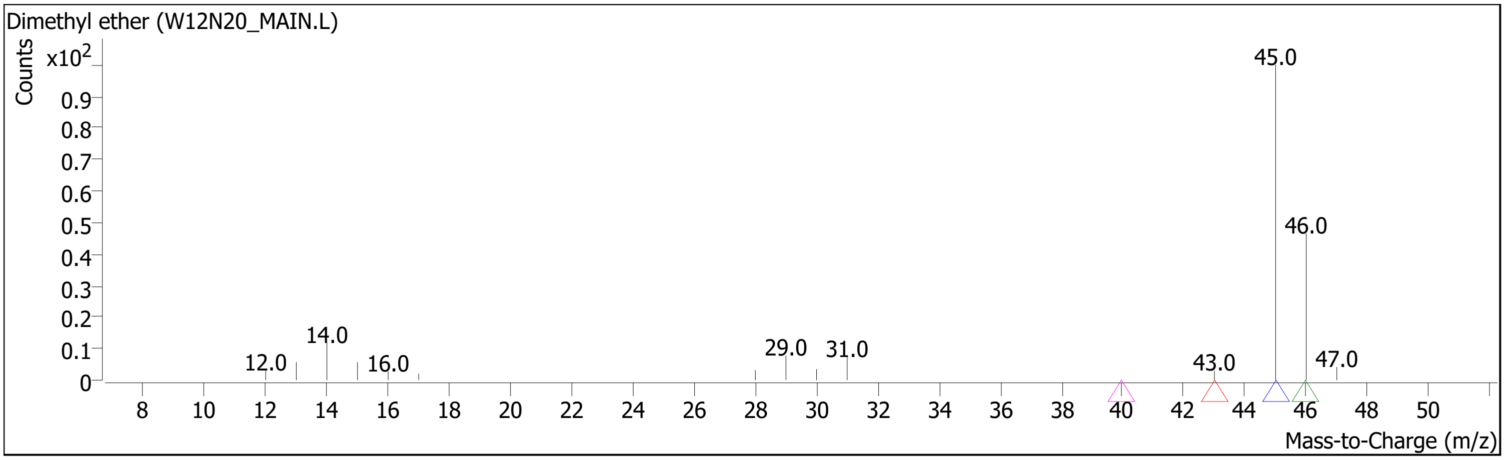

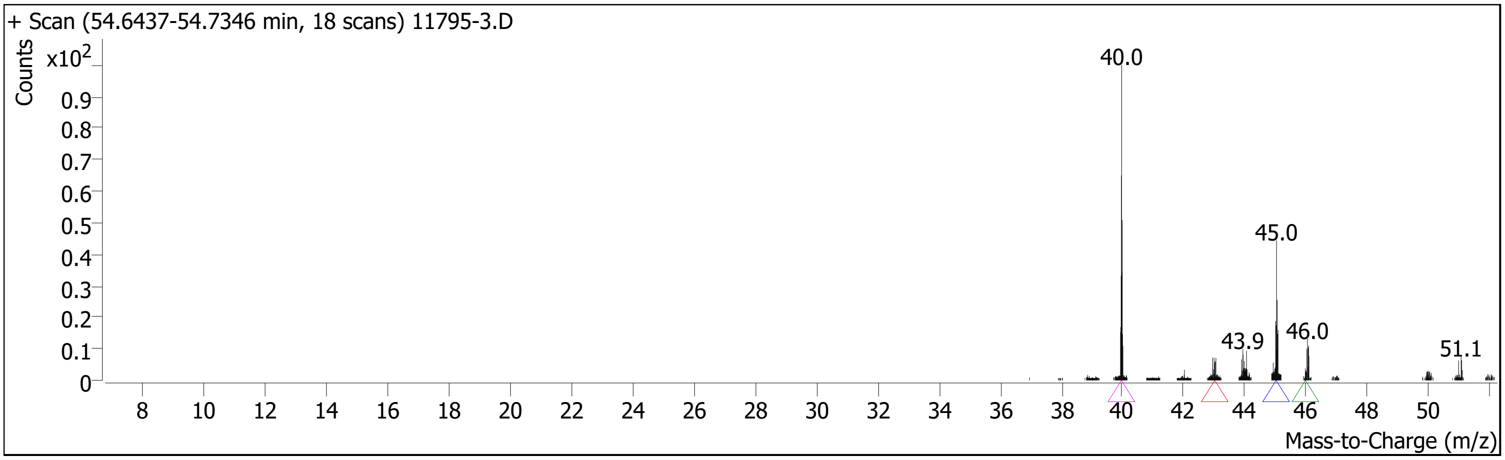

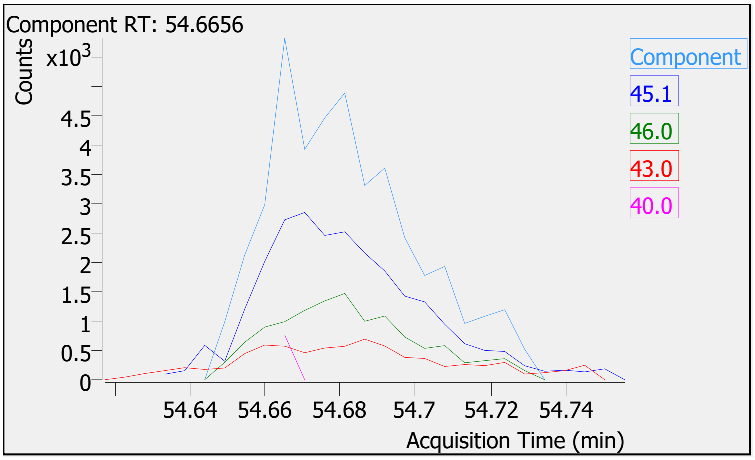

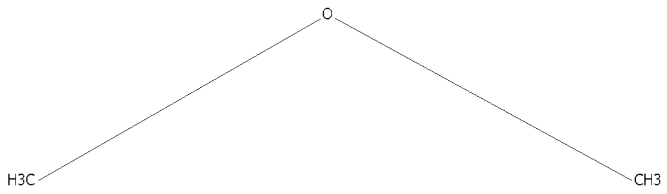

Unknown Analysis Report - Best Hits

RT	Compound Name	CAS#	Formula	Area	MI	Match Score	Sample	Sample
56.3621	2-Hexoxybenzoic acid methyl ester	56306-81-1	C14H20O3	14205		71.7	0.15	0.25

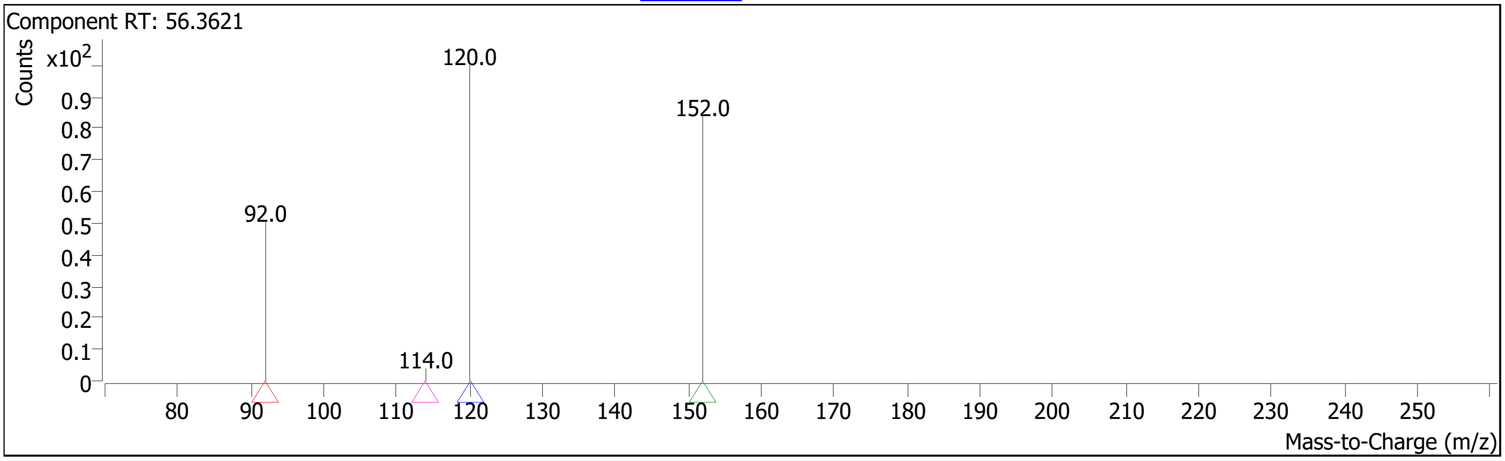

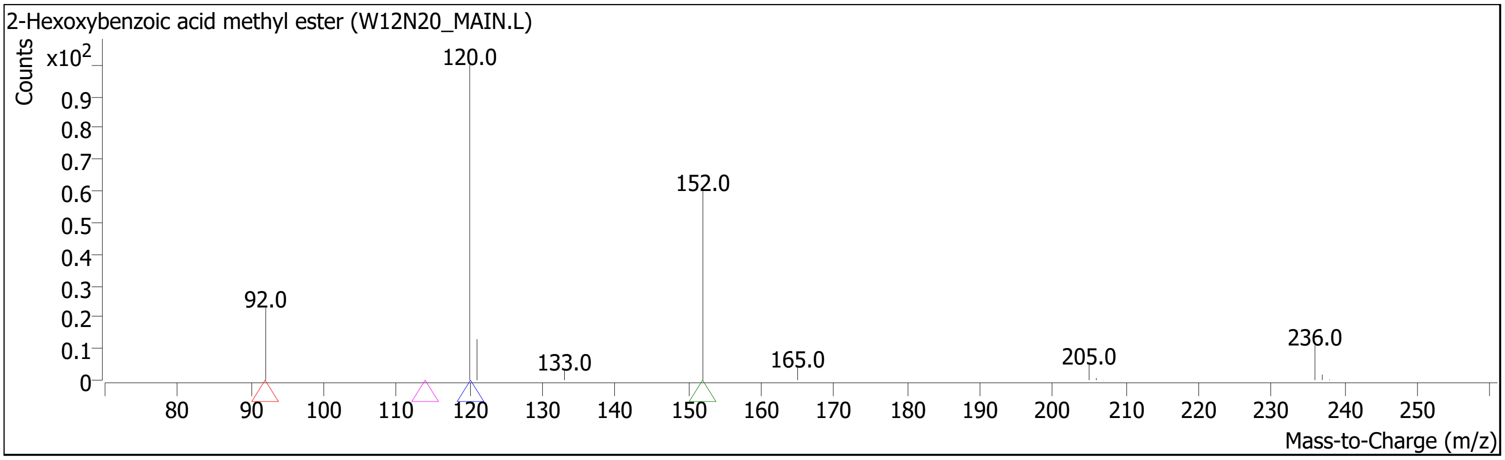

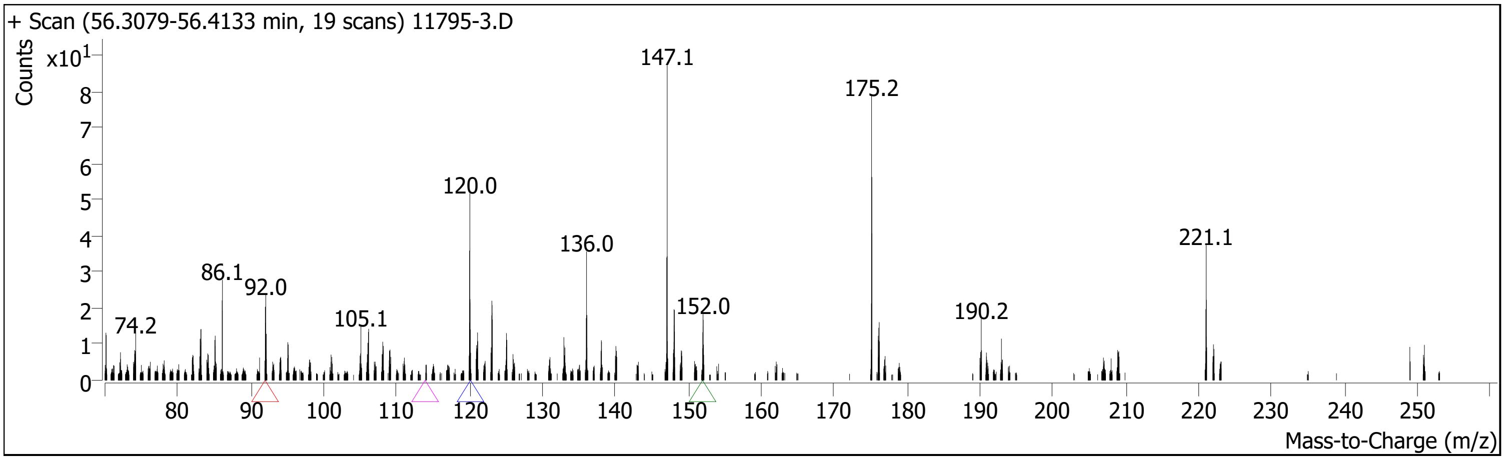

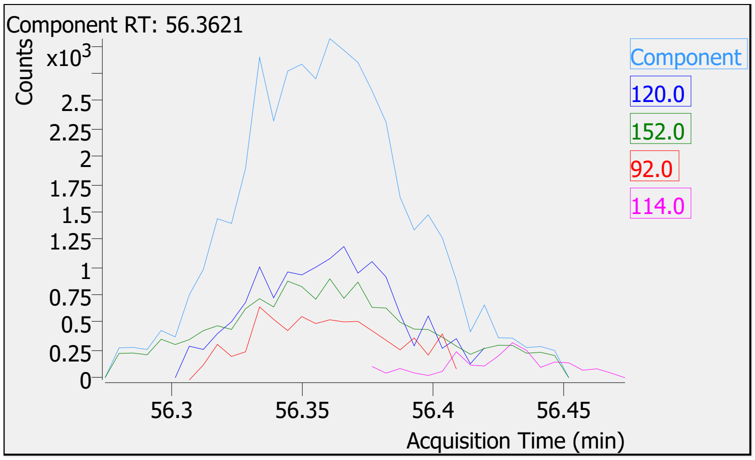

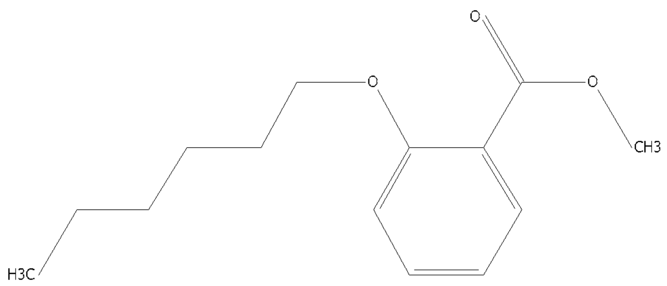

RT	Compound Name	CAS#	Formula	Area	MI	Match Score	Sample	Sample
61.7943	2,2-bis(fluoranyl)-1-phenyl-but-3-en-1-ol	85864-61-5	C10H10F2O	38511		89.3	0.40	0.66

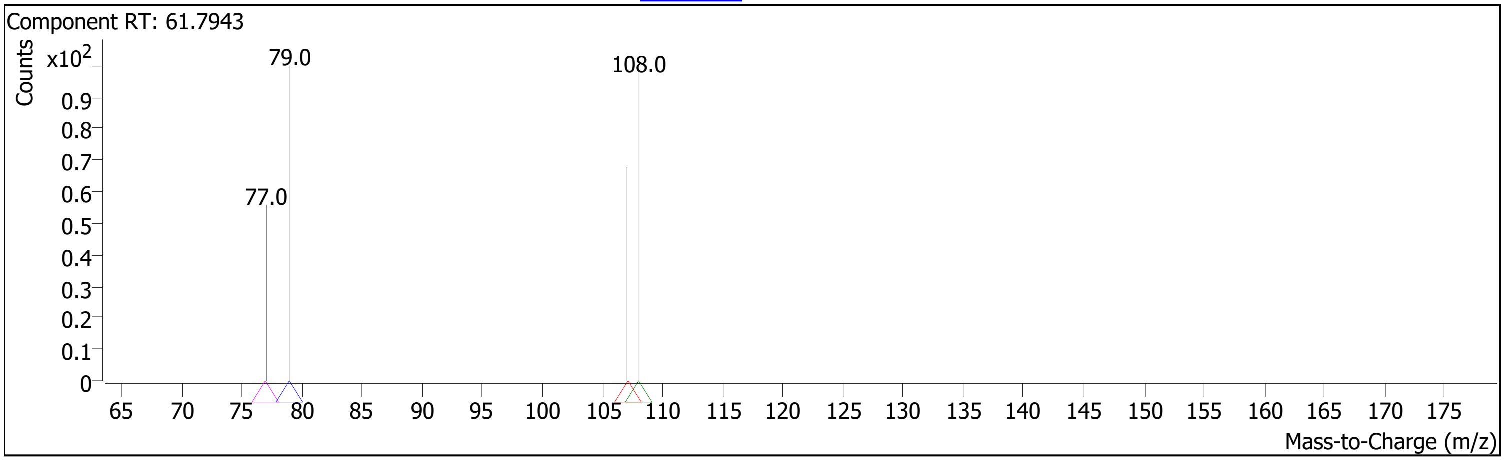

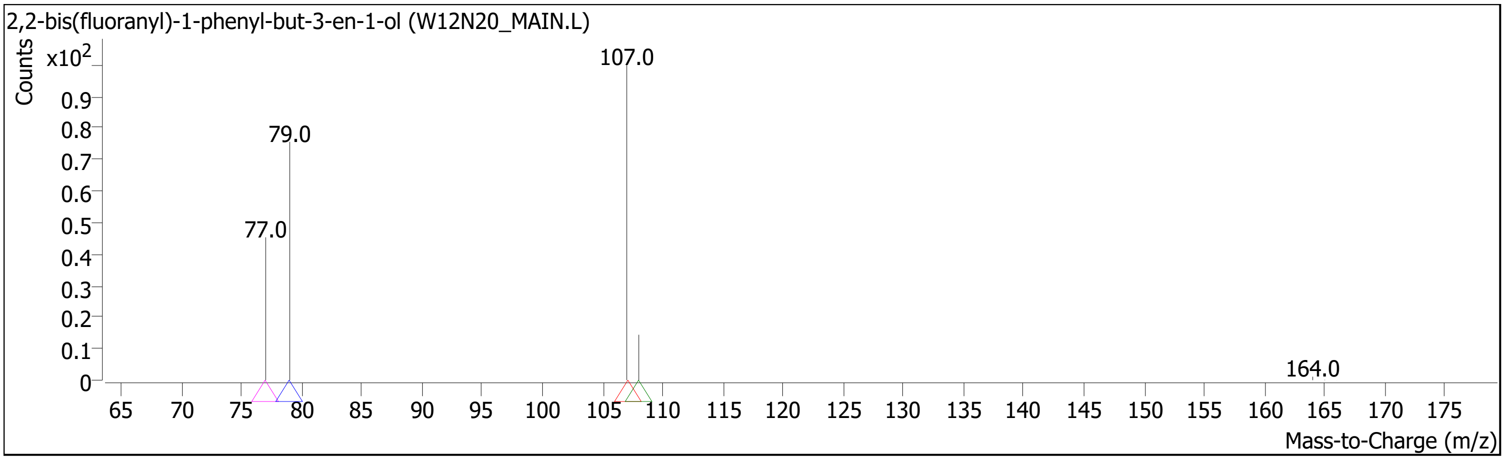

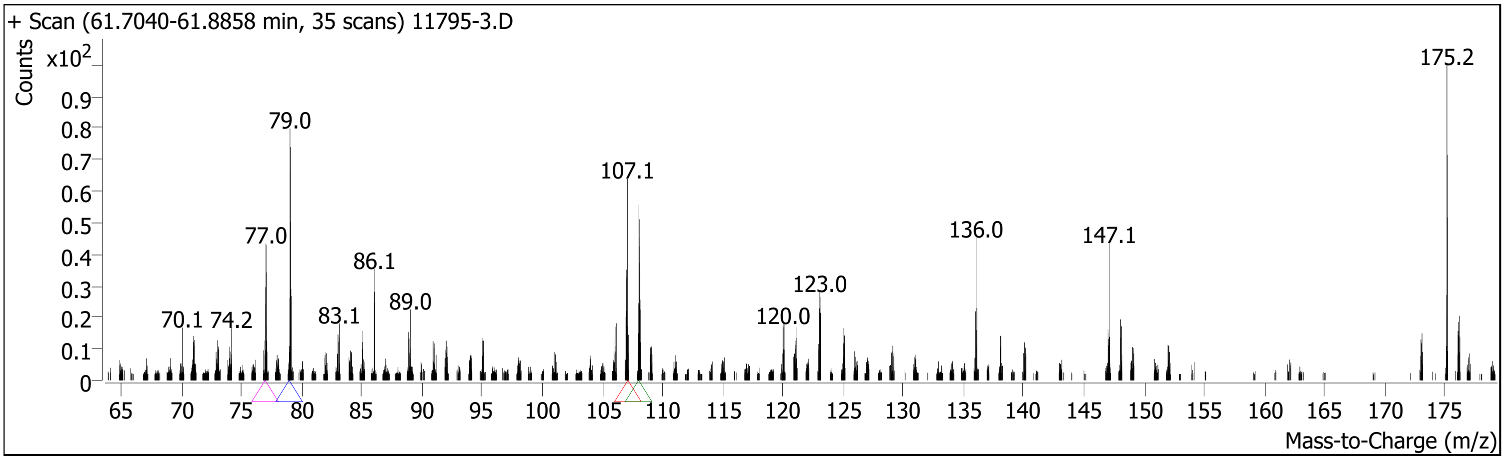

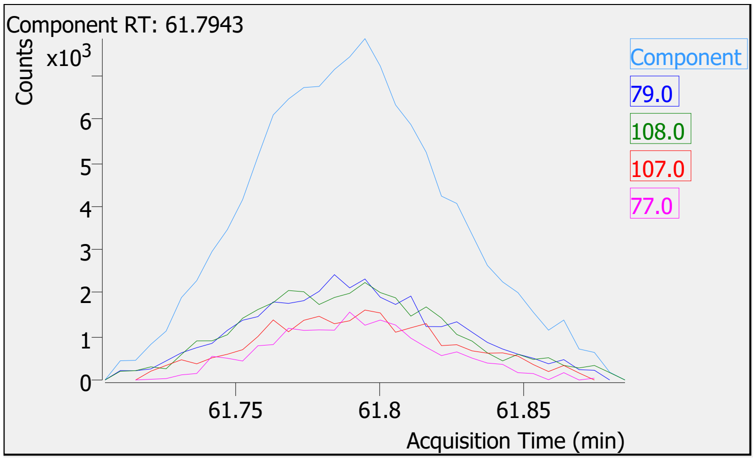

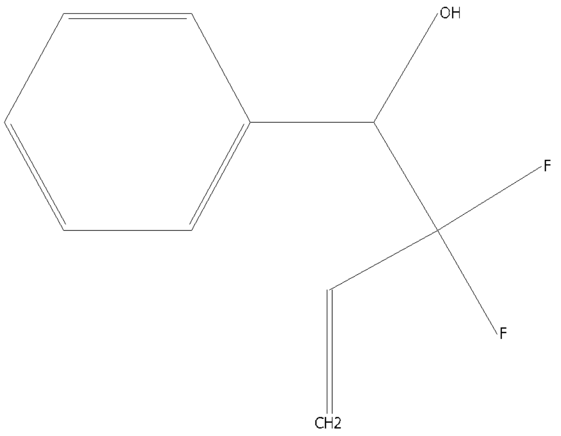

RT	Compound Name	CAS#	Formula	Area	MI	Match Score	Sample	Sample
62.2943	(S)-[1-(1-Allyloxyethyl)-1-vinylalloxymethyl]benzene	990198-79-9	C17H22O2	10490		71.3	0.11	0.18

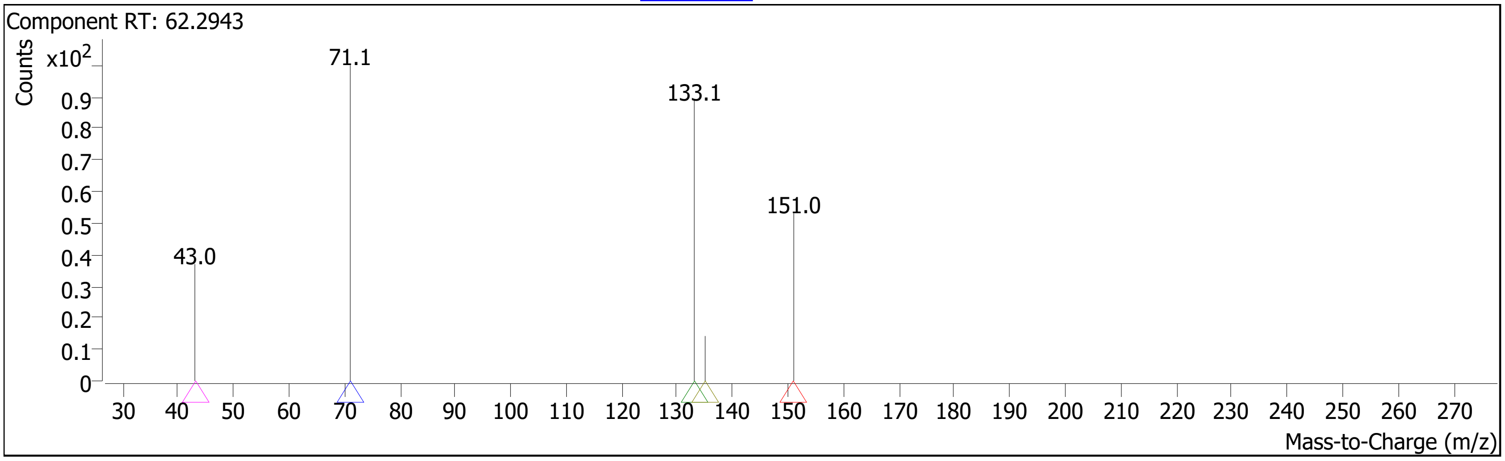

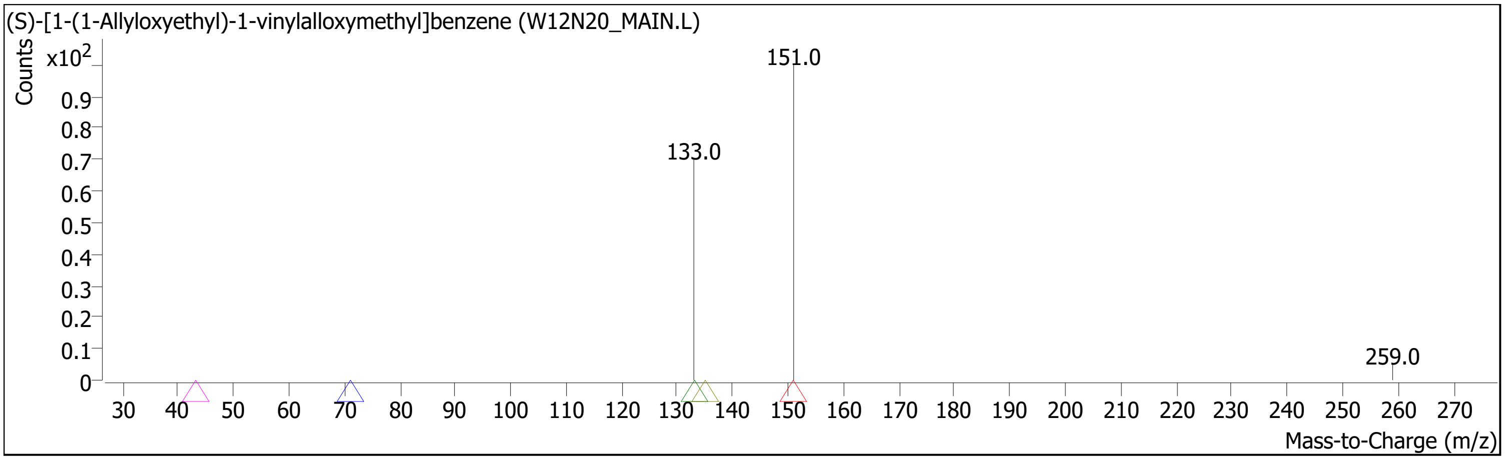

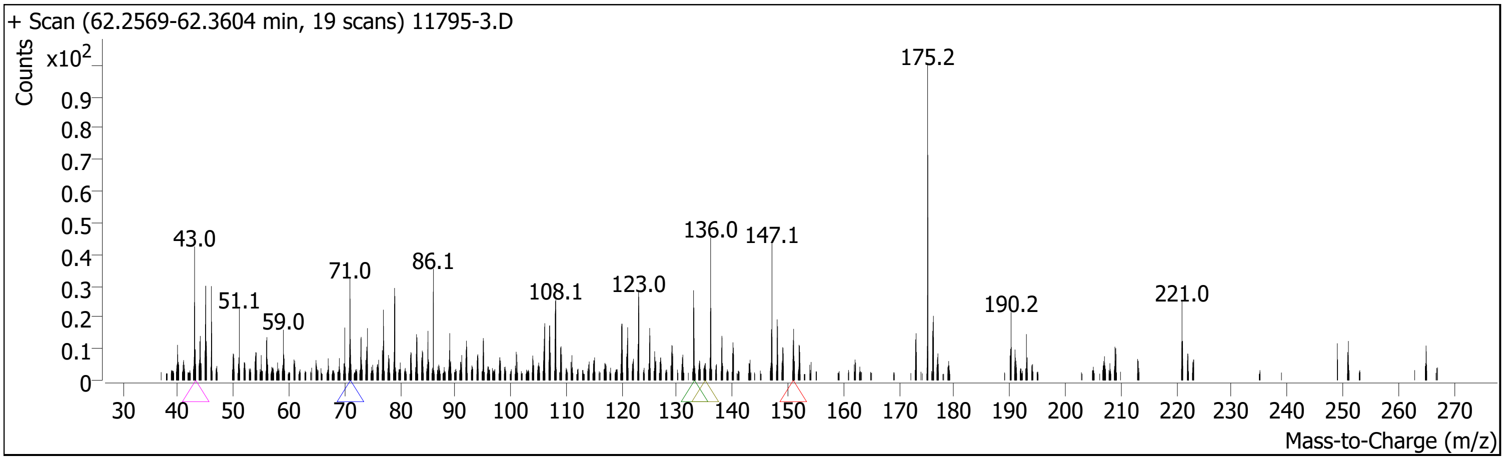

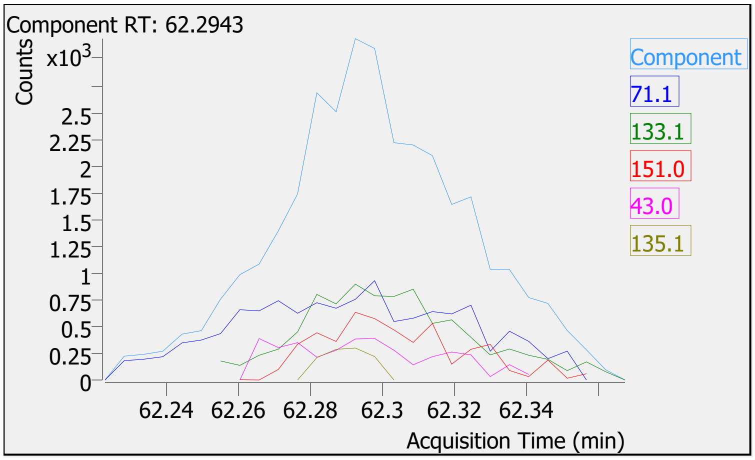

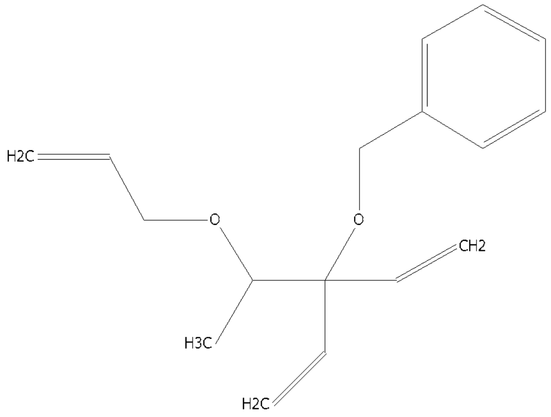

Unknown Analysis Report - Best Hits

RT	Compound Name	CAS#	Formula	Area	MI	Match Score	Sample	Sample
74.8721	trans-3,4-Dimethyl-2,3-epoxypentanal	990005-04-8	C7H12O2	54515		82.5	0.57	0.94

Component RT: 74.8721

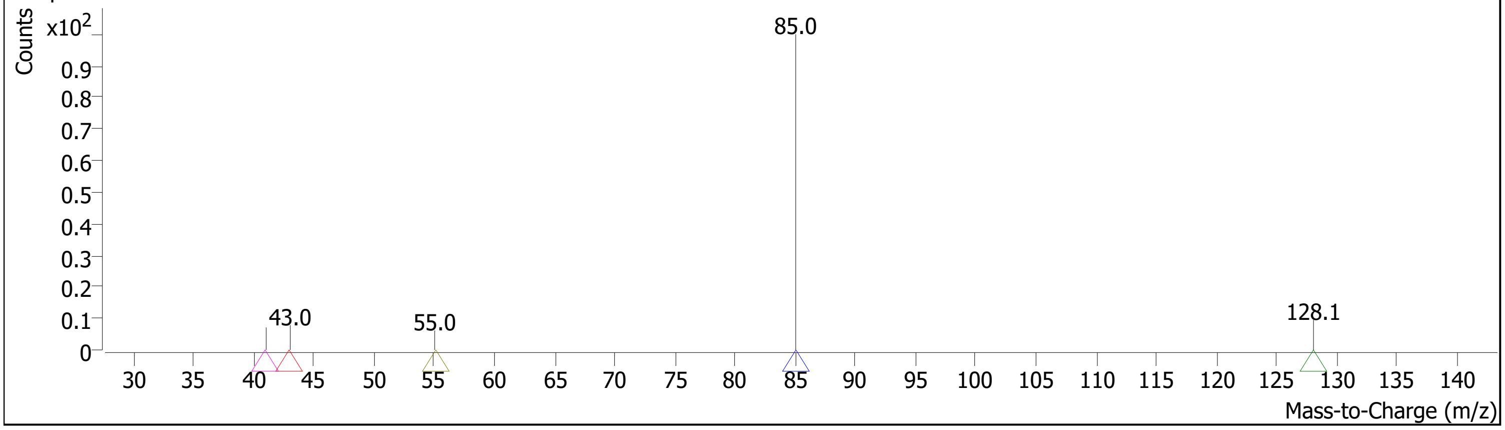

trans-3,4-Dimethyl-2,3-epoxypentanal (W12N20_MAIN.L)

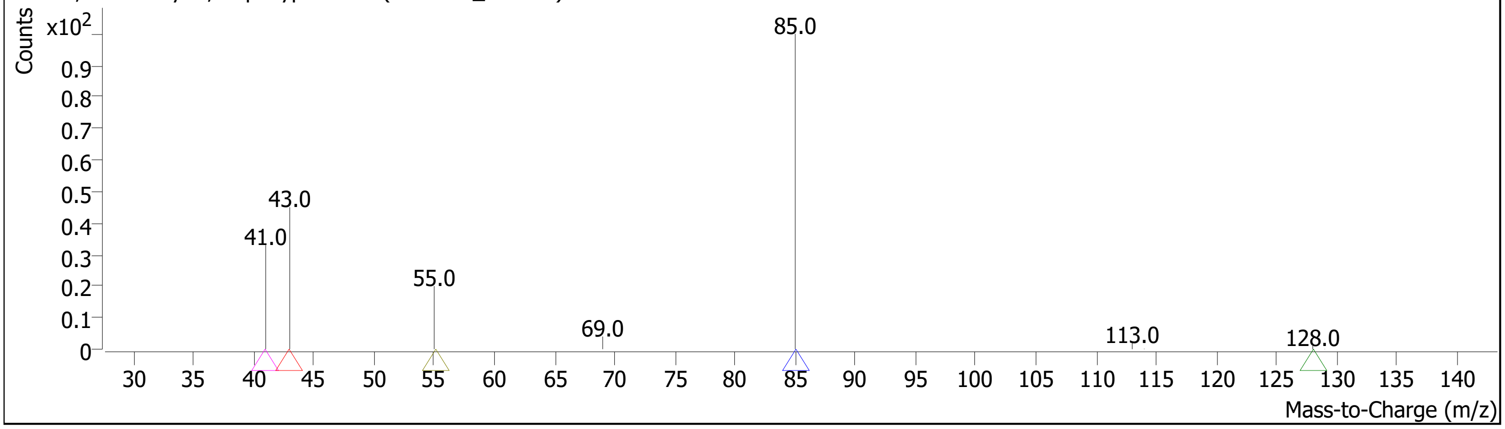

+ Scan (74.7708-74.9848 min, 41 scans) 11795-3.D

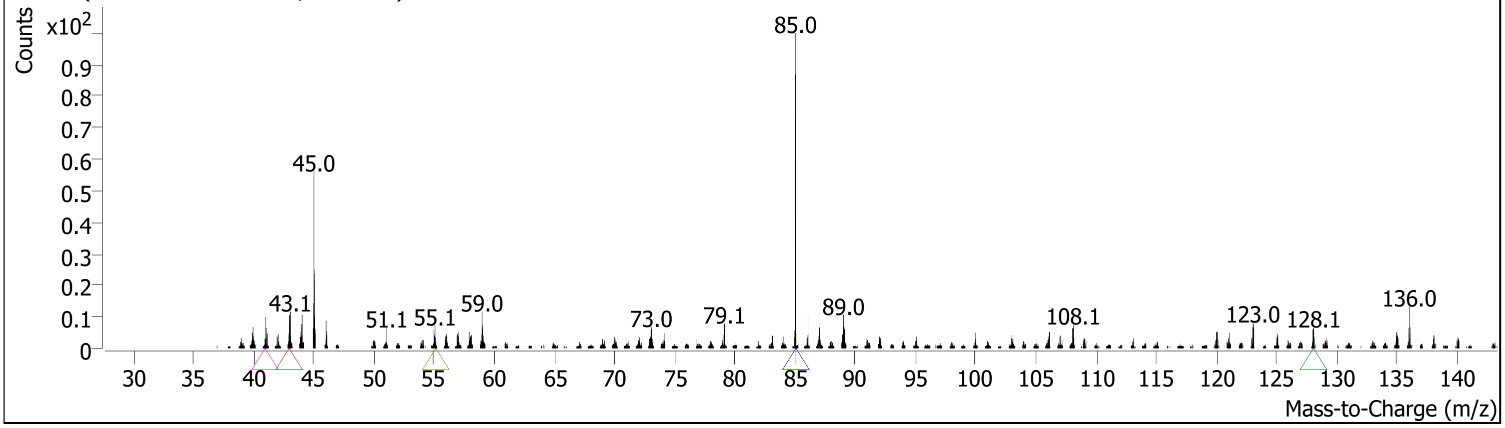

Component RT: 74.8721

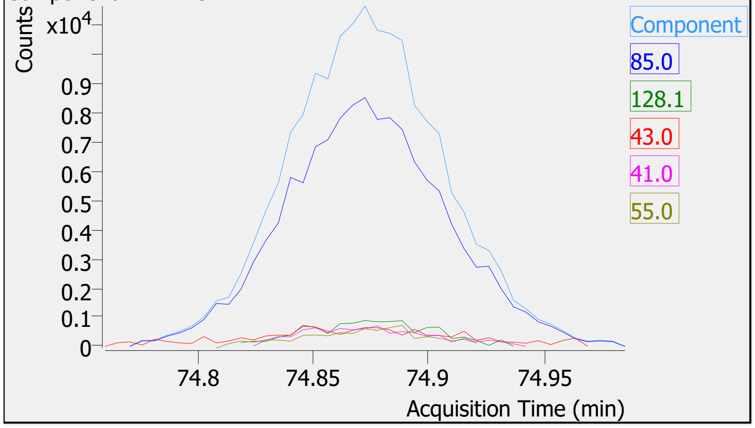

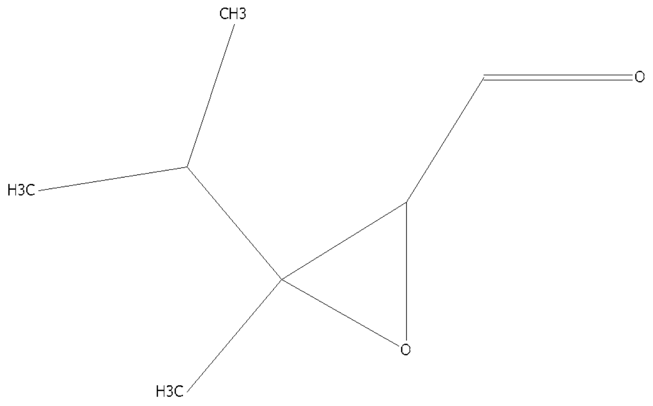

RT	Compound Name	CAS#	Formula	Area	MI	Match Score	Sample	Sample
80.1095	2(3H)-Furanone, 5-heptyldihydro-	104-67-6	C11H20O2	578167		89.7	6.08	9.98

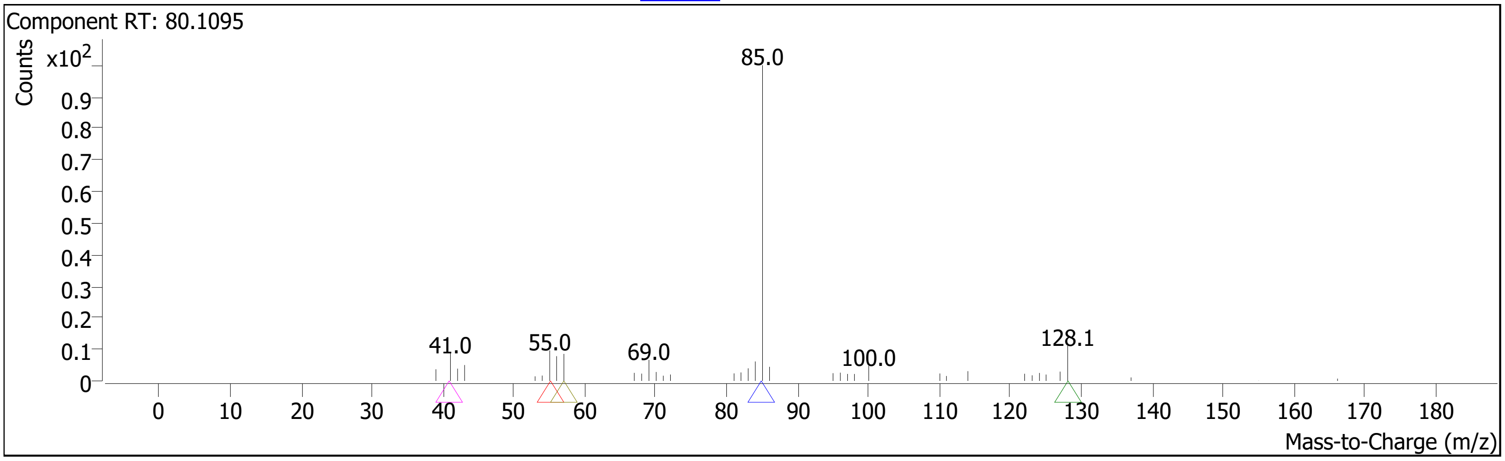

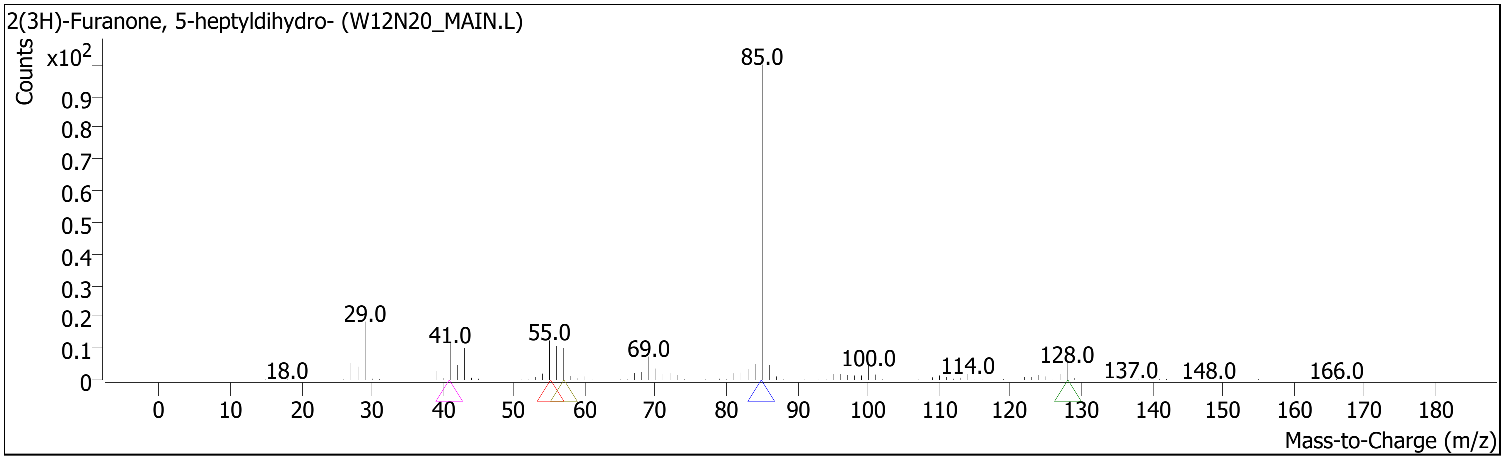

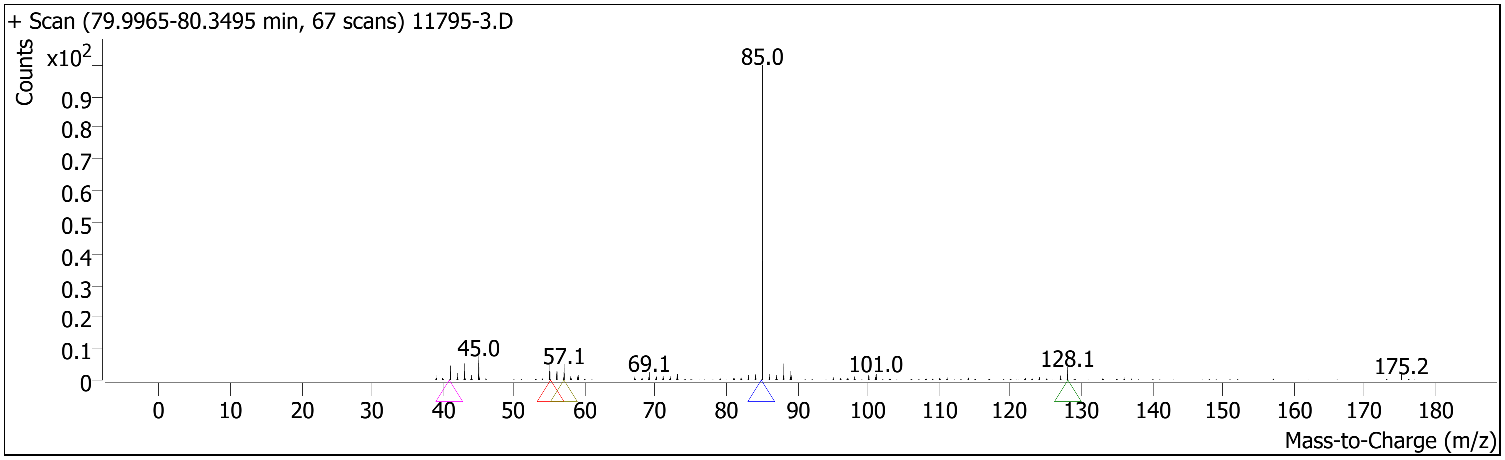

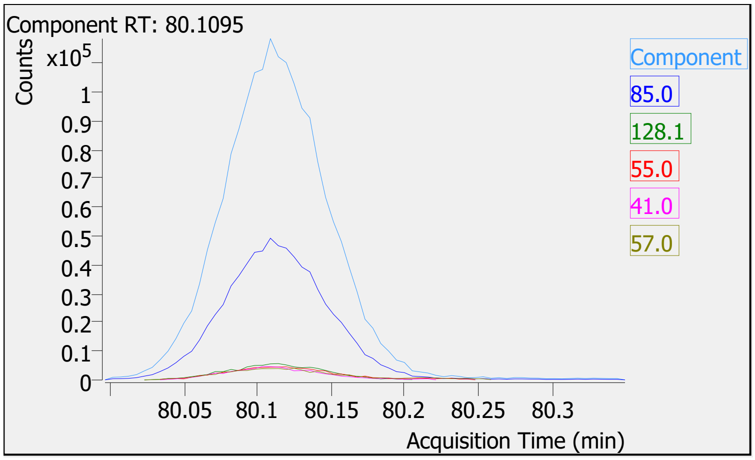

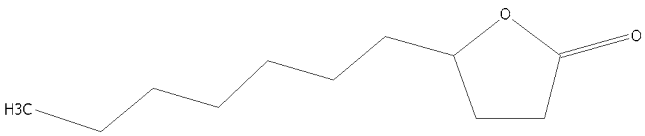

 Generated at 4:34 PM on 11/14/2023

Unknown Analysis Report - Best Hits

RT	Compound Name	CAS#	Formula	Area	MI	Match Score	Sample	Sample
80.2103	3-Acetyloxypropyl 2,3,4,6-tetra-O-methyl-.alpha.,L-(5-D)gulopyranoside	990391-50-2	C15H27DO8	41421		70.3	0.44	0.72

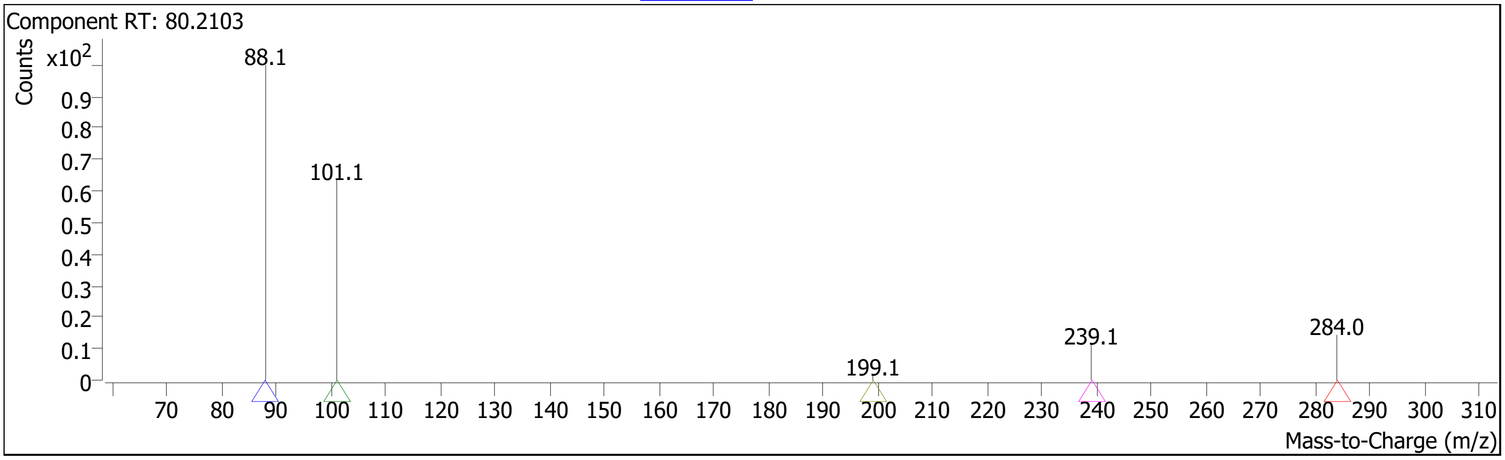

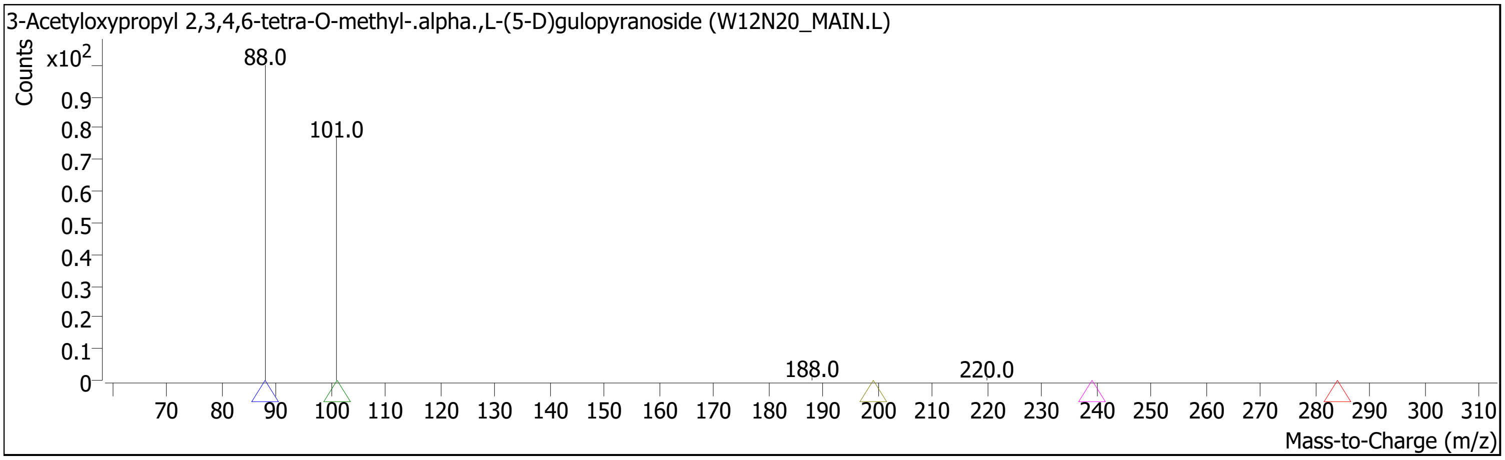

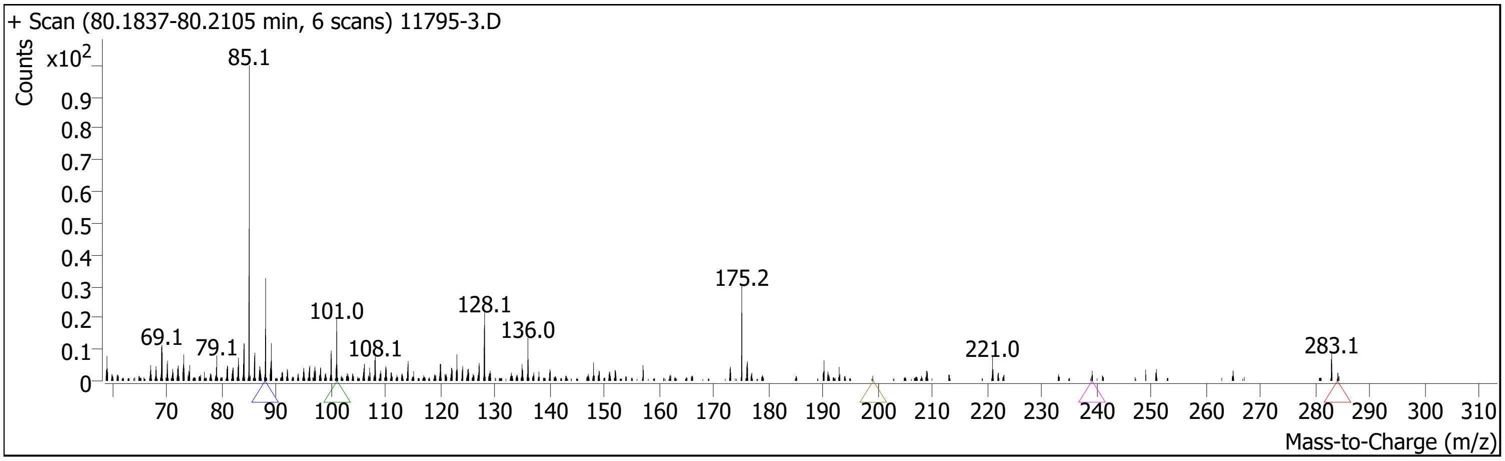

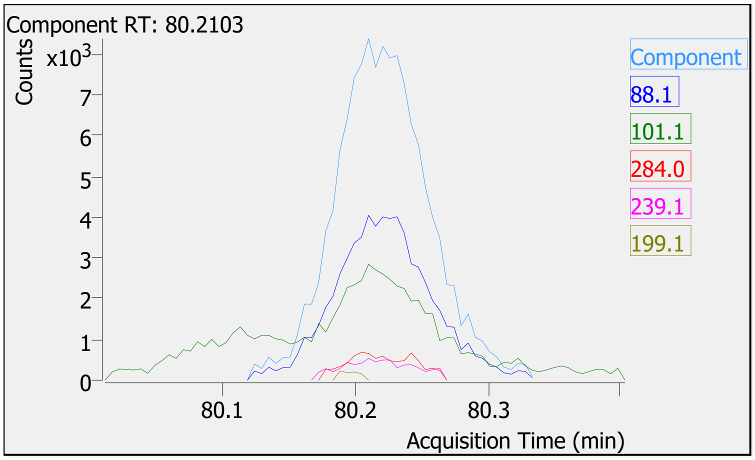

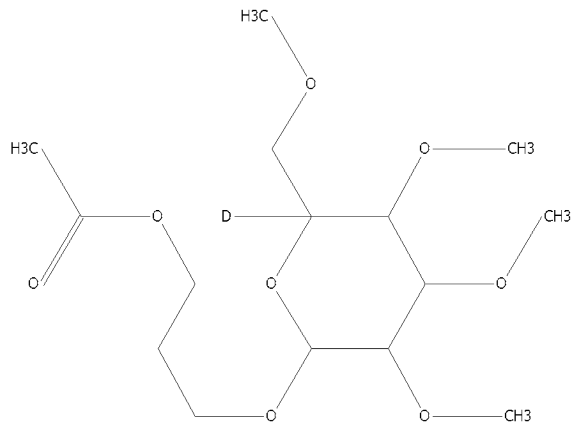

 Generated at 4:34 PM on 11/14/2023

Unknown Analysis Report - Best Hits

RT	Compound Name	CAS#	Formula	Area	MI	Match Score	Sample	Sample
82.5324	Methyl 5-[2-(4-Bromophenyl)-2-chloroethyl]-4,6-dimethylsalicylate	990507-75-8	C18H18BrClO3	60098		70.2	0.63	1.04

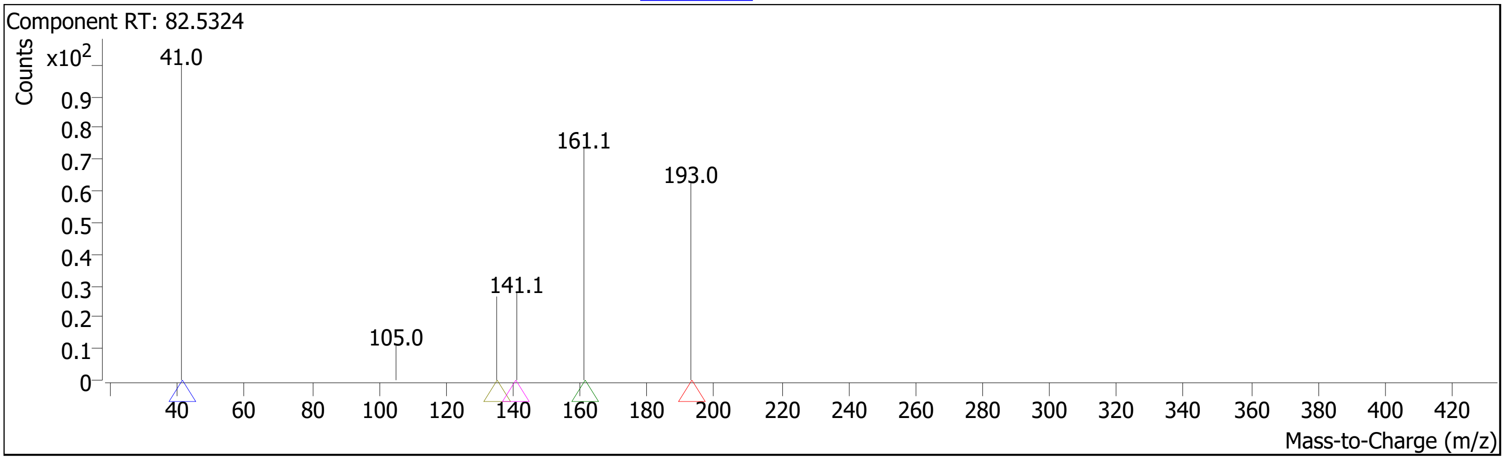

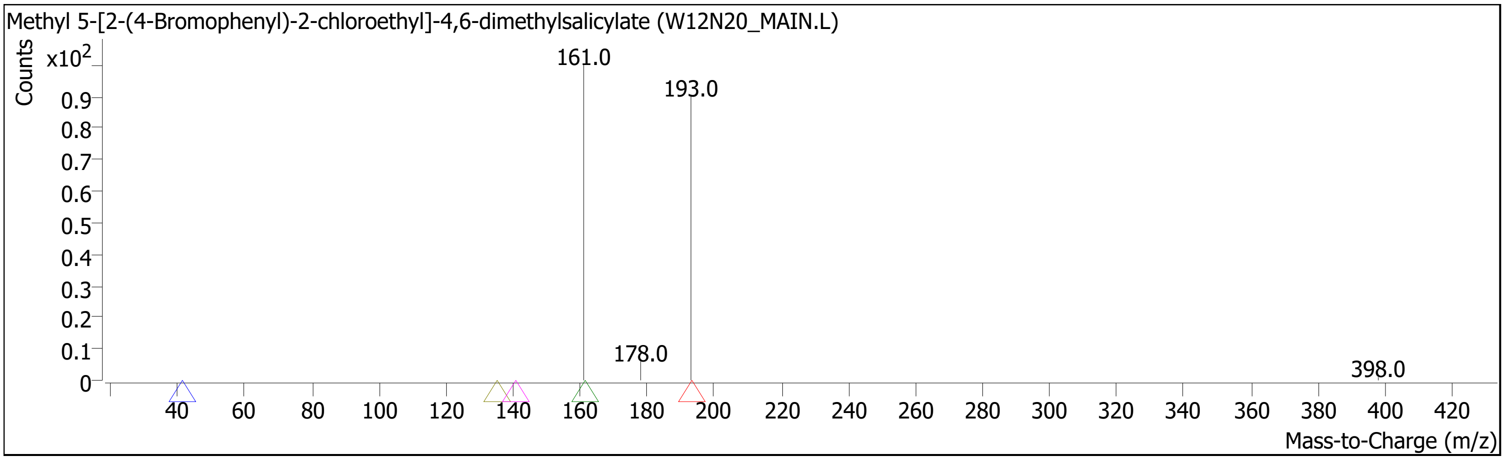

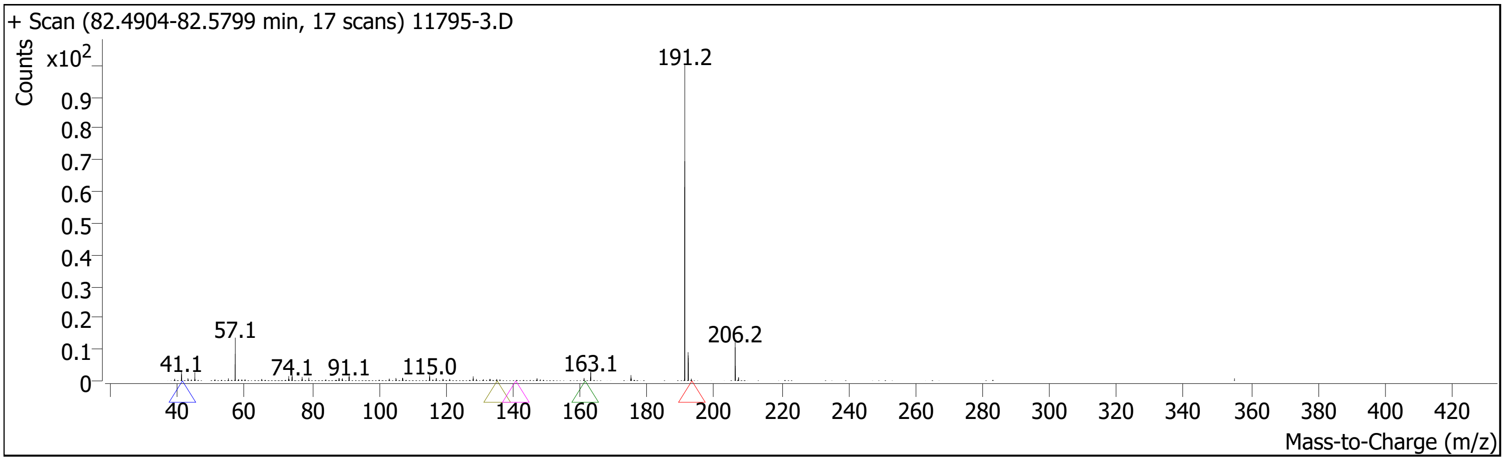

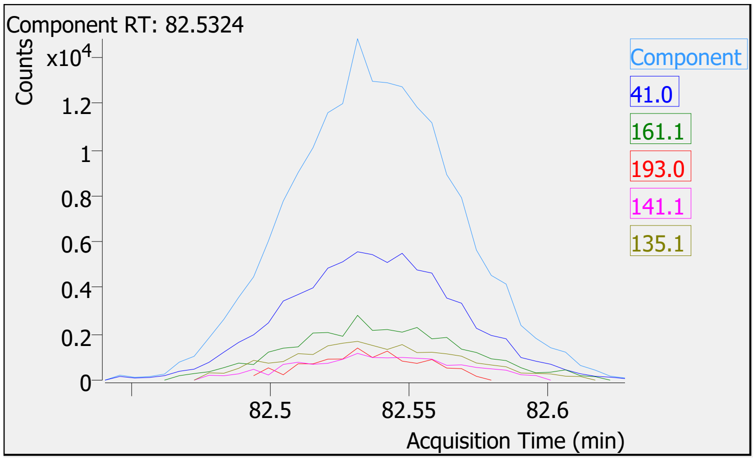

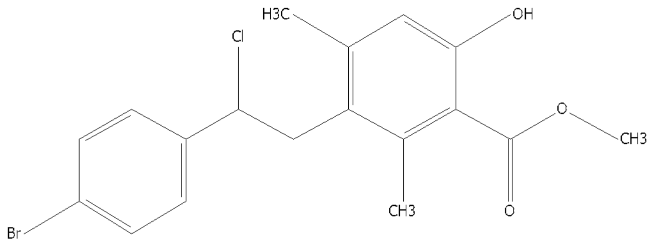

Generated at 4:34 PM on 11/14/2023

RT	Compound Name	CAS#	Formula	Area	MI	Match Score	Sample	Sample
82.5379	6,7-Dimethoxy-1-[N-(1-phenylethyl)amido]-1,2,3,4-tetrahydroisoquinoline isomer	990402-02-5	C20H24N2O3	217579		74.5	2.29	3.76

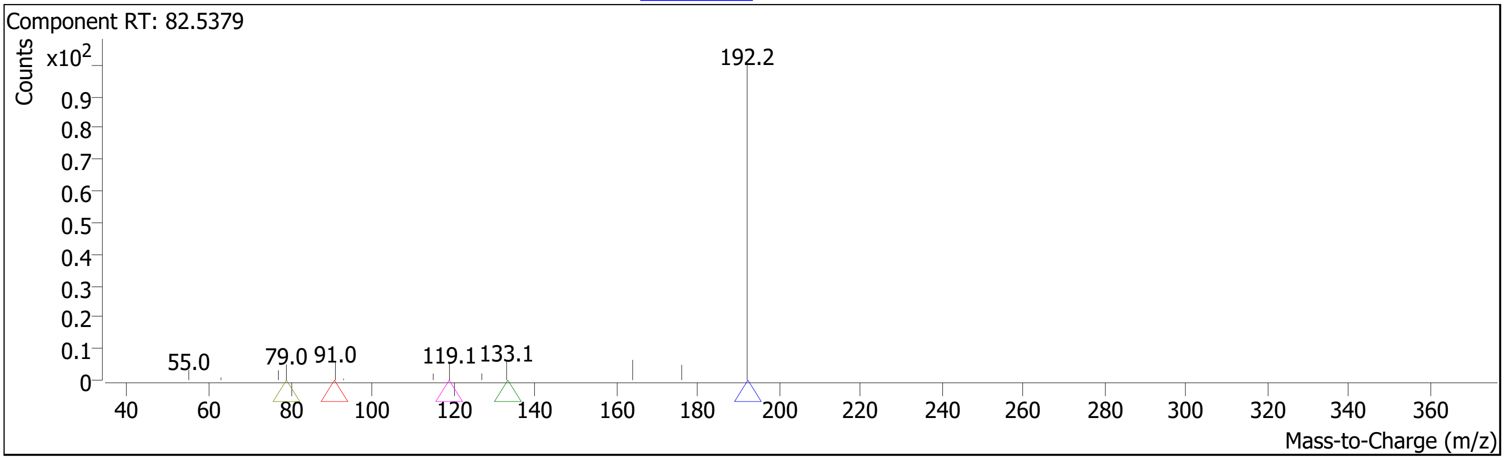

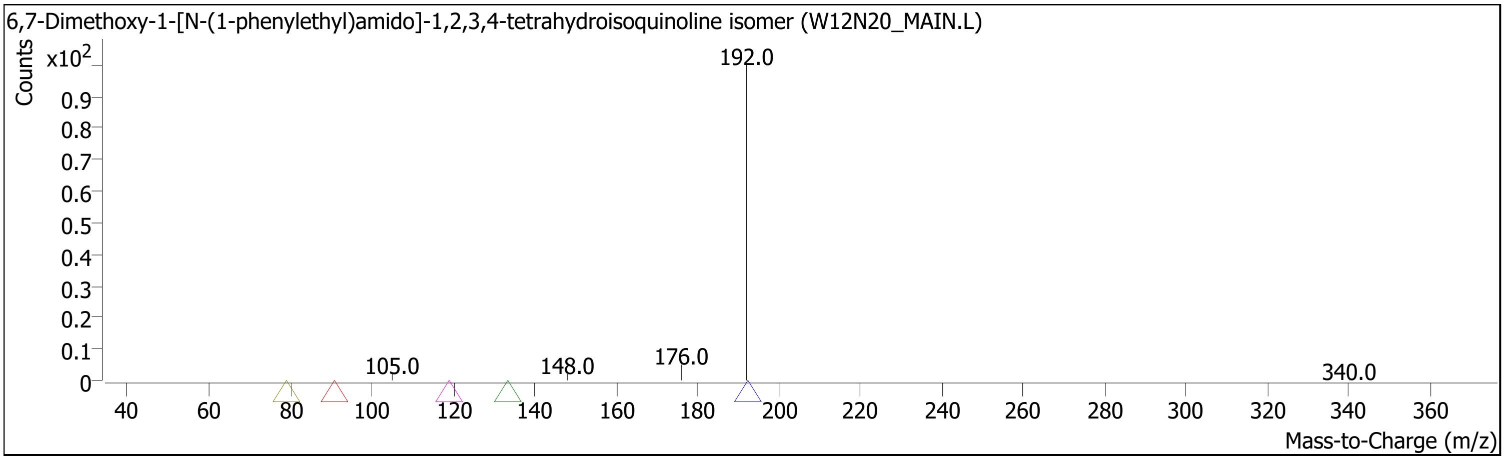

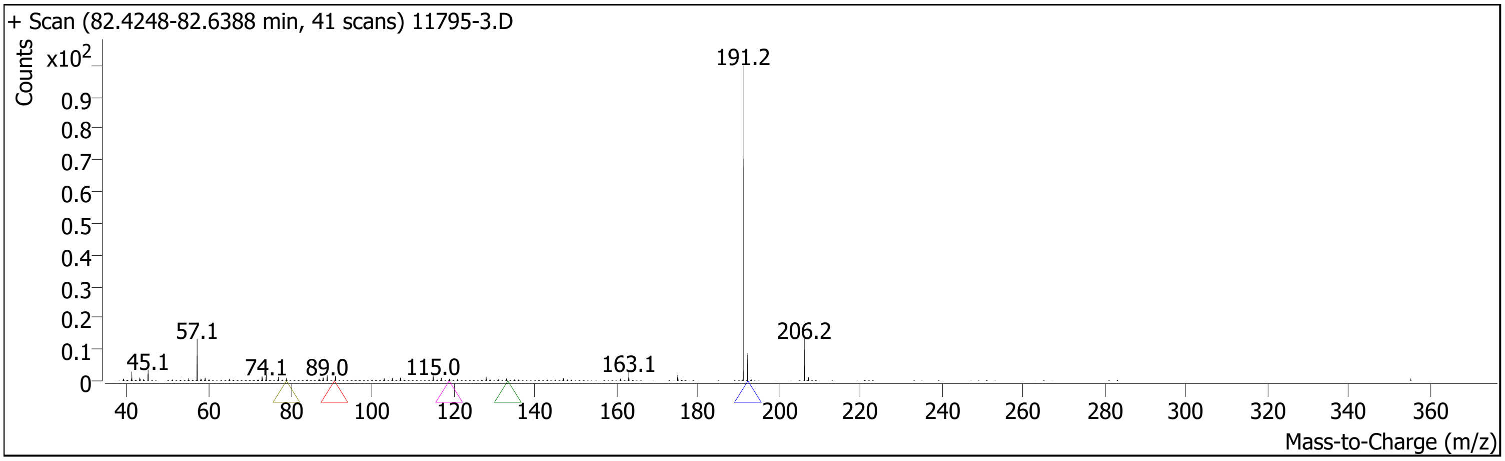

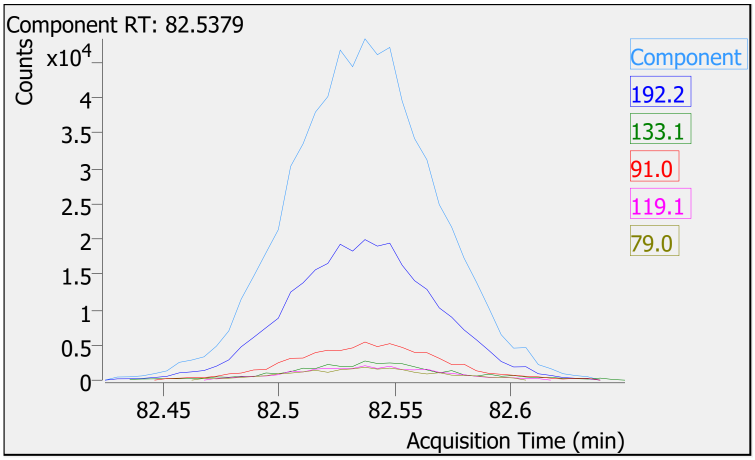

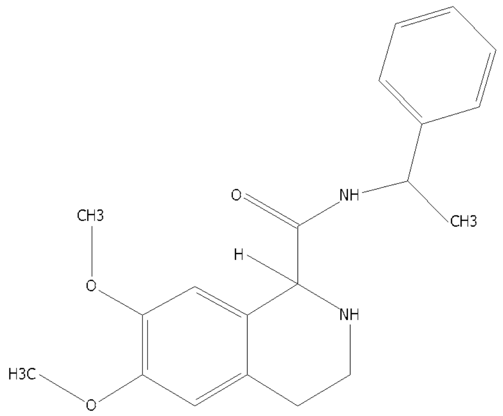

Unknown Analysis Report - Best Hits

RT	Compound Name	CAS#	Formula	Area	MI	Match Score	Sample	Sample
82.5407	Phenol, 2,4-bis(1,1-dimethylethyl)-	96-76-4	C14H22O	1317730		91.8	13.85	22.75

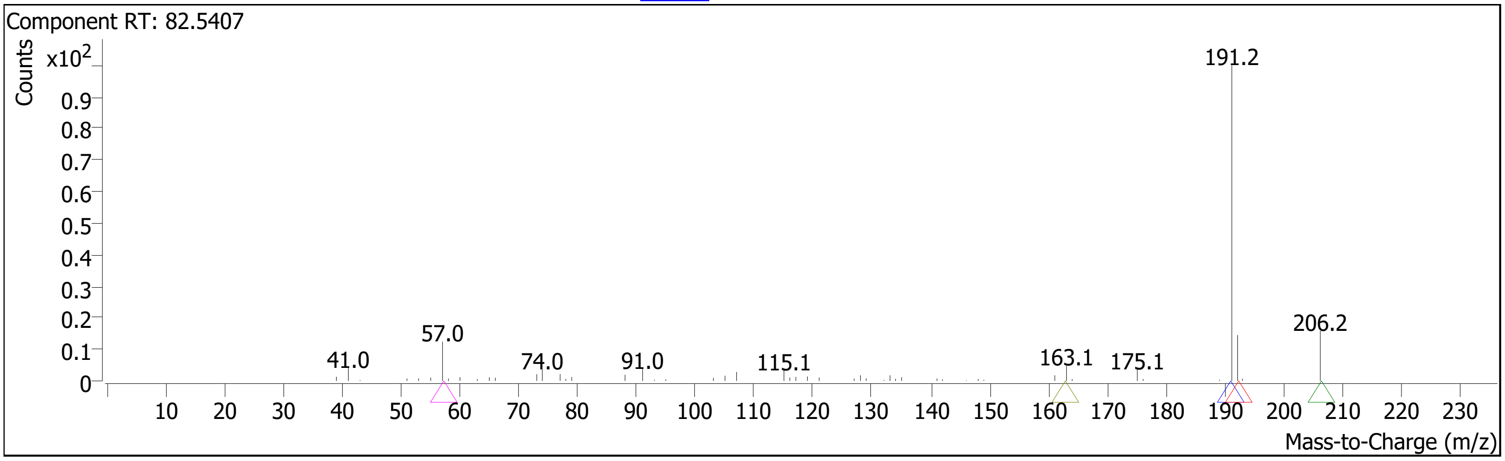

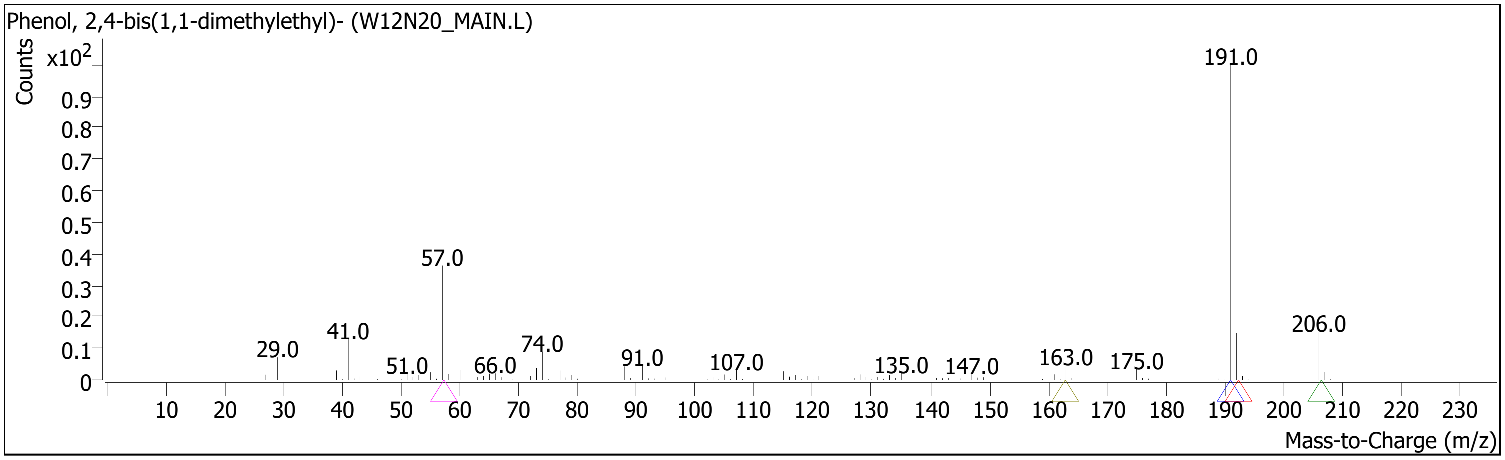

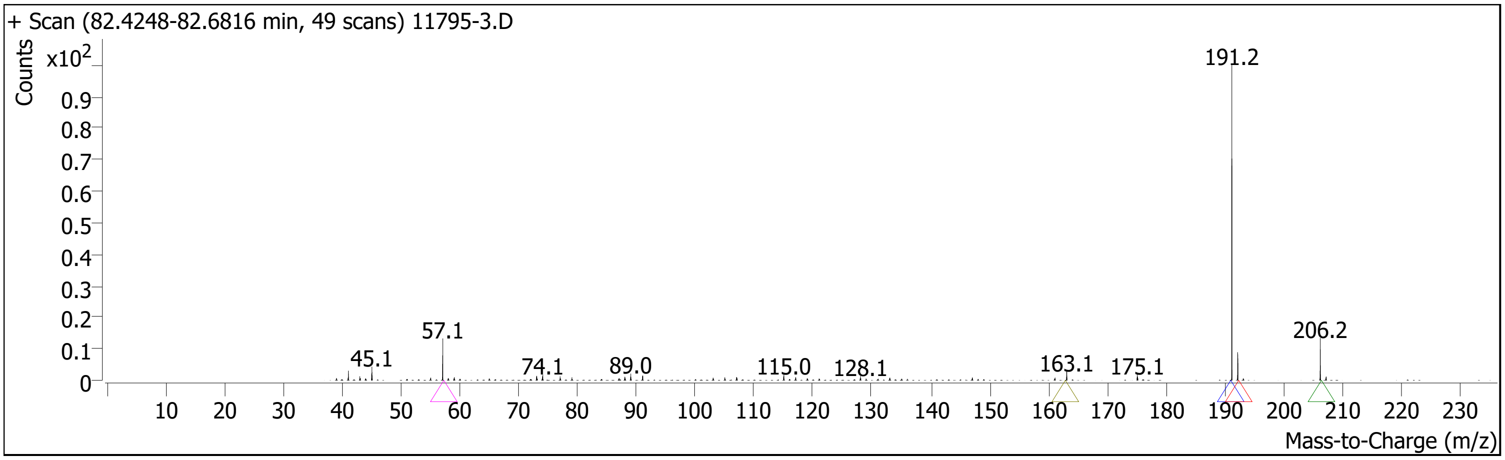

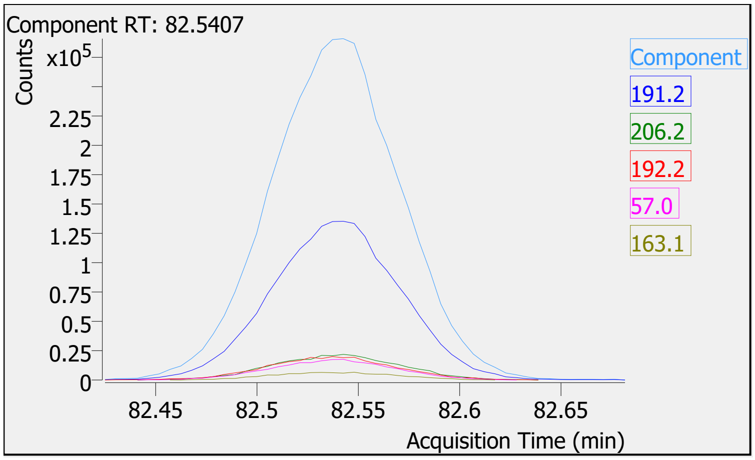

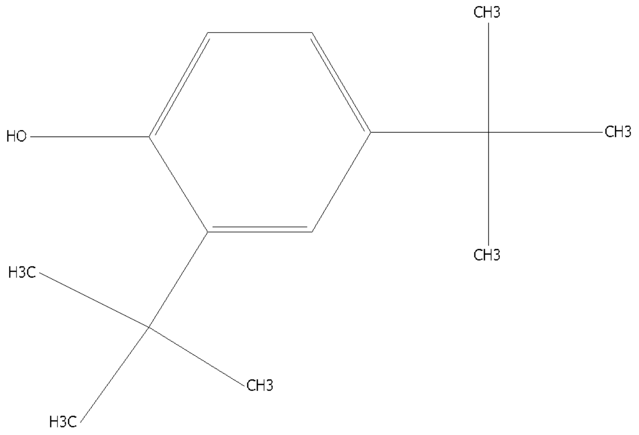

 Generated at 4:34 PM on 11/14/2023

Unknown Analysis Report - Best Hits

RT	Compound Name	CAS#	Formula	Area	MI	Match Score	Sample	Sample
88.6146	1,2,4-Trimethoxy-5-[(1Z)-1-propenyl]benzene	5273-86-9	C12H16O3	50156		78.7	0.53	0.87

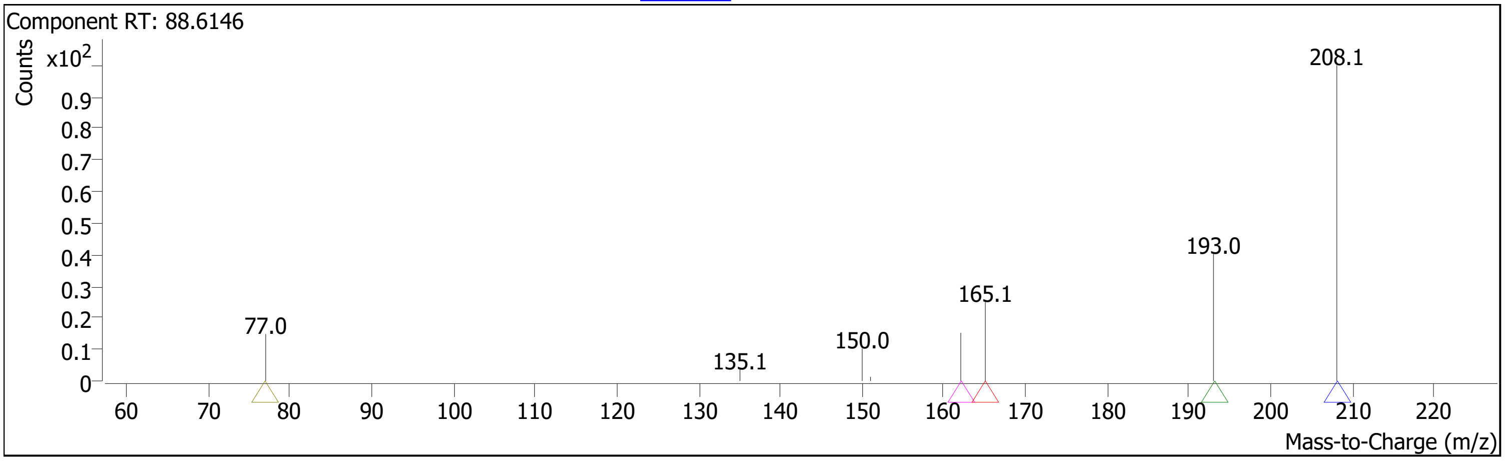

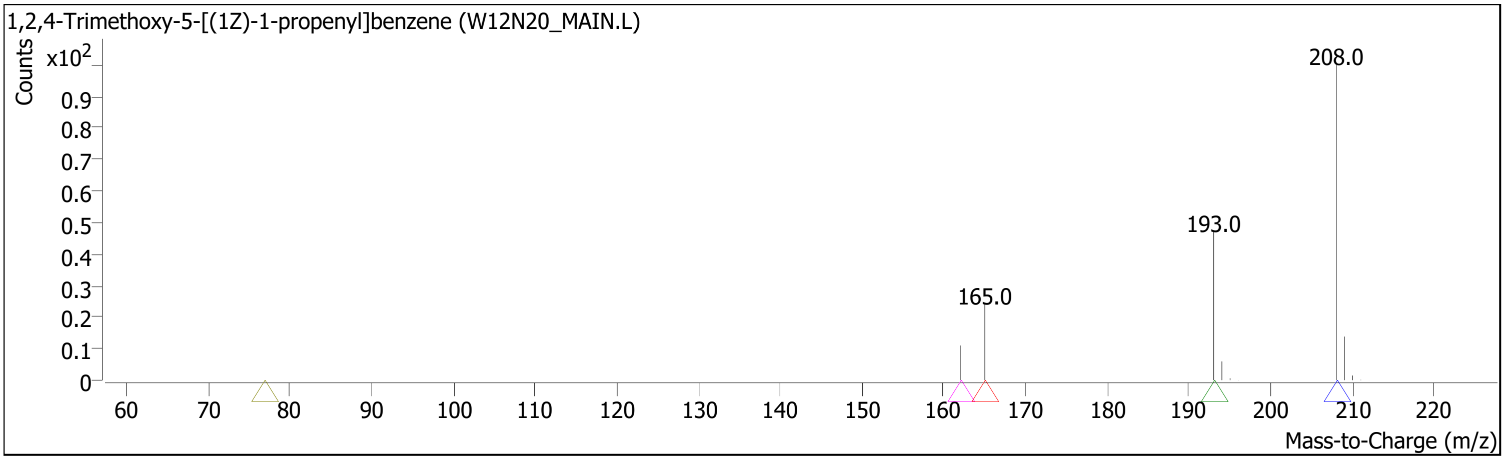

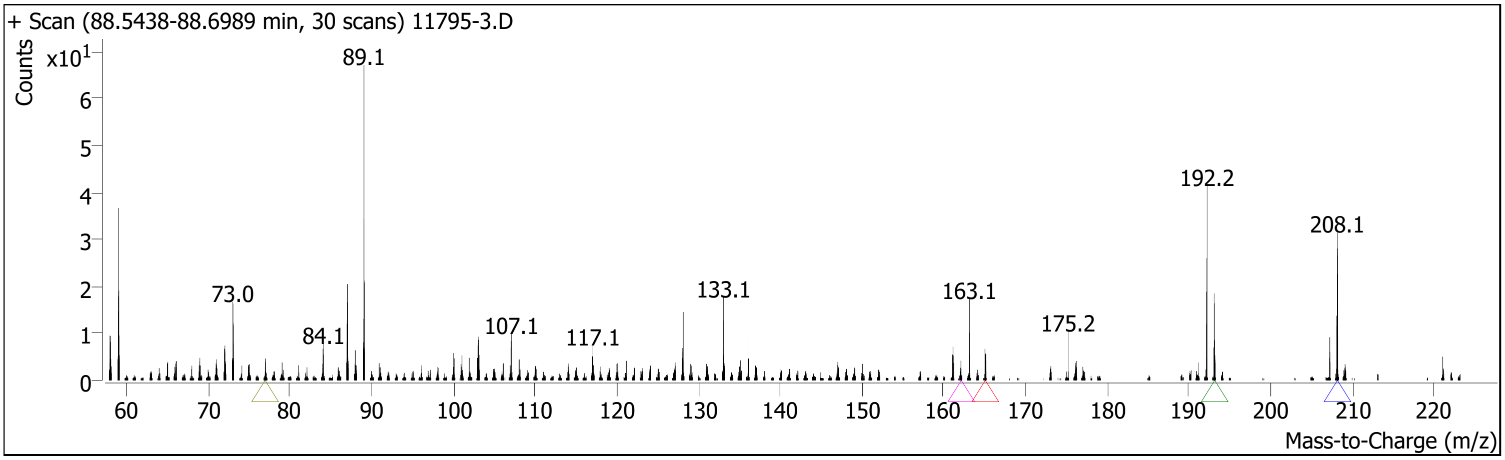

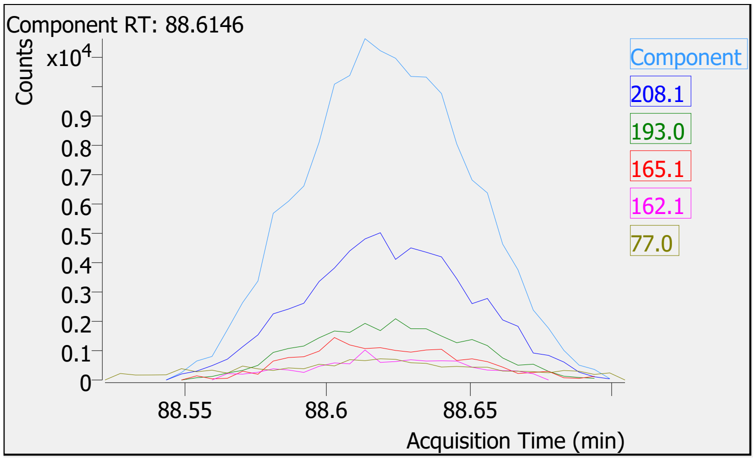

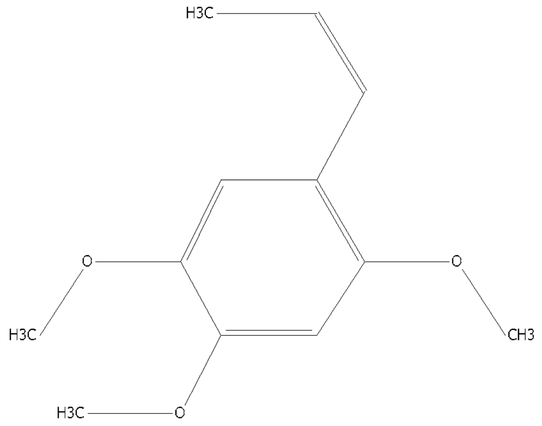

Unknown Analysis Report - Best Hits

RT	Compound Name	CAS#	Formula	Area	MI	Match Score	Sample	Sample
92.0170	1-(Benzyloxy)-2-fluoro-2-phenyl-3-(p-toluenesulsulfonyloxy)propane	990534-16-2	C23H23FO4S	26067		83.8	0.27	0.45

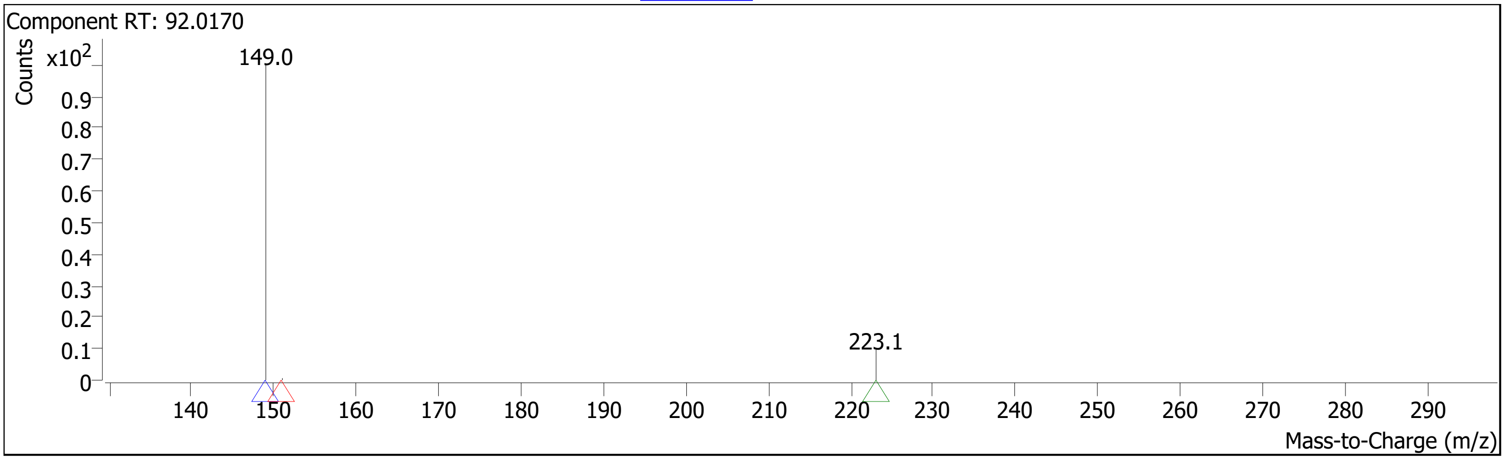

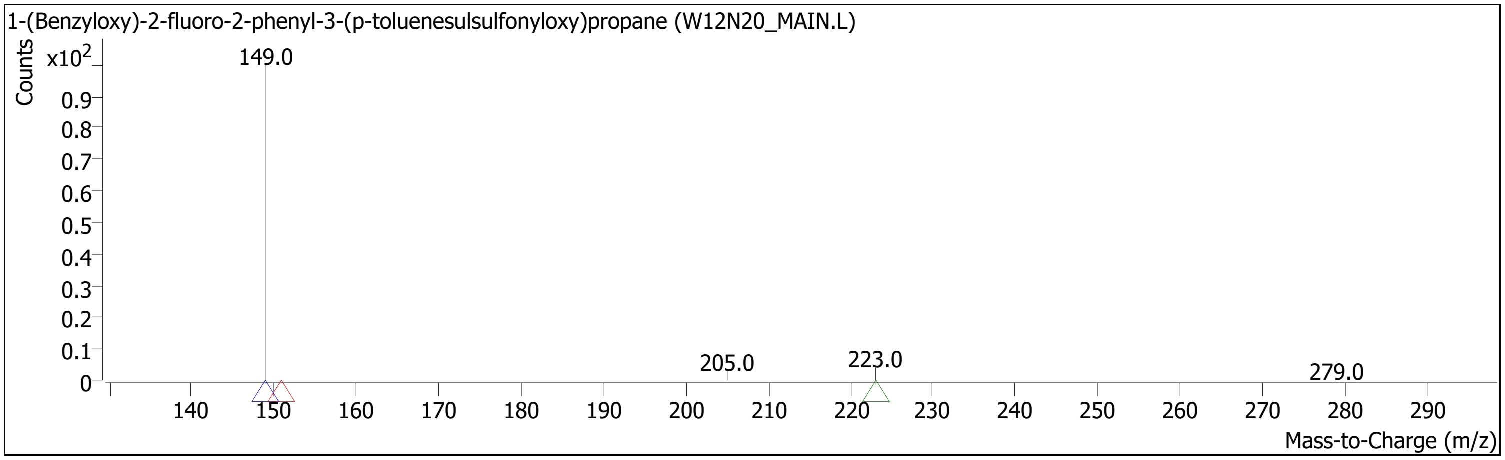

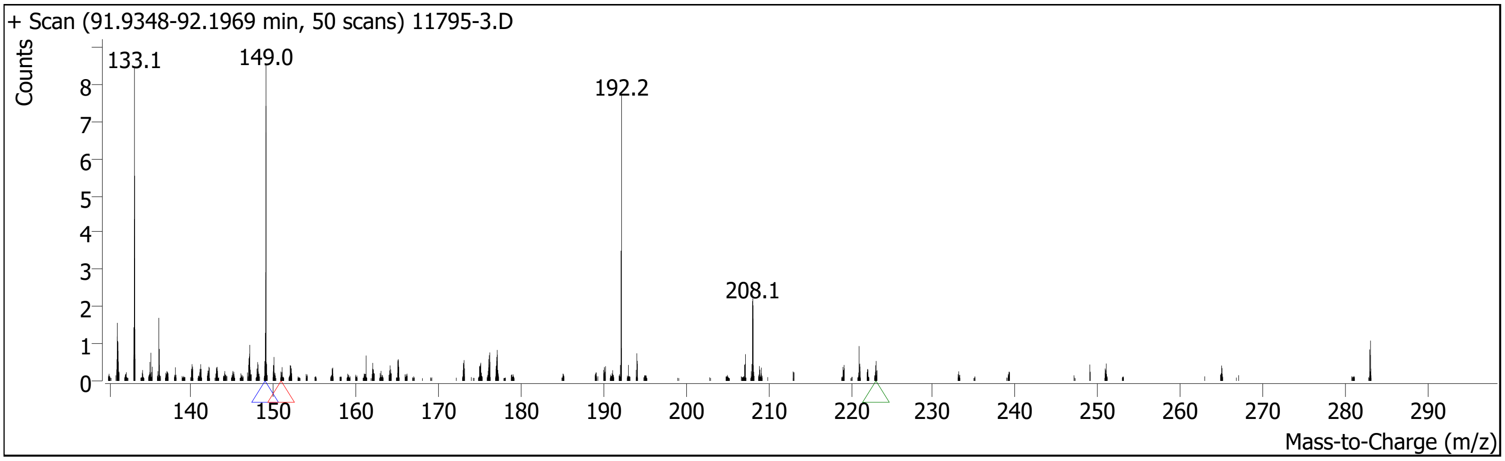

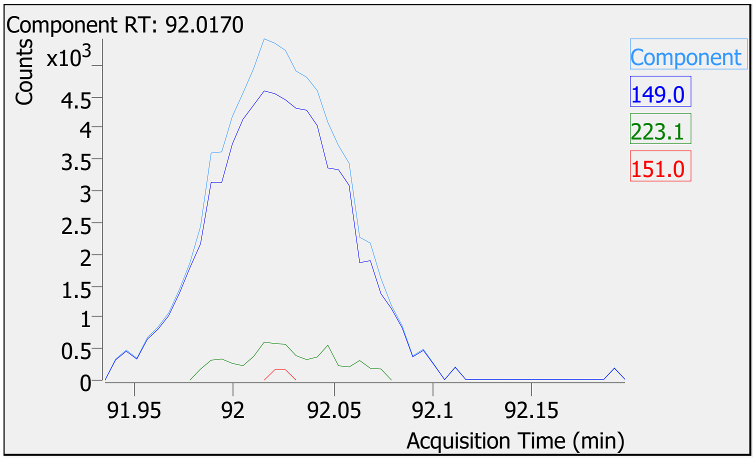

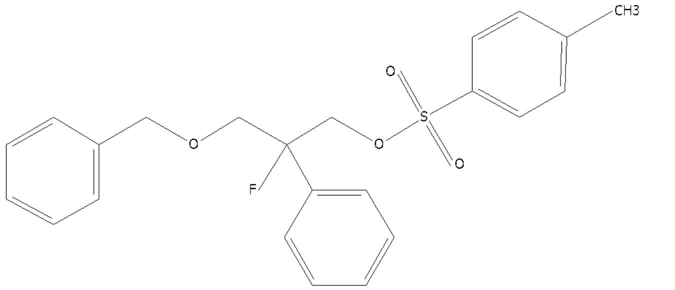

Unknown Analysis Report - Best Hits

RT	Compound Name	CAS#	Formula	Area	MI	Match Score	Sample	Sample
93.7940	3-Methyl-1,1-diphenyl-urea	990124-21-3	C14H14N2O	20182		89.0	0.21	0.35

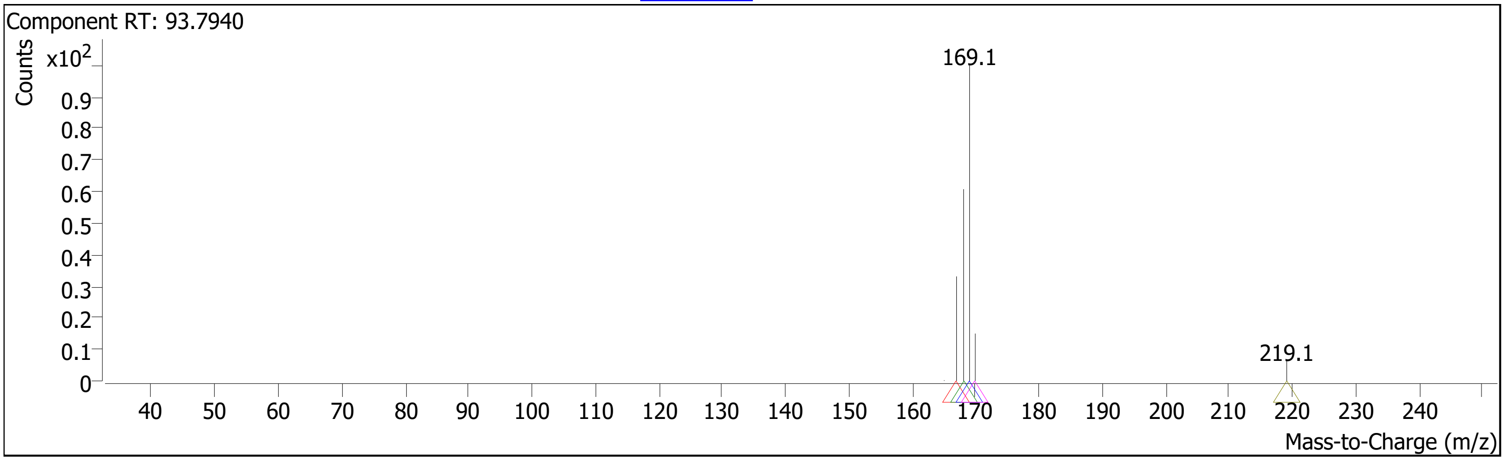

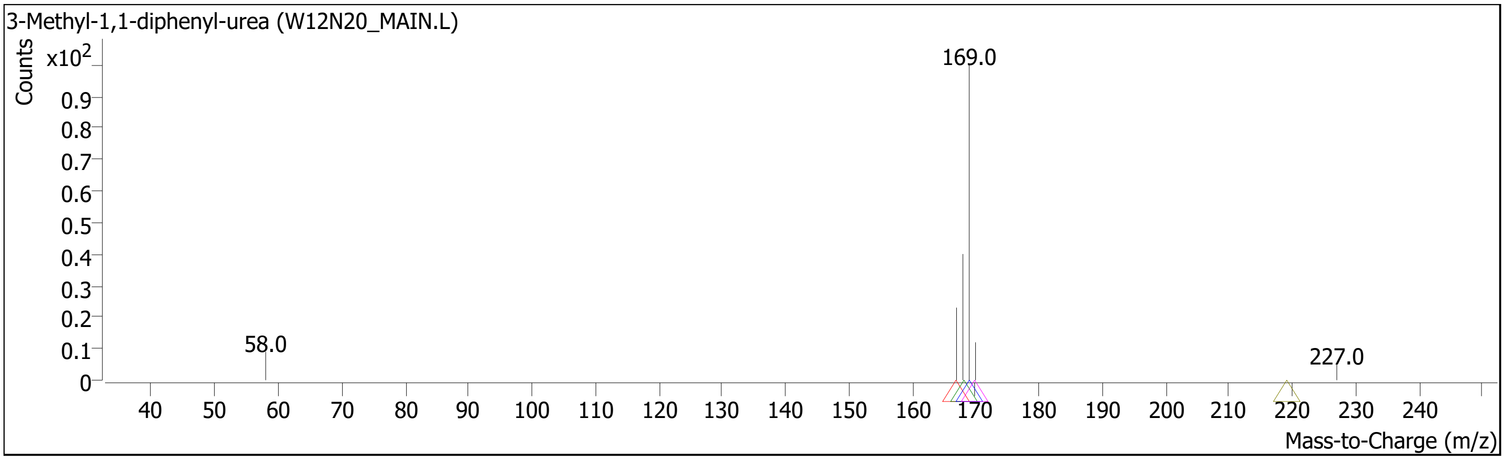

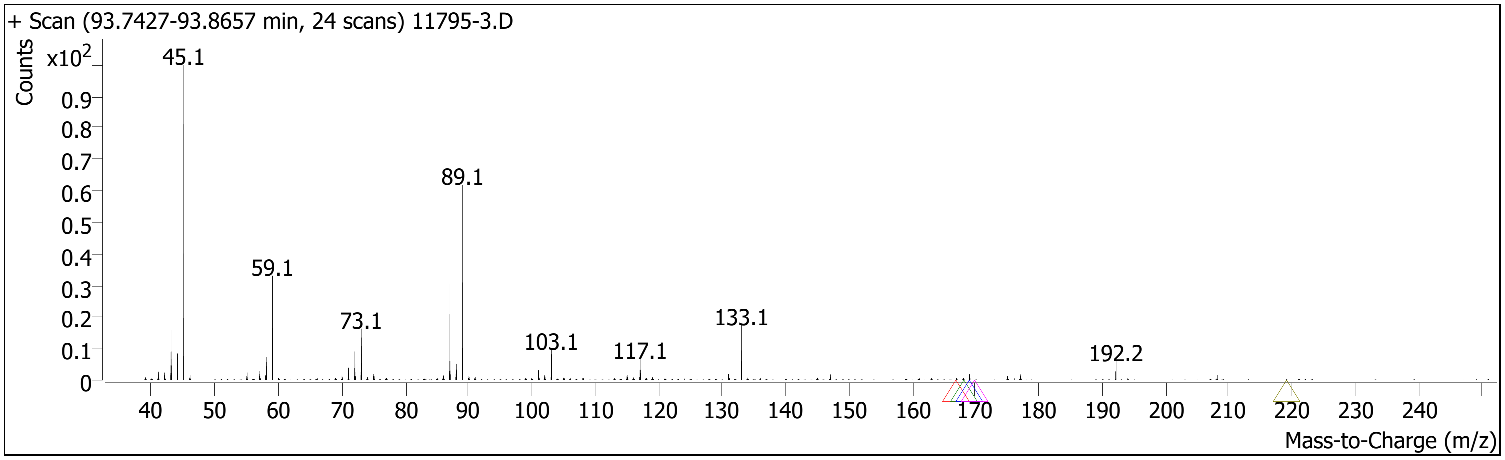

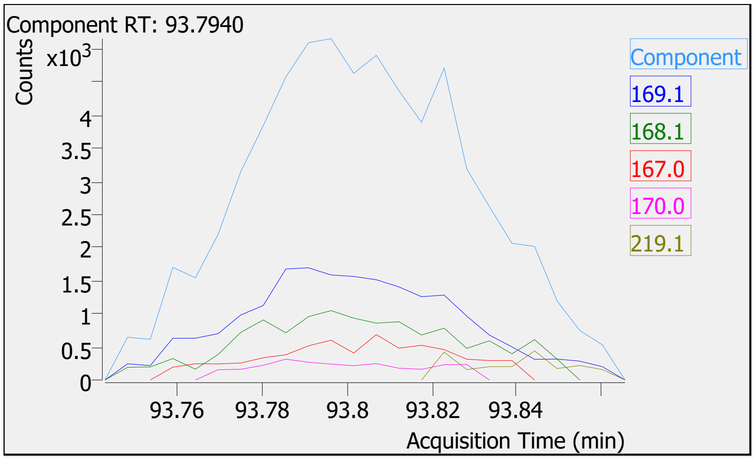

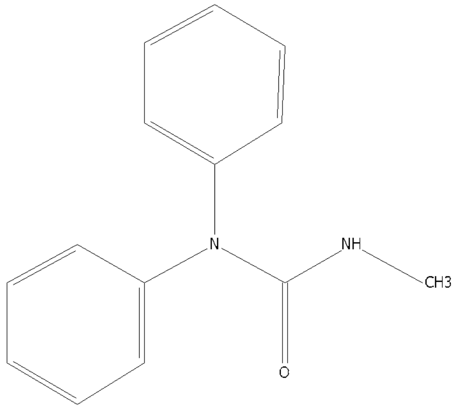

Unknown Analysis Report - Best Hits

RT	Compound Name	CAS#	Formula	Area	MI	Match Score	Sample	Sample
94.5268	1-Phenyl-3-pentyn-1-ol	990021-78-0	C11H12O	22147		87.1	0.23	0.38

Component RT: 94.5268

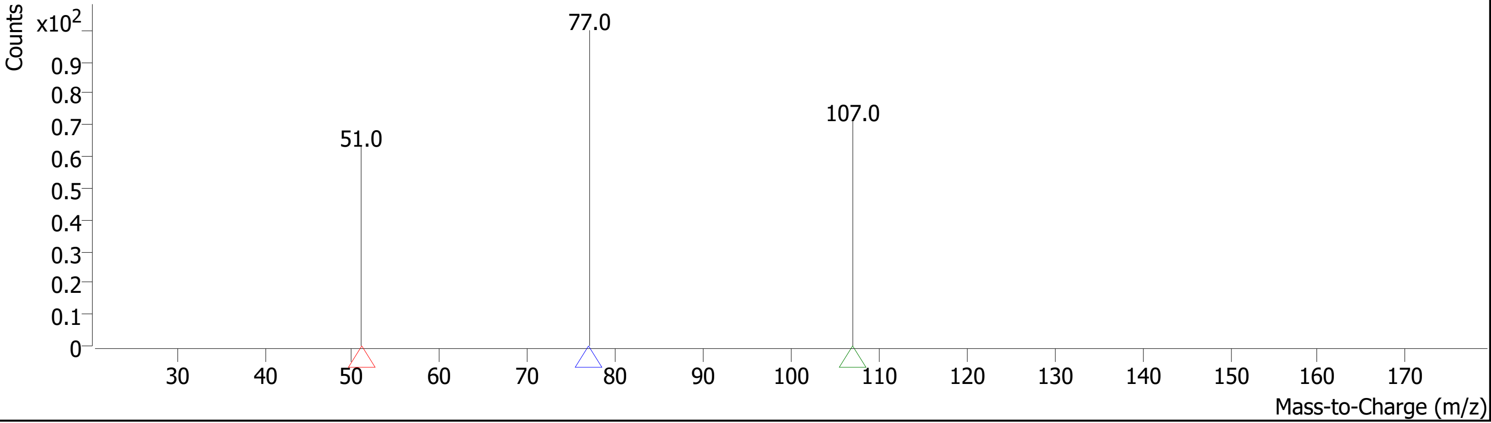

1-Phenyl-3-pentyn-1-ol (W12N20_MAIN.L)

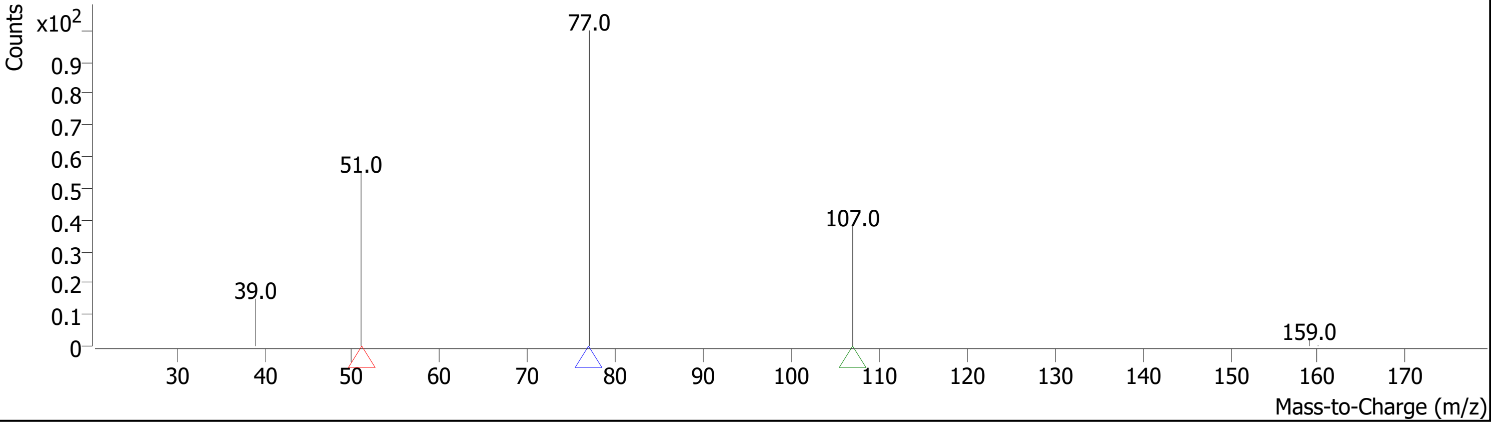

+ Scan (94.3631-94.6413 min, 53 scans) 11795-3.D

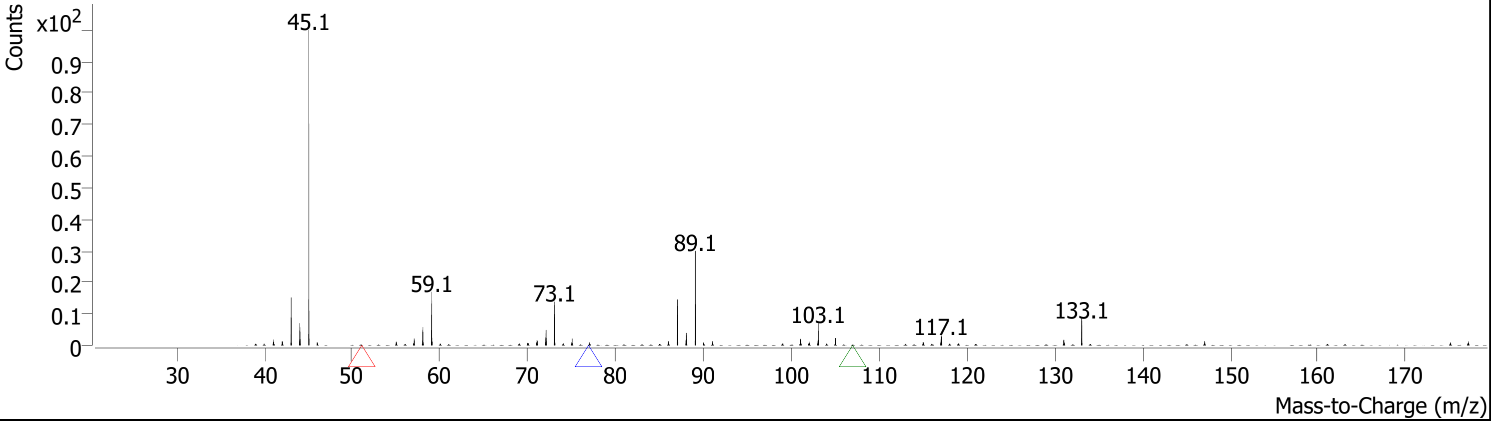

Component RT: 94.5268

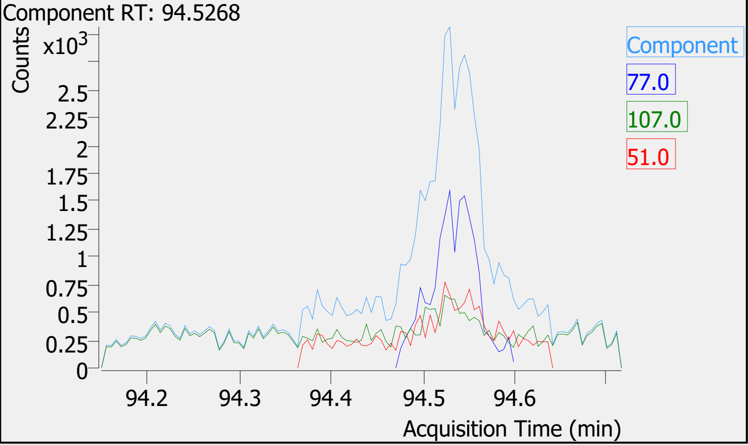

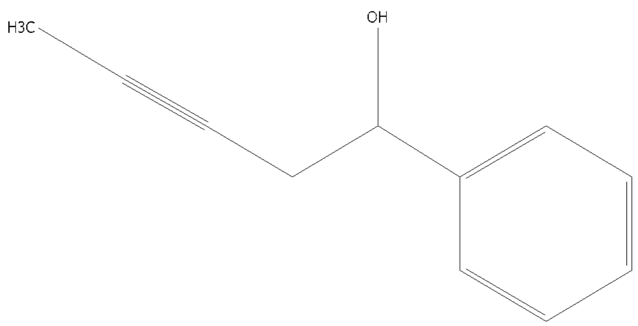

RT	Compound Name	CAS#	Formula	Area	MI	Match Score	Sample	Sample
94.5333	2-(3-Pyridyl)-3-(4-toluenesulfonamido)propylazetidine	62247-30-7	C15H16N2O2S	368892		92.6	3.88	6.37

Component RT: 94.5333

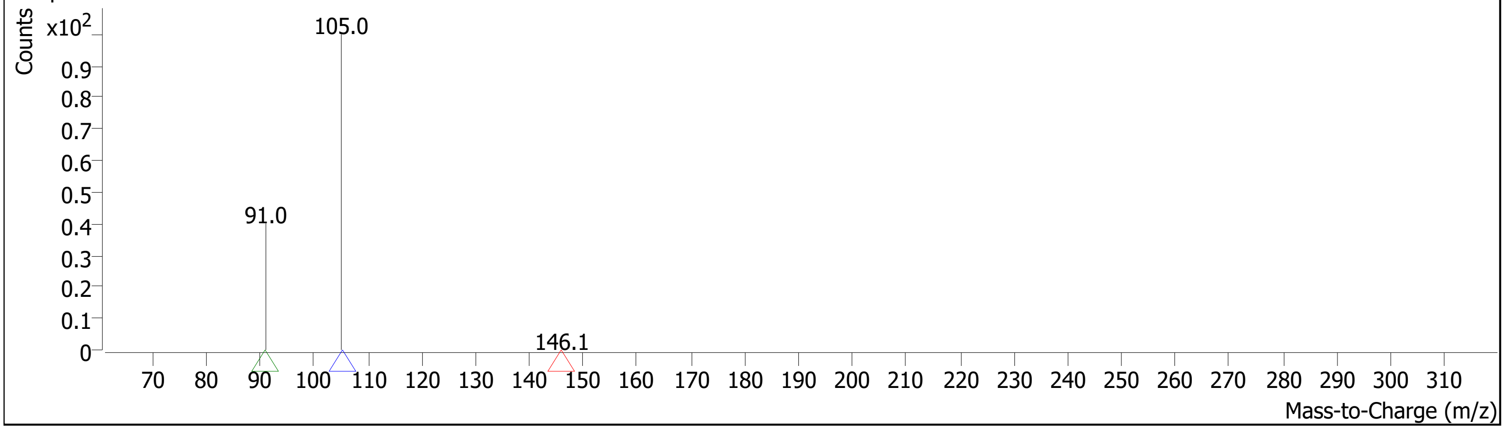

2-(3-Pyridyl)-3-(4-toluenesulfonamido)propylazetidine (W12N20_MAIN.L)

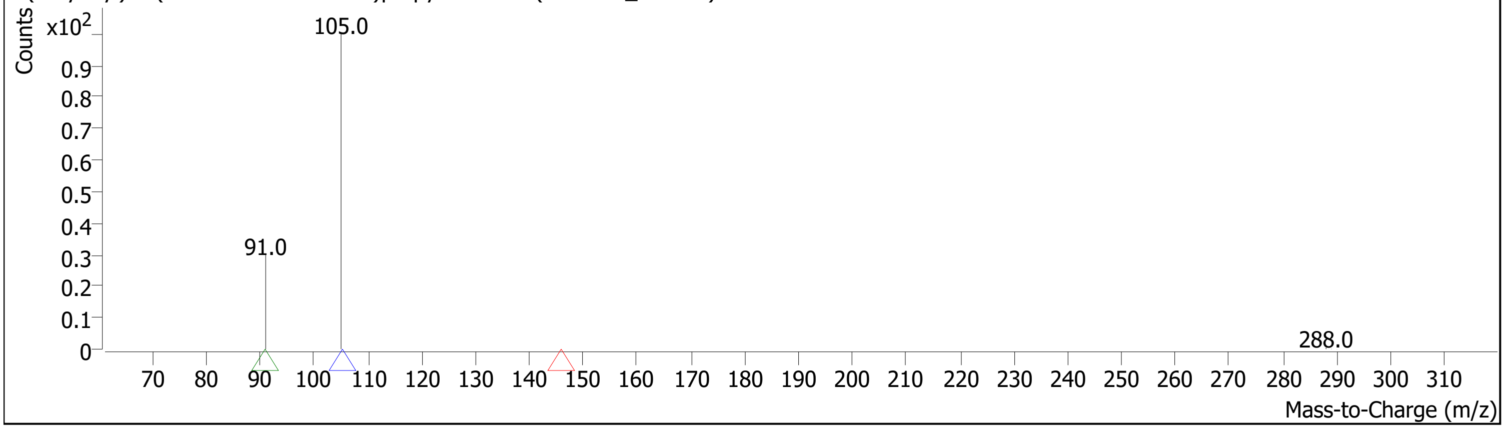

+ Scan (94.2455-98.4602 min, 789 scans) 11795-3.D

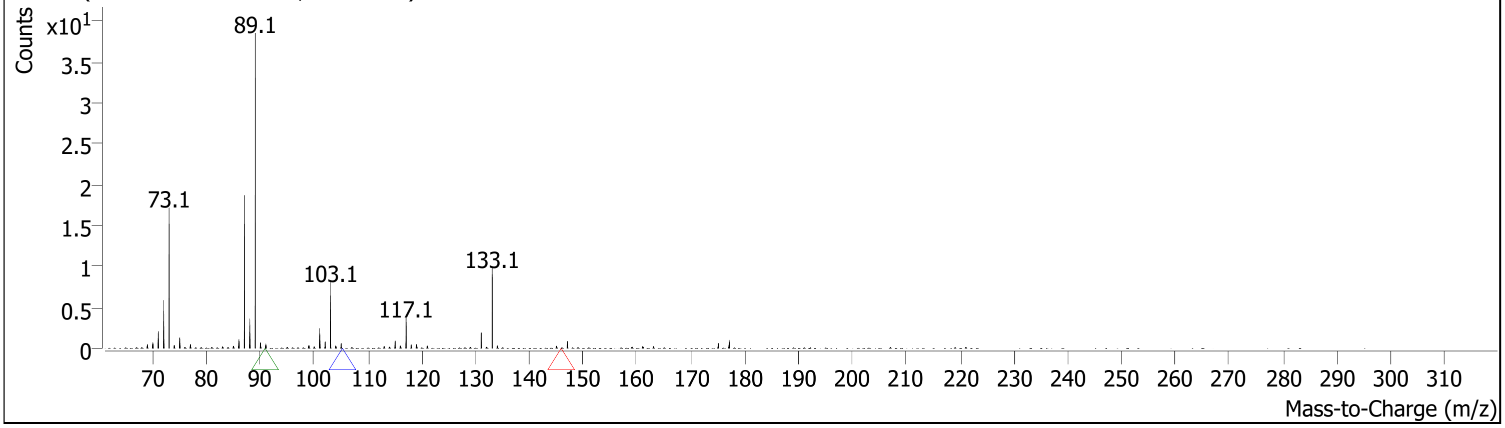

RT	Compound Name	CAS#	Formula	Area	MI	Match Score	Sample	Sample
100.2931	2-Oxo-4-phenylbut-3-enyl oct-2-ynoate	990264-51-1	C18H20O3	5791896		85.6	60.87	100.00

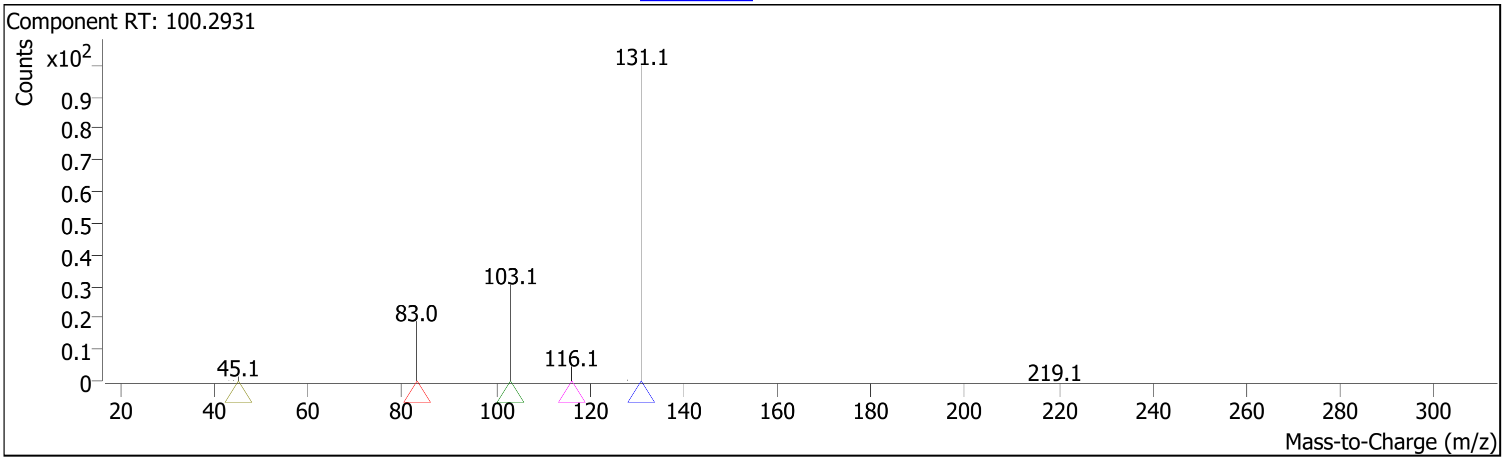

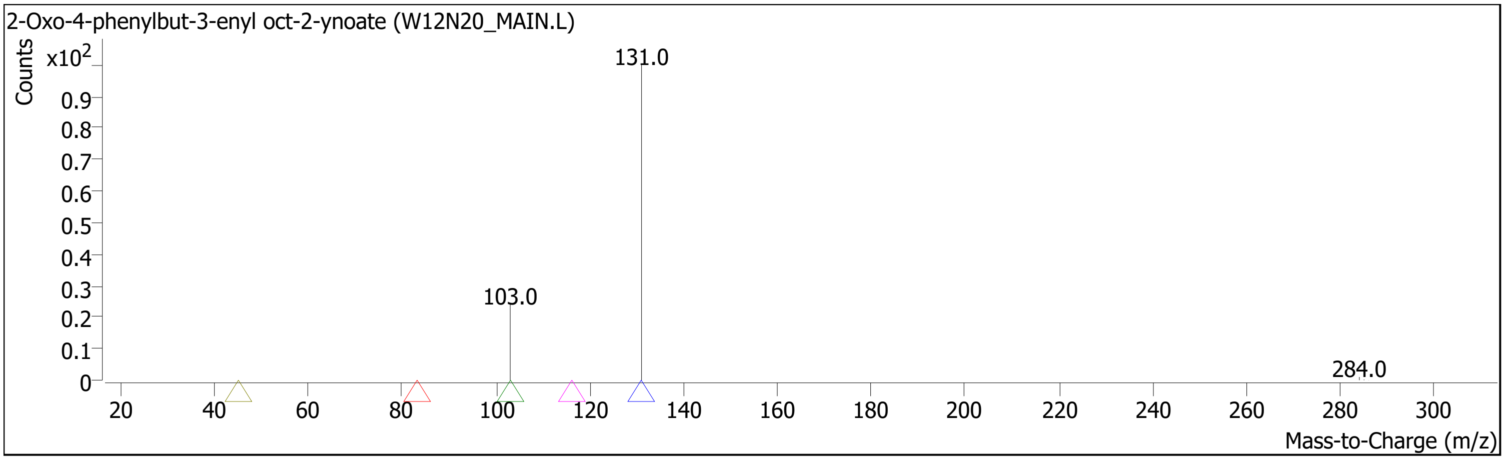

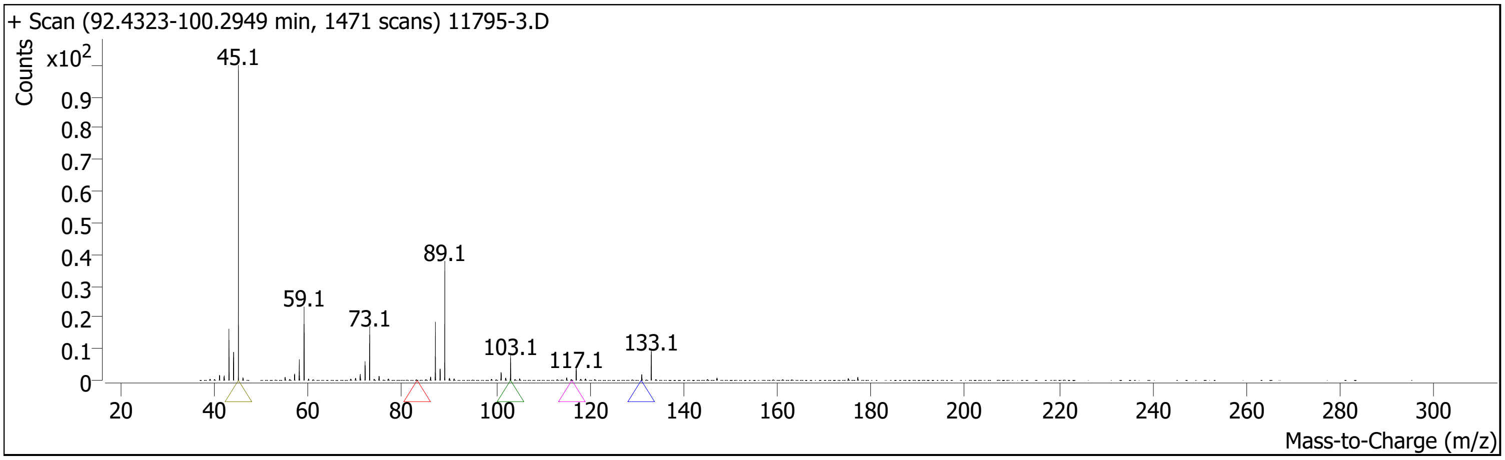

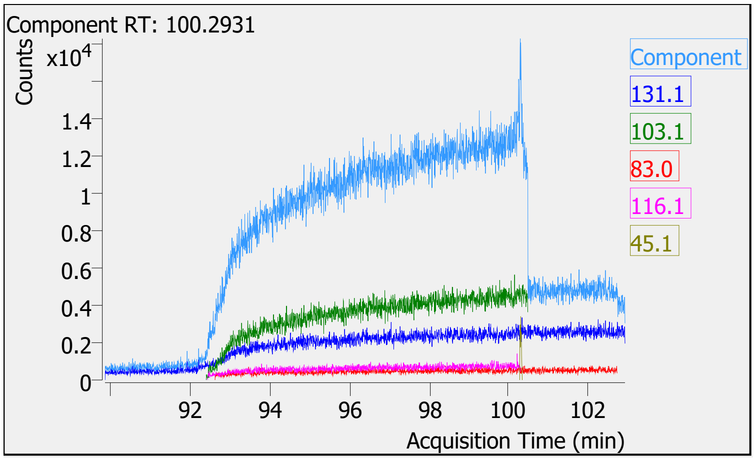

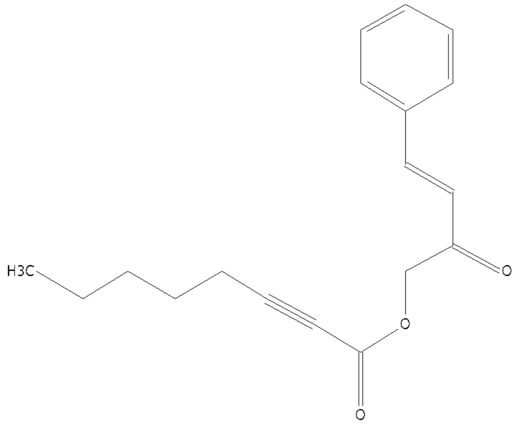

Supplement: Supplementary file 3 — Supplementary Information 3. [file 41598_2024_56958_MOESM3_ESM.pdf]
