# Supplementary material for: Lactiplantibacillus plantarum K8 lysates regulate hypoxia-induced gene expression
Source: Sci Rep. 2024 Mar 15;14:6275. doi: 10.1038/s41598-024-56958-7 (PMC10943017; doi:10.1038/s41598-024-56958-7)
Supplement: Supplementary file 4 — Supplementary Information 4. [file 41598_2024_56958_MOESM4_ESM.pdf]

Unknown Analysis Report - Best Hits

**Batch Path**	D:\MassHunter\GCMS\1\data\2023\2023-10		
**Analysis File Name**	11795.uaf		
**Analyst Name**	admin		
**Analysis Time**	11/14/2023 4:19:03 PM		
**Data File Name**	11795-2.D	**Data Path Name**	D:\MassHunter\GCMS\1\data\2023\2023-10
**Sample Name**	K8 Powder	**Sample Type**	Sample
**Acq Method File**	DB-WAX	**Acq Method Path**	D:\MassHunter\GCMS\1\methods\
**Acq Time**	11/13/2023 8:00:46 PM	**Operator**	
**Instrument Name**	GCMS	**Dilution**	1

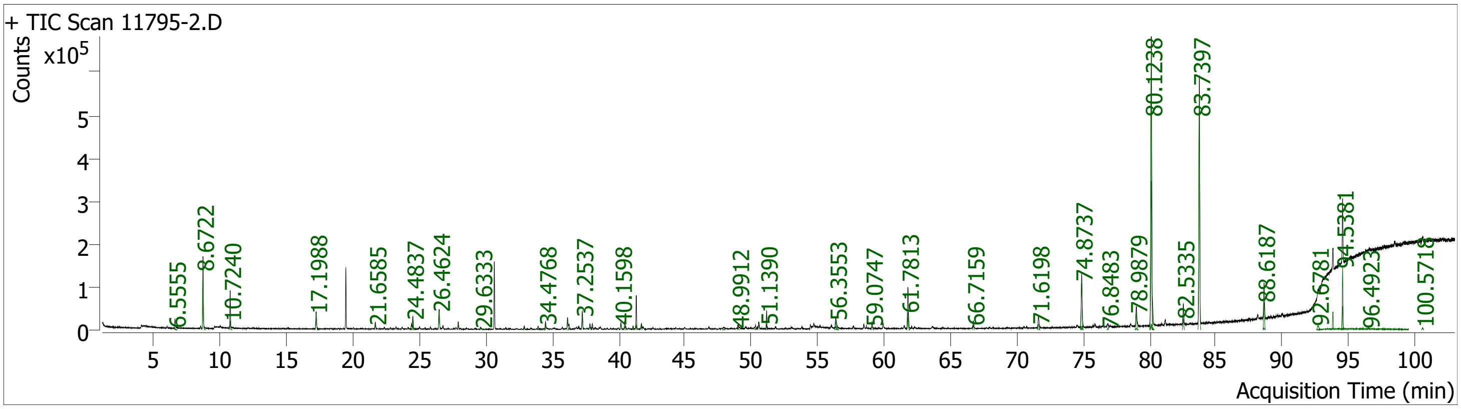

RT	Compound Name	CAS#	Formula	Area	MI	Match Score	Sample	Sample
6.5555	2,3-bis[(E)-3-(3,4-dimethoxyphenyl)-1-oxoprop-2-enoxy]butanedioic acid	990620-32-9	C26H26O12	14959		73.8	0.12	0.46
8.6722	Dimethyl ether	115-10-6	C2H6O	473357		95.0	3.64	14.59
10.7240	3,5-Bis[(E)-2-phenylethenyl]pyridine	990261-79-8	C21H17N	61015		79.2	0.47	1.88
17.1988	2-Propanol, 1-methoxy-	107-98-2	C4H10O2	154280		95.4	1.19	4.76
21.6585	Tridecane, 7-hexyl-	7225-66-3	C19H40	44178		99.6	0.34	1.36
24.4837	2,6,10 - trimethyl - tridecane (without stereochemistry)	3891-99-4	C16H34	100373		85.7	0.77	3.09
24.4866	N-Methoxy-N-methyl-2-ethylhexanoylamide	990051-95-9	C10H21NO2	38017		79.1	0.29	1.17
26.4624	Cyclohexane, isocyanato-	3173-53-3	C7H11NO	188265		88.8	1.45	5.80
27.9117	Heptadecane, 2,6,10,15-tetramethyl-	54833-48-6	C21H44	35349		98.9	0.27	1.09
29.6333	1,3-Dioxolane	646-06-0	C3H6O2	31149		83.0	0.24	0.96
32.8903	Hexadecane, 1-iodo-	544-77-4	C16H33I	13992		90.6	0.11	0.43
33.9234	Anhydro - sugar	0-00-0	C5H8O4	12534		75.1	0.10	0.39
34.4768	Heptadecane, 2,6,10,15-tetramethyl-	54833-48-6	C21H44	13057		91.1	0.10	0.40
36.1422	1,2-Di-tert-butylbenzene	1012-76-6	C14H22	84507		90.1	0.65	2.61
36.2431	2,6-Nonanedione	36452-81-0	C9H16O2	16311		80.5	0.13	0.50
37.2537	Decane, 3-ethyl-3-methyl-	17312-66-2	C13H28	116361		87.3	0.89	3.59
37.8255	Hexadecane, 1-iodo-	544-77-4	C16H33I	32347		91.0	0.25	1.00
38.0086	Hexadecane, 1-iodo-	544-77-4	C16H33I	22539		91.4	0.17	0.69
40.1598	4,4-Dimethylpentan-1-ol	990002-63-9	C7H16O	49005		85.5	0.38	1.51
40.4580	2,2-Dimethylpropanoic acid 3-acetyloxypropyl ester	990075-06-2	C10H18O4	33537		94.0	0.26	1.03
41.0597	2,2-Dimethylpropanoic acid 3-acetyloxypropyl ester	990075-06-2	C10H18O4	11811		92.3	0.09	0.36
41.3152	4H-1-Benzopyran-4-one, 5,6,7-trimethoxy-2-(4-methoxyphenyl)-	1168-42-9	C19H18O6	30496		74.2	0.23	0.94
41.6900	.beta.,D-Xylopyranose Tetrabenzoate	990630-11-9	C33H26O9	32584		93.9	0.25	1.00
47.9288	2,2-Dimethylpropanoic acid 3-acetyloxypropyl ester	990075-06-2	C10H18O4	10105		89.6	0.08	0.31
48.9912	Hexadecane, 1-iodo-	544-77-4	C16H33I	19014		91.3	0.15	0.59
50.2964	1,1-Cyclopropanedicarbonitrile, 2-methyl-2-pentyl-	16738-90-2	C11H16N2	10479		79.3	0.08	0.32
50.5562	3,5-bis[14C]-Trachelantamidine	990009-77-6	C8H15NO	49660		84.2	0.38	1.53
51.1328	3-Methoxy-2'-phenyl-16.beta.,17.beta.-dihydro-4'H-[1,3]oxazino[5',6' : 16,17]estra-1,3,5(10)triene	990516-57-1	C27H31NO2	31498		71.0	0.24	0.97

RT	Compound Name	CAS#	Formula	Area	MI	Match Score	Sample	Sample
51.1390	2,3-Diphenyl-5,8,9,10-tetrahydropyrimido[1,2-c]pteridin-6-one	990434-90-9	C21H17N5O	34090		82.2	0.26	1.05
56.3455	5-Diazo-1,3-cyclopentadiene	1192-27-4	C5H4N2	35619		72.8	0.27	1.10
56.3553	Methyl salicylate	119-36-8	C8H8O3	118665		92.5	0.91	3.66
56.4253	2-Acetamidopentyl acetate	990051-78-0	C9H17NO3	14217		88.8	0.11	0.44
59.0747	4-Methylphthalide	990013-04-1	C9H8O2	39555		84.9	0.30	1.22
61.7813	(Z,Z)-(+-)-cis-2-(2,5-Octdienyl)-3-undecyloxirane	990320-72-3	C21H38O	195491		91.3	1.50	6.03
61.7888	(R)-S-Ethylthio mandelate	990065-39-2	C10H12O2S	134907		86.2	1.04	4.16
66.7159	Bis-(3,5,5-trimethylhexyl) ether	990229-48-0	C18H38O	44402		86.0	0.34	1.37
71.6198	(phenylmethyl) undec-10-ynoate	990234-29-8	C18H24O2	63156		90.4	0.49	1.95
71.6832	2-Methoxyindan-1-one	990023-11-2	C10H10O2	12047		78.2	0.09	0.37
74.8737	2(3H)-Furanone, 5-hexyldihydro-	706-14-9	C10H18O2	529410		93.2	4.07	16.32
74.8827	Methyl (2RS,3RS)-3-Acetoxy-2-[(SR)-1-hydroxyethyl]butanoate	990078-31-9	C9H16O5	35865		74.9	0.28	1.11
74.9159	2H-Cyclopropa[b]naphthalen-2-one, 1,1a,2a,3,6,6a,7,7a-octahydro-1,1,2a,4,5-pentamethyl-, [1aS-(1a.alpha.,2a.beta.,6a.beta.,7a.alpha.)]-	127279-91-8	C16H24O	33019		88.7	0.25	1.02
76.8483	2-Propenoic acid 3-[(1E,3Z)-penta-1,3-dienoxy]propyl ester	990066-09-6	C11H16O3	14830		86.1	0.11	0.46
78.9879	Benzyl lactate	990042-49-0	C10H12O3	189653		88.4	1.46	5.85
78.9925	Heptane, 1,1,1,2,3,3-hexafluoro-	57915-71-6	C7H10F6	18882		86.9	0.15	0.58
80.1048	2-Methyl-1,3-oxazole-4-carboxylic acid ethyl ester	990017-80-8	C7H9NO3	39466		74.9	0.30	1.22
80.1238	2(3H)-Furanone, 5-heptyldihydro-	104-67-6	C11H20O2	3243609		94.5	24.95	100.00
80.1241	(E)-1-(2-Tetrahydropyranyloxy)-7-decene	990155-85-8	C15H28O2	1713054		76.1	13.18	52.81
80.1345	1-(Bromoethynyl)cyclohexan-1-on	0-00-0	C8H11BrO	64737		86.6	0.50	2.00
82.5335	Pyrimidinium, 5-carboxy-4-(1,1-dimethylethyl)-1,6-dimethyl-2-(1-methylethyl)-, hydroxide, inner salt	108169-08-0	C14H22N2O2	110617		82.9	0.85	3.41
83.7397	Benzene, 1,2,3-trimethoxy-5-(1-propenyl)-, (E)-	5273-85-8	C12H16O3	2447567		91.6	18.83	75.46
88.6036	tert-Butyl (2S*,7aR*)-2-(2-methoxy-2-oxoethyl)-2-[(methylsulfonyl)oxy]-3-oxotetrahydr-1H-pyrrolizine-7a(5H)-carboxylate	0-00-0	C16H25NO8	18494		84.1	0.14	0.57
88.6187	.beta.-Asarone	5273-86-9	C12H16O3	294602		84.0	2.27	9.08
92.6781	(E)-phenyl 3-cyclohexyl-2-methylacrylate	990165-06-3	C16H20O2	80979		77.7	0.62	2.50
93.8068	3-(3-Methylphenyl)-pyridine	990030-50-7	C12H11N	151128		91.5	1.16	4.66
94.5309	3-(Benzyloxy)-1-propanal	990024-51-7	C10H12O2	210048		75.4	1.62	6.48
94.5381	Benzyl Benzoate	120-51-4	C14H12O2	540862		95.5	4.16	16.67
96.4923	Spiro[cyclopentane-1,1'(2'H)-naphthalene]-5'-ethanamine, N-ethyl-7',8'-dimethoxy-N-methyl-	63080-55-7	C21H31NO2	818688		79.4	6.30	25.24
100.5718	(2R)-2-(6-methoxy-2-naphthalenyl)propanenitrile	108865-01-6	C14H13NO	23118		76.8	0.18	0.71

RT	Compound Name	CAS#	Formula	Area	MI	Match Score	Sample	Sample
6.5555	2,3-bis[(E)-3-(3,4-dimethoxyphenyl)-1-oxoprop-2-enoxy]butanedioic acid	990620-32-9	C26H26O12	14959		73.8	0.12	0.46

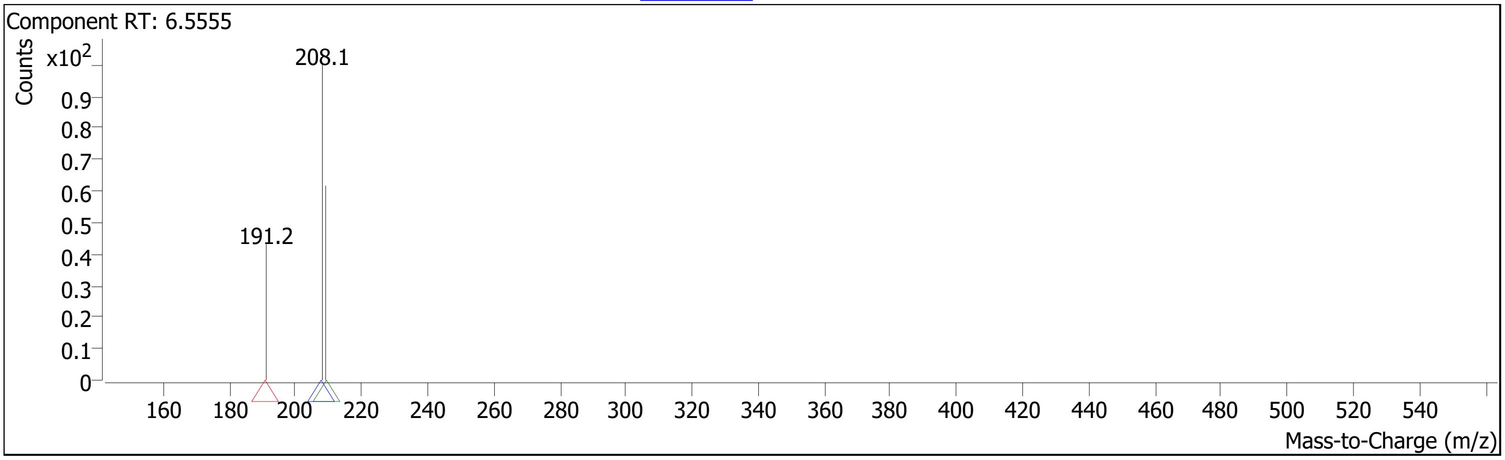

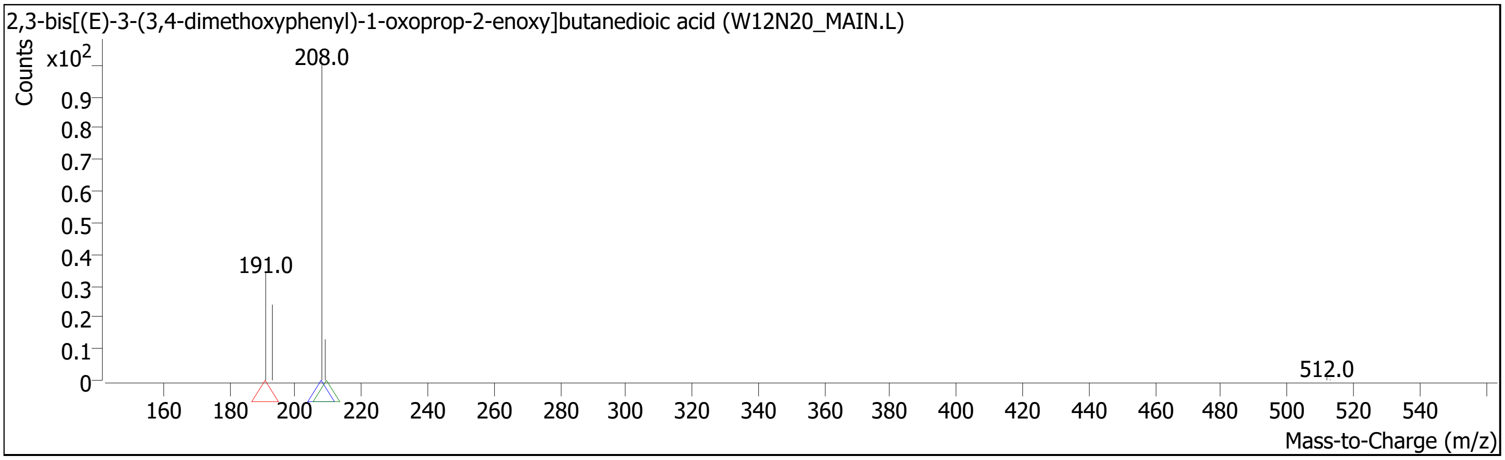

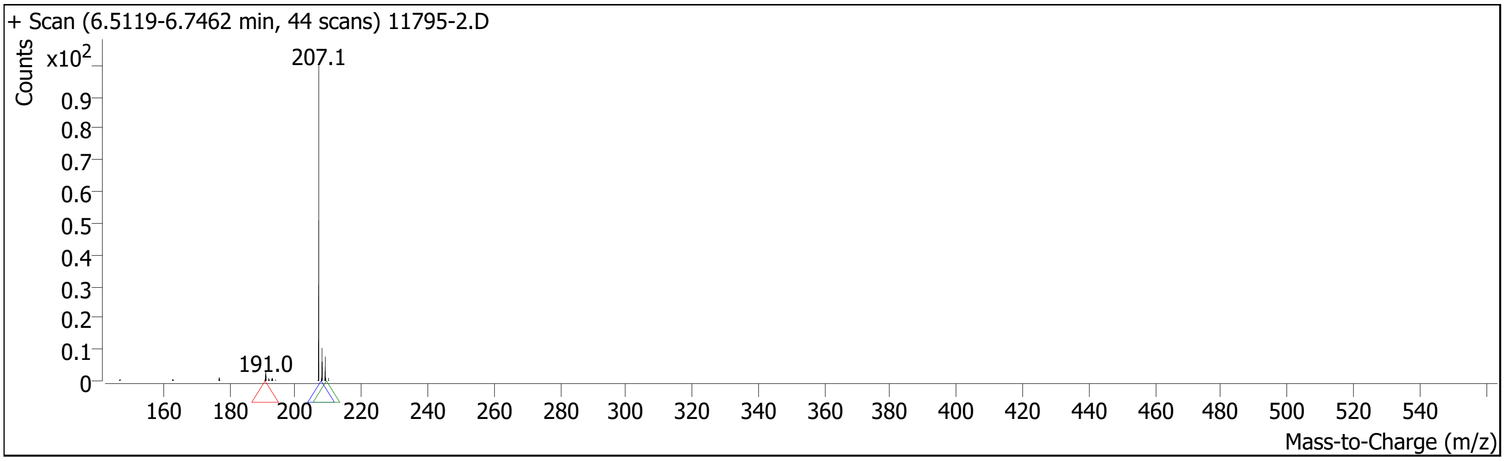

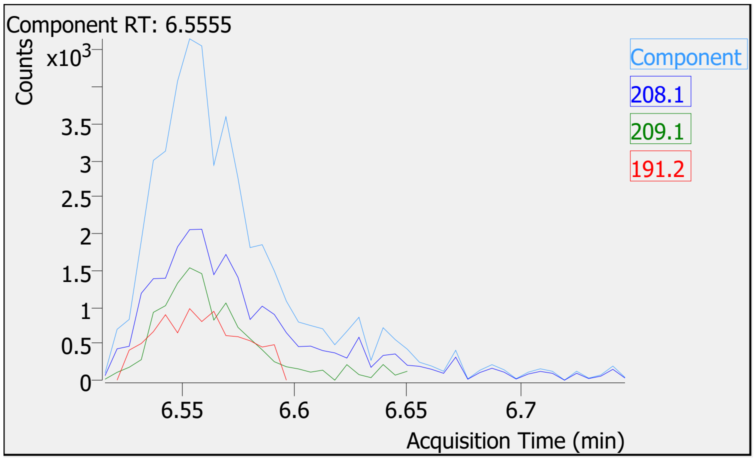

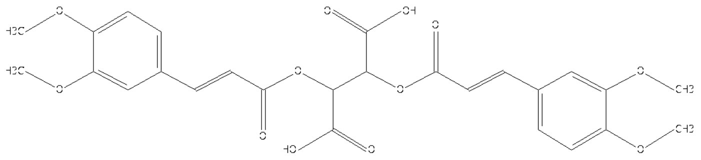

 Generated at 4:32 PM on 11/14/2023

Unknown Analysis Report - Best Hits

RT	Compound Name	CAS#	Formula	Area	MI	Match Score	Sample	Sample
8.6722	Dimethyl ether	115-10-6	C2H6O	473357		95.0	3.64	14.59

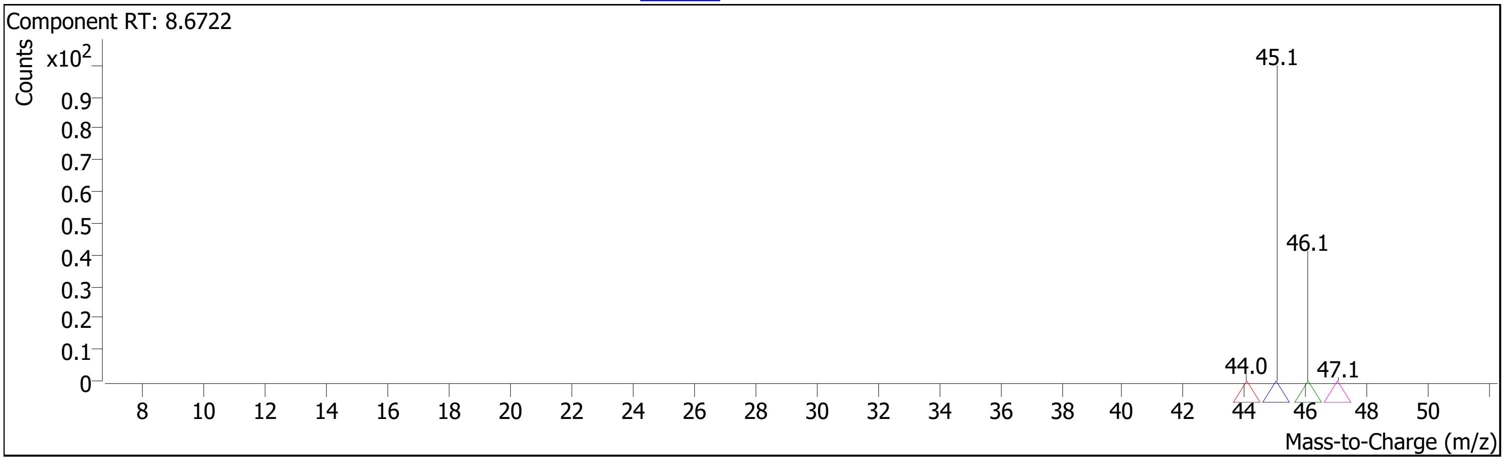

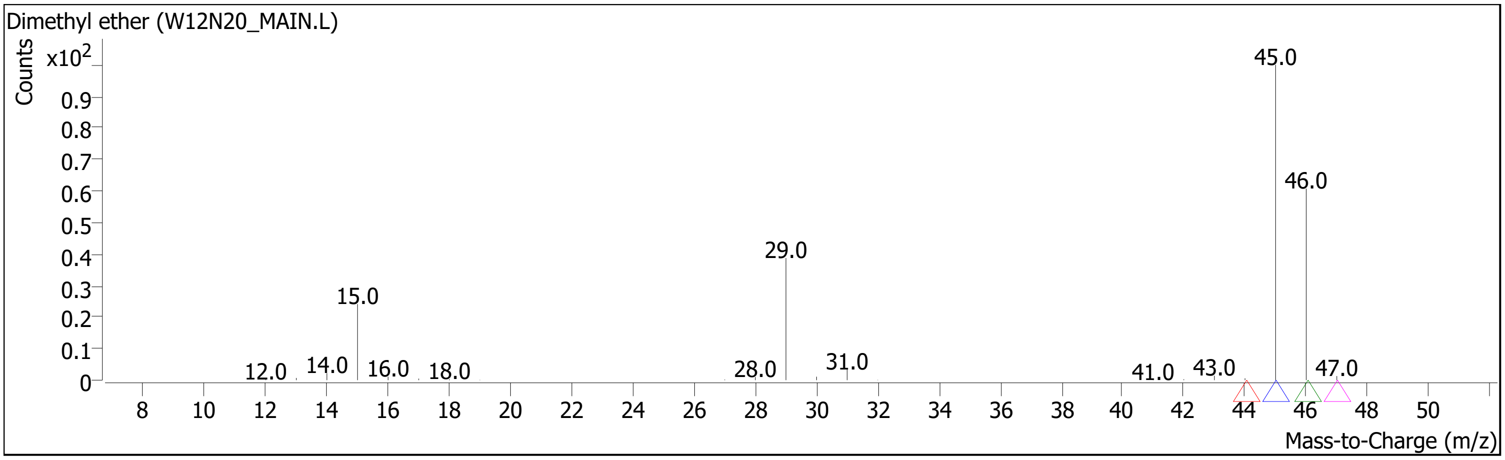

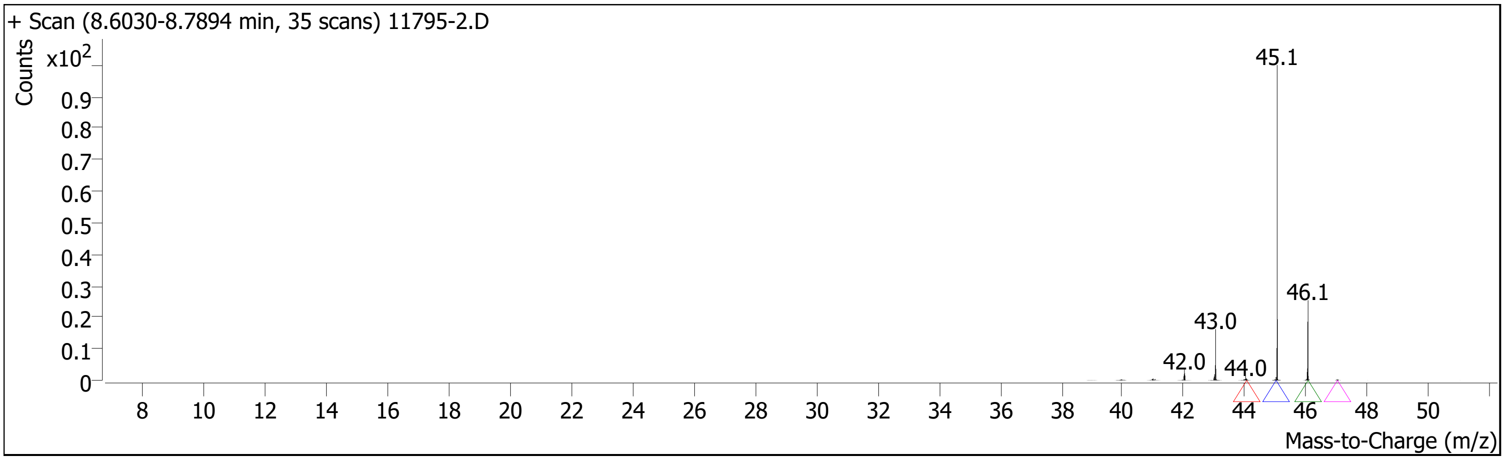

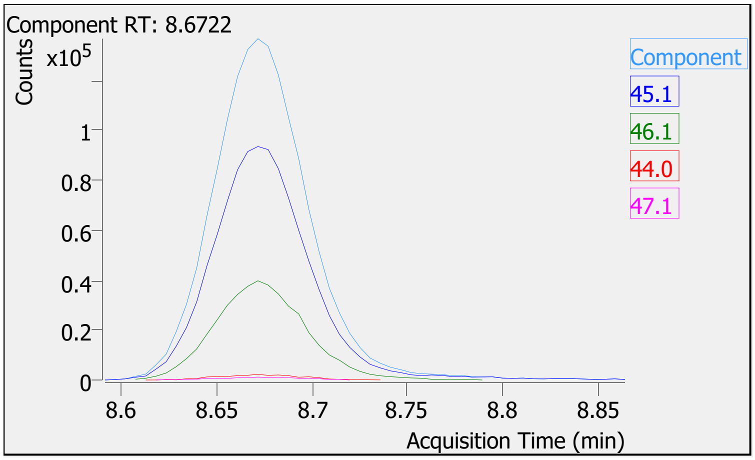

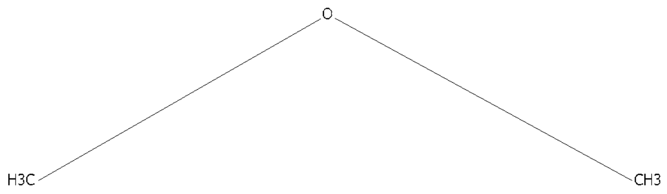

RT	Compound Name	CAS#	Formula	Area	MI	Match Score	Sample	Sample
10.7240	3,5-Bis[(E)-2-phenylethenyl]pyridine	990261-79-8	C21H17N	61015		79.2	0.47	1.88

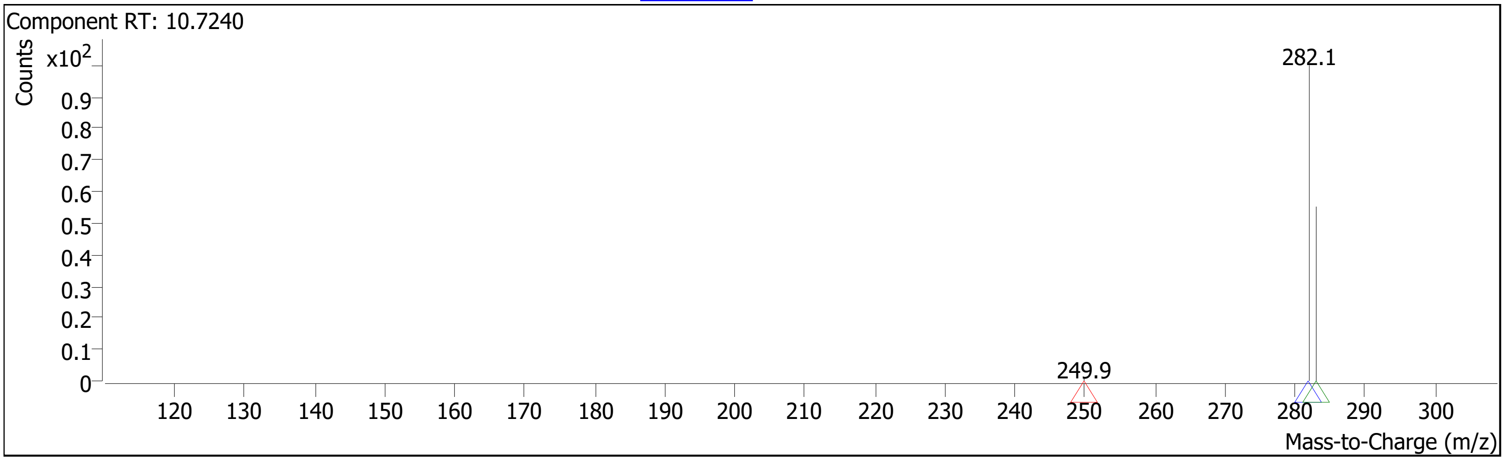

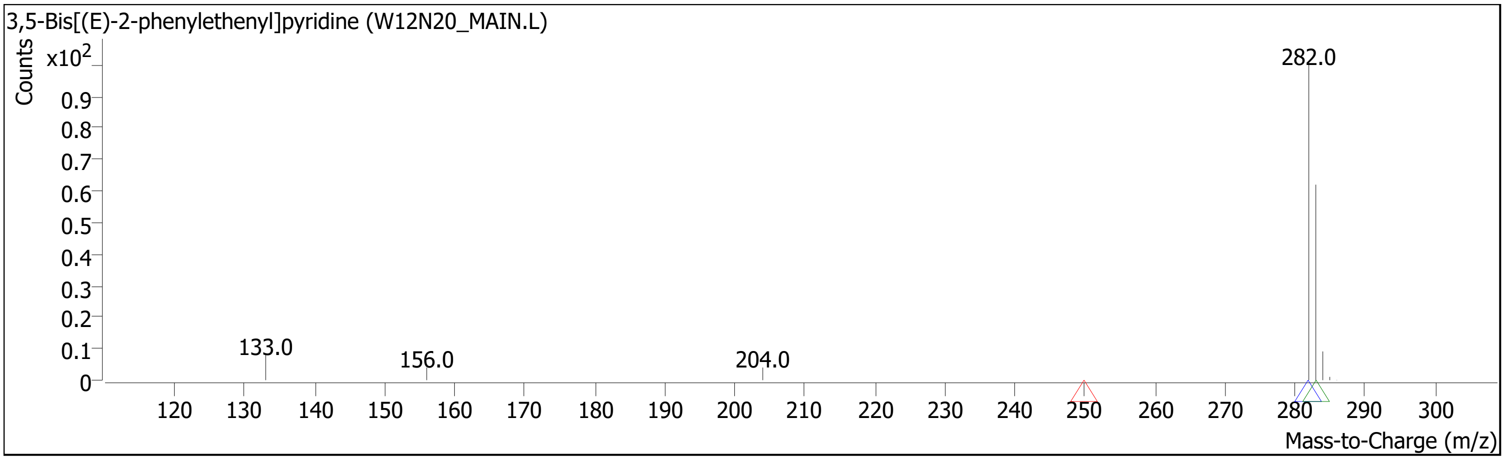

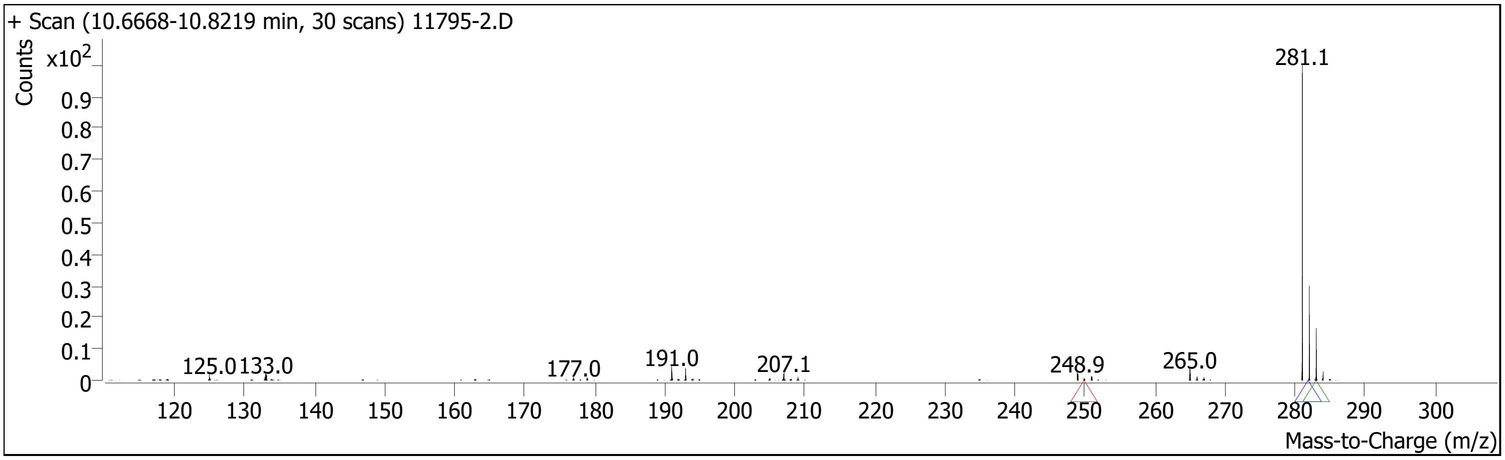

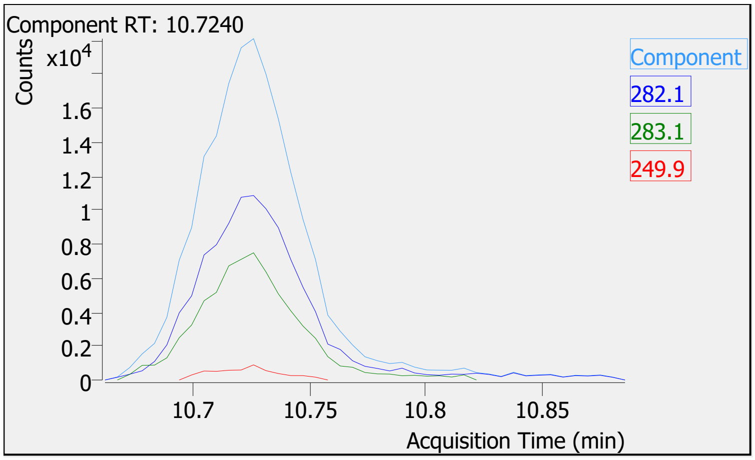

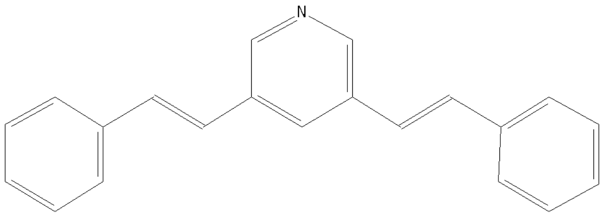

Unknown Analysis Report - Best Hits

RT	Compound Name	CAS#	Formula	Area	MI	Match Score	Sample	Sample
17.1988	2-Propanol, 1-methoxy-	107-98-2	C4H10O2	154280		95.4	1.19	4.76

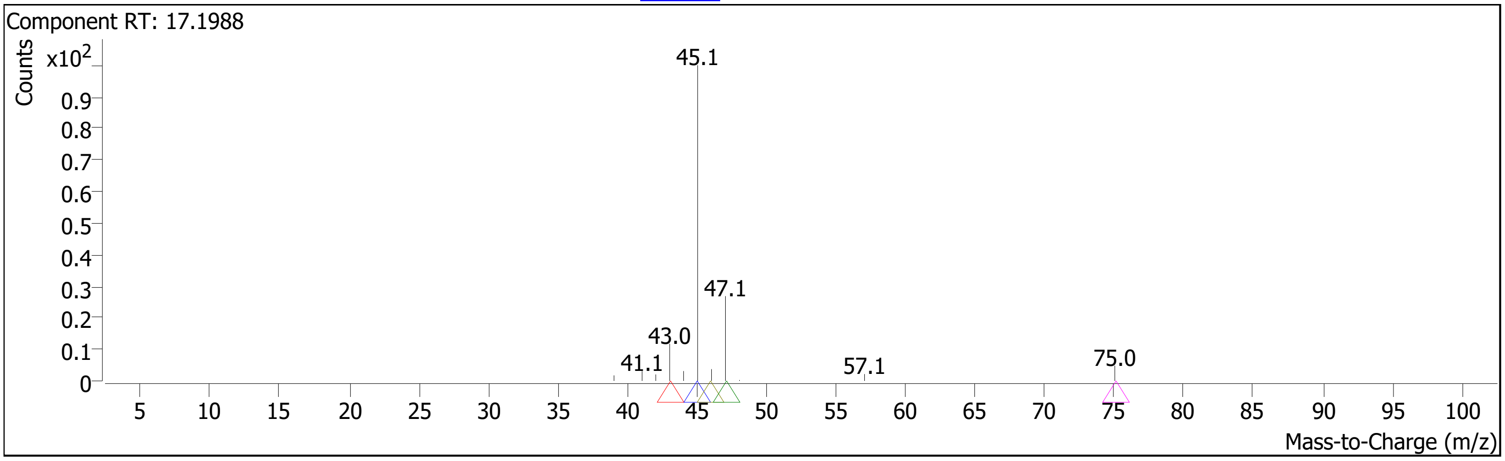

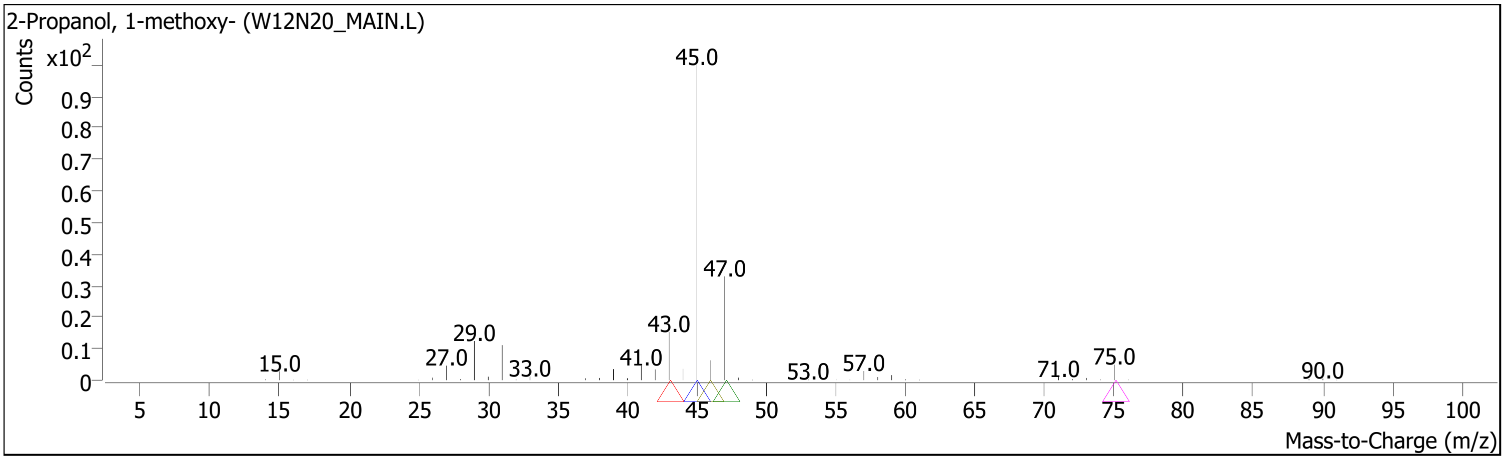

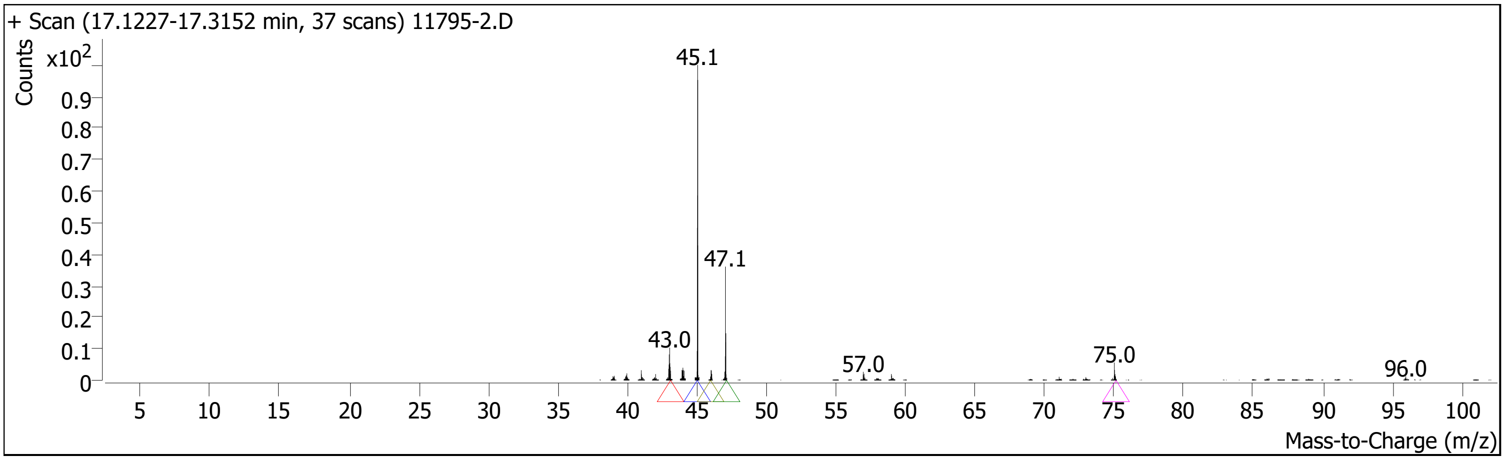

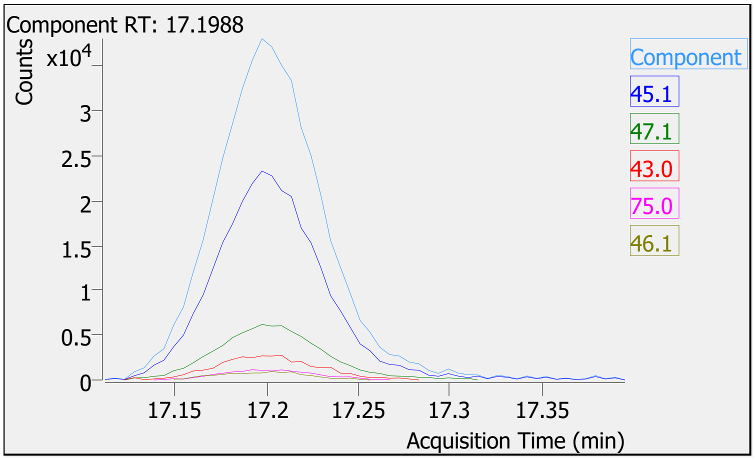

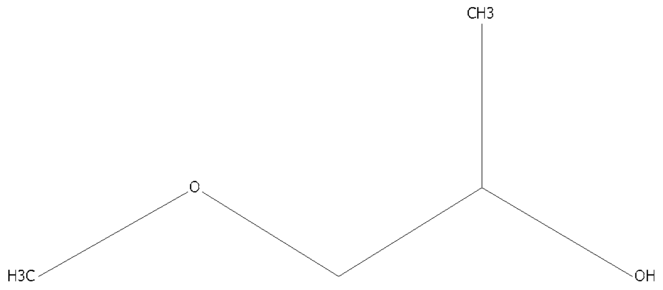

Unknown Analysis Report - Best Hits

RT	Compound Name	CAS#	Formula	Area	MI	Match Score	Sample	Sample
21.6585	Tridecane, 7-hexyl-	7225-66-3	C19H40	44178		99.6	0.34	1.36

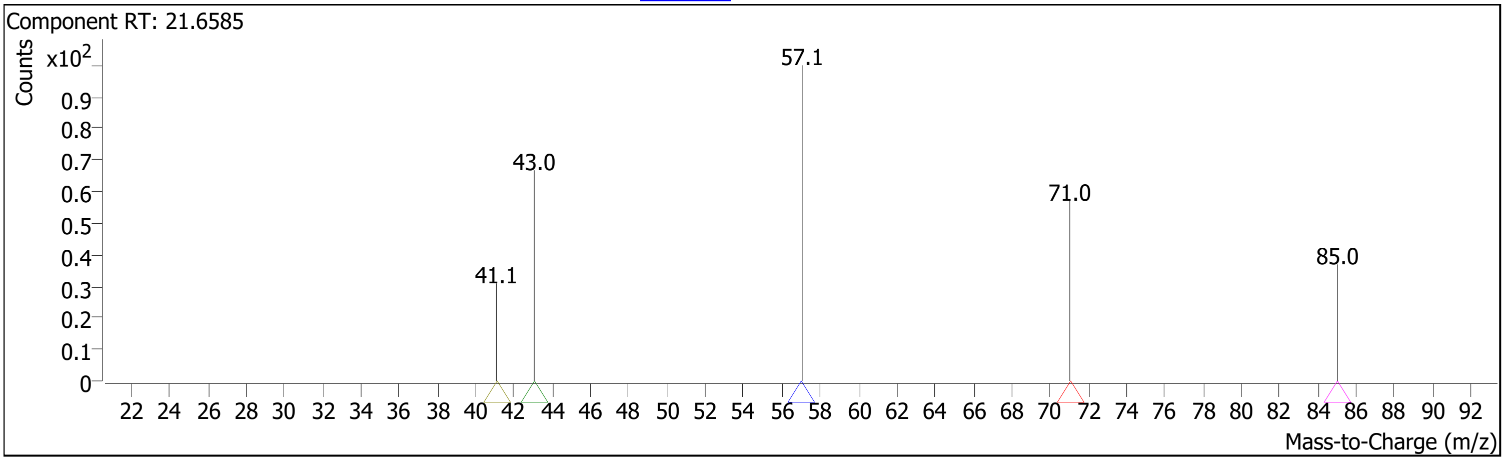

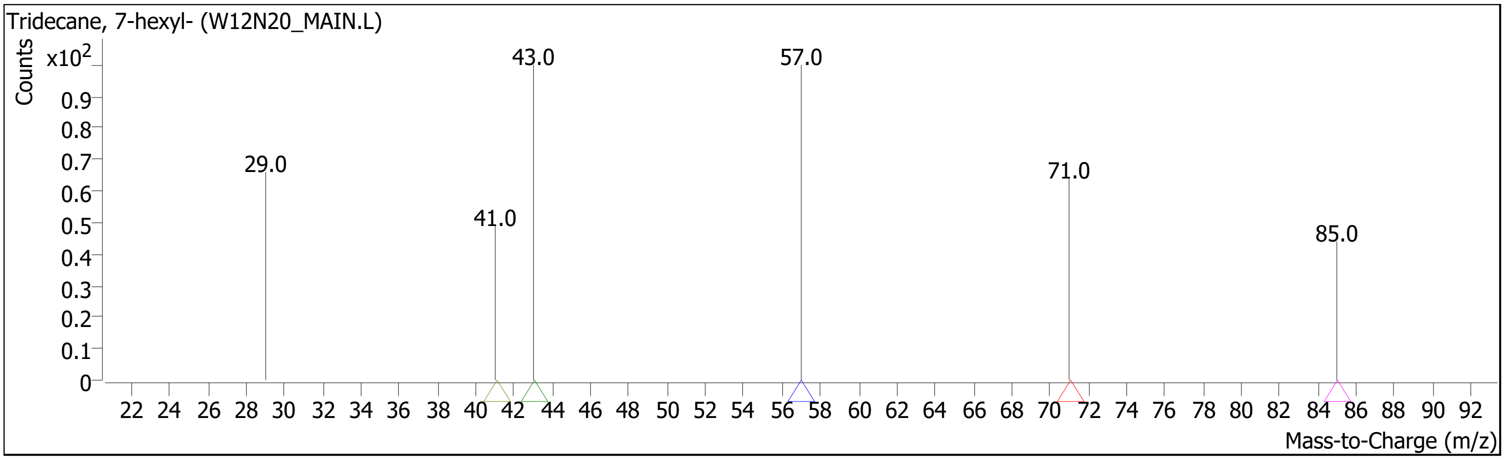

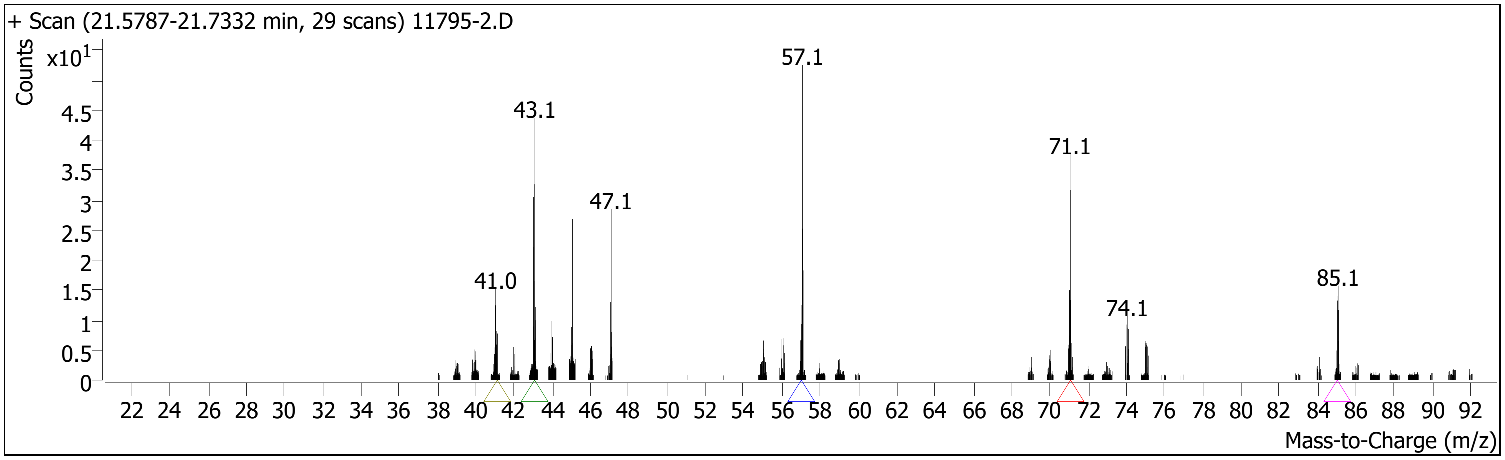

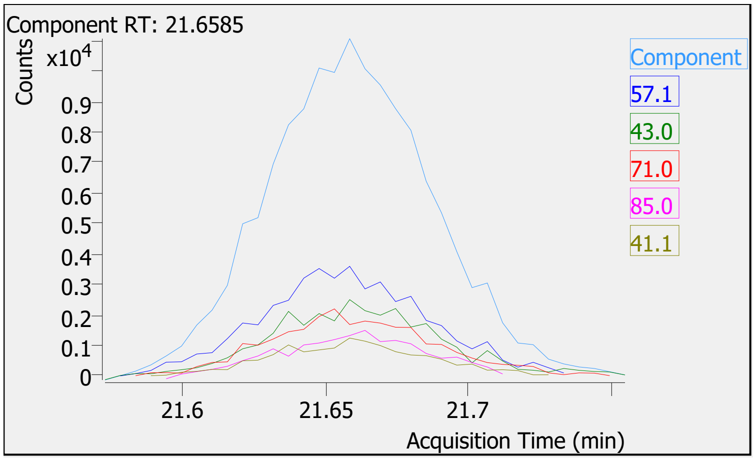

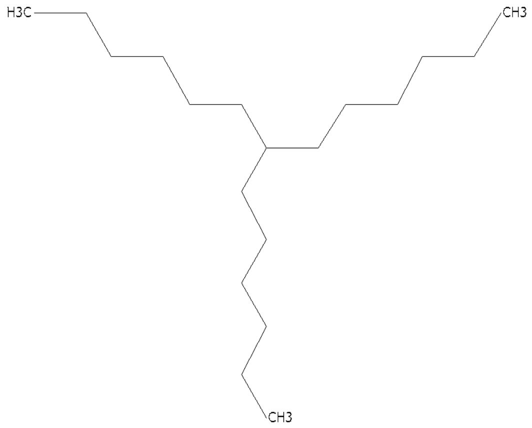

 Generated at 4:32 PM on 11/14/2023

Unknown Analysis Report - Best Hits

RT	Compound Name	CAS#	Formula	Area	MI	Match Score	Sample	Sample
24.4837	2,6,10 - trimethyl - tridecane (without stereochemistry)	3891-99-4	C16H34	100373		85.7	0.77	3.09

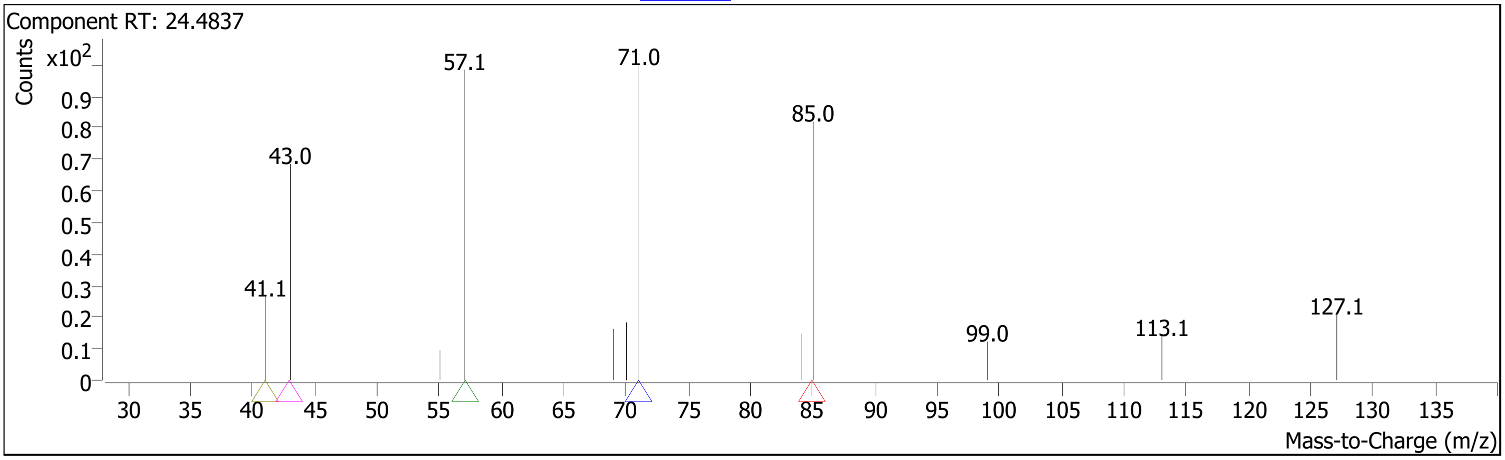

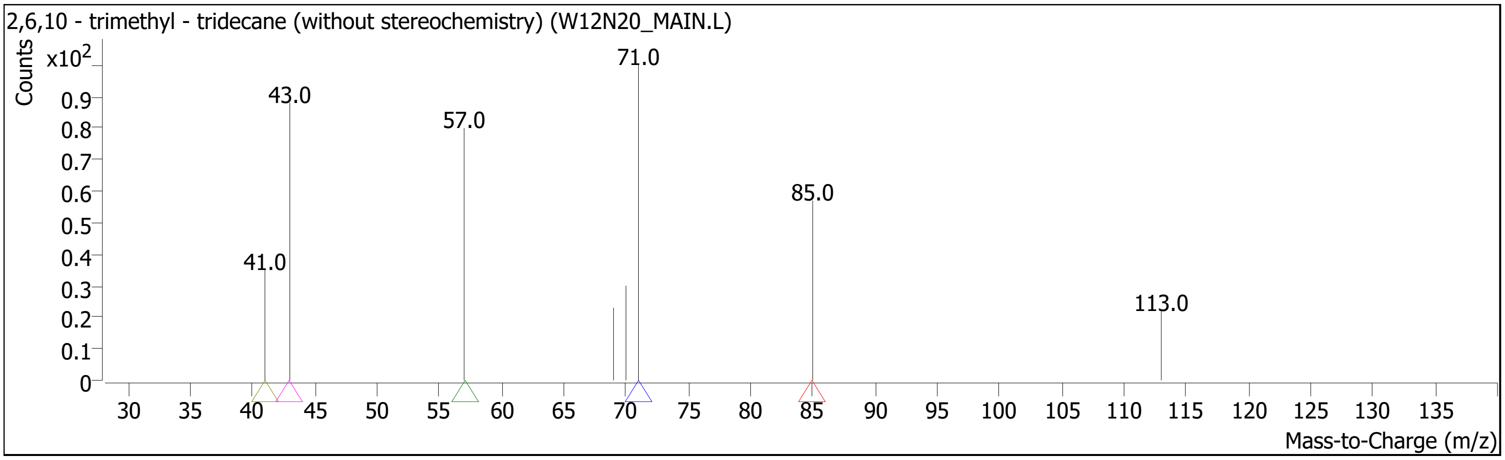

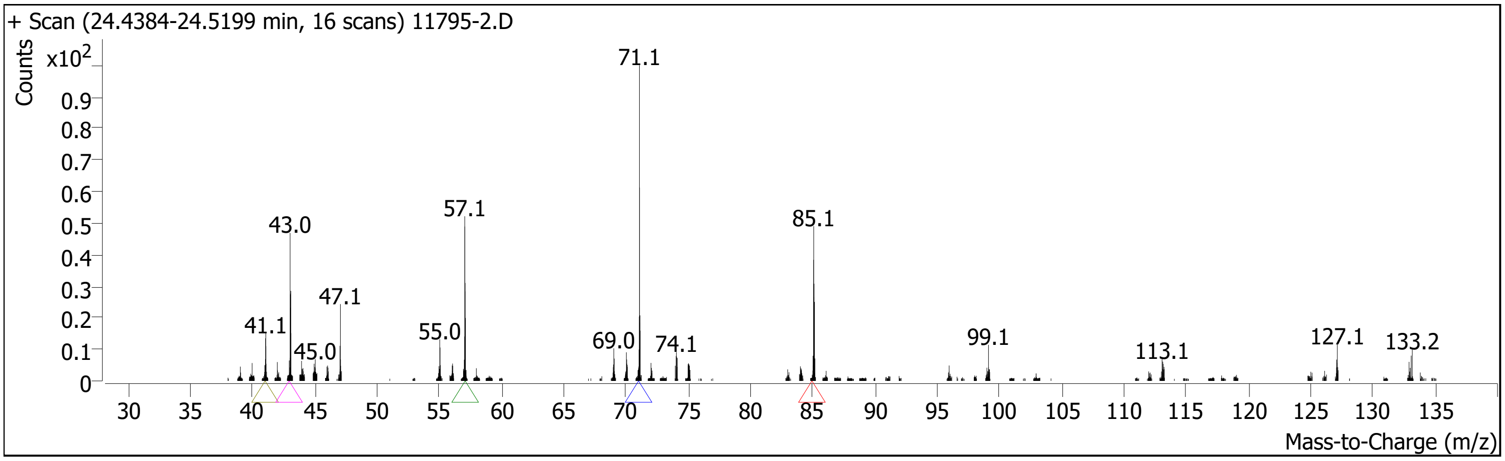

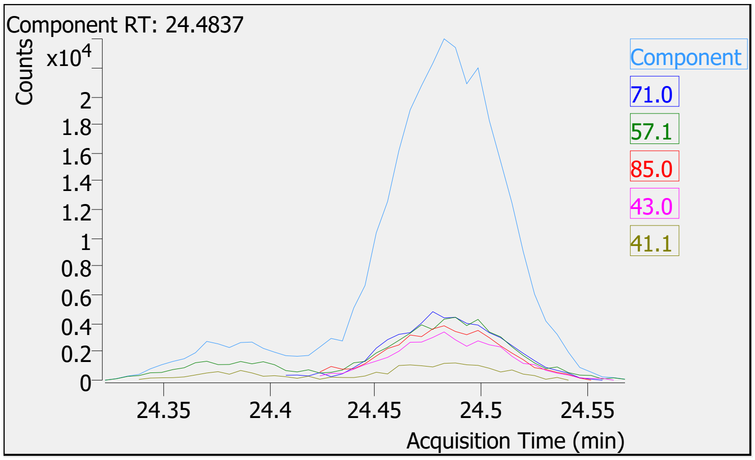

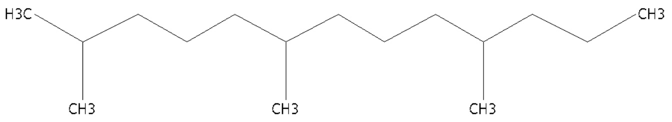

 Generated at 4:32 PM on 11/14/2023

RT	Compound Name	CAS#	Formula	Area	MI	Match Score	Sample	Sample
24.4866	N-Methoxy-N-methyl-2-ethylhexanoylamide	990051-95-9	C10H21NO2	38017		79.1	0.29	1.17

Component RT: 24.4866

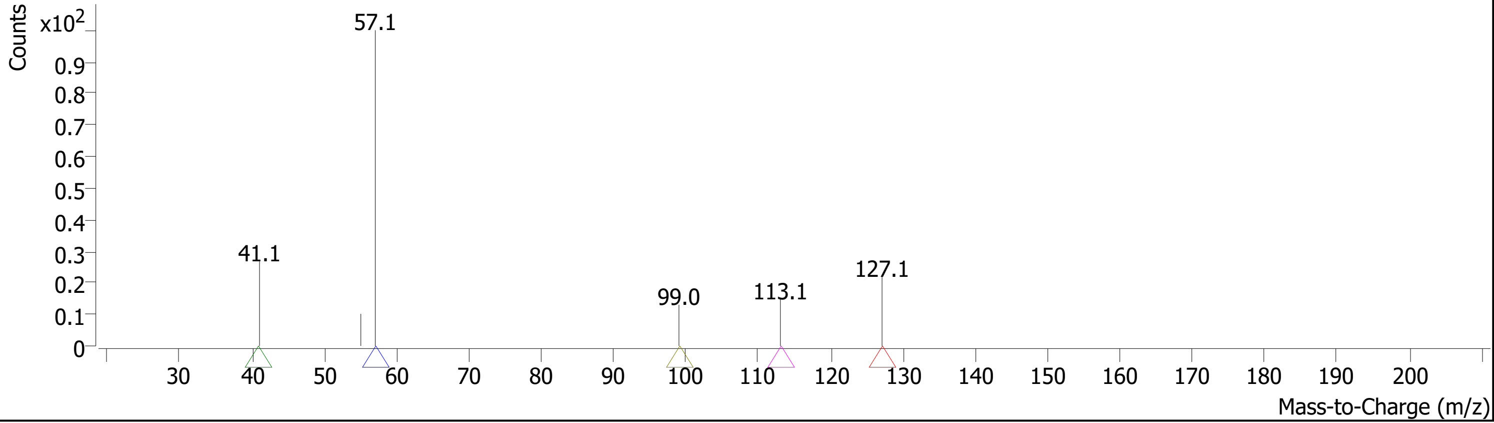

N-Methoxy-N-methyl-2-ethylhexanoylamide (W12N20_MAIN.L)

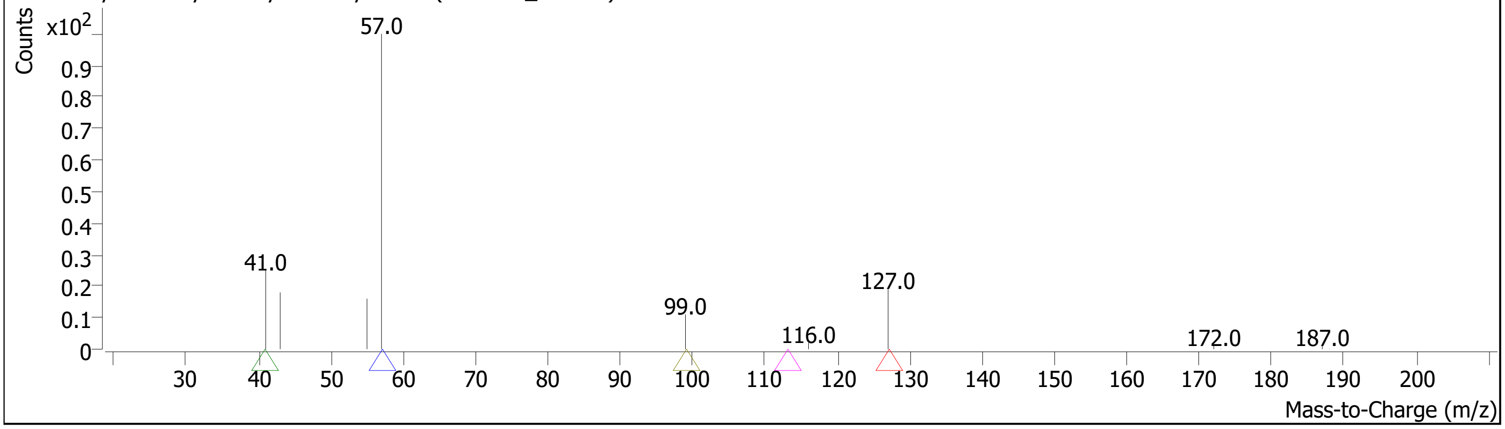

+ Scan (24.4384-24.5199 min, 16 scans) 11795-2.D

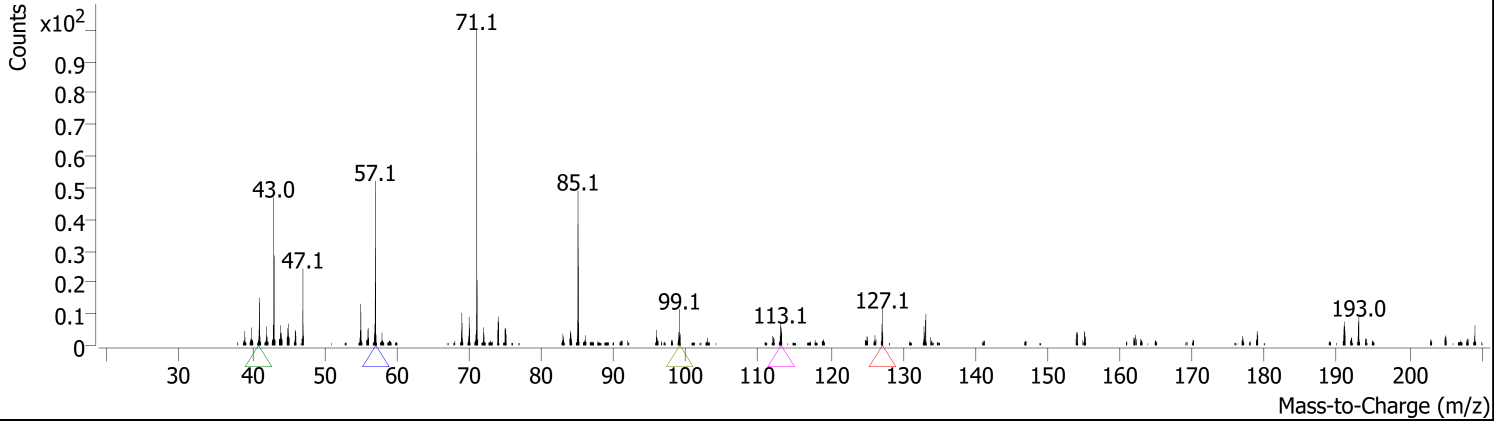

Component RT: 24.4866

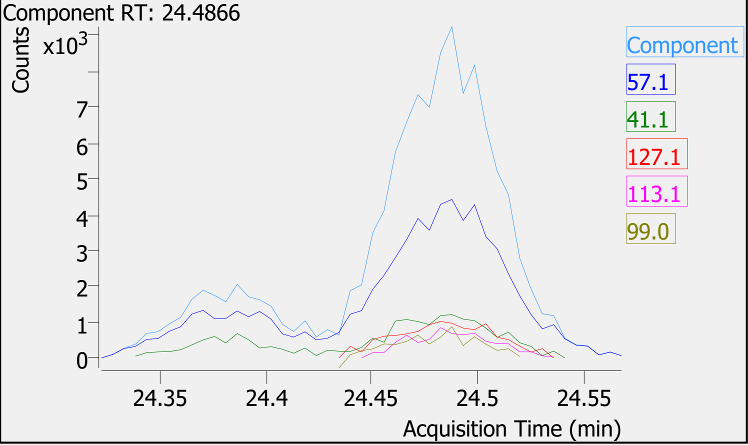

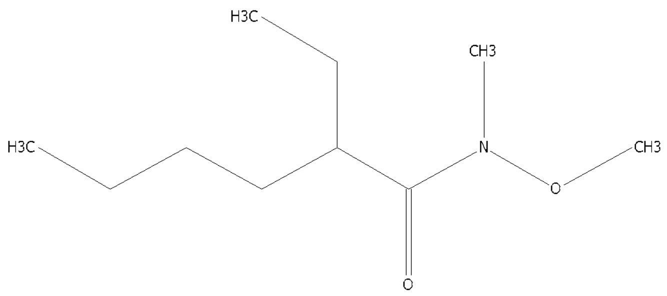

 Generated at 4:32 PM on 11/14/2023

Unknown Analysis Report - Best Hits

RT	Compound Name	CAS#	Formula	Area	MI	Match Score	Sample	Sample
26.4624	Cyclohexane, isocyanato-	3173-53-3	C7H11NO	188265		88.8	1.45	5.80

Component RT: 26.4624

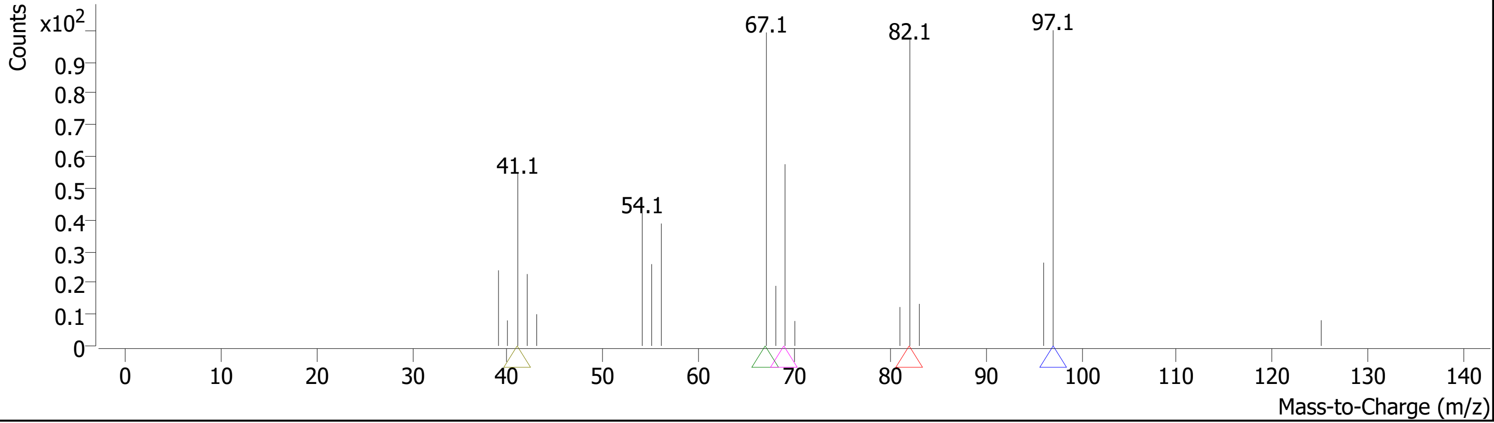

Cyclohexane, isocyanato- (W12N20_MAIN.L)

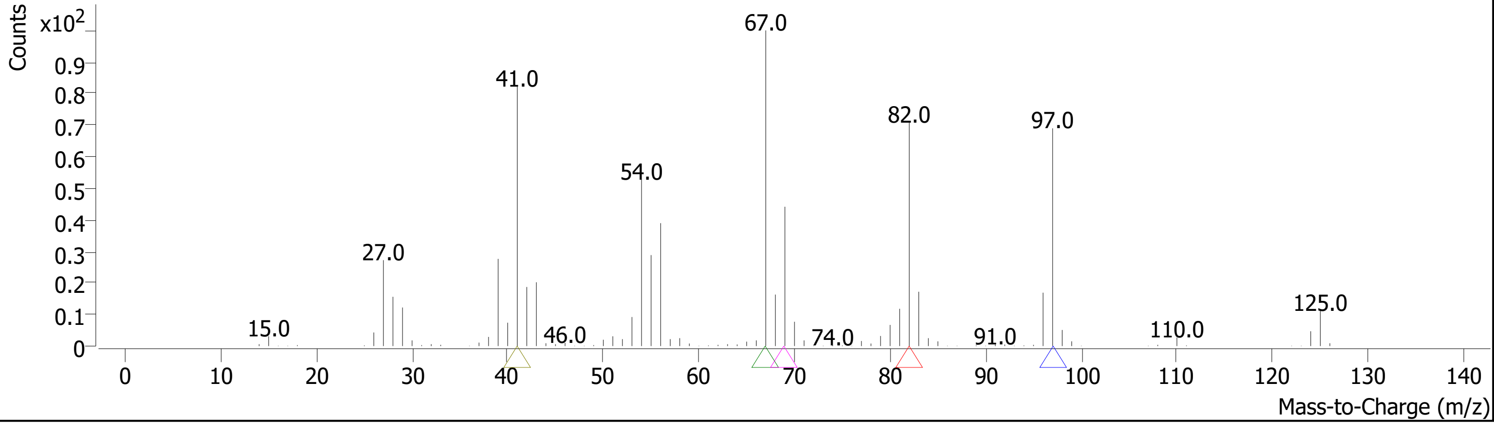

+ Scan (26.3722-26.5621 min, 35 scans) 11795-2.D

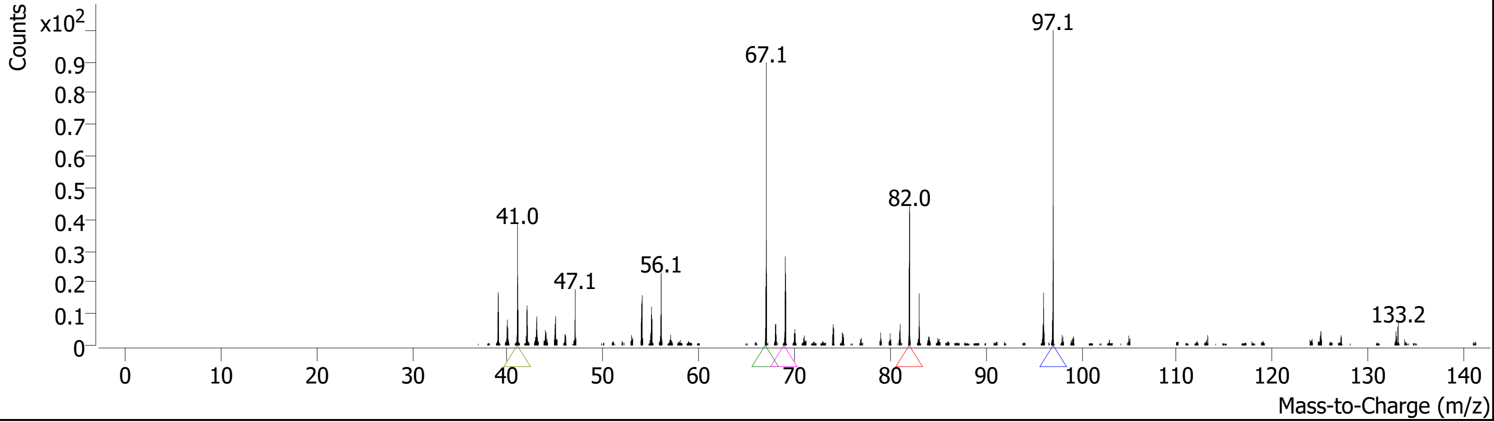

Component RT: 26.4624

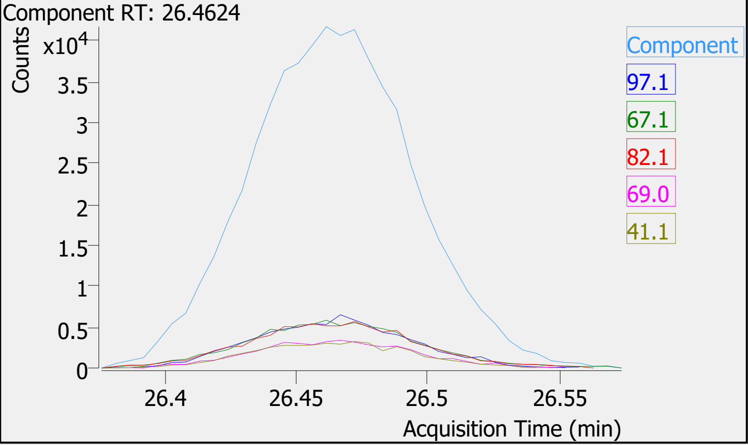

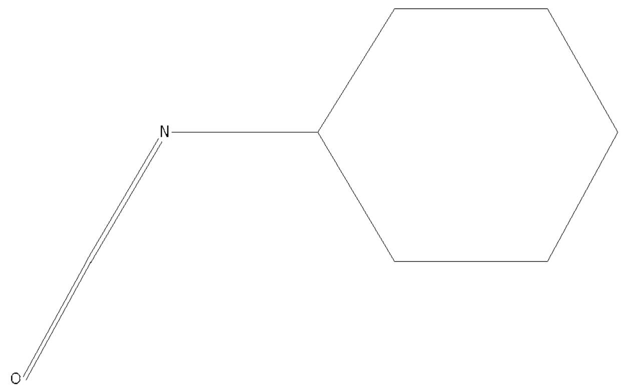

RT	Compound Name	CAS#	Formula	Area	MI	Match Score	Sample	Sample
27.9117	Heptadecane, 2,6,10,15-tetramethyl-	54833-48-6	C21H44	35349		98.9	0.27	1.09

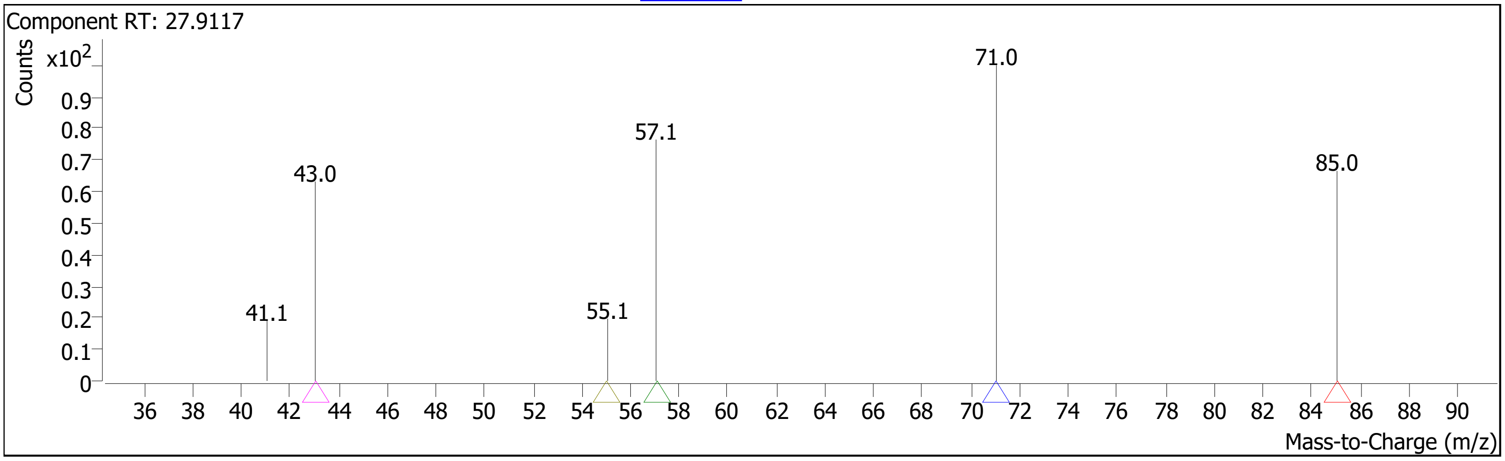

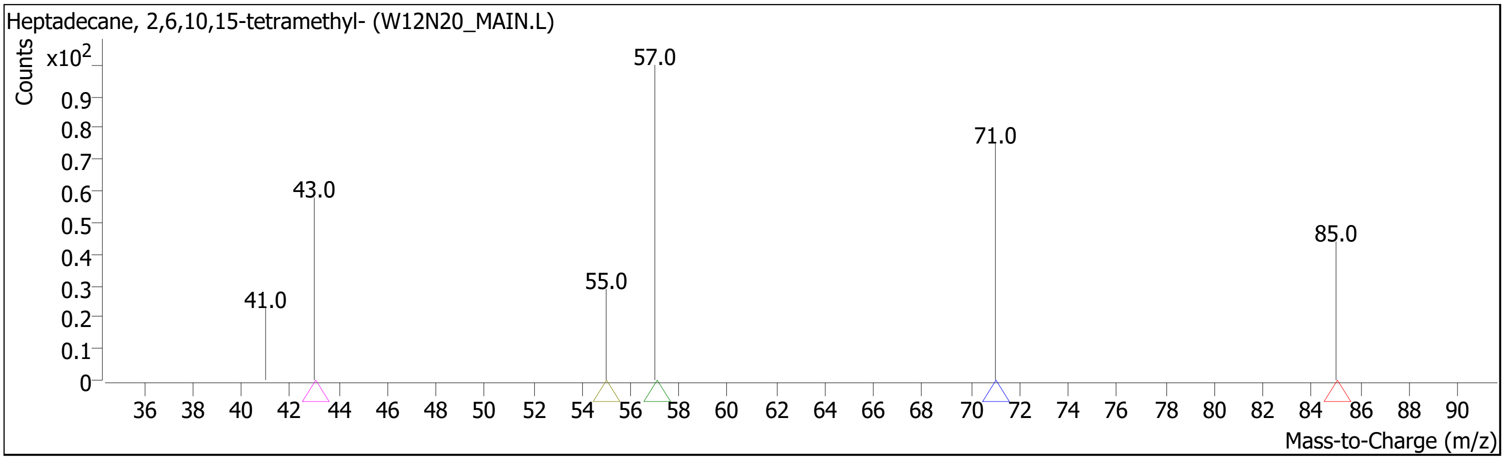

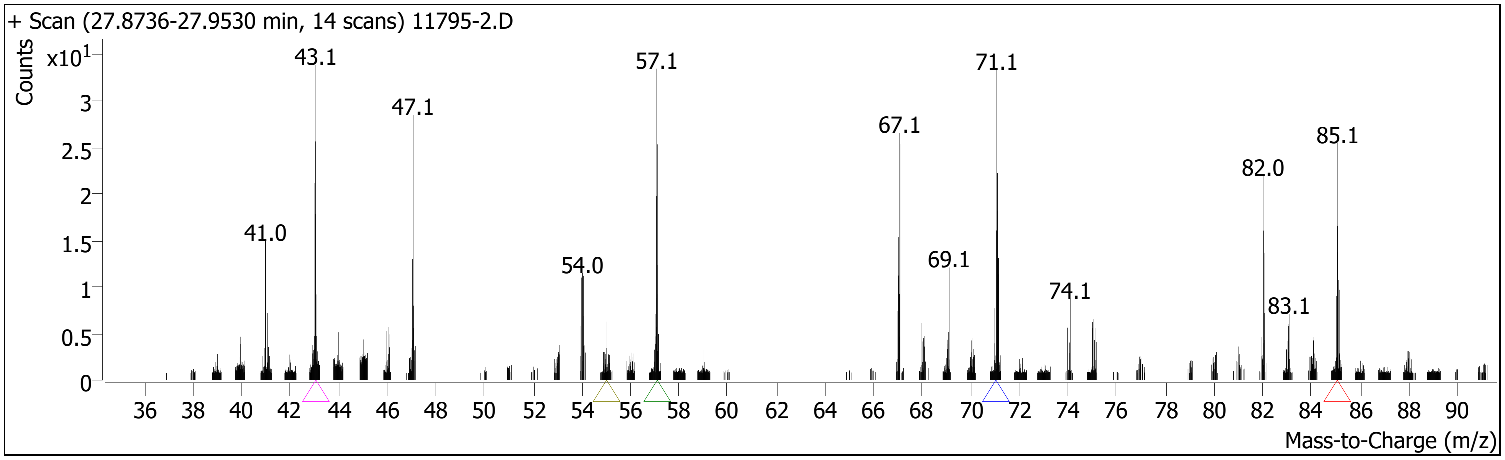

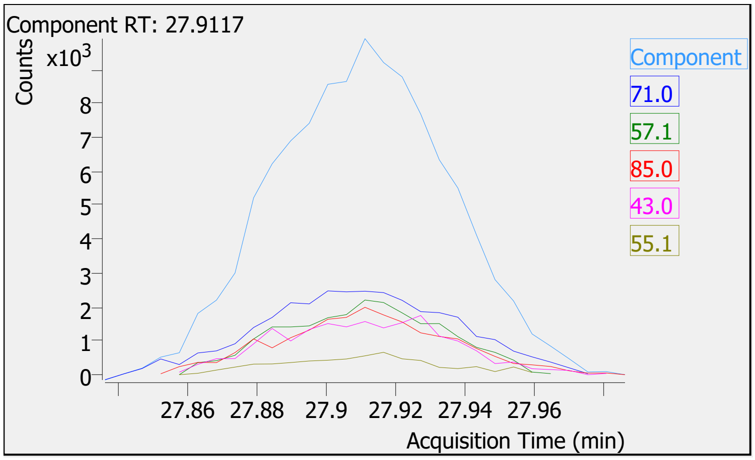

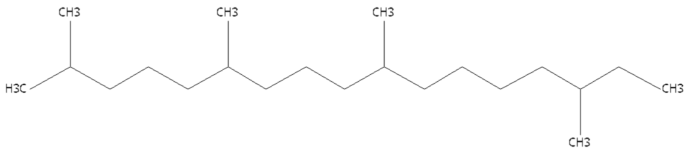

 Generated at 4:32 PM on 11/14/2023

Unknown Analysis Report - Best Hits

RT	Compound Name	CAS#	Formula	Area	MI	Match Score	Sample	Sample
29.6333	1,3-Dioxolane	646-06-0	C3H6O2	31149		83.0	0.24	0.96

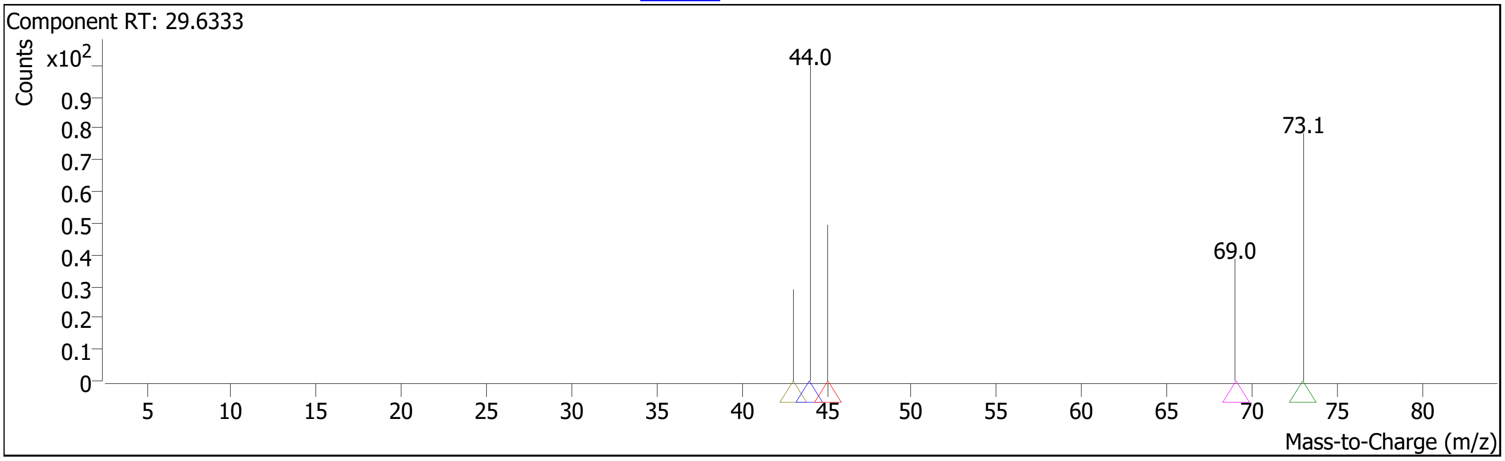

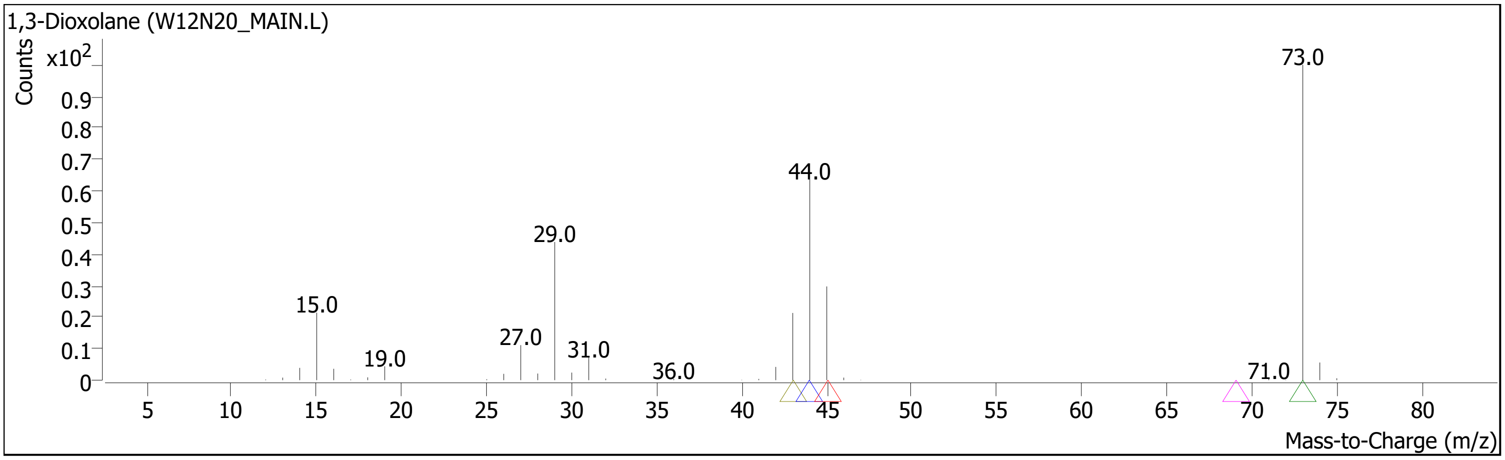

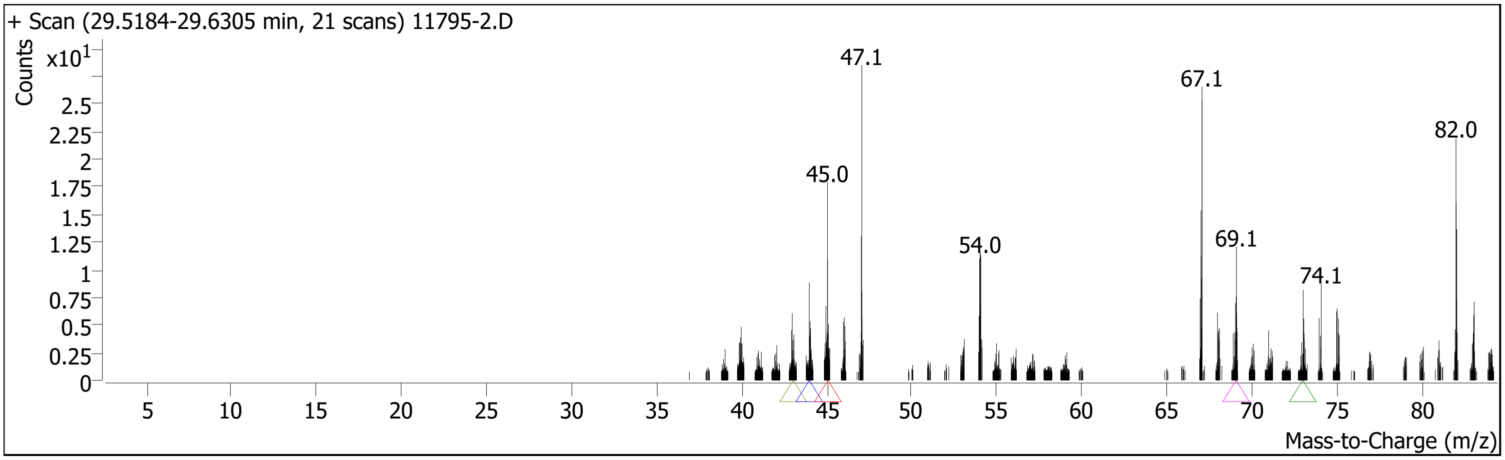

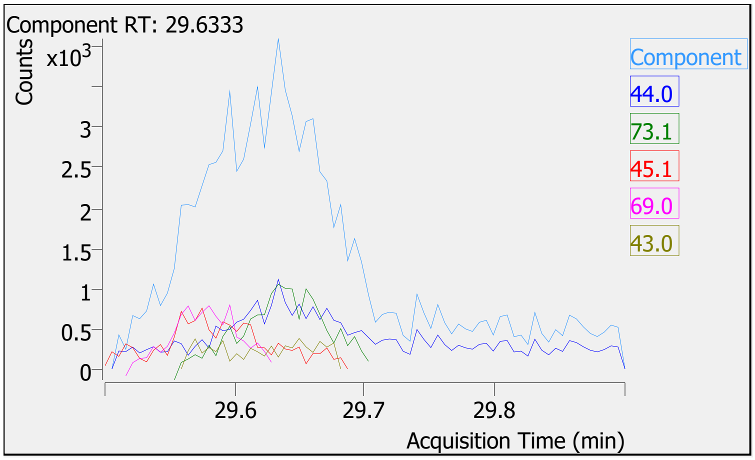

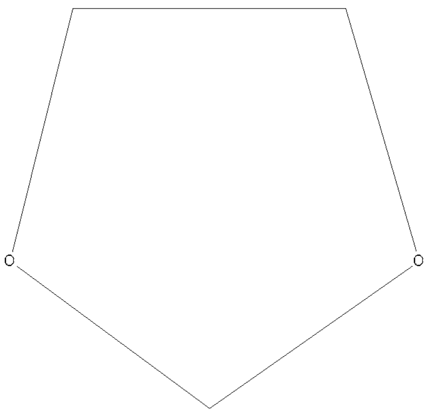

Unknown Analysis Report - Best Hits

RT	Compound Name	CAS#	Formula	Area	MI	Match Score	Sample	Sample
32.8903	Hexadecane, 1-iodo-	544-77-4	C16H33I	13992		90.6	0.11	0.43

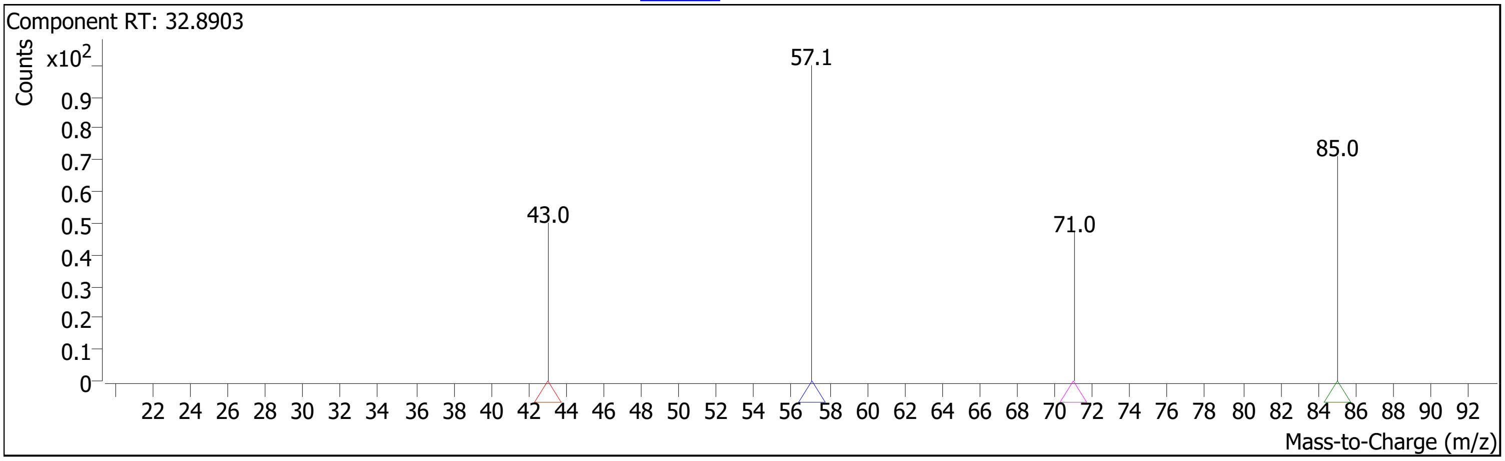

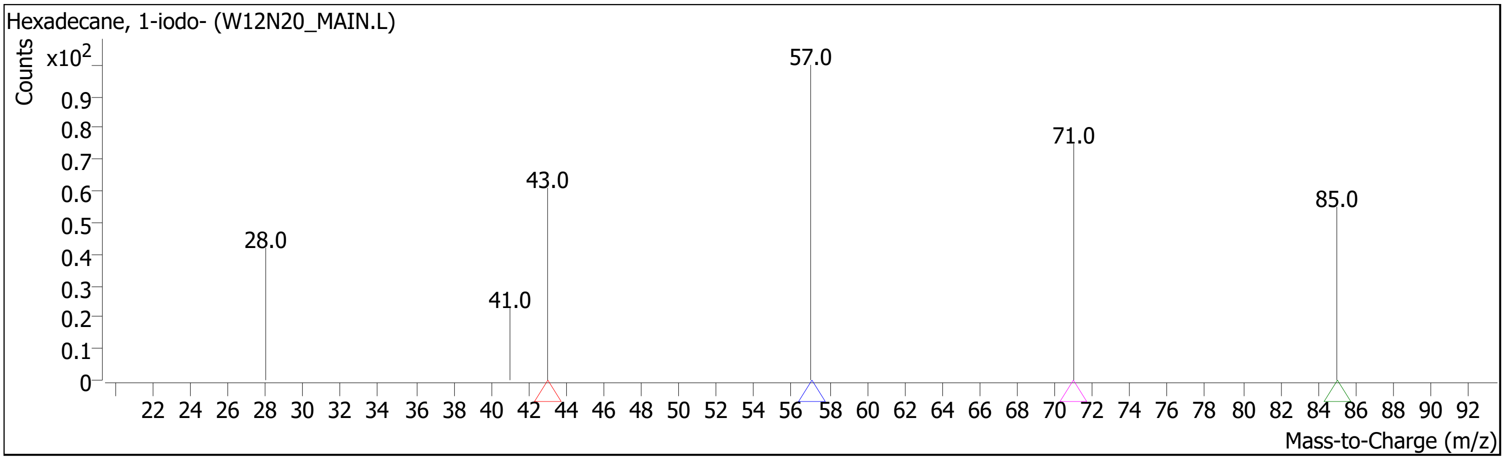

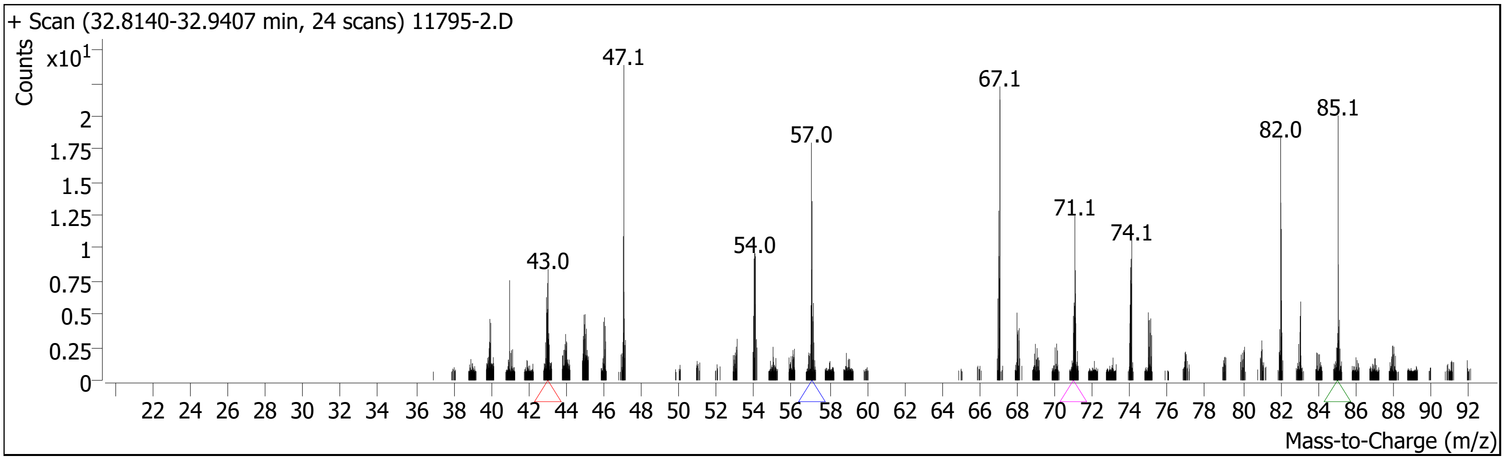

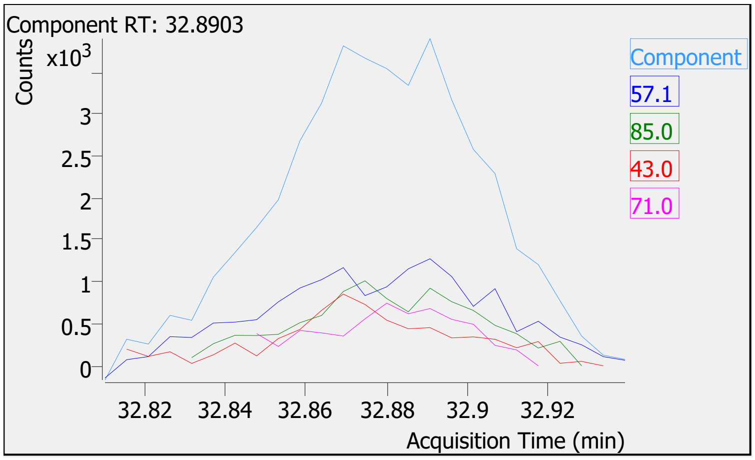

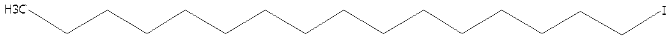

 Generated at 4:32 PM on 11/14/2023

Unknown Analysis Report - Best Hits

RT	Compound Name	CAS#	Formula	Area	MI	Match Score	Sample	Sample
33.9234	Anhydro - sugar	0-00-0	C5H8O4	12534		75.1	0.10	0.39

Component RT: 33.9234

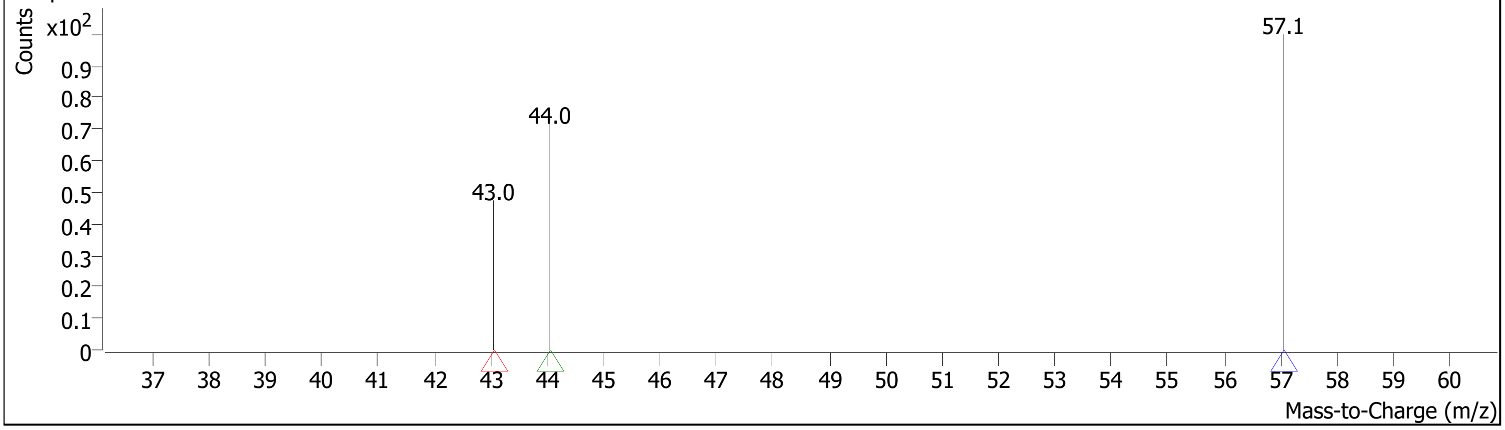

Anhydro - sugar (W12N20_MAIN.L)

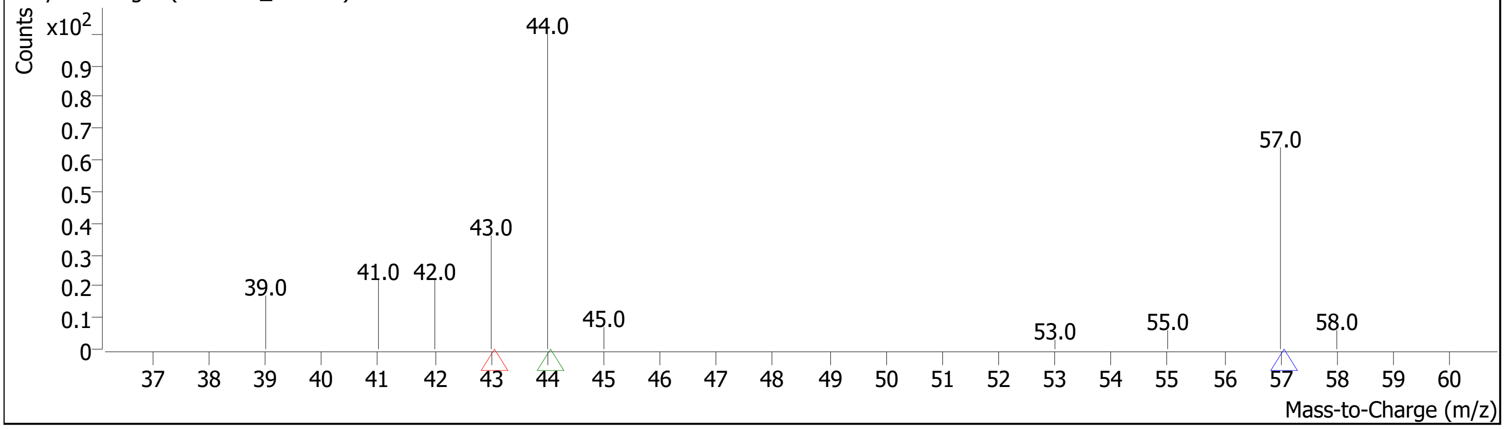

+ Scan (33.8641-33.9978 min, 26 scans) 11795-2.D

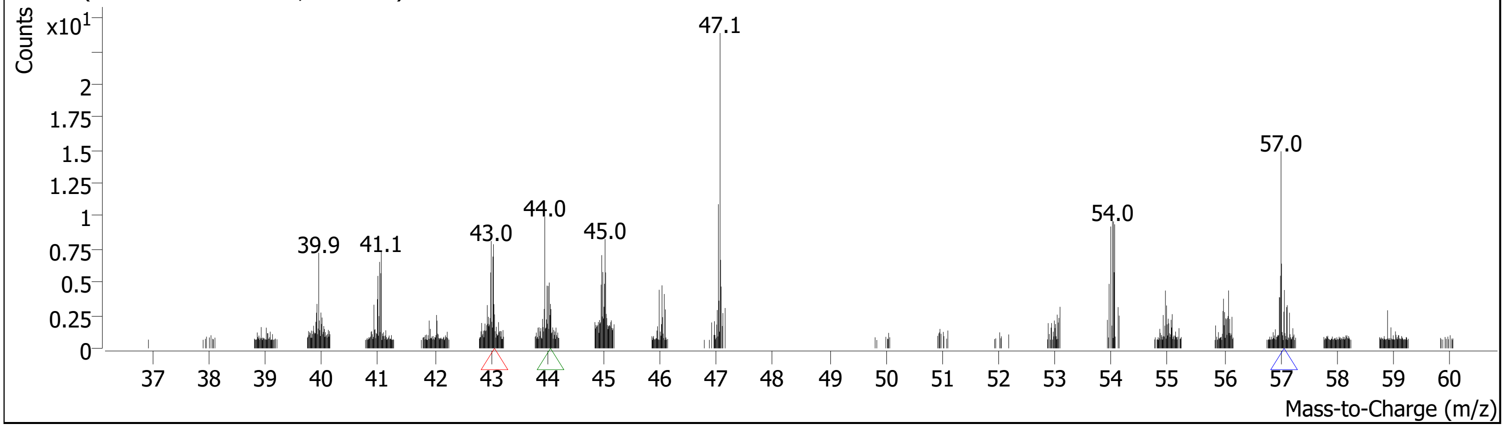

Component RT: 33.9234

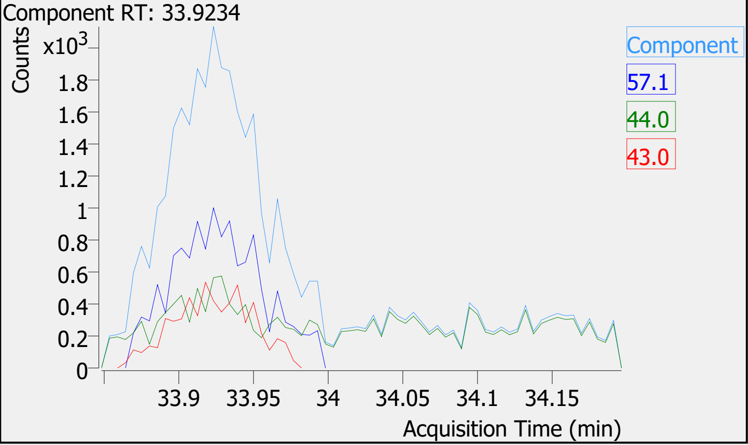

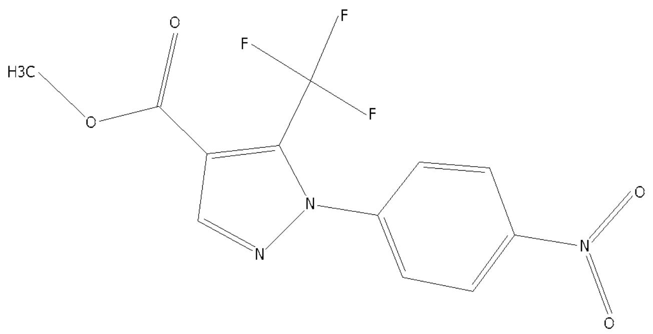

 Generated at 4:32 PM on 11/14/2023

RT	Compound Name	CAS#	Formula	Area	MI	Match Score	Sample	Sample
34.4768	Heptadecane, 2,6,10,15-tetramethyl-	54833-48-6	C21H44	13057		91.1	0.10	0.40

Component RT: 34.4768

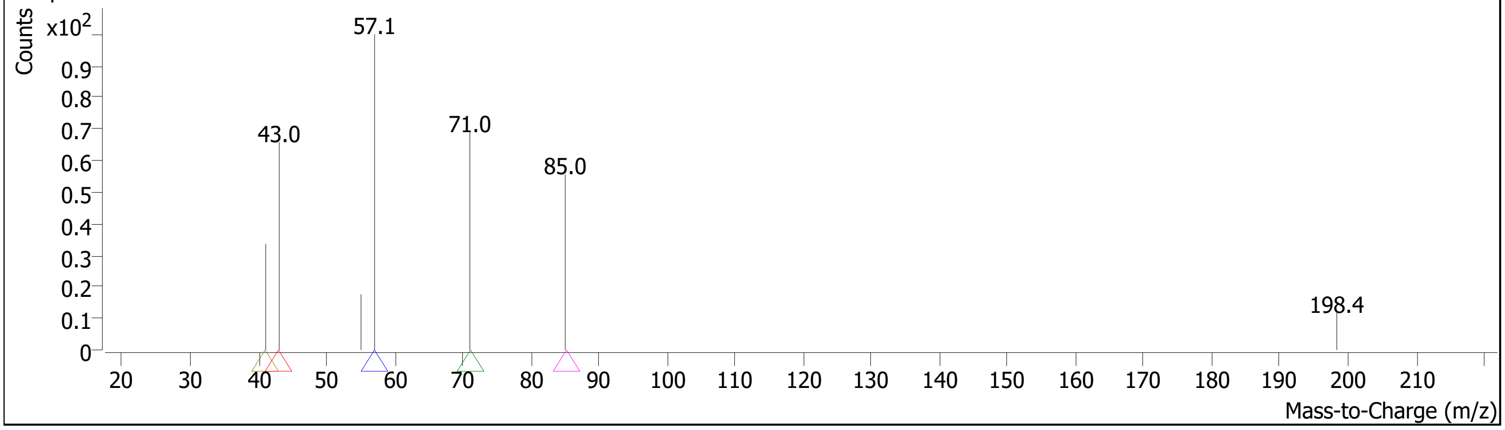

Heptadecane, 2,6,10,15-tetramethyl- (W12N20_MAIN.L)

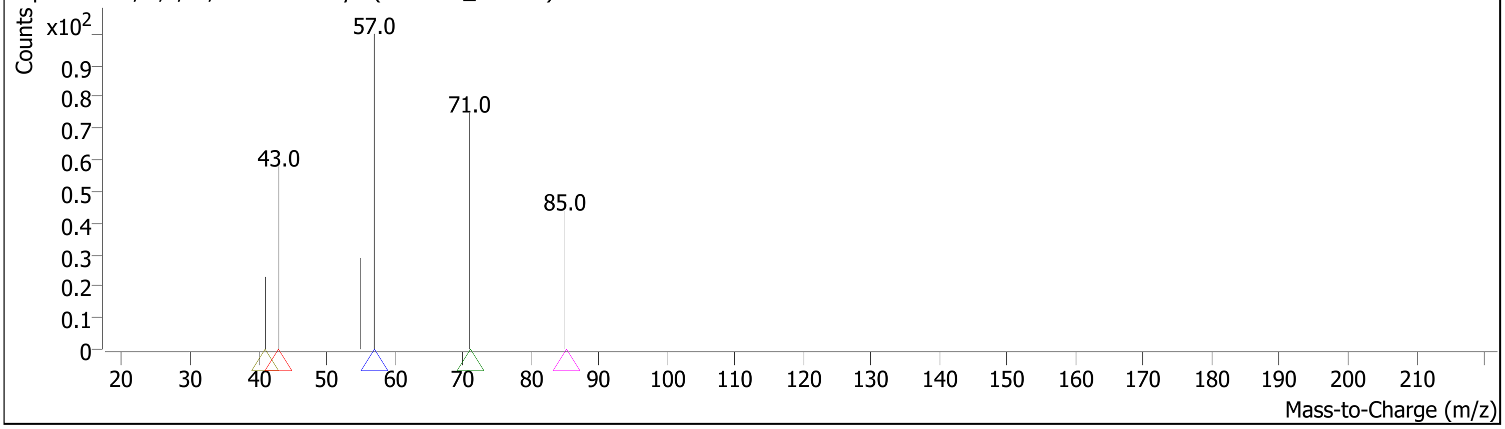

+ Scan (34.4525-34.4846 min, 7 scans) 11795-2.D

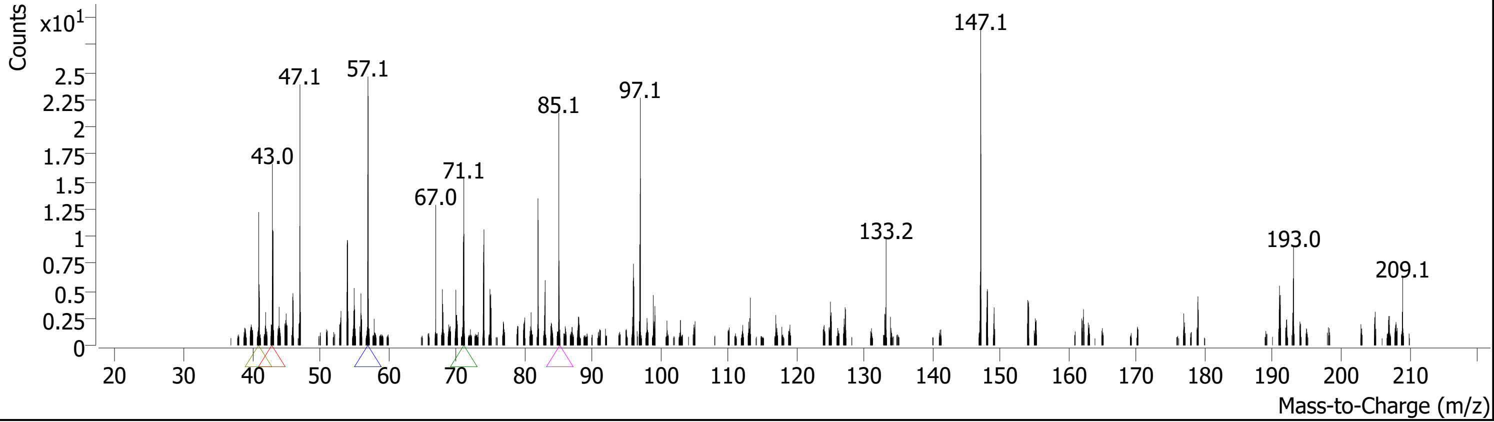

Component RT: 34.4768

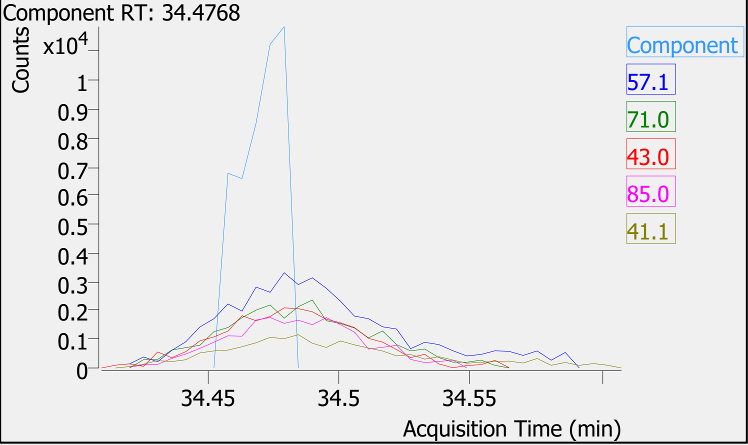

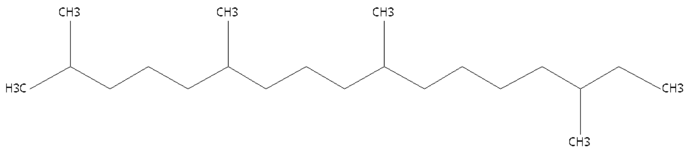

RT	Compound Name	CAS#	Formula	Area	MI	Match Score	Sample	Sample
36.1422	1,2-Di-tert-butylbenzene	1012-76-6	C14H22	84507		90.1	0.65	2.61

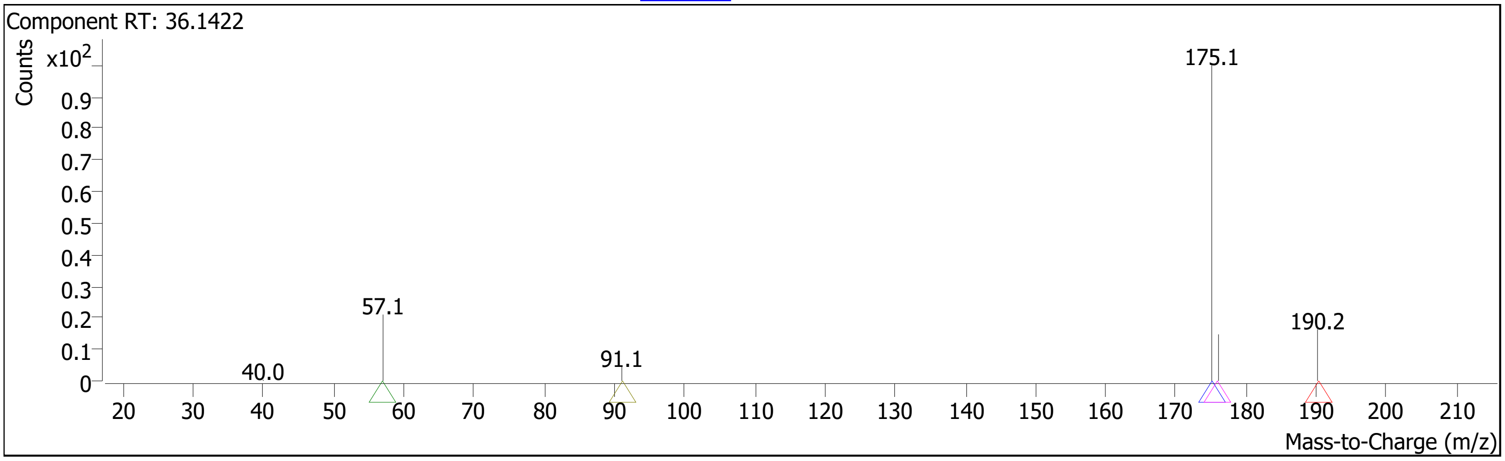

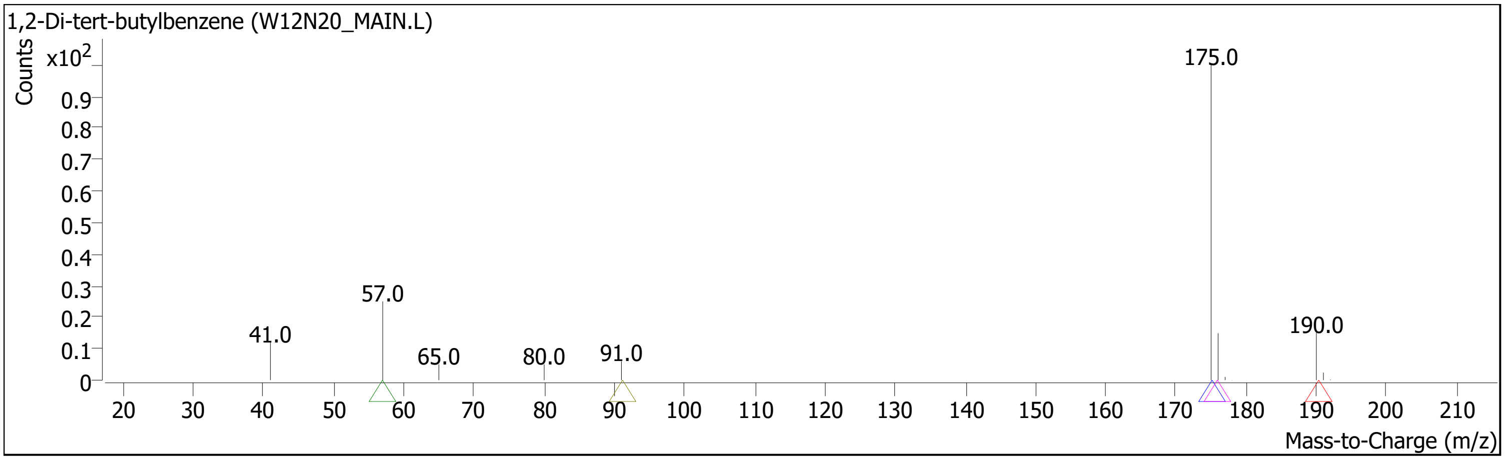

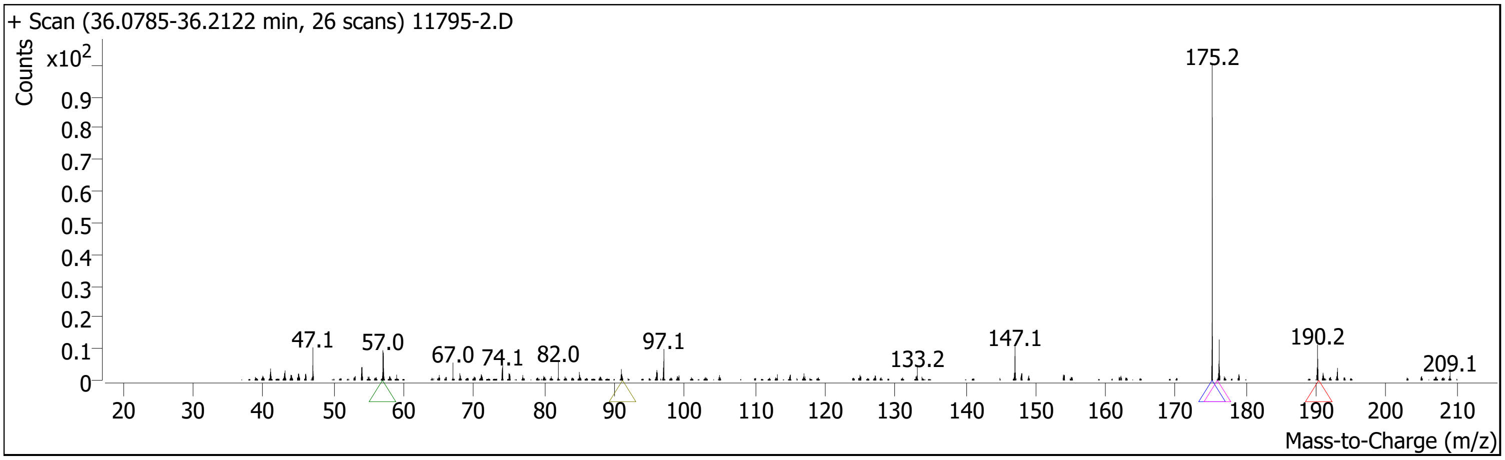

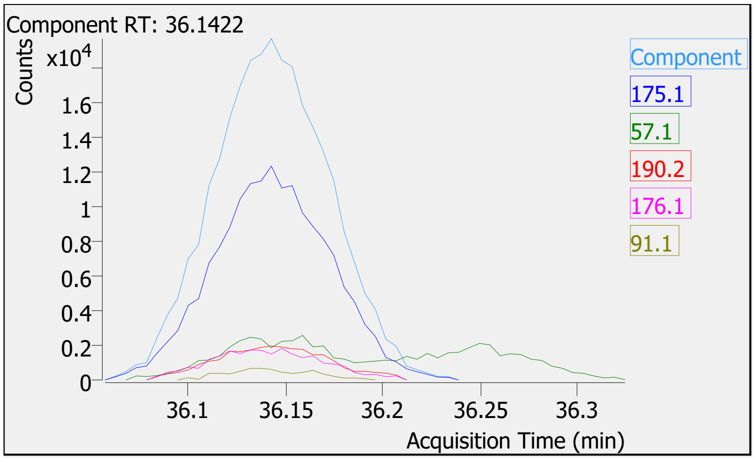

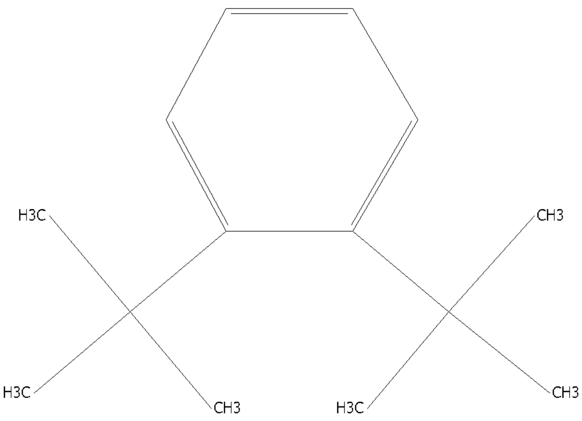

 Generated at 4:32 PM on 11/14/2023

Unknown Analysis Report - Best Hits

RT	Compound Name	CAS#	Formula	Area	MI	Match Score	Sample	Sample
36.2431	2,6-Nonanedione	36452-81-0	C9H16O2	16311		80.5	0.13	0.50

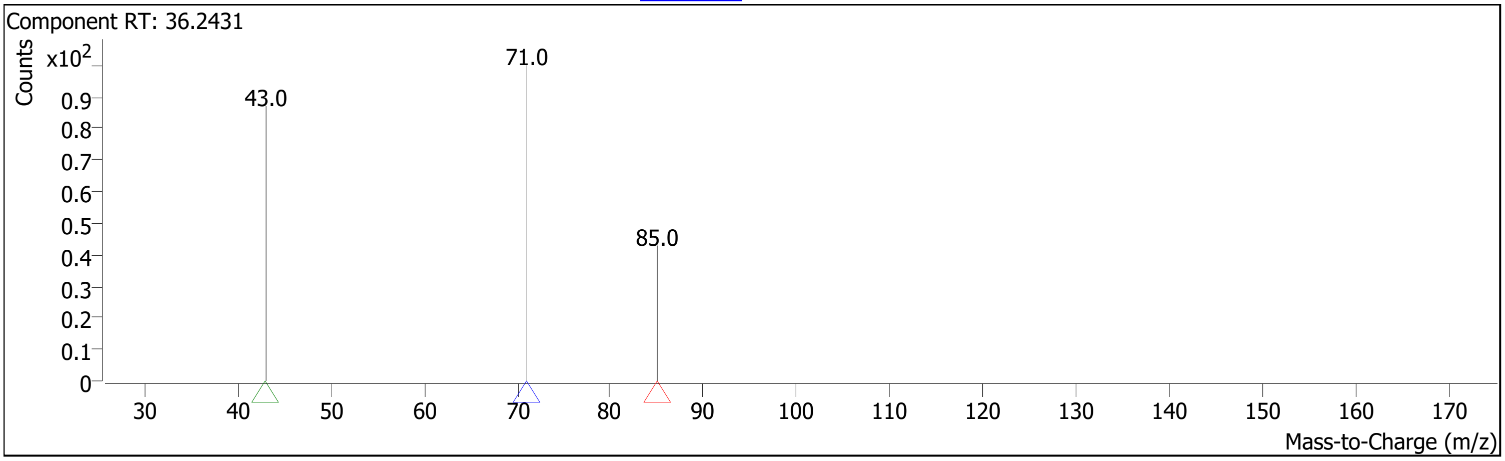

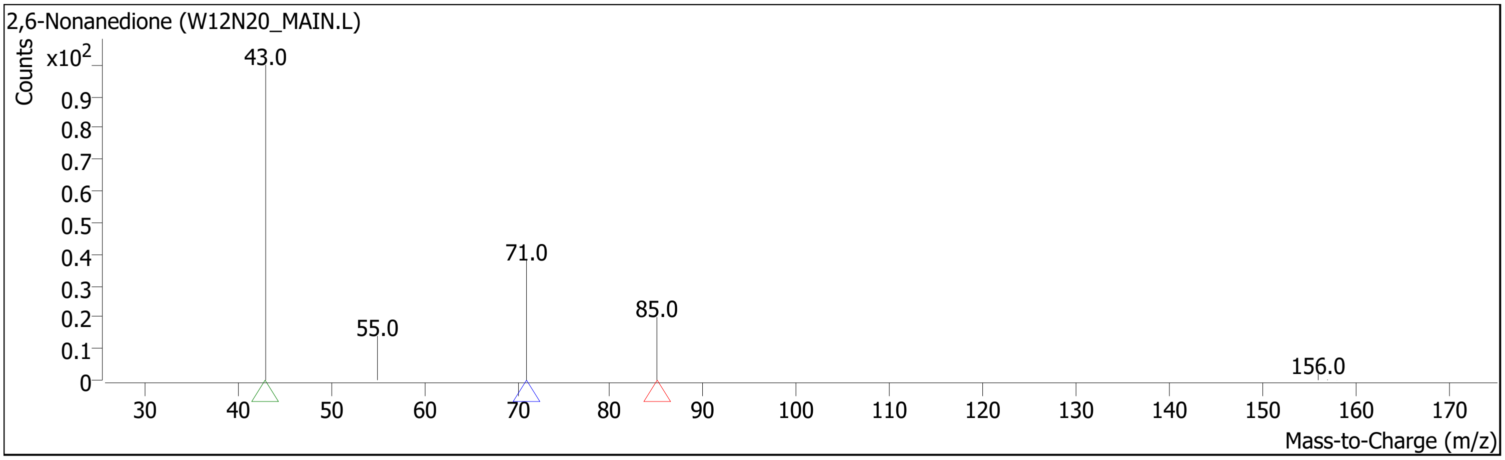

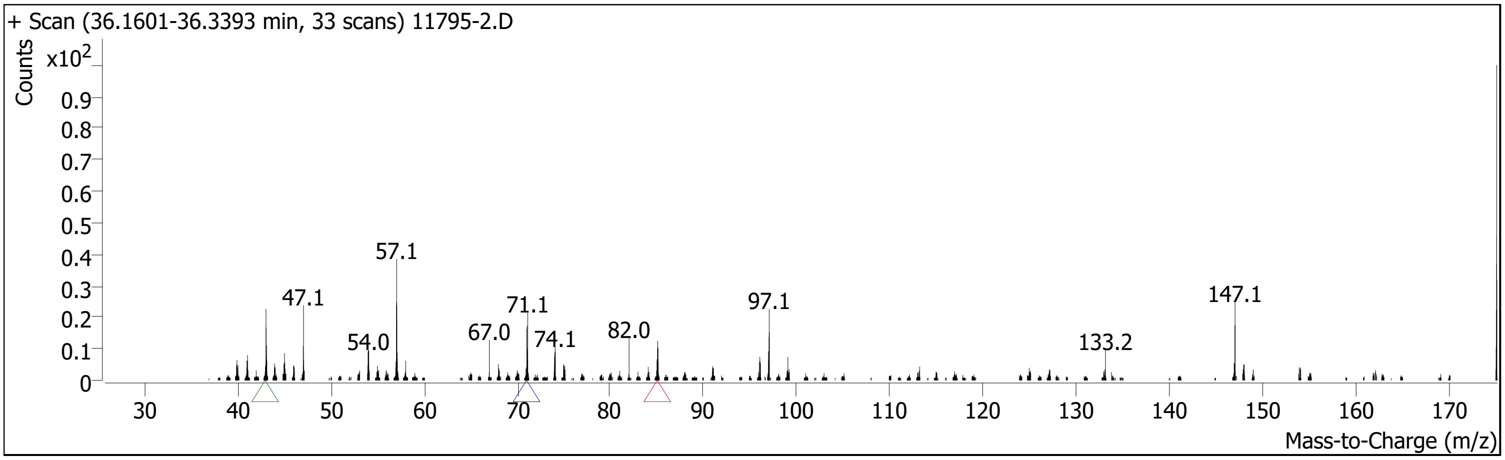

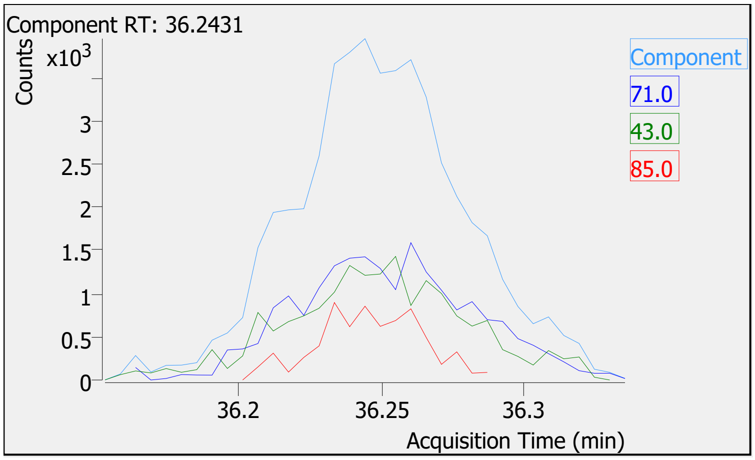

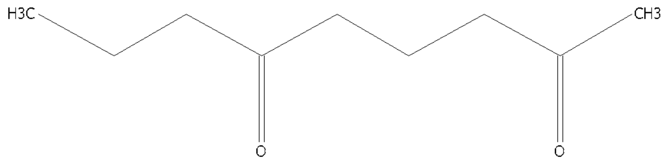

 Generated at 4:32 PM on 11/14/2023

RT	Compound Name	CAS#	Formula	Area	MI	Match Score	Sample	Sample
37.2537	Decane, 3-ethyl-3-methyl-	17312-66-2	C13H28	116361		87.3	0.89	3.59

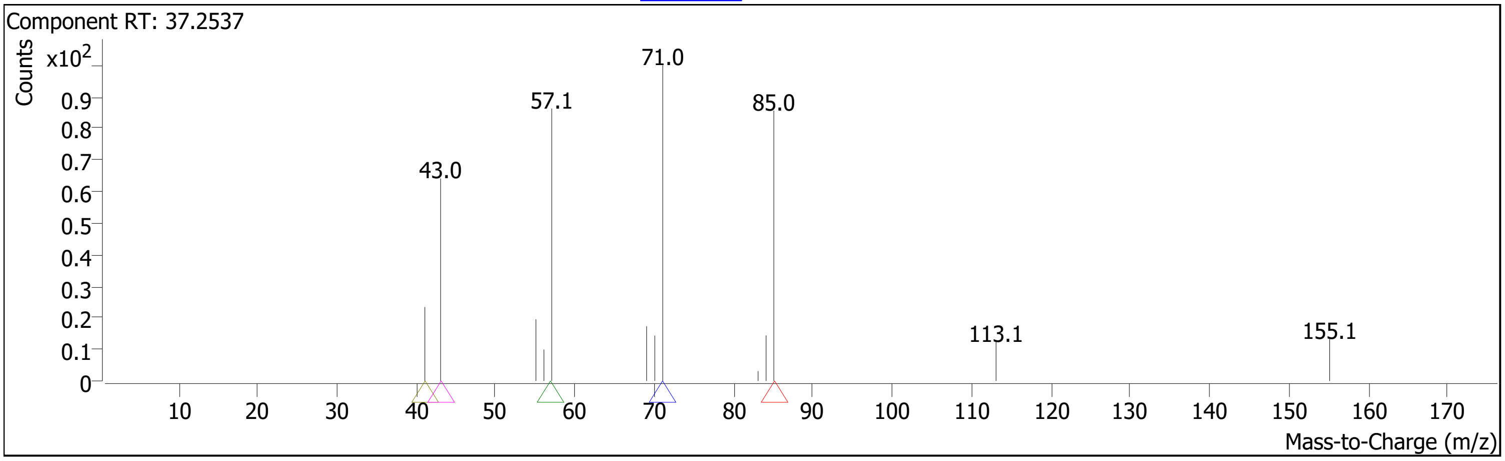

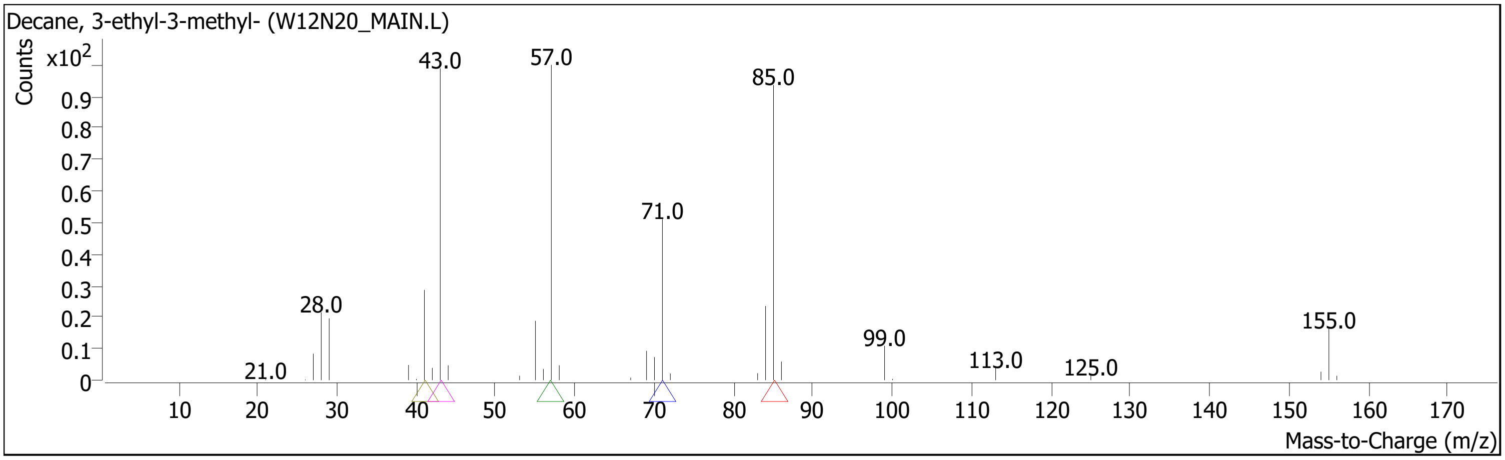

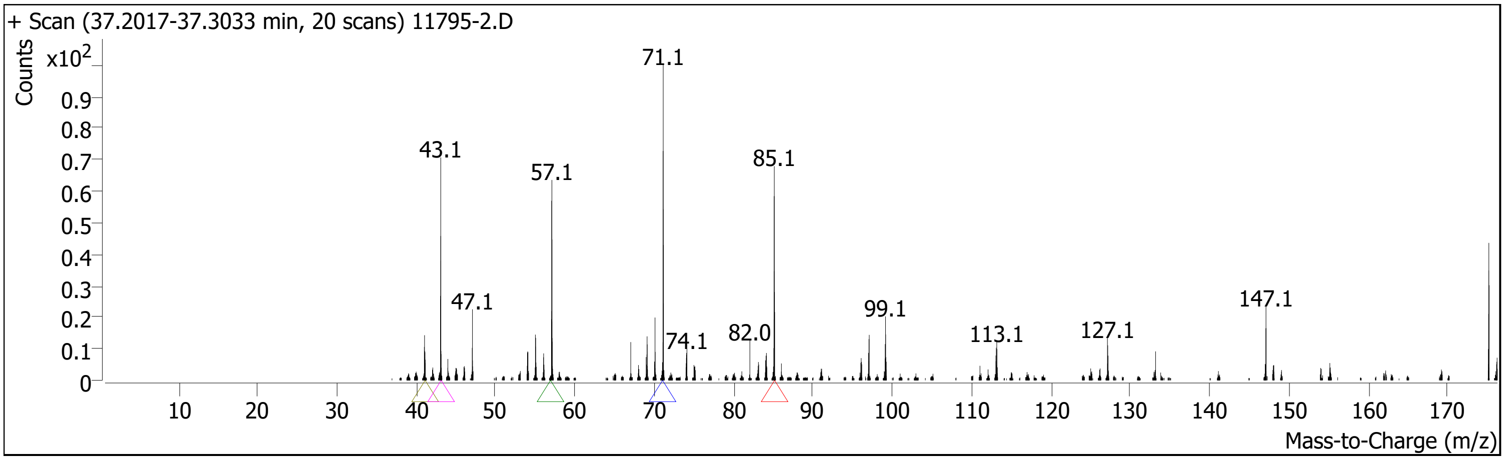

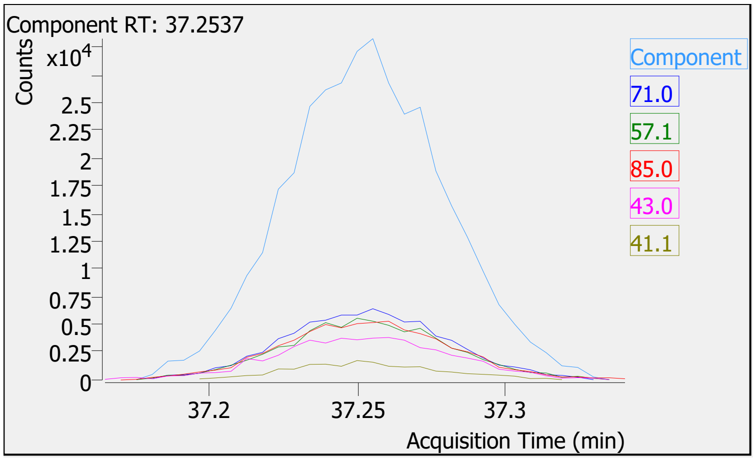

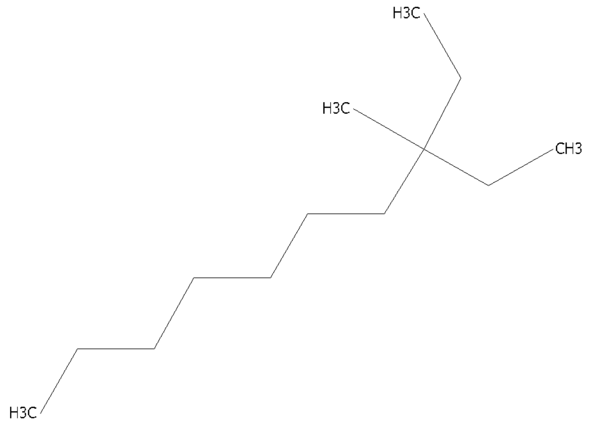

 Generated at 4:32 PM on 11/14/2023

RT	Compound Name	CAS#	Formula	Area	MI	Match Score	Sample	Sample
37.8255	Hexadecane, 1-iodo-	544-77-4	C16H33I	32347		91.0	0.25	1.00

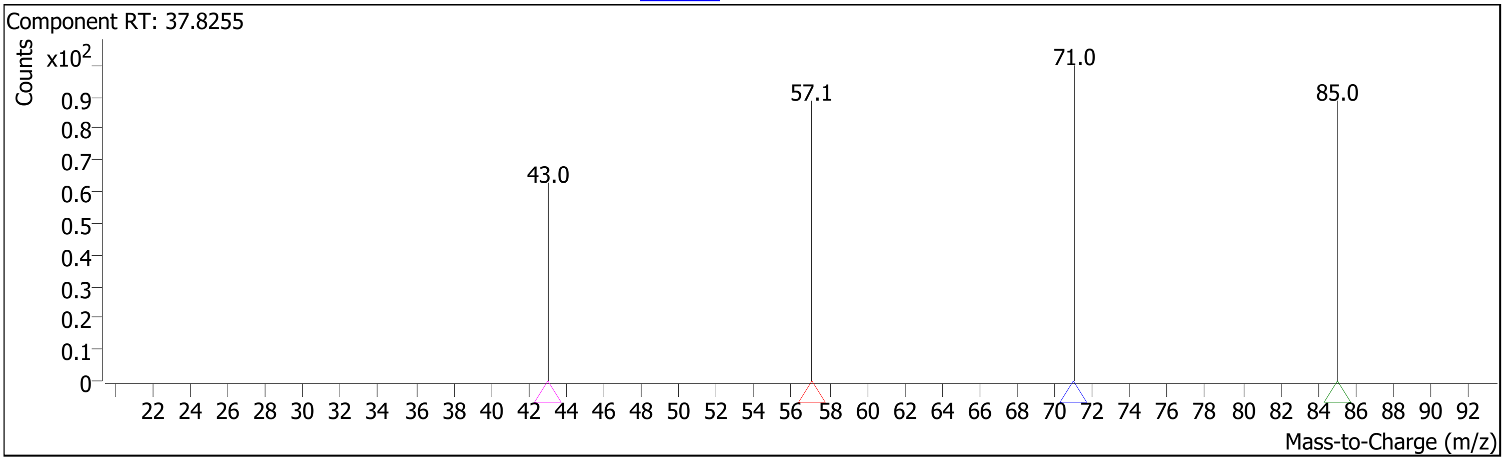

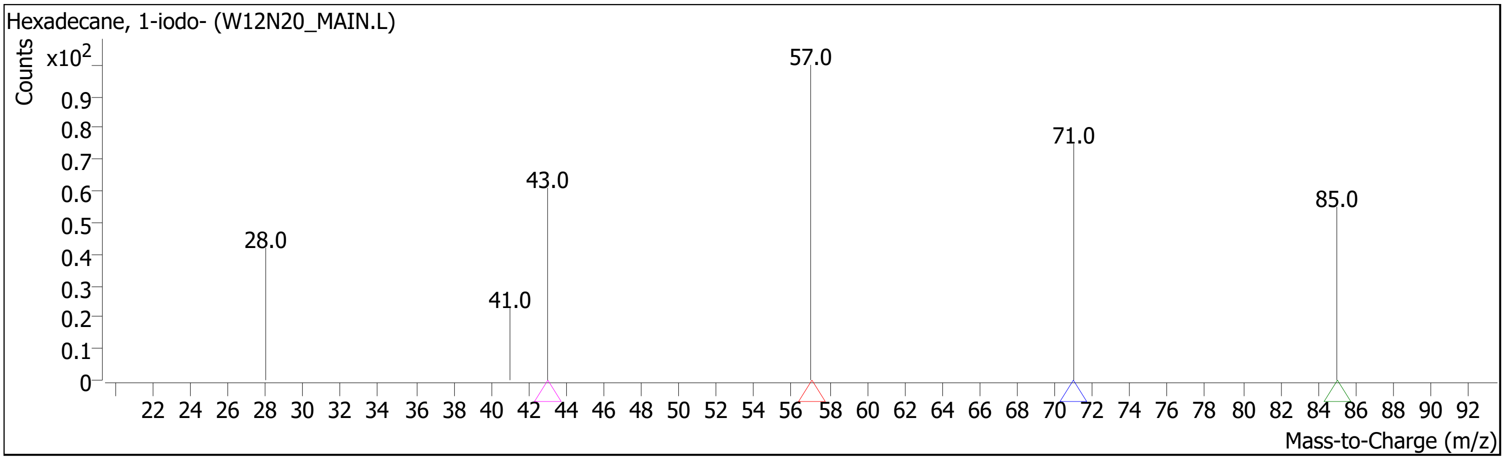

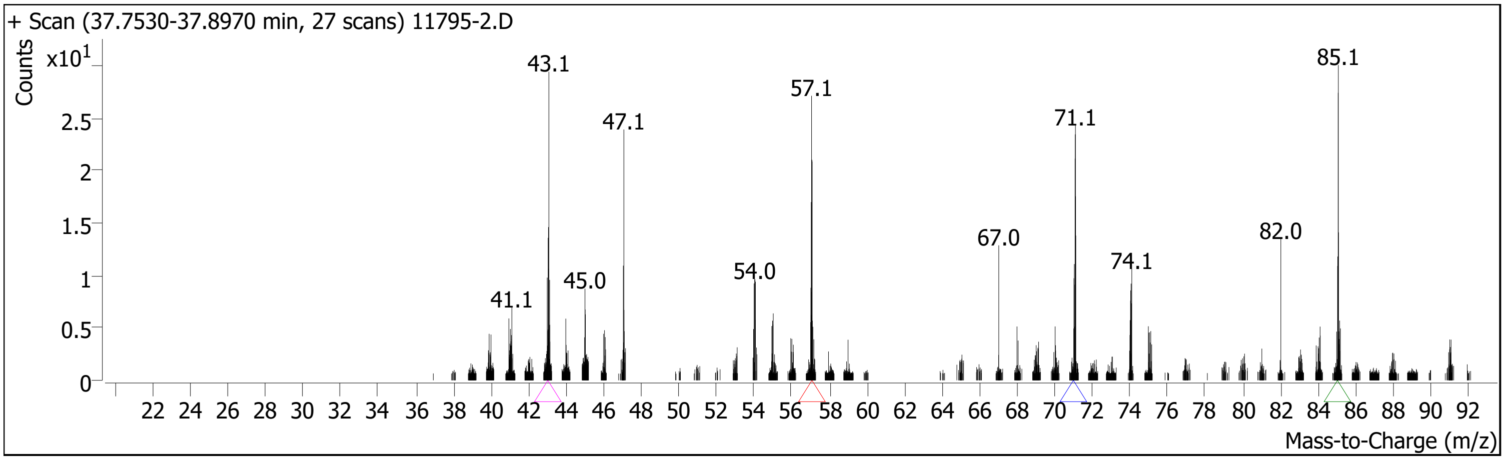

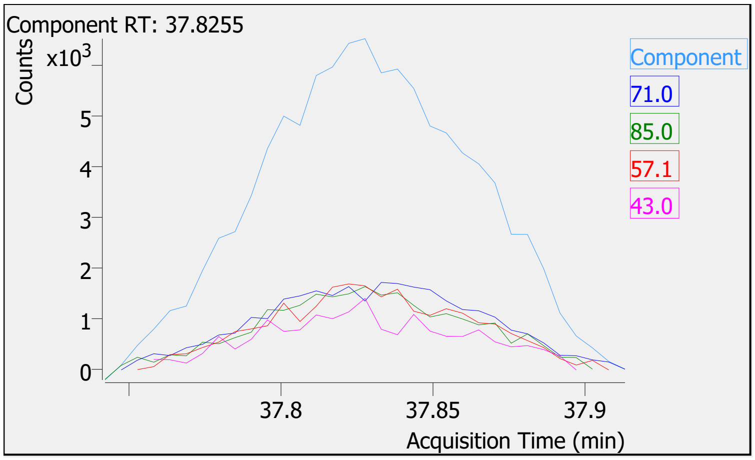

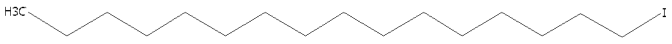

Unknown Analysis Report - Best Hits

RT	Compound Name	CAS#	Formula	Area	MI	Match Score	Sample	Sample
38.0086	Hexadecane, 1-iodo-	544-77-4	C16H33I	22539		91.4	0.17	0.69

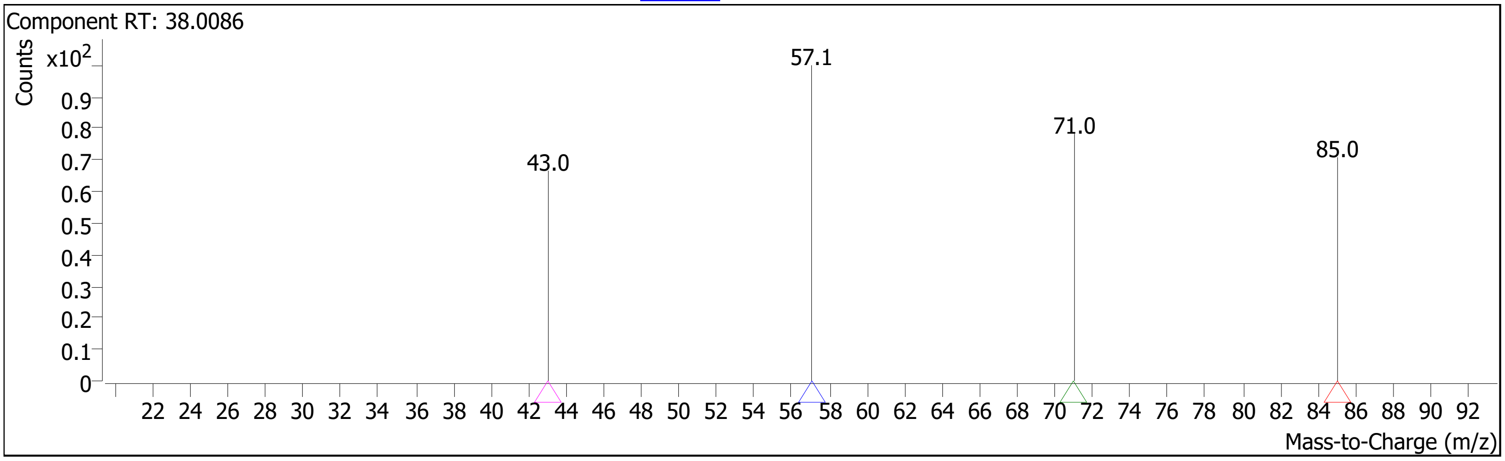

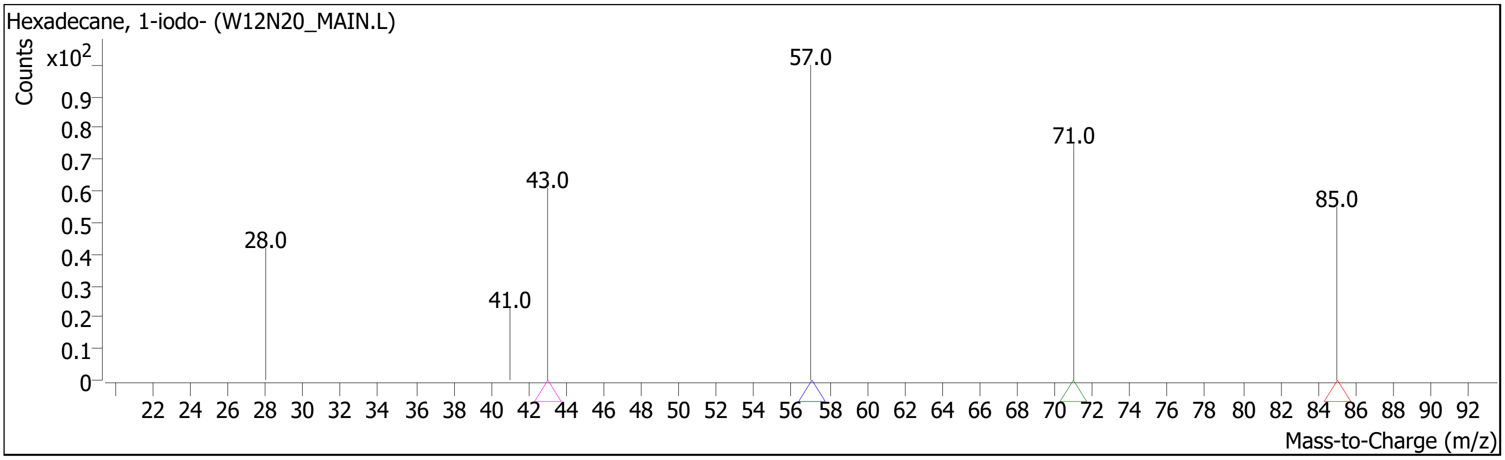

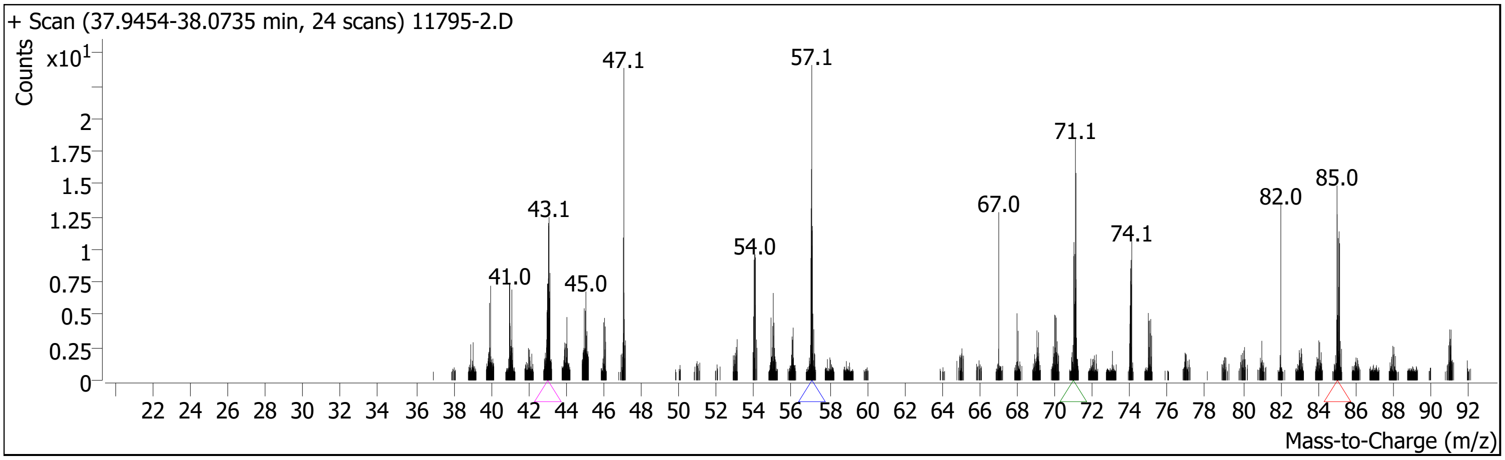

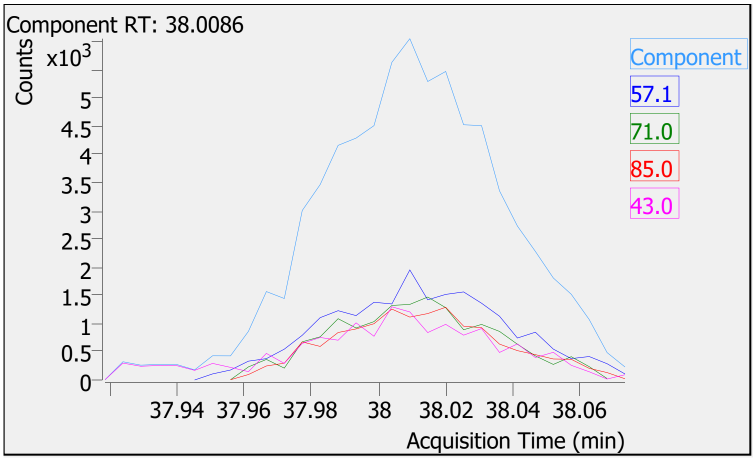

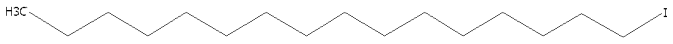

RT	Compound Name	CAS#	Formula	Area	MI	Match Score	Sample	Sample
40.1598	4,4-Dimethylpentan-1-ol	990002-63-9	C7H16O	49005		85.5	0.38	1.51

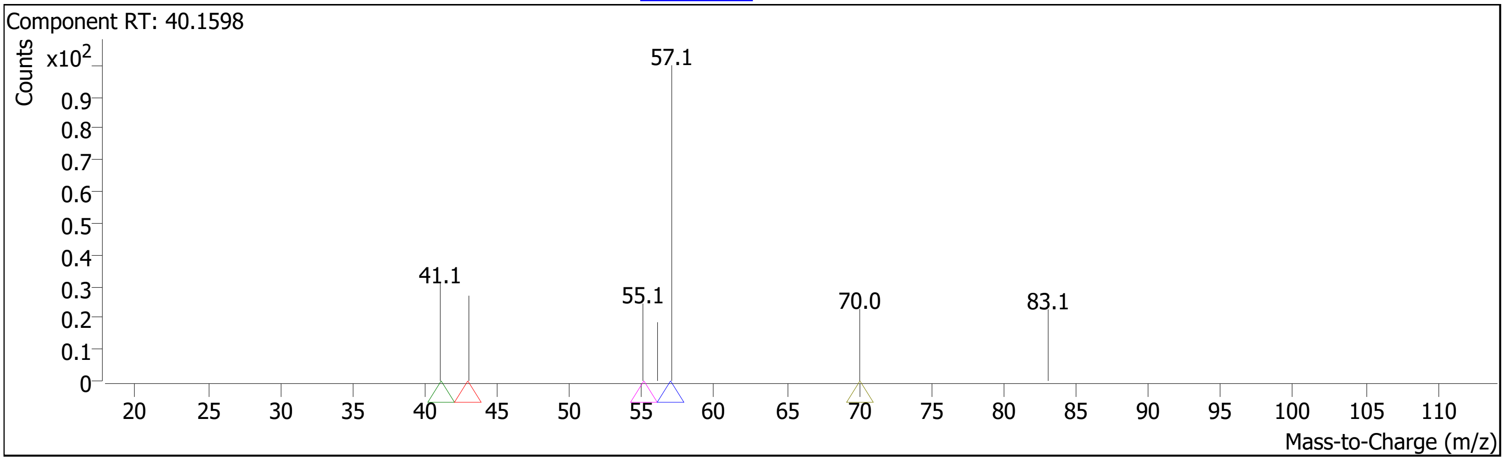

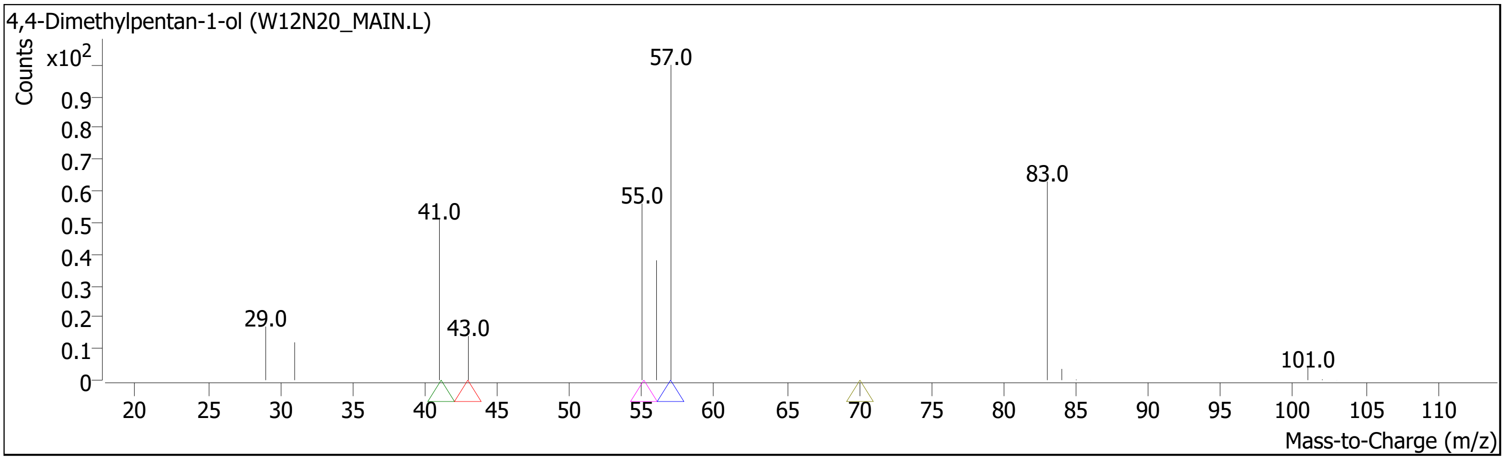

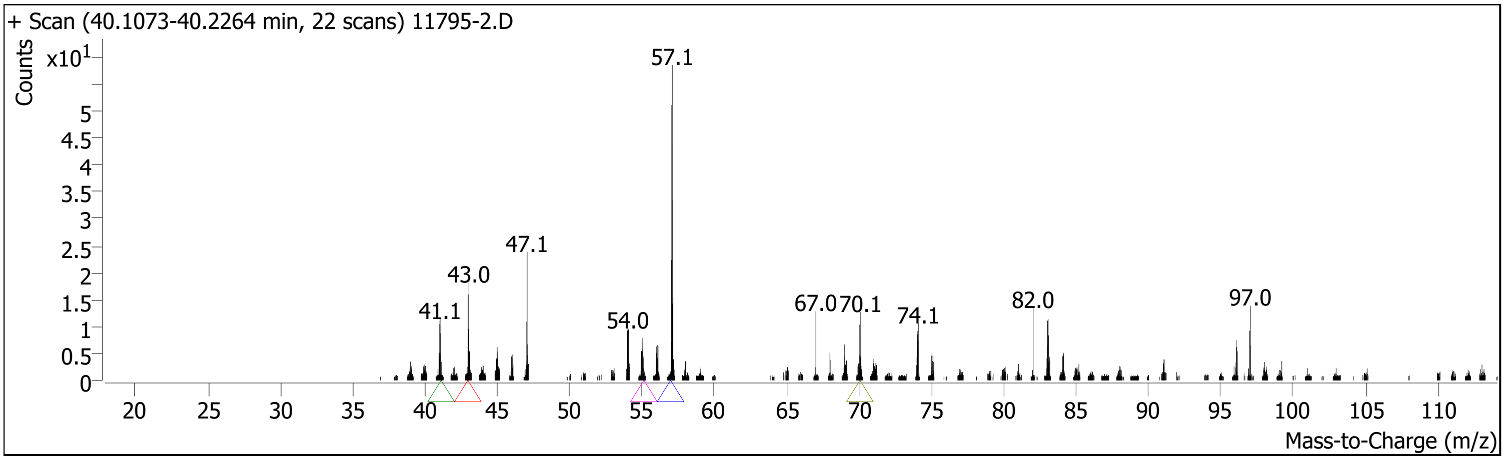

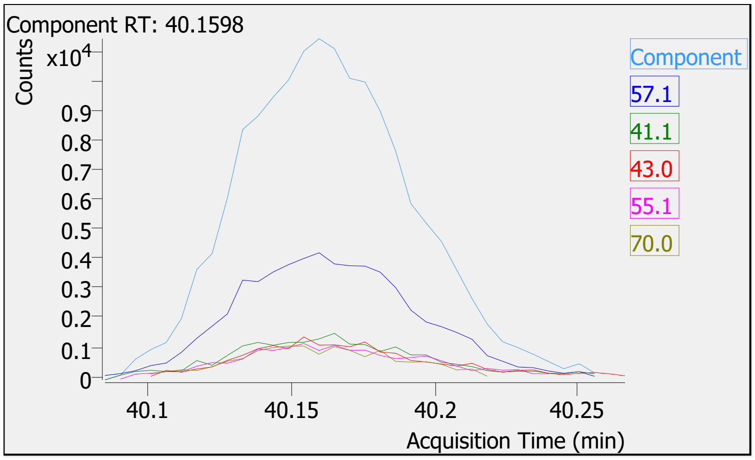

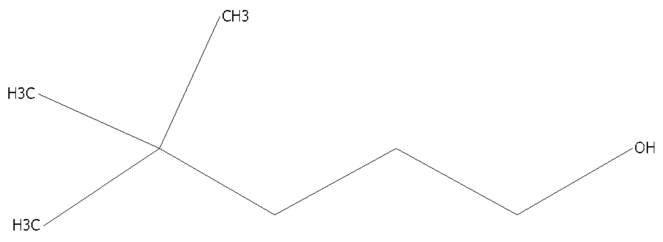

Unknown Analysis Report - Best Hits

RT	Compound Name	CAS#	Formula	Area	MI	Match Score	Sample	Sample
40.4580	2,2-Dimethylpropanoic acid 3-acetyloxypropyl ester	990075-06-2	C10H18O4	33537		94.0	0.26	1.03

Component RT: 40.4580

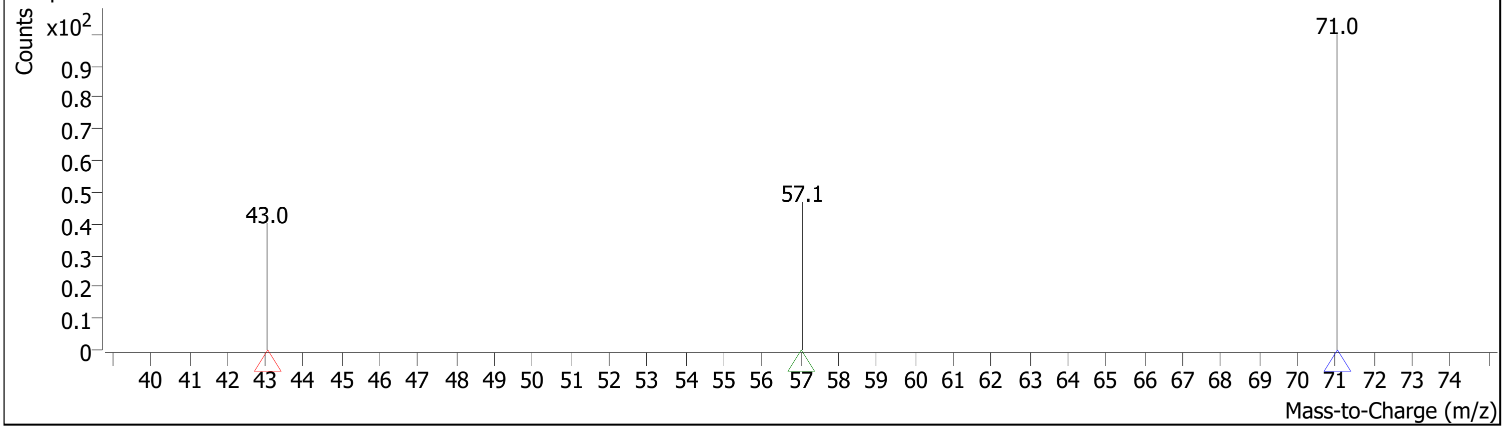

2,2-Dimethylpropanoic acid 3-acetyloxypropyl ester (W12N20_MAIN.L)

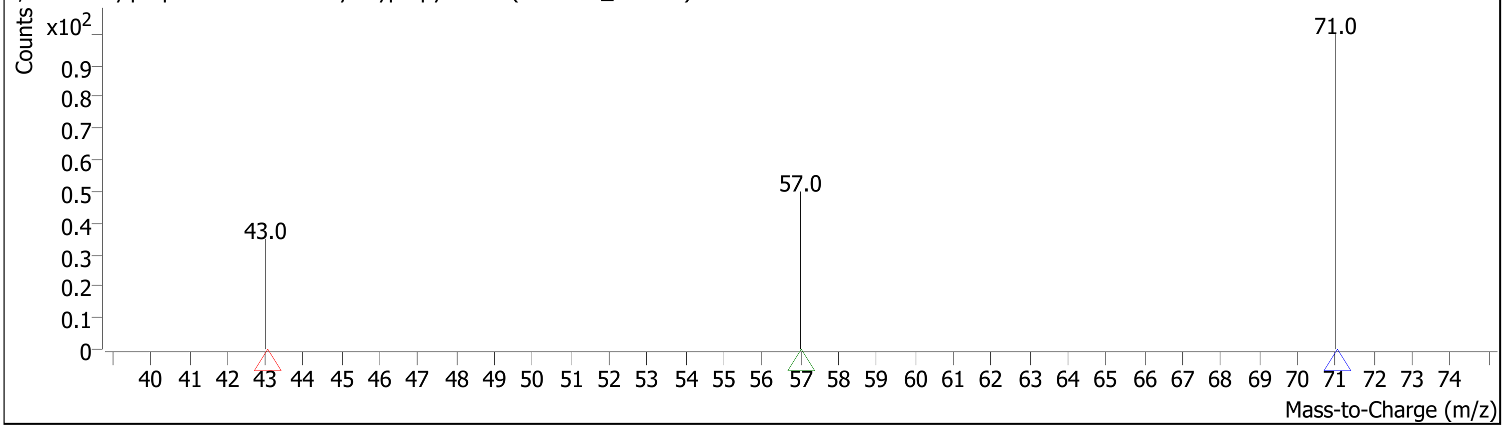

+ Scan (40.3829-40.5714 min, 36 scans) 11795-2.D

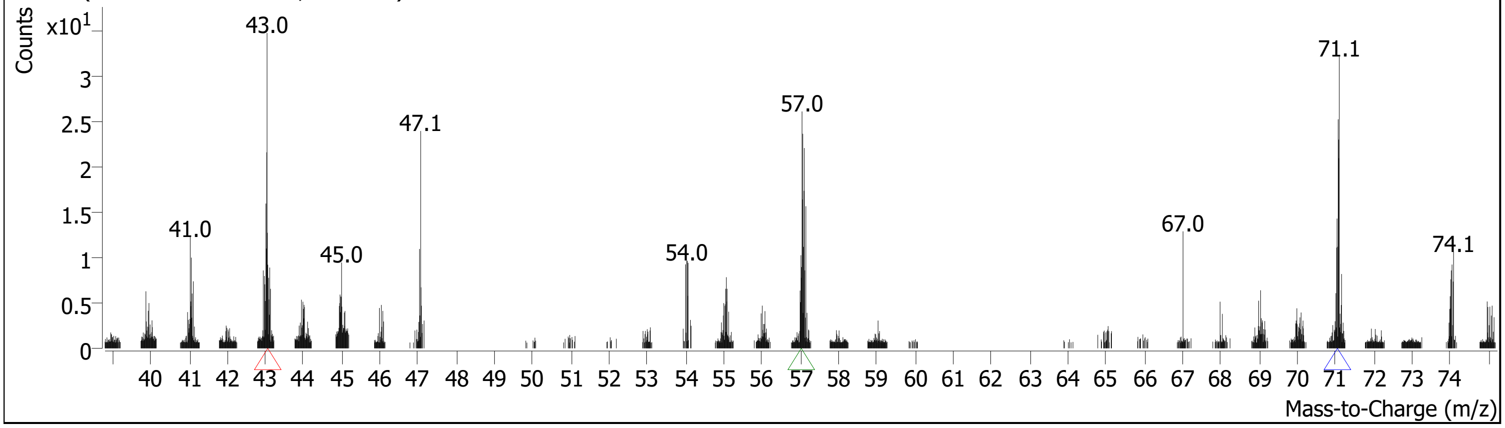

Component RT: 40.4580

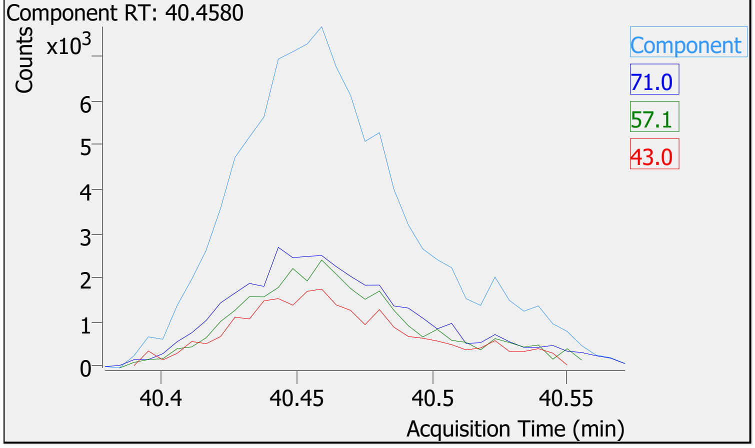

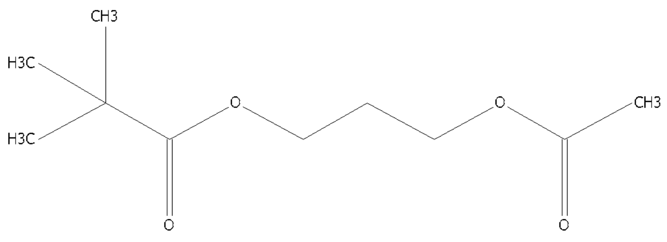

 Generated at 4:32 PM on 11/14/2023

Unknown Analysis Report - Best Hits

RT	Compound Name	CAS#	Formula	Area	MI	Match Score	Sample	Sample
41.0597	2,2-Dimethylpropanoic acid 3-acetyloxypropyl ester	990075-06-2	C10H18O4	11811		92.3	0.09	0.36

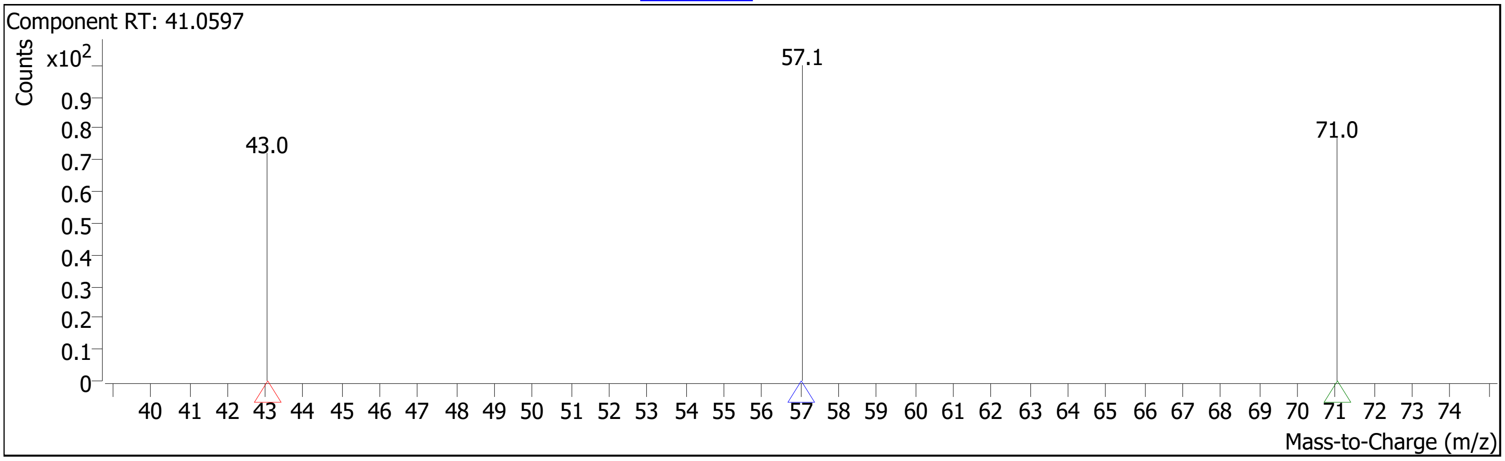

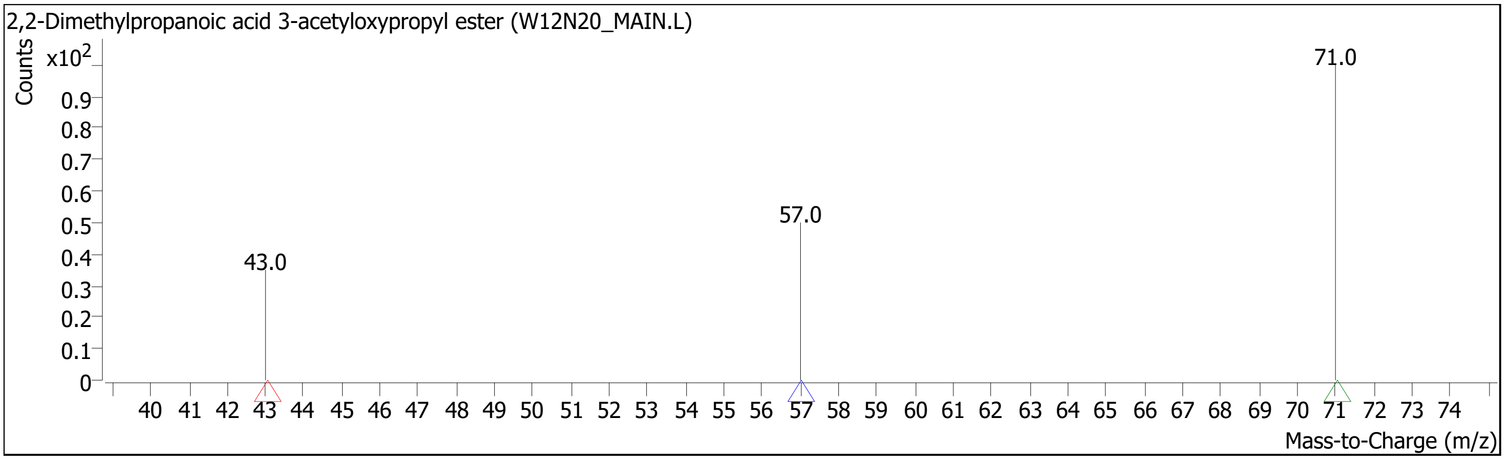

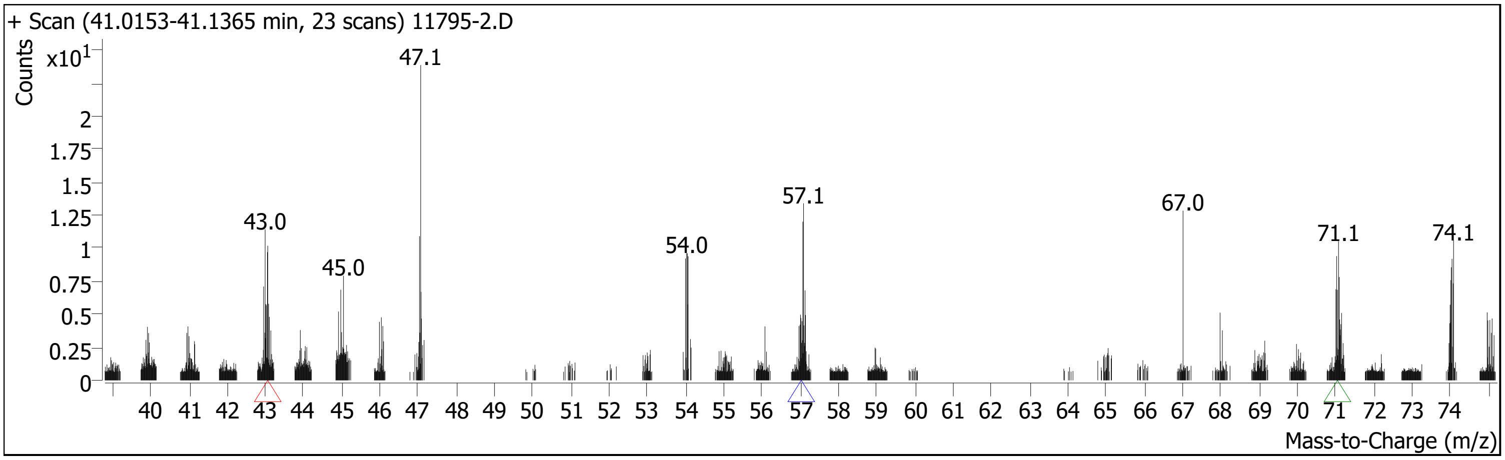

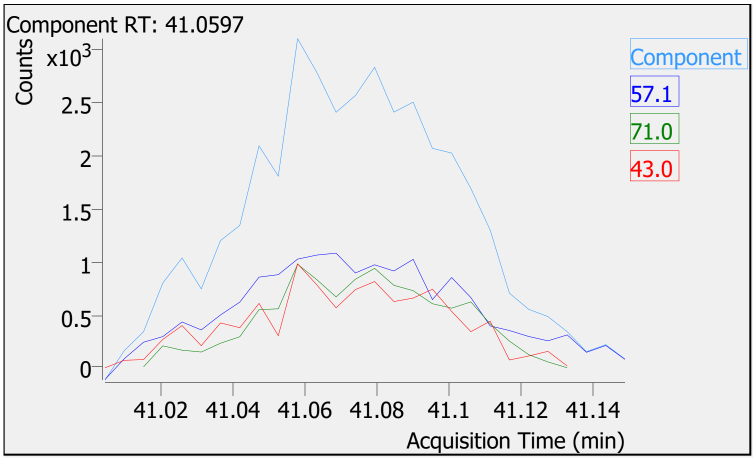

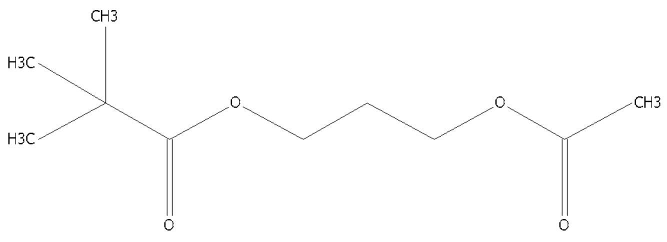

Unknown Analysis Report - Best Hits

RT	Compound Name	CAS#	Formula	Area	MI	Match Score	Sample	Sample
41.3152	4H-1-Benzopyran-4-one, 5,6,7-trimethoxy-2-(4-methoxyphenyl)-	1168-42-9	C19H18O6	30496		74.2	0.23	0.94

Component RT: 41.3152

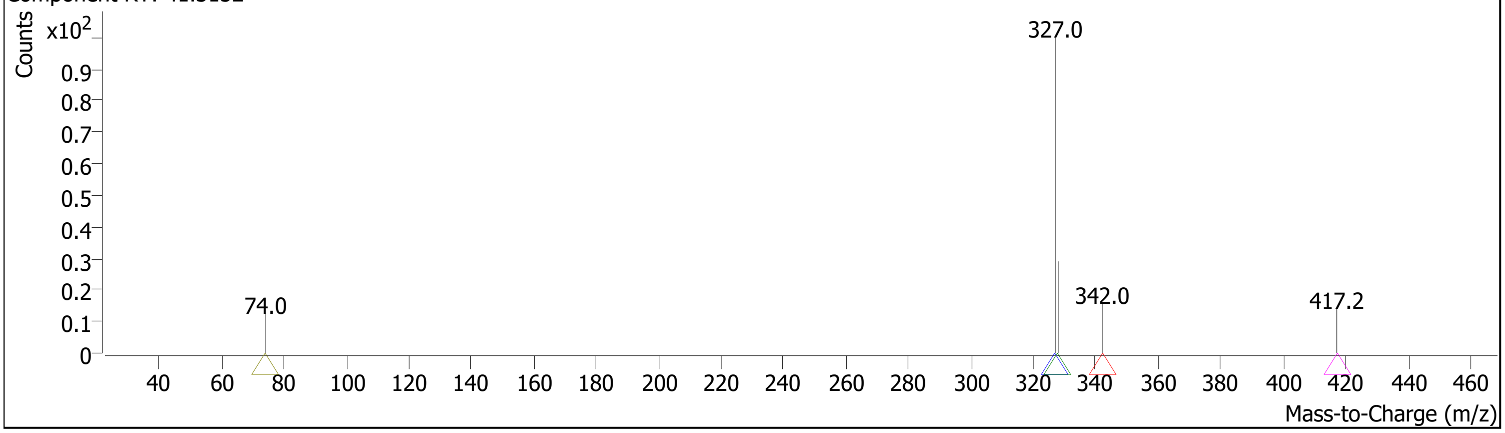

4H-1-Benzopyran-4-one, 5,6,7-trimethoxy-2-(4-methoxyphenyl)- (W12N20_MAIN.L)

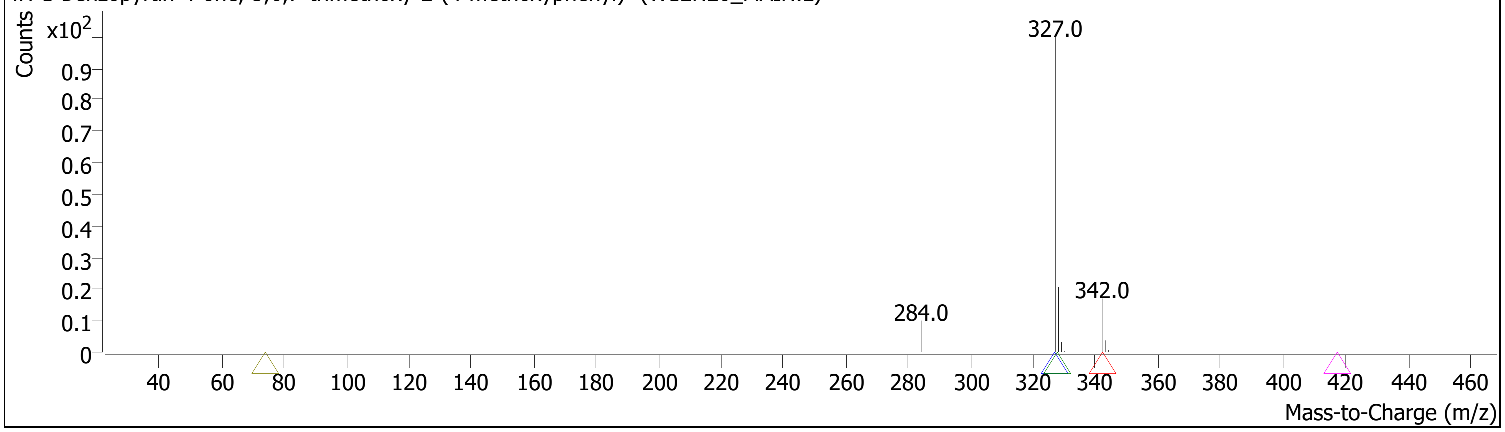

+ Scan (41.2774-41.3576 min, 16 scans) 11795-2.D

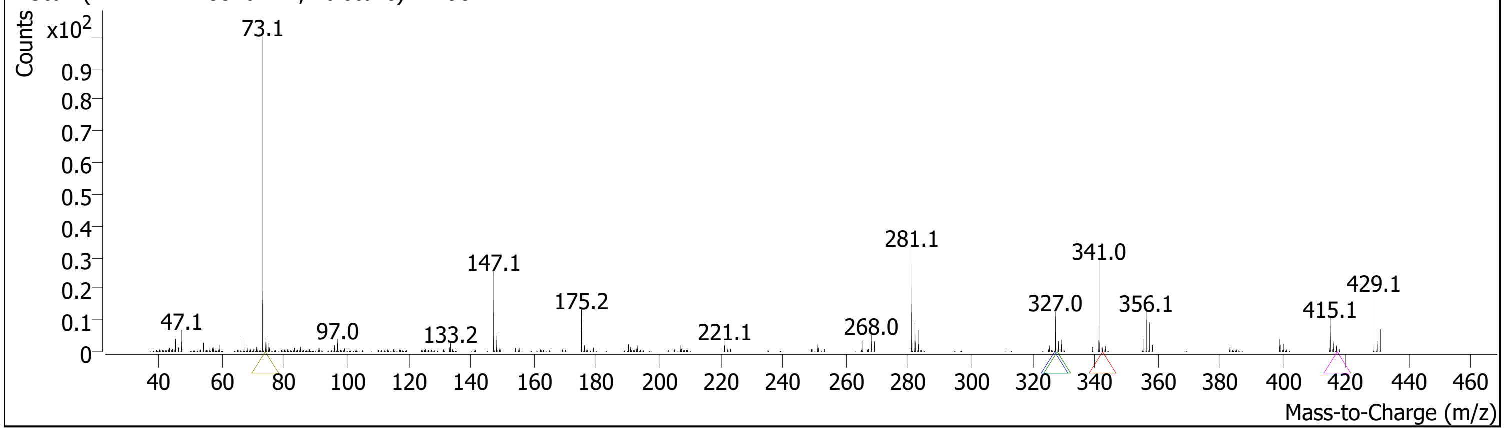

Component RT: 41.3152

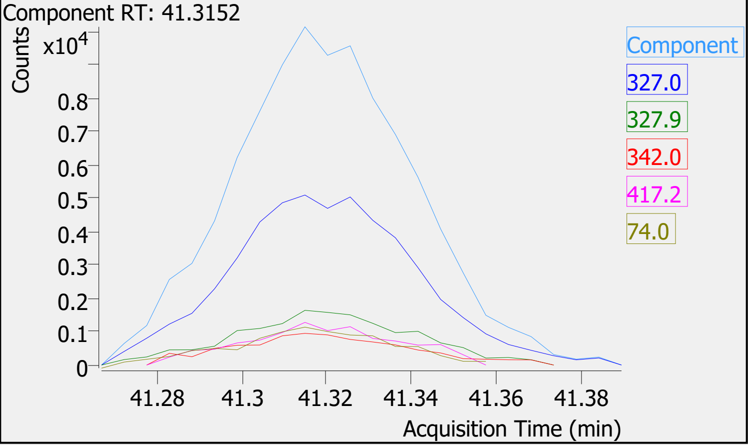

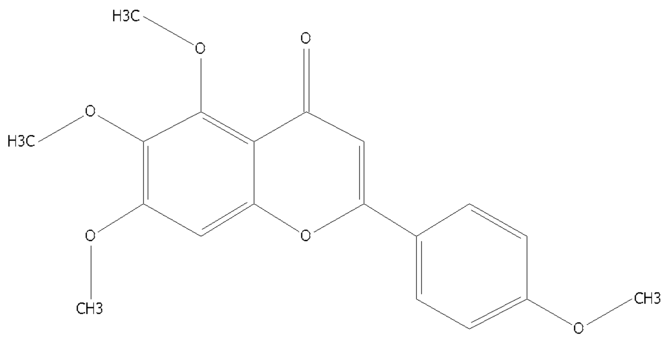

Unknown Analysis Report - Best Hits

RT	Compound Name	CAS#	Formula	Area	MI	Match Score	Sample	Sample
41.6900	.beta.,D-Xylopyranose Tetrabenzoate	990630-11-9	C33H26O9	32584		93.9	0.25	1.00

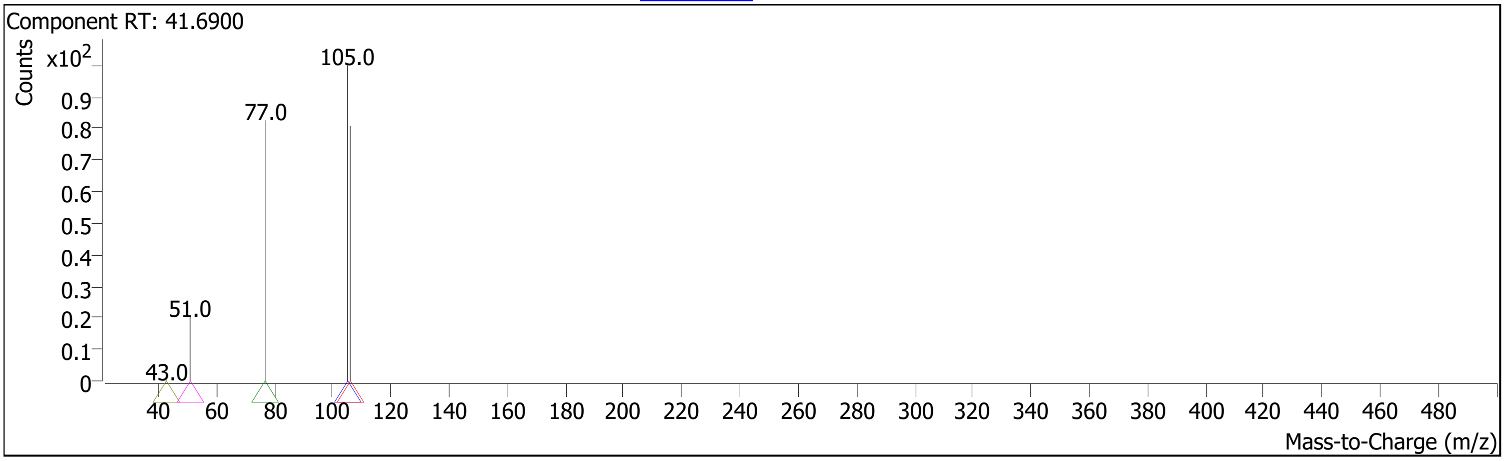

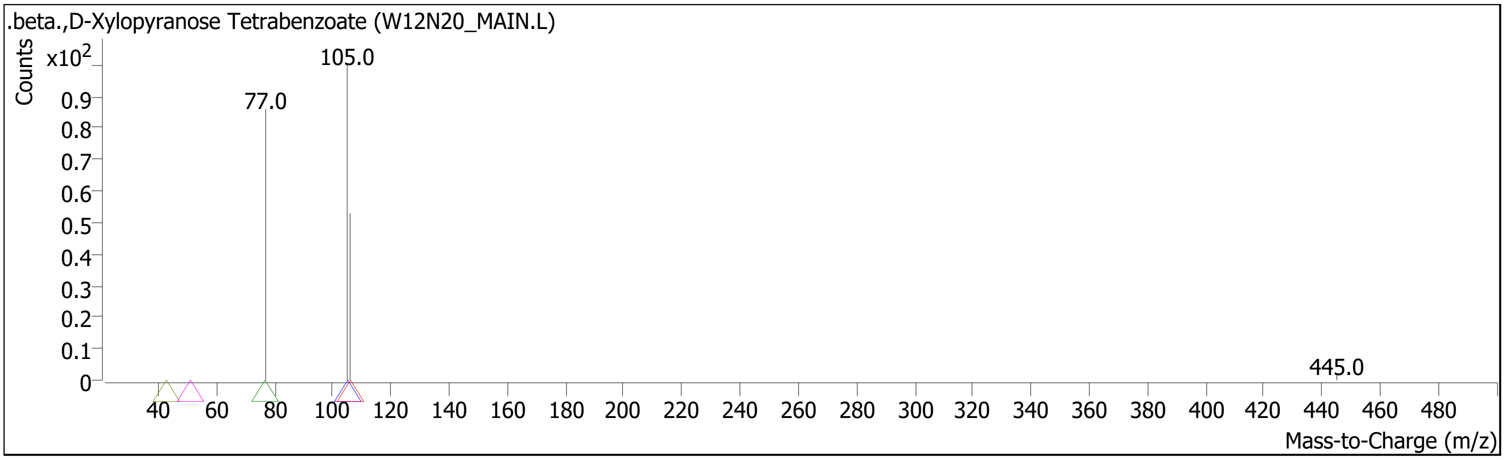

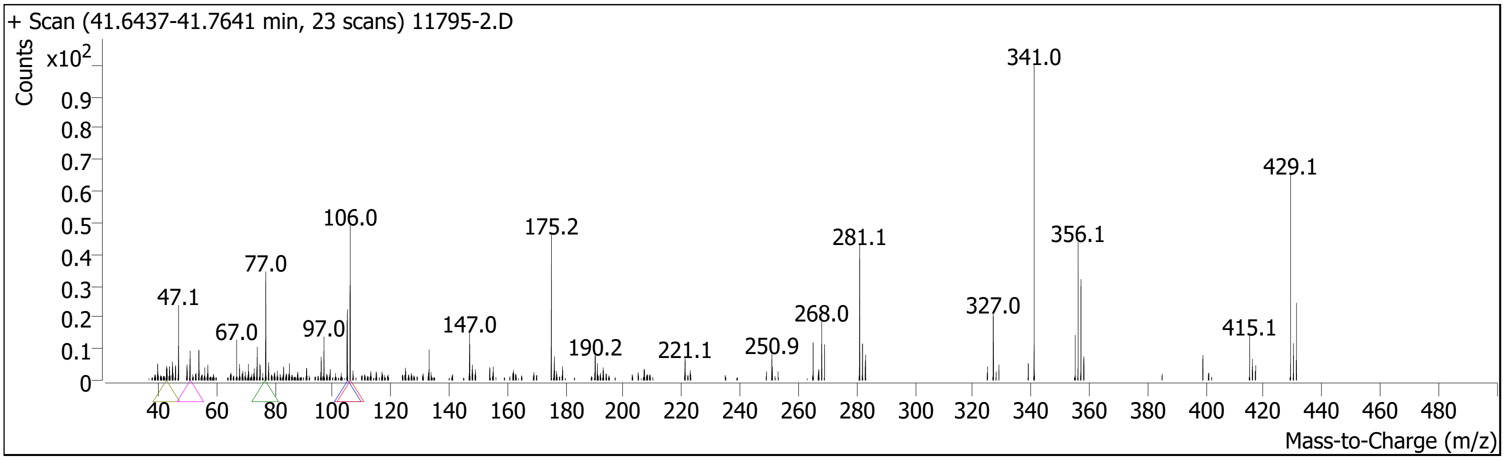

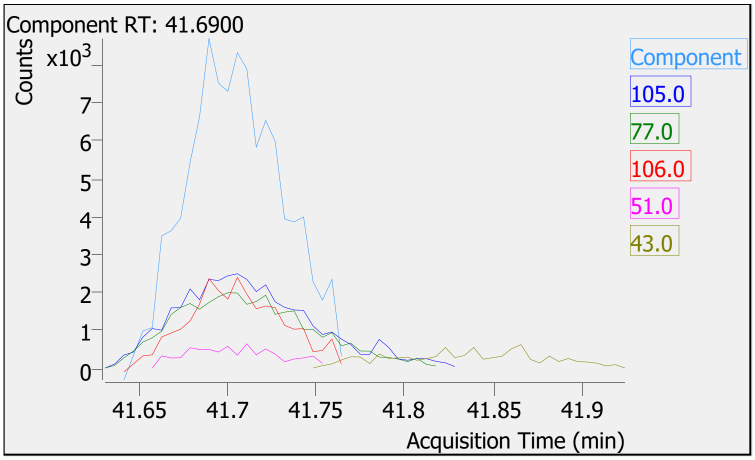

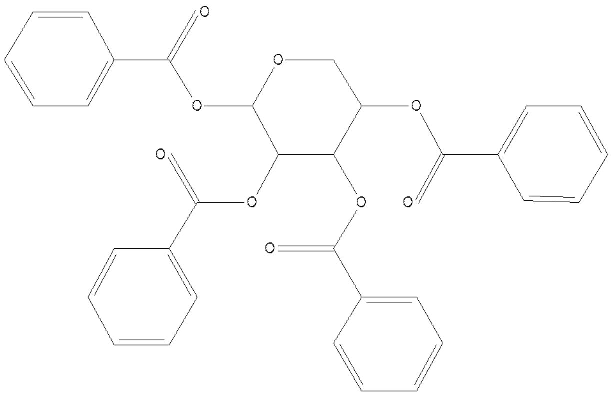

RT	Compound Name	CAS#	Formula	Area	MI	Match Score	Sample	Sample
47.9288	2,2-Dimethylpropanoic acid 3-acetyloxypropyl ester	990075-06-2	C10H18O4	10105		89.6	0.08	0.31

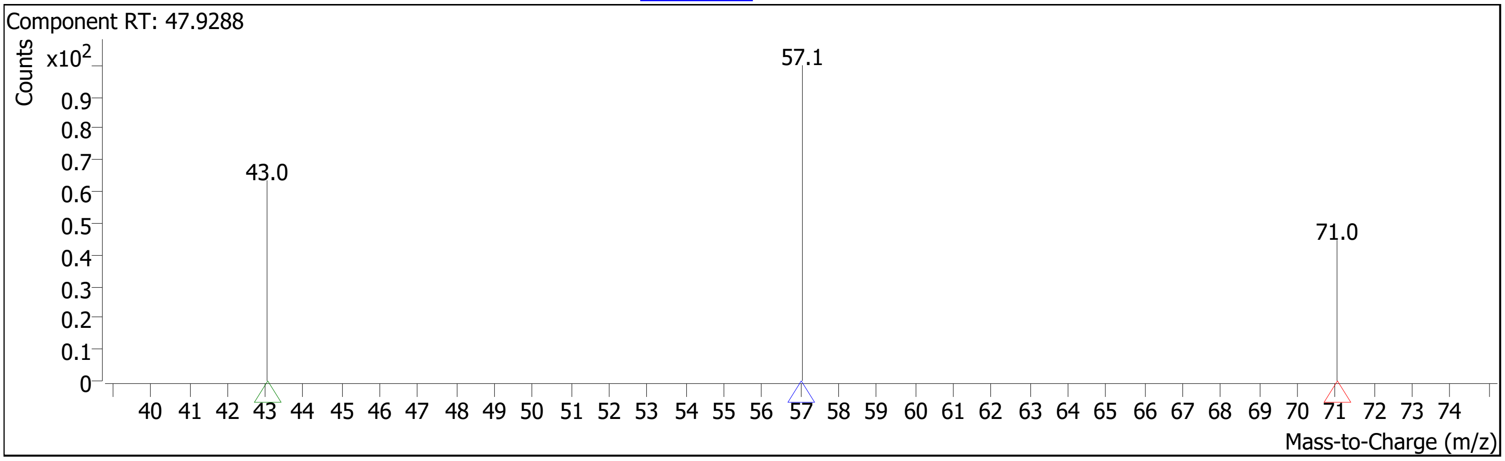

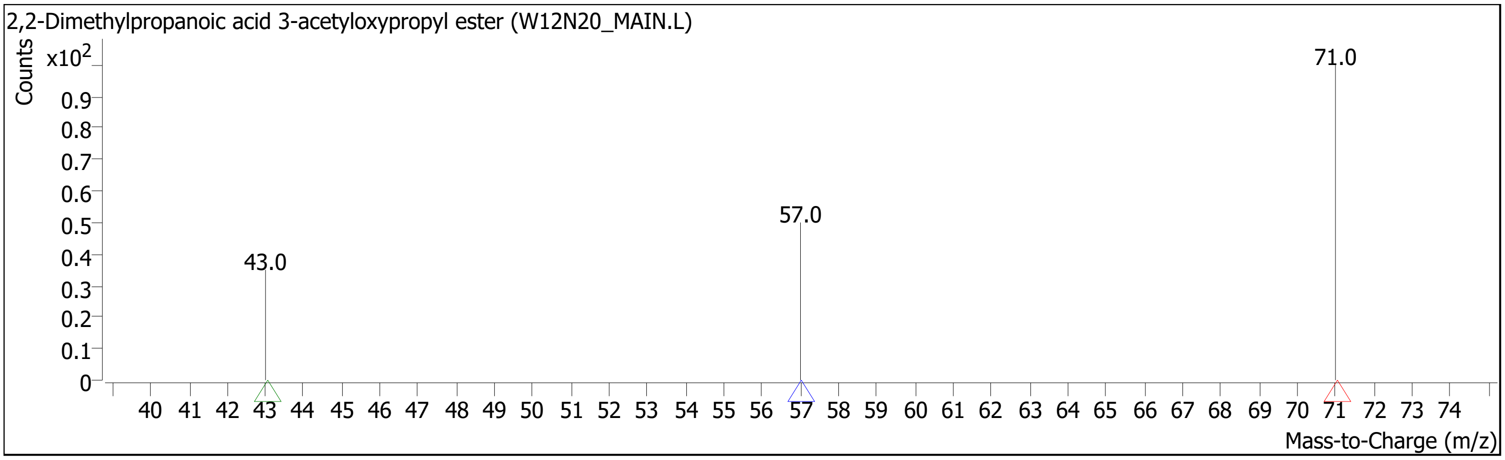

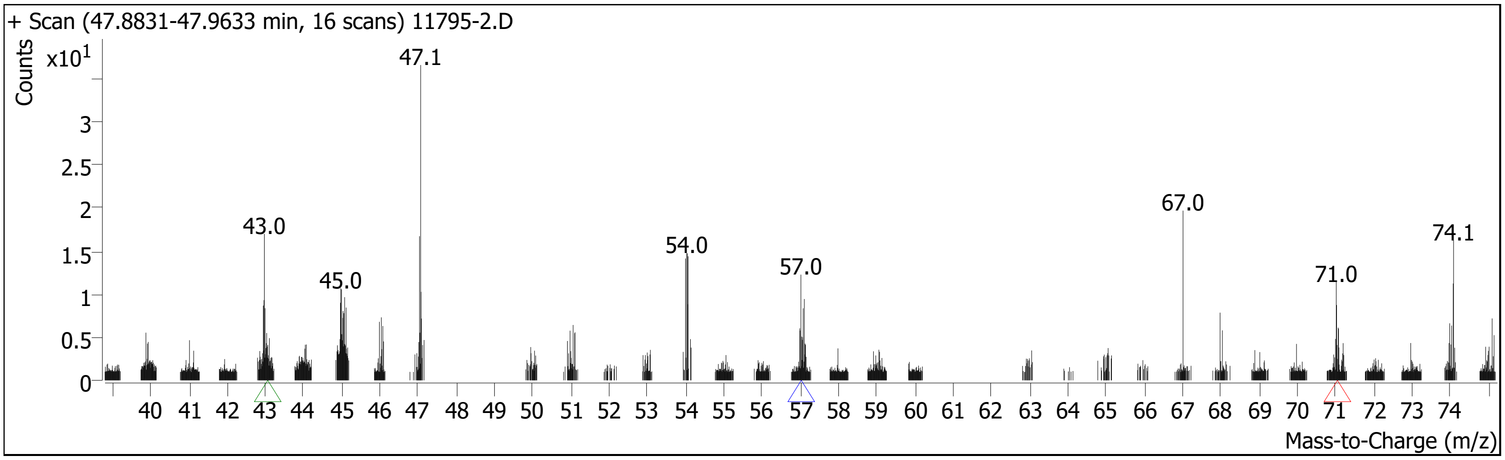

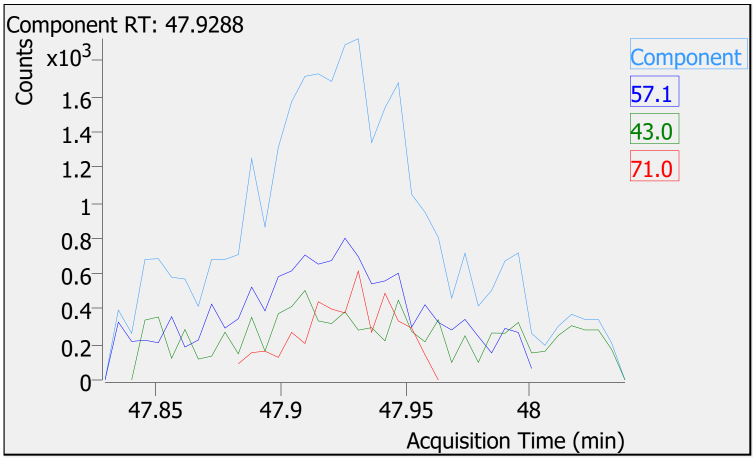

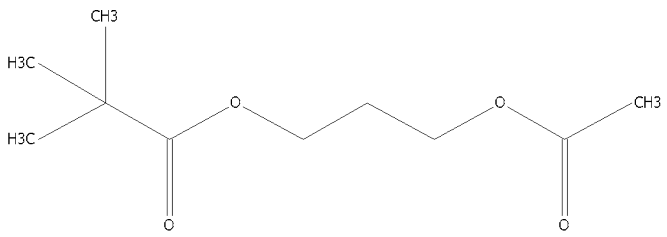

RT	Compound Name	CAS#	Formula	Area	MI	Match Score	Sample	Sample
48.9912	Hexadecane, 1-iodo-	544-77-4	C16H33I	19014		91.3	0.15	0.59

Component RT: 48.9912

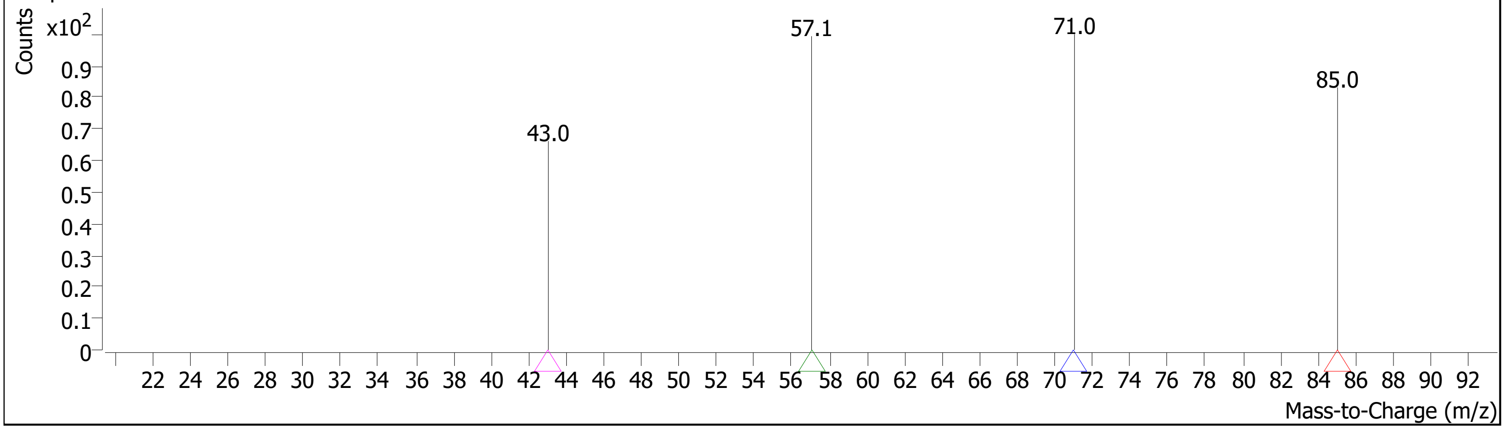

Hexadecane, 1-iodo- (W12N20_MAIN.L)

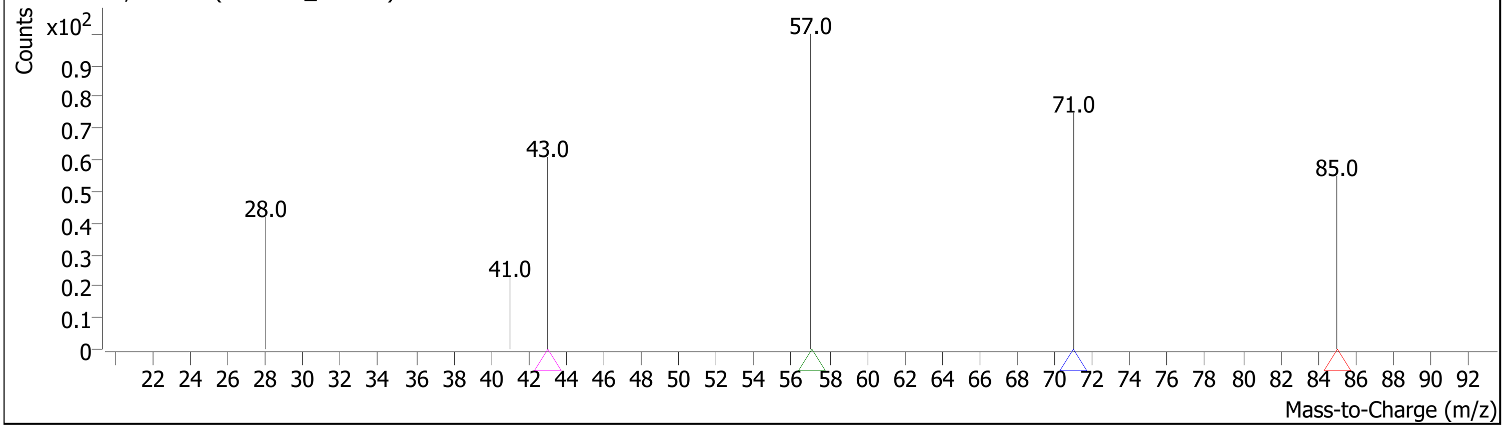

+ Scan (48.9274-49.0865 min, 30 scans) 11795-2.D

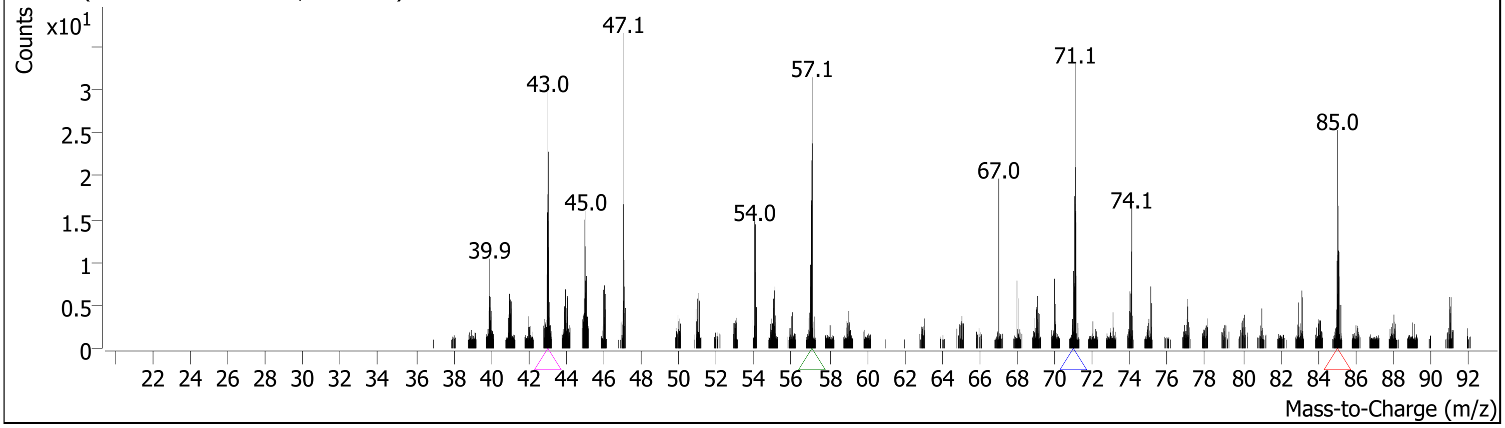

Component RT: 48.9912

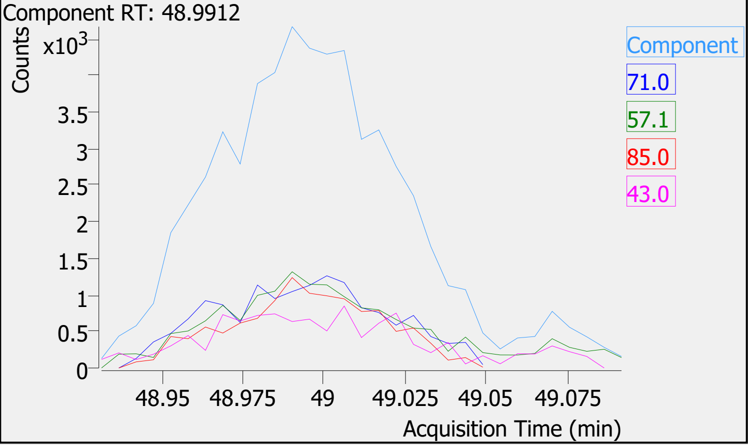

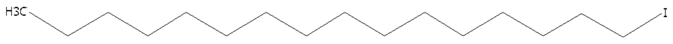

RT	Compound Name	CAS#	Formula	Area	MI	Match Score	Sample	Sample
50.2964	1,1-Cyclopropanedicarbonitrile, 2-methyl-2-pentyl-	16738-90-2	C11H16N2	10479		79.3	0.08	0.32

Component RT: 50.2964

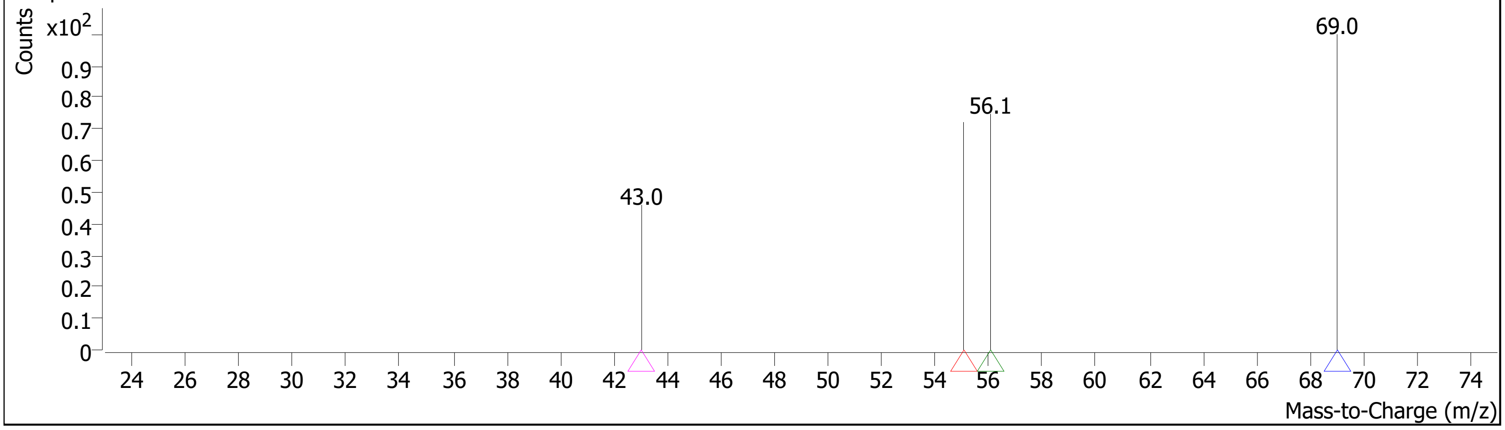

1,1-Cyclopropanedicarbonitrile, 2-methyl-2-pentyl- (W12N20_MAIN.L)

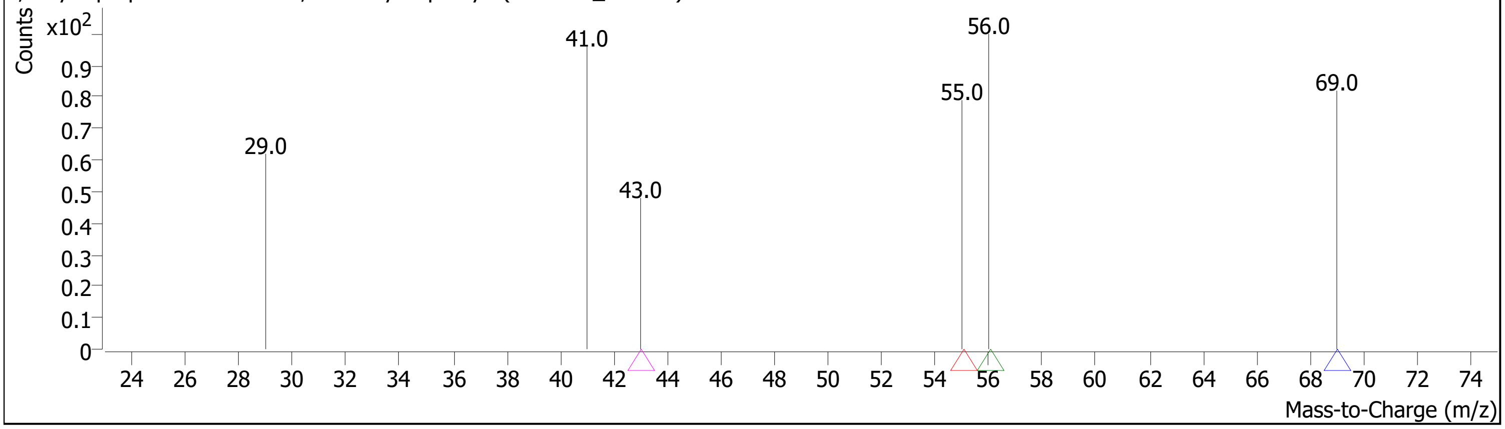

+ Scan (50.2472-50.3616 min, 22 scans) 11795-2.D

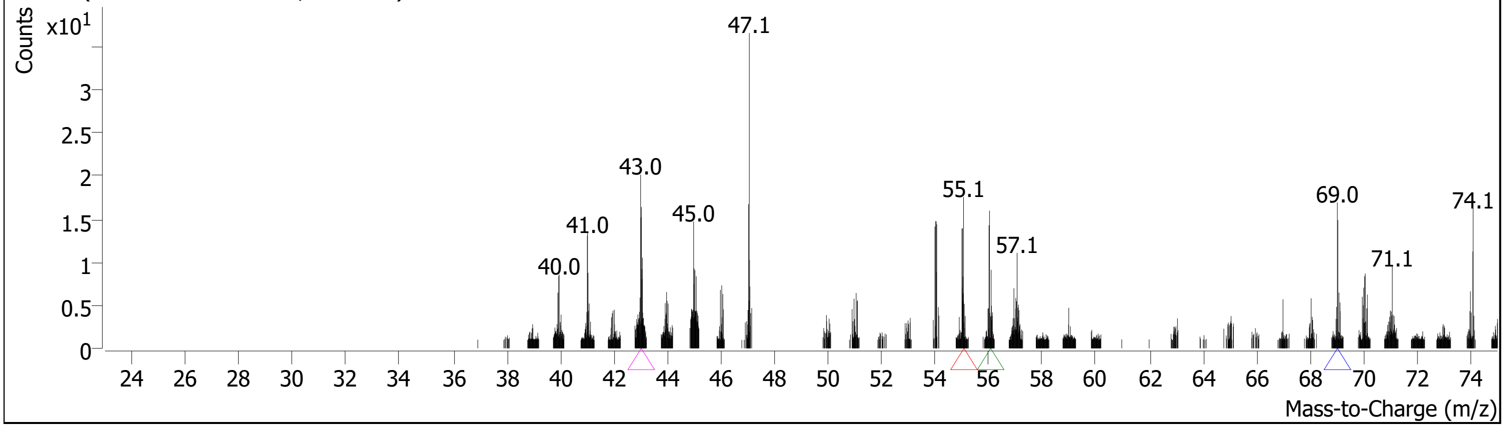

Component RT: 50.2964

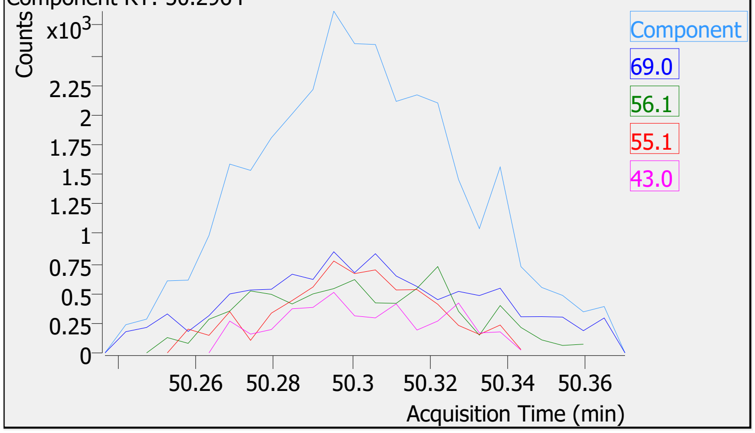

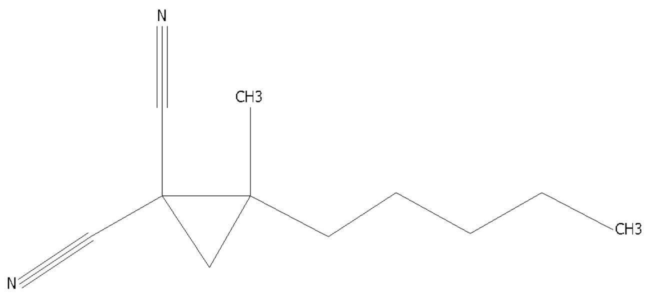

Unknown Analysis Report - Best Hits

RT	Compound Name	CAS#	Formula	Area	MI	Match Score	Sample	Sample
50.5562	3,5-bis[14C]-Trachelantamidine	990009-77-6	C8H15NO	49660		84.2	0.38	1.53

Component RT: 50.5562

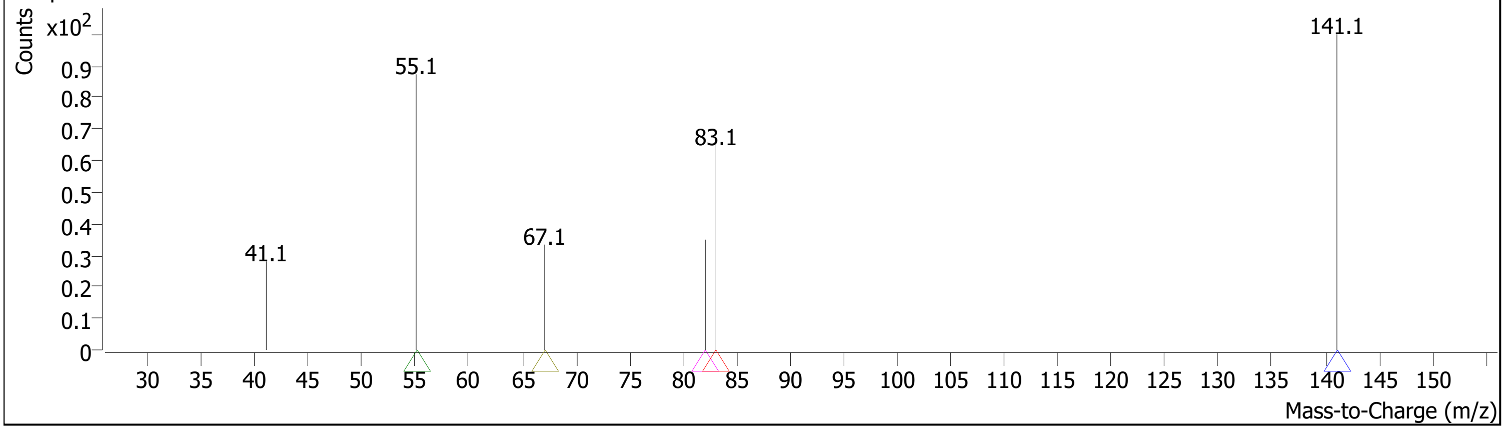

3,5-bis[14C]-Trachelantamidine (W12N20_MAIN.L)

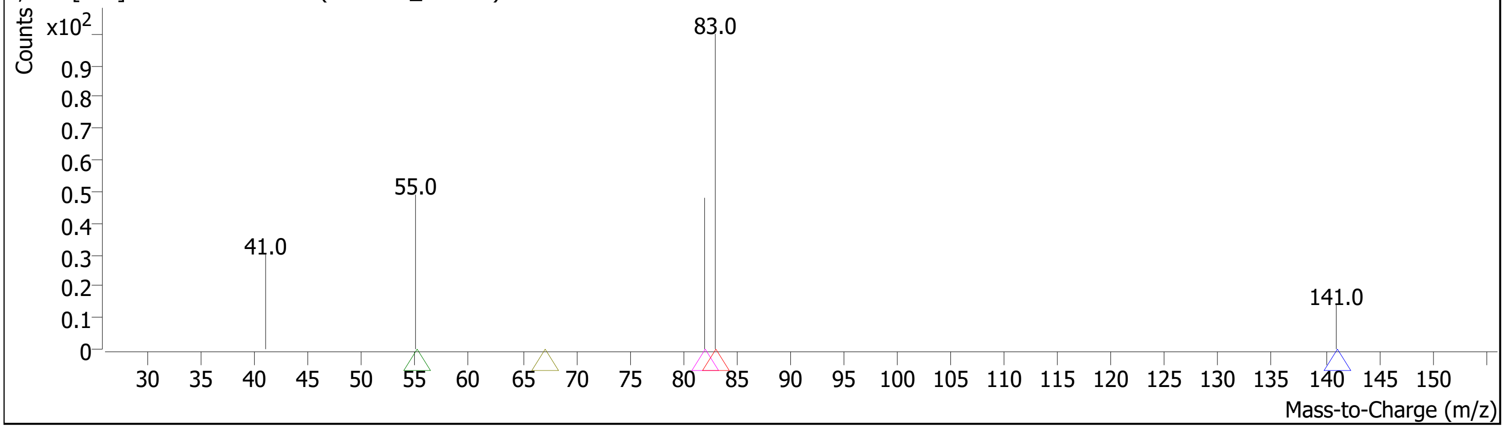

+ Scan (50.4879-50.6323 min, 28 scans) 11795-2.D

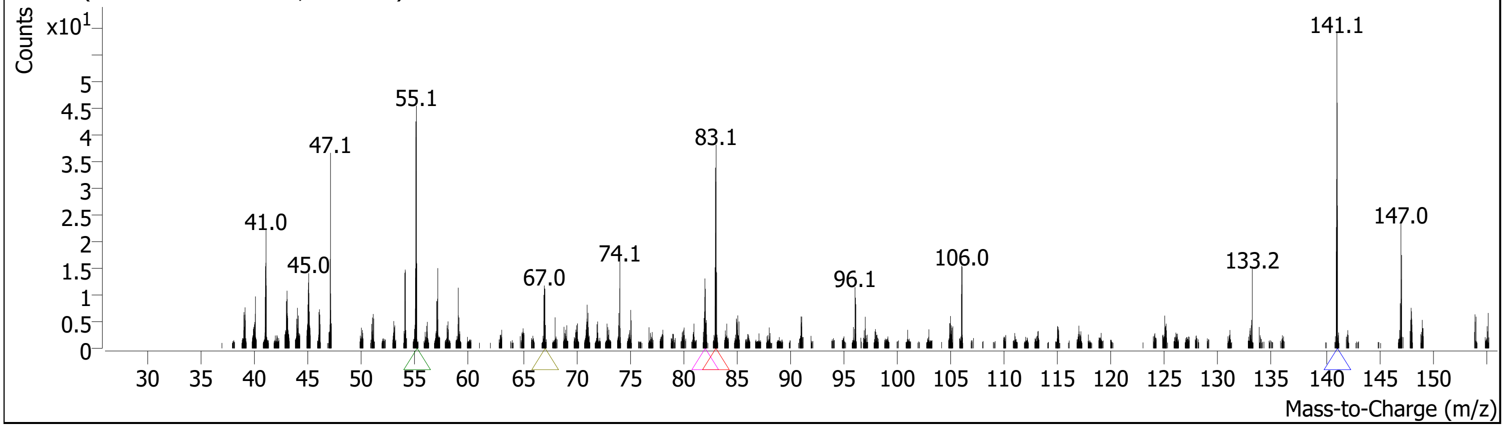

Component RT: 50.5562

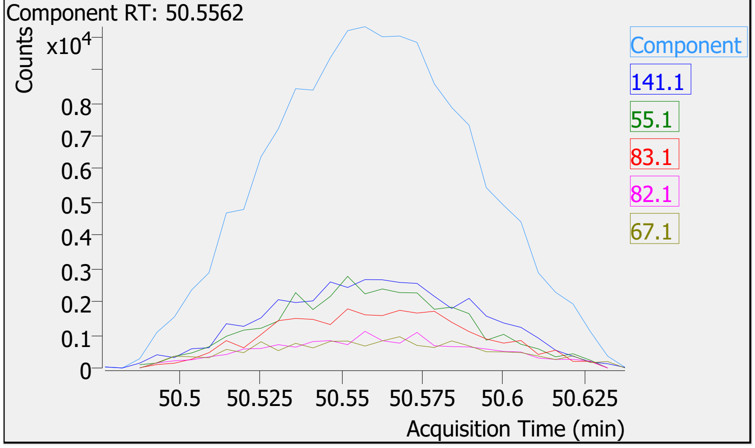

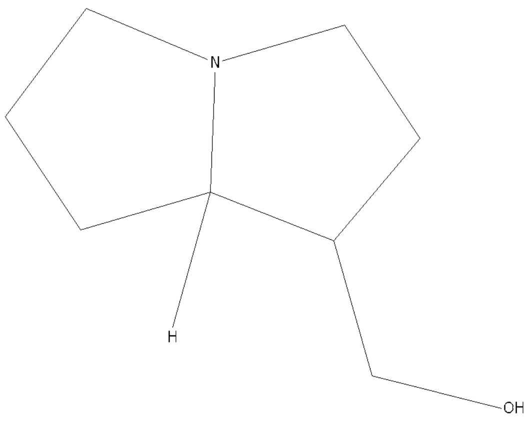

Unknown Analysis Report - Best Hits

RT	Compound Name	CAS#	Formula	Area	MI	Match Score	Sample	Sample
51.1328	3-Methoxy-2'-phenyl-16.beta.,17.beta.-dihydro-4'H-[1,3]oxazino[5',6' : 16,17]estra-1,3,5(10)triene	990516-57-1	C27H31NO2	31498		71.0	0.24	0.97

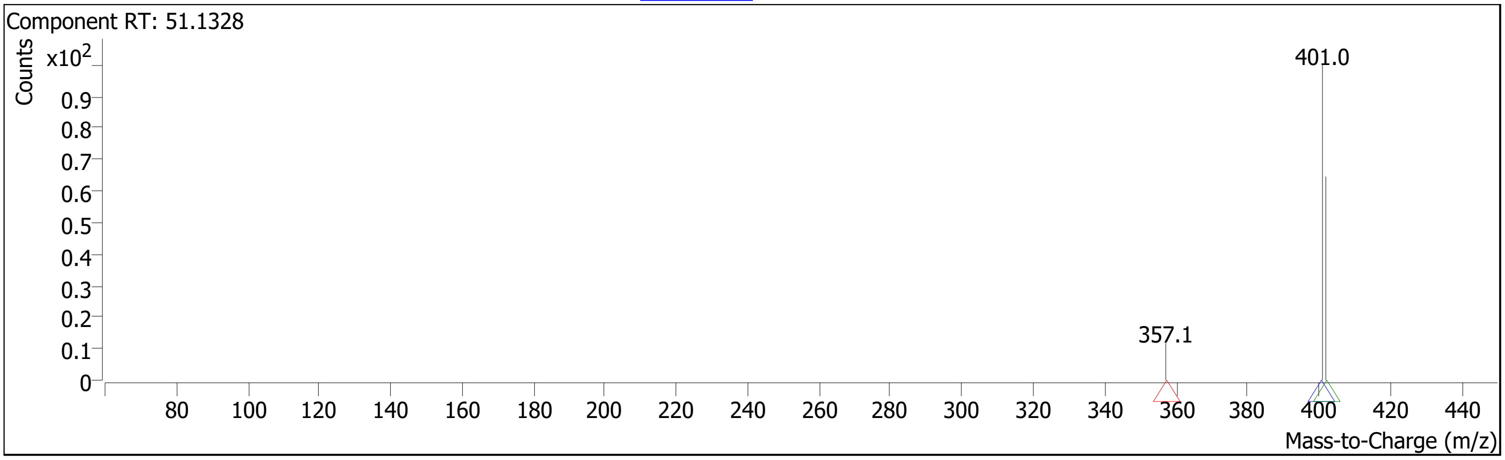

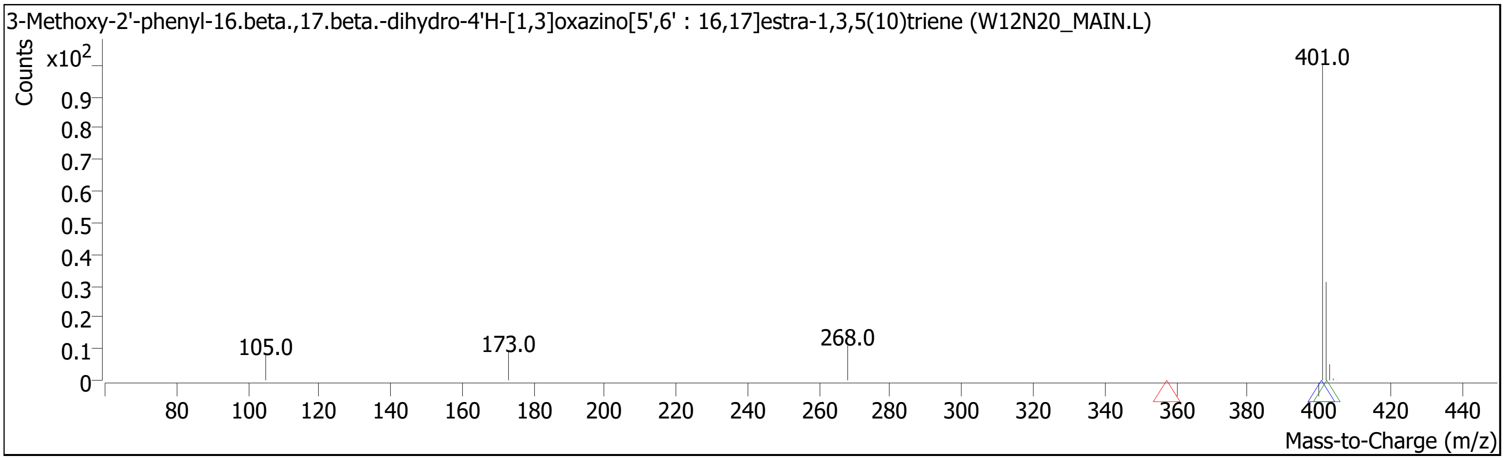

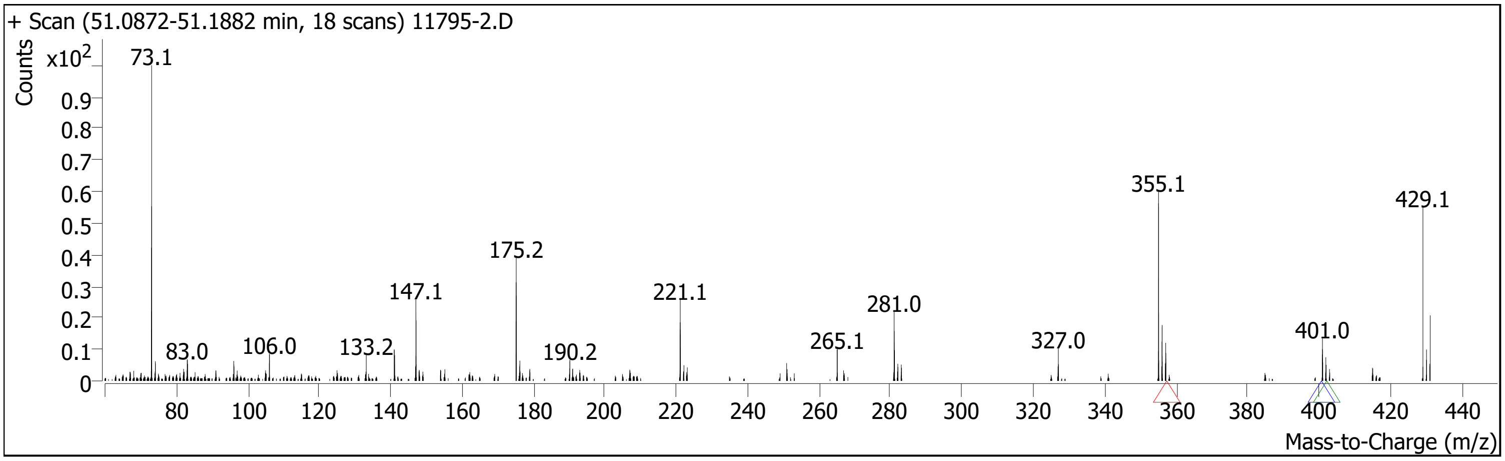

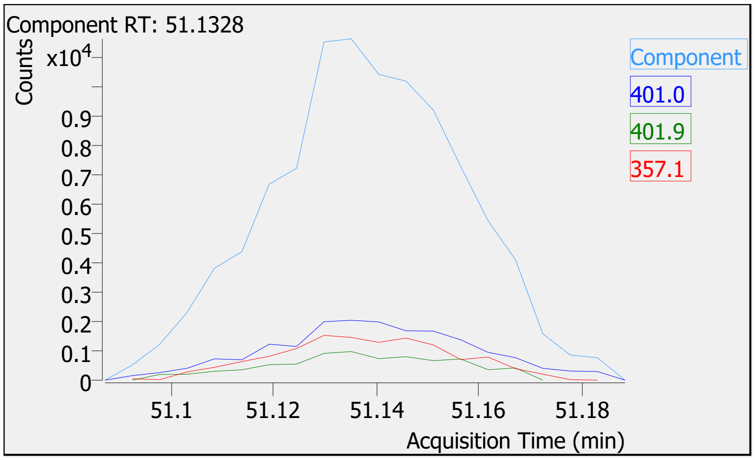

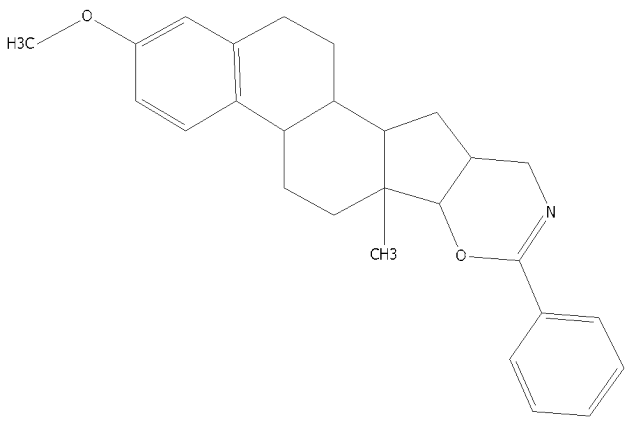

Unknown Analysis Report - Best Hits

RT	Compound Name	CAS#	Formula	Area	MI	Match Score	Sample	Sample
51.1390	2,3-Diphenyl-5,8,9,10-tetrahydropyrimido[1,2-c]pteridin-6-one	990434-90-9	C21H17N5O	34090		82.2	0.26	1.05

Component RT: 51.1390

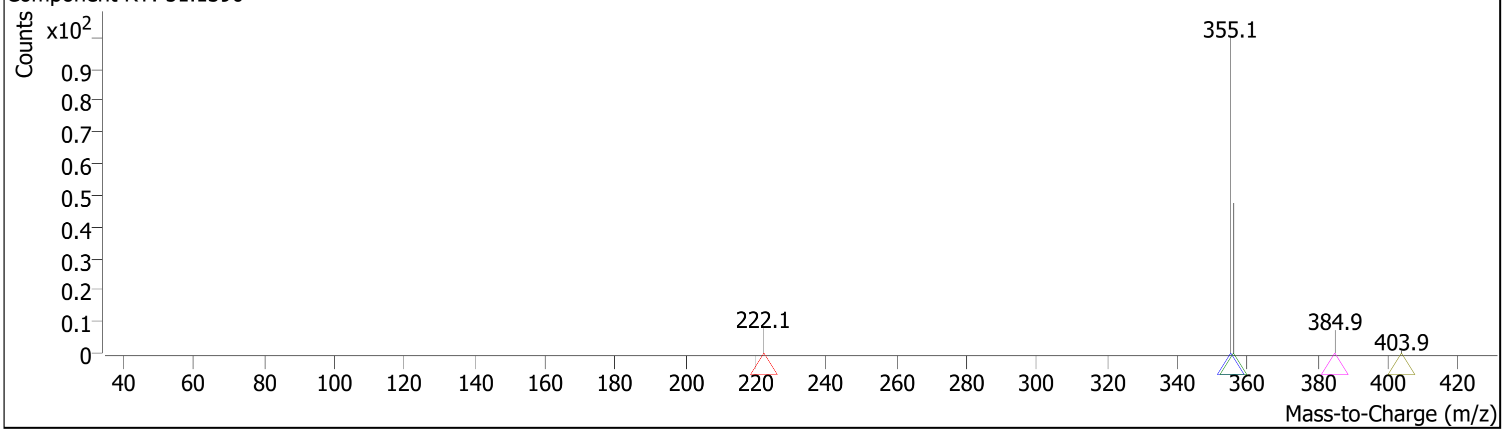

2,3-Diphenyl-5,8,9,10-tetrahydropyrimido[1,2-c]pteridin-6-one (W12N20_MAIN.L)

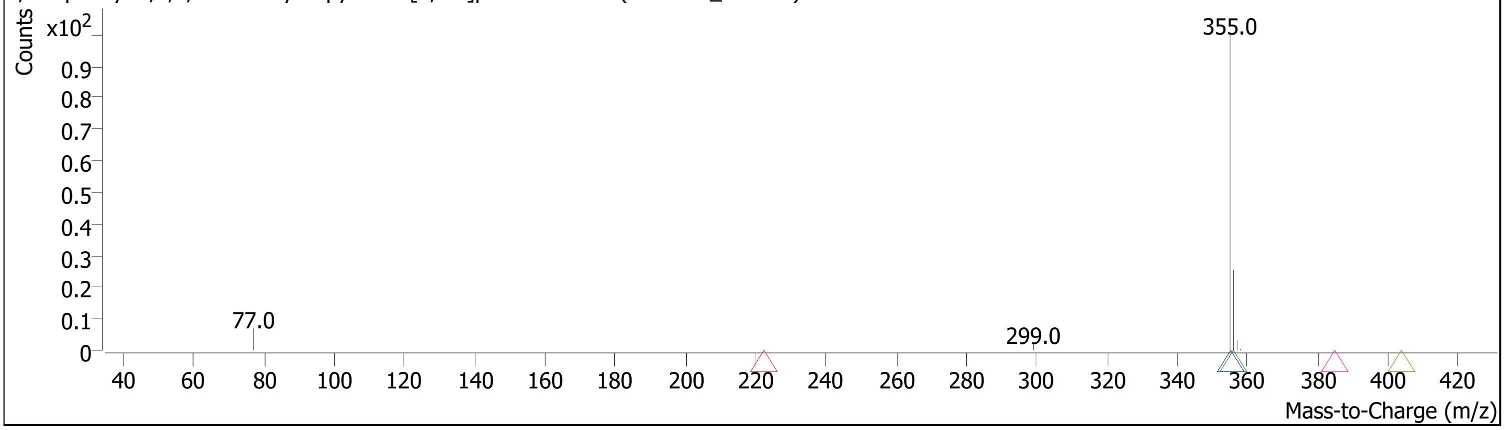

+ Scan (51.1030-51.1672 min, 12 scans) 11795-2.D

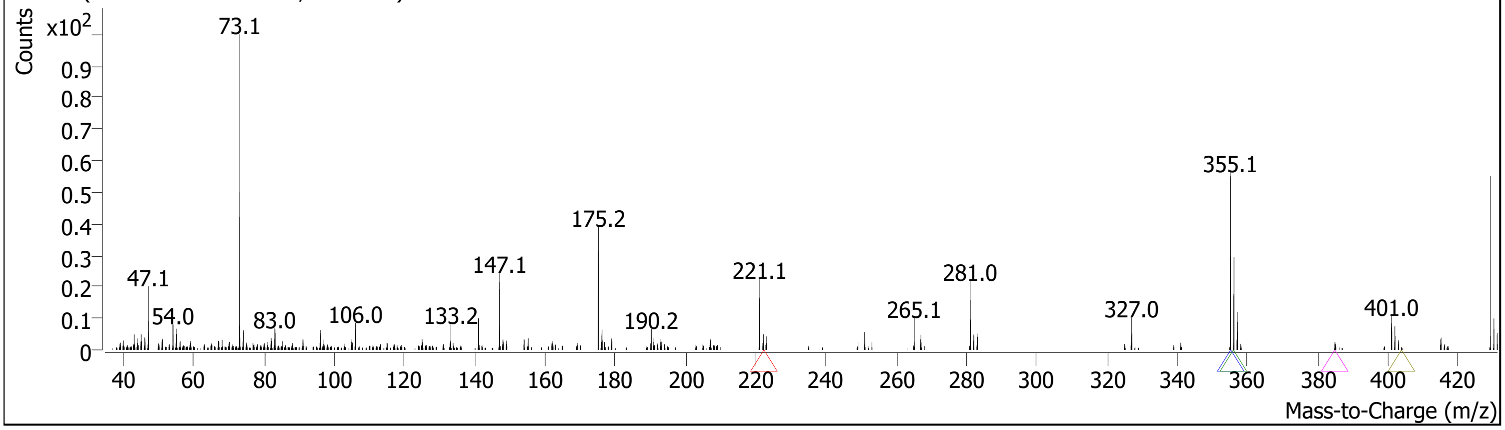

Component RT: 51.1390

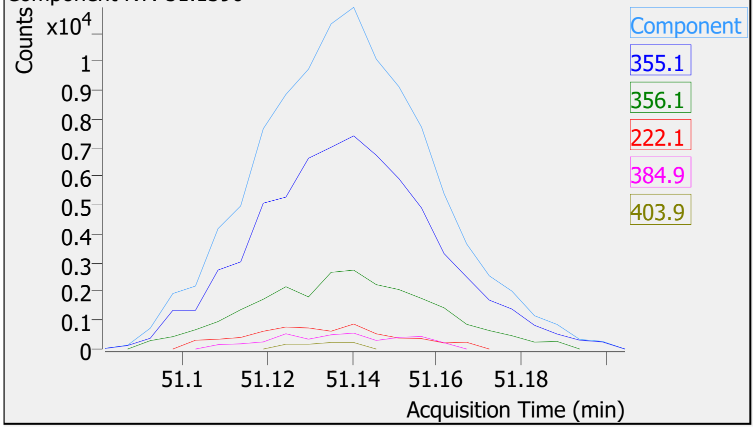

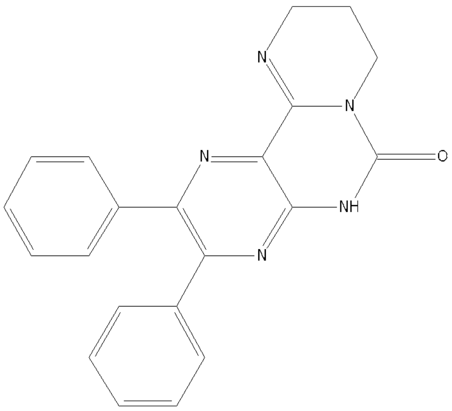

Unknown Analysis Report - Best Hits

RT	Compound Name	CAS#	Formula	Area	MI	Match Score	Sample	Sample
56.3455	5-Diazo-1,3-cyclopentadiene	1192-27-4	C5H4N2	35619		72.8	0.27	1.10

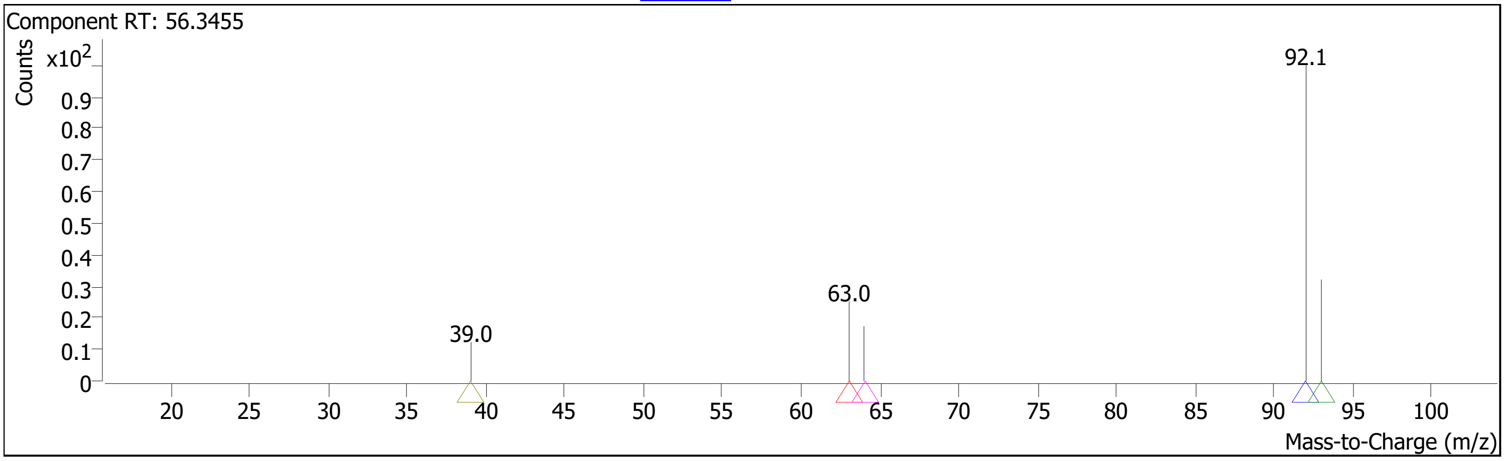

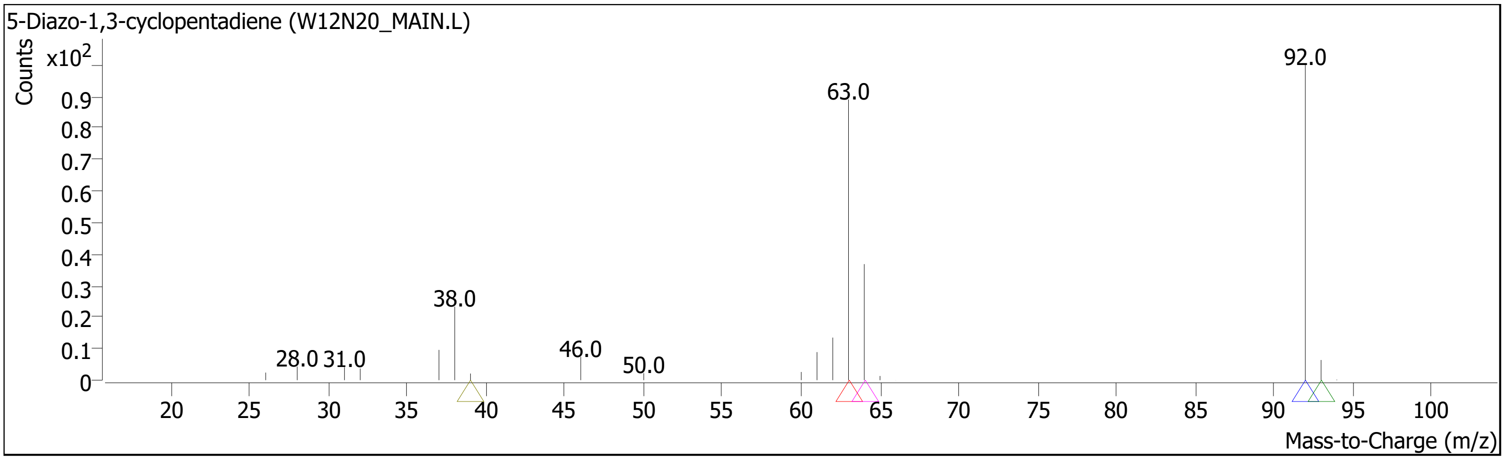

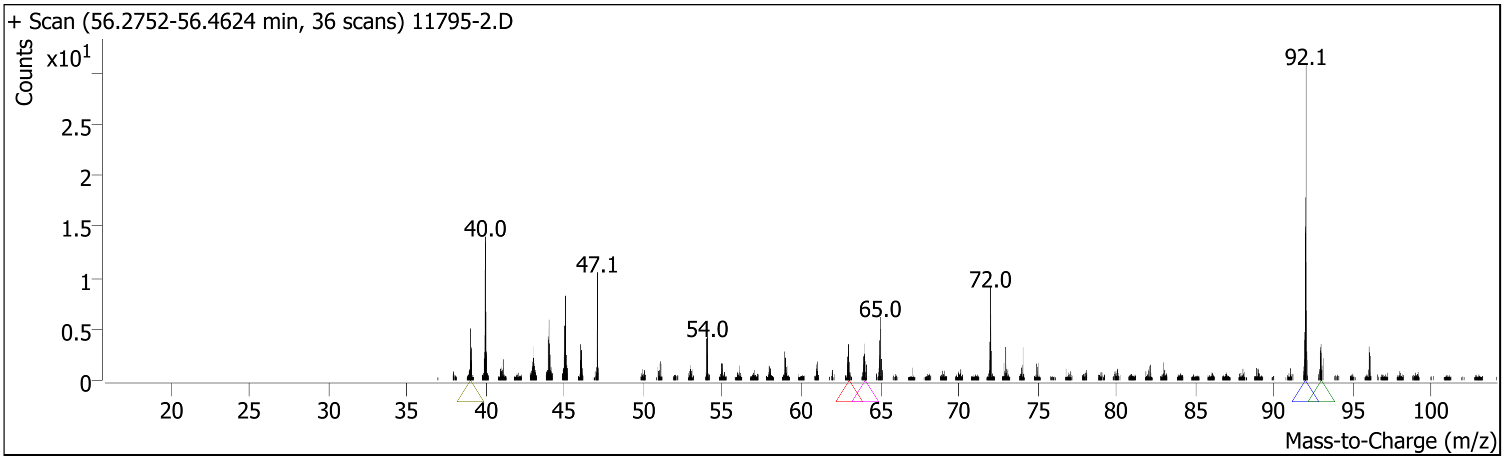

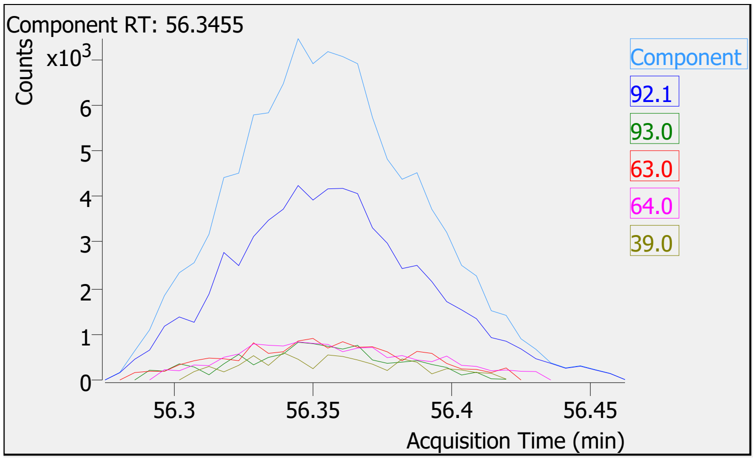

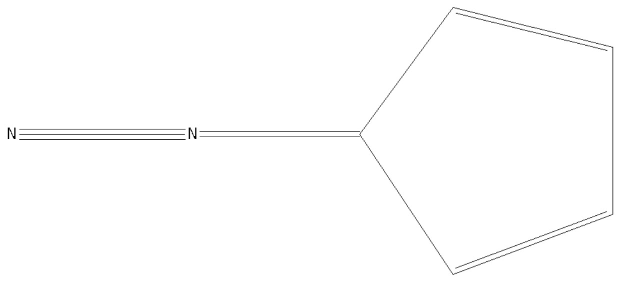

Unknown Analysis Report - Best Hits

RT	Compound Name	CAS#	Formula	Area	MI	Match Score	Sample	Sample
56.3553	Methyl salicylate	119-36-8	C8H8O3	118665		92.5	0.91	3.66

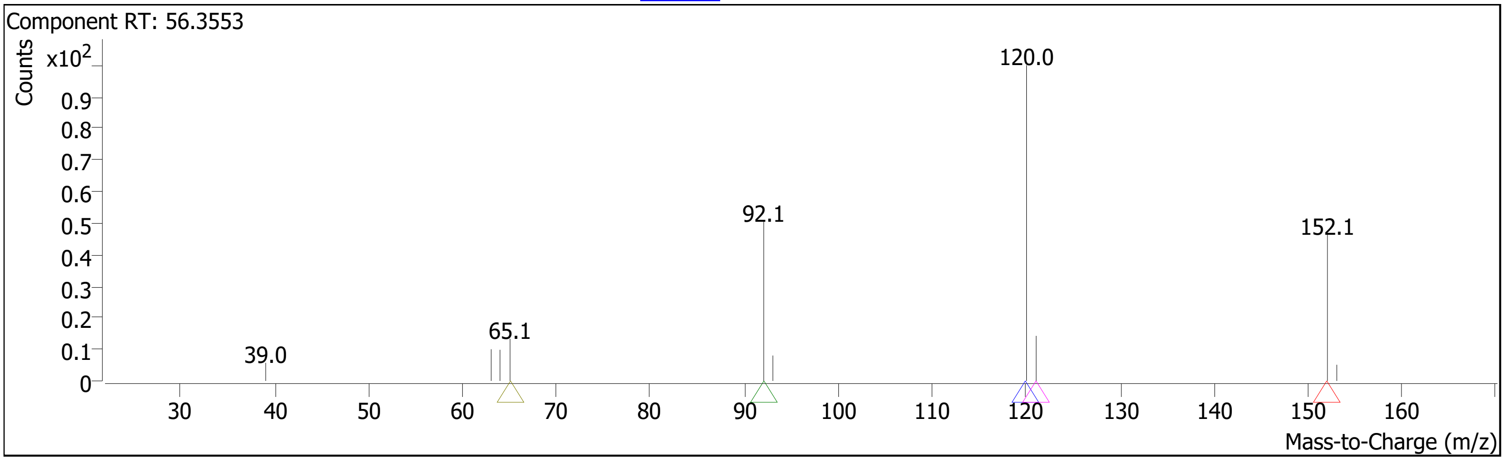

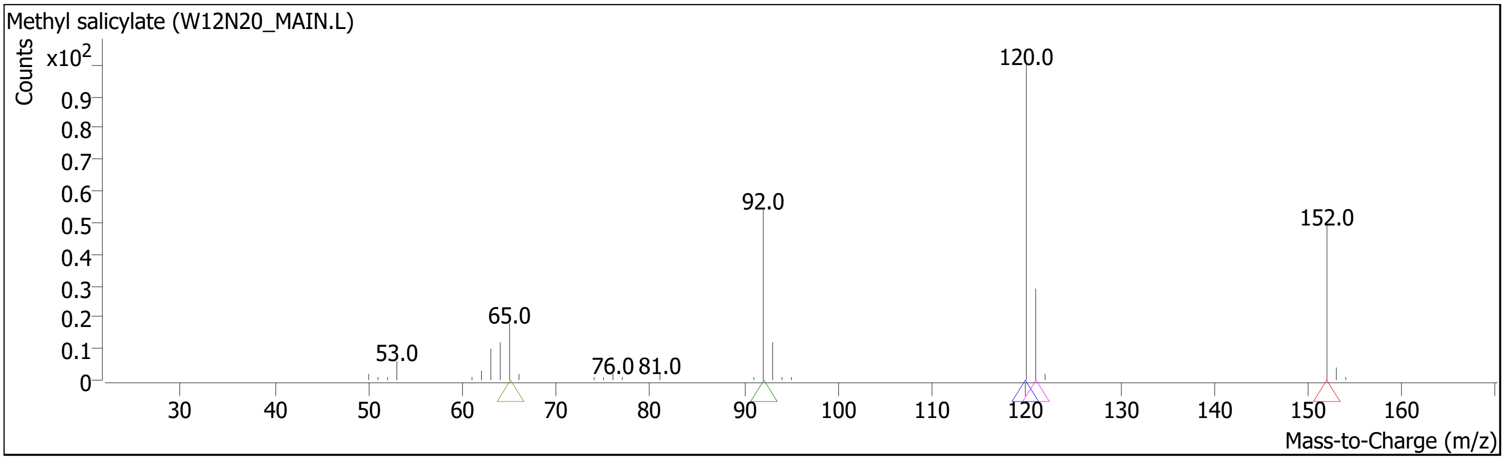

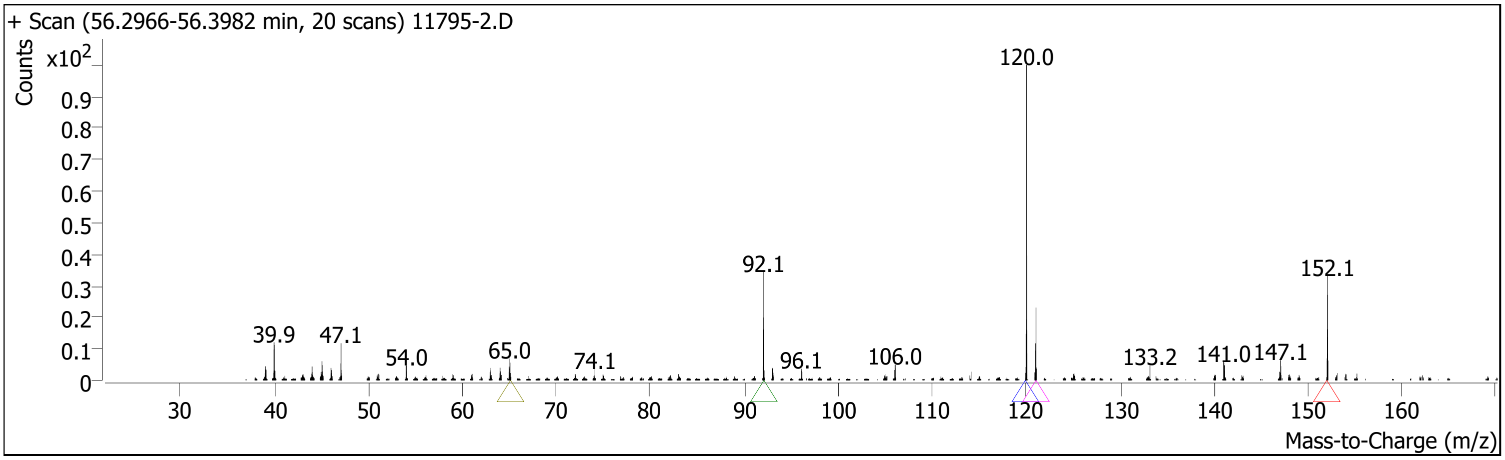

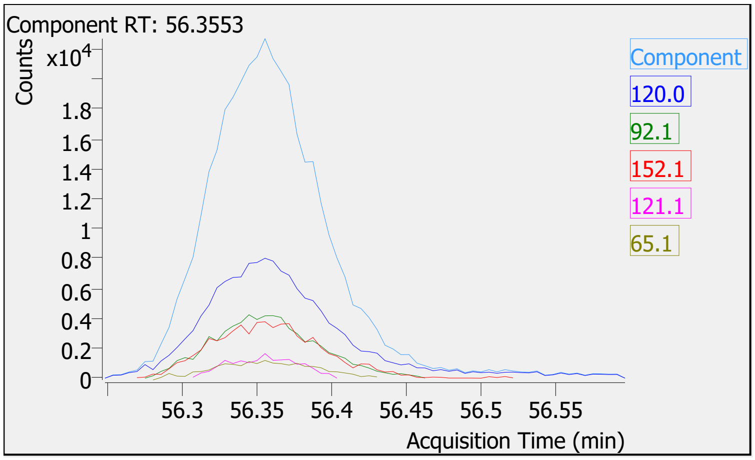

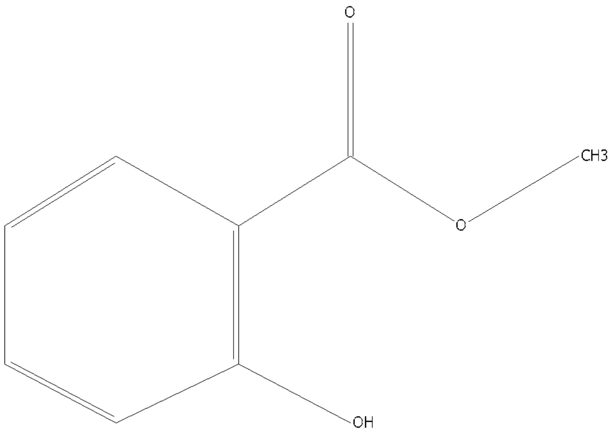

RT	Compound Name	CAS#	Formula	Area	MI	Match Score	Sample	Sample
56.4253	2-Acetamidopentyl acetate	990051-78-0	C9H17NO3	14217		88.8	0.11	0.44

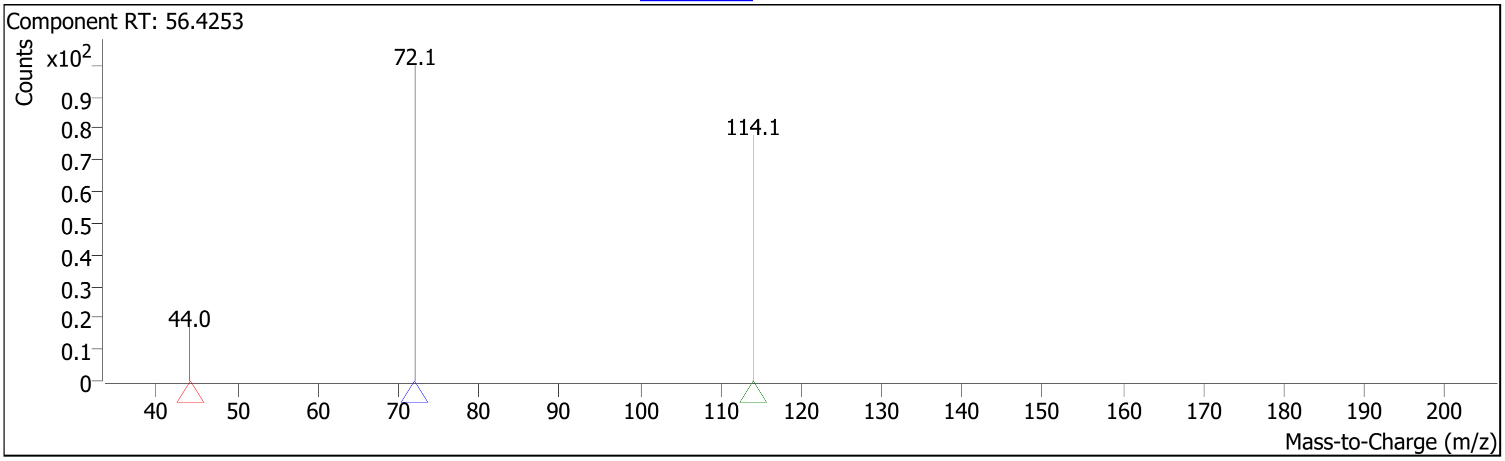

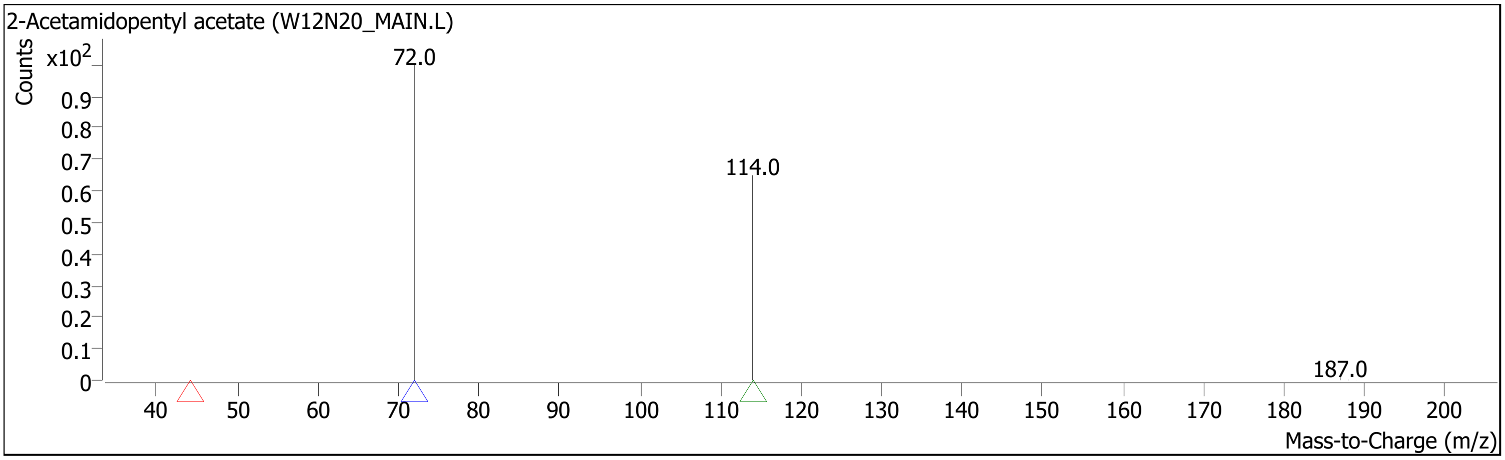

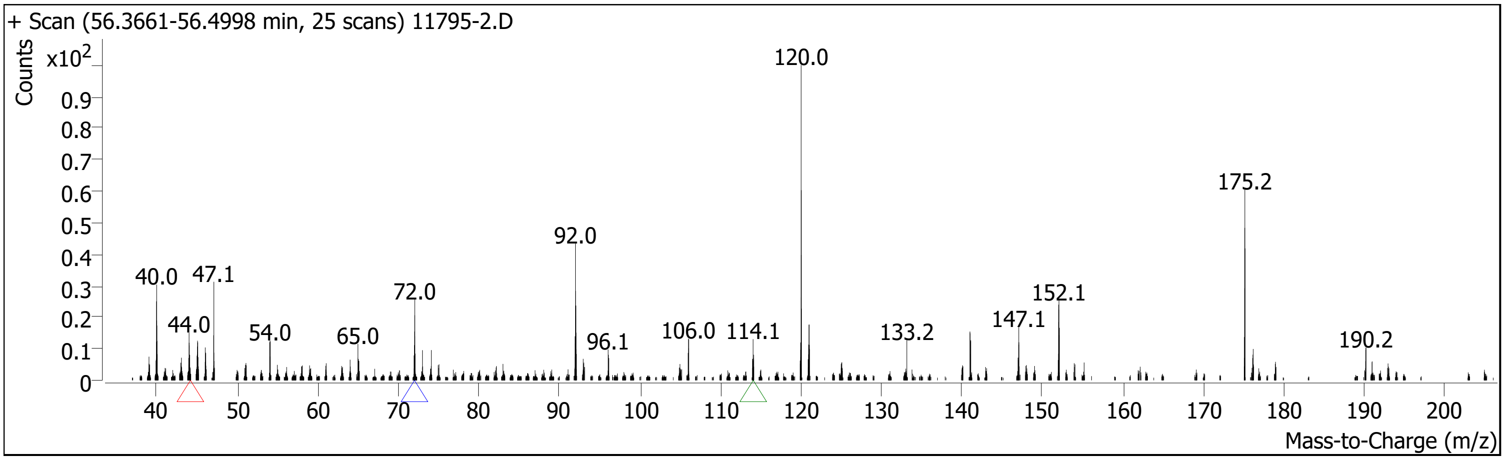

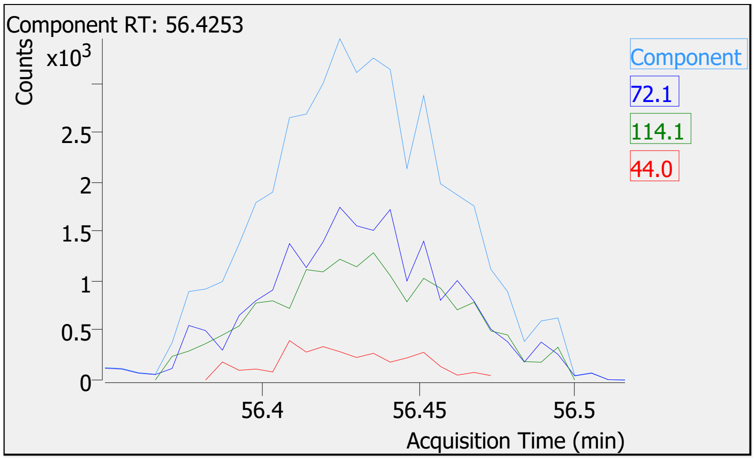

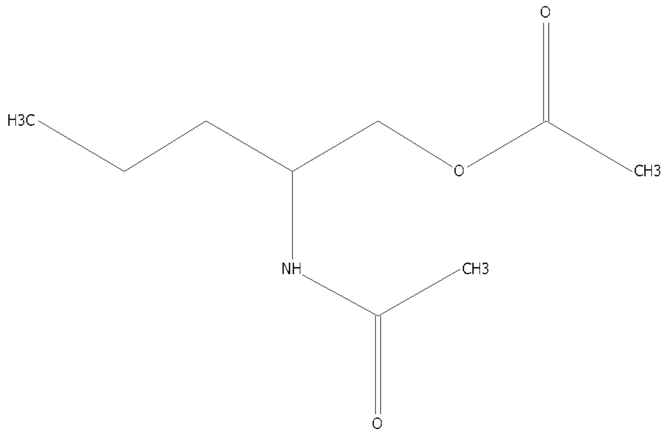

RT	Compound Name	CAS#	Formula	Area	MI	Match Score	Sample	Sample
59.0747	4-Methylphthalide	990013-04-1	C9H8O2	39555		84.9	0.30	1.22

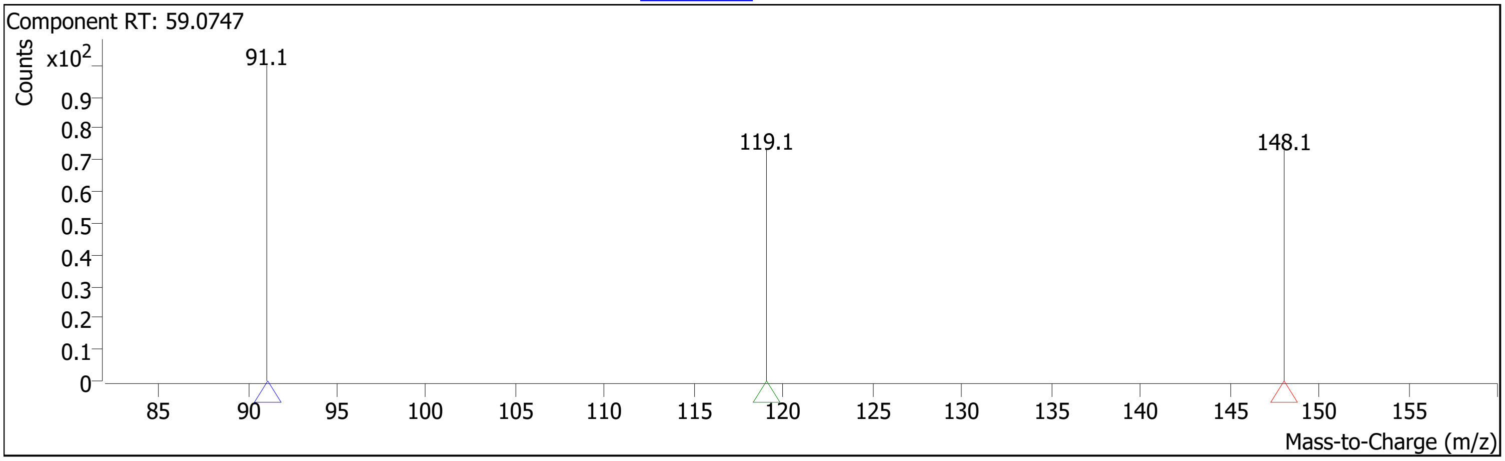

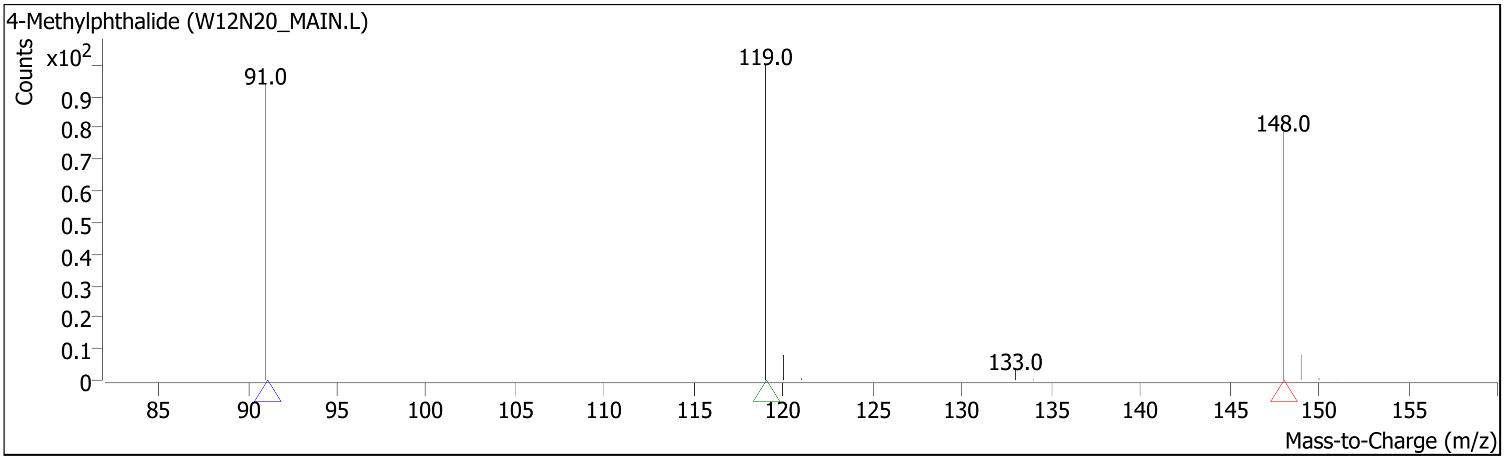

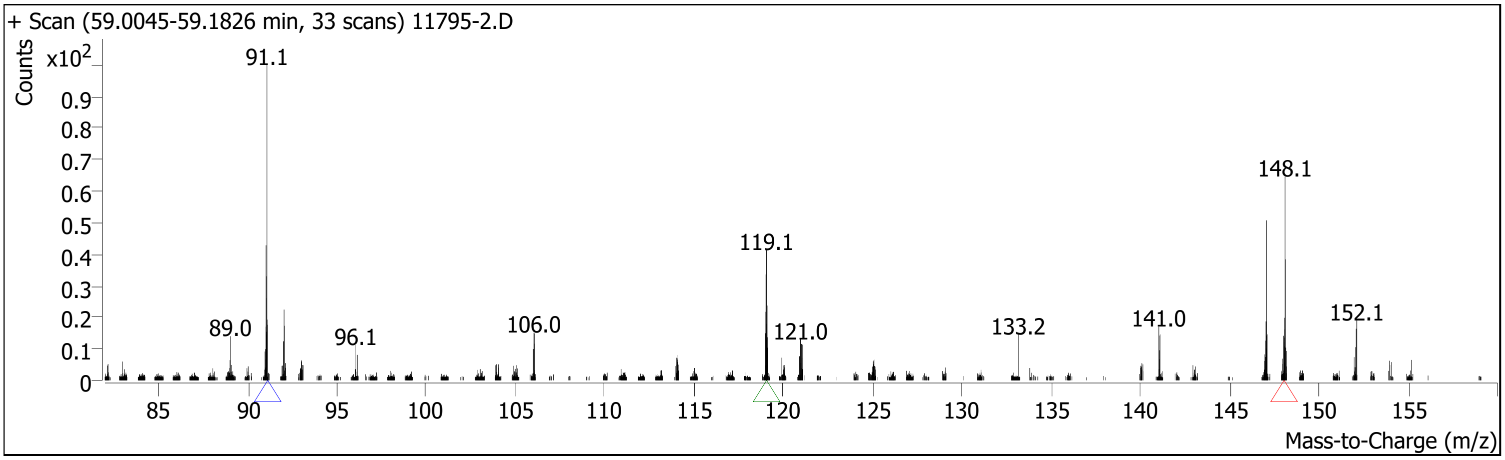

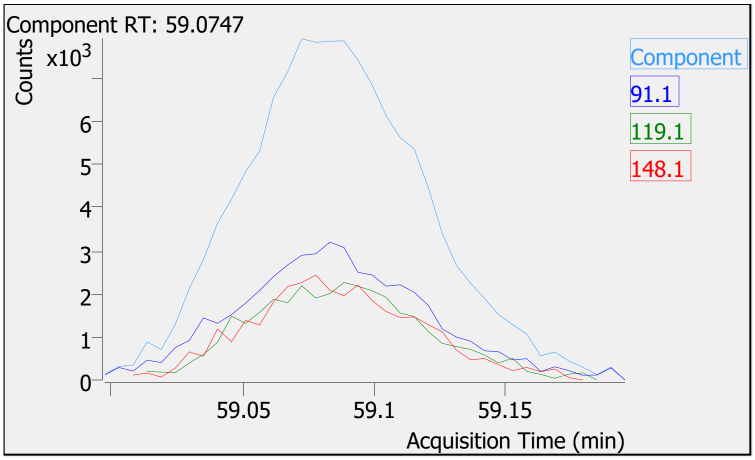

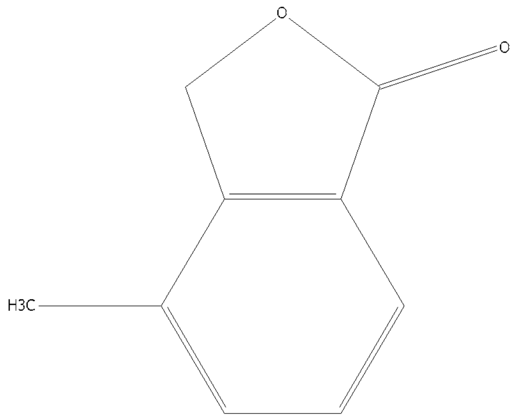

Generated at 4:32 PM on 11/14/2023

Unknown Analysis Report - Best Hits

RT	Compound Name	CAS#	Formula	Area	MI	Match Score	Sample	Sample
61.7813	(Z,Z)-(+-)-cis-2-(2,5-Octdienyl)-3-undecyloxirane	990320-72-3	C21H38O	195491		91.3	1.50	6.03

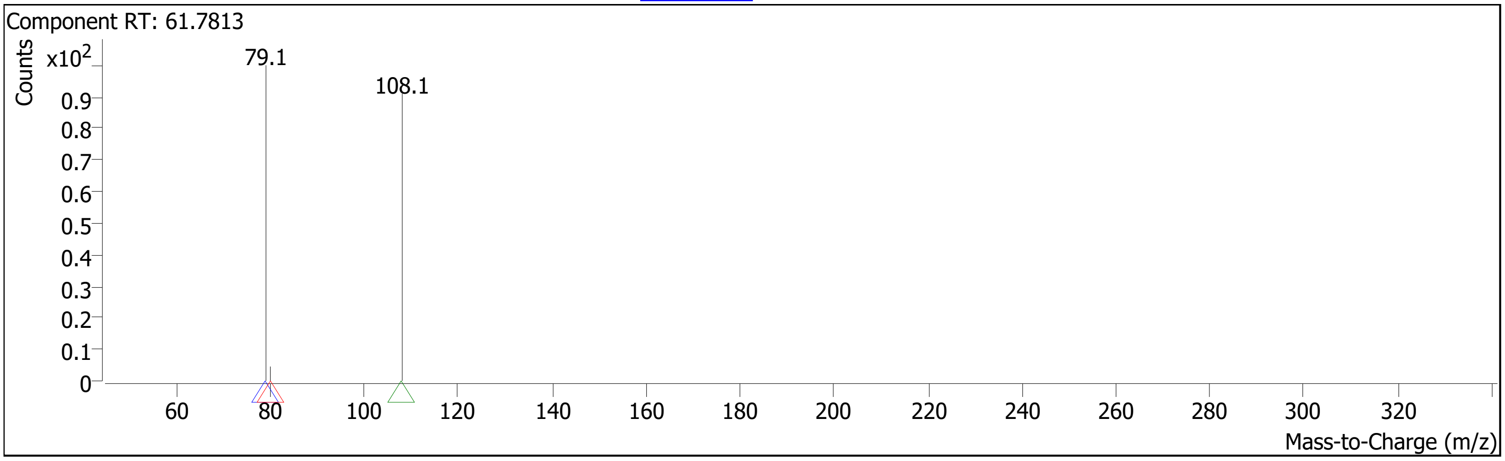

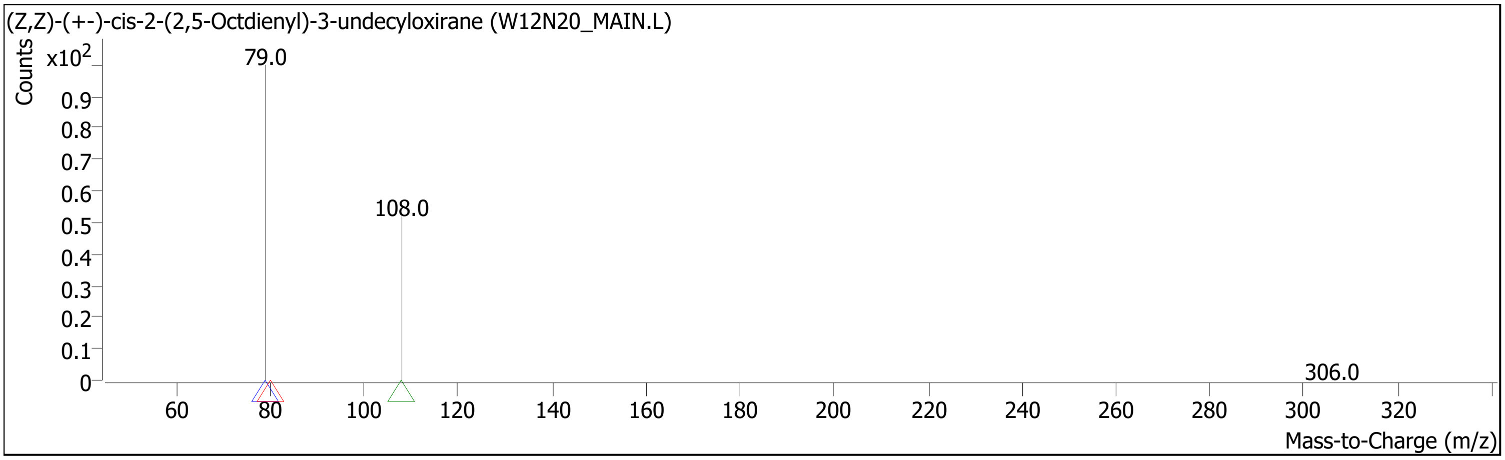

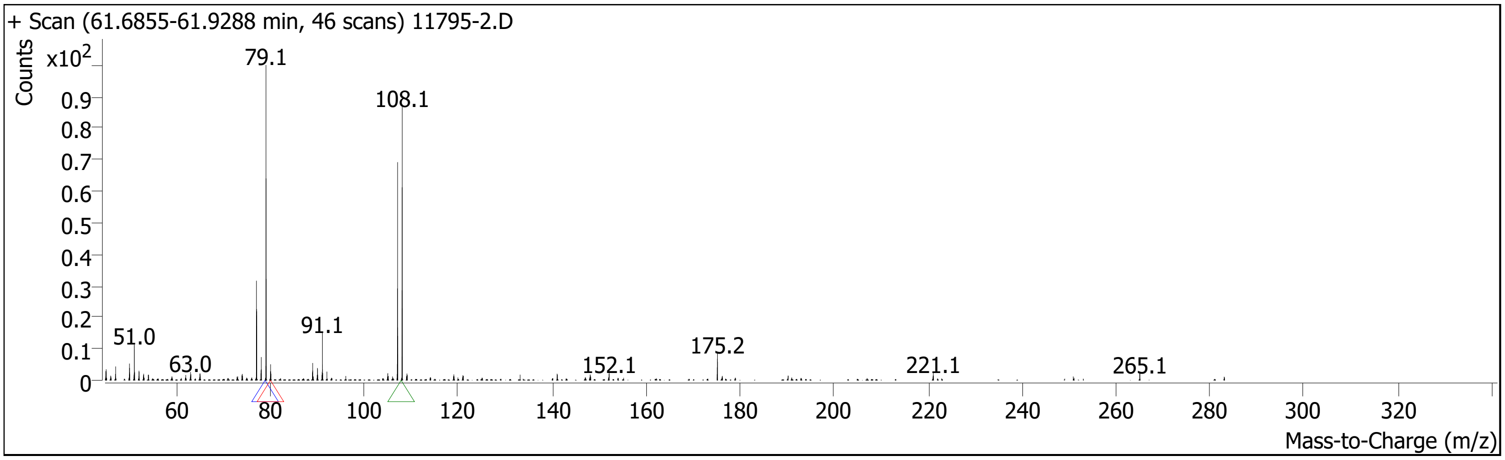

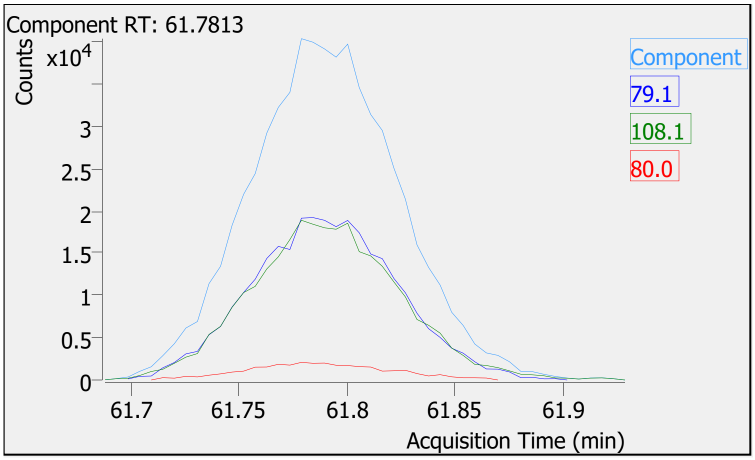

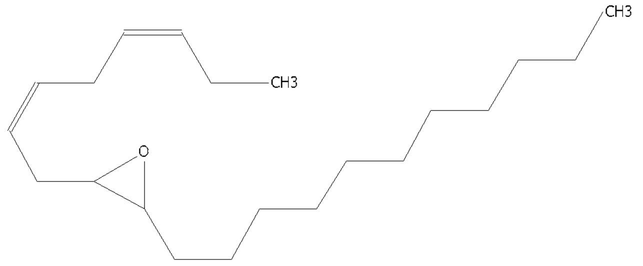

Unknown Analysis Report - Best Hits

RT	Compound Name	CAS#	Formula	Area	MI	Match Score	Sample	Sample
61.7888	(R)-S-Ethylthio mandelate	990065-39-2	C10H12O2S	134907		86.2	1.04	4.16

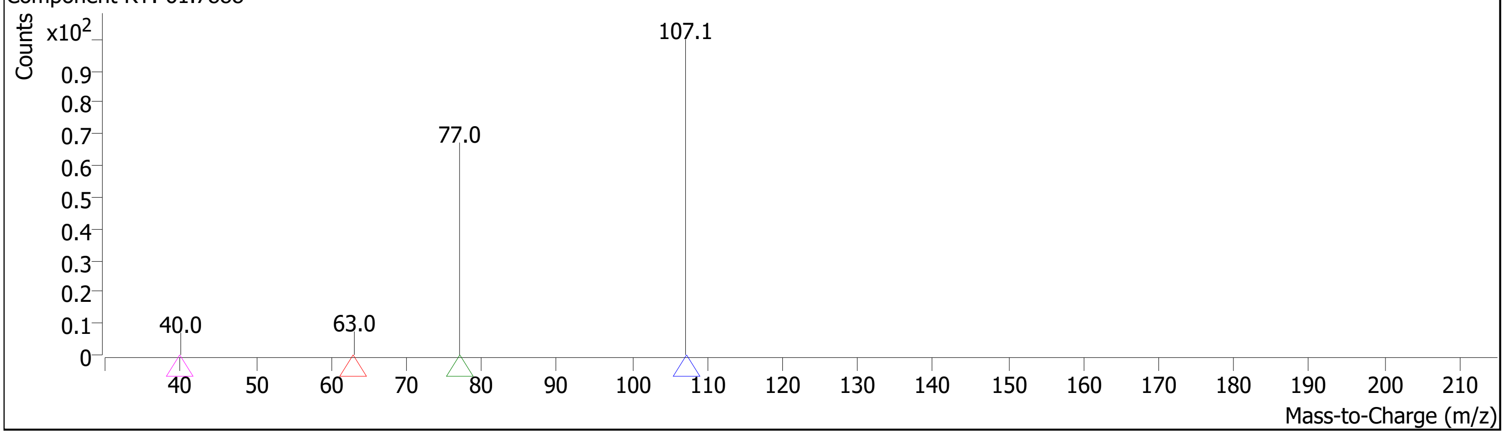

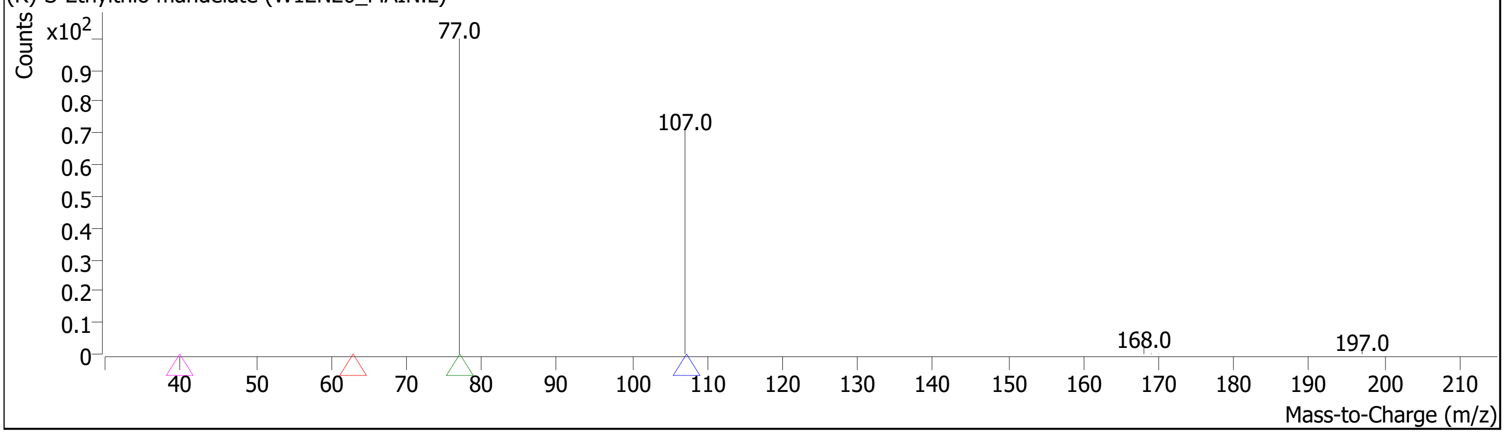

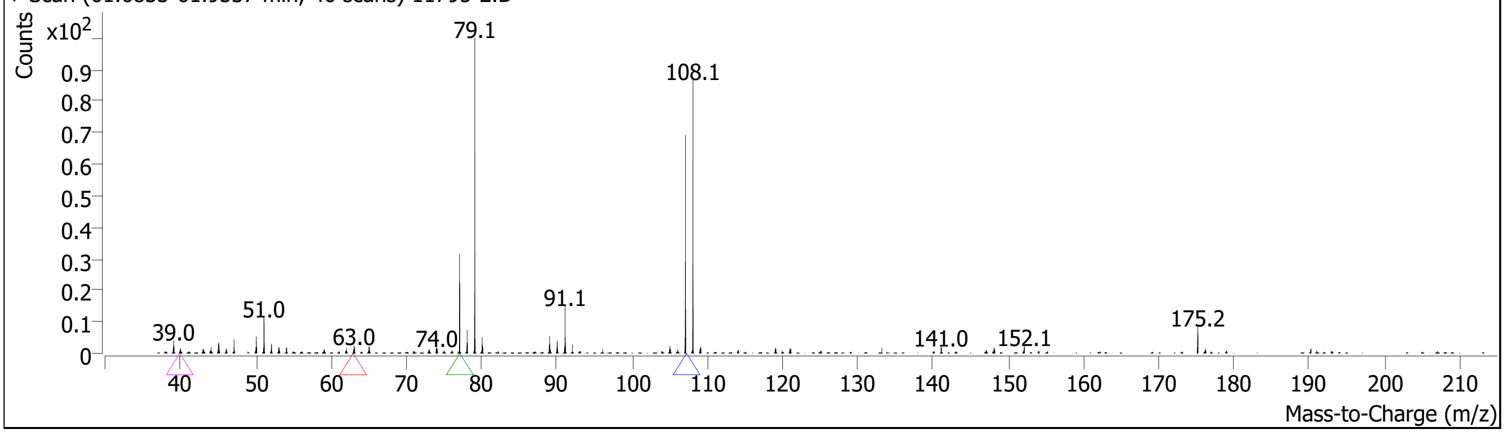

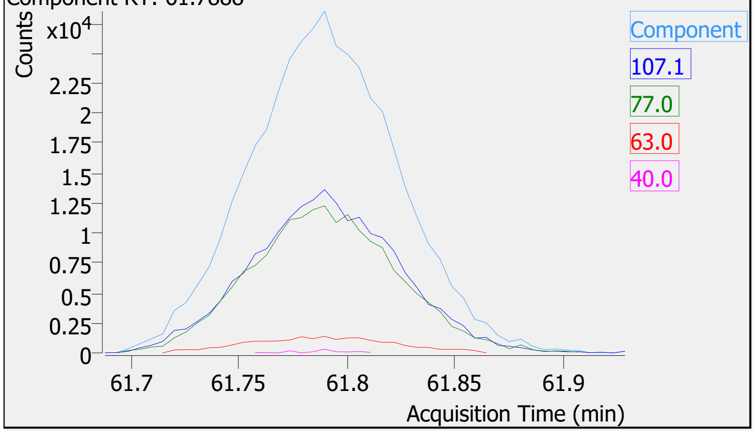

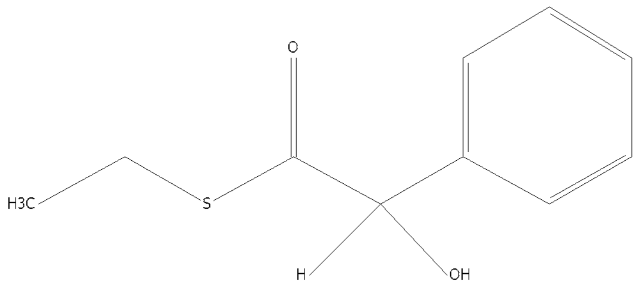

Unknown Analysis Report - Best Hits

RT	Compound Name	CAS#	Formula	Area	MI	Match Score	Sample	Sample
66.7159	Bis-(3,5,5-trimethylhexyl) ether	990229-48-0	C18H38O	44402		86.0	0.34	1.37

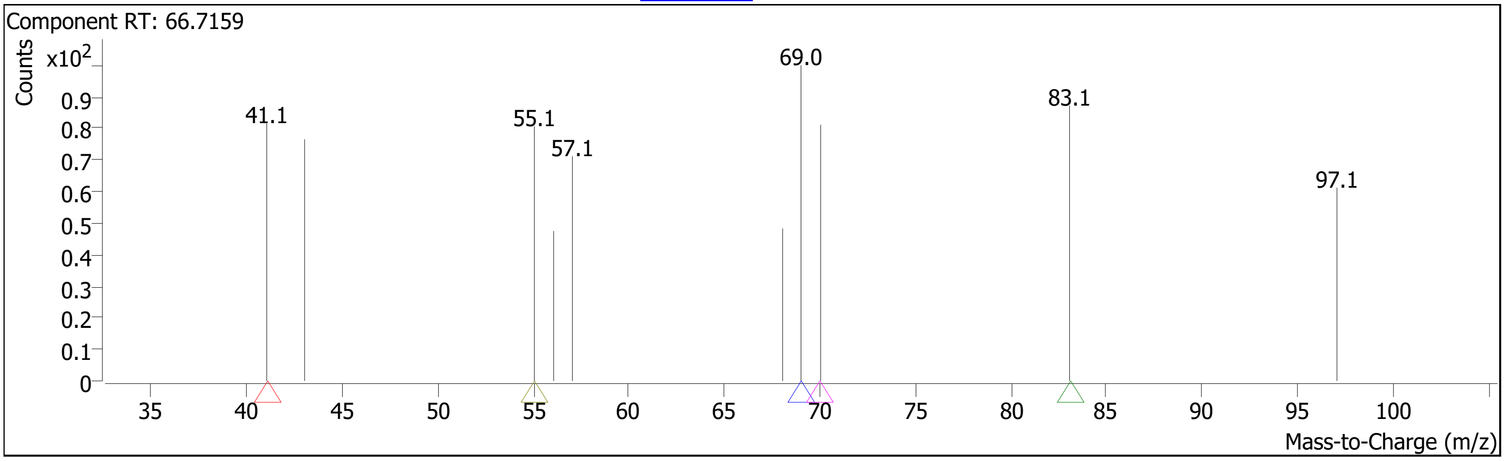

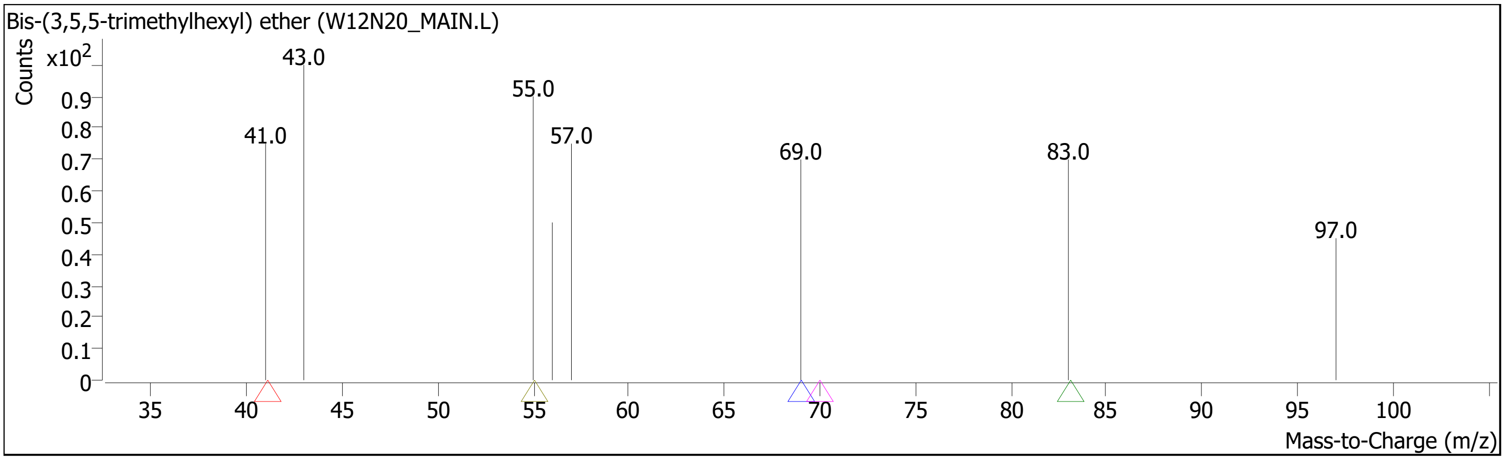

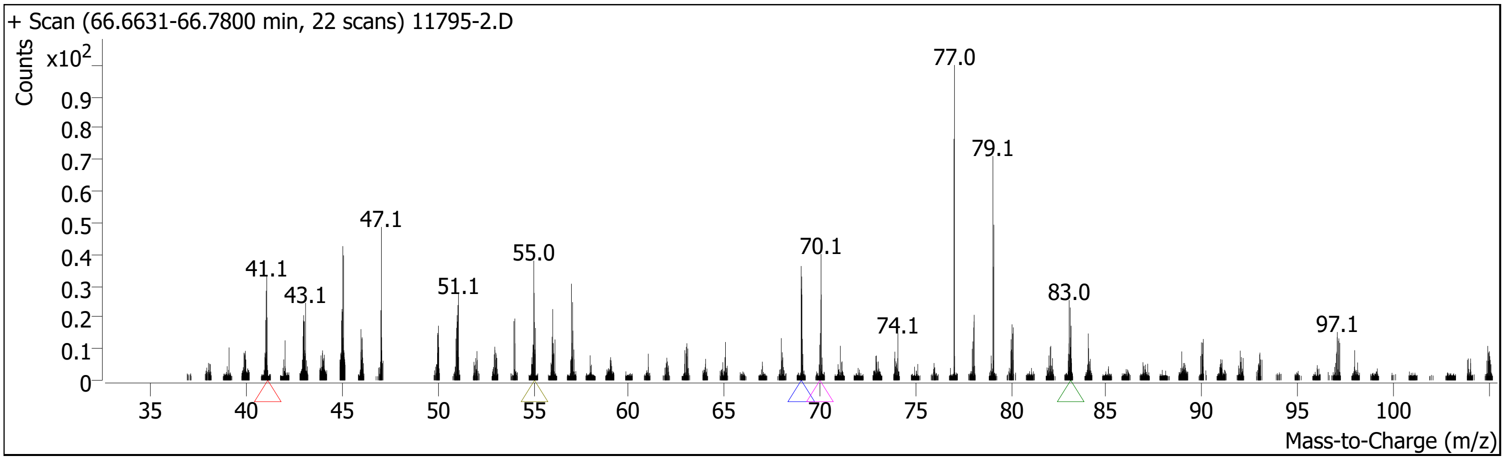

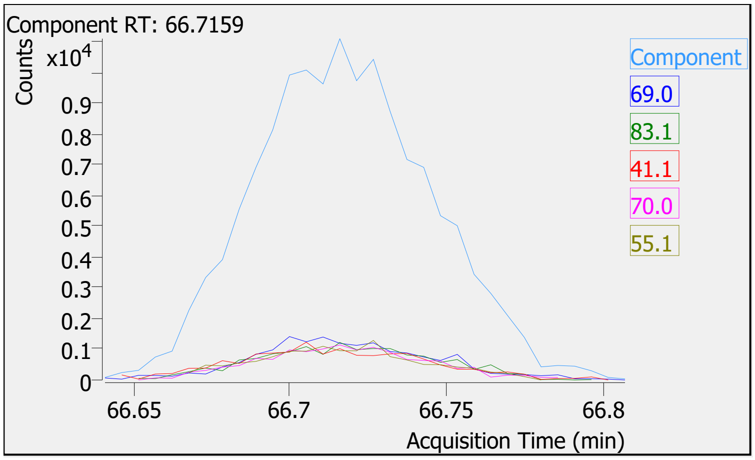

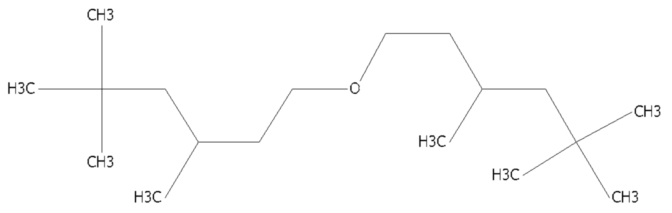

 Generated at 4:32 PM on 11/14/2023

RT	Compound Name	CAS#	Formula	Area	MI	Match Score	Sample	Sample
71.6198	(phenylmethyl) undec-10-ynoate	990234-29-8	C18H24O2	63156		90.4	0.49	1.95

Component RT: 71.6198

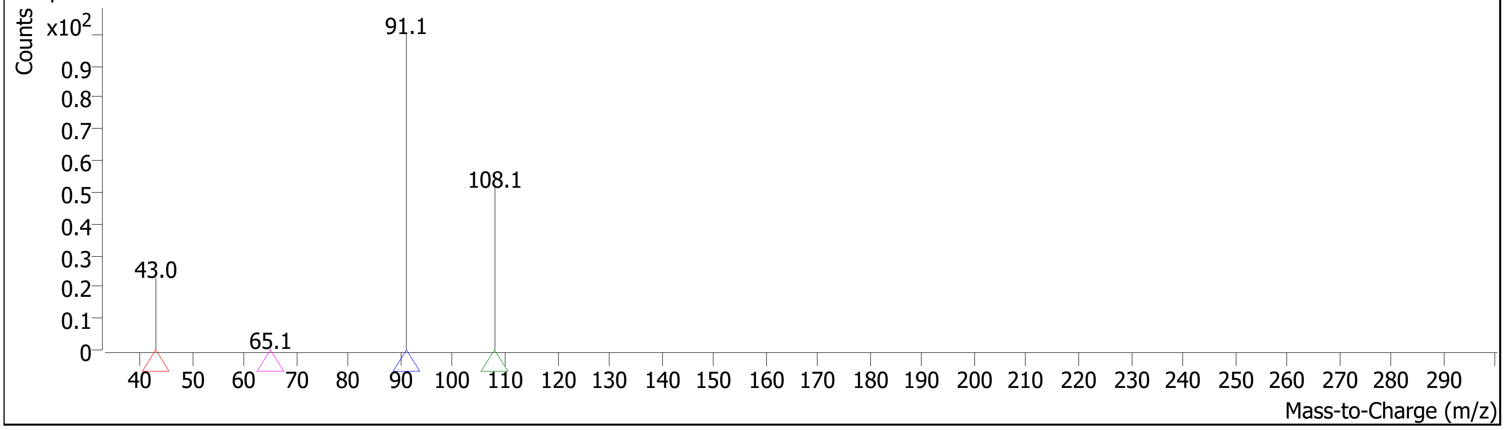

(phenylmethyl) undec-10-ynoate (W12N20_MAIN.L)

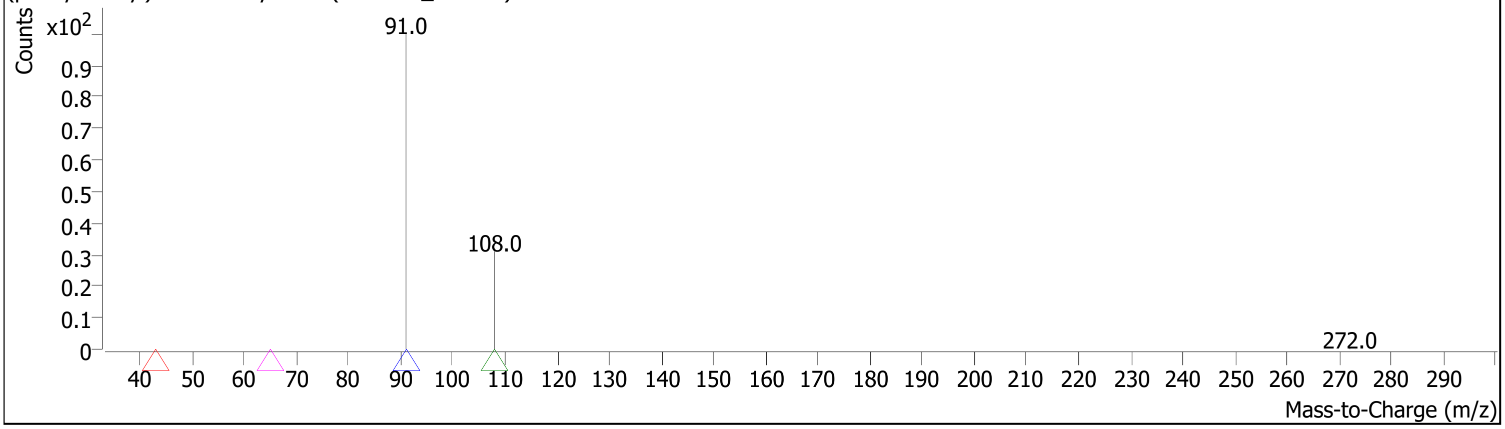

+ Scan (71.5381-71.7158 min, 33 scans) 11795-2.D

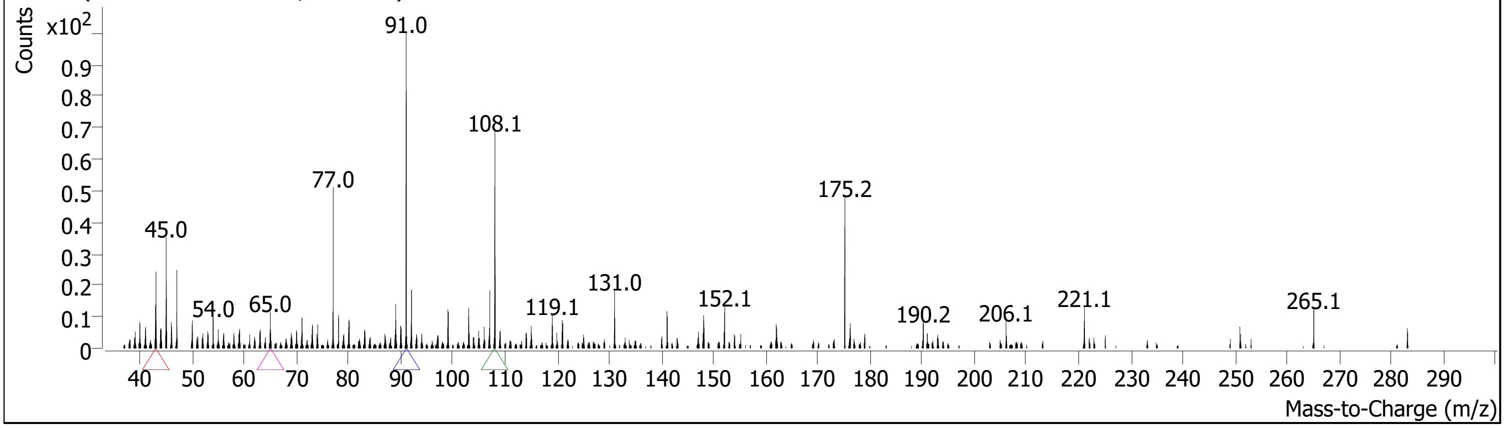

Component RT: 71.6198

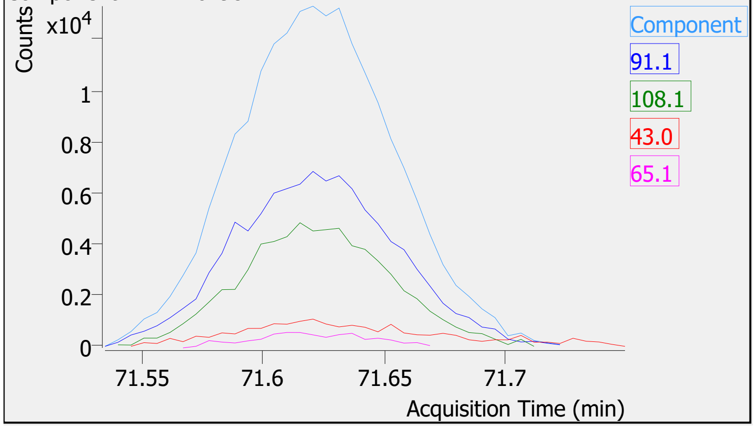

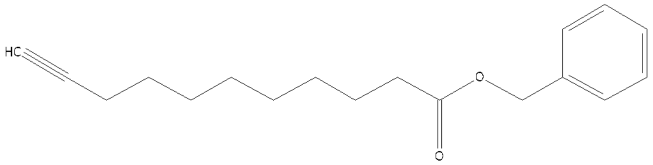

 Generated at 4:32 PM on 11/14/2023

RT	Compound Name	CAS#	Formula	Area	MI	Match Score	Sample	Sample
71.6832	2-Methoxyindan-1-one	990023-11-2	C10H10O2	12047		78.2	0.09	0.37

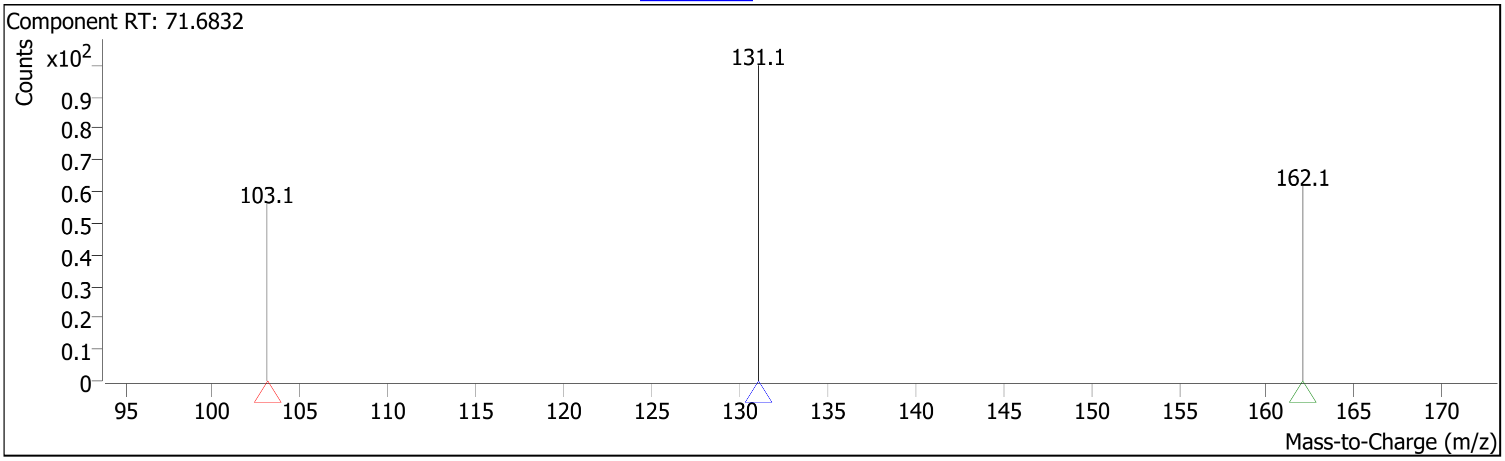

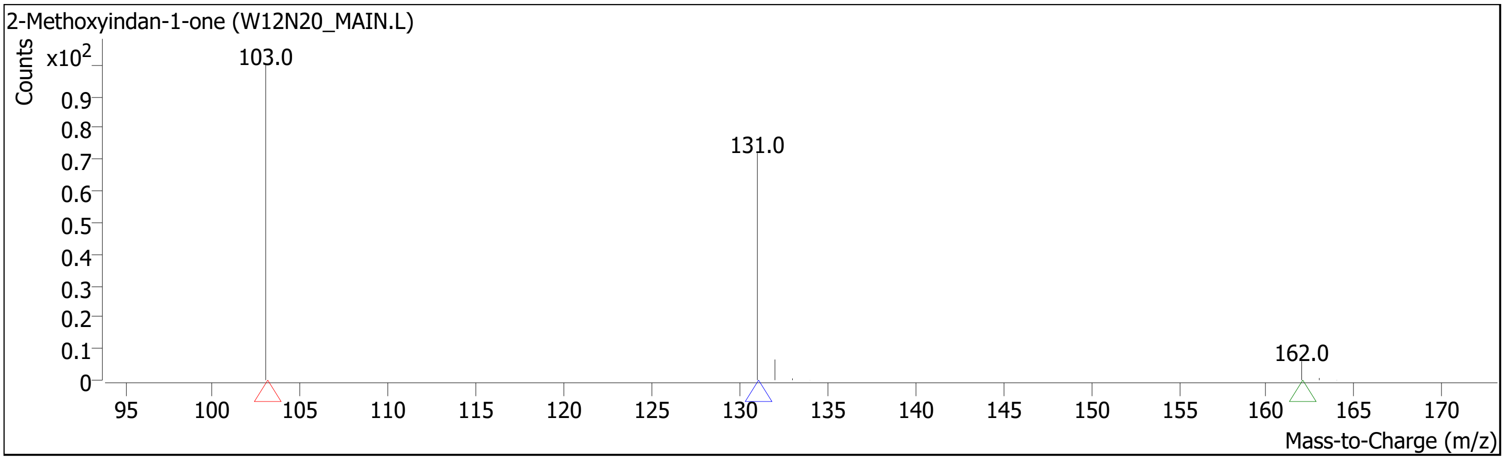

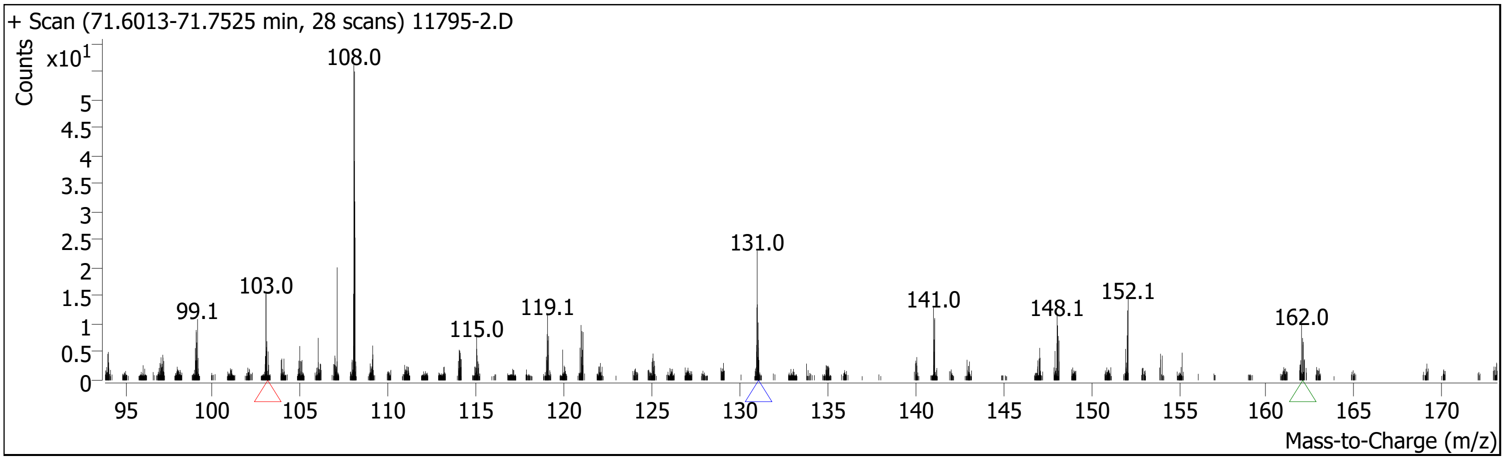

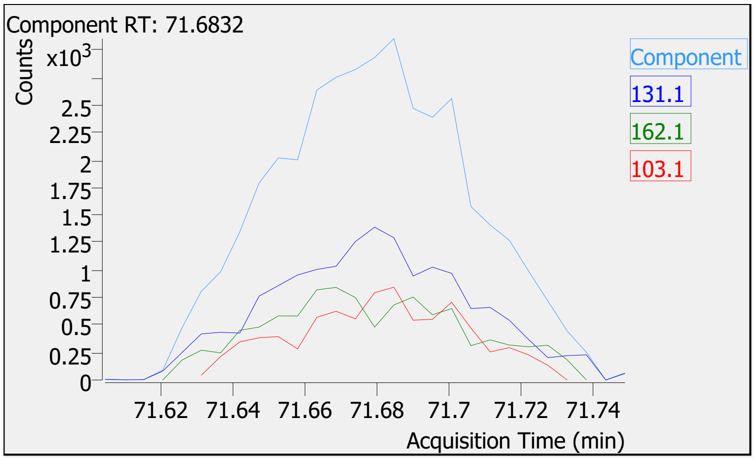

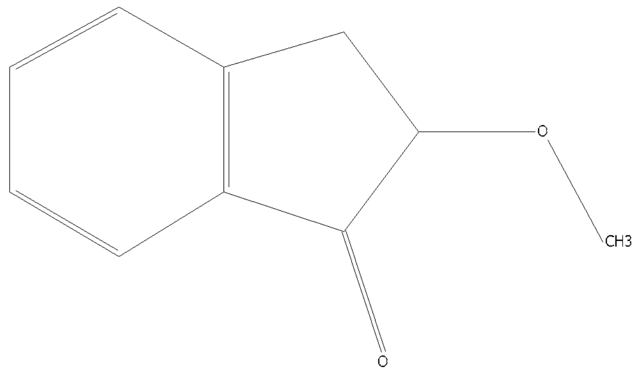

RT	Compound Name	CAS#	Formula	Area	MI	Match Score	Sample	Sample
74.8737	2(3H)-Furanone, 5-hexyldihydro-	706-14-9	C10H18O2	529410		93.2	4.07	16.32

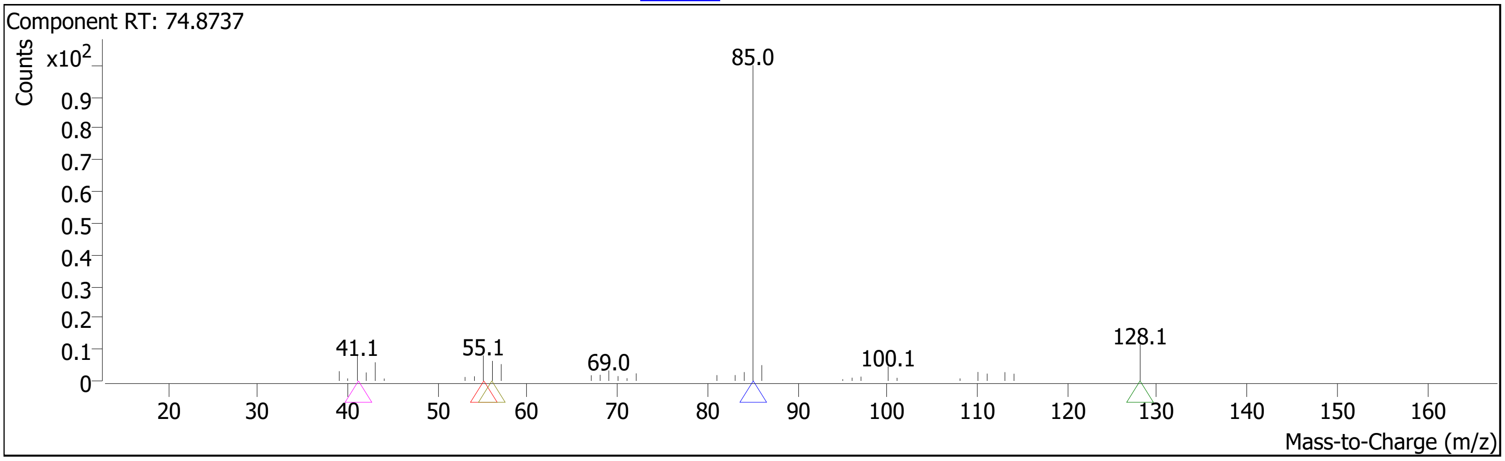

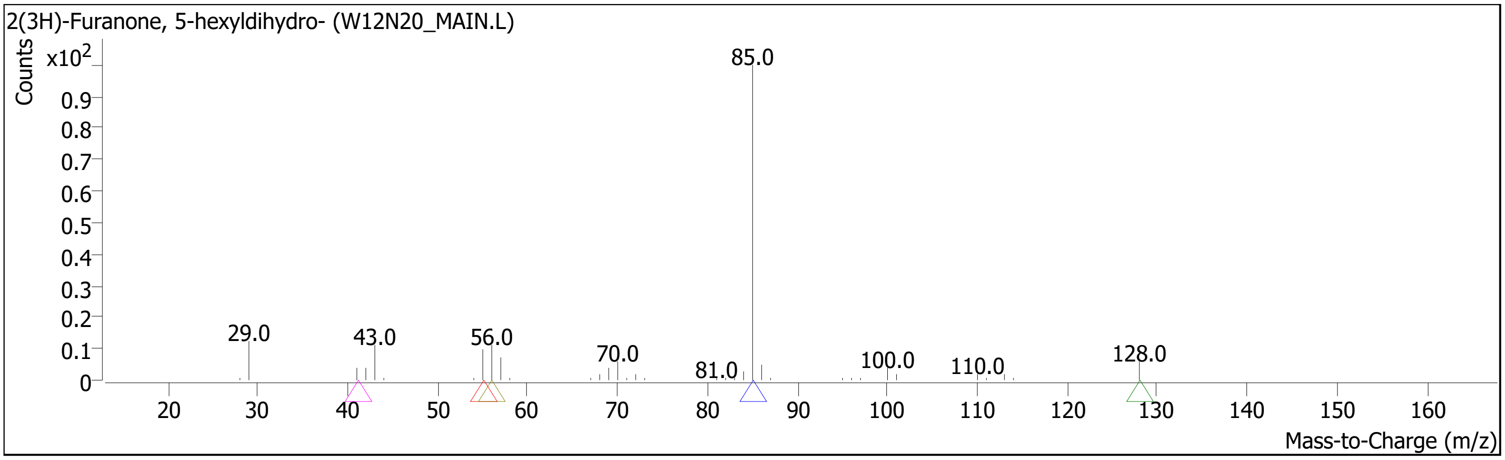

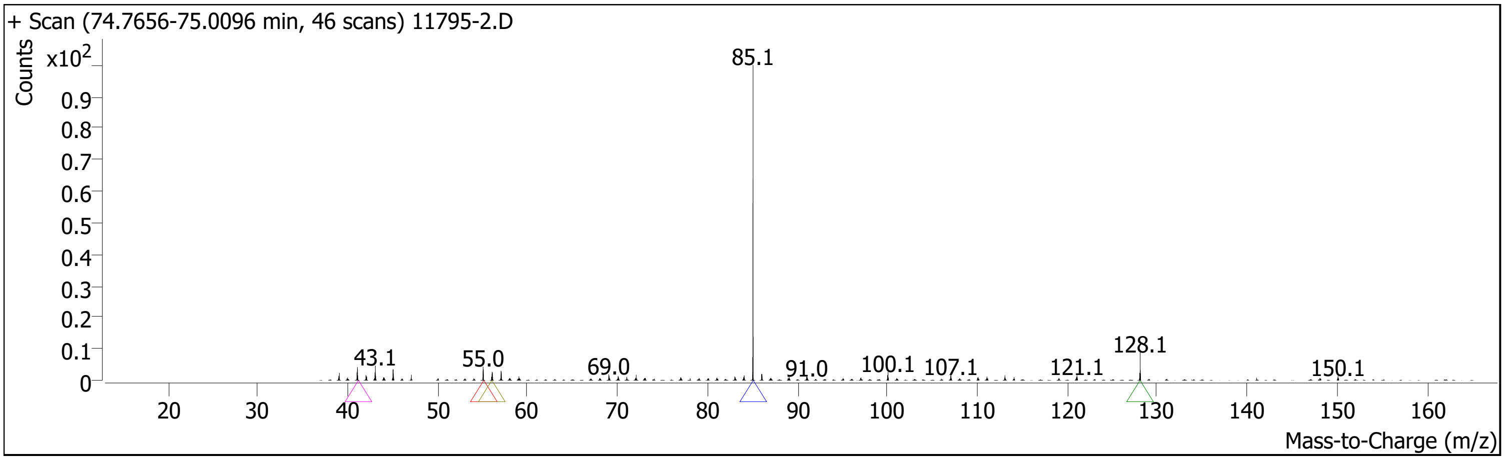

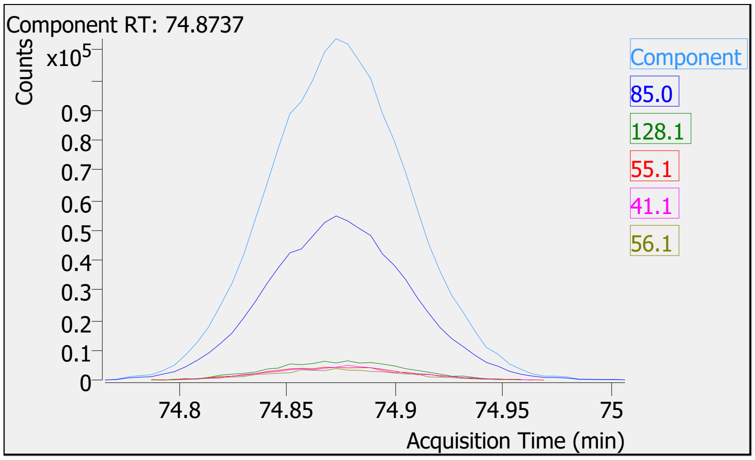

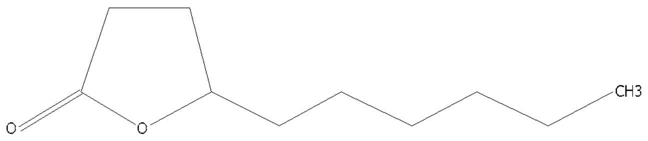

Unknown Analysis Report - Best Hits

RT	Compound Name	CAS#	Formula	Area	MI	Match Score	Sample	Sample
74.8827	Methyl (2RS,3RS)-3-Acetoxy-2-[(SR)-1-hydroxyethyl]butanoate	990078-31-9	C9H16O5	35865		74.9	0.28	1.11

Component RT: 74.8827

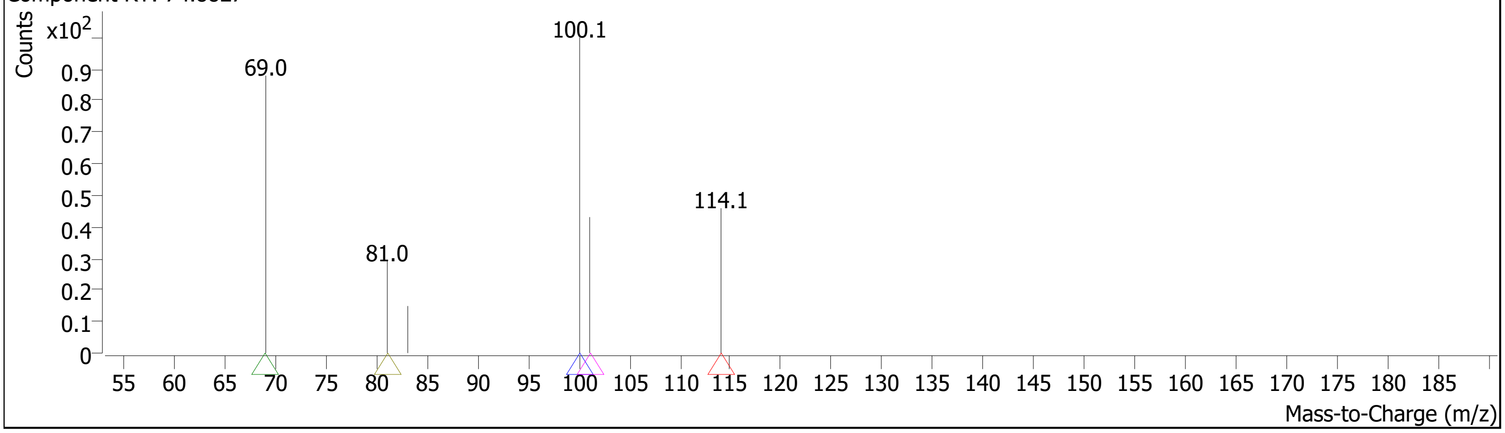

Methyl (2RS,3RS)-3-Acetoxy-2-[(SR)-1-hydroxyethyl]butanoate (W12N20_MAIN.L)

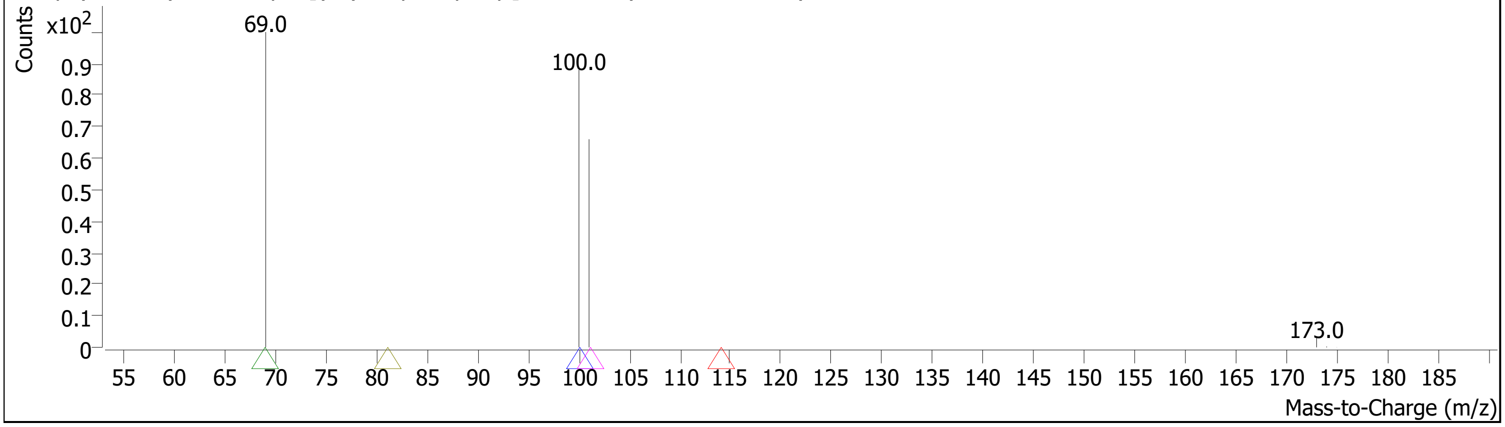

+ Scan (74.7930-74.9575 min, 30 scans) 11795-2.D

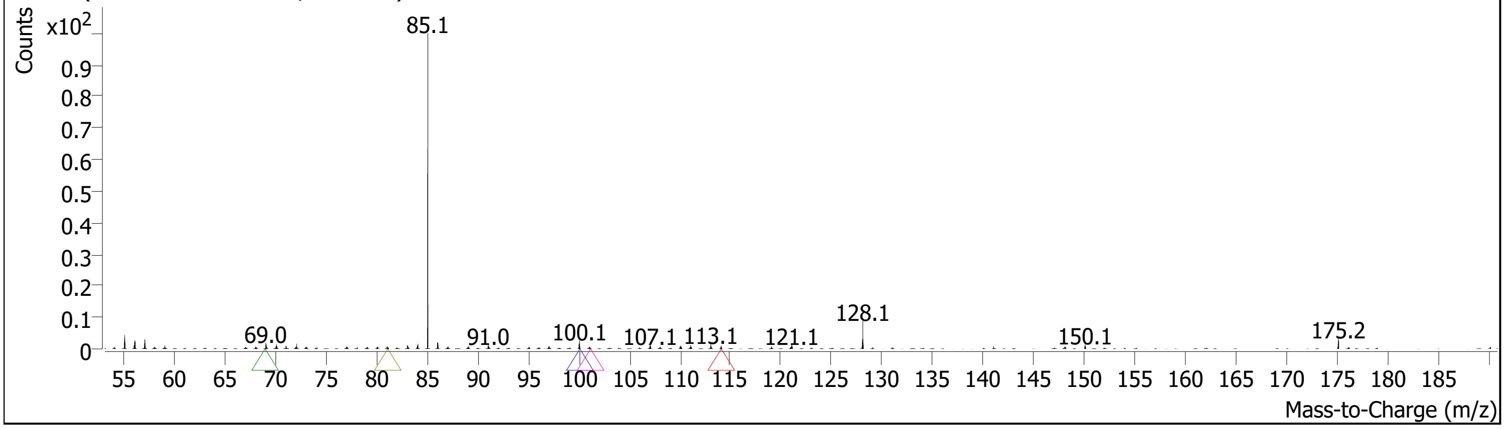

Component RT: 74.8827

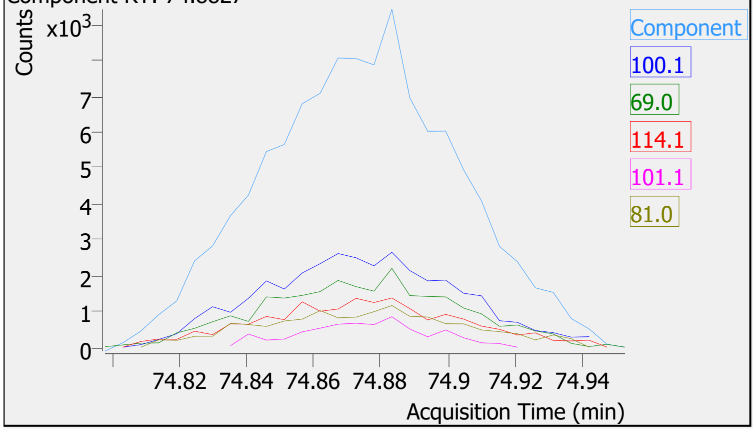

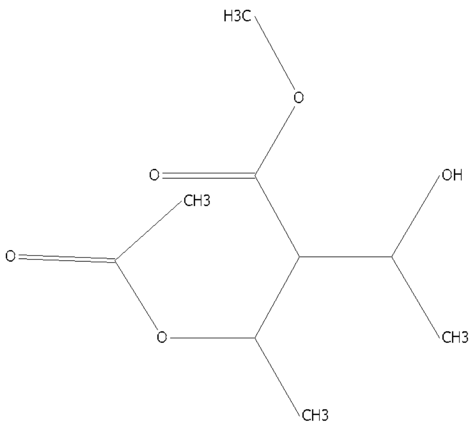

RT	Compound Name	CAS#	Formula	Area	MI	Match Score	Sample	Sample
74.9159	2H-Cyclopropa[b]naphthalen-2-one, 1,1a,2a,3,6,6a,7,7a-octahydro-1,1,2a,4,5-pentamethyl-, [1aS-(1a.alpha.,2a.beta.,6a.beta.,7a.alpha.)]-	127279-91-8	C16H24O	33019		88.7	0.25	1.02

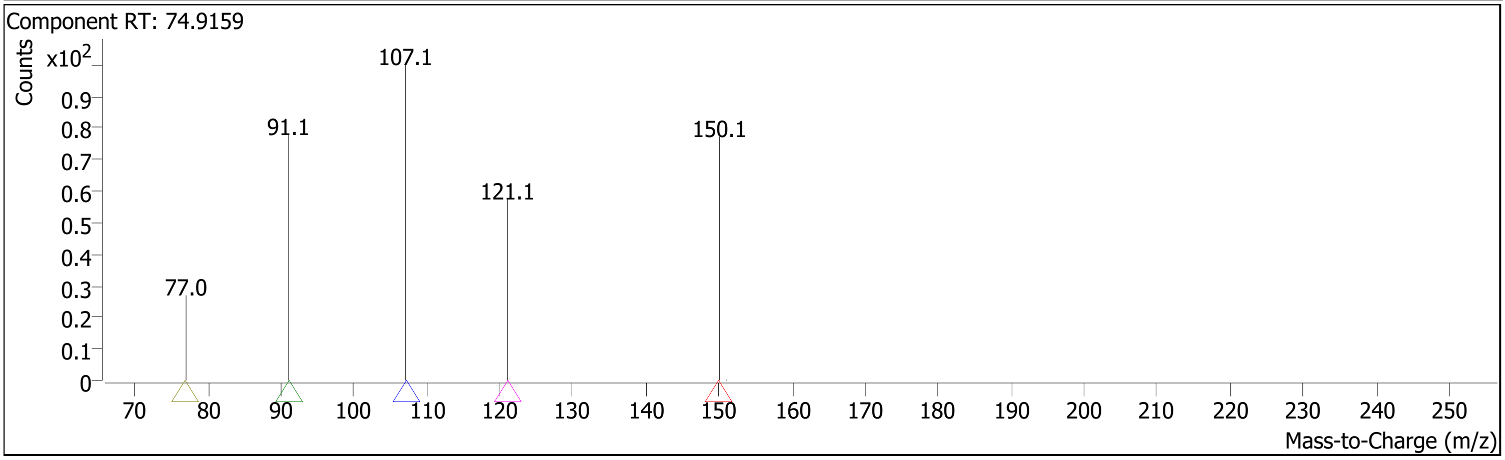

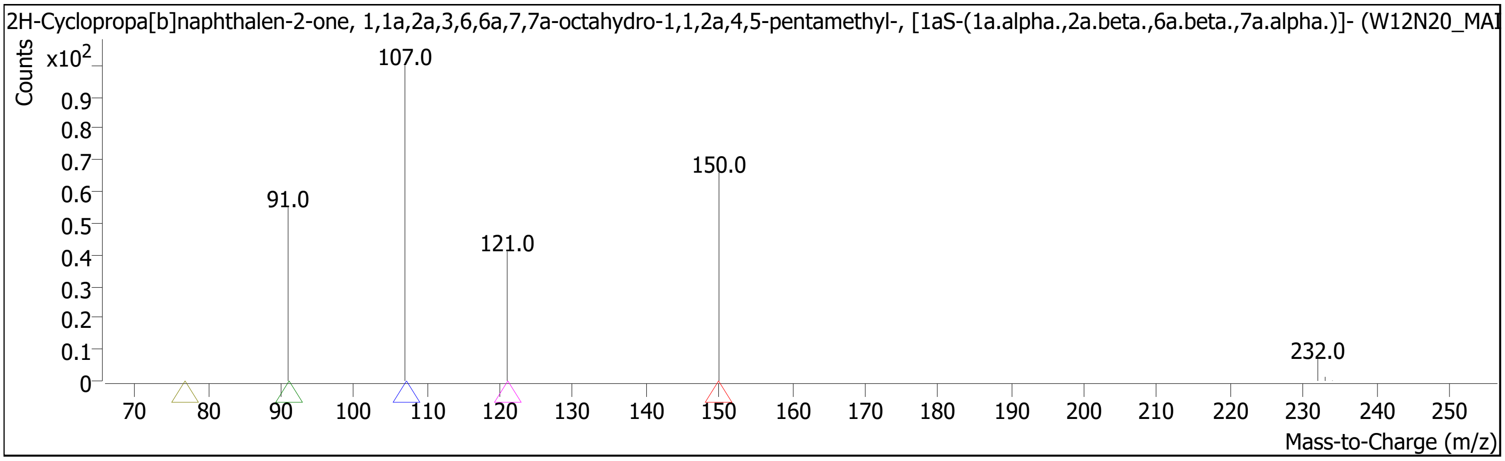

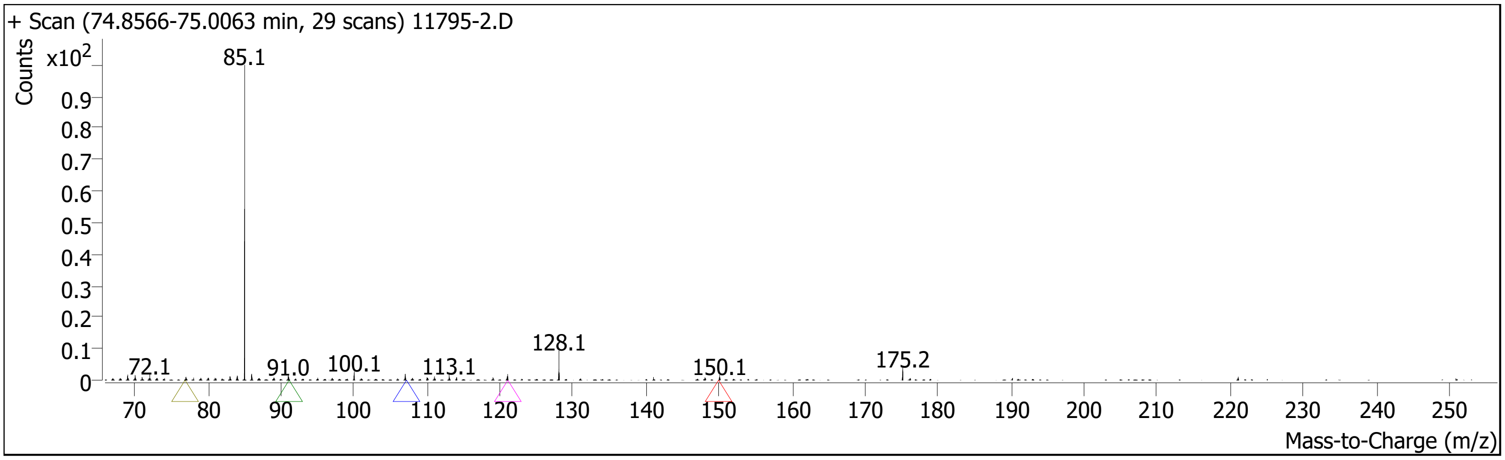

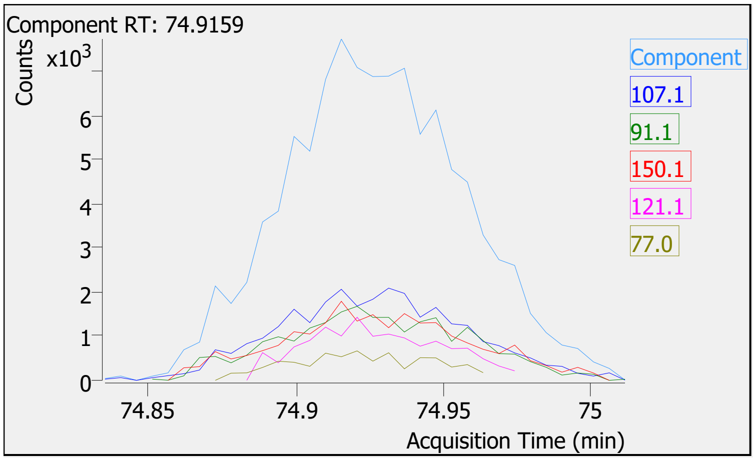

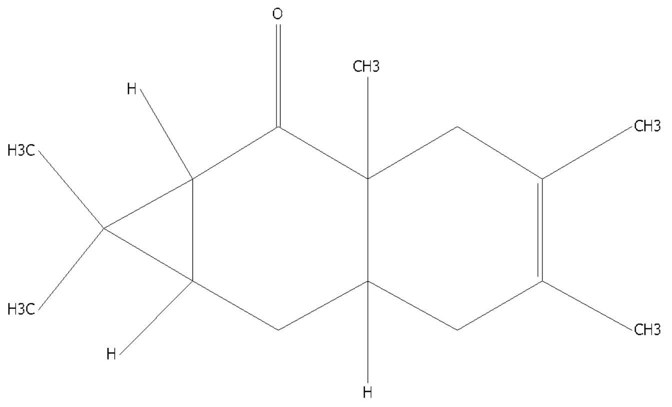

 Generated at 4:32 PM on 11/14/2023

Unknown Analysis Report - Best Hits

RT	Compound Name	CAS#	Formula	Area	MI	Match Score	Sample	Sample
76.8483	2-Propenoic acid 3-[(1E,3Z)-penta-1,3-dienoxy]propyl ester	990066-09-6	C11H16O3	14830		86.1	0.11	0.46

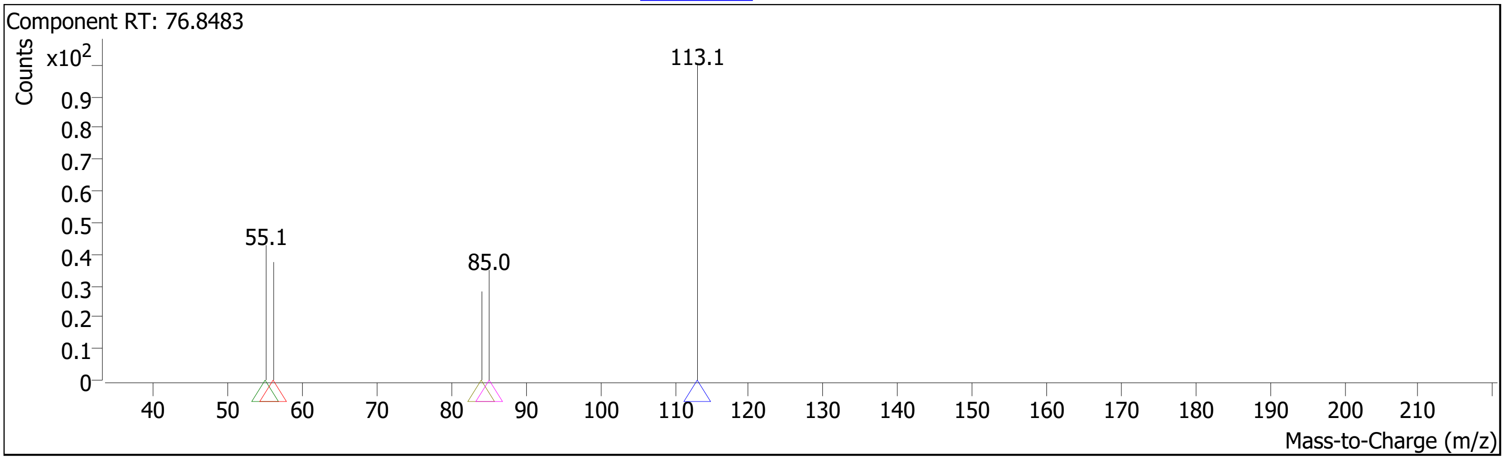

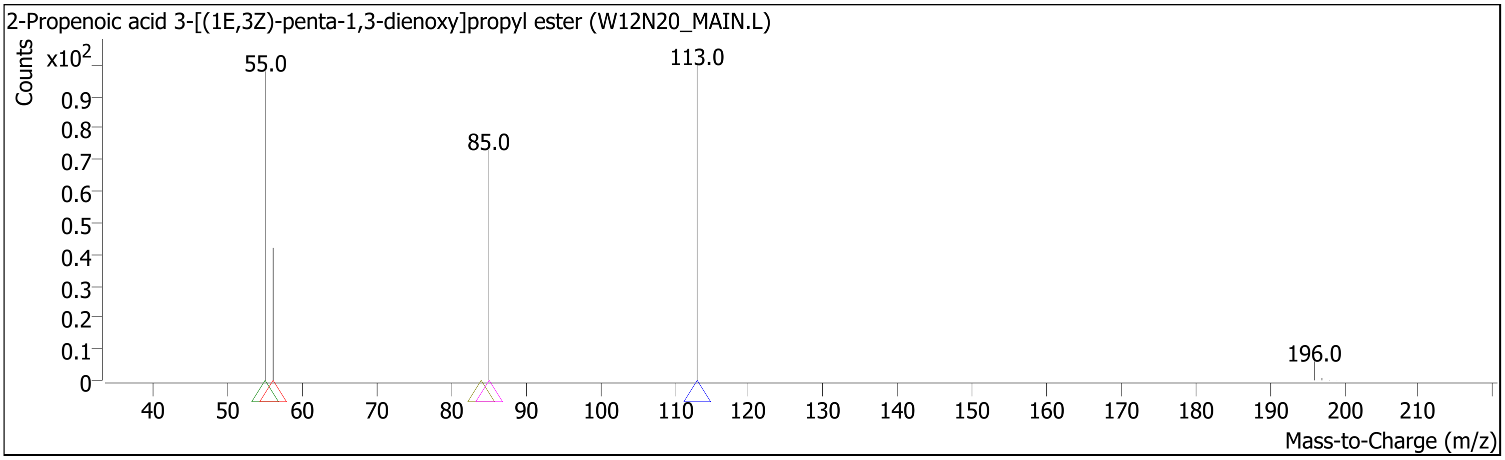

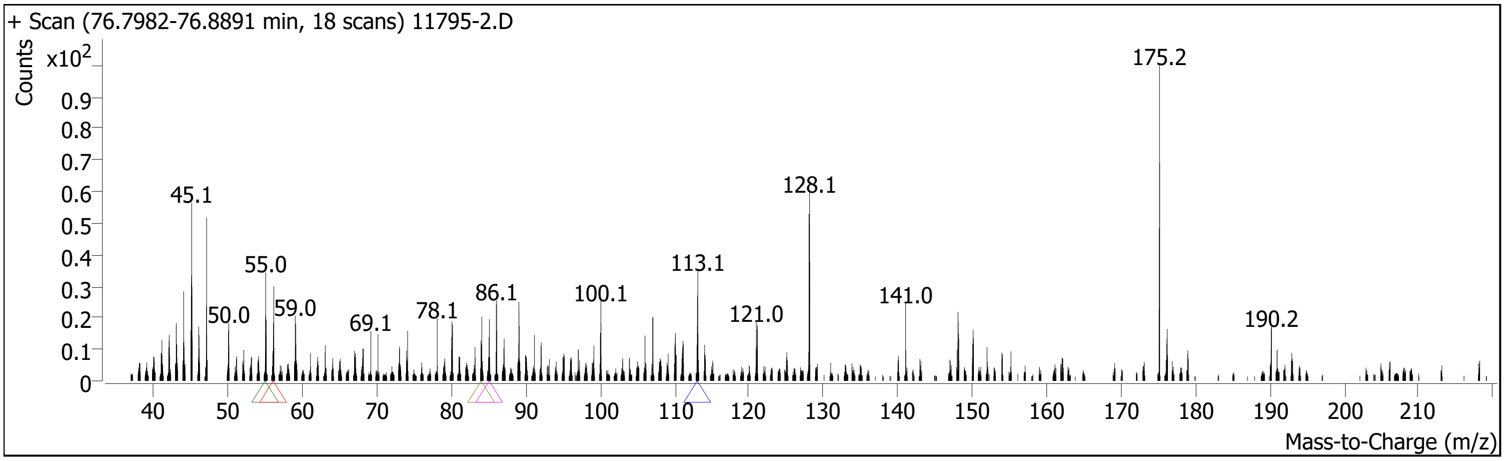

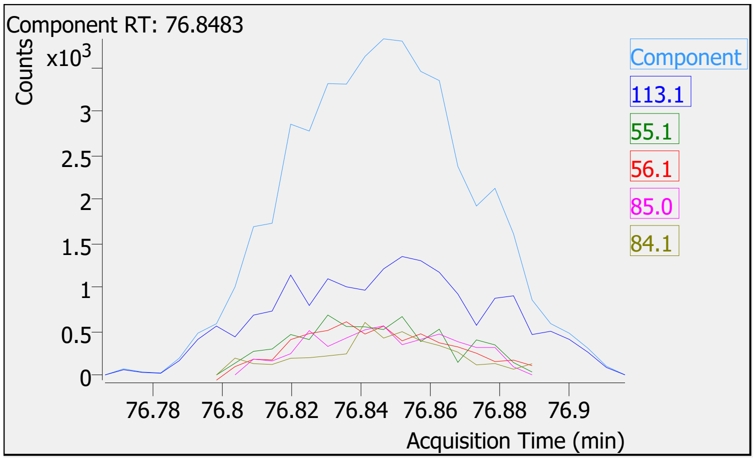

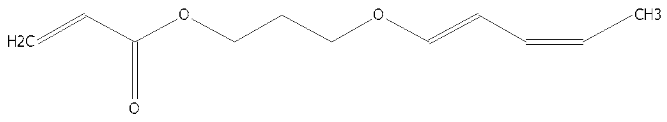

 Generated at 4:32 PM on 11/14/2023

Unknown Analysis Report - Best Hits

RT	Compound Name	CAS#	Formula	Area	MI	Match Score	Sample	Sample
78.9879	Benzyl lactate	990042-49-0	C10H12O3	189653		88.4	1.46	5.85

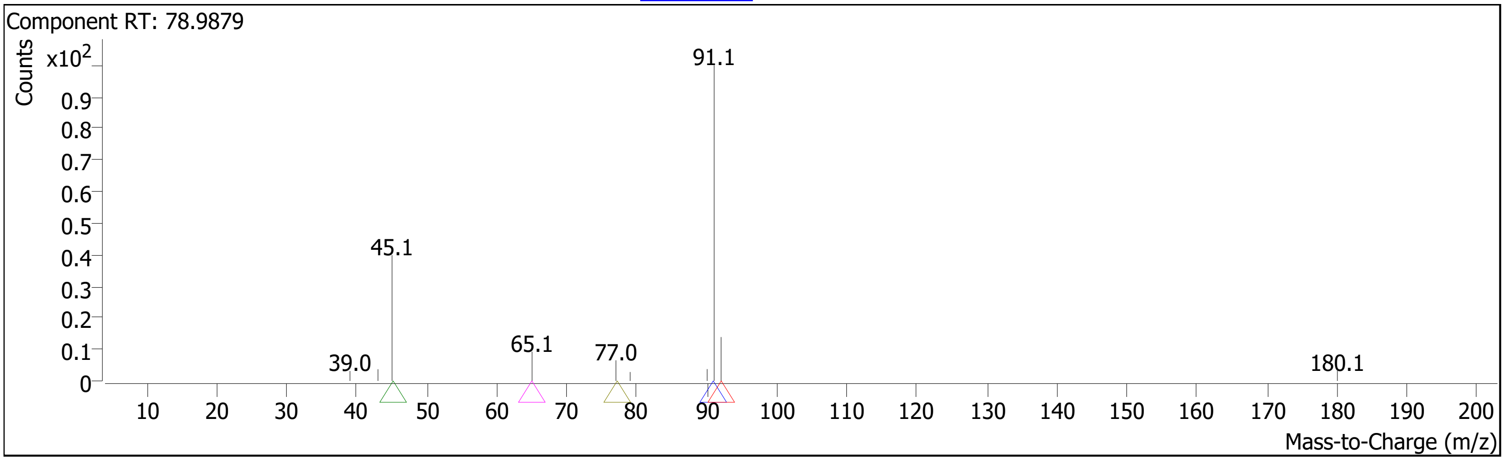

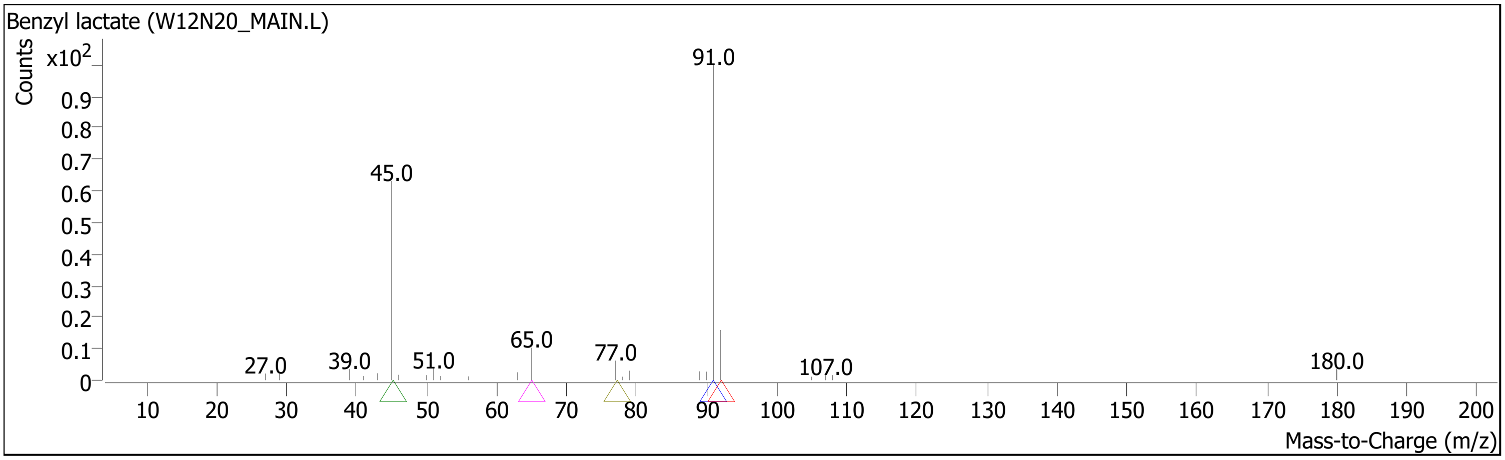

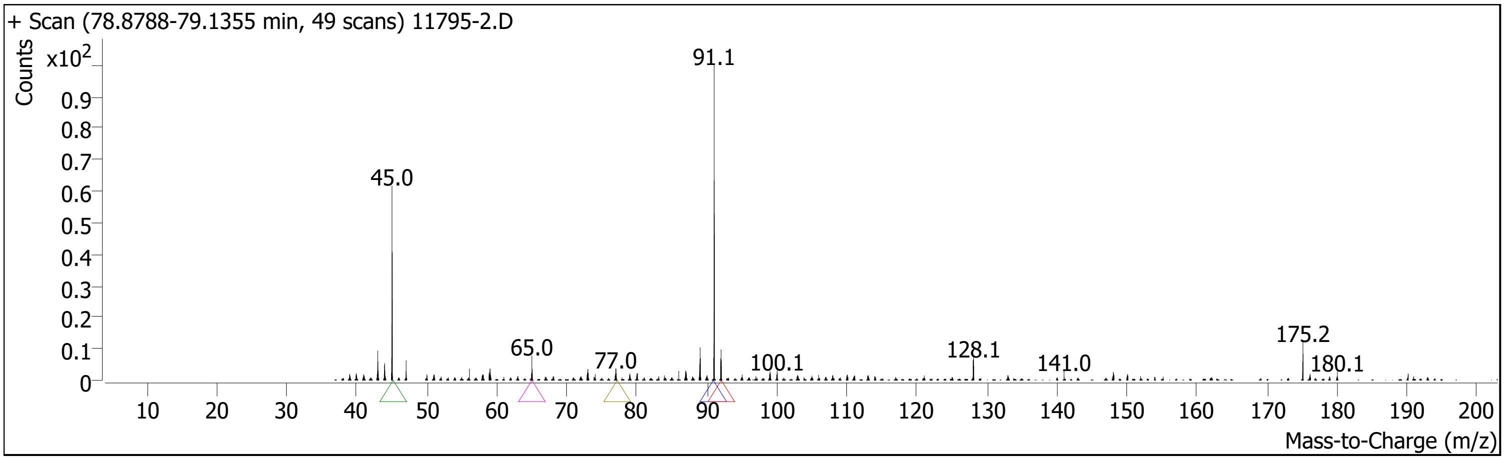

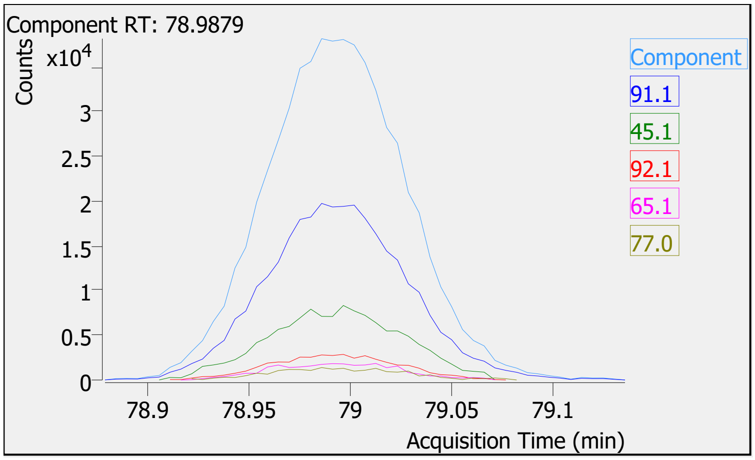

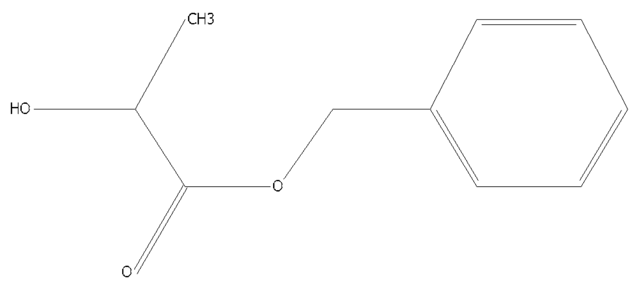

Unknown Analysis Report - Best Hits

RT	Compound Name	CAS#	Formula	Area	MI	Match Score	Sample	Sample
78.9925	Heptane, 1,1,1,2,3,3-hexafluoro-	57915-71-6	C7H10F6	18882		86.9	0.15	0.58

Component RT: 78.9925

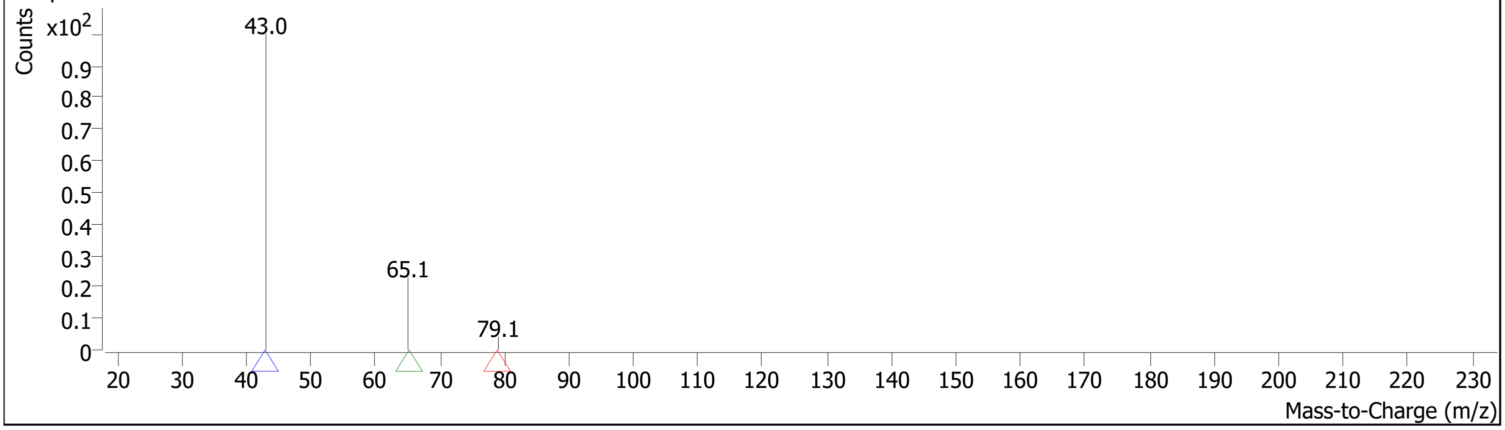

Heptane, 1,1,1,2,3,3-hexafluoro- (W12N20_MAIN.L)

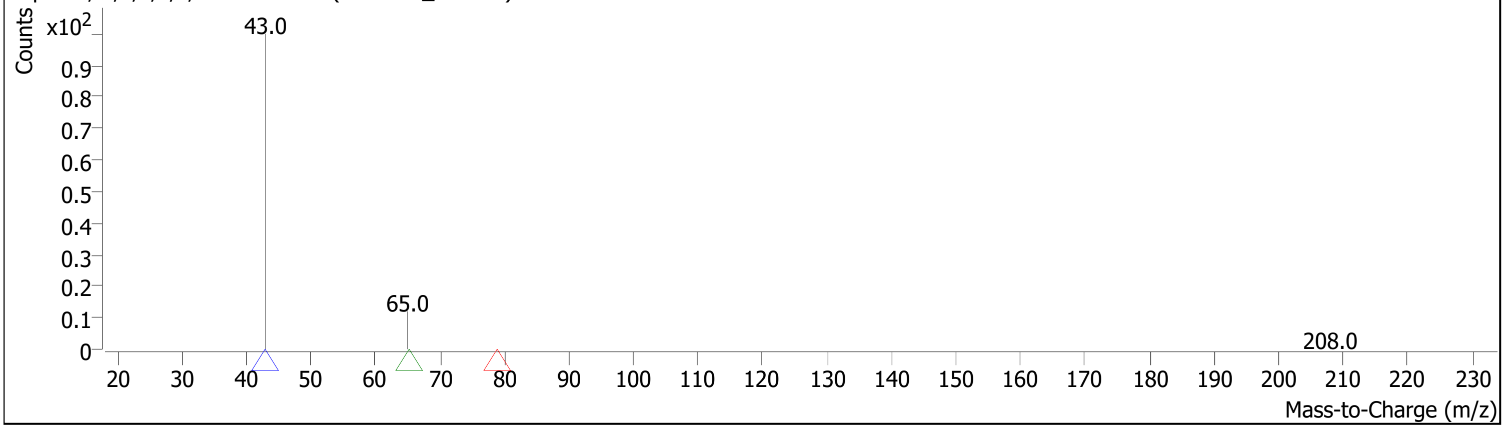

+ Scan (78.9166-79.0714 min, 29 scans) 11795-2.D

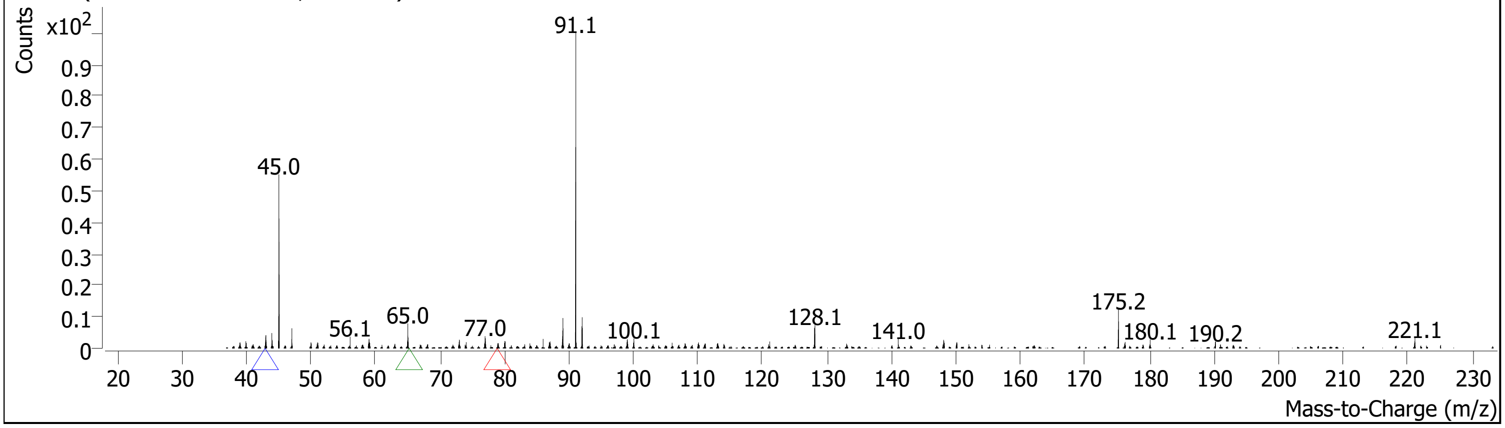

Component RT: 78.9925

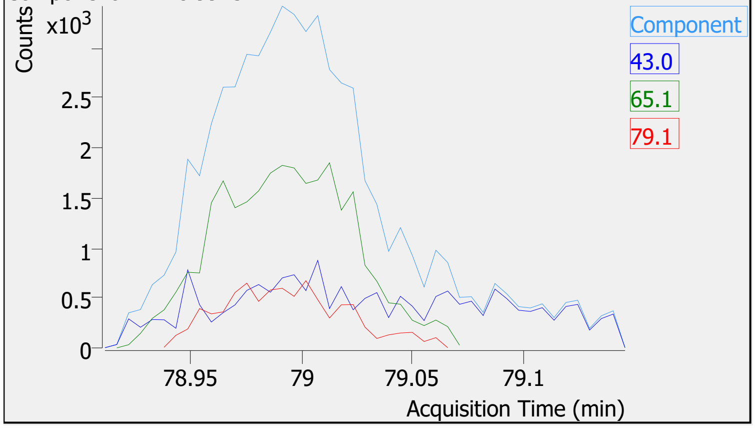

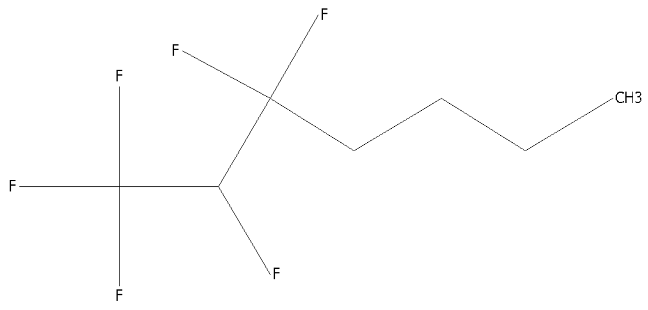

Unknown Analysis Report - Best Hits

RT	Compound Name	CAS#	Formula	Area	MI	Match Score	Sample	Sample
80.1048	2-Methyl-1,3-oxazole-4-carboxylic acid ethyl ester	990017-80-8	C7H9NO3	39466		74.9	0.30	1.22

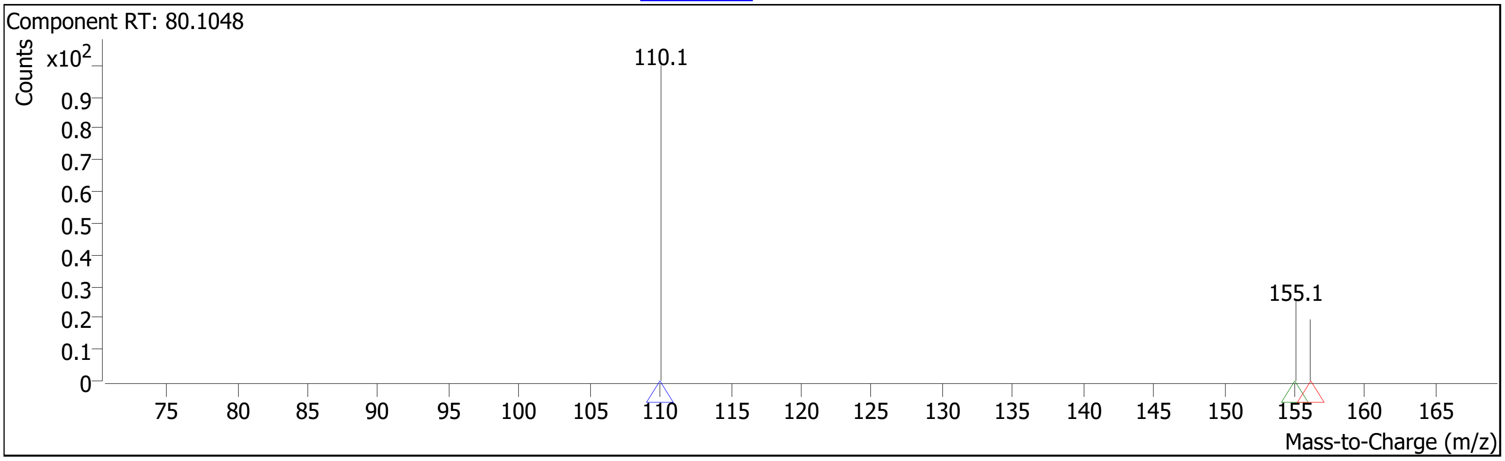

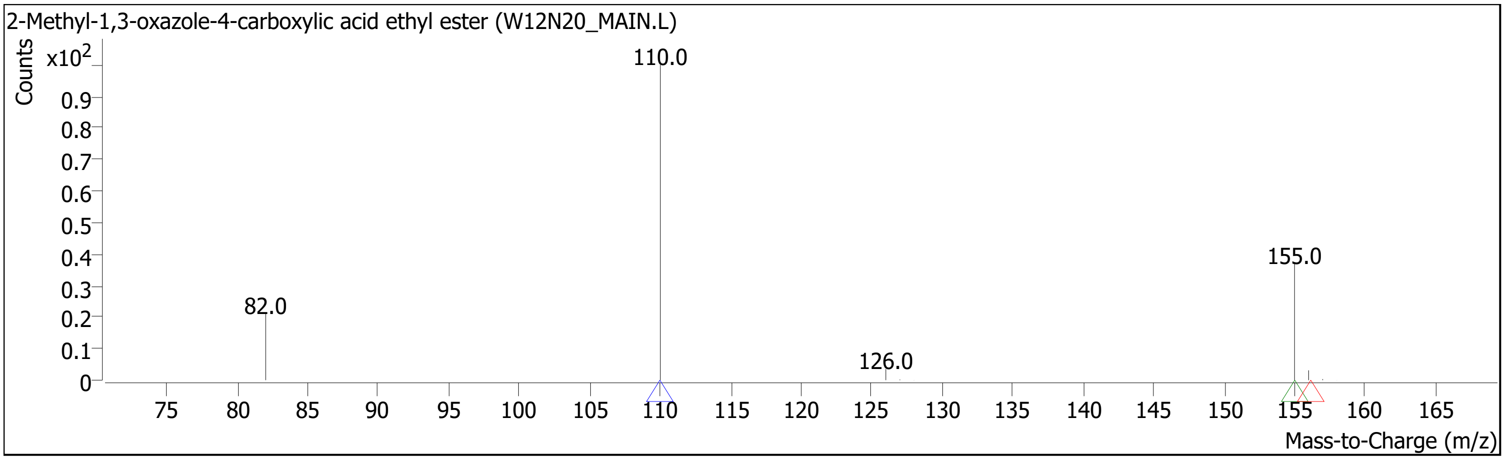

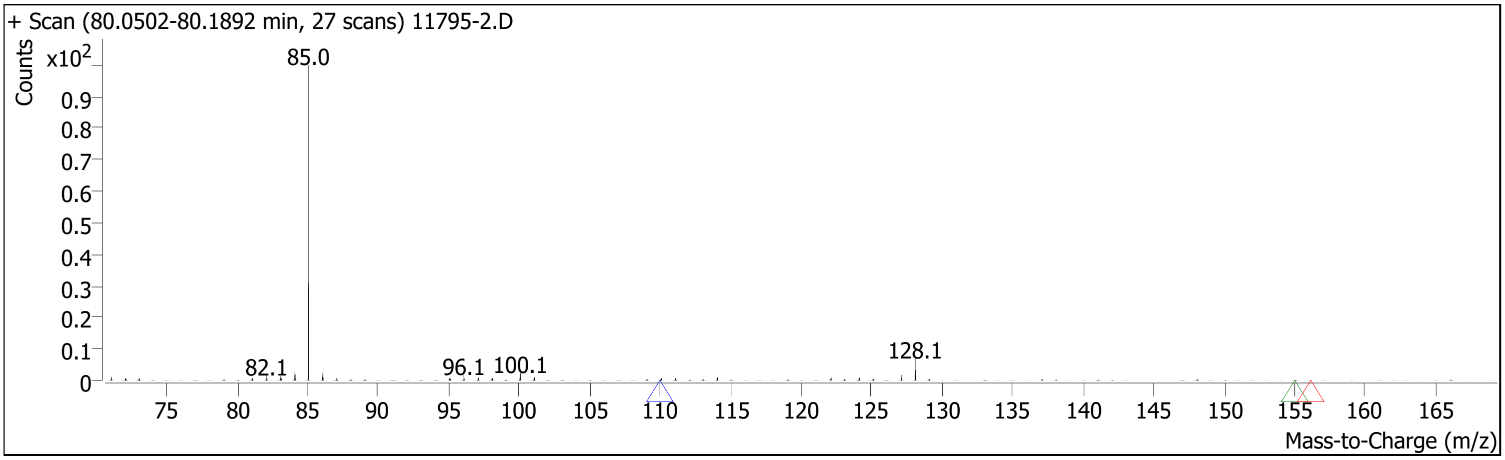

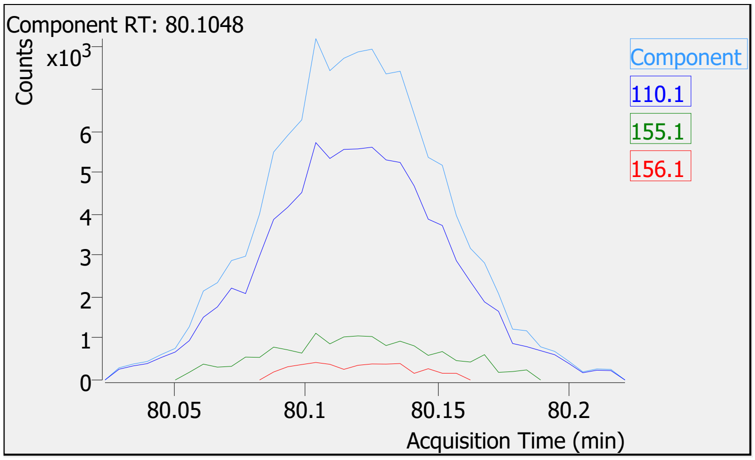

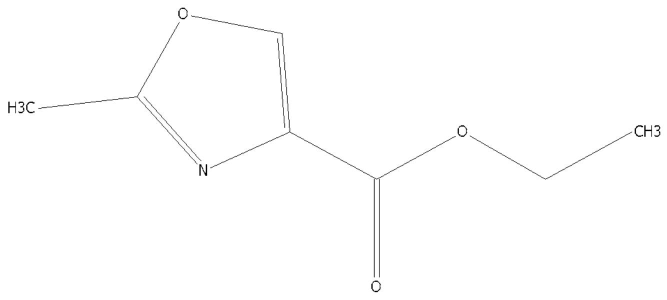

Unknown Analysis Report - Best Hits

RT	Compound Name	CAS#	Formula	Area	MI	Match Score	Sample	Sample
80.1238	2(3H)-Furanone, 5-heptyldihydro-	104-67-6	C11H20O2	3243609		94.5	24.95	100.00

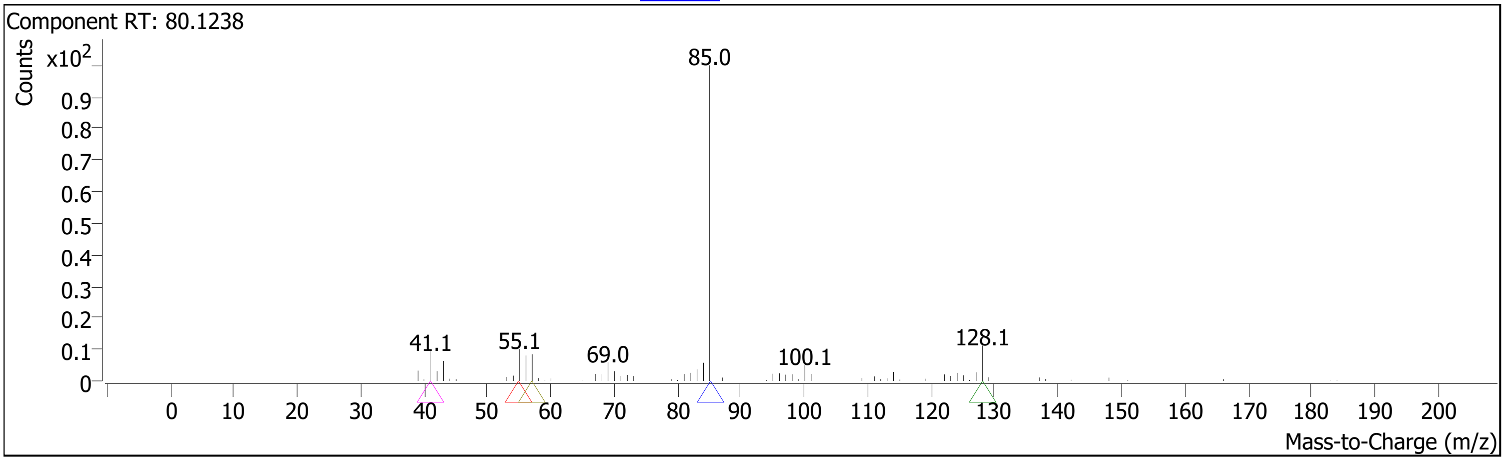

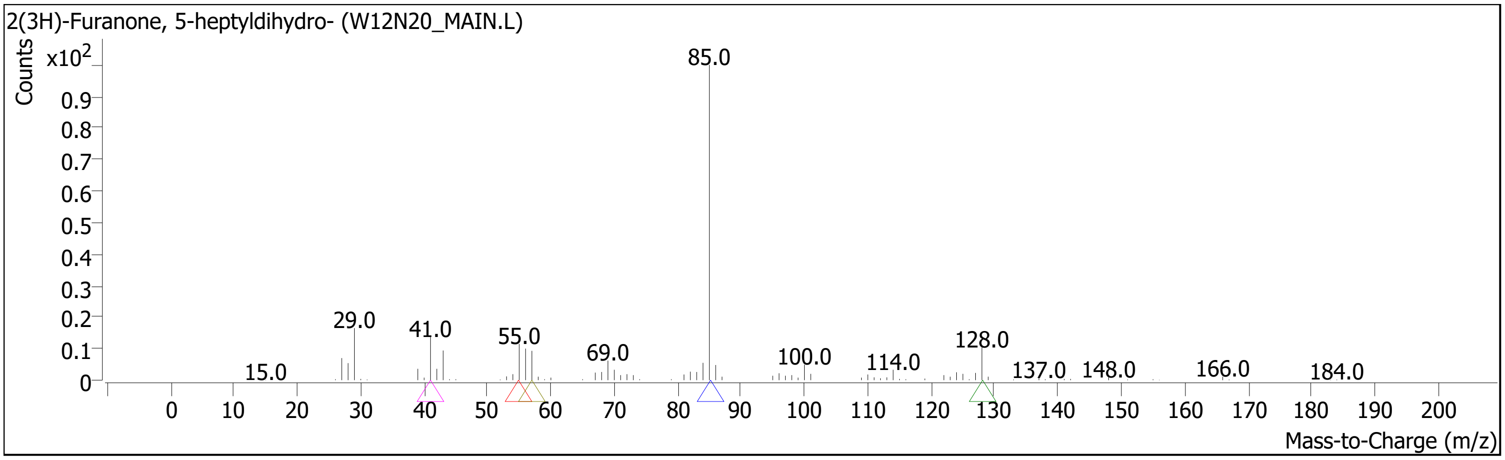

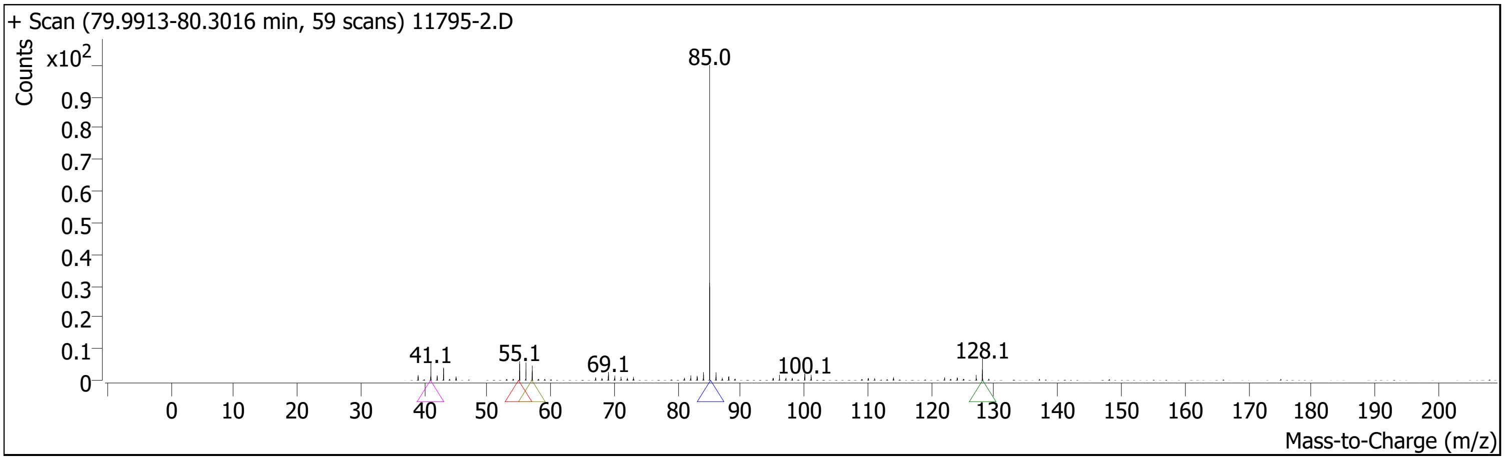

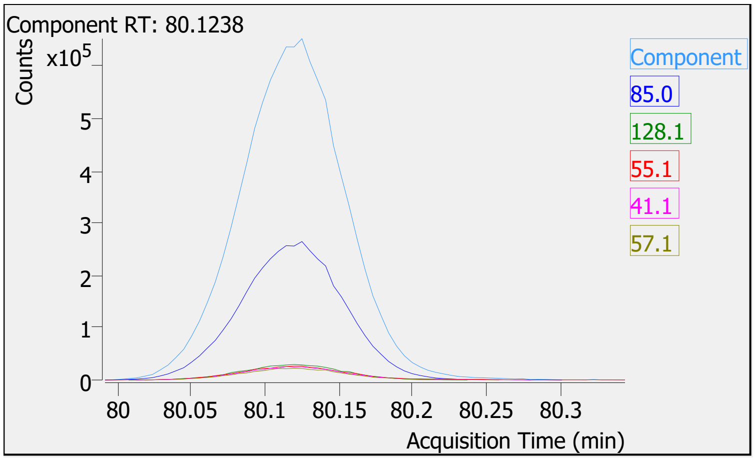

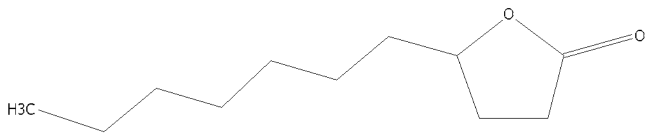

RT	Compound Name	CAS#	Formula	Area	MI	Match Score	Sample	Sample
80.1241	(E)-1-(2-Tetrahydropyranyloxy)-7-decene	990155-85-8	C15H28O2	1713054		76.1	13.18	52.81

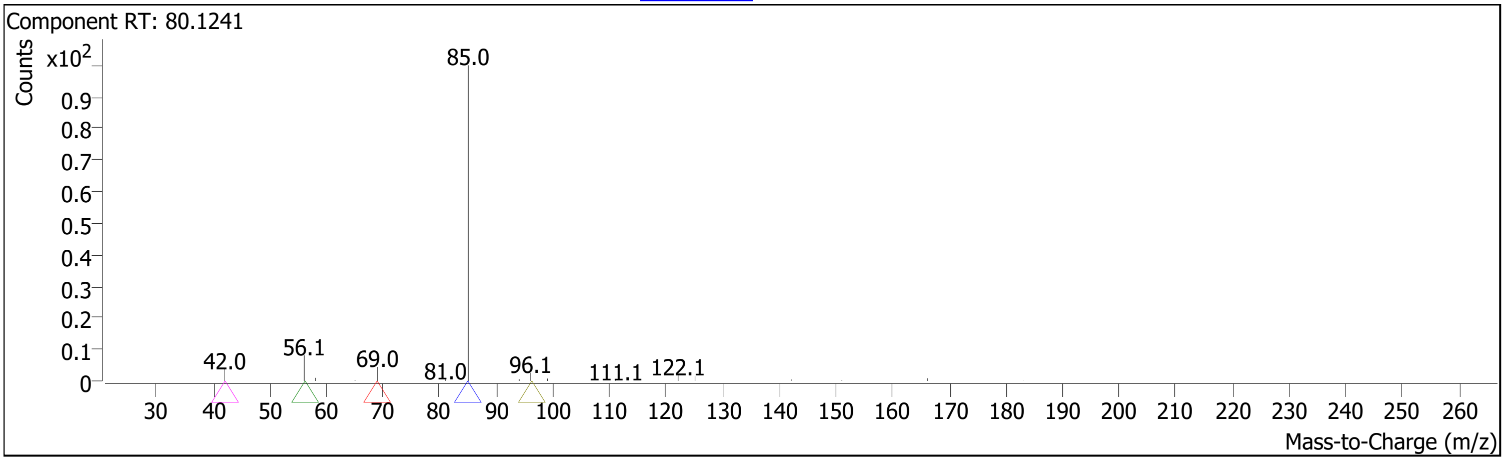

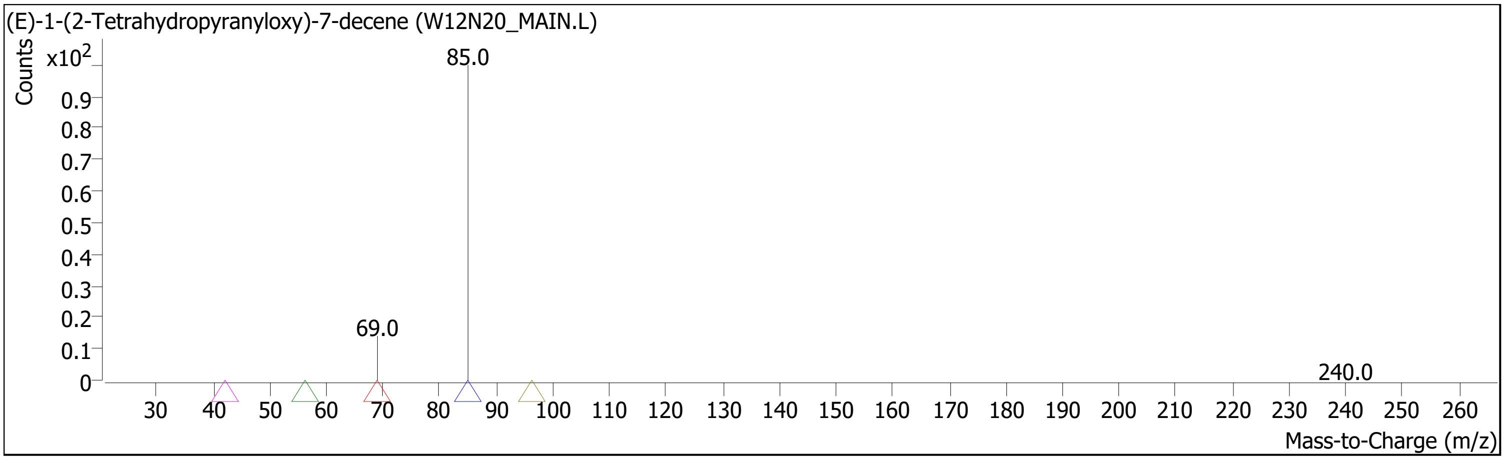

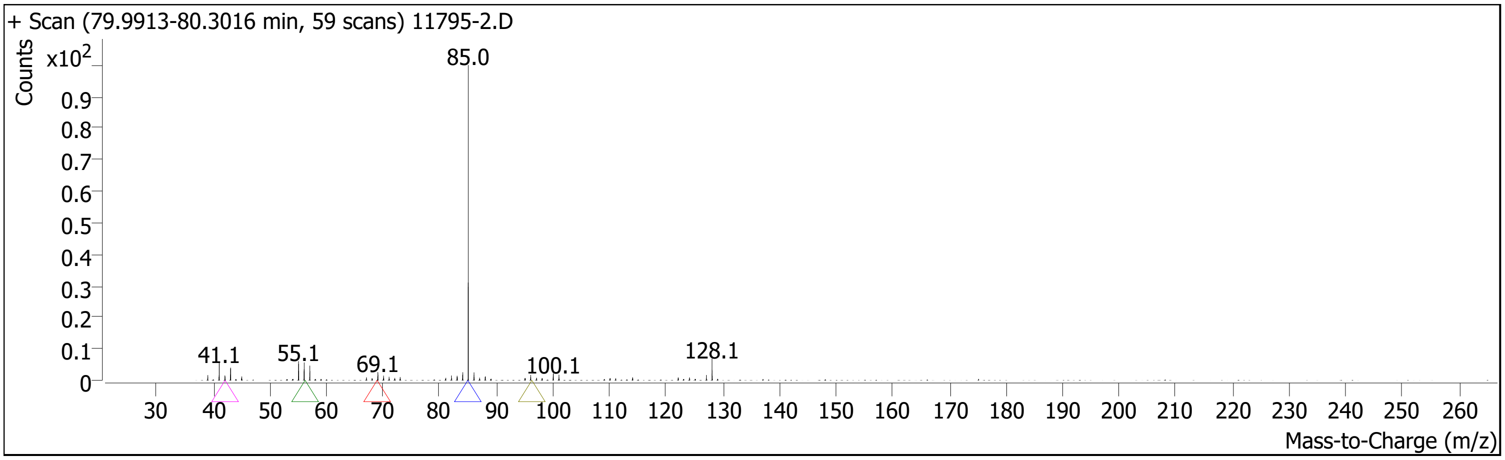

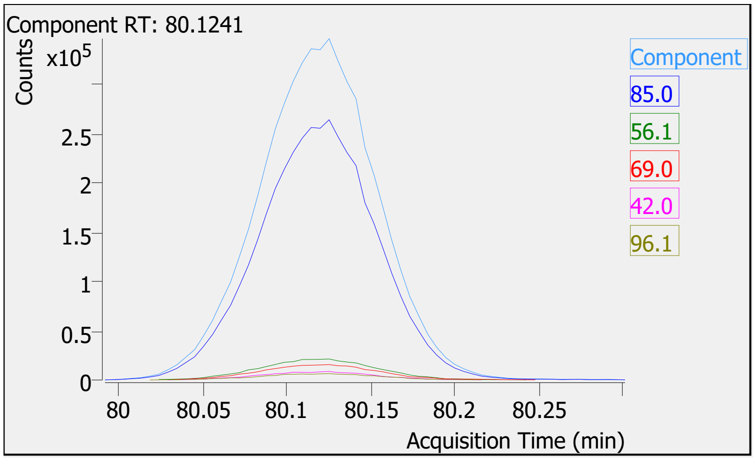

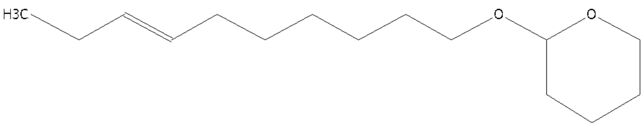

RT	Compound Name	CAS#	Formula	Area	MI	Match Score	Sample	Sample
80.1345	1-(Bromoethynyl)cyclohexan-1-on	0-00-0	C8H11BrO	64737		86.6	0.50	2.00

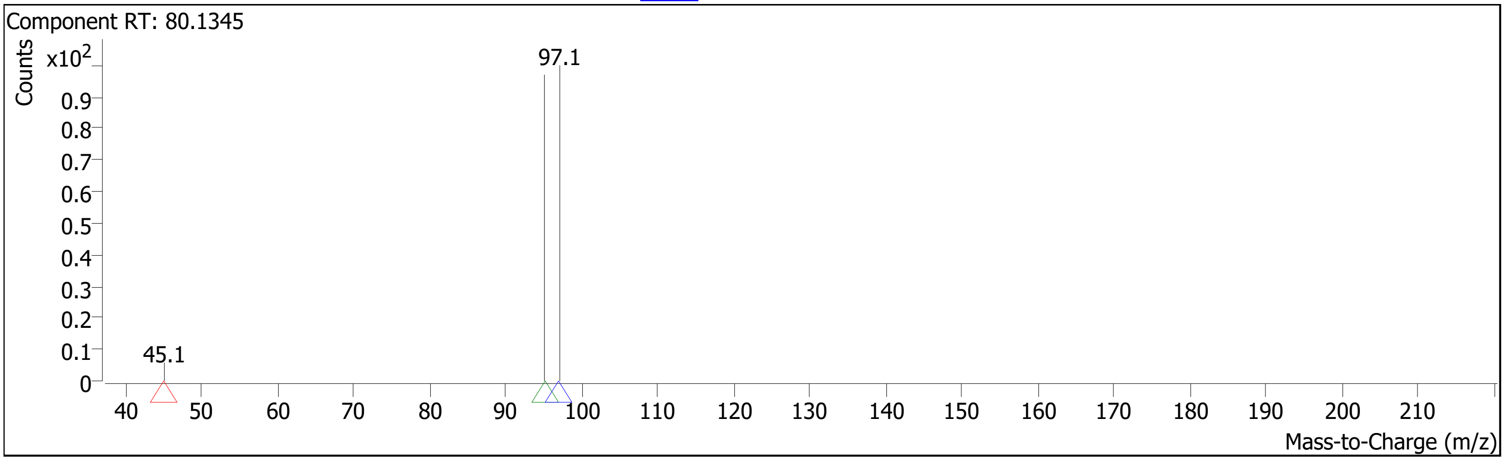

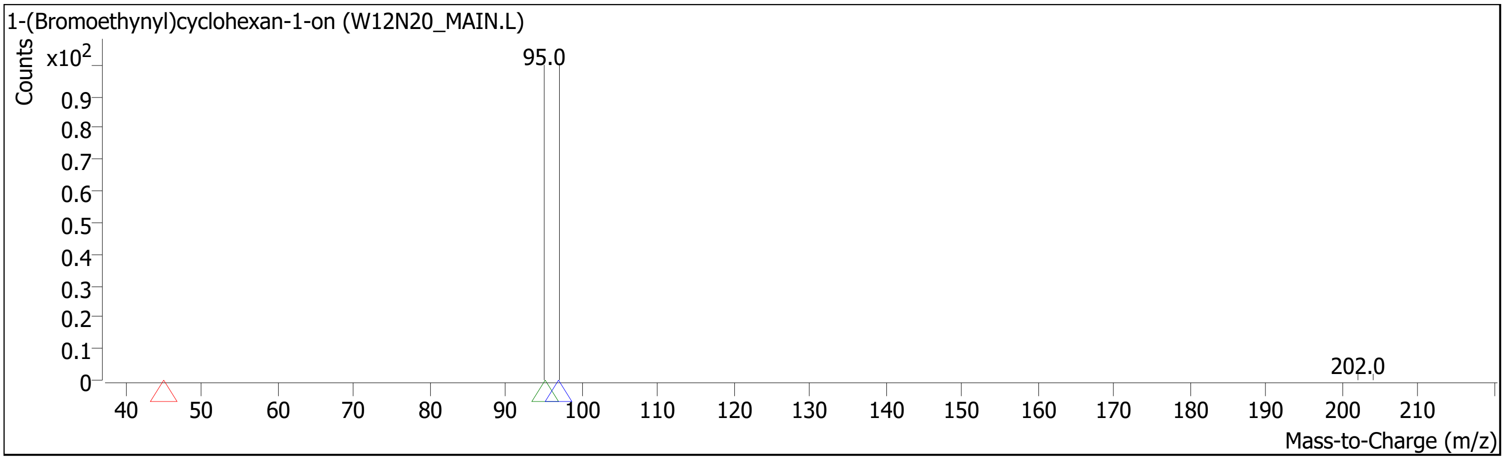

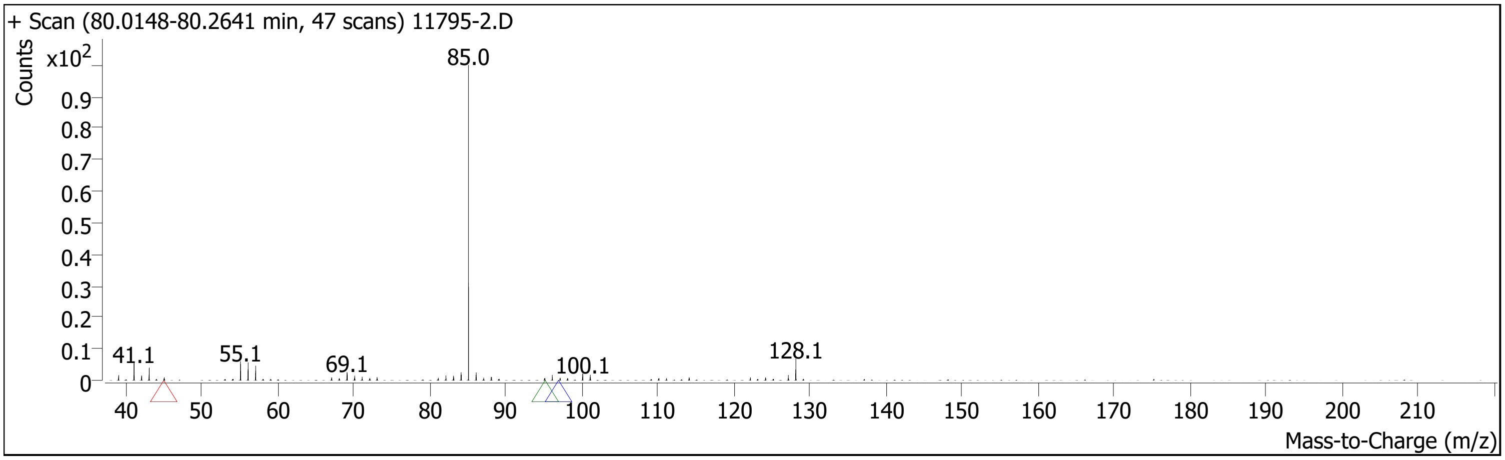

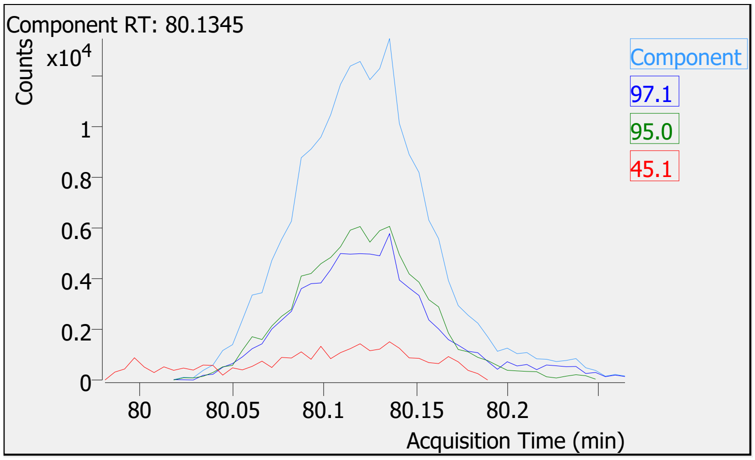

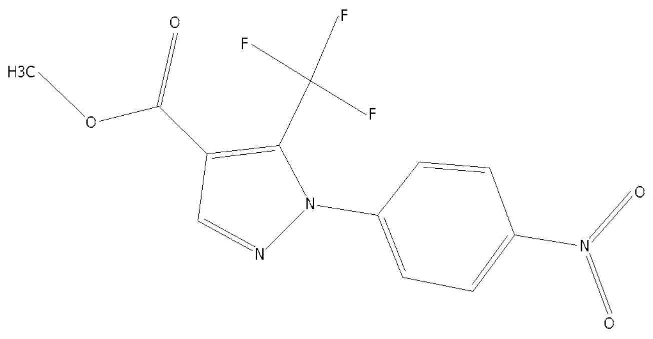

Unknown Analysis Report - Best Hits

RT	Compound Name	CAS#	Formula	Area	MI	Match Score	Sample	Sample
82.5335	Pyrimidinium, 5-carboxy-4-(1,1-dimethylethyl)-1,6-dimethyl-2-(1-methylethyl)-, hydroxide, inner salt	108169-08-0	C14H22N2O2	110617		82.9	0.85	3.41

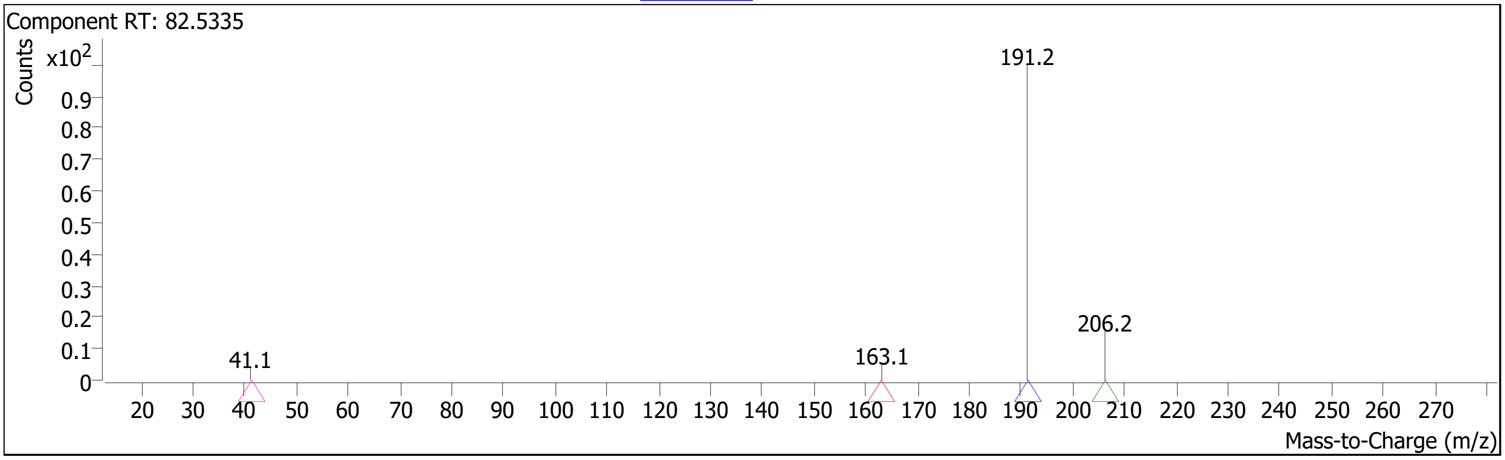

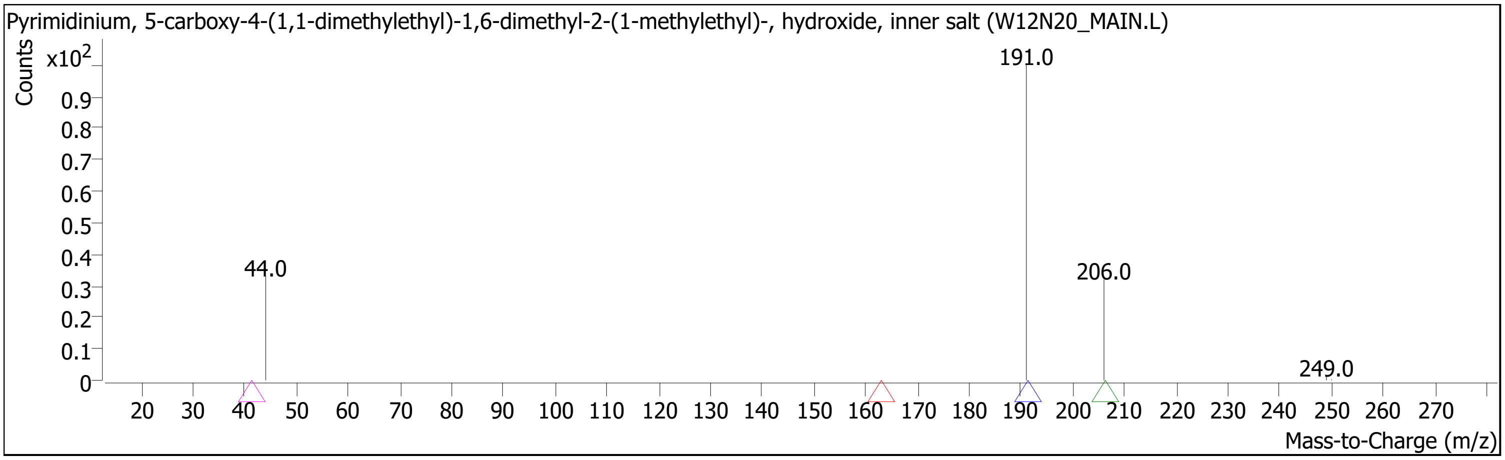

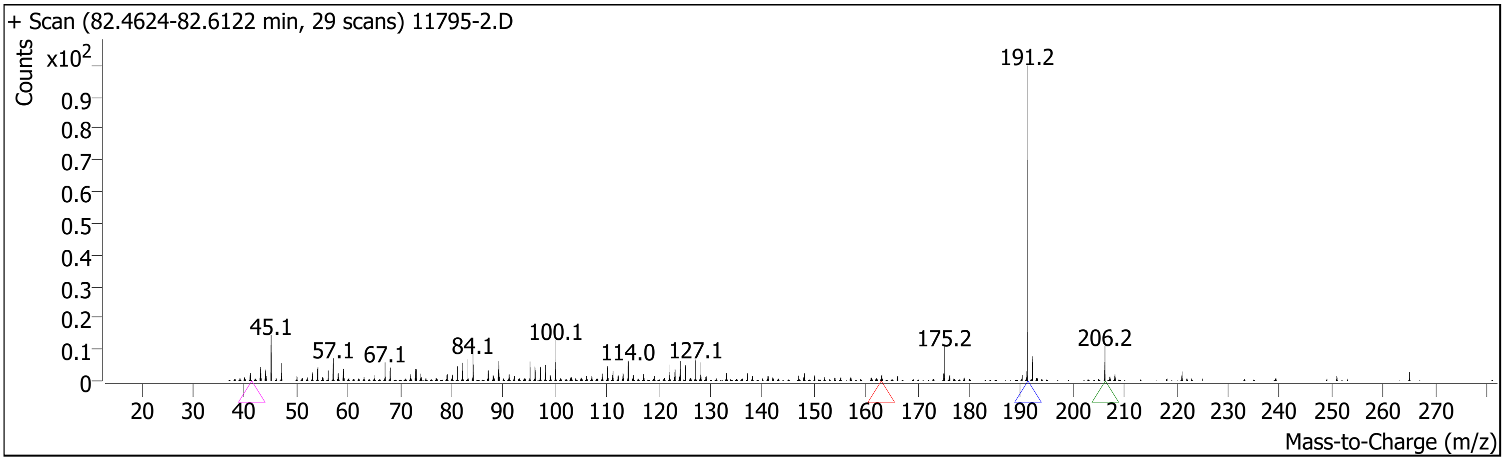

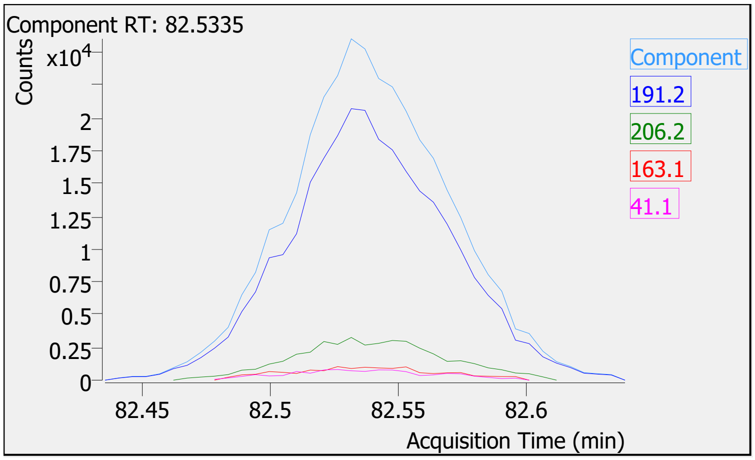

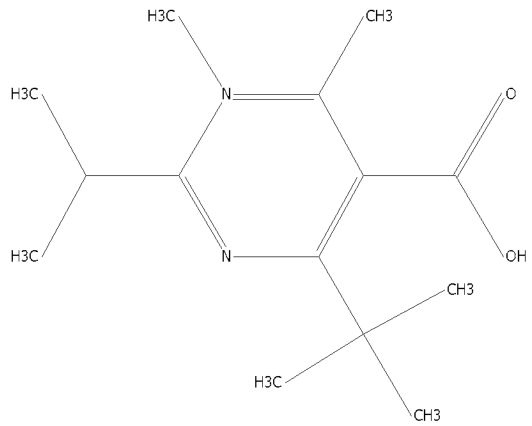

 Generated at 4:32 PM on 11/14/2023

RT	Compound Name	CAS#	Formula	Area	MI	Match Score	Sample	Sample
83.7397	Benzene, 1,2,3-trimethoxy-5-(1-propenyl)-, (E)-	5273-85-8	C12H16O3	2447567		91.6	18.83	75.46

Component RT: 83.7397

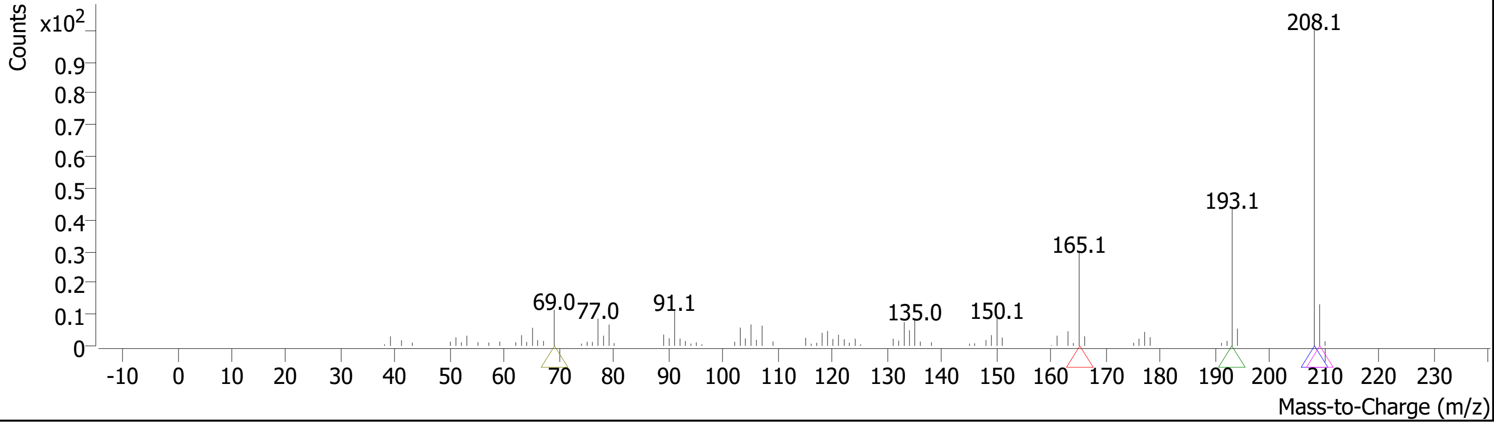

Benzene, 1,2,3-trimethoxy-5-(1-propenyl)-, (E)- (W12N20_MAIN.L)

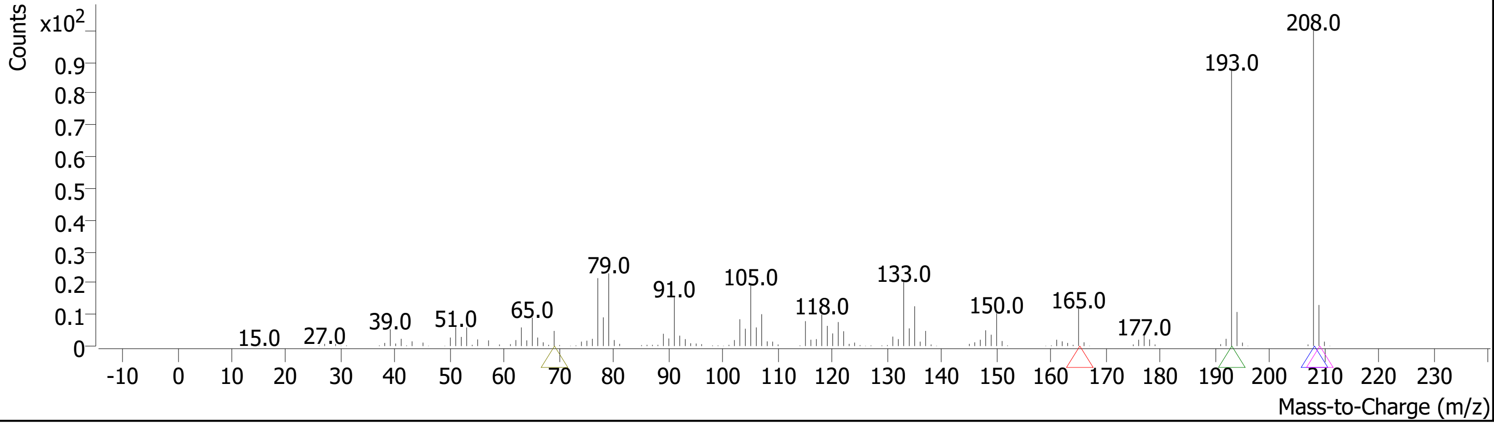

+ Scan (83.6242-83.8619 min, 44 scans) 11795-2.D

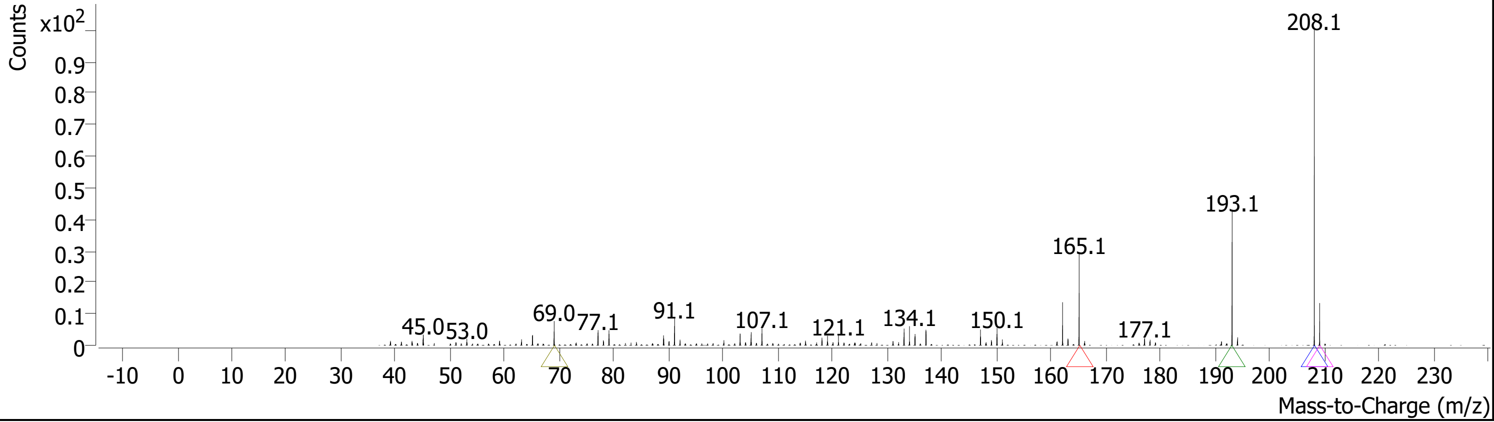

Component RT: 83.7397

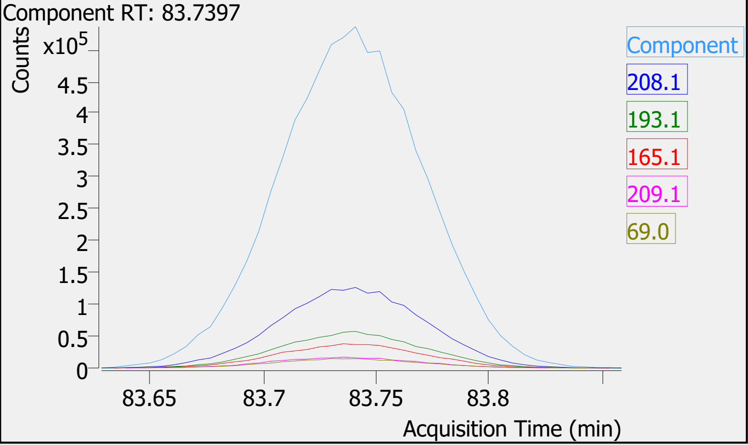

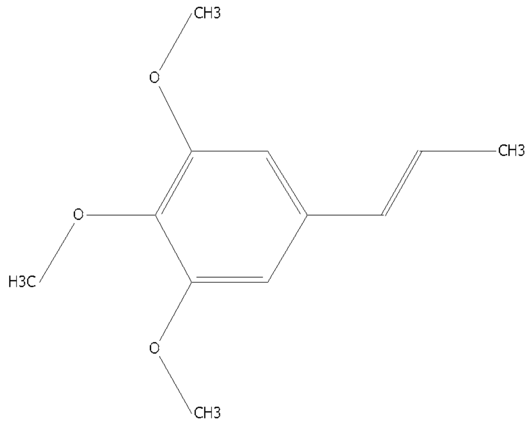

RT	Compound Name	CAS#	Formula	Area	MI	Match Score	Sample	Sample
88.6036	tert-Butyl (2S*,7aR*)-2-(2-methoxy-2-oxoethyl)-2-[(methylsulfonyl)oxy]-3-oxotetrahydr-1H-pyrrolizine-7a(5H)-carboxylate	0-00-0	C16H25NO8	18494		84.1	0.14	0.57

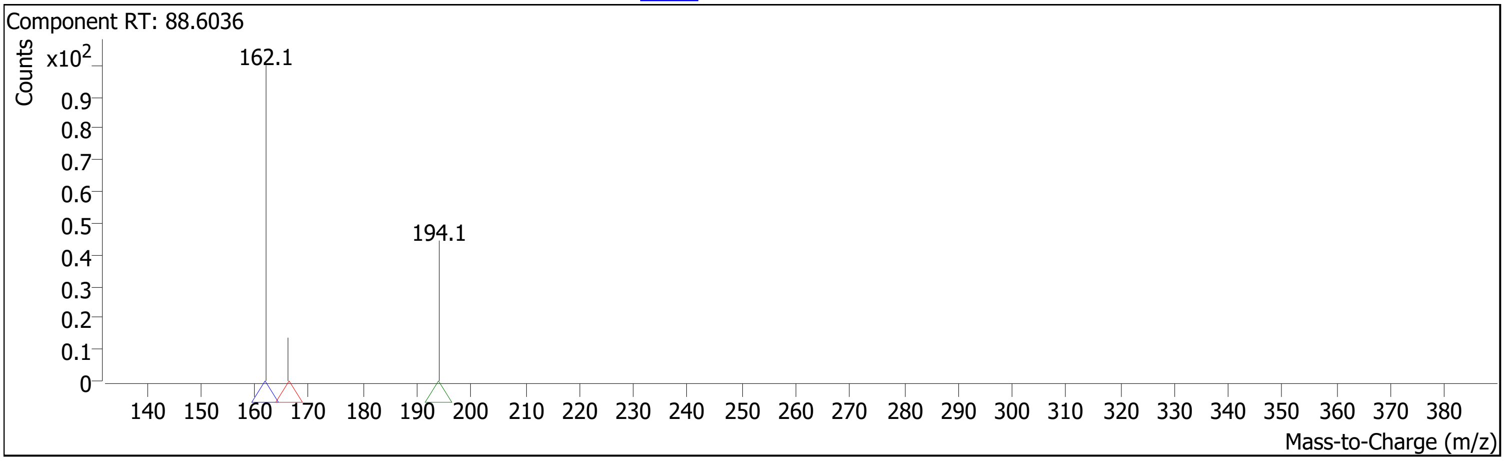

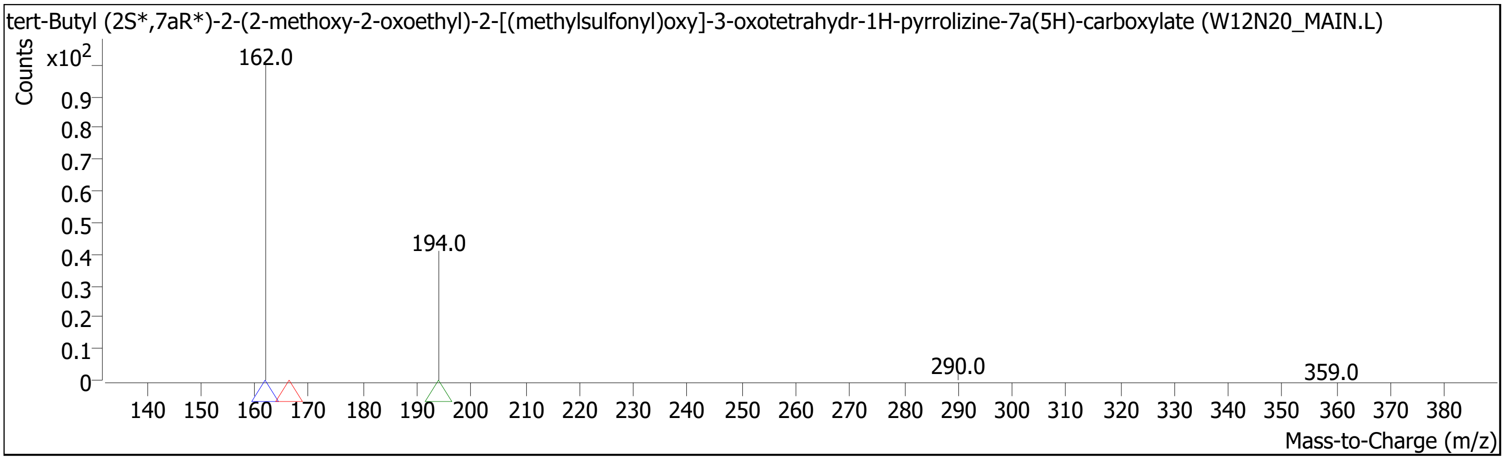

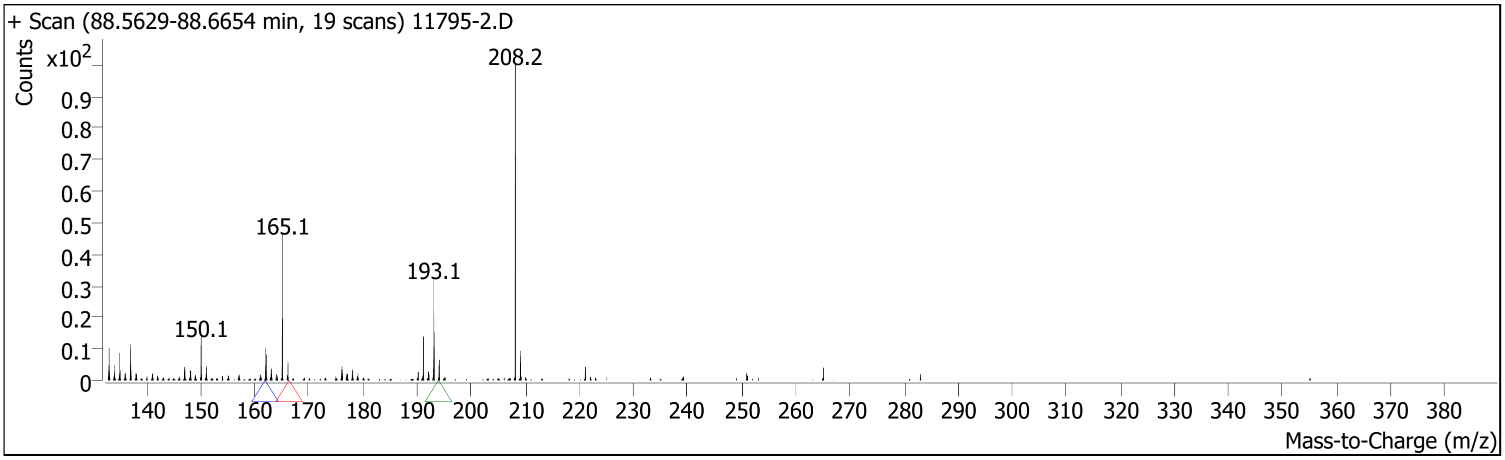

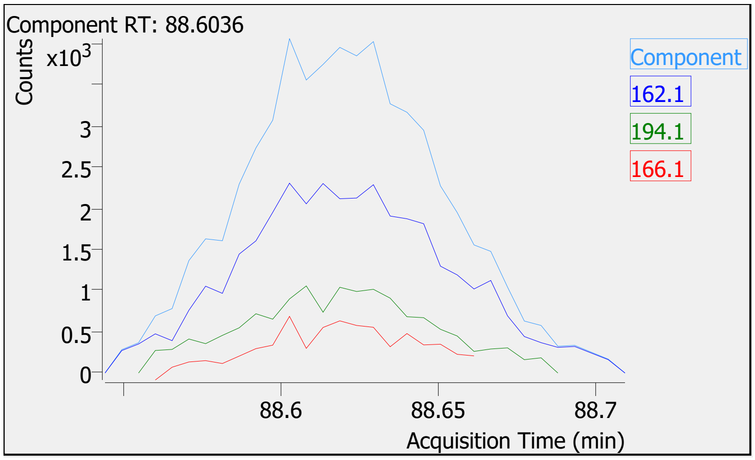

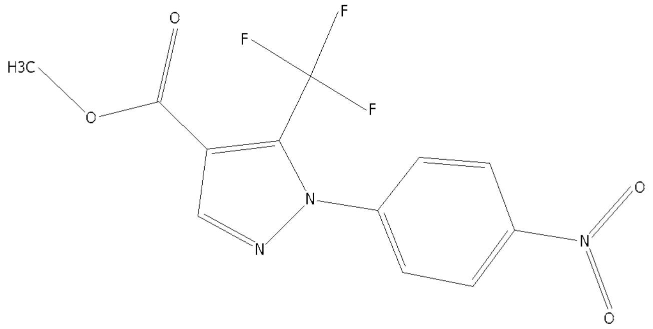

 Generated at 4:32 PM on 11/14/2023

Unknown Analysis Report - Best Hits

Agilent Trusted Answers

RT	Compound Name	CAS#	Formula	Area	MI	Match Score	Sample	Sample
88.6187	.beta.-Asarone	5273-86-9	C12H16O3	294602		84.0	2.27	9.08

Component RT: 88.6187

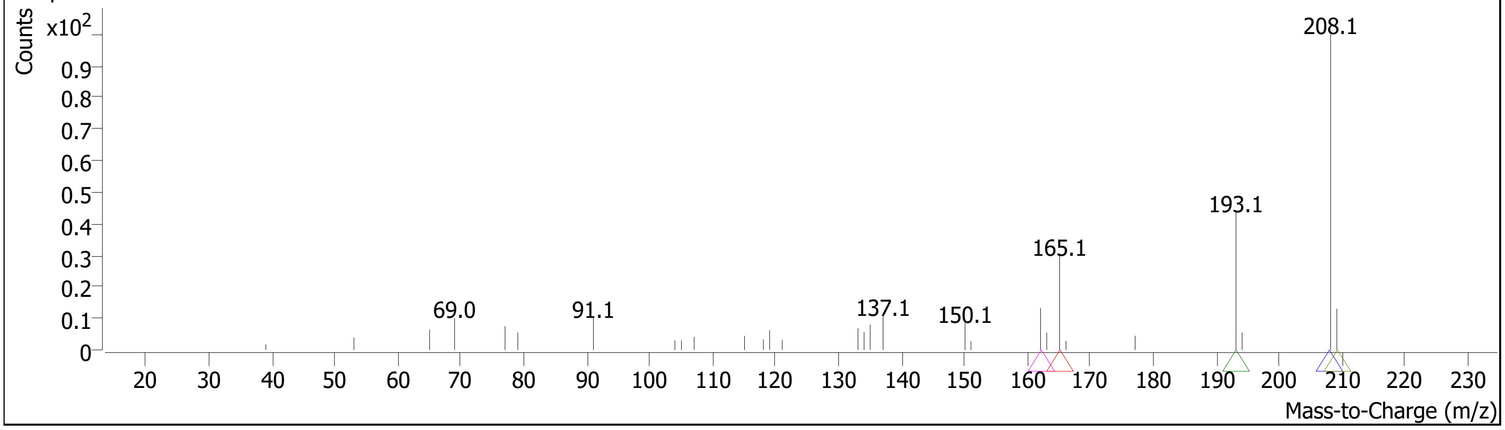

.beta.-Asarone (W12N20_MAIN.L)

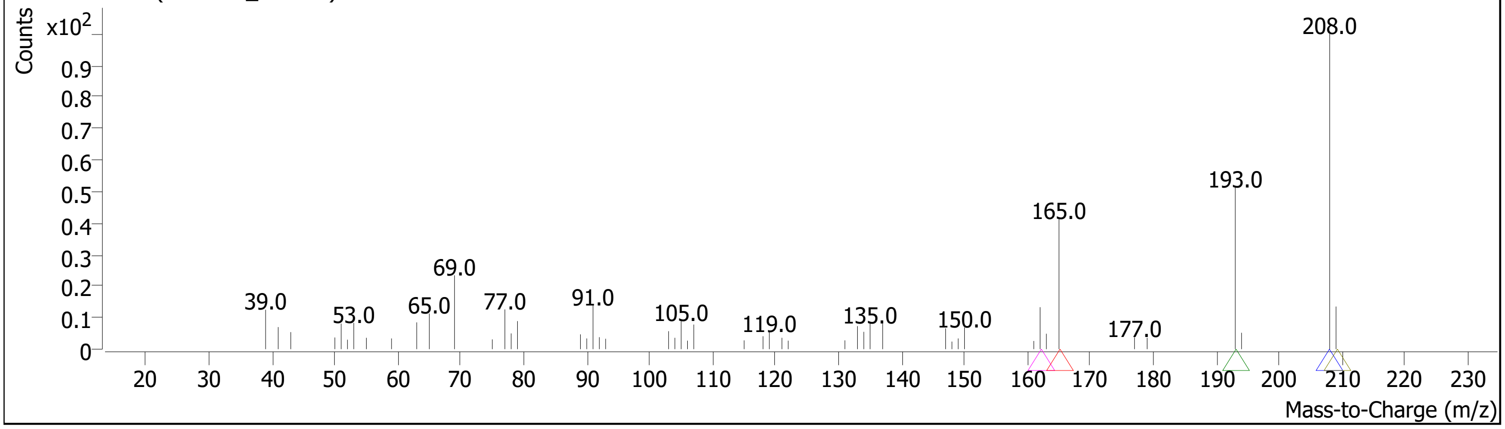

+ Scan (88.5175-88.7200 min, 37 scans) 11795-2.D

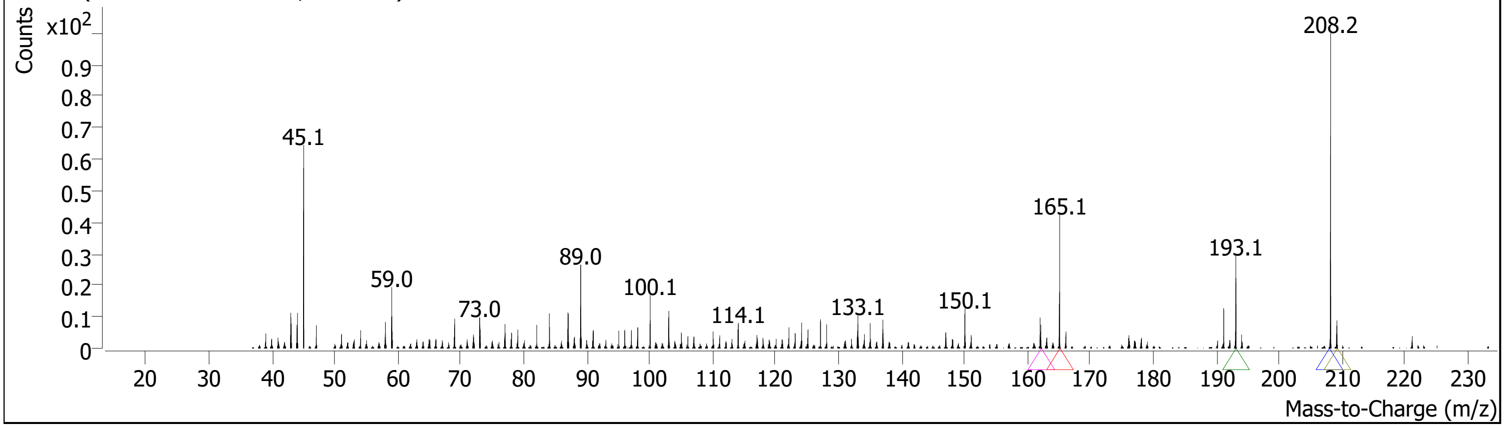

Component RT: 88.6187

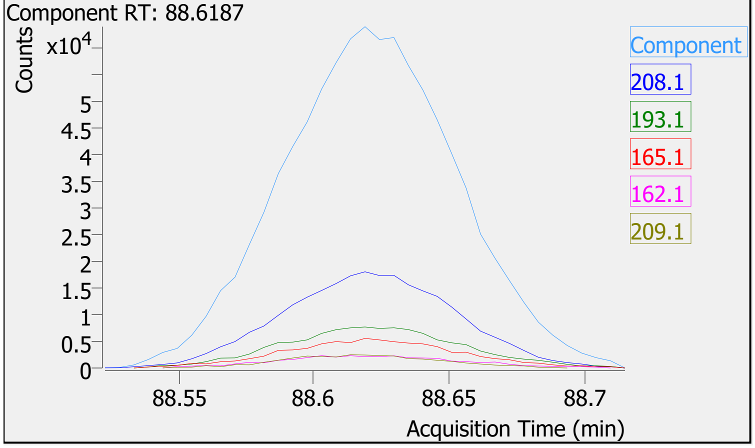

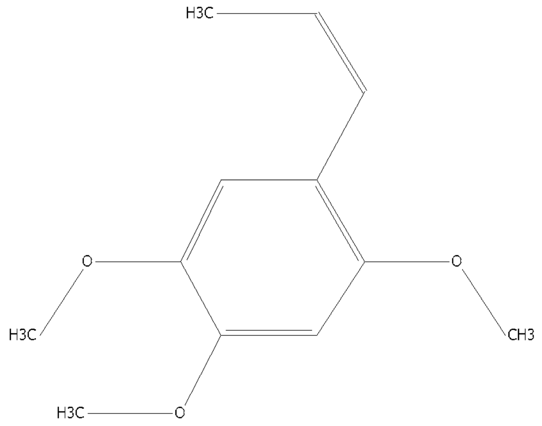

 Generated at 4:32 PM on 11/14/2023

Unknown Analysis Report - Best Hits

RT	Compound Name	CAS#	Formula	Area	MI	Match Score	Sample	Sample
92.6781	(E)-phenyl 3-cyclohexyl-2-methylacrylate	990165-06-3	C16H20O2	80979		77.7	0.62	2.50

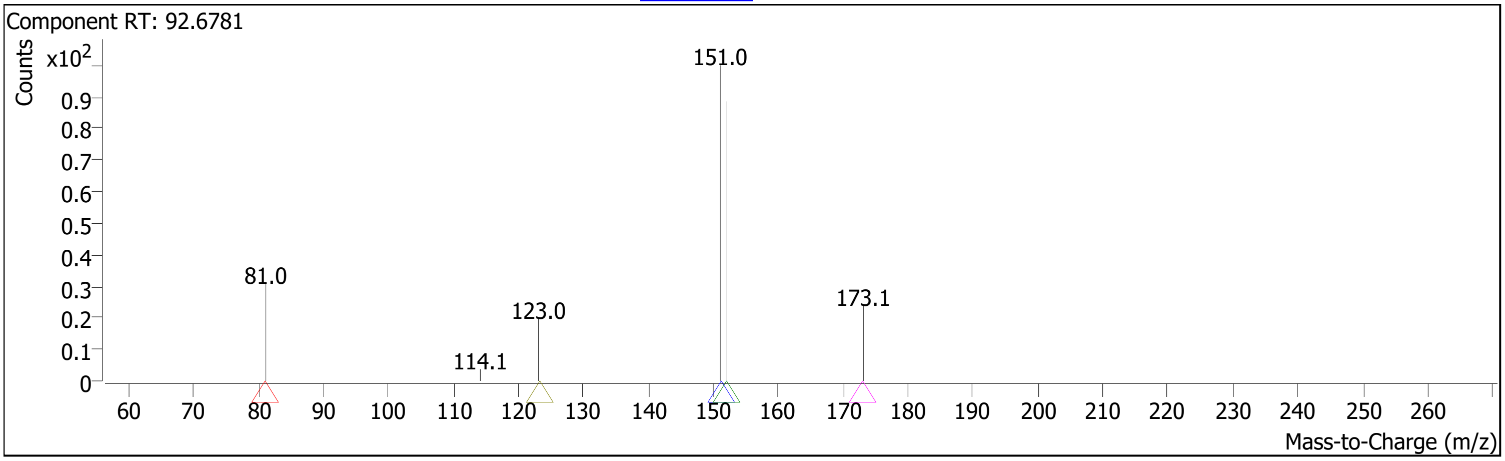

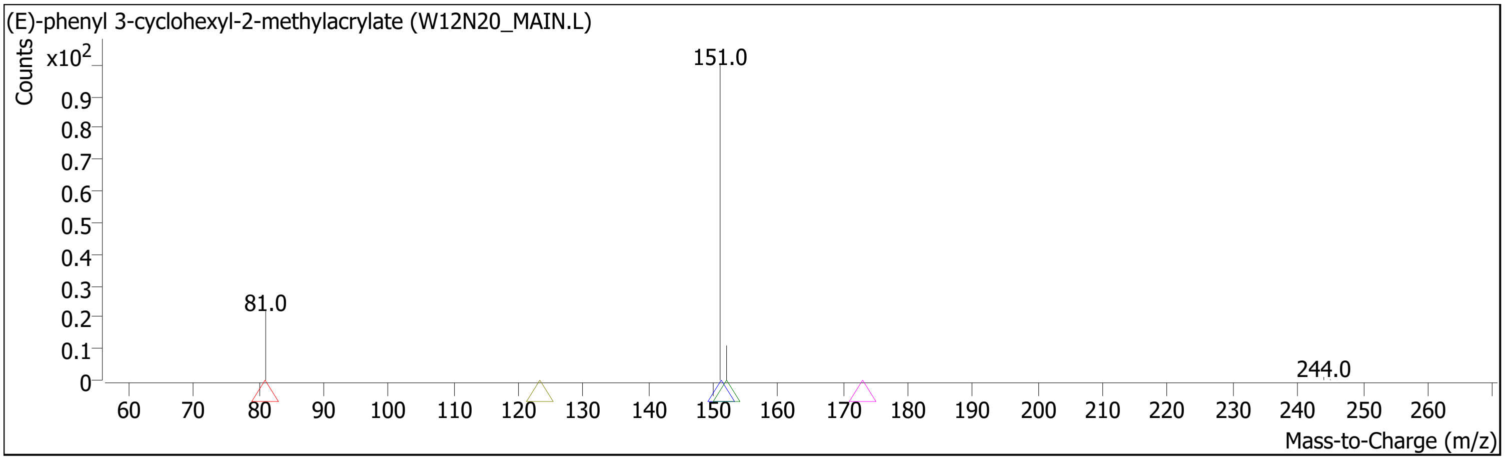

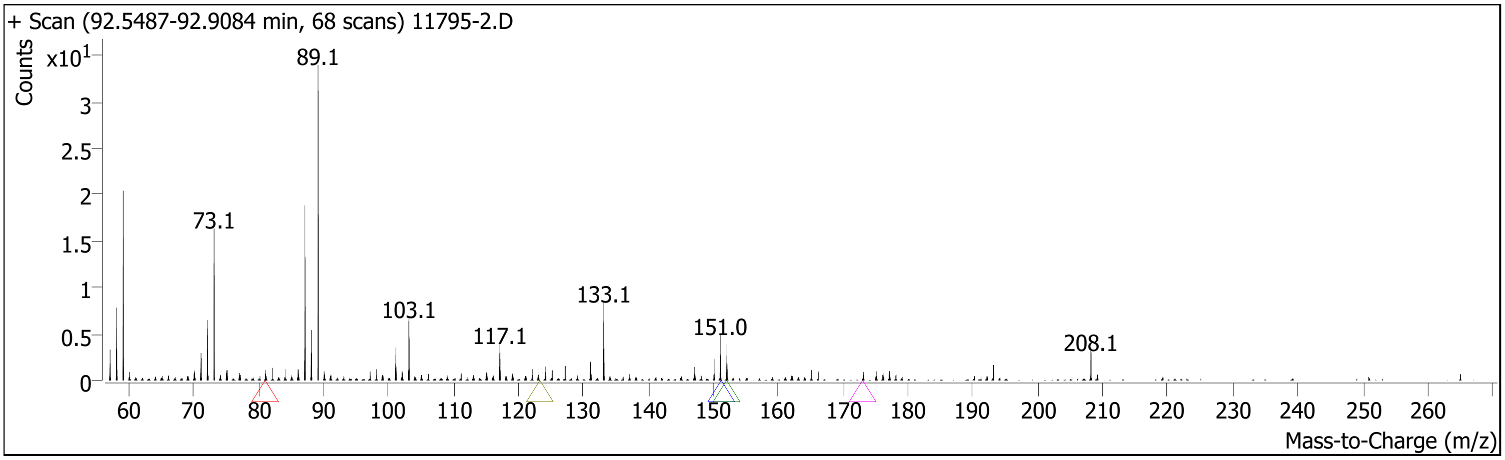

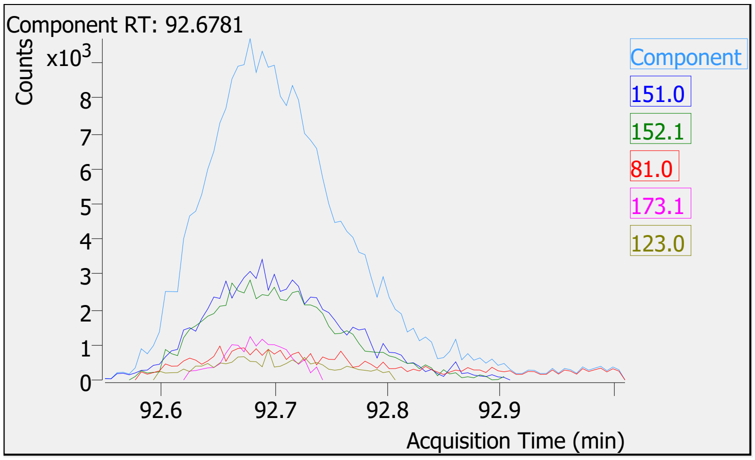

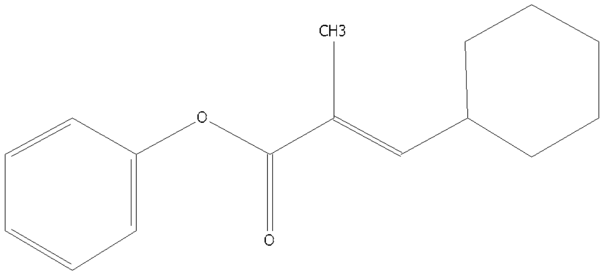

Generated at 4:32 PM on 11/14/2023

RT	Compound Name	CAS#	Formula	Area	MI	Match Score	Sample	Sample
93.8068	3-(3-Methylphenyl)-pyridine	990030-50-7	C12H11N	151128		91.5	1.16	4.66

Component RT: 93.8068

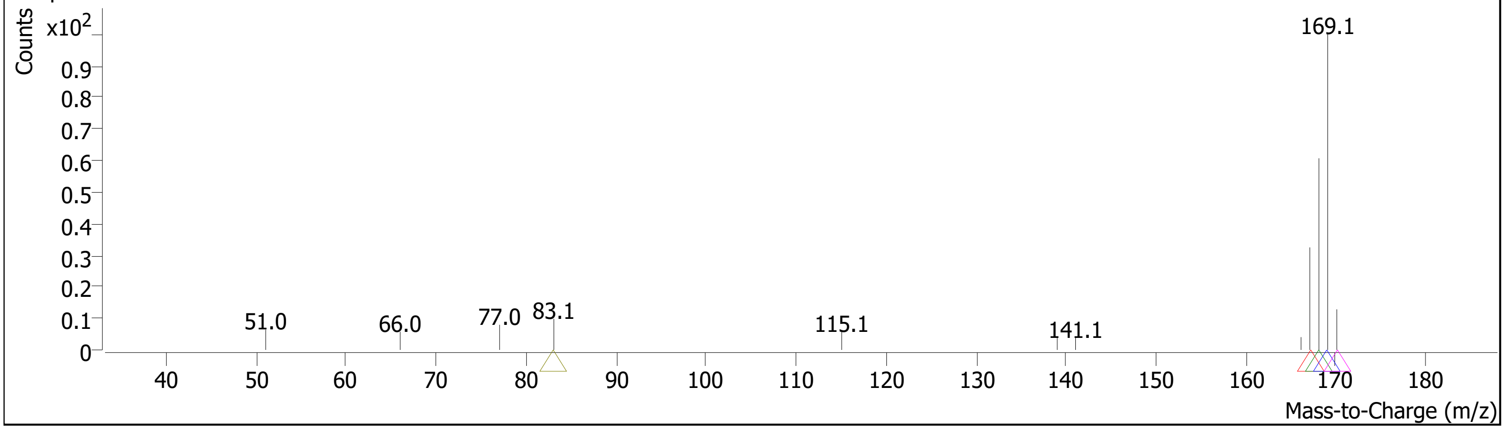

3-(3-Methylphenyl)-pyridine (W12N20_MAIN.L)

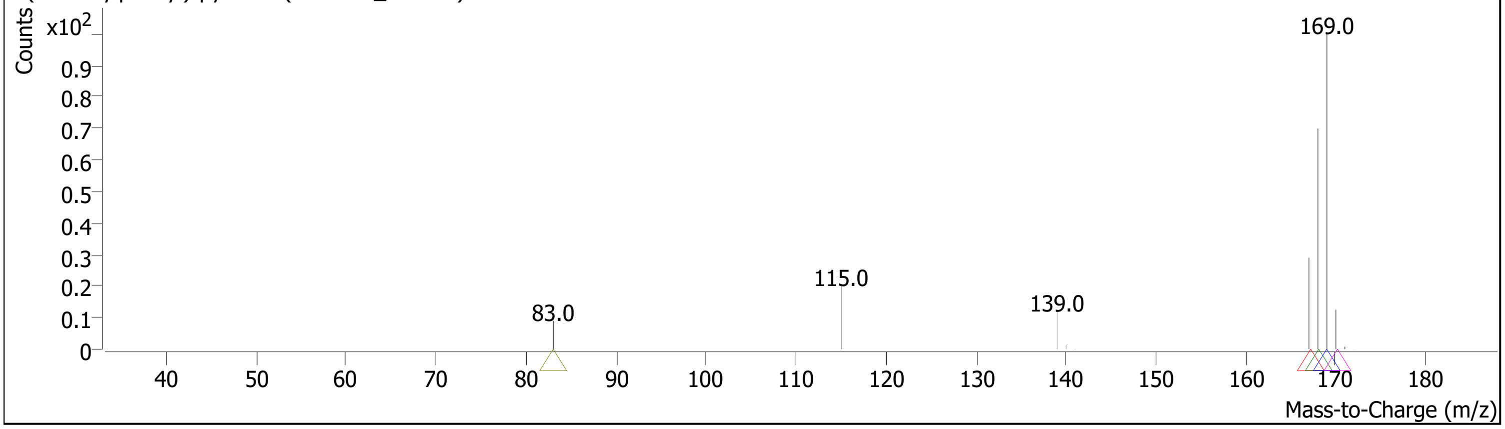

+ Scan (93.7215-93.8873 min, 32 scans) 11795-2.D

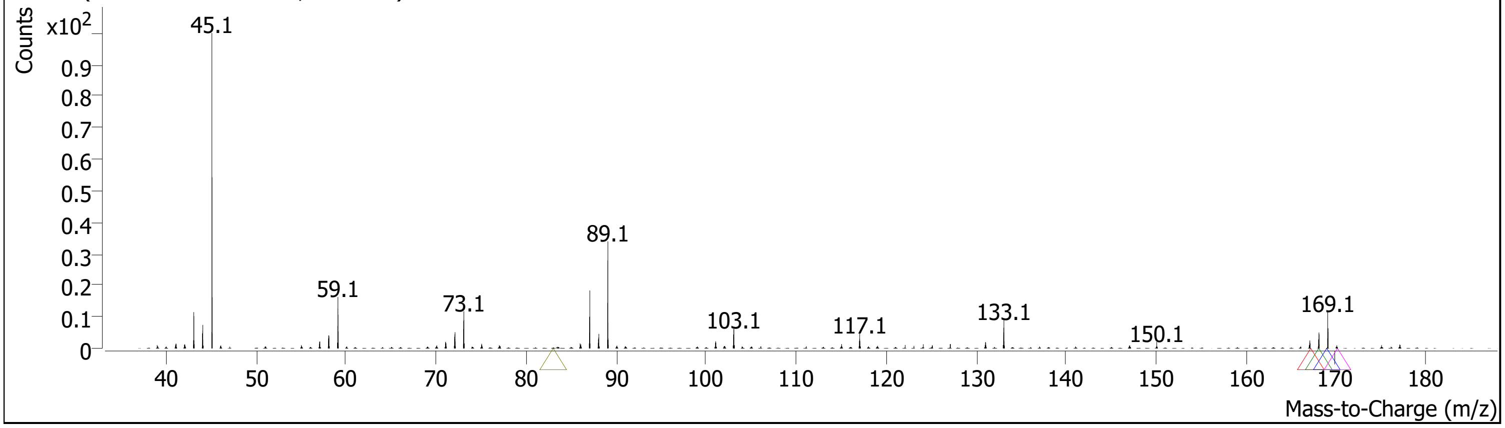

Component RT: 93.8068

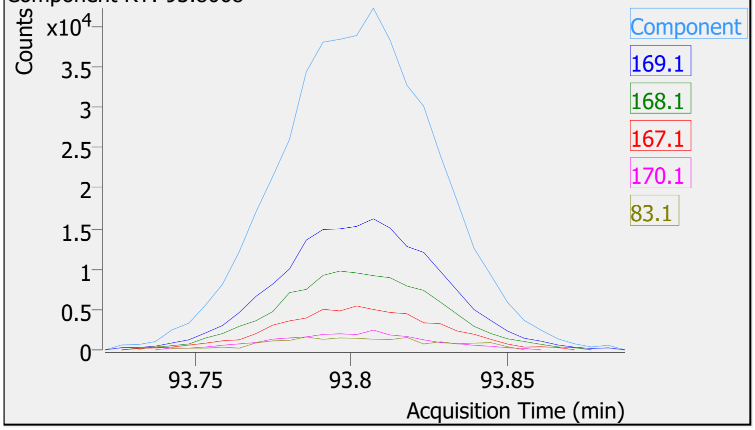

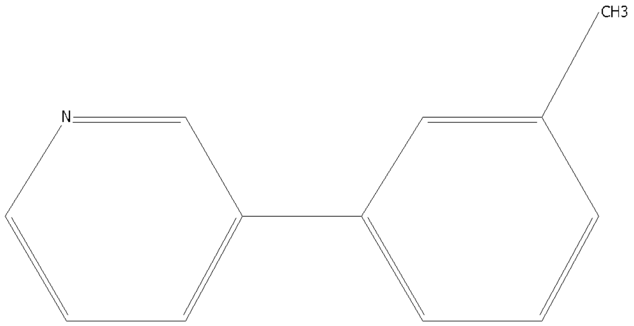

RT	Compound Name	CAS#	Formula	Area	MI	Match Score	Sample	Sample
94.5309	3-(Benzyloxy)-1-propanal	990024-51-7	C10H12O2	210048		75.4	1.62	6.48

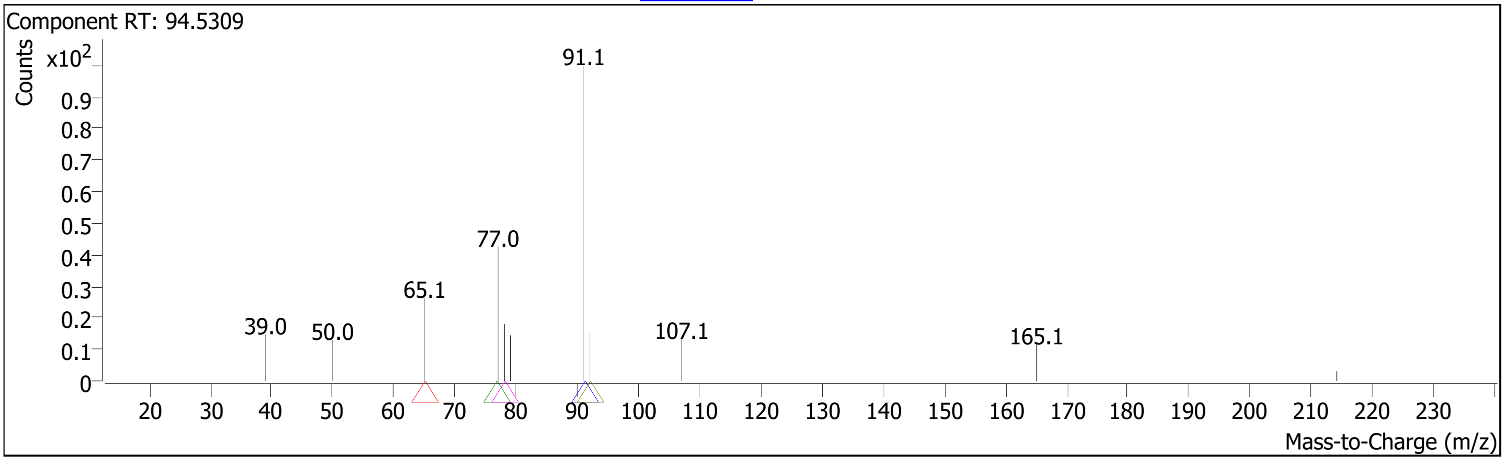

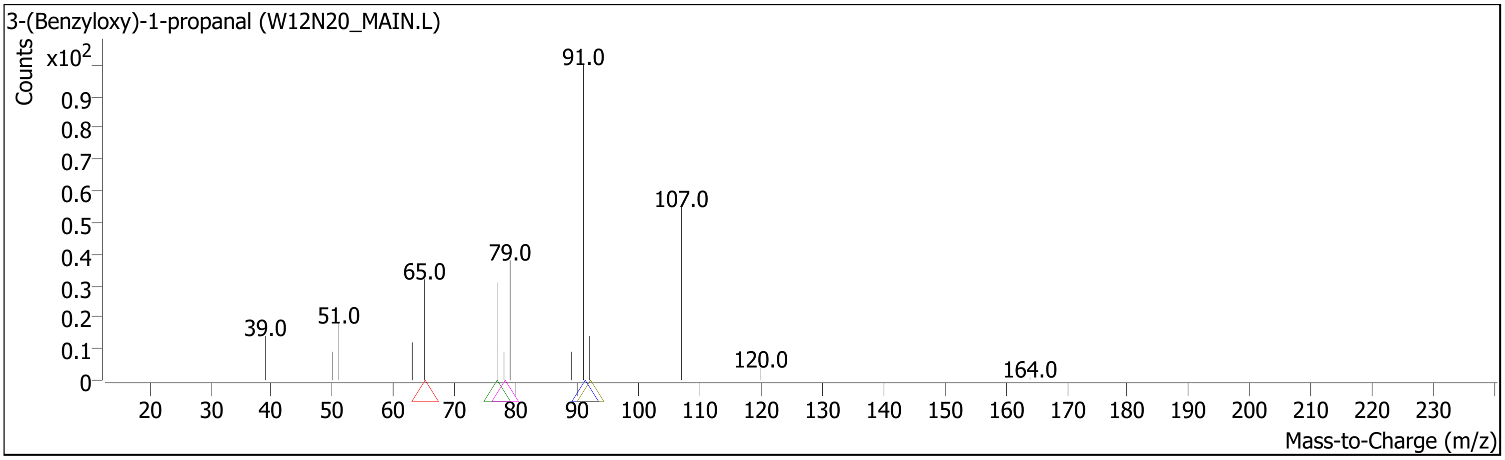

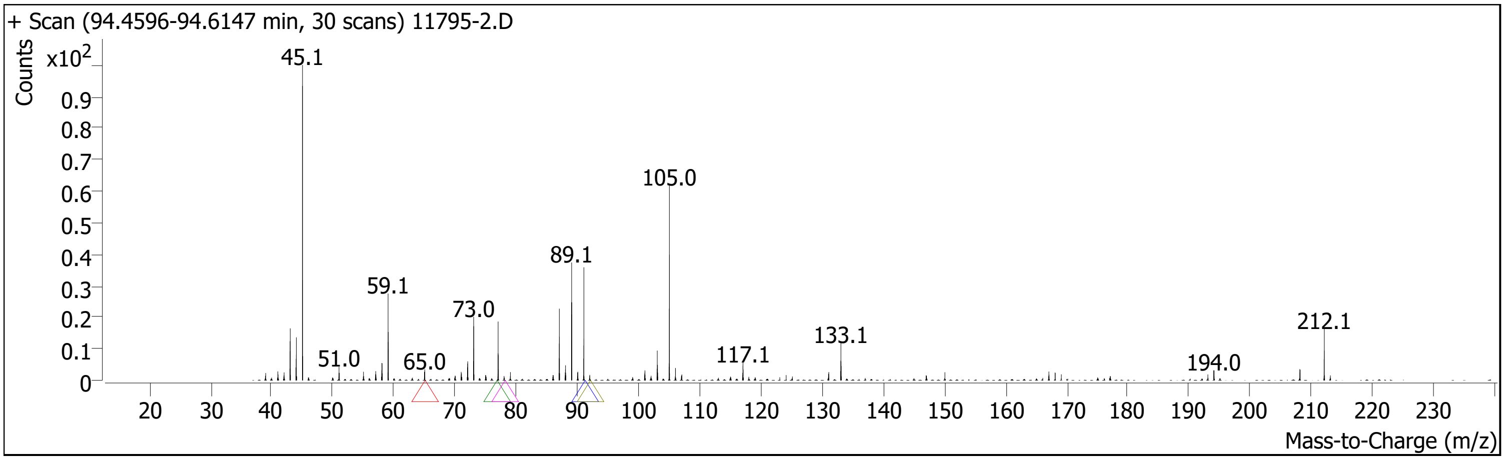

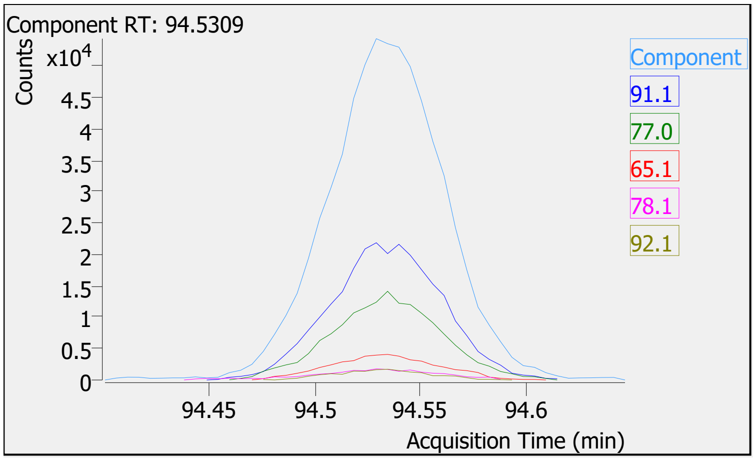

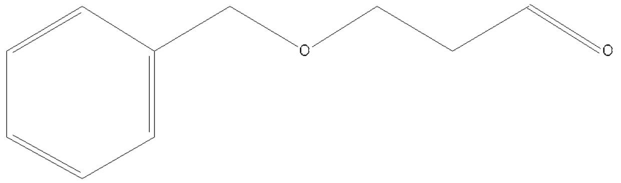

Unknown Analysis Report - Best Hits

Agilent Trusted Answers

RT	Compound Name	CAS#	Formula	Area	MI	Match Score	Sample	Sample
94.5381	Benzyl Benzoate	120-51-4	C14H12O2	540862		95.5	4.16	16.67

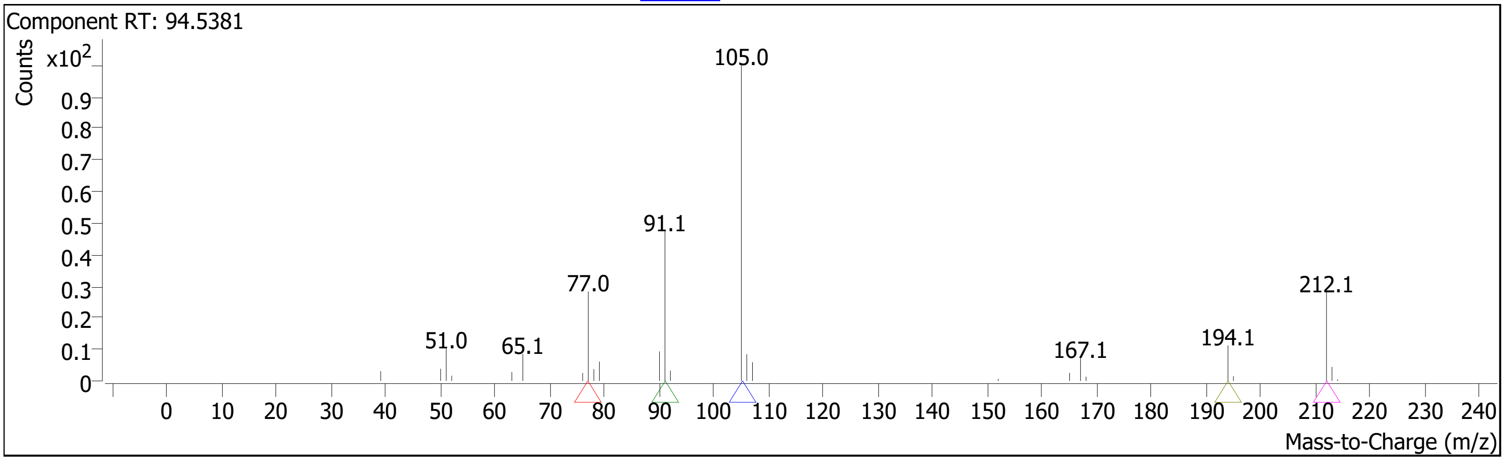

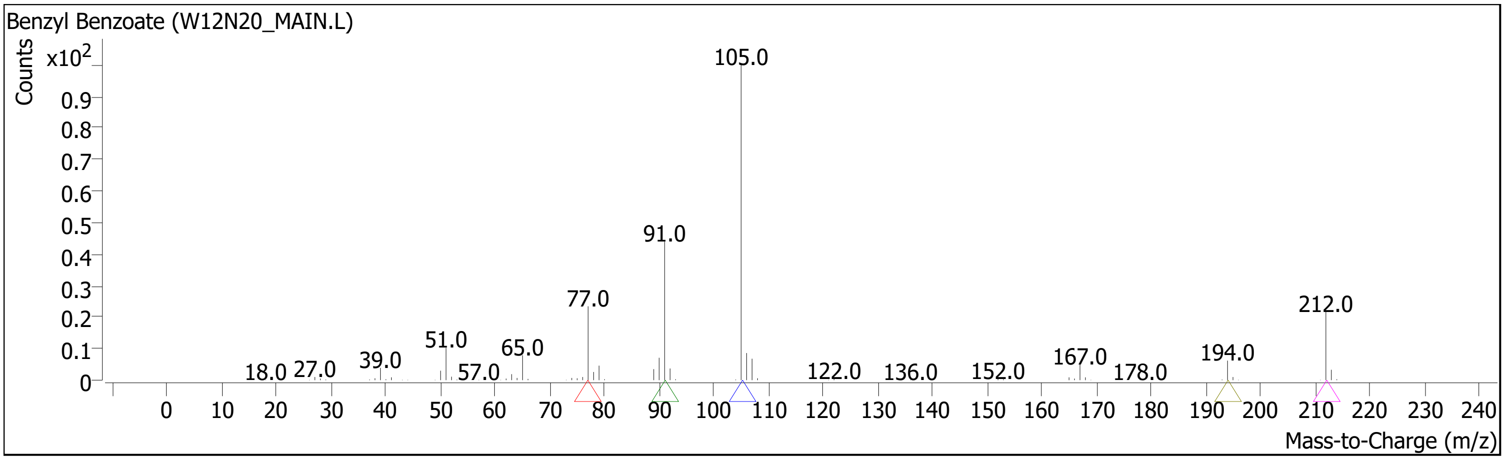

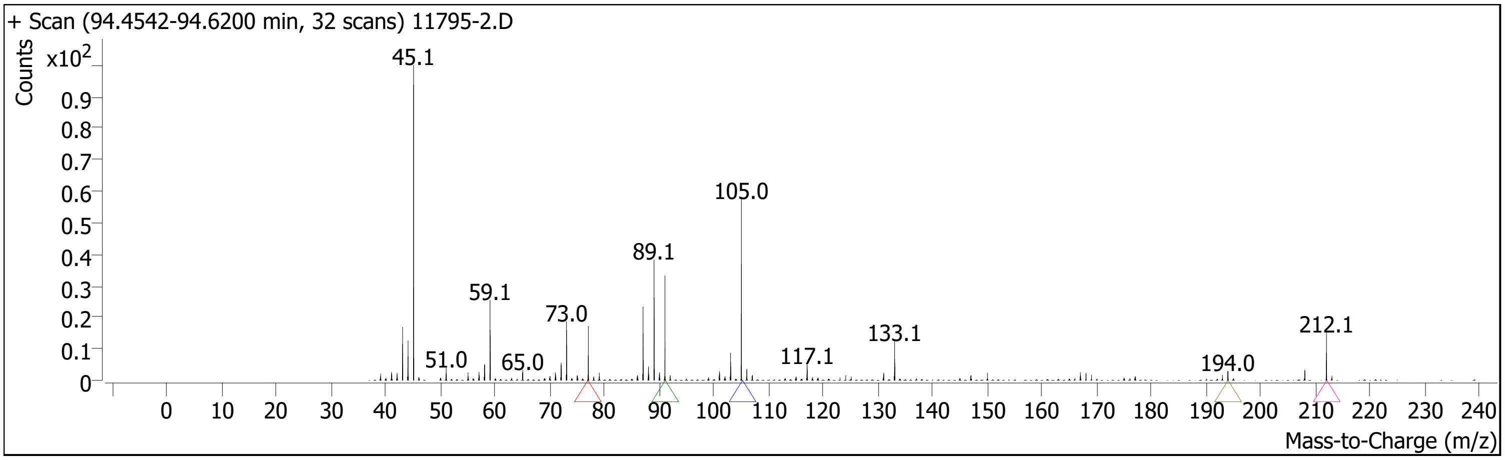

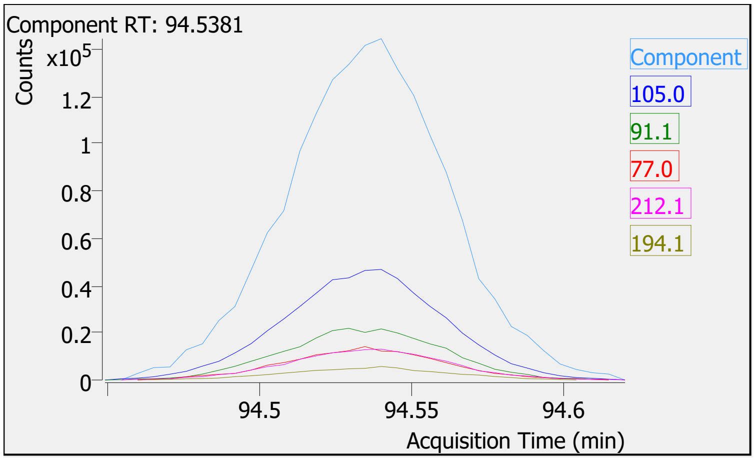

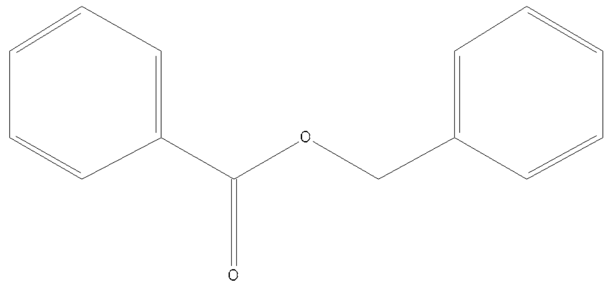

Unknown Analysis Report - Best Hits

RT	Compound Name	CAS#	Formula	Area	MI	Match Score	Sample	Sample
96.4923	Spiro[cyclopentane-1,1'(2'H)-naphthalene]-5'-ethanamine, N-ethyl-7',8'-dimethoxy-N-methyl-	63080-55-7	C21H31NO2	818688		79.4	6.30	25.24

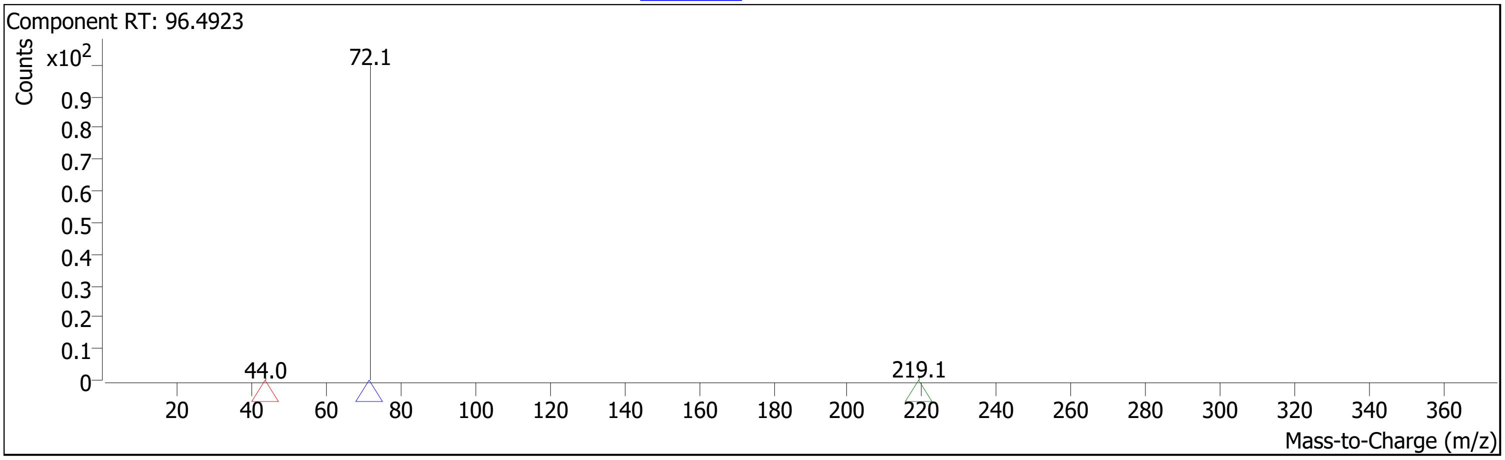

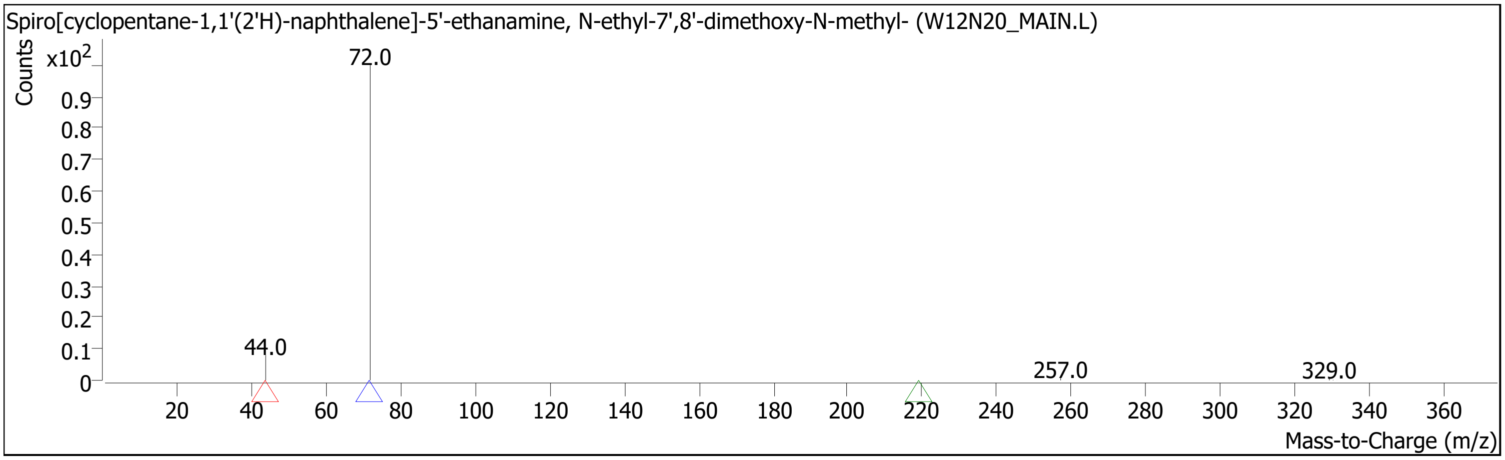

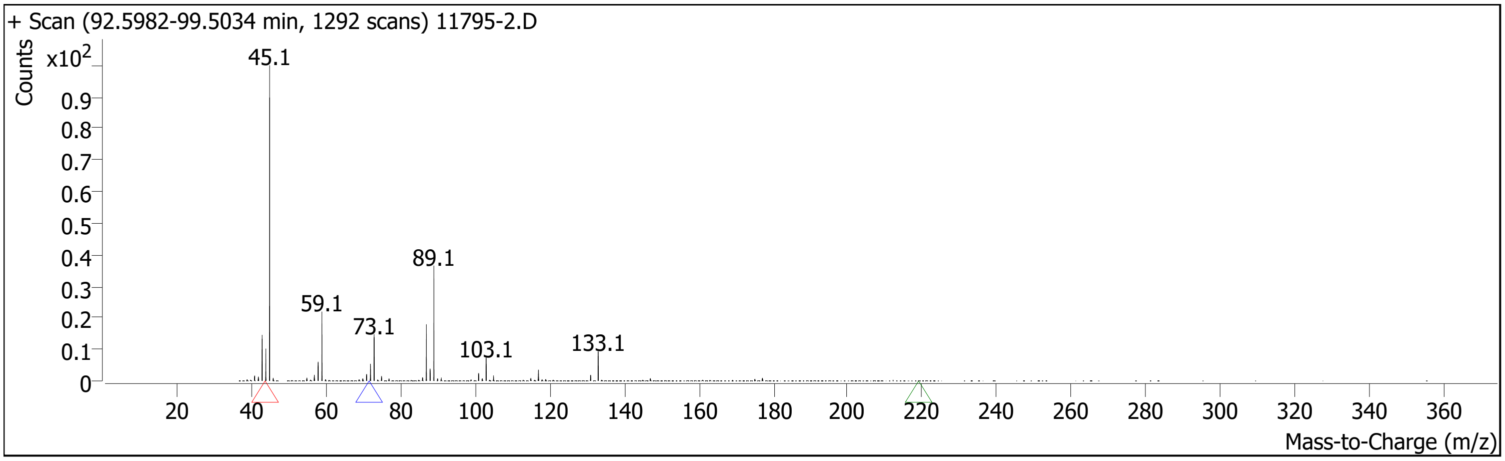

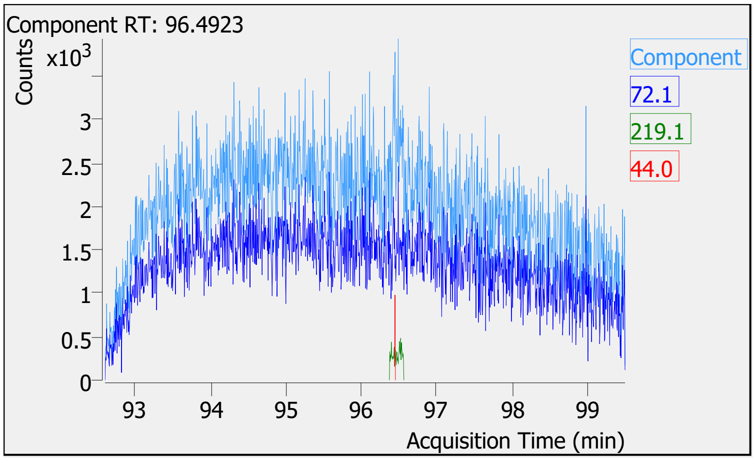

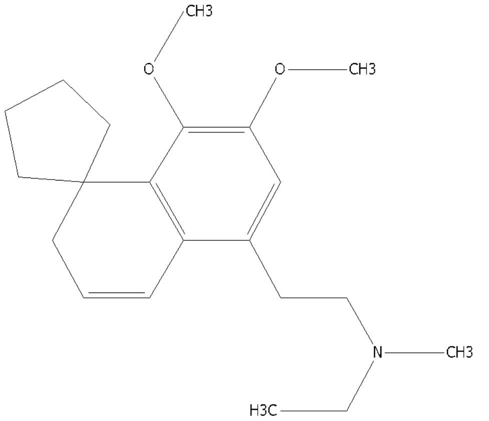

 Generated at 4:32 PM on 11/14/2023

Unknown Analysis Report - Best Hits

RT	Compound Name	CAS#	Formula	Area	MI	Match Score	Sample	Sample
100.5718	(2R)-2-(6-methoxy-2-naphthalenyl)propanenitrile	108865-01-6	C14H13NO	23118		76.8	0.18	0.71

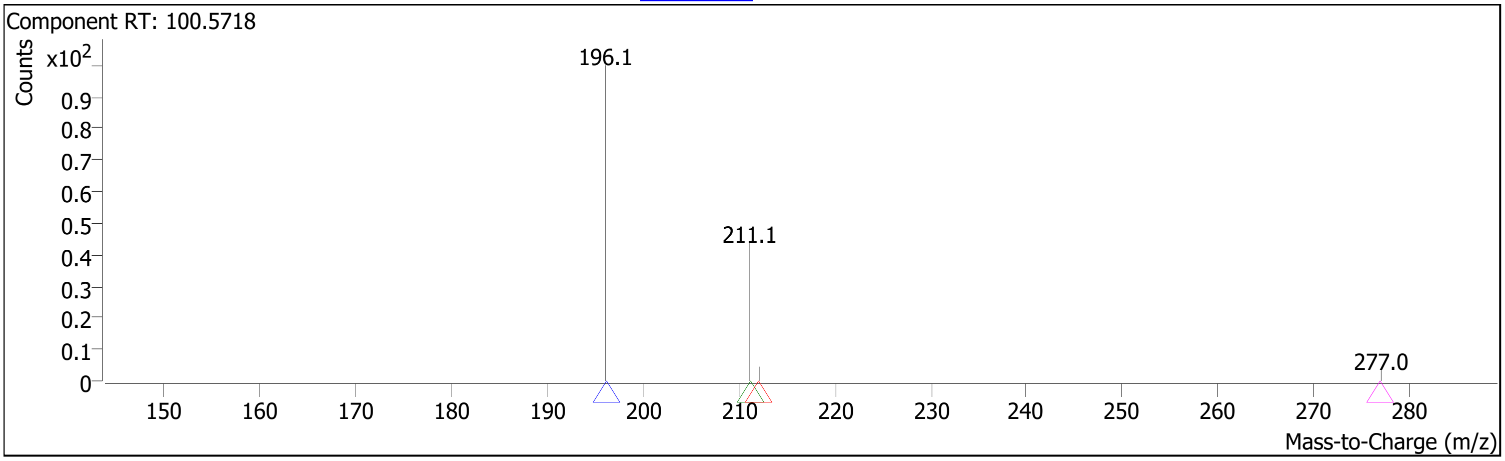

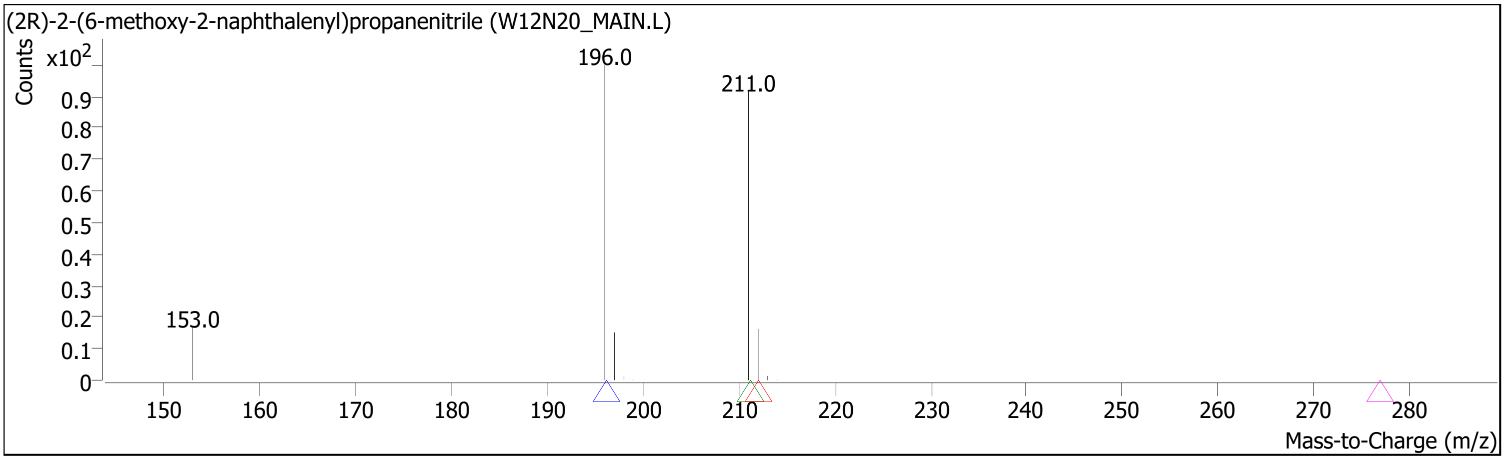

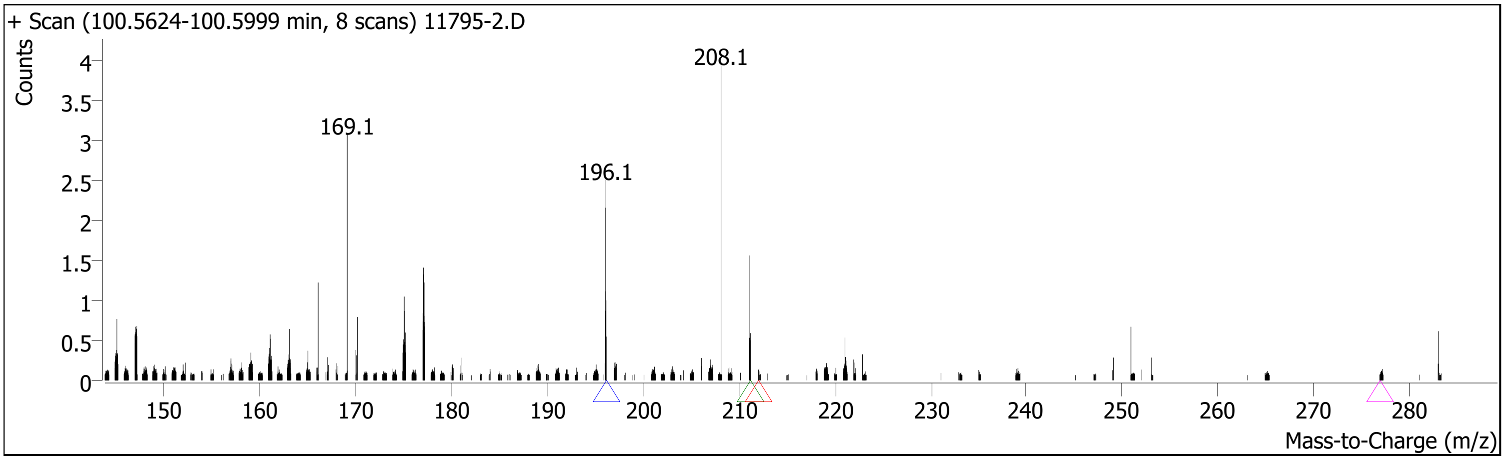

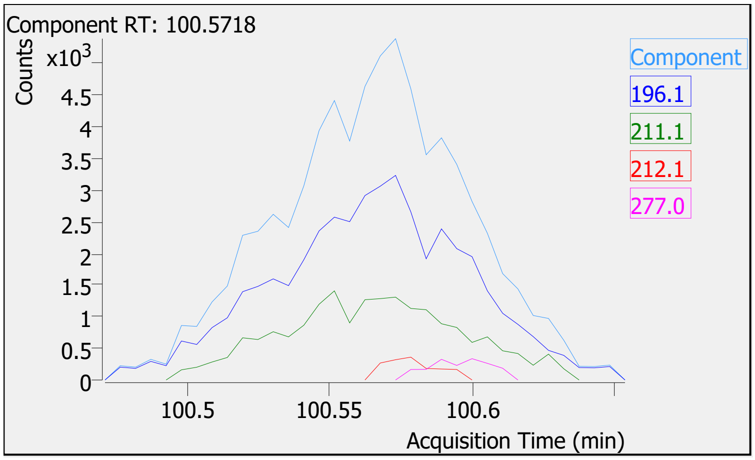

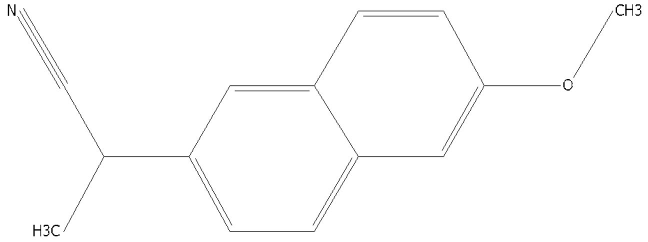

Generated at 4:32 PM on 11/14/2023
